# Morpho-biometric characterization of indigenous chicken ecotypes in north-western Ethiopia

**DOI:** 10.1371/journal.pone.0286299

**Published:** 2023-06-02

**Authors:** Bekalu Muluneh, Mengistie Taye, Tadelle Dessie, Dessie Salilew Wondim, Damitie Kebede, Andualem Tenagne

**Affiliations:** 1 Department of Animal Science, College of Agriculture and Environmental Sciences, Bahir Dar University, Bahir Dar, Ethiopia; 2 Department of Animal and Range Sciences, Dawuro Tarcha Campus, Wolaita Sodo University, Sodo, Ethiopia; 3 Institute of Biotechnology, Bahir Dar University, Bahir Dar, Ethiopia; 4 International Livestock Research Institute (ILRI), Addis Ababa, Ethiopia; 5 Department of Animal Breeding and Husbandry, Institute of Animal Sciences, University of Bonn, Bonn, Germany; 6 Department of Animal Sciences, Assosa University, Assosa, Ethiopia; Tokai University School of Medicine, JAPAN

## Abstract

Morphological characterization of Animal Genetic Resources is the first step to documenting diversity and designing breed specific breeding programs. The current study characterized the morpho-biometric variation of indigenous chicken ecotypes prevailing in northwestern Ethiopia. A multi-stage purposive, stratified, and random sampling method was employed to select the study areas and chickens. A total of 1200 adult chickens were sampled and characterized for 12 qualitative and 11 quantitative traits. Univariate and multivariate data analysis methods were employed to analyze the data using SAS and R statistical software. Red plumage colour (33.2%), white and red earlobe colour (73.8%) and yellow shank colour (57.0%) were the most predominant colour trait categories. Sex, agro-ecology, location, and the interaction of sex and location had a highly significant (p<0.001) effect on all body measurements. Shank traits were found to have the highest discriminating power in both sexes. The overall classification rates for the female and male sample populations were 57.47% and 69.97%, respectively. The squared Mahalanobis distances between sites were significant (p<0.001) for both sexes. The longest distance was obtained between North Achefer and Banja (19.25) and between North Achefer and Dembecha (16.80) in female and male chickens, respectively. In female chickens, canonical variates 1 (CAN 1) and 2 (CAN 2) explained 82% of total variation and distinctly separated the sample populations of North Achefer and Jawi from others. In male chickens, 90% of the total variance is explained by CAN1, CAN2, and CAN3, which distinctly separate the sample populations of the North Achefer, Sinan, and Jawi, among others. Using cluster analysis, the indigenous chickens found in the study area could be classified into four ecotypes: ecotype 1 (Banja, Dembecha, and Aneded), ecotype 2 (North Achefer), ecotype 3 (Sinan), and ecotype 4 (Jawi).

## Introduction

Indigenous chicken production is a common practice in rural, resource-poor households in developing countries like Ethiopia. The majority of families at the village level, including the landless and the poorest, are owners of poultry [1]. According to a report [2], the total poultry population in Ethiopia is estimated to be about 56.99 million, most of which are laying hens (34.26%) followed by chicks (32.86%). With regard to breed, 78.85%, 9.14%, and 12.03% of the total poultry were reported to be indigenous, exotic, and hybrid, respectively. Although indigenous chicken breeds are claimed to be slow growers and poor producers of small eggs, they are characterized by better egg and meat flavour, top brooding and natural incubation capacity, and high dressing percentages [1]. Characterization and identification of the chicken genetic resources require information on the population, adaptation to a specific environment, possession of traits of future value, and their socio-cultural importance [3]. Dorji and Sunar [4] stated that phenotypic approaches are fundamental in chicken breed management because they are fast, easy, and cost-effective. Indigenous chickens have various unique morphological features and possess genes that have adaptive values for their environment and local diseases [5]. Local chicken populations are often described and grouped based on their location or phenotypic features, while their classification into breeds is inadequate [6]. In the literature, the terms "ecotype" and "breed" are used interchangeably. "Ecotype" is described in evolutionary ecology as a population that is genetically adapted to specific environmental conditions [7]. On the other hand, ’’ecotype is described as "a non-static adaptive variation over many traits across the natural landscape with no discernible boundaries" [8]. The terms ’indigenous’, ’native’, ’local’ or ’traditional’ are also used interchangeably. For instance, Tadelle *et al* [9] used “local chicken ecotypes” while Halima *et al* [10] called them “native chicken populations”. The indigenous chickens of Ethiopia are named after the location where they are prevalent including Jarso, Chefe, Tilili, Tepi and Horro [9]; Guangua, Tilili, Debre-Elias, Melo-Hamusit, Gelila, Gassay and Mecha [10]; Mandura, Horro, Farta, Konso and sheka [11]; Gasgie, and Gugut [12]. Based on their plumage colour type, scholars named local chicken as *Gebsima*, Red, White, Black, Grey, *Kokima*, *Key Teterima*, *Lebework*, *Tikur Gebsat*, Brown and *Netch teterima* [13]. In Ethiopia, several scholars conducted phenotypic characterization of indigenous chickens [7–15]. However, there is still a dearth of information in the Northwestern parts of Ethiopia, where the presence of huge genetic resources is anticipated. Therefore, this study was aimed at characterizing the morpho-biometric variation of indigenous chicken ecotypes found in Northwest Ethiopia as an input for designing breeding and conservation programs.

## Material and methods

### Ethics approval and consent to participate

This study was reviewed and approved by Bahir Dar University College of Agriculture and Environmental Science (BDU-CAES). Following endorsement by the BDU-CAES, the Bahir Dar University department of Animal Science wrote a support letter (dated on February 24, 2020) about the objectives of the study for research site agricultural and development offices. In addition, chicken owners provided their verbal informed consent to participate in this study.

### Description of the study areas

This study was conducted in three zones (Awi zone, West Gojjam zone, and East Gojjam zone) of the Amhara National Regional State, Ethiopia. Banja and Jawi districts from the Awi administrative zone, Sinan and Aneded from the East Gojjam zone, and Dembecha and North Achefer from the West Gojjam zone were considered for the study. The agro-ecological description, the number of indigenous chickens found in the study area, and the major feed resources for the chickens are presented in Table 1.

**Table 1:** Agro-ecological description, number of chickens, and major feed resources for chickens in northwest Ethiopia.

District/site	*Kebele*	Agro-ecology	Altitude (m.a.s.l.)	Annual rainfall (mm)	Annual temperature (°C)	Indigenous Chicken number	Major feed resources for chickens
**Banja**	1	Highland	3028	2200–2560	7–25	36,894	wheat, maize, barley, oat, *injera*
	2	Highland	2685				
	3	Highland	2723				
**Jawi**	1	Lowland	995	650–1250	12–40	256,000	sorghum, finger-millet, maize, rice, *Gobe*, groundnut
	2	Lowland	1171				
	3	Lowland	1365				
**Sinan**	1	Highland	3214	900–1500	0–15	19,652	barley, maize, wheat
	2	Highland	3192				
	3	Highland	3081				
**Aneded**	1	Midland	2203	1200–1660	10–23	48,440	maize, wheat, *injera*, barley, *Engido*
	2	Midland	2071				
	3	Midland	2142				
**Dembecha**	1	Midland	1857	980–1100	18–27	113,219	wheat, maize, barley, figer-millet, *teff*
	2	Midland	2287				
	3	Midland	1979				
**North Achefer**	1	Lowland	1480	1100–1420	23–33	248,671	finger-millet, maize, sorghum, *injera*
	2	Lowland	1495				
	3	Lowland	1386				

Source: districts agricultural bureau, 2021; m.a.s.l. = meter above sea level

### Sampling and data collection

A multi-stage purposive, random, and stratified sampling method was employed for the selection of zones, districts, households, and chickens for the study. In the first stage, three zones (Awi zone, East Gojjam zone, and West Gojjam zone) were purposefully selected based on research gaps and their location (surrounded by *Abay* Gorge). In the second stage, districts and *kebeles/*peasant associations were stratified based on agro-ecology, and six districts (two districts from each agro-ecology and three *kebeles* from each district) were selected considering their chicken population numbers (Table 1). Banja and Sinan from highland agro-ecology, Dembecha and Aneded from midland agro-ecology, and Jawi and North Achefer from lowland agro-ecology were considered. At the third stage, households that have only indigenous chickens were purposefully selected to avoid biases during the measurement of chickens. Last, chickens (200 chickens from each district) were randomly sampled based on the FAO guidelines for phenotypic characterization [14]. Accordingly, a total of 1200 adult chickens older than seven months (877 females and 323 males) were sampled. While sampling chickens from each household, the distance between households was considered to minimize the error, which might come due to the genetic relatedness between chickens. Each chicken was identified by its sex and location.

**Fig 1:**
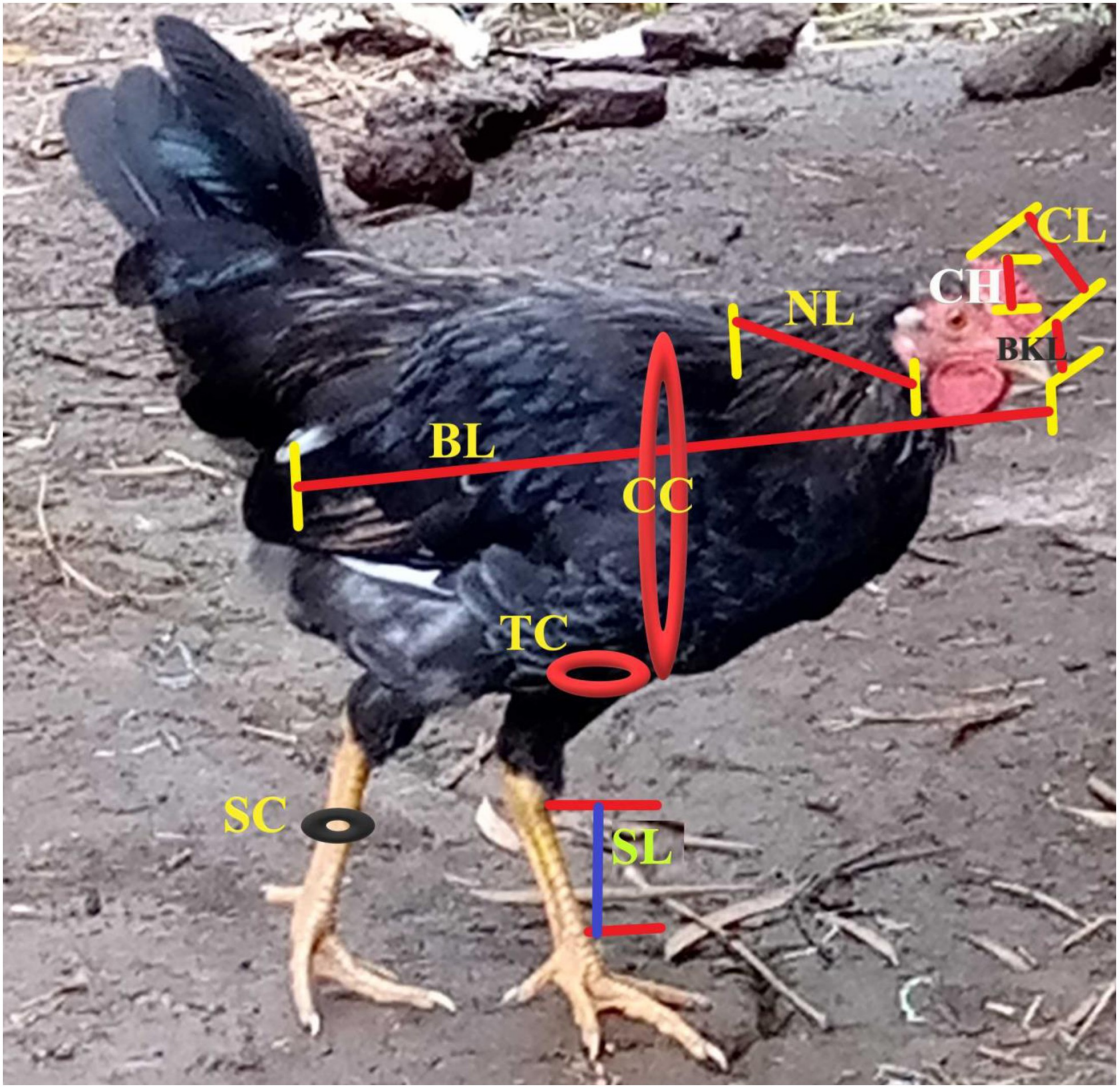
Location of linear body measurements (measurement points).

The chicken were characterized for ten linear body measurements: wing span (WS), shank length (SL), body length (BL), beak length (BKL), neck length (NL), comb length (CL), shank circumference (SC), chest circumference (CC), thigh circumference (TC), and comb height (CH) using a textile measuring tape (Fig 1) and body weight was measured using a suspended spring. A visual observation was made, and 12 morphological features (plumage colour pattern, plumage colour type, shank colour, earlobe colour, skin colour, earlobe presence, wattle presence, shank feather presence, comb type, head shape, feather morphology, and feather distribution) were also recorded based on the FAO breed morphological characteristics descriptor list [14].

### Statistical analysis

All data were coded and recorded in a Microsoft Excel sheet and saved as a “csv” file. Statistical analyses were made via SAS [15]. R-software [16] was used for graphical presentation/data visualization. Homogeneity tests, normality tests, and screening of outliers were employed before conducting the main data analysis work.

#### Qualitative morphological traits analysis

*Univariate analysis*. Qualitative morphological traits were subjected to the frequency procedure of the chi-square (χ^2^) test to assess the statistical significance among qualitative variables.

*Multivariate analysis*. The associations among qualitative morphological traits were assessed via a multiple correspondence analysis. "FactoMineR" and "factoextra" packages were used for analysis and plot visualization.

#### Quantitative morphological traits analysis

*Univariate analysis*. Quantitative morphological traits were analyzed using the general linear model procedure to determine the effects of agro-ecology, location, sex, and location with sex interaction. Agro-ecology, location, and sex of the indigenous chickens were fitted as fixed independent variables. The effects of class variables and their interaction were expressed as least square means (LSM) ± SE. Mean comparisons were made using Tukey’s studentized range test method at *P*<0.05. The following model was utilized to show the effect of agro-ecology, location, and sex on different linear body measurements and body weight of indigenous chickens.

$${\text{Y}}_{\text{ijk}}=\mu +{\text{A}}_{\text{i}}+{\text{L}}_{\text{j}}+{\text{S}}_{\text{k}}+{\left(\text{LS}\right)}_{\text{jk}}+{\text{e}}_{\text{ijk}},$$

Where:

Y_ijk_ = individual phenotypic observation;

μ = the overall mean;

A_i_ = the fixed effect of agro-ecology (i = Highland, Midland, and Lowland);

L_j_ = the fixed effect of location (j = Banja, Sinan, Dembecha, Aneded, Jawi and North Achefer);

S_k_ = the fixed effect of sex (k = Male and Female);

LS_jk_ = the interaction effect of location with sex and

e_ijk_ = the effect of random error.

*Multivariate analysis*. The stepwise discriminant analysis procedure was run to rank the quantitative morphological traits by their discriminating power. Selected significant traits from stepwise discriminant analysis were then subjected to canonical discriminant analysis. The quantitative variables from male and female chickens were separately subjected to discriminate analysis to ascertain the existence of population-level phenotypic differences among them. Non-parametric discriminant analysis was also done for male and female chickens together to classify them based on qualitative traits. The degree of morphological similarity between the chickens was determined using Euclidean and Ward’s option of R in hierarchical clustering and hierarchical clustering on principal components analysis (HCPC). “Cluster”, “factoextra” and “FactoMineR” packages were utilized in the clustering procedures. The hierarchical clustering on principal components analysis was done by using the quantitative traits, which have high discriminating power in the classification of chickens. In clustering of female chickens, wing span, shank length, beak length, neck length, shank circumference, and chest circumference were considered. In male chickens, wing span, shank length, shank circumference, comb length, body weight, and chest circumference were included.

## Results

### Qualitative morphological traits

The frequency and percent of qualitative traits observed in male and female chickens are presented in Table 2. The most frequently observed plumage colour pattern in the study area was plain (55.3%), followed by patchy (34.8%). Red and *Gebisma* (white strips on black background, grayish with varying mixtures, mixtures of white and black with varying shades of multicolours and red brownish with black) were the most frequently observed plumage colour types in almost all study areas, with the overall values of 33.2% and 30.1%, respectively. The dominant shank colours found in the study areas were yellow (57%) and white (32.9%). Some of the indigenous chickens, around 1%, had uncommon shank colour, such as blue and green. About 70.8% of indigenous chickens have earlobes, and white with red earlobe colour (73.8%) was mostly observed in the study area, followed by red earlobe colour (18.4%).

**Table 2:** Qualitative morphological traits characteristics of indigenous chicken ecotypes in north western Ethiopia.

Morphological character	Locations/sites												
	Banja		Sinan		Dembecha		Aneded		Jawi		North Achefer		Overall
	M	F	M	F	M	F	M	F	M	F	M	F	N (%)
	N (%)	N (%)	N (%)	N (%)	N (%)	N (%)	N (%)	N (%)	N (%)	N (%)	N (%)	N (%)	
**Plumage colour pattern**													
Plain	39(78)	99(59.6)	50(79.4)	79(49.7)	25(58.1)	82(62.6)	44(80.0)	72(47.1)	45(75.0)	57(39.0)	40(76.9)	31(25.4)	663(55.3)
Patchy	8(16)	50(30.1)	11(17.5)	68(42.8)	14(32.6)	29(22.1)	5(9.1)	68(44.4)	10(16.7)	57(39.0)	12(23.1)	86(70.5)	418(34.8)
Spotted	3(6)	17(10.2)	2(3.2)	12(7.5)	4(9.3)	20(15.3)	6(10.9)	13(8.5)	5(8.3)	32(21.9)	0(0.0)	5(4.1)	119(9.9)
*χ*^*2*^ *= 72*.694***; *Phi = 0*.*246; Cramer’s V = 0*.*174*													
**Plumage colour type**													
Completely white	9(18.0)	36(21.7)	0(0.0)	8(5.0)	0(0.0)	24(18.3)	10(18.2)	30(19.6)	0(0.0)	5(3.4)	0(0.0)	5(4.1)	127(10.6)
Completely black	8(16.0)	22(13.3)	4(6.3)	19(11.9)	0(0.0)	0(0.0)	5(9.1)	10(6.5)	0(0.0)	20(13.7)	0(0.0)	0(0.0)	88(7.3)
Completely red	22(44.0	41(24.7)	33(52.4)	43(27.0)	25(58.1)	38(29.0)	25(45.5)	21(13.7)	45(75.0)	27(18.5)	46(88.5)	32(26.2)	398(33.2)
Gebisma	8(16.0)	50(30.1)	11(17.5)	75(47.2)	10(23.3)	29(22.1)	5(9.1)	67(43.8)	5(8.3)	42(28.8)	6(11.5)	53(43.4)	361(30.1)
Multi-colour	0(0.0)	11(6.6)	0(0.0)	0(0.0)	4(9.3)	14(10.7)	5(9.1)	6(3.9)	0(0.0)	15(10.3)	0(0.0)	22(18.0)	77(6.4)
Teterima	3(6.0)	6(3.6)	6(9.5)	7(4.4)	0(0.0)	6(4.6)	0(0.0)	6(3.9)	10(16.7)	37(25.3)	0(0.0)	10(8.2)	91(7.6)
Wosera	0(0.0)	0(0.0)	5(7.9)	7(4.4)	4(9.3)	20(15.3)	5(9.1)	13(8.5)	0(0.0)	0(0.0)	0(0.0)	0(0.0)	54(4.5)
Sora	0(0.0)	0(0.0)	4(6.3)	0(0.0)	0(0.0)	0(0.0)	0(0.0)	0(0.0)	0(0.0)	0(0.0)	0(0.0)	0(0.0)	4(0.3)
*χ*^*2*^ *= 339*.85***; *Phi = 0*.*532; Cramer’s V = 0*.*238*													
**Shank colour**													
Yellow	50(100)	98(59.0)	51(81.0)	65(40.9)	29(67.4)	32(24.4)	55(100)	115(75.2)	45(75.0)	43(29.5)	46(88.5)	55(45.1)	684(57.0)
Black	0(0.0)	26(15.7)	5(7.9)	21(13.2)	0(0.0)	0(0.0)	0(0.0)	15(9.8)	0(0.0)	31(21.2)	0(0.0)	11(9.0)	109(9.1)
White	0(0.0)	42(25.3)	7(11.1)	73(45.9)	14(32.6)	99(75.6)	0(0.0)	23(15)	15(25)	72(49.3)	0(0.0)	50(41.0)	395(32.9)
Others	0(0.0)	0(0.0)	0(0.0)	0(0.0)	0(0.0)	0(0.0)	0(0.0)	0(0.0)	0(0.0)	0(0.0)	6(11.5)	6(4.9)	12(1.0)
*χ*^*2*^ *= 254*.32***; *Phi = 0*.*46; Cramer’s V = 0*.*266*													
**Earlobe colour**													
White	0(0.0)	0(0.0)	0(0.0)	6(7.0)	0(0.0)	0(0.0)	0(0.0)	0(0.0)	0(0.0)	16(20.5)	0(0.0)	44(45.8)	66(7.8)
Red	24(59.0)	0(0.0)	24(45.0)	0(0.0)	12(28.0)	0(0.0)	24(43.6)	8(7.0)	10(18.1)	0(0.0)	47(90.4)	7(7.2)	156(18.4)
White and red	17(41.0)	89(100)	29(55.0)	80(93.0)	31(72.0)	86(100)	31(56.4)	107(93.0)	45(81.9)	62(79.5)	5(9.6)	45(47.0)	627(73.8)
*χ*^*2*^ *= 295*.24***; *Phi = 0*.*496; Cramer’s V = 0*.*286*													
**Skin colour**													
White	36(72.0)	138(83.1)	35(55.6)	154(96.9)	14(32.6)	131(100)	35(63.6)	145(94.8)	60(100)	146(100)	52(100)	115(94.3)	1061(88.4)
Yellow	11(22.0)	17(10.2)	28(44.4)	5(3.1)	29(67.4)	0(0.0)	20(36.4)	8(5.2)	0(0.0)	0(0.0)	0(0.0)	0(0.0)	118(9.8)
Others	3(6.0)	11(6.6)	0(0.0)	0(0.0)	0(0.0)	0(0.0)	0(0.0)	0(0.0)	0(0.0)	0(0.0)	0(0.0)	7(5.7)	21(1.8)
*χ*^*2*^ *= 109*.85***; *Phi = 0*.*30; Cramer’s V = 0*.*214*													
**Earlobe**													
Present	41(82.0)	89(53.6)	53(84.1)	86(54.1)	43(100)	86(65.6)	55(100)	115(75.2)	55(91.7)	78(53.4)	52(100)	96(78.7)	849(70.8)
Absent	9(18.0)	77(46.4)	10(15.9)	73(45.9)	0(0.0)	45(34.4)	0(0.0)	38(24.8)	5(8.3)	68(46.6)	0(0.0)	26(21.3)	351(29.3)
*χ*^*2*^ *= 52*.86***; *Phi = 0*.*21; Cramer’s V = 0*.*21*													
**Wattle**													
Present	50(100)	166(100)	63(100)	159(100)	43(100)	131(100)	55(100)	153(100)	55(91.7)	146(100)	52(100)	122(100)	1195(99.6)
Absent	0(0.0)	0(0.0)	0(0.0)	0(0.0)	0(0.0)	0(0.0)	0(0.0)	0(0.0)	5(8.3)	0(0.0)	0(0.0)	0(0.0)	5(0.4)
*χ*^*2*^ *= 24*.23***; *Phi = 0*.*142; Cramer’s V = 0*.*142*													
**Shank feather**													
Present	0(0.0)	3(1.8)	0(0.0)	0(0.0)	7(16.3)	10(7.6)	2(3.6)	0(0.0)	0(0.0)	1(0.7)	0(0.0)	0(0.0)	23(1.9)
Absent	50(100)	163(98.2)	63(100)	159(100)	36(83.7)	121(92.4)	53(96.4)	153(100)	60(100)	145(99.3)	52(100)	122(100)	1177(98.1)
*χ*^*2*^ *= 68*.39***; *Phi = 0*.*239; Cramer’s V = 0*.*239*													
**Comb type**													
Rose	38(76.0)	55(33.1)	33(52.4)	61(38.4)	29(67.4)	59(45.0)	25(45.5)	51(33.3)	25(41.7)	49(33.6)	13(25.0)	16(13.1)	454(37.8)
Pea	0(0.0)	45(27.1)	3(4.8)	14(8.8)	0(0.0)	26(19.8)	0(0.0)	39(25.5)	5(8.3)	56(38.4)	0(0.0)	27(22.1)	215(17.9)
Single	12(24.0)	51(30.7)	21(33.3)	47(29.6)	4(9.3)	39(29.8)	25(45.5)	44(28.8)	15(25.0)	25(17.1)	23(44.2)	52(42.6)	358(29.8)
Others	0(0.00)	15(9.0)	6(9.5)	37(23.3)	10(23.3)	7(5.3)	5(9.1)	19(12.4)	15(25.0)	16(11.0)	16(30.8)	27(22.1)	173(14.4)
*χ*^*2*^ *= 111*.35***; *Phi = 0*.*305; Cramer’s V = 0*.*176*													
**Head shape**													
Plain(Ebab-eras)	34(68.0)	99(59.6)	25(39.7)	80(50.3)	33(76.7)	66(50.4)	50(90.9)	55(35.9)	45(75)	61(41.8)	22(42.3)	53(43.4)	623(51.9)
Crest(Guteya)	16(32.0)	67(40.4)	38(60.3)	79(49.7)	10(23.3)	65(49.6)	5(9.1)	98(64.1)	15(25)	85(58.2)	24(46.2)	64(52.5)	566(47.2)
Others	0(0.0)	0(0.0)	0(0.0)	0(0.0)	0(0.0)	0(0.0)	0(0.0)	0(0.0)	0(0.0)	0(0.0)	6(11.5)	5(4.1)	11(0.9)
*χ*^*2*^ *= 79*.49***; *Phi = 0*.*257; Cramer’s V = 0*.*182*													
**Feather morphology**													
Normal	39(78.0)	166(100)	39(61.9)	148(93.1)	10(23.3)	127(97.0)	11(20.0)	153(100)	55(91.7)	146(100)	38(73.1)	104(85.2)	1036(86.3)
Frizzle	0(0.0)	0(0.0)	0(0.0)	6(3.8)	0(0.0)	0(0.00)	0(0.0)	0(0.0)	0(0.0)	0(0.0)	0(0.0)	0(0.0)	6(0.5)
Silky	11(22.0)	0(0.0)	24(38.1)	0(0.0)	33(76.7)	4(3.0)	44(80.0)	0(0.0)	5(8.3)	0(0.0)	14(26.9)	18(14.8)	158(13.2)
*χ*^*2*^ *= 85*.46***; *Phi = 0*.*267; Cramer’s V = 0*.*189*													
**Feather distribution**													
Normal	50(100)	166(100)	63(100)	159(100)	43(100)	131(100)	55(100)	153(100)	55(91.7)	141(96.6)	47(90.4)	112(91.8)	1175(97.9)
Naked neck	0(0.0)	0(0.0)	0(0.0)	0(0.0)	0(0.0)	0(0.0)	0(0.0)	0(0.0)	5(8.3)	5(3.4)	5(9.6)	10(8.2)	25(2.1)
*χ*^*2*^ *= 61*.65***; *Phi = 0*.*227; Cramer’s V = 0*.*227*													

N = Number of chickens; M = Male; F = Female;

*p<0.05;

**p<0.01;

***p<0.001

**Fig 2:**
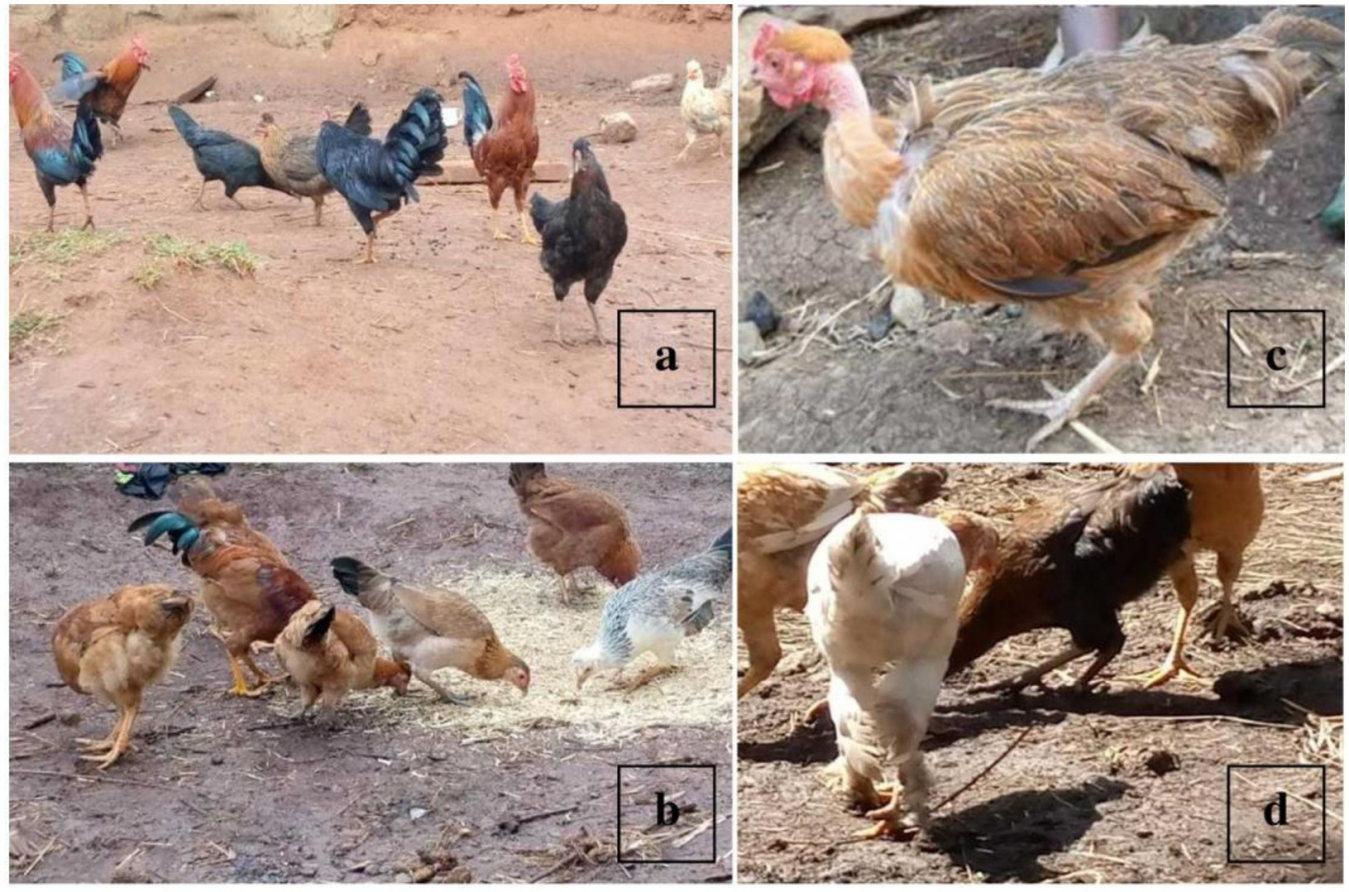
Indigenous chickens with different plumage colour in Aneded (a) and Dembecha (b), naked-neck chicken in Jawi (c), and white chicken with a shank feather in Banja (d).

Almost all (99.6%) of the sample chickens had wattles, and only 1.9% of them possessed shank feathers (Fig 2). White skin colour was predominant (88.4%) in the sample chicken population. Uncommon skin colour types of black and blue-black (1.8%) were also observed. Among female and male sample chickens, the majority (37.8%) possessed the rose comb type, followed by single and pea comb types with 29.8% and 17.9%, respectively. The plain head shape was most frequently observed, accounting for 51.9% of the sampled population, whereas 47.2% of chickens had a crest head shape. The majority (86.3%) of chickens had normal feather morphology. However, there were still very few chickens (0.5%) with frizzle feather morphology. All chickens had normal feather distribution except in lowland areas (Jawi and North Achefer), which possessed a small number of naked neck chickens (Fig 2).

**Fig 3:**
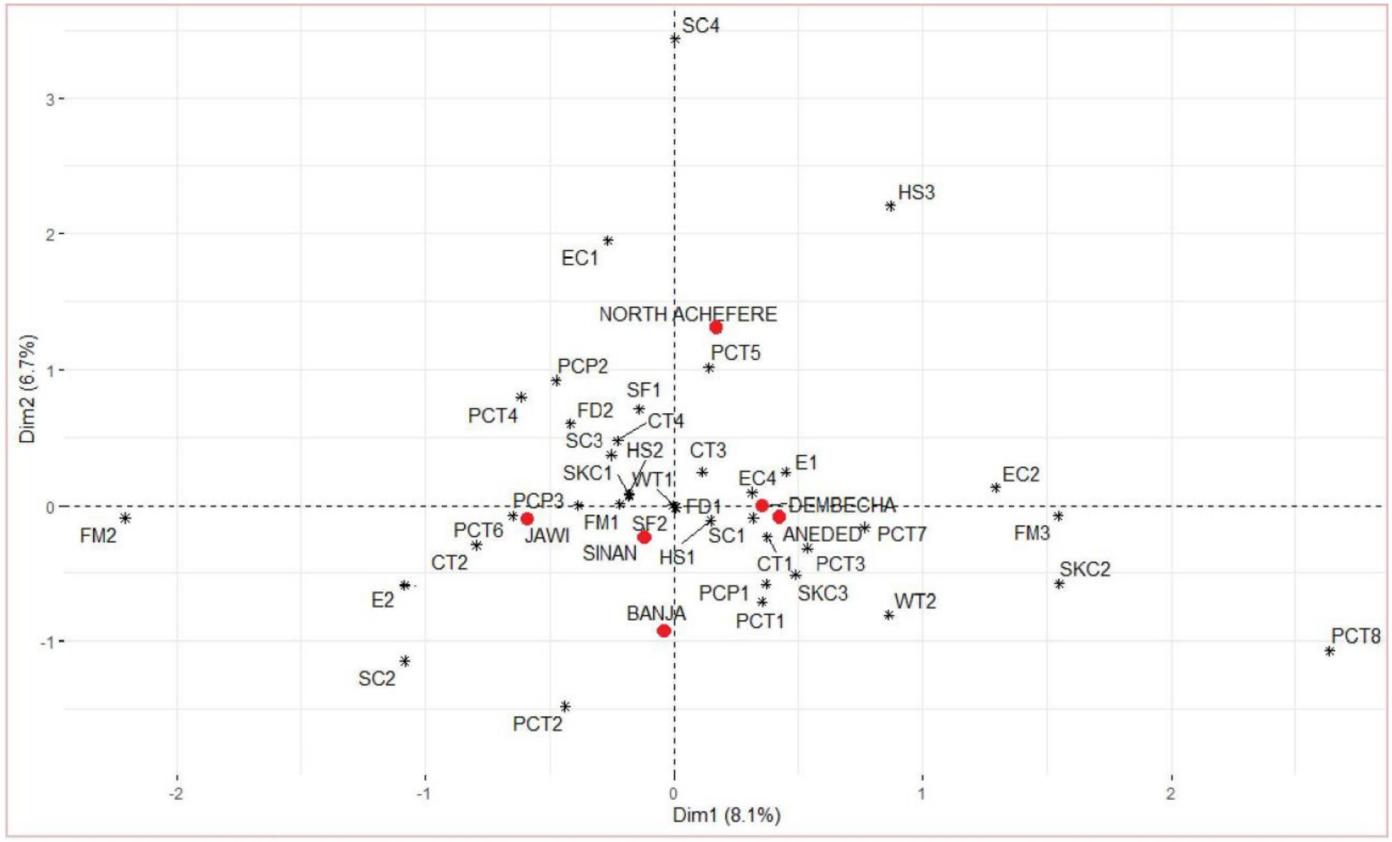
Bi-dimensional plot showing the associations among the categories of the different morphological variables. Plumage colour pattern: PCP1 = plain, PCP2 = patchy, PCP3 = spotted; Plumage colour type: PCT1 = completely white, PCT2 = completely black, PCT3 = completely red, PCT4 = *Gebisma*, PCT5 = multicolour, PCT6 = *teterima*, PCT7 = white with red, PCT8 = *sora*; Shank colour: SC1 = yellow, SC2 = black, SC3 = white, SC4 = others (blue, green…); Earlobe colour: EC1 = White, EC2 = red, EC4 = white and red; Skin colour: SKC1 = white, SKC2 = yellow, SKC3 = others; Comb type: CT1 = rose, CT2 = pea, CT3 = single, CT4 = others; Head shape: HS1 = plain, HS2 = crest, HS3 = others; Earlobe: E1 = present, E2 = absent; Wattle: WT1 = present, WT2 = absent; Shank feather: SF1 = present, SF2 = absent; Feather morphology: FM1 = normal, FM2 = frizzle, FM3 = silky; Feather distribution: FD1 = normal, FD2 = naked neck.

Multiple correspondence analyses were executed on twelve qualitative traits, and a bi-dimensional plot representing the associations among the various categories of qualitative traits is presented in Fig 3. About 14.8% of the total variation was explained by the first two dimensions, 8.1% by the first and 6.7% by the second. The indigenous chickens in North Achefer were clustered together with multicolour plumage colour types and head shapes other than plain and crest. Some of these chickens were also clustered by shank colour which is not common, such as blue and green, and by naked neck feather distribution. Indigenous chickens in Jawi were closely associated with pea comb type; some of the chickens had no earlobe and *teterima* (black spot on white, black with white tips, and white with black or red spots) plumage colour type. In the Dembecha site, the chickens were closely associated with white with red plumage colour type, silky feather morphology, and yellow skin colour. Yellow skin colour and silky feather morphology were also closely associated with the chickens found at the Aneded site. Indigenous chickens in Sinan were clustered together with normal feather distribution and an absence of shank feathers. In Banja, the indigenous chickens were closely associated with black plumage.

### Body weight and linear body measurements

The least squares means and standard errors for the effects of agro-ecology, sex, and location on body weight and other body measurements are presented in Table 3. In all study areas, sex had a significant (p<0.001) effect on body weight and all linear body measurements. Male chickens had consistently higher values than females. All body measurements were significantly affected by agro-ecology and location. However, the significant variation was also observed among chickens found in similar agro-ecologies. Indigenous chickens found in the lowland areas (Jawi and North Achefer) showed higher values than others for the majority of body measurements. The North Achefer chickens had significantly (p<0.001) higher average shank length, chest circumference, thigh circumference, comb height, and body weight than others. Chickens from Jawi had a higher value in body length, beak length, and shank circumference than others. The interaction of sex group and location was significant (p<0.001) for body weight and all other body measurements (Table 3). Indigenous female chickens from Banja had significantly (p<0.001) higher measurements in most of the variables. Whereas, the North Achefer male chickens were showing significantly (p<0.001) higher values in wing span, shank length, neck length, chest circumference, thigh circumference, body weight, and comb height.

**Table 3:** Least squares mean (± SE) body weight (kg) and other linear body measurements (cm) by agro-ecology, sex and location of indigenous chicken ecotypes in north-western Ethiopia.

Effect and levels	WS	SL	BL	BKL	NL	CL	SC	CC	TC	CH	BW
	LSM±SE	LSM±SE	LSM±SE	LSM±SE	LSM±SE	LSM±SE	LSM±SE	LSM±SE	LSM±SE	LSM±SE	LSM±SE
**Overall**	42.9±0.12	7.38±0.03	35.9±0.10	2.44±0.01	10.7±0.05	3.32±0.05	3.2±0.02	24.9±0.06	9.91±0.05	1.27±0.03	1.36±0.01
CV %	6.38	9.49	7.09	11.04	11.41	30.4	17.2	7.33	11.80	19.90	16.50
**Sex**	***	***	***	***	***	***	***	***	***	***	***
Male	47.47±0.16^a^	8.47±0.04^a^	39.3±0.14^a^	2.6±0.01^a^	11.8±0.07^a^	5.71±0.06^a^	3.66±0.03^a^	26.17±0.10^a^	11.4±0.07^a^	2.36±0.05^a^	1.59±0.01^a^
Female	41.23±0.09^b^	6.99±0.02^b^	34.6±0.08^b^	2.38±0.01^b^	10.2±0.04^b^	2.4±0.04^b^	3.0±0.02^b^	24.4±0.06^b^	9.36±0.04^b^	0.85±0.03^b^	1.27±0.01^b^
**Agro-ecology**	***	***	***	***	***	***	**	***	***	***	***
Highland	43.70±0.15^c^	7.41±0.03^b^	36.98±0.14^b^	2.49±0.02^ab^	11.3±0.07^a^	3.99±0.05^b^	3.26±0.03^b^	24.6±0.10^c^	9.96±0.06^b^	1.56±0.04^b^	1.39±0.01^b^
Midland	44.36±0.16^b^	7.34±0.03^b^	36.18±0.15^c^	2.46±0.02^b^	11.1±0.07^a^	4.06±0.06^ab^	3.43±0.04^a^	25.3±0.11^b^	10.6±0.07^a^	1.43±0.04^c^	1.37±0.01^b^
Lowland	44.82±0.16^a^	8.40±0.03^a^	37.56±0.15^a^	2.51±0.02^a^	10.6±0.07^b^	4.08±0.06^a^	3.31±0.04^ab^	25.9±0.10^a^	10.5±0.07^a^	1.70±0.04^a^	1.50±0.01^a^
**Location**	***	***	***	***	***	**	***	***	**	***	***
Banja	44.8±0.23^b^	7.59±0.06^b^	37.4±0.21^a^	2.52±0.02^b^	11.5±0.09^a^	4.15±0.08^ab^	3.37±0.04^b^	25±0.15^bc^	10.3±0.10^b^	1.6±0.07^b^	1.41±0.01^b^
Sinan	42.1±0.21^d^	7.14±0.05^c^	35.7±0.20^cd^	2.39±0.02^c^	10.8±0.09^b^	3.70±0.08^bc^	3.12±0.04^c^	23.8±0.13^d^	9.6±0.09^c^	1.65±0.07^ab^	1.33±0.01^c^
Dembecha	45.2±0.25^ab^	7.38±0.06^c^	37.2±0.23^bc^	2.51±0.02^c^	11.1±0.10^b^	3.38±0.09^c^	3.44±0.05^b^	25.5±0.16^bc^	10.4±0.10^ab^	1.1±0.08^c^	1.48±0.02^ab^
Aneded	43.2±0.22^c^	7.25±0.05^c^	35.5±0.20^d^	2.45±0.02^c^	11±0.09^b^	4.54±0.08^a^	3.38±0.04^b^	24.9±0.14^c^	10.6±0.10^a^	1.6±0.07^bc^	1.28±0.01^c^
Jawi	46.1±0.22^a^	8.48±0.05^a^	38.6±0.20^a^	2.66±0.02^a^	10.9±0.09^b^	4.38±0.08^a^	3.97±0.04^a^	26±0.14^ab^	10.6±0.10^a^	1.7±0.07^ab^	1.54±0.01^a^
N/Achefer	44.7±0.23^c^	8.53±0.06^a^	37.4±0.21^b^	2.35±0.02^d^	10.5±0.10^c^	4.21±0.09^ab^	2.72±0.05^d^	26.5±0.15^a^	10.8±0.10^ab^	1.95±0.08^a^	1.55±0.01^ab^
**Location** * **sex**	***	***	***	**	***	***	***	***	***	***	***
Banja, M	47.19±0.39	8.17±0.10	38.38±0.37	2.54±0.04	11.97±0.17	5.77±0.15	3.65±0.08	24.91±0.26	11.18±0.17	2.28±0.13	1.46±0.03
Banja, F	42.41±0.22	7.0±0.05	36.40±0.21	2.51±0.02	11.08±0.09	2.54±0.08	3.09±0.04	24.9±0.14	9.37±0.09	0.91±0.07	1.36±0.02
Sinan, M	44.68±0.35	7.76±0.09	37.26±0.33	2.48±0.03	11.49±0.15	4.96±0.13	3.34±0.07	24.46±0.23	10.44±0.15	2.42±0.11	1.47±0.03
Sinan, F	39.58±0.22	6.52±0.06	34.08±0.20	2.31±0.02	10.25±0.09	2.45±0.08	2.90±0.04	23.1±0.14	8.77±0.09	0.89±0.07	1.18±0.02
Dembecha, M	47.60±0.43	7.99±0.10	40.23±0.39	2.70±0.04	11.76±0.18	4.23±0.16	3.69±0.08	26.34±0.28	11.17±0.18	1.38±0.14	1.67±0.03
Dembecha, F	42.75±0.25	6.76±0.06	34.14±0.23	2.32±0.02	10.42±0.10	2.53±0.09	3.18±0.05	24.65±0.16	9.7±0.10	0.83±0.08	1.30±0.03
Aneded, M	45.59±0.38	7.90±0.09	37.16±0.35	2.56±0.04	11.67±0.16	6.59±0.14	3.61±0.07	25.32±0.24	11.42±0.16	2.41±0.12	1.36±0.03
Aneded, F	40.88±0.23	6.6±0.05	33.87±0.21	2.34±0.02	10.37±0.09	2.48±0.08	3.15±0.04	24.62±0.15	9.74±0.09	0.78±0.07	1.19±0.01
Jawi, M	49.54±0.36	9.41±0.09	41.57±0.33	2.72±0.03	11.22±0.15	6.38±0.13	4.51±0.07	27.49±0.23	11.71±0.16	2.63±0.12	1.77±0.03
Jawi, F	42.66±0.23	7.55±0.06	35.69±0.21	2.61±0.02	10.58±0.10	2.37±0.08	3.43±0.04	24.56±0.15	9.56±0.10	0.82±0.07	1.32±0.02
N/Achefer, M	50.22±0.39	9.56±0.10	41.27±0.36	2.57±0.04	12.71±0.17	6.36±0.15	3.17±0.08	28.49±0.25	12.66±0.17	3.06±0.12	1.84±0.03
N/Achefer, F	39.13±0.25	7.51±0.06	33.59±0.23	2.14±0.02	8.34±0.11	2.06±0.09	2.26±0.05	24.42±0.16	9.0±0.11	0.86±0.08	1.27±0.02

LSM = Least Square Mean; SE = Standard error; M = Male; F = Female; WS = Wing Span; SL = Shank Length; BL = Body Length; BKL = Beak Length; NL = Neck Length; CL = Comb Length; SC = Shank Circumference; CC = Chest Circumference; TC = Thigh Circumference; CH = Comb Height; BW = Body Weight;

*p<0.05;

**p<0.01;

***p<0.001

### Step-wise discriminant analysis

The stepwise discriminant analysis procedure revealed that, in both sexes, all the variables had a significant discriminating power between the sample populations. For both the female and male sample populations, shank traits (shank circumference and shank length) had the highest discriminating power, with a standard deviation of 0.59 and 1.07 for the female and male populations, respectively (Table 4).

**Table 4:** Traits used in discriminating the chicken population from different sites in stepwise discriminant analysis.

Sex	Variable	Total standard deviation	Partial R^2^	F-value	Pr>F	Wilks’ Lambda	Pr < Lambda	ASCC	Pr > ASCC
**Female**	SC	0.59	0.34	90.47	< .0001	0.66	< .0001	0.07	< .0001
	SL	0.79	0.29	70.27	< .0001	0.47	< .0001	0.12	< .0001
	NL	1.46	0.28	67.73	< .0001	0.34	< .0001	0.16	< .0001
	BKL	0.30	0.13	26.42	< .0001	0.29	< .0001	0.18	< .0001
	CC	1.85	0.12	23.39	< .0001	0.26	< .0001	0.21	< .0001
	WS	3.17	0.11	20.35	< .0001	0.23	< .0001	0.22	< .0001
	BL	2.69	0.09	17.46	< .0001	0.21	< .0001	0.24	< .0001
	TC	1.18	0.10	19.90	< .0001	0.19	< .0001	0.25	< .0001
	BW	0.23	0.08	15.36	< .0001	0.17	< .0001	0.27	< .0001
	CL	0.74	0.05	9.59	< .0001	0.16	< .0001	0.27	< .0001
**Male**	SL	1.07	0.48	58.90	< .0001	0.52	< .0001	0.10	< .0001
	SC	0.86	0.27	23.71	< .0001	0.38	< .0001	0.15	< .0001
	CL	1.85	0.18	14.27	< .0001	0.31	< .0001	0.19	< .0001
	BW	0.32	0.20	15.72	< .0001	0.25	< .0001	0.22	< .0001
	CC	2.49	0.19	14.31	< .0001	0.20	< .0001	0.25	< .0001
	WS	3.44	0.15	10.95	< .0001	0.17	< .0001	0.27	< .0001
	NL	1.31	0.14	9.89	< .0001	0.15	< .0001	0.30	< .0001
	CH	1.52	0.09	6.41	< .0001	0.13	< .0001	0.31	< .0001
	TC	1.58	0.08	5.29	.0001	0.12	< .0001	0.32	< .0001
	BL	3.42	0.08	5.11	.0002	0.11	< .0001	0.33	< .0001
	BKL	0.33	0.08	5.19	.0001	0.10	< .0001	0.34	< .0001

*p<0.05; **p<0.01; ***p<0.001; ASCC = Average Squared Canonical Correlation

### Discriminant analysis

The validity of the discriminant analysis procedure was assessed by means of reclassification statistics (Table 5). The overall classification rates (hit rate) of the female and male sample populations were 57.47% and 69.97%, respectively (Table 5). The overall hit rate in the female population was not high, as the hit rates observed for Banja (48.80%) and Dembecha (34.35%) were small. In the case of male chickens, the lowest classification rate was obtained for Banja with 42.00%, followed by Dembecha with about 65.00% (Table 5).

**Table 5:** Number of observations and percent (bracket) correct classified for female and male sample population using discriminant analysis.

Sex	Site	Banja	Sinan	Dembecha	Aneded	Jawi	N/Achefer	Total
**Female**	**Banja**	81(48.80)	27(16.27)	23(13.86)	13(7.83)	22(13.25)	0(0.00)	166(100)
	**Sinan**	23(14.47)	99(62.26)	4(2.52)	24(15.09)	3(1.89)	6(3.77)	159(100)
	**Dembecha**	25(19.08)	27(20.61)	45(34.35)	25(19.08)	9(6.87)	0(0.00)	131(100)
	**Aneded**	13(8.50)	27(17.65)	18(11.76)	89(58.17)	6(3.92)	0(0.00)	153(100)
	**Jawi**	19(13.01)	16(10.96)	6(4.11)	11(7.53)	92(63.01)	2(1.37)	146(100)
	**N/Achefer**	1(0.82)	8(6.56)	5(4.10)	1(0.82)	9(7.38)	98(80.33)	122(100)
**Male**	**Banja**	21(42.00)	10(20.00)	2(4.00)	10(20.00)	5(10.00)	2(4.00)	50(100)
	**Sinan**	13(20.63)	44(69.84)	3(4.76)	3(4.76)	0(0.00)	0(0.00)	63(100)
	**Dembecha**	0(0.00)	9(20.93)	28(65.12)	0(0.00)	4(9.30)	2(4.65)	43(100)
	**Aneded**	6(10.91)	0(0.00)	0(0.00)	47(85.45)	0(0.00)	2(3.64)	55(100)
	**Jawi**	0(0.00)	0(0.00)	10(16.67)	0(0.00)	50(83.33)	0(0.00)	60(100)
	**N/Achefer**	0(0.00)	1(1.92)	0(0.00)	0(0.00)	15(28.85)	36(69.23)	52(100)

### Non-parametric discriminant analysis

All the categorical variables that were used for the chi-square test were included in the nonparametric analysis to see how the sample populations differed as group entities. Both male and female populations were merged and analyzed together, and the result is presented in Table 6. The overall classification rate estimate was 55.42%.

**Table 6:** Number of observations and percent-classified (in brackets) into the site using a non-parametric discriminant for both male and female sample chicken populations.

**From site**	**Banja**	**Sinan**	**Dembecha**	**Aneded**	**Jawi**	**North Achefer**
**Banja**	81(37.50)	44(20.37)	35(16.20)	25(11.57)	27(12.50)	4(1.85)
**Sinan**	21(9.46)	139(62.61)	13(5.86)	39(17.57)	9(4.05)	1(0.45)
**Dembecha**	25(14.37)	40(22.99)	52(29.89)	36(20.69)	18(10.34)	3(1.72)
**Aneded**	9(4.33)	28(13.46)	23(11.06)	142(68.27)	4(1.92)	2(0.96)
**Jawi**	25(12.14)	15(7.28)	12(5.83)	17(8.25)	132(64.08)	5(2.43)
**North Achefer**	8(4.60)	9(5.17)	5(2.87)	4(2.30)	29(16.67)	119(68.39)

### Canonical discriminant analysis

The squared Mahalanobis distances between sites (Table 7) for female sample populations were significant (p<0.001). The shortest distance (4.73) was measured between Aneded and Dembecha, and the longest distance (19.25) was measured between Banja and North Achefer. In male sample populations, the shortest distance was between Banja and Aneded, with a value of 5.87 standard units, and the longest was between Dembecha and North Achefer, with a value of 16.8 (Table 7). The multivariate statistics for differences between the districts were also significant (p<0.001) in all four multivariate tests (Wilks’ Lambda, Pillai’s Trace, Hotelling-Lawley Trace, and Roy’s Greatest Root). Similarly, the univariate statistic testing the hypothesis that class means are equal showed that the means of each variable are significantly different (p<0.001) between sites. The values of Wilks’ lambda were 0.16 and 0.10 for the female and male sample populations, respectively.

**Table 7:** The squared Mahalanobis distance between sites for the female (below the diagonal) and male (above the diagonal) sample chickens.

**From site**	**Banja**	**Sinan**	**Dembecha**	**Aneded**	**Jawi**	**North Achefer**
**Banja**	*	7.35	8.27	5.87	8.79	12.48
**Sinan**	5.33	*	7.41	9.38	12.09	15.32
**Dembecha**	5.80	5.96	*	12.55	10.17	16.80
**Aneded**	6.52	5.97	4.73	*	11.99	15.09
**Jawi**	5.70	7.55	7.84	8.06	*	12.10
**North Achefer**	19.25	16.56	18.77	18.00	18.22	*

The procedure of canonical discriminant analysis extracted five canonical variates for the female chicken. For female sample populations, the ratio of between-group variability to within-group variability detected by canonical variate 1 (CAN1) was much larger than the other four canonical variates. The five canonical variates had different discriminatory powers, with a larger proportion (82%) of the total variance explained by the first two canonical variates (Table 8). Unlike the female sample population, the Eigen values for the male populations were larger for CAN1, CAN2, and CAN3, indicating their better discriminating capacities (Table 8). These three variates explained 90% of the total variance in the population.

**Table 8:** Summary of canonical correlations in female and male chickens.

Sex	Functions	Canonical correlation	Eigen values				Likelihood ratio	Approximate F value	Pr>F
			Eigen value	Difference	Proportion	Cumulative			
**Female**	**CAN1**	0.79	1.65	1.15	0.63	0.63	0.16	38.35	< .0001
	**CAN2**	0.58	0.50	0.21	0.19	0.82	0.43	22.50	< .0001
	**CAN3**	0.47	0.28	0.16	0.11	0.93	0.65	16.81	< .0001
	**CAN4**	0.34	0.13	0.06	0.05	0.98	0.83	11.82	< .0001
	**CAN5**	0.25	0.07		0.02	1.00	0.94	9.43	< .0001
**Male**	**CAN1**	0.76	1.34	0.51	0.42	0.42	0.10	16.30	< .0001
	**CAN2**	0.67	0.82	0.16	0.26	0.68	0.24	13.18	< .0001
	**CAN3**	0.63	0.67	0.44	0.21	0.90	0.44	10.70	< .0001
	**CAN4**	0.43	0.23	0.14	0.07	0.97	0.74	6.25	< .0001
	**CAN5**	0.29	0.10		0.03	1.00	0.91	4.23	0.00

The first two canonical variates (CAN 1 and CAN 2) distinctly separated the female sample populations from North Achefer and Jawi, which were found in lowland areas (Table 9). In male chickens, the first three canonical variates (CAN1, CAN2, and CAN3) distinctly separated the sample populations of the North Achefer, Sinan, and Jawi (Table 9).

**Table 9:** Class means on canonical variables of female and male chickens.

Sex	District/Site	CAN1	CAN2	CAN3	CAN4	CAN5
**Female**	**Banja**	0.75	0.48	-0.39	0.57	0.15
	**Sinan**	0.30	-0.23	-0.88	-0.43	-0.09
	**Dembecha**	0.59	-0.65	0.41	0.24	-0.48
	**Aneded**	0.49	-0.88	0.46	-0.14	0.37
	**Jawi**	0.41	1.21	0.54	-0.34	-0.04
	**North Achefer**	-3.16	0.01	0.02	0.10	0.01
**Male**	**Banja**	-0.41	-0.39	-0.36	-0.14	-0.67
	**Sinan**	-1.12	-0.01	1.01	0.59	0.06
	**Dembecha**	-0.77	1.16	0.47	-0.93	0.14
	**Aneded**	-0.64	-1.28	-0.89	-0.15	0.33
	**Jawi**	0.74	1.19	-0.96	0.41	0.06
	**North Achefer**	2.22	-0.59	0.78	-0.13	0.03

### Cluster analysis

**Fig 4:**
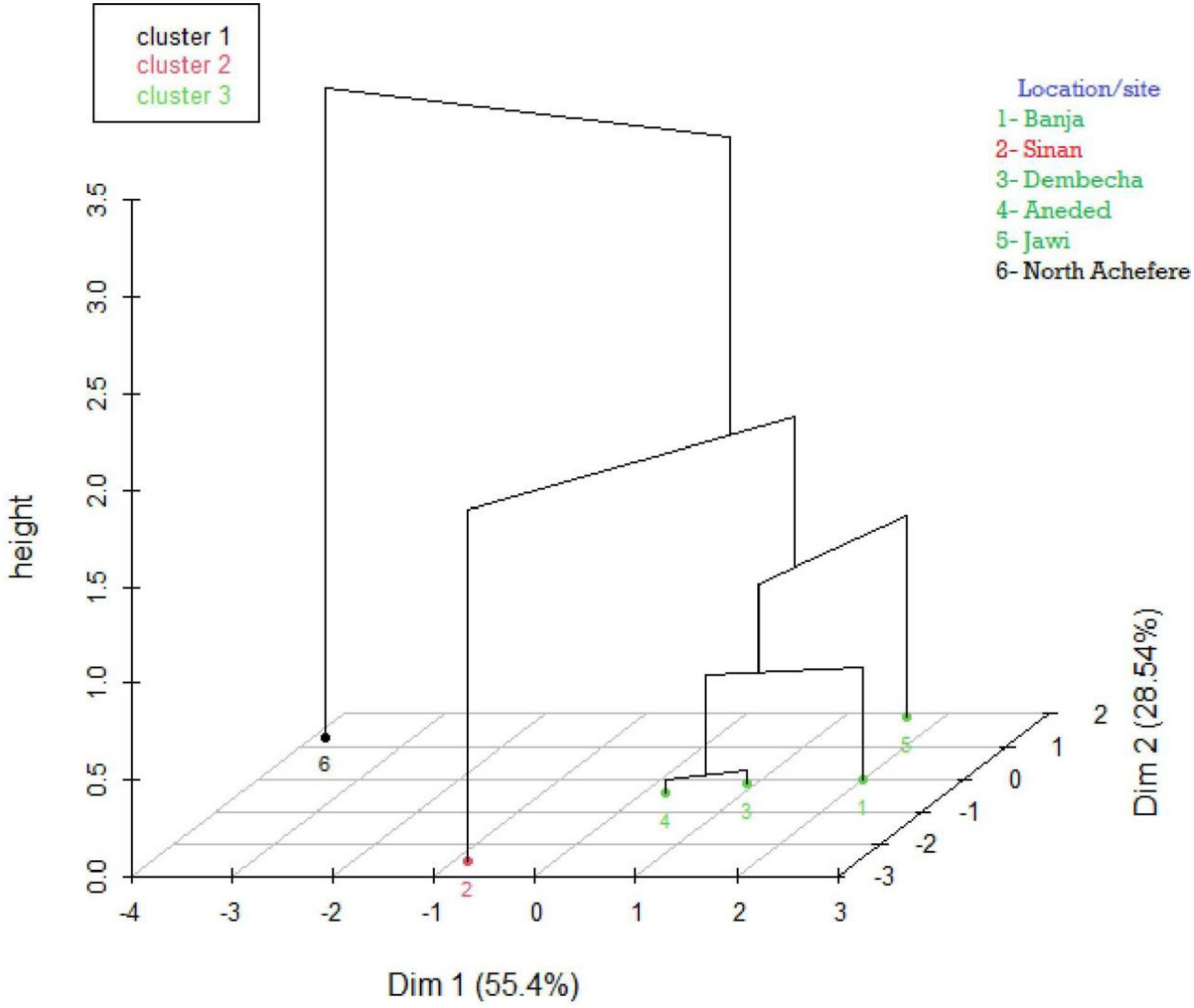
Hierarchical clustering on the factor map (3D map) for female chickens by using quantitative traits having a high discriminating power in classification of chickens.

**Fig 5:**
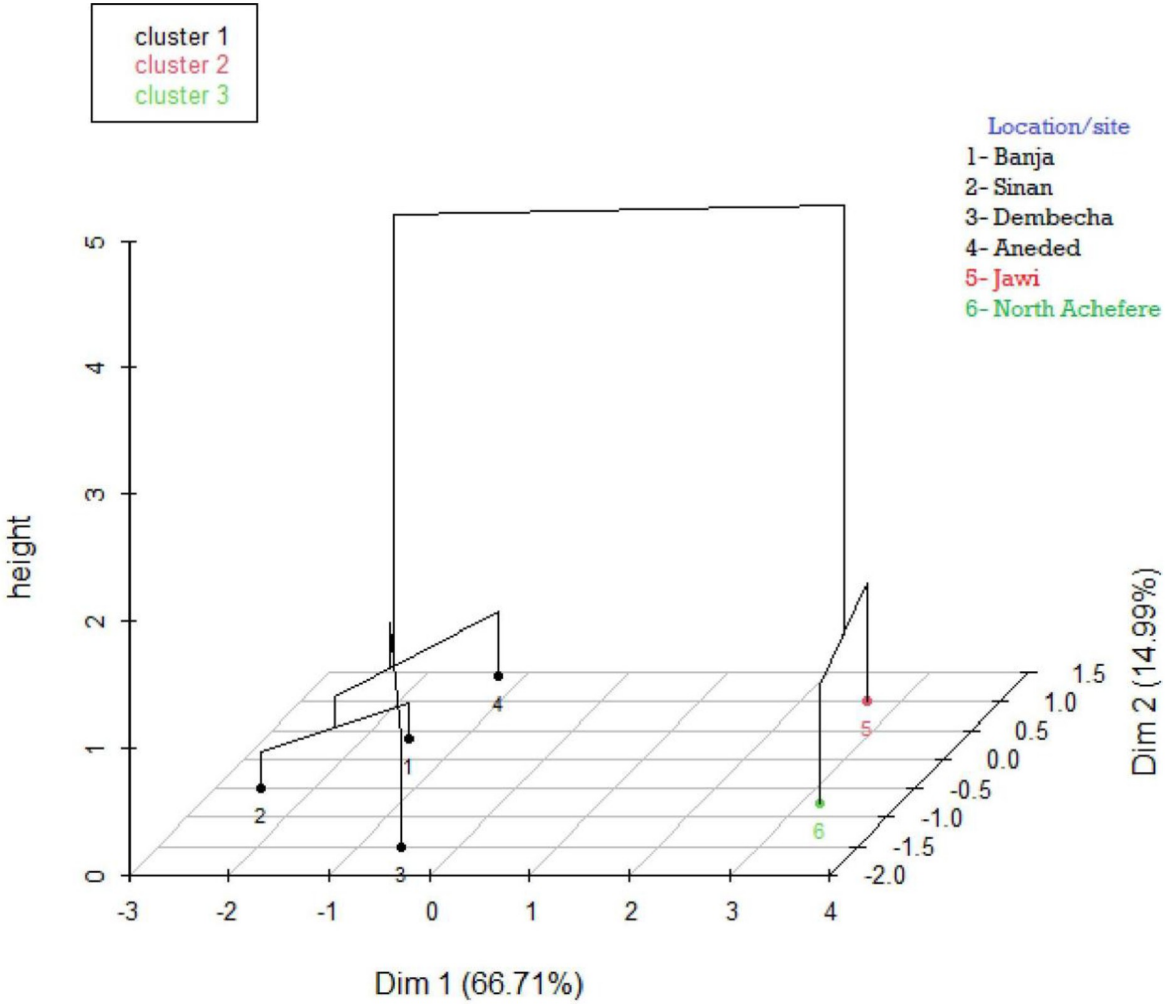
Hierarchical clustering on the factor map (3D map) for male chickens by using quantitative traits having a high discriminating power in classification of chickens.

**Fig 6:**
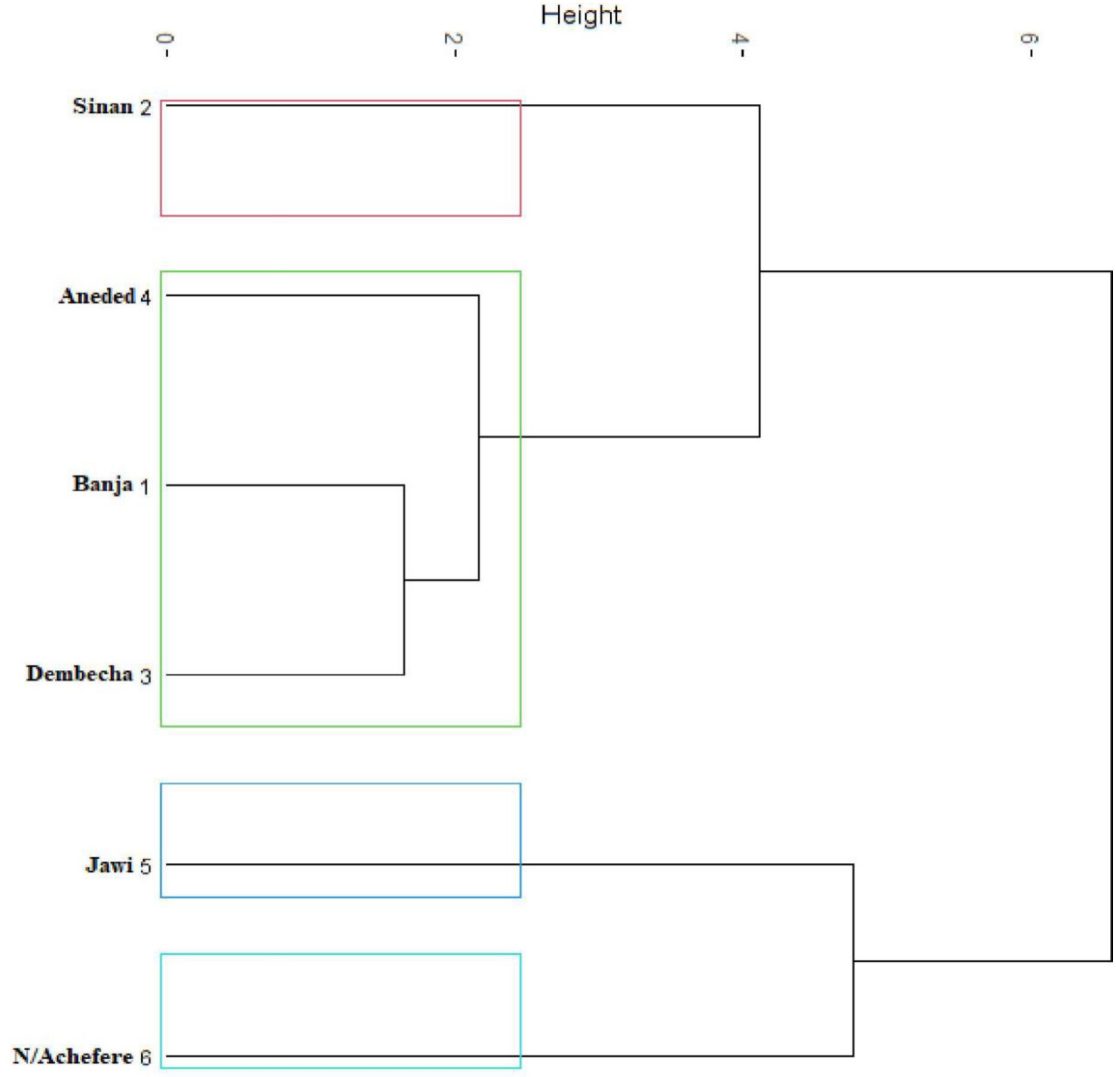
Dendrogram constructed based on quantitative traits having a high discriminating power in classification of chickens for two sexes.

The hierarchical clustering map for female sample chickens (Fig 4) was established using principal components and showed three clusters. About 84% of the variation was explained by dimension 1 (55.4%) and dimension 2 (28.54%). In males, about 82% of the total variation was explained by the first two dimensions (Fig 5). While female and male chickens were analyzed together, four distinct clusters were formed (Fig 6). Chickens from Banja, Dembecha, and Aneded were clustered together, and chickens from North Achefer, Sinan, and Jawi were clustered independently.

## Discussions

### Qualitative morphological traits

The red plumage colour was the most frequently observed in almost all study areas. Similar results were reported in southern Ethiopia and southeastern Oromia, Ethiopia [17, 18]. Similar results in indigenous chickens are also found in Abobo and Gambella *Ketema Zuria* districts of the Gambella region [13]. In line with the present study, red was reported as the most dominant plumage colour in those chicken populations from the southern part of Ethiopia, Oromia, and Benshangul-Gumuz regions [18]. In contrast to the present finding, 25.49% of indigenous chickens in the northwest parts of Ethiopia had white plumage [10]. The dominancy of white plumage colour across all chicken populations in Burkina Faso has also been reported [19]. The predominant occurrence of indigenous chickens with red and *Gebisma* plumage colour in the studied area is possibly attributed to farmers’ preferences. In some study areas, farmers’ do not prefer white colour due to its capability of attracting people, and they believe the eyes of human beings may negatively affect the growth and egg production of the chicken. In addition, some farmers prefer chicken with colour that is not bright to protect it from predators [11].

The majority of chickens in this study had yellow shank colour. The result of the current study is in line with the findings in Northwest Ethiopia [10], Gambella regions of Ethiopia [13], Southern Ethiopia [17], Rwanda [20], Nigeria [21], and Southeastern Oromia region [22], in which the majority of indigenous chickens had a yellow shank colour. On the contrary, the frequent occurrence of grey and green shank colours in some areas of Southeastern Oromia was reported [22]. Shank colour indicates the chicken’s foraging efficiency, nutritional and immune status, and sexual desirability [23]. In the current study, the presence of yellow shank colour was higher in males than females in all the districts. This might indicate the foraging efficiency of males, which enables them to get high carotenes. Similarly, a report of [22] suggested that cocks might have good access to feed resources than hens as they can compete better. The mixture of white and red earlobe colour was predominant in the indigenous chickens. In contrast to the current study, the majority of indigenous chickens from southern Ethiopia [17] and southeastern Oromia [22] had red earlobes. On the other hand, the predominance of yellow earlobes was reported in the Gambella region of Ethiopia [13]. The difference in earlobe colour might be due to their adaptation to the local environment and differences in ancestral lineages [10]. The nutritional status of birds could also be a possible cause for this variation [22]. The rare incidence of shank feather in this study is in line with the report in Northwest Ethiopia [10], and Gambella region [13]. The predominant white skin colour in this study is different from the reports from different regions of Ethiopia [11] and Southeastern Oromia [22], which indicated the frequent occurrence of yellow skin colour. A high proportion of yellow skin colour was found relatively in highland areas (Banja and Sinan). Similar results were obtained in different regions of Ethiopia [11], in which the populations in the high altitudes (Sheka, Farta and Horro) were comprised a huge proportions of chickens with yellow skin relative to the others. Besides the agro-ecological region, the proportion of males with yellow skin colour was higher than that of females. This is probably because the scavenging feed resource base is relatively better in the high altitude regions, and cocks have a stronger foraging behavior than the hens [11].

The present study showed that the majority of the indigenous male chicken possessed rose comb, which is in line with the findings found in Southeastern Oromia [22]. In contrast to the current study, 55% of chickens were single combed in Southern Ethiopia [17]. Similarly, in Gambella region of Ethiopia [13], the frequent occurrence of single combed indigenous chickens reported. The majority of indigenous chickens found in Northwest Ethiopia and different regions of Ethiopia (Sheka, Mandura, Konso, and Farta) had pea combs [10, 11]. Single-comb male chickens were culturally not preferred by farmers in all study areas, and this might be the possible driving force behind the low frequency of single-comb chickens. In harmony with the current study, about 51% of indigenous chickens in Northwestern Ethiopia had a plain head shape. Similarly, the frequent occurrence of indigenous chickens with plain heads reported in the Gambella region [13]. On the contrary, most of the chickens in Farta were identified with crest heads [11]. On the other hand, the majority of indigenous chickens in Southeastern Oromia had snake head shape [22]. The current result agrees with the findings of [17] in which the highest proportion of chickens in southern Ethiopia had a normal feather distribution and morphology. Similar results were also reported in the Gambella region of Ethiopia [13]. The naked-neck gene is one of the major genes in indigenous chickens and is important for heat tolerance and adult fitness [24]. In line with the above statement, small proportions of naked neck chickens were found in lowland areas (Jawi and North Achefer). Unattractive aesthetic nature of these chickens by farmers’ might be the possible reason for low distribution of these chickens in the study area.

### Quantitative morphological traits

Sex had significant effect on body weight and all linear body measurements. Male chickens were showing higher value for all body measurements than females. In line with the current study, the effect of sex was significant for all traits being higher in males than female indigenous chickens found in Ethiopia [25]. Similarly, sex had a significant effect (p<0.05) on all linear body measurement and body weight of indigenous chickens of Ethiopia with the higher value of male chickens [26]. The superiority of males over females could be attributed to sexual dimorphism due to differences in the level of male sex hormones, which is responsible for larger muscle development in males than in females [27]. The average live weight values of 1.59 and 1.27 kilograms for males and females, respectively, in the current study are comparable with those reported by [25] in Ethiopian indigenous chickens. But, the values were higher than those reported in Northwest Ethiopia [10], Gambella region [13], Southeaster Oromia [22], and different agro-ecologies of Ethiopia [26]. The chest circumference reported by [26] and the wing span reported by [25] are in line with the current findings. Nevertheless, body length values of indigenous chickens of Ethiopia reported by [26] were lower, 26.07 cm and 23.52cm for male and female chickens, respectively. On the other study, the higher values of wing span (65.77 cm for males and 56.83 cm for females), shank length (10.19 cm and 8.36 cm), and neck length (18.47 cm and 16.92 cm) reported for the indigenous chickens of Gambella [13]. These variations in traits could be attributed to the genetic background of local chickens and the quality and quantity of the available scavengeble feed resources in different regions. Agro-ecology and location significantly affected all body measurements. Accordingly, indigenous chickens found in lowland areas (Jawi and North Achefer) had a higher value than others for the majority of their body measurements. However, the Banja indigenous chickens had the longest necks. Similarly, the Aneded chickens had the longest combs compared to the indigenous chickens found at the other sites. These significant differences observed between indigenous chickens from different locations could be due to management practices [28], and genetic make-up [29]. The body measurement differences of chickens found in similar agro-ecologies also might be due to the management system used by farmers and the genetic make-up of chickens.

All variables that were subjected to the stepwise discriminant analysis procedure had a significant discriminating power for both sexes. Shank circumference for females and shank length for males showed a high discriminating power. Comb length and beak length had the least discriminating power in female and male chickens, respectively. On the contrary, the body length has shown the highest level of significant discriminating power, while shank length had the least impact in differentiating the chicken populations of indigenous chickens found in the other part of Ethiopia [25]. In the other study, chest circumference and body weight showed a high discriminating power in indigenous male and female chickens, respectively, which were located in the Metekel zone of Northwestern Ethiopia [30]. Consistent with the current findings, [12] reported that shank length was the most important variable to discriminate among three chicken ecotypes reared in North Gondar, Ethiopia. The variation of variables in discriminating power for the classification of chickens in different studies might be due to the analysis method utilized and the unique importance of these variables in the classification of chickens in the specific study areas. The overall classification rates for the female and male sample populations were 57.47% and 69.97%, respectively. Comparable to the current study, the correct classification ranged from 41–84% in the case of female chickens found in West Hararghe zone [31]. In the correct classification percent table, the diagonal values represent the proportion of observations that were correctly classified as belonging to a particular group. Whereas the above and below diagonal values enable us to know the misclassified percentage of groups. The difference between the above and below diagonal values might be due to the size and distribution of the groups being classified or the difference in the sample size between groups.

The female sample populations from Banja, Dembecha, and Aneded and the male sample populations from Banja and Dembecha were more heterogeneous on the quantitative variables, as can be witnessed from their respective low hit ratios. It indicates the populations from these sites were morphologically more closely related to each other. For instance, the discriminant analysis correctly classified 48.8% of chickens in Banja into their respective origin populations, with 16.27%, 13.86%, 7.83%, 13.25%, and 0.00% being misclassified as Sinan, Dembecha, Aneded, Jawi, and North Achefer, respectively. Whereas, in male chickens, the analysis correctly classified 42% of chickens in Banja into their respective populations, with 20%, 4%, 20%, 10%, and 4% being misclassified as Sinan, Dembecha, Aneded, Jawi, and North Achefer, respectively. The higher classification rates were recorded on North Achefer and Jawi for female chickens. This indicates the homogeneous and distinctive nature of the chicken populations found in these areas.

In the current study, all the squared Mahalanobis distances were highly significant (*P* < 0.001). This revealed that the population from each site is distinct and has its own measurable differences from others. These observations are in good agreement with those obtained in different parts of Ethiopia [25] and Metekel zone [30]. The multivariate statistics for differences between the sites was also significant (p<0.001) in all of the four multivariate tests (Wilks’ Lambda, Pillai’s Trace, Hotelling-Lawley Trace and Roy’s Greatest Root. The value of Wilks’ lambda for the female sample populations was 0.16. This shows that most (84%) of the variability in the discriminator variables was due to differences between populations rather than variation within populations. In females, the shortest distance was measured between Aneded and Dembecha, and the longest distance was measured between Banja and North Achefer. Whereas, in male sample populations, the shortest distance was between Banja and Aneded and the longest was between Dembecha and North Achefer. The shortest distance observed between chickens might be attributed to the sharing of similar genetic identities as a result of non-selection, inbreeding, and migration among these chickens over many generations [32]. The squared Mahalanobis distances reported among the Ethiopian indigenous chickens [25] ranged from 4.39 to 23.9, which is comparable with the current findings (ranging from 4.73 to 19.25). On the other hand, lower squared Mahalanobis distances ranging from 1.19 to 4.8 and 2.19 to 7.95 for female and male chickens, respectively, were reported for indigenous chickens in West Hararghe zone [31]. Such variations might arise in the methods applied for computing the Mahalanobis distances and the number of samples used in the discriminant analysis, being higher in a smaller sample size than in a larger [25].

The canonical discriminant analysis extracted five canonical variables for female chickens, of which CAN1 and CAN2 accounted for 63% and 19% of the total variations, respectively. In males, three canonical variates, CAN1, CAN2, and CAN3, accounted for 42%, 26%, and 21%, respectively. These canonical variates in female and male chickens were distinctly separated among indigenous chickens found in North Achefer, Sinan, and Jawi. This observation is somehow comparable with that of indigenous chicken populations in Northwestern Ethiopia, which reported that CAN1 and CAN2 accounted for 66.75% and 33.3% of the total variation, respectively [12]. The higher CAN1 values of 73.2%, 91.46%, and 99.36%, reported for indigenous chickens in Ethiopia [26, 31]. Three clusters were found for each male and female chicken by utilizing quantitative traits that have high discriminating power in the classification of chickens. Some grouping variation was found in male and female chickens, and it might be due to sample size differences. On the other hand, four distinct clusters were found while the quantitative traits had high discriminating power in classifying male and female chickens together. By combining the above two clustering results, the chickens in the study area could be clustered into four; cluster one (North Achefer), cluster two (Sinan), cluster three (Jawi), and cluster four (Banja, Dembecha, and Aneded).

## Conclusion

The univariate and multivariate analysis of qualitative and quantitative traits revealed the existence of morphological differences between indigenous chickens in different districts. Generally, the results of the analyses enabled us to classify the indigenous chickens found in the study area into four ecotypes: ecotype 1 (Banja, Dembecha, and Aneded), ecotype 2 (North Achefer), ecotype 3 (Sinan), and ecotype 4 (Jawi). Molecular-based characterization studies should be conducted to strengthen the current findings.

## Supporting information

S1 File Raw data (qualitative traits) used in chicken morpho-biometric characterization. (XLSX)

S2 File Raw data (quantitative traits) used in chicken morpho-biometric characterization. (XLSX)

S3 File Syntax used for multiple correspondence analysis (MCA) in R software. (DOCX)

S4 File The SAS result of discriminant analysis for female chickens. (DOCX)

S5 File The SAS result of discriminant analysis for male chickens. (DOCX)

S6 File R syntax used in cluster analysis. (DOCX)

## References

[1] Wong J, de Bruyn J, Bagnol B, Grieve H, Li M, Pym R, et al. Small-scale poultry and food security in resource-poor settings: A review. Global Food Security. 2017;15:43–52. doi: 10.1016/j.gfs.2017.04.003

[2] CSA. Agricultural sample survey, report on livestock and livestock characteristics. 2021 Contract No.: 589.

[3] Weigend S, Romanov MN. Current strategies for the assessment and evaluation of genetic diversity in chicken resources. World’s Poultry Science Journal. 2001;57(3):275–88. doi: 10.1079/WPS20010020

[4] Dorji N, Sunar S. Short communication Morphometric variations among five Bhutanese indigenous chickens (Gallus domesticus). Journal of Animal and Poultry Sciences. 2014;3(3):76–85.

[5] Aklilu E, Kebede K, Dessie T, Banerjee A. Phenotypic characterization of indigenous chicken population in Ethiopia. International Journal of Interdisciplinary and Multidisciplinary Studies. 2013;1(1):24–32.

[6] Manyelo TG, Selaledi L, Hassan ZM, Mabelebele M. Local chicken breeds of Africa: their description, uses and conservation methods. Animals. 2020;10(12):2257. doi: 10.3390/ani10122257 33266253 PMC7760642

[7] Vallejo-Trujillo A, Kebede A, Lozano-Jaramillo M, Dessie T, Smith J, Hanotte O, et al. Ecological niche modelling for delineating livestock ecotypes and exploring environmental genomic adaptation: The example of Ethiopian village chicken. Frontiers in Ecology and Evolution. 2022;10.

[8] Lowry DB. Ecotypes and the controversy over stages in the formation of new species. Biological Journal of the Linnean Society. 2012;106(2):241–57.

[9] Tadelle D, Kijora C, Peters K. Indigenous chicken ecotypes in Ethiopia: growth and feed utilization potentials. International Journal of Poultry Science. 2003;2(2):144–52.

[10] Halima H, Neser FW, van Marle-Koster E, de Kock A. Phenotypic variation of native chicken populations in northwest Ethiopia. Trop Anim Health Prod. 2007;39(7):507–13. doi: 10.1007/s11250-007-9032-2 . 17969713

[11] Dana N, Dessie T, van der Waaij LH, van Arendonk JAM. Morphological features of indigenous chicken populations of Ethiopia. Animal Genetic Resources/Ressources génétiques animales/Recursos genéticos animales. 2010;46:11–23. doi: 10.1017/s2078633610000652

[12] Getu A, Alemayehu K, Wuletaw Z. Canonical Analysis for Assessment of Genetic Diversity of Three Indigenous Chicken Ecotypesin North Gondar Zone, Ethiopia. Iranian Journal of Applied Animal Science. 2014;4(4):871–6.

[13] Bekele G, Goshu G, Melesse A, Esatu W, Dessie T. On-Farm Phenotypic and Morphological Characterization of Indigenous Chicken Populations in Gambella Region, Ethiopia. International Journal of Poultry Science. 2021;20(1):27–38. doi: 10.3923/ijps.2021.27.38

[14] FAO. Phenotypic characterization of animal genetic resources. FAO Animal Production and Health Guidelines. 2012.

[15] SAS. SAS for Windows, ver. 9.4. 2012.

[16] Team RC. R: A language and environment for statistical computing. 2021.

[17] Melesse A, Negesse T. Phenotypic and morphological characterization of indigenous chicken populations in southern region of Ethiopia. Animal Genetic Resources/Ressources génétiques animales/Recursos genéticos animales. 2011;49:19–31. doi: 10.1017/s2078633611000099

[18] Dana N, Van der Waaij LH, Dessie T, Van Arendonk JA. Production objectives and trait preferences of village poultry producers of Ethiopia: implications for designing breeding schemes utilizing indigenous chicken genetic resources. Tropical animal health and production. 2010;42(7):1519–29. doi: 10.1007/s11250-010-9602-6 20512411 PMC2929342

[19] Zare Y, Gnanda I, Houaga I, Kere M, Traore B, Zongo M, et al. Morpho-Biometric Evaluation of the Genetic Diversity of Local Chicken Ecotypes in Four Regions (Centre-East, Sahel, Centre-North and South-West) of Burkina Faso. International Journal of Poultry Science. 2021;20(6):231–42.

[20] Habimana R, Ngeno K, Mahoro J, Ntawubizi M, Shumbusho F, Manzi M, et al. Morphobiometrical characteristics of indigenous chicken ecotype populations in Rwanda. Tropical Animal Health and Production. 2021;53(1):1–11. doi: 10.1007/s11250-020-02475-4 33219485

[21] Daikwo IS, Okpe AA, Ocheja JO. Phenotypic Characterization of Local Chickens in Dekina. International Journal of Poultry Science. 2011;10(6):444–7. doi: 10.3923/ijps.2011.444.447

[22] Negassa D, Melesse A, Banerjee S. Phenotypic characterization of indigenous chicken populations in Southeastern Oromia Regional State of Ethiopia. Animal Genetic Resources/Ressources génétiques animales/Recursos genéticos animales. 2014;55:101–13. doi: 10.1017/s2078633614000319

[23] Eriksson J, Larson G, Gunnarsson U, Bed’Hom B, Tixier-Boichard M, Strömstedt L, et al. Identification of the yellow skin gene reveals a hybrid origin of the domestic chicken. PLoS genetics. 2008;4(2):e1000010. doi: 10.1371/journal.pgen.1000010 18454198 PMC2265484

[24] Melesse A, Maak S, Von Lengerken G. The performance of naked neck and their F1 crosses with Lohmann White and New Hampshire chicken breeds under long-term heat stress conditions. Ethiopian J Anim Product. 2005;5:91–107.

[25] Melesse A, Tadele A, Assefa H, Taye K, Kebede T, Taye M, et al. Assessing the Morphological Diversity of Ethiopian Indigenous Chickens Using Multivariate Discriminant Analysis of Morphometric Traits for Sustainable Utilization and Conservation. Poultry Science Journal. 2021;9 (1):61–72. doi: 10.22069/psj.2021.18469.1644

[26] Bekele B, Melesse A, Esatu W, Dessie T. Statistical Modeling of Live Body Weight and Linear Body Measurements of Local Chicken at Different Agro-Ecologies of Ethiopia. International Journal of Poultry Science. 2021;20(4):146–51. doi: 10.3923/ijps.2021.146.151

[27] Rotimi E, Egahi J, Adeoye A. Phenotypic characterization of indigenous chicken population in Gwer-West, Benue State, Nigeria. World Scientific News. 2016;53(3):343–53.

[28] Moges F. Indigenous chicken production and marketing systems in Ethiopia: Characteristics and opportunities for market-oriented development: ILRI (aka ILCA and ILRAD); 2010.

[29] Apuno A, Mbap S, Ibrahim T. Characterization of local chickens (Gallus gallus domesticus) in shelleng and song local government areas of Adamawa State, Nigeria. Agriculture and Biology Journal of North America. 2011;2(1):6–14. doi: 10.5251/abjna.2011.2.1.6.14

[30] Getachew F, Abegaz S, Assefa A, Misganaw M, Emshaw Y, Hailu A, et al. Multivariate analyses of morphological traits in indigenous chicken populations of Metekel zone, Northwestern Ethiopia. Animal Genetic Resources/Ressources génétiques animales/Recursos genéticos animales. 2017;59:15–25. doi: 10.1017/s2078633616000084

[31] Wolde Kawole B, Mengesha YT, Zeleke NA. On farm phenotypic characterization of indigenous chicken ecotypes in west Hararghe zone, Oromia region, Ethiopia. 2019.

[32] Daikwo S, Dike U, Dim N. Discriminant analysis of morphometric differences in the normal feathered and frizzle feathered chickens of north central Nigeria. Agro-Science. 2015;14(3):12–5.

