# Supplementary material for: Morpho-biometric characterization of indigenous chicken ecotypes in north-western Ethiopia
Source: PLoS One. 2023 Jun 2;18(6):e0286299. doi: 10.1371/journal.pone.0286299 (PMC10237646; doi:10.1371/journal.pone.0286299)
Supplement: S3 File — (DOCX) [file pone.0286299.s003.docx]

setwd("E:/my data with R")

library(readr)

MCA <- read_csv("mca.csv")

View(MCA)

install.packages(c("FactoMineR", "factoextra"))

library("FactoMineR")

library("factoextra")

MCA(MCA, ncp = 6, graph = TRUE)

ca <- MCA(MCA, graph = FALSE)

print(ca)

library("factoextra")

eig.val <- get_eigenvalue(ca)

fviz_screeplot(ca, addlabels = TRUE, ylim = c(0, 45))

fviz_mca_biplot(ca,

repel = TRUE,

ggtheme = theme_minimal())

var <- get_mca_var(ca)

var

head(var$coord)

head(var$cos2)

head(var$contrib)

fviz_mca_var(ca, choice = "mca.cor",

repel = TRUE,

ggtheme = theme_minimal())

fviz_mca_var(ca,

repel = TRUE,

ggtheme = theme_minimal())

fviz_mca_var(ca, col.var="black", shape.var =8,

repel = TRUE)

gradient.cols = c("#00AFBB", "#E7B800", "#FC4E07")

head(var$cos2, 4)

fviz_mca_var(ca, col.var = "cos2",

gradient.cols = c("#00AFBB", "#E7B800", "#FC4E07"),

repel = TRUE,

ggtheme = theme_minimal())

fviz_mca_var(ca, alpha.var="cos2",

repel = TRUE,

ggtheme = theme_minimal())

fviz_mca_ind(ca, col.ind = "cos2",

gradient.cols = c("#00AFBB", "#E7B800", "#FC4E07"),

repel = TRUE,

ggtheme = theme_minimal())

fviz_mca_ind(ca,

label = "none",

habillage = "dist",

palette = c("#00AFBB", "#E7B800"),

addEllipses = TRUE, ellipse.type = "confidence",

ggtheme = theme_minimal())

fviz_mca_var(ca,

label = "none",

habillage = "dist", # color by groups

palette = c("#00AFBB", "#E7B800"),

addEllipses = TRUE, ellipse.type = "confidence",

ggtheme = theme_minimal())

####HCPC###

library(readr)

both_qual <- read_csv("E:/my data with R/DATA/both qual.csv")

View(both_qual)

res.mca <- MCA(Qual_female,

ncp = 1200, # Number of components kept

quanti.sup = 0, # Quantitative supplementary variables

quali.sup = 0, # Qualitative supplementary variables

graph=FALSE)

res.hcpc <- HCPC (res.mca, graph = FALSE, max = 3)
