# Supplementary material for: Morpho-biometric characterization of indigenous chicken ecotypes in north-western Ethiopia
Source: PLoS One. 2023 Jun 2;18(6):e0286299. doi: 10.1371/journal.pone.0286299 (PMC10237646; doi:10.1371/journal.pone.0286299)
Supplement: S4 File — (DOCX) [file pone.0286299.s004.docx]

| | | | **Discriminant Analysis Results** | | --- | | | --- | --- | | | | **The DISCRIM Procedure** | | --- | | | --- | --- | | | | Total Sample Size | 877 | DF Total | 876 | | --- | --- | --- | --- | | Variables | 10 | DF Within Classes | 871 | | Classes | 6 | DF Between Classes | 5 | | | --- | --- | --- | --- | --- | --- | --- | --- | --- | --- | --- | --- | --- | | | Number of Observations Read | 877 | | --- | --- | | Number of Observations Used | 877 | | | | **Class Level Information** | | | | | | | --- | --- | --- | --- | --- | --- | | **DIST** | **Variable Name** | **Frequency** | **Weight** | **Proportion** | **Prior Probability** | | 1 | 1 | 166 | 166.0000 | 0.189282 | 0.189282 | | 2 | 2 | 159 | 159.0000 | 0.181300 | 0.181300 | | 3 | 3 | 131 | 131.0000 | 0.149373 | 0.149373 | | 4 | 4 | 153 | 153.0000 | 0.174458 | 0.174458 | | 5 | 5 | 146 | 146.0000 | 0.166477 | 0.166477 | | 6 | 6 | 122 | 122.0000 | 0.139111 | 0.139111 | | | | **Pooled Covariance Matrix Information** | | | --- | --- | | **Covariance Matrix Rank** | **Natural Log of the Determinant of the Covariance Matrix** | | 10 | -5.92971 | | | |  | | | | --- | --- | --- | --- | --- | --- | --- | --- | --- | --- | --- | --- | --- | --- | --- | --- | --- | --- | --- | --- | --- | --- | --- | --- | --- | --- | --- | --- | --- | --- | --- | --- | --- | --- | --- | --- | --- | --- | --- | --- | --- | --- | --- | --- | --- | --- | --- | --- | --- | --- | --- | --- | --- | --- | --- | --- | --- | --- | --- | --- | --- | --- | --- | --- | --- | --- | --- | --- | --- | --- | --- | --- | --- | --- | --- | --- | --- | --- | --- | --- | --- | --- | | | | **Discriminant Analysis Results** | | --- | | | --- | --- | | | | **The DISCRIM Procedure** | | --- | | | --- | --- | | | | **Generalized Squared Distance to DIST** | | | | | | | | --- | --- | --- | --- | --- | --- | --- | | **From DIST** | **1** | **2** | **3** | **4** | **5** | **6** | | 1 | 3.32904 | 5.42110 | 6.27628 | 6.67862 | 5.95413 | 19.86918 | | 2 | 5.33493 | 3.41521 | 6.34595 | 6.04990 | 7.71863 | 17.09142 | | 3 | 5.80270 | 5.95854 | 3.80262 | 4.42353 | 7.62443 | 18.90829 | | 4 | 6.51552 | 5.97297 | 4.73401 | 3.49214 | 8.15581 | 18.45149 | | 5 | 5.69737 | 7.54804 | 7.84124 | 8.06214 | 3.58580 | 18.57418 | | 6 | 19.25325 | 16.56166 | 18.76594 | 17.99866 | 18.21501 | 3.94497 | | | --- | --- | --- | --- | --- | --- | --- | --- | --- | --- | --- | --- | --- | --- | --- | --- | --- | --- | --- | --- | --- | --- | --- | --- | --- | --- | --- | --- | --- | --- | --- | --- | --- | --- | --- | --- | --- | --- | --- | --- | --- | --- | --- | --- | --- | --- | --- | --- | --- | --- | --- | --- | --- | --- | --- | --- | --- | | |  | | | | | | **Discriminant Analysis Results** | | --- | | | --- | --- | | | | **The DISCRIM Procedure** | | --- | | | --- | --- | | | | **Univariate Test Statistics** | | | | | | | | | --- | --- | --- | --- | --- | --- | --- | --- | | **F Statistics, Num DF=5, Den DF=871** | | | | | | | | | **Variable** | **Total Standard Deviation** | **Pooled Standard Deviation** | **Between Standard Deviation** | **R-Square** | **R-Square / (1-RSq)** | **F Value** | **Pr > F** | | WS | 3.1666 | 2.8274 | 1.5784 | 0.2073 | 0.2615 | 45.55 | <.0001 | | SL | 0.7893 | 0.6786 | 0.4450 | 0.2651 | 0.3608 | 62.85 | <.0001 | | BL | 2.6939 | 2.4841 | 1.1594 | 0.1545 | 0.1828 | 31.84 | <.0001 | | BKL | 0.2953 | 0.2571 | 0.1605 | 0.2465 | 0.3271 | 56.99 | <.0001 | | NL | 1.4619 | 1.2162 | 0.8938 | 0.3118 | 0.4531 | 78.94 | <.0001 | | CL | 0.7443 | 0.7301 | 0.1695 | 0.0433 | 0.0453 | 7.88 | <.0001 | | SC | 0.5889 | 0.4791 | 0.3770 | 0.3418 | 0.5194 | 90.47 | <.0001 | | CC | 1.8490 | 1.7441 | 0.6873 | 0.1153 | 0.1303 | 22.70 | <.0001 | | TC | 1.1790 | 1.1257 | 0.3950 | 0.0936 | 0.1033 | 18.00 | <.0001 | | BW | 0.2285 | 0.2194 | 0.0722 | 0.0833 | 0.0909 | 15.83 | <.0001 | | | --- | --- | --- | --- | --- | --- | --- | --- | --- | --- | --- | --- | --- | --- | --- | --- | --- | --- | --- | --- | --- | --- | --- | --- | --- | --- | --- | --- | --- | --- | --- | --- | --- | --- | --- | --- | --- | --- | --- | --- | --- | --- | --- | --- | --- | --- | --- | --- | --- | --- | --- | --- | --- | --- | --- | --- | --- | --- | --- | --- | --- | --- | --- | --- | --- | --- | --- | --- | --- | --- | --- | --- | --- | --- | --- | --- | --- | --- | --- | --- | --- | --- | --- | --- | --- | --- | --- | --- | --- | --- | --- | --- | --- | --- | --- | --- | --- | --- | --- | --- | --- | --- | --- | --- | --- | | | **Average R-Square** | | | --- | --- | | Unweighted | 0.1862609 | | Weighted by Variance | 0.1824445 | | | | **Multivariate Statistics and F Approximations** | | | | | | | --- | --- | --- | --- | --- | --- | | **S=5 M=2 N=430** | | | | | | | **Statistic** | **Value** | **F Value** | **Num DF** | **Den DF** | **Pr > F** | | Wilks' Lambda | 0.16358683 | 38.35 | 50 | 3934.7 | <.0001 | | Pillai's Trace | 1.34992872 | 32.03 | 50 | 4330 | <.0001 | | Hotelling-Lawley Trace | 2.62114673 | 45.12 | 50 | 2785.2 | <.0001 | | Roy's Greatest Root | 1.64648716 | 142.59 | 10 | 866 | <.0001 | | **NOTE: F Statistic for Roy's Greatest Root is an upper bound.** | | | | | | | | |  | | | | | | **Discriminant Analysis Results** | | --- | | | --- | --- | | | | **The DISCRIM Procedure Canonical Discriminant Analysis** | | --- | | | --- | --- | | | |  | **Canonical Correlation** | **Adjusted Canonical Correlation** | **Approximate Standard Error** | **Squared Canonical Correlation** | **Eigenvalues of Inv(E)*H = CanRsq/(1-CanRsq)** | | | | **Test of H0: The canonical correlations in the current row and all that follow are zero** | | | | | | --- | --- | --- | --- | --- | --- | --- | --- | --- | --- | --- | --- | --- | --- | | **Eigenvalue** | **Difference** | **Proportion** | **Cumulative** | **Likelihood Ratio** | **Approximate F Value** | **Num DF** | **Den DF** | **Pr > F** | | 1 | 0.788759 | 0.784955 | 0.012767 | 0.622141 | 1.6465 | 1.1483 | 0.6282 | 0.6282 | 0.16358683 | 38.35 | 50 | 3934.7 | <.0001 | | 2 | 0.576648 | 0.567766 | 0.022552 | 0.332523 | 0.4982 | 0.2139 | 0.1901 | 0.8182 | 0.43293045 | 22.50 | 36 | 3235.8 | <.0001 | | 3 | 0.470488 | 0.463015 | 0.026308 | 0.221359 | 0.2843 | 0.1575 | 0.1085 | 0.9267 | 0.64860694 | 16.81 | 24 | 2506.5 | <.0001 | | 4 | 0.335500 | 0.325608 | 0.029984 | 0.112560 | 0.1268 | 0.0615 | 0.0484 | 0.9751 | 0.83299850 | 11.82 | 14 | 1730 | <.0001 | | 5 | 0.247682 | 0.243721 | 0.031714 | 0.061346 | 0.0654 |  | 0.0249 | 1.0000 | 0.93865376 | 9.43 | 6 | 866 | <.0001 | | | --- | --- | --- | --- | --- | --- | --- | --- | --- | --- | --- | --- | --- | --- | --- | --- | --- | --- | --- | --- | --- | --- | --- | --- | --- | --- | --- | --- | --- | --- | --- | --- | --- | --- | --- | --- | --- | --- | --- | --- | --- | --- | --- | --- | --- | --- | --- | --- | --- | --- | --- | --- | --- | --- | --- | --- | --- | --- | --- | --- | --- | --- | --- | --- | --- | --- | --- | --- | --- | --- | --- | --- | --- | --- | --- | --- | --- | --- | --- | --- | --- | --- | --- | --- | --- | --- | --- | --- | --- | --- | --- | --- | --- | --- | | |  | | | | | | **Discriminant Analysis Results** | | --- | | | --- | --- | | | | **The DISCRIM Procedure Canonical Discriminant Analysis** | | --- | | | --- | --- | | | | **Total Canonical Structure** | | | | | | | --- | --- | --- | --- | --- | --- | | **Variable** | **Can1** | **Can2** | **Can3** | **Can4** | **Can5** | | WS | 0.376927 | 0.286110 | 0.462247 | 0.552007 | -0.405160 | | SL | -0.339512 | 0.676287 | 0.415656 | 0.158570 | -0.065844 | | BL | 0.236032 | 0.514238 | -0.064111 | 0.497057 | 0.228910 | | BKL | 0.428202 | 0.600455 | 0.195082 | 0.045256 | 0.251289 | | NL | 0.684098 | 0.175000 | -0.044339 | 0.272409 | 0.167087 | | CL | 0.253408 | -0.065188 | -0.034235 | 0.121419 | -0.013265 | | SC | 0.658798 | 0.240321 | 0.466939 | -0.188154 | -0.076634 | | CC | 0.030949 | 0.111891 | 0.459466 | 0.719863 | 0.298517 | | TC | 0.170739 | -0.057985 | 0.550170 | 0.242497 | 0.111052 | | BW | 0.042056 | 0.327436 | 0.130006 | 0.595504 | -0.216771 | | | --- | --- | --- | --- | --- | --- | --- | --- | --- | --- | --- | --- | --- | --- | --- | --- | --- | --- | --- | --- | --- | --- | --- | --- | --- | --- | --- | --- | --- | --- | --- | --- | --- | --- | --- | --- | --- | --- | --- | --- | --- | --- | --- | --- | --- | --- | --- | --- | --- | --- | --- | --- | --- | --- | --- | --- | --- | --- | --- | --- | --- | --- | --- | --- | --- | --- | --- | --- | --- | --- | --- | --- | --- | | | **Between Canonical Structure** | | | | | | | --- | --- | --- | --- | --- | --- | | **Variable** | **Can1** | **Can2** | **Can3** | **Can4** | **Can5** | | WS | 0.653019 | 0.362383 | 0.477691 | 0.406782 | -0.220417 | | SL | -0.520073 | 0.757366 | 0.379793 | 0.103319 | -0.031672 | | BL | 0.473602 | 0.754349 | -0.076733 | 0.424226 | 0.144231 | | BKL | 0.680286 | 0.697412 | 0.184869 | 0.030582 | 0.125362 | | NL | 0.966266 | 0.180711 | -0.037357 | 0.163662 | 0.074109 | | CL | 0.960619 | -0.180662 | -0.077412 | 0.195779 | -0.015790 | | SC | 0.888773 | 0.237026 | 0.375753 | -0.107969 | -0.032464 | | CC | 0.071896 | 0.190029 | 0.636670 | 0.711304 | 0.217758 | | TC | 0.440112 | -0.109273 | 0.845925 | 0.265880 | 0.089889 | | BW | 0.114940 | 0.654236 | 0.211938 | 0.692270 | -0.186034 | | | | **Pooled Within Canonical Structure** | | | | | | | --- | --- | --- | --- | --- | --- | | **Variable** | **Can1** | **Can2** | **Can3** | **Can4** | **Can5** | | WS | 0.260233 | 0.262537 | 0.458123 | 0.584054 | -0.440878 | | SL | -0.243454 | 0.644535 | 0.427858 | 0.174256 | -0.074416 | | BL | 0.157792 | 0.456912 | -0.061525 | 0.509244 | 0.241195 | | BKL | 0.303228 | 0.565138 | 0.198308 | 0.049114 | 0.280467 | | NL | 0.506919 | 0.172350 | -0.047164 | 0.309347 | 0.195142 | | CL | 0.159256 | -0.054450 | -0.030885 | 0.116941 | -0.013139 | | SC | 0.499171 | 0.242014 | 0.507879 | -0.218481 | -0.091517 | | CC | 0.020226 | 0.097188 | 0.431043 | 0.720971 | 0.307482 | | TC | 0.110242 | -0.049760 | 0.509934 | 0.239951 | 0.113012 | | BW | 0.027001 | 0.279402 | 0.119816 | 0.585920 | -0.219350 | | | |  | | | | | | **Discriminant Analysis Results** | | --- | | | --- | --- | | | | **The DISCRIM Procedure Canonical Discriminant Analysis** | | --- | | | --- | --- | | | | **Total-Sample Standardized Canonical Coefficients** | | | | | | | --- | --- | --- | --- | --- | --- | | **Variable** | **Can1** | **Can2** | **Can3** | **Can4** | **Can5** | | WS | 0.3873276469 | -.1873484271 | 0.4235852962 | 0.5942357807 | -.9388472675 | | SL | -.9346483894 | 0.7326472455 | 0.4309590296 | -.4549661342 | 0.1317357557 | | BL | 0.2524709450 | 0.4810458636 | -.5006002636 | 0.2060553482 | 0.4599264395 | | BKL | 0.3518020931 | 0.6366684456 | 0.0286448231 | -.0036365318 | 0.1172954505 | | NL | 0.6122124361 | -.1462049888 | -.3510761294 | 0.0165012501 | 0.3067149105 | | CL | 0.3140737182 | -.1107789574 | -.0911916770 | -.0732843387 | -.0035115494 | | SC | 0.6697619038 | 0.2287268660 | 0.3944165545 | -.6272048676 | 0.0193545668 | | CC | -.4084616479 | -.3842091412 | 0.5594870782 | 0.6594083324 | 0.9093710725 | | TC | 0.1368882469 | -.5469637179 | 0.6392308585 | -.2172152106 | 0.1512289129 | | BW | -.0420828888 | 0.3775793130 | -.7245539857 | 0.2766393005 | -.8519444439 | | | --- | --- | --- | --- | --- | --- | --- | --- | --- | --- | --- | --- | --- | --- | --- | --- | --- | --- | --- | --- | --- | --- | --- | --- | --- | --- | --- | --- | --- | --- | --- | --- | --- | --- | --- | --- | --- | --- | --- | --- | --- | --- | --- | --- | --- | --- | --- | --- | --- | --- | --- | --- | --- | --- | --- | --- | --- | --- | --- | --- | --- | --- | --- | --- | --- | --- | --- | --- | --- | --- | --- | --- | --- | | | **Pooled Within-Class Standardized Canonical Coefficients** | | | | | | | --- | --- | --- | --- | --- | --- | | **Variable** | **Can1** | **Can2** | **Can3** | **Can4** | **Can5** | | WS | 0.3458455989 | -.1672837700 | 0.3782201235 | 0.5305942686 | -.8382985262 | | SL | -.8035154436 | 0.6298554442 | 0.3704946585 | -.3911335207 | 0.1132529788 | | BL | 0.2328114429 | 0.4435876041 | -.4616193348 | 0.1900101532 | 0.4241127152 | | BKL | 0.3062564980 | 0.5542430031 | 0.0249363587 | -.0031657330 | 0.1021099493 | | NL | 0.5093190747 | -.1216325988 | -.2920714426 | 0.0137279169 | 0.2551659281 | | CL | 0.3080802491 | -.1086649624 | -.0894514661 | -.0718858536 | -.0034445385 | | SC | 0.5449190136 | 0.1860924270 | 0.3208977379 | -.5102945627 | 0.0157468966 | | CC | -.3852971645 | -.3624200545 | 0.5277577122 | 0.6220122796 | 0.8577992511 | | TC | 0.1306956832 | -.5222201205 | 0.6103132713 | -.2073888080 | 0.1443876048 | | BW | -.0404076865 | 0.3625489351 | -.6957115150 | 0.2656270625 | -.8180309148 | | | | **Raw Canonical Coefficients** | | | | | | | --- | --- | --- | --- | --- | --- | | **Variable** | **Can1** | **Can2** | **Can3** | **Can4** | **Can5** | | WS | 0.122317524 | -0.059164369 | 0.133767639 | 0.187658821 | -0.296486642 | | SL | -1.184093382 | 0.928180869 | 0.545976156 | -0.576390431 | 0.166894244 | | BL | 0.093719295 | 0.178568189 | -0.185826943 | 0.076489443 | 0.170728484 | | BKL | 1.191402229 | 2.156121922 | 0.097007683 | -0.012315368 | 0.397229192 | | NL | 0.418785344 | -0.100011863 | -0.240154444 | 0.011287719 | 0.209809049 | | CL | 0.421991605 | -0.148843368 | -0.122525763 | -0.098465341 | -0.004718142 | | SC | 1.137265024 | 0.388381399 | 0.669724793 | -1.065002585 | 0.032864323 | | CC | -0.220908582 | -0.207792082 | 0.302587764 | 0.356628242 | 0.491815755 | | TC | 0.116106649 | -0.463926785 | 0.542186451 | -0.184238828 | 0.128270196 | | BW | -0.184169468 | 1.652419387 | -3.170902143 | 1.210670520 | -3.728407429 | | | | **Class Means on Canonical Variables** | | | | | | | --- | --- | --- | --- | --- | --- | | **DIST** | **Can1** | **Can2** | **Can3** | **Can4** | **Can5** | | 1 | 0.750326461 | 0.482570353 | -0.391881635 | 0.567507435 | 0.154461217 | | 2 | 0.301315961 | -0.233377692 | -0.885527219 | -0.425889504 | -0.092882510 | | 3 | 0.596690193 | -0.654155765 | 0.412937577 | 0.239724555 | -0.480154283 | | 4 | 0.492123485 | -0.878922235 | 0.458072704 | -0.137098160 | 0.371843565 | | 5 | 0.408122873 | 1.205965626 | 0.542374811 | -0.336614346 | -0.039592327 | | 6 | -3.159923592 | 0.009007379 | 0.020364840 | 0.100229453 | 0.007511331 | | | |  | | | | | | **Discriminant Analysis Results** | | --- | | | --- | --- | | | | **The DISCRIM Procedure** | | --- | | | --- | --- | | | | **Linear Discriminant Function for DIST** | | | | | | | | --- | --- | --- | --- | --- | --- | --- | | **Variable** | **1** | **2** | **3** | **4** | **5** | **6** | | Constant | -248.33025 | -221.10336 | -239.33207 | -238.41699 | -250.48737 | -219.63546 | | WS | 3.67978 | 3.48809 | 3.96254 | 3.64577 | 3.60796 | 3.24053 | | SL | 0.39045 | 0.51938 | 0.03971 | 0.33894 | 2.46592 | 5.05089 | | BL | 4.28387 | 4.08747 | 3.78352 | 3.84183 | 4.10508 | 3.69541 | | BKL | 22.60338 | 20.39085 | 19.79944 | 19.53770 | 23.78009 | 16.91102 | | NL | -0.07484 | -0.13584 | -0.35563 | -0.21327 | -0.56579 | -1.80015 | | CL | 5.86157 | 5.93813 | 5.90259 | 5.91948 | 5.58497 | 4.27816 | | SC | 5.16588 | 5.09641 | 5.41691 | 5.67024 | 6.63986 | 1.30388 | | CC | 7.33475 | 6.95741 | 7.41941 | 7.78751 | 7.12485 | 8.18278 | | TC | 0.39790 | 0.56157 | 1.32277 | 1.61809 | 0.67079 | 0.45435 | | BW | -70.03578 | -69.85131 | -72.46857 | -76.59664 | -72.11092 | -71.42318 | | | --- | --- | --- | --- | --- | --- | --- | --- | --- | --- | --- | --- | --- | --- | --- | --- | --- | --- | --- | --- | --- | --- | --- | --- | --- | --- | --- | --- | --- | --- | --- | --- | --- | --- | --- | --- | --- | --- | --- | --- | --- | --- | --- | --- | --- | --- | --- | --- | --- | --- | --- | --- | --- | --- | --- | --- | --- | --- | --- | --- | --- | --- | --- | --- | --- | --- | --- | --- | --- | --- | --- | --- | --- | --- | --- | --- | --- | --- | --- | --- | --- | --- | --- | --- | --- | --- | --- | --- | --- | --- | --- | --- | | |  | | | | | | **Discriminant Analysis Results** | | --- | | | --- | --- | | | | **The DISCRIM Procedure Classification Results for Calibration Data: WORK.SORTTEMPTABLESORTED Resubstitution Results using Linear Discriminant Function** | | --- | | | --- | --- | | | | **Posterior Probability of Membership in DIST** | | | | | | | | | | | --- | --- | --- | --- | --- | --- | --- | --- | --- | --- | | **Obs** | **From DIST** | **Classified into DIST** | | **1** | **2** | **3** | **4** | **5** | **6** | | 1 | 1 | 1 |  | 0.6911 | 0.1480 | 0.0697 | 0.0350 | 0.0562 | 0.0000 | | 2 | 1 | 1 |  | 0.5118 | 0.2256 | 0.1216 | 0.1100 | 0.0310 | 0.0000 | | 3 | 1 | 2 | * | 0.2617 | 0.4828 | 0.1144 | 0.0966 | 0.0438 | 0.0007 | | 4 | 1 | 1 |  | 0.4155 | 0.0533 | 0.0909 | 0.0966 | 0.3437 | 0.0002 | | 5 | 1 | 3 | * | 0.1103 | 0.2186 | 0.4230 | 0.0888 | 0.1448 | 0.0145 | | 6 | 1 | 2 | * | 0.2740 | 0.2761 | 0.0705 | 0.1175 | 0.2443 | 0.0175 | | 7 | 1 | 4 | * | 0.1353 | 0.1094 | 0.1641 | 0.4884 | 0.1027 | 0.0000 | | 8 | 1 | 1 |  | 0.3185 | 0.1589 | 0.2427 | 0.1267 | 0.1529 | 0.0002 | | 9 | 1 | 3 | * | 0.3261 | 0.1268 | 0.4022 | 0.0943 | 0.0506 | 0.0000 | | 10 | 1 | 1 |  | 0.7160 | 0.0586 | 0.0379 | 0.0135 | 0.1740 | 0.0000 | | 11 | 1 | 3 | * | 0.1097 | 0.2132 | 0.4325 | 0.0879 | 0.1429 | 0.0138 | | 12 | 1 | 1 |  | 0.2806 | 0.2618 | 0.0809 | 0.1187 | 0.2430 | 0.0151 | | 13 | 1 | 4 | * | 0.2079 | 0.2261 | 0.2772 | 0.2819 | 0.0068 | 0.0002 | | 14 | 1 | 1 |  | 0.3622 | 0.3460 | 0.1205 | 0.0866 | 0.0846 | 0.0001 | | 15 | 1 | 5 | * | 0.4234 | 0.0285 | 0.0248 | 0.0184 | 0.5050 | 0.0000 | | 16 | 1 | 1 |  | 0.3526 | 0.2761 | 0.0632 | 0.0622 | 0.2394 | 0.0064 | | 17 | 1 | 5 | * | 0.3582 | 0.0446 | 0.1019 | 0.1197 | 0.3754 | 0.0002 | | 18 | 1 | 3 | * | 0.1087 | 0.2357 | 0.3595 | 0.0882 | 0.1808 | 0.0271 | | 19 | 1 | 2 | * | 0.1439 | 0.4915 | 0.1558 | 0.1914 | 0.0166 | 0.0009 | | 20 | 1 | 2 | * | 0.2634 | 0.4768 | 0.1185 | 0.0969 | 0.0438 | 0.0007 | | 21 | 1 | 4 | * | 0.0788 | 0.0919 | 0.1562 | 0.6270 | 0.0460 | 0.0000 | | 22 | 1 | 1 |  | 0.7378 | 0.1373 | 0.0290 | 0.0172 | 0.0787 | 0.0000 | | 23 | 1 | 5 | * | 0.3735 | 0.0559 | 0.0853 | 0.1005 | 0.3845 | 0.0003 | | 24 | 1 | 2 | * | 0.2425 | 0.2943 | 0.1560 | 0.1988 | 0.0947 | 0.0136 | | 25 | 1 | 1 |  | 0.5131 | 0.2177 | 0.1290 | 0.1095 | 0.0307 | 0.0000 | | 26 | 1 | 2 | * | 0.2304 | 0.5078 | 0.1268 | 0.0950 | 0.0392 | 0.0009 | | 27 | 1 | 1 |  | 0.7487 | 0.1406 | 0.0261 | 0.0126 | 0.0720 | 0.0000 | | 28 | 1 | 1 |  | 0.3244 | 0.1605 | 0.2801 | 0.1823 | 0.0527 | 0.0000 | | 29 | 1 | 1 |  | 0.5388 | 0.2727 | 0.1078 | 0.0507 | 0.0298 | 0.0002 | | 30 | 1 | 3 | * | 0.2138 | 0.1188 | 0.4900 | 0.1511 | 0.0263 | 0.0000 | | 31 | 1 | 3 | * | 0.1510 | 0.2918 | 0.4072 | 0.0677 | 0.0559 | 0.0263 | | 32 | 1 | 1 |  | 0.3696 | 0.1106 | 0.1531 | 0.0684 | 0.2982 | 0.0001 | | 33 | 1 | 3 | * | 0.1352 | 0.2629 | 0.4202 | 0.0963 | 0.0583 | 0.0271 | | 34 | 1 | 3 | * | 0.3097 | 0.1212 | 0.3646 | 0.1412 | 0.0632 | 0.0000 | | 35 | 1 | 1 |  | 0.5832 | 0.1847 | 0.1005 | 0.0998 | 0.0318 | 0.0000 | | 36 | 1 | 1 |  | 0.3452 | 0.1040 | 0.2147 | 0.2078 | 0.1272 | 0.0012 | | 37 | 1 | 4 | * | 0.1923 | 0.0985 | 0.1698 | 0.4049 | 0.1345 | 0.0000 | | 38 | 1 | 3 | * | 0.2041 | 0.1051 | 0.5240 | 0.1423 | 0.0244 | 0.0000 | | 39 | 1 | 3 | * | 0.1764 | 0.2028 | 0.3695 | 0.0802 | 0.1605 | 0.0105 | | 40 | 1 | 2 | * | 0.2113 | 0.3380 | 0.1308 | 0.1639 | 0.1327 | 0.0233 | | 41 | 1 | 1 |  | 0.3465 | 0.2996 | 0.0552 | 0.0674 | 0.2193 | 0.0119 | | 42 | 1 | 1 |  | 0.5598 | 0.2687 | 0.1181 | 0.0400 | 0.0132 | 0.0001 | | 43 | 1 | 3 | * | 0.0981 | 0.2315 | 0.3258 | 0.1200 | 0.1915 | 0.0331 | | 44 | 1 | 2 | * | 0.1434 | 0.5408 | 0.1283 | 0.1700 | 0.0164 | 0.0010 | | 45 | 1 | 4 | * | 0.0940 | 0.1215 | 0.1468 | 0.5852 | 0.0524 | 0.0001 | | 46 | 1 | 1 |  | 0.5705 | 0.2497 | 0.1320 | 0.0352 | 0.0125 | 0.0001 | | 47 | 1 | 1 |  | 0.3668 | 0.1925 | 0.0324 | 0.0408 | 0.3654 | 0.0021 | | 48 | 1 | 1 |  | 0.6391 | 0.0583 | 0.0261 | 0.0378 | 0.2387 | 0.0001 | | 49 | 1 | 4 | * | 0.2357 | 0.0929 | 0.2534 | 0.3101 | 0.1078 | 0.0001 | | 50 | 1 | 2 | * | 0.3444 | 0.4257 | 0.0452 | 0.0316 | 0.1524 | 0.0007 | | 51 | 1 | 3 | * | 0.1301 | 0.0813 | 0.3220 | 0.2554 | 0.2110 | 0.0003 | | 52 | 1 | 5 | * | 0.2996 | 0.0229 | 0.0731 | 0.0099 | 0.5938 | 0.0007 | | 53 | 1 | 1 |  | 0.4599 | 0.1666 | 0.2331 | 0.0774 | 0.0627 | 0.0003 | | 54 | 1 | 5 | * | 0.1381 | 0.0460 | 0.2119 | 0.1515 | 0.4525 | 0.0001 | | 55 | 1 | 5 | * | 0.3370 | 0.0449 | 0.0147 | 0.0115 | 0.5209 | 0.0710 | | 56 | 1 | 1 |  | 0.5528 | 0.0195 | 0.0289 | 0.0193 | 0.3792 | 0.0003 | | 57 | 1 | 1 |  | 0.5346 | 0.0451 | 0.2922 | 0.0565 | 0.0716 | 0.0000 | | 58 | 1 | 1 |  | 0.3734 | 0.1385 | 0.0371 | 0.1767 | 0.2743 | 0.0000 | | 59 | 1 | 5 | * | 0.2840 | 0.0516 | 0.0097 | 0.0169 | 0.6373 | 0.0004 | | 60 | 1 | 5 | * | 0.4320 | 0.0147 | 0.0076 | 0.0126 | 0.5331 | 0.0000 | | 61 | 1 | 5 | * | 0.1382 | 0.0470 | 0.2062 | 0.1522 | 0.4563 | 0.0001 | | 62 | 1 | 1 |  | 0.3634 | 0.0770 | 0.1701 | 0.2388 | 0.1506 | 0.0000 | | 63 | 1 | 5 | * | 0.3004 | 0.1002 | 0.0131 | 0.0107 | 0.5756 | 0.0001 | | 64 | 1 | 5 | * | 0.2694 | 0.0148 | 0.0441 | 0.0048 | 0.6667 | 0.0002 | | 65 | 1 | 1 |  | 0.3130 | 0.1211 | 0.2679 | 0.1325 | 0.1652 | 0.0003 | | 66 | 1 | 5 | * | 0.0883 | 0.0275 | 0.1346 | 0.1314 | 0.6182 | 0.0001 | | 67 | 1 | 5 | * | 0.3171 | 0.0327 | 0.0150 | 0.0144 | 0.6108 | 0.0099 | | 68 | 1 | 1 |  | 0.4816 | 0.0165 | 0.0209 | 0.0119 | 0.4688 | 0.0003 | | 69 | 1 | 1 |  | 0.4879 | 0.0347 | 0.3288 | 0.0673 | 0.0813 | 0.0000 | | 70 | 1 | 1 |  | 0.3499 | 0.1397 | 0.0353 | 0.2350 | 0.2401 | 0.0000 | | 71 | 1 | 1 |  | 0.3589 | 0.1225 | 0.0396 | 0.1661 | 0.3129 | 0.0000 | | 72 | 1 | 5 | * | 0.1759 | 0.0606 | 0.0161 | 0.0441 | 0.7013 | 0.0020 | | 73 | 1 | 1 |  | 0.7125 | 0.0580 | 0.0248 | 0.0453 | 0.1593 | 0.0000 | | 74 | 1 | 4 | * | 0.1825 | 0.1044 | 0.2654 | 0.3997 | 0.0479 | 0.0001 | | 75 | 1 | 2 | * | 0.3280 | 0.4136 | 0.0707 | 0.0955 | 0.0899 | 0.0024 | | 76 | 1 | 5 | * | 0.2405 | 0.0268 | 0.0724 | 0.0178 | 0.6349 | 0.0076 | | 77 | 1 | 3 | * | 0.1962 | 0.1430 | 0.3739 | 0.2213 | 0.0636 | 0.0019 | | 78 | 1 | 5 | * | 0.1255 | 0.0488 | 0.2387 | 0.2154 | 0.3714 | 0.0002 | | 79 | 1 | 5 | * | 0.3853 | 0.0747 | 0.0307 | 0.0297 | 0.4061 | 0.0735 | | 80 | 1 | 1 |  | 0.5669 | 0.0248 | 0.0352 | 0.0223 | 0.3501 | 0.0008 | | 81 | 1 | 1 |  | 0.4048 | 0.0471 | 0.3985 | 0.0968 | 0.0528 | 0.0000 | | 82 | 1 | 1 |  | 0.4586 | 0.1630 | 0.2391 | 0.0769 | 0.0621 | 0.0003 | | 83 | 1 | 2 | * | 0.2538 | 0.4845 | 0.0802 | 0.1165 | 0.0502 | 0.0148 | | 84 | 1 | 1 |  | 0.6460 | 0.1242 | 0.0523 | 0.1068 | 0.0703 | 0.0004 | | 85 | 1 | 4 | * | 0.1149 | 0.1107 | 0.3226 | 0.4321 | 0.0196 | 0.0001 | | 86 | 1 | 2 | * | 0.1626 | 0.6148 | 0.0961 | 0.0896 | 0.0343 | 0.0027 | | 87 | 1 | 5 | * | 0.2698 | 0.0285 | 0.0452 | 0.0122 | 0.6400 | 0.0042 | | 88 | 1 | 3 | * | 0.1048 | 0.1332 | 0.4269 | 0.3189 | 0.0135 | 0.0027 | | 89 | 1 | 3 | * | 0.1287 | 0.0774 | 0.3370 | 0.2509 | 0.2057 | 0.0002 | | 90 | 1 | 1 |  | 0.3425 | 0.0941 | 0.0290 | 0.0268 | 0.2648 | 0.2428 | | 91 | 1 | 1 |  | 0.6307 | 0.0457 | 0.0513 | 0.0338 | 0.2368 | 0.0017 | | 92 | 1 | 3 | * | 0.2751 | 0.0538 | 0.5225 | 0.1251 | 0.0235 | 0.0000 | | 93 | 1 | 2 | * | 0.2592 | 0.2922 | 0.0486 | 0.2623 | 0.1372 | 0.0005 | | 94 | 1 | 5 | * | 0.3249 | 0.0682 | 0.0276 | 0.0363 | 0.4312 | 0.1118 | | 95 | 1 | 1 |  | 0.5099 | 0.0239 | 0.0345 | 0.0216 | 0.4083 | 0.0018 | | 96 | 1 | 5 | * | 0.3419 | 0.0438 | 0.0157 | 0.0116 | 0.5209 | 0.0660 | | 97 | 1 | 1 |  | 0.4037 | 0.0503 | 0.3863 | 0.1003 | 0.0593 | 0.0000 | | 98 | 1 | 1 |  | 0.3056 | 0.1330 | 0.0302 | 0.2899 | 0.2412 | 0.0000 | | 99 | 1 | 2 | * | 0.2553 | 0.4403 | 0.0999 | 0.1402 | 0.0518 | 0.0125 | | 100 | 1 | 1 |  | 0.6395 | 0.1354 | 0.0450 | 0.1075 | 0.0721 | 0.0005 | | 101 | 1 | 1 |  | 0.3378 | 0.0946 | 0.0278 | 0.0265 | 0.2631 | 0.2503 | | 102 | 1 | 1 |  | 0.5957 | 0.0414 | 0.0596 | 0.0446 | 0.2568 | 0.0019 | | 103 | 1 | 3 | * | 0.2432 | 0.0486 | 0.5304 | 0.1571 | 0.0206 | 0.0000 | | 104 | 1 | 3 | * | 0.2247 | 0.0732 | 0.3206 | 0.2858 | 0.0957 | 0.0001 | | 105 | 1 | 2 | * | 0.3500 | 0.4163 | 0.0486 | 0.0319 | 0.1526 | 0.0007 | | 106 | 1 | 5 | * | 0.2996 | 0.0229 | 0.0731 | 0.0099 | 0.5938 | 0.0007 | | 107 | 1 | 1 |  | 0.9362 | 0.0180 | 0.0318 | 0.0077 | 0.0062 | 0.0000 | | 108 | 1 | 1 |  | 0.7534 | 0.0184 | 0.0503 | 0.0291 | 0.1487 | 0.0002 | | 109 | 1 | 1 |  | 0.4167 | 0.1233 | 0.2601 | 0.1074 | 0.0925 | 0.0000 | | 110 | 1 | 2 | * | 0.3069 | 0.6195 | 0.0011 | 0.0066 | 0.0658 | 0.0000 | | 111 | 1 | 3 | * | 0.3411 | 0.1195 | 0.3702 | 0.1489 | 0.0203 | 0.0000 | | 112 | 1 | 4 | * | 0.2983 | 0.1404 | 0.0974 | 0.3787 | 0.0851 | 0.0000 | | 113 | 1 | 1 |  | 0.4364 | 0.4275 | 0.0558 | 0.0698 | 0.0106 | 0.0000 | | 114 | 1 | 3 | * | 0.3351 | 0.0973 | 0.3613 | 0.1863 | 0.0200 | 0.0000 | | 115 | 1 | 1 |  | 0.4059 | 0.2095 | 0.0280 | 0.0096 | 0.3337 | 0.0133 | | 116 | 1 | 2 | * | 0.2536 | 0.4674 | 0.0169 | 0.0152 | 0.2460 | 0.0009 | | 117 | 1 | 4 | * | 0.3730 | 0.0453 | 0.0893 | 0.3935 | 0.0990 | 0.0000 | | 118 | 1 | 1 |  | 0.4341 | 0.3299 | 0.0656 | 0.0592 | 0.1110 | 0.0001 | | 119 | 1 | 2 | * | 0.2939 | 0.3716 | 0.0140 | 0.0130 | 0.3073 | 0.0003 | | 120 | 1 | 1 |  | 0.8685 | 0.0144 | 0.0833 | 0.0228 | 0.0111 | 0.0000 | | 121 | 1 | 1 |  | 0.5228 | 0.0930 | 0.1892 | 0.0687 | 0.1263 | 0.0000 | | 122 | 1 | 2 | * | 0.3719 | 0.4658 | 0.0007 | 0.0045 | 0.1570 | 0.0000 | | 123 | 1 | 1 |  | 0.8056 | 0.0159 | 0.0333 | 0.0147 | 0.1304 | 0.0001 | | 124 | 1 | 1 |  | 0.4930 | 0.1137 | 0.0503 | 0.0853 | 0.2578 | 0.0000 | | 125 | 1 | 1 |  | 0.7535 | 0.0180 | 0.0517 | 0.0290 | 0.1477 | 0.0002 | | 126 | 1 | 1 |  | 0.4981 | 0.3438 | 0.0640 | 0.0717 | 0.0224 | 0.0000 | | 127 | 1 | 1 |  | 0.3921 | 0.0590 | 0.3308 | 0.1784 | 0.0395 | 0.0000 | | 128 | 1 | 1 |  | 0.4294 | 0.1490 | 0.0253 | 0.0129 | 0.3685 | 0.0148 | | 129 | 1 | 1 |  | 0.5070 | 0.0427 | 0.0890 | 0.2743 | 0.0869 | 0.0000 | | 130 | 1 | 1 |  | 0.4347 | 0.2366 | 0.0439 | 0.0444 | 0.2403 | 0.0002 | | 131 | 1 | 1 |  | 0.8817 | 0.0180 | 0.0679 | 0.0201 | 0.0123 | 0.0000 | | 132 | 1 | 1 |  | 0.7558 | 0.0235 | 0.0473 | 0.0192 | 0.1538 | 0.0004 | | 133 | 1 | 1 |  | 0.3638 | 0.1490 | 0.2880 | 0.1215 | 0.0777 | 0.0000 | | 134 | 1 | 2 | * | 0.2836 | 0.6528 | 0.0011 | 0.0066 | 0.0558 | 0.0000 | | 135 | 1 | 1 |  | 0.6928 | 0.2113 | 0.0699 | 0.0226 | 0.0033 | 0.0000 | | 136 | 1 | 1 |  | 0.4707 | 0.1854 | 0.0774 | 0.1537 | 0.1128 | 0.0000 | | 137 | 1 | 1 |  | 0.4287 | 0.3827 | 0.0726 | 0.0993 | 0.0166 | 0.0000 | | 138 | 1 | 1 |  | 0.7480 | 0.1713 | 0.0504 | 0.0204 | 0.0099 | 0.0000 | | 139 | 1 | 1 |  | 0.3978 | 0.1872 | 0.0263 | 0.0113 | 0.3665 | 0.0108 | | 140 | 1 | 2 | * | 0.2379 | 0.4814 | 0.0101 | 0.0108 | 0.2590 | 0.0008 | | 141 | 1 | 1 |  | 0.4025 | 0.2478 | 0.0520 | 0.0573 | 0.2402 | 0.0002 | | 142 | 1 | 1 |  | 0.9188 | 0.0251 | 0.0423 | 0.0100 | 0.0038 | 0.0000 | | 143 | 1 | 1 |  | 0.6748 | 0.0082 | 0.0882 | 0.1453 | 0.0731 | 0.0103 | | 144 | 1 | 4 | * | 0.2993 | 0.1356 | 0.1034 | 0.3774 | 0.0842 | 0.0000 | | 145 | 1 | 2 | * | 0.2488 | 0.7024 | 0.0010 | 0.0051 | 0.0427 | 0.0000 | | 146 | 1 | 1 |  | 0.5976 | 0.2613 | 0.0777 | 0.0598 | 0.0038 | 0.0000 | | 147 | 1 | 4 | * | 0.2493 | 0.2520 | 0.1180 | 0.3366 | 0.0440 | 0.0000 | | 148 | 1 | 2 | * | 0.3940 | 0.4633 | 0.0648 | 0.0679 | 0.0100 | 0.0000 | | 149 | 1 | 3 | * | 0.1959 | 0.1235 | 0.4288 | 0.2412 | 0.0106 | 0.0000 | | 150 | 1 | 5 | * | 0.3402 | 0.2441 | 0.0357 | 0.0155 | 0.3528 | 0.0117 | | 151 | 1 | 1 |  | 0.6905 | 0.1890 | 0.0757 | 0.0402 | 0.0045 | 0.0000 | | 152 | 1 | 2 | * | 0.1741 | 0.5268 | 0.0150 | 0.0143 | 0.2682 | 0.0014 | | 153 | 1 | 4 | * | 0.2290 | 0.0544 | 0.0812 | 0.5217 | 0.1137 | 0.0000 | | 154 | 1 | 2 | * | 0.4150 | 0.4364 | 0.0527 | 0.0572 | 0.0387 | 0.0000 | | 155 | 1 | 1 |  | 0.8841 | 0.0187 | 0.0644 | 0.0203 | 0.0125 | 0.0000 | | 156 | 1 | 1 |  | 0.7553 | 0.0244 | 0.0447 | 0.0194 | 0.1559 | 0.0004 | | 157 | 1 | 1 |  | 0.3741 | 0.1821 | 0.2297 | 0.1288 | 0.0852 | 0.0000 | | 158 | 1 | 1 |  | 0.6916 | 0.1857 | 0.0780 | 0.0401 | 0.0045 | 0.0000 | | 159 | 1 | 2 | * | 0.2763 | 0.6609 | 0.0010 | 0.0065 | 0.0552 | 0.0000 | | 160 | 1 | 1 |  | 0.6896 | 0.2186 | 0.0658 | 0.0227 | 0.0034 | 0.0000 | | 161 | 1 | 3 | * | 0.2547 | 0.1801 | 0.3623 | 0.1570 | 0.0458 | 0.0000 | | 162 | 1 | 1 |  | 0.4072 | 0.1095 | 0.2929 | 0.1032 | 0.0872 | 0.0000 | | 163 | 1 | 2 | * | 0.3145 | 0.6110 | 0.0012 | 0.0068 | 0.0665 | 0.0000 | | 164 | 1 | 1 |  | 0.4681 | 0.1916 | 0.0727 | 0.1538 | 0.1138 | 0.0000 | | 165 | 1 | 1 |  | 0.4423 | 0.4170 | 0.0598 | 0.0703 | 0.0106 | 0.0000 | | 166 | 1 | 3 | * | 0.3376 | 0.0999 | 0.3539 | 0.1883 | 0.0203 | 0.0000 | | 167 | 2 | 2 |  | 0.0266 | 0.4805 | 0.1522 | 0.3348 | 0.0050 | 0.0009 | | 168 | 2 | 2 |  | 0.1184 | 0.5453 | 0.2020 | 0.1305 | 0.0037 | 0.0001 | | 169 | 2 | 4 | * | 0.0644 | 0.0559 | 0.1012 | 0.7467 | 0.0279 | 0.0039 | | 170 | 2 | 4 | * | 0.1784 | 0.2554 | 0.2301 | 0.3074 | 0.0285 | 0.0001 | | 171 | 2 | 4 | * | 0.1201 | 0.1535 | 0.1291 | 0.5319 | 0.0612 | 0.0042 | | 172 | 2 | 2 |  | 0.1459 | 0.4611 | 0.2270 | 0.1312 | 0.0325 | 0.0022 | | 173 | 2 | 1 | * | 0.4369 | 0.1375 | 0.2226 | 0.0502 | 0.1527 | 0.0000 | | 174 | 2 | 3 | * | 0.1926 | 0.2245 | 0.2796 | 0.2644 | 0.0388 | 0.0001 | | 175 | 2 | 5 | * | 0.3197 | 0.1359 | 0.0234 | 0.0136 | 0.5074 | 0.0000 | | 176 | 2 | 2 |  | 0.0742 | 0.8743 | 0.0164 | 0.0189 | 0.0160 | 0.0001 | | 177 | 2 | 2 |  | 0.0459 | 0.5702 | 0.1345 | 0.2421 | 0.0061 | 0.0012 | | 178 | 2 | 2 |  | 0.1667 | 0.5062 | 0.1739 | 0.1419 | 0.0108 | 0.0005 | | 179 | 2 | 2 |  | 0.2835 | 0.4213 | 0.1624 | 0.0659 | 0.0666 | 0.0003 | | 180 | 2 | 2 |  | 0.1048 | 0.4562 | 0.0761 | 0.0894 | 0.2728 | 0.0007 | | 181 | 2 | 2 |  | 0.1356 | 0.3305 | 0.2488 | 0.2515 | 0.0336 | 0.0001 | | 182 | 2 | 4 | * | 0.1494 | 0.1524 | 0.1688 | 0.4598 | 0.0664 | 0.0033 | | 183 | 2 | 2 |  | 0.3656 | 0.3743 | 0.0909 | 0.0448 | 0.1234 | 0.0009 | | 184 | 2 | 1 | * | 0.4840 | 0.1283 | 0.1726 | 0.0304 | 0.1846 | 0.0000 | | 185 | 2 | 2 |  | 0.2156 | 0.5073 | 0.1726 | 0.0691 | 0.0345 | 0.0010 | | 186 | 2 | 2 |  | 0.1525 | 0.3790 | 0.3000 | 0.1318 | 0.0339 | 0.0028 | | 187 | 2 | 2 |  | 0.0863 | 0.8431 | 0.0164 | 0.0189 | 0.0349 | 0.0004 | | 188 | 2 | 2 |  | 0.2211 | 0.3959 | 0.2503 | 0.0755 | 0.0570 | 0.0001 | | 189 | 2 | 2 |  | 0.0238 | 0.4447 | 0.1957 | 0.3334 | 0.0023 | 0.0001 | | 190 | 2 | 2 |  | 0.1109 | 0.5827 | 0.1896 | 0.1145 | 0.0024 | 0.0000 | | 191 | 2 | 2 |  | 0.0794 | 0.6351 | 0.1035 | 0.0988 | 0.0832 | 0.0000 | | 192 | 2 | 5 | * | 0.3280 | 0.1193 | 0.0202 | 0.0089 | 0.5235 | 0.0000 | | 193 | 2 | 4 | * | 0.0573 | 0.1023 | 0.1450 | 0.6763 | 0.0186 | 0.0005 | | 194 | 2 | 2 |  | 0.1324 | 0.3354 | 0.2453 | 0.2710 | 0.0155 | 0.0004 | | 195 | 2 | 2 |  | 0.1036 | 0.4900 | 0.2467 | 0.1362 | 0.0226 | 0.0010 | | 196 | 2 | 1 | * | 0.3319 | 0.1427 | 0.2827 | 0.0772 | 0.1655 | 0.0000 | | 197 | 2 | 1 | * | 0.4119 | 0.2458 | 0.0260 | 0.0157 | 0.3005 | 0.0000 | | 198 | 2 | 2 |  | 0.0663 | 0.9010 | 0.0091 | 0.0109 | 0.0127 | 0.0001 | | 199 | 2 | 2 |  | 0.1016 | 0.5286 | 0.2099 | 0.1357 | 0.0229 | 0.0012 | | 200 | 2 | 2 |  | 0.0617 | 0.8170 | 0.0520 | 0.0682 | 0.0009 | 0.0002 | | 201 | 2 | 4 | * | 0.0722 | 0.1054 | 0.1361 | 0.6694 | 0.0116 | 0.0053 | | 202 | 2 | 4 | * | 0.0765 | 0.1474 | 0.1234 | 0.6351 | 0.0135 | 0.0041 | | 203 | 2 | 2 |  | 0.1459 | 0.4611 | 0.2270 | 0.1312 | 0.0325 | 0.0022 | | 204 | 2 | 2 |  | 0.1288 | 0.5624 | 0.1920 | 0.0942 | 0.0195 | 0.0031 | | 205 | 2 | 1 | * | 0.4269 | 0.2199 | 0.2530 | 0.0463 | 0.0539 | 0.0001 | | 206 | 2 | 1 | * | 0.3960 | 0.2342 | 0.0282 | 0.0210 | 0.3206 | 0.0000 | | 207 | 2 | 2 |  | 0.0479 | 0.9075 | 0.0140 | 0.0186 | 0.0115 | 0.0005 | | 208 | 2 | 2 |  | 0.0786 | 0.6408 | 0.0996 | 0.0981 | 0.0829 | 0.0000 | | 209 | 2 | 2 |  | 0.1184 | 0.3463 | 0.1849 | 0.3153 | 0.0350 | 0.0001 | | 210 | 2 | 4 | * | 0.0572 | 0.1062 | 0.1369 | 0.6803 | 0.0189 | 0.0005 | | 211 | 2 | 4 | * | 0.1784 | 0.2554 | 0.2301 | 0.3074 | 0.0285 | 0.0001 | | 212 | 2 | 2 |  | 0.0188 | 0.5576 | 0.1473 | 0.2749 | 0.0011 | 0.0003 | | 213 | 2 | 1 | * | 0.3353 | 0.1527 | 0.2623 | 0.0787 | 0.1709 | 0.0000 | | 214 | 2 | 1 | * | 0.3722 | 0.2550 | 0.0450 | 0.0266 | 0.3012 | 0.0000 | | 215 | 2 | 2 |  | 0.0664 | 0.8941 | 0.0103 | 0.0151 | 0.0141 | 0.0001 | | 216 | 2 | 2 |  | 0.2197 | 0.4789 | 0.1970 | 0.0695 | 0.0341 | 0.0008 | | 217 | 2 | 2 |  | 0.0191 | 0.5444 | 0.1582 | 0.2770 | 0.0011 | 0.0003 | | 218 | 2 | 4 | * | 0.1201 | 0.1535 | 0.1291 | 0.5319 | 0.0612 | 0.0042 | | 219 | 2 | 2 |  | 0.1276 | 0.5268 | 0.2172 | 0.1069 | 0.0174 | 0.0041 | | 220 | 2 | 1 | * | 0.3950 | 0.1927 | 0.2869 | 0.0621 | 0.0632 | 0.0000 | | 221 | 2 | 1 | * | 0.3907 | 0.2645 | 0.0474 | 0.0264 | 0.2709 | 0.0000 | | 222 | 2 | 2 |  | 0.0484 | 0.9113 | 0.0138 | 0.0169 | 0.0086 | 0.0010 | | 223 | 2 | 2 |  | 0.1168 | 0.6032 | 0.1864 | 0.0910 | 0.0026 | 0.0000 | | 224 | 2 | 2 |  | 0.0738 | 0.5811 | 0.1115 | 0.1341 | 0.0995 | 0.0000 | | 225 | 2 | 2 |  | 0.1210 | 0.5063 | 0.2378 | 0.1311 | 0.0037 | 0.0000 | | 226 | 2 | 4 | * | 0.0640 | 0.0611 | 0.0872 | 0.7542 | 0.0287 | 0.0048 | | 227 | 2 | 1 | * | 0.4369 | 0.1375 | 0.2226 | 0.0502 | 0.1527 | 0.0000 | | 228 | 2 | 1 | * | 0.3894 | 0.3470 | 0.0547 | 0.1821 | 0.0267 | 0.0002 | | 229 | 2 | 3 | * | 0.1217 | 0.2079 | 0.3124 | 0.2487 | 0.1085 | 0.0009 | | 230 | 2 | 2 |  | 0.0879 | 0.4475 | 0.1298 | 0.2269 | 0.0776 | 0.0302 | | 231 | 2 | 2 |  | 0.2680 | 0.5338 | 0.0626 | 0.1240 | 0.0117 | 0.0000 | | 232 | 2 | 1 | * | 0.3250 | 0.1498 | 0.0523 | 0.2714 | 0.1447 | 0.0568 | | 233 | 2 | 2 |  | 0.2449 | 0.6043 | 0.0729 | 0.0247 | 0.0527 | 0.0006 | | 234 | 2 | 2 |  | 0.0372 | 0.9049 | 0.0149 | 0.0291 | 0.0138 | 0.0001 | | 235 | 2 | 4 | * | 0.2958 | 0.1289 | 0.0999 | 0.4087 | 0.0664 | 0.0003 | | 236 | 2 | 2 |  | 0.2845 | 0.3853 | 0.0754 | 0.0391 | 0.2158 | 0.0000 | | 237 | 2 | 2 |  | 0.2424 | 0.6098 | 0.0701 | 0.0245 | 0.0525 | 0.0006 | | 238 | 2 | 2 |  | 0.1917 | 0.6615 | 0.0771 | 0.0386 | 0.0308 | 0.0003 | | 239 | 2 | 6 | * | 0.0534 | 0.3283 | 0.0517 | 0.1153 | 0.0300 | 0.4212 | | 240 | 2 | 2 |  | 0.0929 | 0.7514 | 0.0852 | 0.0455 | 0.0247 | 0.0003 | | 241 | 2 | 2 |  | 0.2997 | 0.3943 | 0.0744 | 0.0295 | 0.2021 | 0.0000 | | 242 | 2 | 2 |  | 0.1360 | 0.6981 | 0.0280 | 0.0773 | 0.0590 | 0.0016 | | 243 | 2 | 2 |  | 0.1649 | 0.5594 | 0.0640 | 0.0249 | 0.1866 | 0.0002 | | 244 | 2 | 1 | * | 0.3510 | 0.3410 | 0.0483 | 0.2318 | 0.0277 | 0.0002 | | 245 | 2 | 2 |  | 0.0763 | 0.4232 | 0.1104 | 0.2778 | 0.0774 | 0.0350 | | 246 | 2 | 4 | * | 0.2970 | 0.1222 | 0.1092 | 0.4061 | 0.0653 | 0.0002 | | 247 | 2 | 2 |  | 0.2287 | 0.4921 | 0.0592 | 0.2074 | 0.0127 | 0.0000 | | 248 | 2 | 4 | * | 0.2790 | 0.1402 | 0.0440 | 0.3290 | 0.1428 | 0.0650 | | 249 | 2 | 2 |  | 0.0339 | 0.9007 | 0.0133 | 0.0375 | 0.0145 | 0.0001 | | 250 | 2 | 4 | * | 0.2490 | 0.1183 | 0.0824 | 0.4857 | 0.0643 | 0.0003 | | 251 | 2 | 2 |  | 0.2629 | 0.3883 | 0.0683 | 0.0510 | 0.2294 | 0.0000 | | 252 | 2 | 2 |  | 0.2261 | 0.6204 | 0.0641 | 0.0323 | 0.0563 | 0.0007 | | 253 | 2 | 2 |  | 0.1777 | 0.6686 | 0.0700 | 0.0505 | 0.0328 | 0.0004 | | 254 | 2 | 6 | * | 0.0354 | 0.2617 | 0.0431 | 0.1346 | 0.0250 | 0.5001 | | 255 | 2 | 4 | * | 0.1075 | 0.2003 | 0.2706 | 0.3103 | 0.1103 | 0.0011 | | 256 | 2 | 2 |  | 0.0829 | 0.7247 | 0.0842 | 0.0797 | 0.0281 | 0.0003 | | 257 | 2 | 2 |  | 0.1077 | 0.6262 | 0.0958 | 0.1427 | 0.0267 | 0.0009 | | 258 | 2 | 2 |  | 0.1282 | 0.6774 | 0.0282 | 0.1018 | 0.0625 | 0.0018 | | 259 | 2 | 2 |  | 0.1517 | 0.5655 | 0.0579 | 0.0325 | 0.1921 | 0.0003 | | 260 | 2 | 4 | * | 0.2495 | 0.1163 | 0.0850 | 0.4850 | 0.0639 | 0.0003 | | 261 | 2 | 2 |  | 0.2333 | 0.6042 | 0.0720 | 0.0330 | 0.0569 | 0.0006 | | 262 | 2 | 2 |  | 0.2082 | 0.5897 | 0.1194 | 0.0493 | 0.0331 | 0.0002 | | 263 | 2 | 2 |  | 0.1191 | 0.6348 | 0.1080 | 0.1117 | 0.0256 | 0.0007 | | 264 | 2 | 6 | * | 0.0551 | 0.3053 | 0.0695 | 0.1626 | 0.0332 | 0.3743 | | 265 | 2 | 3 | * | 0.1220 | 0.2125 | 0.3046 | 0.2503 | 0.1096 | 0.0010 | | 266 | 2 | 2 |  | 0.0960 | 0.7499 | 0.0887 | 0.0410 | 0.0241 | 0.0002 | | 267 | 2 | 2 |  | 0.1168 | 0.5714 | 0.1340 | 0.1501 | 0.0271 | 0.0006 | | 268 | 2 | 2 |  | 0.0996 | 0.7315 | 0.0235 | 0.0781 | 0.0665 | 0.0007 | | 269 | 2 | 2 |  | 0.1680 | 0.6142 | 0.0482 | 0.0227 | 0.1469 | 0.0001 | | 270 | 2 | 1 | * | 0.3220 | 0.3041 | 0.0515 | 0.2942 | 0.0280 | 0.0002 | | 271 | 2 | 1 | * | 0.3552 | 0.3130 | 0.0577 | 0.2463 | 0.0276 | 0.0002 | | 272 | 2 | 3 | * | 0.1223 | 0.2172 | 0.2969 | 0.2518 | 0.1107 | 0.0010 | | 273 | 2 | 2 |  | 0.0940 | 0.7462 | 0.0887 | 0.0459 | 0.0248 | 0.0003 | | 274 | 2 | 2 |  | 0.1225 | 0.6164 | 0.1210 | 0.1137 | 0.0258 | 0.0007 | | 275 | 2 | 2 |  | 0.1331 | 0.7112 | 0.0253 | 0.0713 | 0.0573 | 0.0017 | | 276 | 2 | 2 |  | 0.1596 | 0.5364 | 0.0699 | 0.0334 | 0.2004 | 0.0003 | | 277 | 2 | 6 | * | 0.0560 | 0.3312 | 0.0574 | 0.1201 | 0.0310 | 0.4044 | | 278 | 2 | 2 |  | 0.2704 | 0.5283 | 0.0650 | 0.1246 | 0.0117 | 0.0000 | | 279 | 2 | 1 | * | 0.3269 | 0.1478 | 0.0541 | 0.2721 | 0.1445 | 0.0546 | | 280 | 2 | 2 |  | 0.2883 | 0.3758 | 0.0808 | 0.0393 | 0.2156 | 0.0000 | | 281 | 2 | 2 |  | 0.0385 | 0.9012 | 0.0163 | 0.0299 | 0.0141 | 0.0001 | | 282 | 2 | 2 |  | 0.1999 | 0.6389 | 0.0900 | 0.0397 | 0.0312 | 0.0003 | | 283 | 2 | 4 | * | 0.0515 | 0.1554 | 0.2470 | 0.5292 | 0.0169 | 0.0000 | | 284 | 2 | 1 | * | 0.3708 | 0.2974 | 0.0677 | 0.2367 | 0.0272 | 0.0001 | | 285 | 2 | 2 |  | 0.0910 | 0.4208 | 0.1547 | 0.2309 | 0.0775 | 0.0251 | | 286 | 2 | 2 |  | 0.2727 | 0.5228 | 0.0674 | 0.1253 | 0.0117 | 0.0000 | | 287 | 2 | 1 | * | 0.4727 | 0.2910 | 0.1319 | 0.0187 | 0.0848 | 0.0009 | | 288 | 2 | 1 | * | 0.3339 | 0.1398 | 0.0619 | 0.2742 | 0.1434 | 0.0468 | | 289 | 2 | 2 |  | 0.0398 | 0.8972 | 0.0179 | 0.0307 | 0.0144 | 0.0001 | | 290 | 2 | 4 | * | 0.2461 | 0.1287 | 0.0707 | 0.4883 | 0.0659 | 0.0004 | | 291 | 2 | 2 |  | 0.2530 | 0.4112 | 0.0570 | 0.0499 | 0.2288 | 0.0000 | | 292 | 2 | 2 |  | 0.1026 | 0.3714 | 0.2916 | 0.2211 | 0.0134 | 0.0000 | | 293 | 2 | 2 |  | 0.2177 | 0.6493 | 0.0507 | 0.0308 | 0.0510 | 0.0006 | | 294 | 2 | 2 |  | 0.1777 | 0.6686 | 0.0700 | 0.0505 | 0.0328 | 0.0004 | | 295 | 2 | 6 | * | 0.0471 | 0.3190 | 0.0396 | 0.1036 | 0.0275 | 0.4632 | | 296 | 2 | 4 | * | 0.1236 | 0.2558 | 0.2392 | 0.2614 | 0.1185 | 0.0015 | | 297 | 2 | 2 |  | 0.0814 | 0.7975 | 0.0563 | 0.0413 | 0.0232 | 0.0004 | | 298 | 2 | 2 |  | 0.1131 | 0.6637 | 0.0891 | 0.1080 | 0.0252 | 0.0009 | | 299 | 2 | 2 |  | 0.1282 | 0.6774 | 0.0282 | 0.1018 | 0.0625 | 0.0018 | | 300 | 2 | 2 |  | 0.1435 | 0.5841 | 0.0474 | 0.0311 | 0.1936 | 0.0004 | | 301 | 2 | 2 |  | 0.1073 | 0.4295 | 0.0602 | 0.0445 | 0.2427 | 0.1157 | | 302 | 2 | 4 | * | 0.0589 | 0.0597 | 0.3413 | 0.5151 | 0.0246 | 0.0004 | | 303 | 2 | 2 |  | 0.2489 | 0.4203 | 0.0530 | 0.0494 | 0.2283 | 0.0000 | | 304 | 2 | 1 | * | 0.2495 | 0.2327 | 0.1957 | 0.2343 | 0.0878 | 0.0000 | | 305 | 2 | 5 | * | 0.2380 | 0.1143 | 0.0483 | 0.0135 | 0.5669 | 0.0190 | | 306 | 2 | 2 |  | 0.0921 | 0.4098 | 0.1657 | 0.2320 | 0.0773 | 0.0233 | | 307 | 2 | 1 | * | 0.6194 | 0.2197 | 0.0686 | 0.0419 | 0.0505 | 0.0000 | | 308 | 2 | 2 |  | 0.1865 | 0.4707 | 0.1415 | 0.0773 | 0.1238 | 0.0002 | | 309 | 2 | 4 | * | 0.0519 | 0.1722 | 0.2160 | 0.5422 | 0.0177 | 0.0000 | | 310 | 2 | 4 | * | 0.2649 | 0.1312 | 0.0734 | 0.4621 | 0.0680 | 0.0003 | | 311 | 2 | 2 |  | 0.0405 | 0.8951 | 0.0187 | 0.0311 | 0.0145 | 0.0001 | | 312 | 2 | 2 |  | 0.2140 | 0.6461 | 0.0527 | 0.0311 | 0.0553 | 0.0008 | | 313 | 2 | 6 | * | 0.0437 | 0.2930 | 0.0415 | 0.1333 | 0.0283 | 0.4602 | | 314 | 2 | 2 |  | 0.2213 | 0.6308 | 0.0593 | 0.0318 | 0.0559 | 0.0008 | | 315 | 2 | 2 |  | 0.1191 | 0.6348 | 0.1080 | 0.1117 | 0.0256 | 0.0007 | | 316 | 2 | 2 |  | 0.0769 | 0.8132 | 0.0475 | 0.0395 | 0.0226 | 0.0004 | | 317 | 2 | 2 |  | 0.0982 | 0.7412 | 0.0768 | 0.0614 | 0.0219 | 0.0005 | | 318 | 2 | 2 |  | 0.0837 | 0.7983 | 0.0505 | 0.0386 | 0.0284 | 0.0005 | | 319 | 2 | 2 |  | 0.1373 | 0.5292 | 0.0555 | 0.0431 | 0.2346 | 0.0002 | | 320 | 2 | 2 |  | 0.1126 | 0.4549 | 0.0559 | 0.0337 | 0.2296 | 0.1133 | | 321 | 2 | 2 |  | 0.2774 | 0.5117 | 0.0726 | 0.1266 | 0.0117 | 0.0000 | | 322 | 2 | 1 | * | 0.3355 | 0.1378 | 0.0640 | 0.2746 | 0.1431 | 0.0450 | | 323 | 2 | 2 |  | 0.1298 | 0.6733 | 0.0294 | 0.1028 | 0.0629 | 0.0018 | | 324 | 2 | 4 | * | 0.2665 | 0.1271 | 0.0840 | 0.4560 | 0.0660 | 0.0003 | | 325 | 2 | 2 |  | 0.2570 | 0.4021 | 0.0613 | 0.0504 | 0.2291 | 0.0000 | | 326 | 3 | 2 | * | 0.1922 | 0.4398 | 0.1644 | 0.1605 | 0.0431 | 0.0000 | | 327 | 3 | 4 | * | 0.0627 | 0.0205 | 0.4470 | 0.4668 | 0.0030 | 0.0000 | | 328 | 3 | 2 | * | 0.2190 | 0.3807 | 0.1994 | 0.1625 | 0.0384 | 0.0000 | | 329 | 3 | 4 | * | 0.0509 | 0.2524 | 0.2591 | 0.3307 | 0.1016 | 0.0054 | | 330 | 3 | 2 | * | 0.1070 | 0.3864 | 0.2207 | 0.2520 | 0.0330 | 0.0008 | | 331 | 3 | 1 | * | 0.4951 | 0.2054 | 0.1493 | 0.0539 | 0.0955 | 0.0009 | | 332 | 3 | 5 | * | 0.1150 | 0.0805 | 0.2519 | 0.2610 | 0.2753 | 0.0163 | | 333 | 3 | 3 |  | 0.0513 | 0.0540 | 0.4993 | 0.3924 | 0.0029 | 0.0000 | | 334 | 3 | 2 | * | 0.1741 | 0.4264 | 0.1503 | 0.2047 | 0.0446 | 0.0000 | | 335 | 3 | 4 | * | 0.0532 | 0.0188 | 0.3698 | 0.5553 | 0.0030 | 0.0000 | | 336 | 3 | 4 | * | 0.0435 | 0.2350 | 0.2169 | 0.3987 | 0.0998 | 0.0061 | | 337 | 3 | 2 | * | 0.0968 | 0.3740 | 0.1863 | 0.3081 | 0.0337 | 0.0010 | | 338 | 3 | 1 | * | 0.4704 | 0.2128 | 0.1390 | 0.0723 | 0.1043 | 0.0011 | | 339 | 3 | 4 | * | 0.0994 | 0.0759 | 0.2136 | 0.3187 | 0.2737 | 0.0188 | | 340 | 3 | 3 |  | 0.0660 | 0.0619 | 0.6136 | 0.2557 | 0.0029 | 0.0000 | | 341 | 3 | 2 | * | 0.1760 | 0.3922 | 0.1514 | 0.2377 | 0.0426 | 0.0000 | | 342 | 3 | 3 |  | 0.0790 | 0.0278 | 0.4597 | 0.4302 | 0.0034 | 0.0000 | | 343 | 3 | 2 | * | 0.0661 | 0.3093 | 0.2954 | 0.2199 | 0.1042 | 0.0051 | | 344 | 3 | 2 | * | 0.1269 | 0.4041 | 0.2416 | 0.1938 | 0.0328 | 0.0007 | | 345 | 3 | 1 | * | 0.5161 | 0.2120 | 0.1417 | 0.0403 | 0.0891 | 0.0008 | | 346 | 3 | 5 | * | 0.1315 | 0.0894 | 0.2699 | 0.2135 | 0.2797 | 0.0159 | | 347 | 3 | 4 | * | 0.0624 | 0.0205 | 0.4468 | 0.4674 | 0.0029 | 0.0000 | | 348 | 3 | 4 | * | 0.0509 | 0.2524 | 0.2591 | 0.3307 | 0.1016 | 0.0054 | | 349 | 3 | 3 |  | 0.0507 | 0.0524 | 0.5075 | 0.3865 | 0.0029 | 0.0000 | | 350 | 3 | 2 | * | 0.1136 | 0.3946 | 0.2121 | 0.2450 | 0.0338 | 0.0008 | | 351 | 3 | 1 | * | 0.4951 | 0.2054 | 0.1493 | 0.0539 | 0.0955 | 0.0009 | | 352 | 3 | 5 | * | 0.1150 | 0.0805 | 0.2519 | 0.2610 | 0.2753 | 0.0163 | | 353 | 3 | 3 |  | 0.0513 | 0.0540 | 0.4993 | 0.3924 | 0.0029 | 0.0000 | | 354 | 3 | 2 | * | 0.1732 | 0.4324 | 0.1454 | 0.2043 | 0.0446 | 0.0000 | | 355 | 3 | 4 | * | 0.1063 | 0.0798 | 0.2080 | 0.3014 | 0.2846 | 0.0199 | | 356 | 3 | 4 | * | 0.0508 | 0.2376 | 0.2813 | 0.3264 | 0.0992 | 0.0047 | | 357 | 3 | 4 | * | 0.1125 | 0.0689 | 0.2728 | 0.2766 | 0.2527 | 0.0165 | | 358 | 3 | 2 | * | 0.1009 | 0.3649 | 0.2436 | 0.2579 | 0.0318 | 0.0008 | | 359 | 3 | 1 | * | 0.4954 | 0.2016 | 0.1536 | 0.0537 | 0.0948 | 0.0008 | | 360 | 3 | 3 |  | 0.2171 | 0.0234 | 0.3942 | 0.1832 | 0.1821 | 0.0000 | | 361 | 3 | 2 | * | 0.0107 | 0.4465 | 0.1864 | 0.3511 | 0.0052 | 0.0000 | | 362 | 3 | 2 | * | 0.0393 | 0.4487 | 0.1093 | 0.3521 | 0.0505 | 0.0001 | | 363 | 3 | 5 | * | 0.2163 | 0.0215 | 0.2473 | 0.1017 | 0.4132 | 0.0000 | | 364 | 3 | 4 | * | 0.0122 | 0.0086 | 0.3789 | 0.5862 | 0.0141 | 0.0000 | | 365 | 3 | 3 |  | 0.0805 | 0.0816 | 0.6638 | 0.1342 | 0.0399 | 0.0000 | | 366 | 3 | 4 | * | 0.1267 | 0.0414 | 0.1472 | 0.6623 | 0.0224 | 0.0000 | | 367 | 3 | 1 | * | 0.6154 | 0.1939 | 0.1046 | 0.0744 | 0.0116 | 0.0000 | | 368 | 3 | 3 |  | 0.1607 | 0.0229 | 0.5256 | 0.2073 | 0.0834 | 0.0000 | | 369 | 3 | 2 | * | 0.0072 | 0.4403 | 0.1876 | 0.3607 | 0.0041 | 0.0000 | | 370 | 3 | 4 | * | 0.0058 | 0.0054 | 0.3313 | 0.6520 | 0.0056 | 0.0000 | | 371 | 3 | 2 | * | 0.0276 | 0.4418 | 0.1268 | 0.3813 | 0.0224 | 0.0000 | | 372 | 3 | 4 | * | 0.0082 | 0.0073 | 0.3732 | 0.6045 | 0.0068 | 0.0000 | | 373 | 3 | 3 |  | 0.0523 | 0.0716 | 0.7193 | 0.1408 | 0.0160 | 0.0000 | | 374 | 3 | 4 | * | 0.0856 | 0.0380 | 0.1558 | 0.7095 | 0.0110 | 0.0000 | | 375 | 3 | 2 | * | 0.0481 | 0.4859 | 0.0890 | 0.2380 | 0.1386 | 0.0005 | | 376 | 3 | 1 | * | 0.5138 | 0.2646 | 0.1269 | 0.0825 | 0.0122 | 0.0000 | | 377 | 3 | 5 | * | 0.2163 | 0.0215 | 0.2473 | 0.1017 | 0.4132 | 0.0000 | | 378 | 3 | 2 | * | 0.0146 | 0.5258 | 0.1816 | 0.2675 | 0.0105 | 0.0001 | | 379 | 3 | 3 |  | 0.0511 | 0.0704 | 0.7226 | 0.1395 | 0.0165 | 0.0000 | | 380 | 3 | 2 | * | 0.0481 | 0.4859 | 0.0890 | 0.2380 | 0.1386 | 0.0005 | | 381 | 3 | 4 | * | 0.0183 | 0.0111 | 0.4152 | 0.5235 | 0.0319 | 0.0001 | | 382 | 3 | 3 |  | 0.1060 | 0.0953 | 0.5827 | 0.0978 | 0.1180 | 0.0001 | | 383 | 3 | 4 | * | 0.1881 | 0.0537 | 0.1507 | 0.5467 | 0.0607 | 0.0000 | | 384 | 3 | 1 | * | 0.6714 | 0.1828 | 0.0816 | 0.0454 | 0.0188 | 0.0000 | | 385 | 3 | 3 |  | 0.0805 | 0.0816 | 0.6638 | 0.1342 | 0.0399 | 0.0000 | | 386 | 3 | 4 | * | 0.1267 | 0.0414 | 0.1472 | 0.6623 | 0.0224 | 0.0000 | | 387 | 3 | 1 | * | 0.6154 | 0.1939 | 0.1046 | 0.0744 | 0.0116 | 0.0000 | | 388 | 3 | 3 |  | 0.1607 | 0.0229 | 0.5256 | 0.2073 | 0.0834 | 0.0000 | | 389 | 3 | 2 | * | 0.0072 | 0.4403 | 0.1876 | 0.3607 | 0.0041 | 0.0000 | | 390 | 3 | 1 | * | 0.4920 | 0.3138 | 0.1164 | 0.0641 | 0.0137 | 0.0000 | | 391 | 3 | 4 | * | 0.0046 | 0.0047 | 0.3386 | 0.6473 | 0.0048 | 0.0000 | | 392 | 3 | 3 |  | 0.0523 | 0.0716 | 0.7193 | 0.1408 | 0.0160 | 0.0000 | | 393 | 3 | 4 | * | 0.0660 | 0.0291 | 0.1357 | 0.7598 | 0.0094 | 0.0000 | | 394 | 3 | 1 | * | 0.5009 | 0.2906 | 0.1240 | 0.0712 | 0.0133 | 0.0000 | | 395 | 3 | 3 |  | 0.0117 | 0.0123 | 0.5268 | 0.4411 | 0.0081 | 0.0000 | | 396 | 3 | 3 |  | 0.0494 | 0.0639 | 0.7401 | 0.1318 | 0.0148 | 0.0000 | | 397 | 3 | 4 | * | 0.0855 | 0.0373 | 0.1601 | 0.7062 | 0.0109 | 0.0000 | | 398 | 3 | 3 |  | 0.1057 | 0.0914 | 0.5856 | 0.1020 | 0.1152 | 0.0001 | | 399 | 3 | 1 | * | 0.5259 | 0.2360 | 0.1270 | 0.1002 | 0.0110 | 0.0000 | | 400 | 3 | 5 | * | 0.2176 | 0.0224 | 0.2352 | 0.1030 | 0.4217 | 0.0001 | | 401 | 3 | 2 | * | 0.0128 | 0.4832 | 0.1650 | 0.3285 | 0.0104 | 0.0001 | | 402 | 3 | 4 | * | 0.1838 | 0.0505 | 0.1486 | 0.5590 | 0.0581 | 0.0000 | | 403 | 3 | 2 | * | 0.0276 | 0.4418 | 0.1268 | 0.3813 | 0.0224 | 0.0000 | | 404 | 3 | 4 | * | 0.0179 | 0.0105 | 0.4308 | 0.5099 | 0.0308 | 0.0001 | | 405 | 3 | 3 |  | 0.1046 | 0.0922 | 0.5913 | 0.0962 | 0.1156 | 0.0001 | | 406 | 3 | 4 | * | 0.1884 | 0.0559 | 0.1427 | 0.5513 | 0.0617 | 0.0000 | | 407 | 3 | 1 | * | 0.6654 | 0.1993 | 0.0702 | 0.0458 | 0.0193 | 0.0000 | | 408 | 3 | 2 | * | 0.0276 | 0.4418 | 0.1268 | 0.3813 | 0.0224 | 0.0000 | | 409 | 3 | 4 | * | 0.0074 | 0.0067 | 0.3839 | 0.5956 | 0.0063 | 0.0000 | | 410 | 3 | 3 |  | 0.0464 | 0.0581 | 0.7564 | 0.1246 | 0.0144 | 0.0000 | | 411 | 3 | 3 |  | 0.0654 | 0.1413 | 0.4305 | 0.3062 | 0.0558 | 0.0007 | | 412 | 3 | 1 | * | 0.4581 | 0.0182 | 0.1369 | 0.0421 | 0.3443 | 0.0003 | | 413 | 3 | 3 |  | 0.0431 | 0.0525 | 0.4849 | 0.3607 | 0.0348 | 0.0240 | | 414 | 3 | 3 |  | 0.0406 | 0.0583 | 0.5425 | 0.2832 | 0.0420 | 0.0333 | | 415 | 3 | 1 | * | 0.4114 | 0.0113 | 0.1413 | 0.0528 | 0.3820 | 0.0012 | | 416 | 3 | 3 |  | 0.0568 | 0.1479 | 0.4200 | 0.3258 | 0.0491 | 0.0004 | | 417 | 3 | 1 | * | 0.5233 | 0.0715 | 0.3318 | 0.0491 | 0.0243 | 0.0000 | | 418 | 3 | 3 |  | 0.0336 | 0.0562 | 0.5251 | 0.3302 | 0.0347 | 0.0201 | | 419 | 3 | 1 | * | 0.4188 | 0.0159 | 0.1617 | 0.0553 | 0.3480 | 0.0003 | | 420 | 3 | 3 |  | 0.1409 | 0.2170 | 0.3016 | 0.2182 | 0.1220 | 0.0004 | | 421 | 3 | 2 | * | 0.0818 | 0.4205 | 0.2665 | 0.2146 | 0.0156 | 0.0011 | | 422 | 3 | 3 |  | 0.0163 | 0.0404 | 0.4975 | 0.4293 | 0.0113 | 0.0052 | | 423 | 3 | 3 |  | 0.0475 | 0.1357 | 0.4129 | 0.3643 | 0.0396 | 0.0002 | | 424 | 3 | 1 | * | 0.4482 | 0.0717 | 0.3891 | 0.0676 | 0.0233 | 0.0000 | | 425 | 3 | 2 | * | 0.0882 | 0.3723 | 0.2803 | 0.2390 | 0.0183 | 0.0018 | | 426 | 3 | 3 |  | 0.0302 | 0.0574 | 0.5081 | 0.3637 | 0.0303 | 0.0103 | | 427 | 3 | 1 | * | 0.3800 | 0.0151 | 0.1872 | 0.0700 | 0.3476 | 0.0001 | | 428 | 3 | 3 |  | 0.1678 | 0.2241 | 0.2708 | 0.2217 | 0.1154 | 0.0002 | | 429 | 3 | 2 | * | 0.0625 | 0.3360 | 0.2861 | 0.3023 | 0.0126 | 0.0005 | | 430 | 3 | 3 |  | 0.0559 | 0.0662 | 0.5042 | 0.3086 | 0.0393 | 0.0258 | | 431 | 3 | 1 | * | 0.4297 | 0.0146 | 0.1452 | 0.0423 | 0.3677 | 0.0005 | | 432 | 3 | 3 |  | 0.0772 | 0.1333 | 0.4431 | 0.2816 | 0.0635 | 0.0014 | | 433 | 3 | 1 | * | 0.6332 | 0.0686 | 0.2468 | 0.0240 | 0.0274 | 0.0001 | | 434 | 3 | 3 |  | 0.0385 | 0.0458 | 0.5085 | 0.2933 | 0.0421 | 0.0717 | | 435 | 3 | 5 | * | 0.3562 | 0.0106 | 0.1251 | 0.0401 | 0.4677 | 0.0003 | | 436 | 3 | 3 |  | 0.1632 | 0.1992 | 0.2933 | 0.1777 | 0.1657 | 0.0010 | | 437 | 3 | 2 | * | 0.1055 | 0.3356 | 0.3028 | 0.2319 | 0.0205 | 0.0037 | | 438 | 3 | 3 |  | 0.1412 | 0.1964 | 0.3047 | 0.2155 | 0.1416 | 0.0006 | | 439 | 3 | 3 |  | 0.0957 | 0.1394 | 0.4475 | 0.2374 | 0.0777 | 0.0023 | | 440 | 3 | 1 | * | 0.6791 | 0.0683 | 0.2061 | 0.0160 | 0.0304 | 0.0001 | | 441 | 3 | 3 |  | 0.0462 | 0.0507 | 0.4980 | 0.2498 | 0.0545 | 0.1008 | | 442 | 3 | 5 | * | 0.3983 | 0.0097 | 0.1035 | 0.0301 | 0.4567 | 0.0018 | | 443 | 3 | 3 |  | 0.1891 | 0.1972 | 0.2471 | 0.1338 | 0.2306 | 0.0022 | | 444 | 3 | 2 | * | 0.1161 | 0.3460 | 0.2849 | 0.2216 | 0.0262 | 0.0052 | | 445 | 3 | 3 |  | 0.0541 | 0.3123 | 0.3264 | 0.2932 | 0.0134 | 0.0007 | | 446 | 3 | 3 |  | 0.0768 | 0.0458 | 0.4866 | 0.1512 | 0.0744 | 0.1651 | | 447 | 3 | 1 | * | 0.5889 | 0.0713 | 0.2836 | 0.0311 | 0.0250 | 0.0000 | | 448 | 3 | 3 |  | 0.0265 | 0.0534 | 0.5174 | 0.3639 | 0.0280 | 0.0108 | | 449 | 3 | 3 |  | 0.1413 | 0.1929 | 0.2989 | 0.2264 | 0.1398 | 0.0007 | | 450 | 3 | 3 |  | 0.0608 | 0.1082 | 0.4357 | 0.3364 | 0.0575 | 0.0013 | | 451 | 3 | 5 | * | 0.4028 | 0.0129 | 0.1337 | 0.0431 | 0.4067 | 0.0008 | | 452 | 3 | 3 |  | 0.1720 | 0.2334 | 0.2779 | 0.1489 | 0.1667 | 0.0012 | | 453 | 3 | 3 |  | 0.0981 | 0.1512 | 0.4211 | 0.2457 | 0.0813 | 0.0026 | | 454 | 3 | 1 | * | 0.6491 | 0.0577 | 0.2395 | 0.0231 | 0.0305 | 0.0001 | | 455 | 3 | 1 | * | 0.5301 | 0.0697 | 0.3288 | 0.0474 | 0.0240 | 0.0000 | | 456 | 3 | 3 |  | 0.0270 | 0.0495 | 0.4984 | 0.3714 | 0.0317 | 0.0220 | | 457 | 4 | 4 |  | 0.2845 | 0.1105 | 0.0796 | 0.4293 | 0.0956 | 0.0004 | | 458 | 4 | 4 |  | 0.0249 | 0.0822 | 0.1708 | 0.6801 | 0.0410 | 0.0011 | | 459 | 4 | 4 |  | 0.0046 | 0.0036 | 0.3623 | 0.6255 | 0.0041 | 0.0000 | | 460 | 4 | 4 |  | 0.0096 | 0.0857 | 0.0799 | 0.7857 | 0.0113 | 0.0278 | | 461 | 4 | 4 |  | 0.0249 | 0.0822 | 0.1708 | 0.6801 | 0.0410 | 0.0011 | | 462 | 4 | 1 | * | 0.3949 | 0.0819 | 0.2840 | 0.2258 | 0.0134 | 0.0000 | | 463 | 4 | 2 | * | 0.1997 | 0.4651 | 0.0562 | 0.2088 | 0.0694 | 0.0007 | | 464 | 4 | 4 |  | 0.0160 | 0.0150 | 0.2037 | 0.7495 | 0.0158 | 0.0000 | | 465 | 4 | 4 |  | 0.2936 | 0.1108 | 0.0758 | 0.3877 | 0.1317 | 0.0004 | | 466 | 4 | 4 |  | 0.0237 | 0.0193 | 0.2544 | 0.6697 | 0.0329 | 0.0000 | | 467 | 4 | 4 |  | 0.0052 | 0.0039 | 0.3735 | 0.6114 | 0.0061 | 0.0000 | | 468 | 4 | 4 |  | 0.0101 | 0.0825 | 0.0820 | 0.7846 | 0.0160 | 0.0247 | | 469 | 4 | 4 |  | 0.0314 | 0.0992 | 0.1899 | 0.6140 | 0.0643 | 0.0012 | | 470 | 4 | 1 | * | 0.4126 | 0.0815 | 0.2816 | 0.2057 | 0.0186 | 0.0000 | | 471 | 4 | 2 | * | 0.2105 | 0.4591 | 0.0565 | 0.1791 | 0.0942 | 0.0007 | | 472 | 4 | 4 |  | 0.3242 | 0.1011 | 0.0735 | 0.3423 | 0.1572 | 0.0016 | | 473 | 4 | 4 |  | 0.0251 | 0.0189 | 0.2800 | 0.6141 | 0.0614 | 0.0005 | | 474 | 4 | 2 | * | 0.1571 | 0.6031 | 0.0530 | 0.1616 | 0.0219 | 0.0035 | | 475 | 4 | 4 |  | 0.0062 | 0.0038 | 0.3943 | 0.5877 | 0.0079 | 0.0000 | | 476 | 4 | 4 |  | 0.0189 | 0.1379 | 0.0998 | 0.5611 | 0.0260 | 0.1562 | | 477 | 4 | 4 |  | 0.0481 | 0.1204 | 0.2133 | 0.5223 | 0.0890 | 0.0070 | | 478 | 4 | 1 | * | 0.4549 | 0.0757 | 0.2651 | 0.1820 | 0.0223 | 0.0000 | | 479 | 4 | 2 | * | 0.2303 | 0.4305 | 0.0525 | 0.1685 | 0.1154 | 0.0028 | | 480 | 4 | 4 |  | 0.2348 | 0.0946 | 0.0763 | 0.5205 | 0.0735 | 0.0003 | | 481 | 4 | 4 |  | 0.0046 | 0.0036 | 0.3623 | 0.6255 | 0.0041 | 0.0000 | | 482 | 4 | 4 |  | 0.0034 | 0.0027 | 0.3183 | 0.6729 | 0.0028 | 0.0000 | | 483 | 4 | 4 |  | 0.0081 | 0.0634 | 0.1014 | 0.8071 | 0.0079 | 0.0120 | | 484 | 4 | 4 |  | 0.0135 | 0.0477 | 0.1245 | 0.7902 | 0.0236 | 0.0005 | | 485 | 4 | 4 |  | 0.0351 | 0.0331 | 0.2944 | 0.5510 | 0.0858 | 0.0006 | | 486 | 4 | 1 | * | 0.3305 | 0.0711 | 0.2987 | 0.2891 | 0.0106 | 0.0000 | | 487 | 4 | 2 | * | 0.1785 | 0.4309 | 0.0584 | 0.2740 | 0.0577 | 0.0005 | | 488 | 4 | 4 |  | 0.2812 | 0.1202 | 0.0683 | 0.4317 | 0.0980 | 0.0005 | | 489 | 4 | 4 |  | 0.0213 | 0.0214 | 0.2211 | 0.7068 | 0.0293 | 0.0001 | | 490 | 4 | 4 |  | 0.0046 | 0.0036 | 0.3623 | 0.6255 | 0.0041 | 0.0000 | | 491 | 4 | 4 |  | 0.0096 | 0.0857 | 0.0799 | 0.7857 | 0.0113 | 0.0278 | | 492 | 4 | 1 | * | 0.3949 | 0.0819 | 0.2840 | 0.2258 | 0.0134 | 0.0000 | | 493 | 4 | 2 | * | 0.1997 | 0.4651 | 0.0562 | 0.2088 | 0.0694 | 0.0007 | | 494 | 4 | 1 | * | 0.4547 | 0.0463 | 0.2339 | 0.2201 | 0.0450 | 0.0000 | | 495 | 4 | 2 | * | 0.0990 | 0.4693 | 0.0605 | 0.3444 | 0.0245 | 0.0023 | | 496 | 4 | 1 | * | 0.3487 | 0.0695 | 0.0534 | 0.2536 | 0.2747 | 0.0001 | | 497 | 4 | 4 |  | 0.0158 | 0.0209 | 0.2869 | 0.6621 | 0.0143 | 0.0000 | | 498 | 4 | 4 |  | 0.0108 | 0.0025 | 0.3163 | 0.6391 | 0.0313 | 0.0000 | | 499 | 4 | 4 |  | 0.0082 | 0.1333 | 0.1065 | 0.7009 | 0.0037 | 0.0474 | | 500 | 4 | 4 |  | 0.0413 | 0.0797 | 0.1553 | 0.5854 | 0.1378 | 0.0005 | | 501 | 4 | 4 |  | 0.0179 | 0.1291 | 0.1400 | 0.6718 | 0.0153 | 0.0259 | | 502 | 4 | 3 | * | 0.3215 | 0.1351 | 0.3477 | 0.1907 | 0.0050 | 0.0000 | | 503 | 4 | 2 | * | 0.2055 | 0.5228 | 0.0533 | 0.1215 | 0.0966 | 0.0004 | | 504 | 4 | 4 |  | 0.2910 | 0.1195 | 0.0620 | 0.3460 | 0.1803 | 0.0013 | | 505 | 4 | 4 |  | 0.0238 | 0.0220 | 0.1987 | 0.7277 | 0.0276 | 0.0001 | | 506 | 4 | 4 |  | 0.0051 | 0.0032 | 0.4101 | 0.5742 | 0.0074 | 0.0000 | | 507 | 4 | 4 |  | 0.0239 | 0.2169 | 0.0946 | 0.4538 | 0.0328 | 0.1780 | | 508 | 4 | 4 |  | 0.0431 | 0.1422 | 0.1798 | 0.5275 | 0.1019 | 0.0055 | | 509 | 4 | 1 | * | 0.5020 | 0.0861 | 0.2288 | 0.1611 | 0.0221 | 0.0000 | | 510 | 4 | 2 | * | 0.1741 | 0.4453 | 0.0523 | 0.2701 | 0.0575 | 0.0006 | | 511 | 4 | 4 |  | 0.0209 | 0.0191 | 0.2501 | 0.6822 | 0.0278 | 0.0001 | | 512 | 4 | 4 |  | 0.3263 | 0.1671 | 0.0599 | 0.3352 | 0.1108 | 0.0006 | | 513 | 4 | 3 | * | 0.0835 | 0.2068 | 0.3690 | 0.3124 | 0.0281 | 0.0002 | | 514 | 4 | 4 |  | 0.1397 | 0.1004 | 0.2939 | 0.3041 | 0.1609 | 0.0010 | | 515 | 4 | 2 | * | 0.1632 | 0.3378 | 0.3225 | 0.1719 | 0.0046 | 0.0000 | | 516 | 4 | 4 |  | 0.0349 | 0.1783 | 0.0692 | 0.6317 | 0.0851 | 0.0008 | | 517 | 4 | 2 | * | 0.0711 | 0.4074 | 0.0689 | 0.3076 | 0.1422 | 0.0026 | | 518 | 4 | 4 |  | 0.1427 | 0.0720 | 0.2849 | 0.3349 | 0.1653 | 0.0003 | | 519 | 4 | 2 | * | 0.0644 | 0.4374 | 0.0734 | 0.3550 | 0.0636 | 0.0061 | | 520 | 4 | 4 |  | 0.0147 | 0.0114 | 0.4148 | 0.5234 | 0.0356 | 0.0001 | | 521 | 4 | 4 |  | 0.0499 | 0.3380 | 0.0556 | 0.4896 | 0.0667 | 0.0002 | | 522 | 4 | 4 |  | 0.1135 | 0.2868 | 0.2164 | 0.3804 | 0.0028 | 0.0000 | | 523 | 4 | 3 | * | 0.1291 | 0.2175 | 0.3912 | 0.2547 | 0.0073 | 0.0003 | | 524 | 4 | 4 |  | 0.0525 | 0.1503 | 0.3815 | 0.4008 | 0.0148 | 0.0001 | | 525 | 4 | 4 |  | 0.0975 | 0.0495 | 0.3103 | 0.4023 | 0.1383 | 0.0021 | | 526 | 4 | 1 | * | 0.3708 | 0.3090 | 0.0723 | 0.1078 | 0.1399 | 0.0000 | | 527 | 4 | 2 | * | 0.0842 | 0.3473 | 0.0785 | 0.3291 | 0.1566 | 0.0043 | | 528 | 4 | 3 | * | 0.1786 | 0.2870 | 0.3294 | 0.1997 | 0.0053 | 0.0000 | | 529 | 4 | 2 | * | 0.1920 | 0.4456 | 0.1396 | 0.2051 | 0.0175 | 0.0002 | | 530 | 4 | 2 | * | 0.3008 | 0.3402 | 0.0963 | 0.1437 | 0.1189 | 0.0001 | | 531 | 4 | 2 | * | 0.0988 | 0.3892 | 0.0625 | 0.2717 | 0.1769 | 0.0009 | | 532 | 4 | 4 |  | 0.0139 | 0.0084 | 0.3955 | 0.5466 | 0.0357 | 0.0000 | | 533 | 4 | 4 |  | 0.0521 | 0.2579 | 0.0540 | 0.5582 | 0.0778 | 0.0001 | | 534 | 4 | 3 | * | 0.1178 | 0.1495 | 0.3618 | 0.3430 | 0.0255 | 0.0025 | | 535 | 4 | 2 | * | 0.2112 | 0.3615 | 0.1732 | 0.2498 | 0.0043 | 0.0000 | | 536 | 4 | 4 |  | 0.0483 | 0.1034 | 0.4122 | 0.4123 | 0.0237 | 0.0003 | | 537 | 4 | 3 | * | 0.0395 | 0.2238 | 0.4130 | 0.3147 | 0.0067 | 0.0024 | | 538 | 4 | 3 | * | 0.1343 | 0.2202 | 0.3855 | 0.2521 | 0.0077 | 0.0003 | | 539 | 4 | 4 |  | 0.0121 | 0.0055 | 0.3980 | 0.5493 | 0.0350 | 0.0001 | | 540 | 4 | 4 |  | 0.0998 | 0.2870 | 0.2214 | 0.3902 | 0.0015 | 0.0000 | | 541 | 4 | 4 |  | 0.0373 | 0.2055 | 0.0572 | 0.6000 | 0.0992 | 0.0008 | | 542 | 4 | 3 | * | 0.0469 | 0.2288 | 0.4516 | 0.2641 | 0.0069 | 0.0018 | | 543 | 4 | 4 |  | 0.0096 | 0.0060 | 0.3851 | 0.5616 | 0.0375 | 0.0003 | | 544 | 4 | 4 |  | 0.1415 | 0.2645 | 0.2288 | 0.3594 | 0.0055 | 0.0003 | | 545 | 4 | 3 | * | 0.0918 | 0.3394 | 0.3821 | 0.1854 | 0.0011 | 0.0002 | | 546 | 4 | 4 |  | 0.0157 | 0.0063 | 0.4394 | 0.4988 | 0.0398 | 0.0001 | | 547 | 4 | 4 |  | 0.0136 | 0.0177 | 0.3739 | 0.5844 | 0.0103 | 0.0001 | | 548 | 4 | 2 | * | 0.0599 | 0.5183 | 0.0506 | 0.3478 | 0.0227 | 0.0005 | | 549 | 4 | 4 |  | 0.0935 | 0.3172 | 0.2145 | 0.3736 | 0.0011 | 0.0001 | | 550 | 4 | 2 | * | 0.0757 | 0.4847 | 0.0697 | 0.2491 | 0.1190 | 0.0019 | | 551 | 4 | 1 | * | 0.3224 | 0.2605 | 0.1061 | 0.1638 | 0.1469 | 0.0003 | | 552 | 4 | 4 |  | 0.1295 | 0.1087 | 0.2958 | 0.3258 | 0.1395 | 0.0007 | | 553 | 4 | 2 | * | 0.2796 | 0.3650 | 0.1089 | 0.1616 | 0.0849 | 0.0000 | | 554 | 4 | 2 | * | 0.0462 | 0.4195 | 0.0546 | 0.4195 | 0.0601 | 0.0001 | | 555 | 4 | 3 | * | 0.1347 | 0.3177 | 0.3523 | 0.1932 | 0.0020 | 0.0000 | | 556 | 4 | 2 | * | 0.1112 | 0.3290 | 0.0818 | 0.2640 | 0.2116 | 0.0024 | | 557 | 4 | 4 |  | 0.0144 | 0.0133 | 0.4462 | 0.5001 | 0.0259 | 0.0000 | | 558 | 4 | 3 | * | 0.1748 | 0.2115 | 0.4076 | 0.1961 | 0.0099 | 0.0001 | | 559 | 4 | 4 |  | 0.0608 | 0.1777 | 0.3534 | 0.3893 | 0.0187 | 0.0001 | | 560 | 4 | 1 | * | 0.3721 | 0.0309 | 0.2167 | 0.2729 | 0.1075 | 0.0000 | | 561 | 4 | 4 |  | 0.1280 | 0.2695 | 0.2180 | 0.3631 | 0.0212 | 0.0002 | | 562 | 4 | 4 |  | 0.1645 | 0.0809 | 0.2475 | 0.3726 | 0.1345 | 0.0000 | | 563 | 4 | 4 |  | 0.1097 | 0.0387 | 0.3119 | 0.4120 | 0.1274 | 0.0003 | | 564 | 4 | 4 |  | 0.1007 | 0.0552 | 0.3322 | 0.4538 | 0.0579 | 0.0003 | | 565 | 4 | 4 |  | 0.0321 | 0.0675 | 0.2234 | 0.6562 | 0.0135 | 0.0073 | | 566 | 4 | 4 |  | 0.1038 | 0.0994 | 0.3044 | 0.4450 | 0.0472 | 0.0001 | | 567 | 4 | 3 | * | 0.0574 | 0.1446 | 0.4003 | 0.3937 | 0.0036 | 0.0004 | | 568 | 4 | 4 |  | 0.0879 | 0.1379 | 0.3662 | 0.3960 | 0.0108 | 0.0011 | | 569 | 4 | 4 |  | 0.0249 | 0.0274 | 0.2527 | 0.6862 | 0.0086 | 0.0002 | | 570 | 4 | 4 |  | 0.0246 | 0.0835 | 0.2553 | 0.6307 | 0.0030 | 0.0029 | | 571 | 4 | 4 |  | 0.1421 | 0.1016 | 0.2765 | 0.4032 | 0.0767 | 0.0000 | | 572 | 4 | 5 | * | 0.2076 | 0.1619 | 0.0201 | 0.0398 | 0.5678 | 0.0026 | | 573 | 4 | 3 | * | 0.1120 | 0.2628 | 0.4791 | 0.1212 | 0.0240 | 0.0009 | | 574 | 4 | 4 |  | 0.1648 | 0.2452 | 0.2189 | 0.3344 | 0.0367 | 0.0001 | | 575 | 4 | 3 | * | 0.0873 | 0.2926 | 0.4924 | 0.1116 | 0.0141 | 0.0020 | | 576 | 4 | 4 |  | 0.0397 | 0.0284 | 0.2596 | 0.6572 | 0.0150 | 0.0001 | | 577 | 4 | 4 |  | 0.1372 | 0.0345 | 0.2776 | 0.3498 | 0.2007 | 0.0002 | | 578 | 4 | 4 |  | 0.0997 | 0.0282 | 0.2181 | 0.5494 | 0.1046 | 0.0001 | | 579 | 4 | 4 |  | 0.2180 | 0.2271 | 0.1822 | 0.3206 | 0.0521 | 0.0000 | | 580 | 4 | 2 | * | 0.1221 | 0.4086 | 0.2073 | 0.1526 | 0.1093 | 0.0000 | | 581 | 4 | 4 |  | 0.0799 | 0.1032 | 0.3296 | 0.4692 | 0.0180 | 0.0001 | | 582 | 4 | 5 | * | 0.1600 | 0.0278 | 0.1902 | 0.2342 | 0.3877 | 0.0002 | | 583 | 4 | 4 |  | 0.1009 | 0.0645 | 0.1831 | 0.5423 | 0.1060 | 0.0032 | | 584 | 4 | 1 | * | 0.3978 | 0.0240 | 0.1699 | 0.2254 | 0.1830 | 0.0000 | | 585 | 4 | 5 | * | 0.1541 | 0.0952 | 0.0118 | 0.0212 | 0.7163 | 0.0014 | | 586 | 4 | 4 |  | 0.0825 | 0.3213 | 0.2498 | 0.3388 | 0.0073 | 0.0003 | | 587 | 4 | 4 |  | 0.1964 | 0.1142 | 0.2763 | 0.3451 | 0.0675 | 0.0005 | | 588 | 4 | 2 | * | 0.0915 | 0.4930 | 0.2014 | 0.1625 | 0.0517 | 0.0000 | | 589 | 4 | 5 | * | 0.1621 | 0.2496 | 0.1402 | 0.1102 | 0.3379 | 0.0000 | | 590 | 4 | 1 | * | 0.3856 | 0.0158 | 0.1356 | 0.2065 | 0.2565 | 0.0000 | | 591 | 4 | 5 | * | 0.0844 | 0.0340 | 0.0041 | 0.0093 | 0.8680 | 0.0001 | | 592 | 4 | 4 |  | 0.1774 | 0.0472 | 0.3629 | 0.3990 | 0.0134 | 0.0001 | | 593 | 4 | 2 | * | 0.2230 | 0.4173 | 0.0510 | 0.0886 | 0.2111 | 0.0089 | | 594 | 4 | 3 | * | 0.1476 | 0.2640 | 0.4388 | 0.0982 | 0.0507 | 0.0008 | | 595 | 4 | 4 |  | 0.0469 | 0.0247 | 0.2567 | 0.6474 | 0.0242 | 0.0001 | | 596 | 4 | 4 |  | 0.0590 | 0.3398 | 0.2539 | 0.3446 | 0.0022 | 0.0006 | | 597 | 4 | 4 |  | 0.3013 | 0.0365 | 0.2780 | 0.3265 | 0.0576 | 0.0000 | | 598 | 4 | 4 |  | 0.0242 | 0.0732 | 0.2561 | 0.6358 | 0.0050 | 0.0057 | | 599 | 4 | 3 | * | 0.0781 | 0.3738 | 0.4421 | 0.0950 | 0.0057 | 0.0053 | | 600 | 4 | 2 | * | 0.0864 | 0.4046 | 0.2522 | 0.2034 | 0.0534 | 0.0001 | | 601 | 4 | 3 | * | 0.0625 | 0.0692 | 0.4313 | 0.4154 | 0.0203 | 0.0014 | | 602 | 4 | 4 |  | 0.0427 | 0.0599 | 0.2118 | 0.6584 | 0.0230 | 0.0041 | | 603 | 4 | 4 |  | 0.0576 | 0.1127 | 0.3440 | 0.4722 | 0.0134 | 0.0001 | | 604 | 4 | 3 | * | 0.2121 | 0.1884 | 0.3420 | 0.0906 | 0.1668 | 0.0002 | | 605 | 4 | 5 | * | 0.1813 | 0.2600 | 0.0351 | 0.0749 | 0.4264 | 0.0222 | | 606 | 4 | 4 |  | 0.1151 | 0.1300 | 0.3633 | 0.3720 | 0.0191 | 0.0005 | | 607 | 4 | 4 |  | 0.0141 | 0.0256 | 0.2680 | 0.6902 | 0.0015 | 0.0006 | | 608 | 4 | 2 | * | 0.1537 | 0.3511 | 0.1954 | 0.1261 | 0.1737 | 0.0000 | | 609 | 4 | 4 |  | 0.0587 | 0.1277 | 0.3742 | 0.4314 | 0.0060 | 0.0021 | | 610 | 5 | 5 |  | 0.2147 | 0.0074 | 0.0056 | 0.0106 | 0.7616 | 0.0002 | | 611 | 5 | 5 |  | 0.3432 | 0.0335 | 0.0417 | 0.2176 | 0.3567 | 0.0072 | | 612 | 5 | 5 |  | 0.0692 | 0.0270 | 0.1125 | 0.1354 | 0.6559 | 0.0000 | | 613 | 5 | 5 |  | 0.0224 | 0.1671 | 0.0095 | 0.0446 | 0.7561 | 0.0003 | | 614 | 5 | 5 |  | 0.0464 | 0.0078 | 0.0369 | 0.0434 | 0.8655 | 0.0000 | | 615 | 5 | 5 |  | 0.3938 | 0.0286 | 0.0468 | 0.0128 | 0.5176 | 0.0004 | | 616 | 5 | 5 |  | 0.0176 | 0.0779 | 0.0045 | 0.0189 | 0.8810 | 0.0000 | | 617 | 5 | 5 |  | 0.0740 | 0.0258 | 0.1182 | 0.3293 | 0.4284 | 0.0242 | | 618 | 5 | 5 |  | 0.0150 | 0.0576 | 0.0030 | 0.0114 | 0.9130 | 0.0000 | | 619 | 5 | 5 |  | 0.1402 | 0.1081 | 0.0250 | 0.0280 | 0.6986 | 0.0000 | | 620 | 5 | 4 | * | 0.0620 | 0.0631 | 0.2639 | 0.3612 | 0.2498 | 0.0000 | | 621 | 5 | 5 |  | 0.1083 | 0.0015 | 0.0012 | 0.0023 | 0.8866 | 0.0000 | | 622 | 5 | 5 |  | 0.3026 | 0.0148 | 0.0113 | 0.0195 | 0.6513 | 0.0004 | | 623 | 5 | 1 | * | 0.3524 | 0.0479 | 0.0526 | 0.2626 | 0.2668 | 0.0178 | | 624 | 5 | 1 | * | 0.4096 | 0.0756 | 0.1119 | 0.0339 | 0.3588 | 0.0101 | | 625 | 5 | 5 |  | 0.0263 | 0.3969 | 0.0265 | 0.1475 | 0.4018 | 0.0010 | | 626 | 5 | 5 |  | 0.1678 | 0.0041 | 0.0036 | 0.0070 | 0.8174 | 0.0001 | | 627 | 5 | 5 |  | 0.1179 | 0.0675 | 0.0158 | 0.0199 | 0.7789 | 0.0000 | | 628 | 5 | 5 |  | 0.0683 | 0.0193 | 0.0728 | 0.0822 | 0.7574 | 0.0000 | | 629 | 5 | 1 | * | 0.3668 | 0.1465 | 0.1841 | 0.0480 | 0.1314 | 0.1232 | | 630 | 5 | 5 |  | 0.1105 | 0.0329 | 0.0094 | 0.0113 | 0.8358 | 0.0000 | | 631 | 5 | 5 |  | 0.1718 | 0.1821 | 0.0327 | 0.0313 | 0.5821 | 0.0000 | | 632 | 5 | 5 |  | 0.0764 | 0.0183 | 0.0940 | 0.2580 | 0.5460 | 0.0073 | | 633 | 5 | 6 | * | 0.0428 | 0.0301 | 0.1106 | 0.3436 | 0.0894 | 0.3836 | | 634 | 5 | 5 |  | 0.1026 | 0.0014 | 0.0010 | 0.0017 | 0.8933 | 0.0000 | | 635 | 5 | 5 |  | 0.1187 | 0.0666 | 0.0163 | 0.0200 | 0.7784 | 0.0000 | | 636 | 5 | 4 | * | 0.0682 | 0.0340 | 0.1446 | 0.3781 | 0.3077 | 0.0674 | | 637 | 5 | 1 | * | 0.4966 | 0.0394 | 0.0276 | 0.0444 | 0.3862 | 0.0058 | | 638 | 5 | 5 |  | 0.0251 | 0.2608 | 0.0148 | 0.0664 | 0.6321 | 0.0007 | | 639 | 5 | 5 |  | 0.3628 | 0.0511 | 0.0694 | 0.0222 | 0.4905 | 0.0040 | | 640 | 5 | 5 |  | 0.3690 | 0.0265 | 0.0495 | 0.0167 | 0.5379 | 0.0004 | | 641 | 5 | 6 | * | 0.0428 | 0.0301 | 0.1106 | 0.3436 | 0.0894 | 0.3836 | | 642 | 5 | 5 |  | 0.0650 | 0.0428 | 0.1889 | 0.2805 | 0.4227 | 0.0000 | | 643 | 5 | 5 |  | 0.2072 | 0.3342 | 0.0549 | 0.0485 | 0.3546 | 0.0006 | | 644 | 5 | 5 |  | 0.0602 | 0.0098 | 0.0576 | 0.2156 | 0.6526 | 0.0041 | | 645 | 5 | 5 |  | 0.2809 | 0.0176 | 0.0239 | 0.1185 | 0.5583 | 0.0008 | | 646 | 5 | 1 | * | 0.4113 | 0.1043 | 0.1416 | 0.0431 | 0.2689 | 0.0308 | | 647 | 5 | 5 |  | 0.0695 | 0.0261 | 0.1197 | 0.1351 | 0.6496 | 0.0000 | | 648 | 5 | 5 |  | 0.0224 | 0.1671 | 0.0095 | 0.0446 | 0.7561 | 0.0003 | | 649 | 5 | 5 |  | 0.3323 | 0.0486 | 0.0672 | 0.0213 | 0.5242 | 0.0064 | | 650 | 5 | 5 |  | 0.0176 | 0.0779 | 0.0045 | 0.0189 | 0.8810 | 0.0000 | | 651 | 5 | 4 | * | 0.2430 | 0.0696 | 0.0724 | 0.4279 | 0.0919 | 0.0952 | | 652 | 5 | 5 |  | 0.0736 | 0.0267 | 0.1111 | 0.3299 | 0.4324 | 0.0263 | | 653 | 5 | 5 |  | 0.0150 | 0.0576 | 0.0030 | 0.0114 | 0.9130 | 0.0000 | | 654 | 5 | 4 | * | 0.3021 | 0.0572 | 0.0674 | 0.3443 | 0.1862 | 0.0429 | | 655 | 5 | 5 |  | 0.2613 | 0.0680 | 0.0483 | 0.0202 | 0.6019 | 0.0002 | | 656 | 5 | 1 | * | 0.5305 | 0.1595 | 0.2079 | 0.0785 | 0.0229 | 0.0007 | | 657 | 5 | 5 |  | 0.0305 | 0.0191 | 0.1121 | 0.1483 | 0.6900 | 0.0001 | | 658 | 5 | 1 | * | 0.4628 | 0.1611 | 0.0227 | 0.0474 | 0.3023 | 0.0037 | | 659 | 5 | 2 | * | 0.1083 | 0.3750 | 0.0611 | 0.1466 | 0.0091 | 0.2999 | | 660 | 5 | 5 |  | 0.0691 | 0.0668 | 0.0141 | 0.0006 | 0.8482 | 0.0012 | | 661 | 5 | 5 |  | 0.0747 | 0.0292 | 0.0552 | 0.0118 | 0.8292 | 0.0000 | | 662 | 5 | 4 | * | 0.0162 | 0.0352 | 0.1670 | 0.7791 | 0.0020 | 0.0005 | | 663 | 5 | 5 |  | 0.0122 | 0.0111 | 0.0073 | 0.0018 | 0.9630 | 0.0046 | | 664 | 5 | 1 | * | 0.6766 | 0.0863 | 0.1255 | 0.0523 | 0.0592 | 0.0001 | | 665 | 5 | 5 |  | 0.0156 | 0.0050 | 0.0016 | 0.0001 | 0.9776 | 0.0000 | | 666 | 5 | 5 |  | 0.1204 | 0.0111 | 0.0645 | 0.0155 | 0.7886 | 0.0000 | | 667 | 5 | 5 |  | 0.1333 | 0.0171 | 0.1049 | 0.0259 | 0.7188 | 0.0000 | | 668 | 5 | 5 |  | 0.0577 | 0.0384 | 0.0088 | 0.0004 | 0.8944 | 0.0003 | | 669 | 5 | 2 | * | 0.3536 | 0.3565 | 0.0585 | 0.1327 | 0.0816 | 0.0171 | | 670 | 5 | 3 | * | 0.1678 | 0.0587 | 0.3497 | 0.0949 | 0.3268 | 0.0021 | | 671 | 5 | 5 |  | 0.3877 | 0.0224 | 0.0699 | 0.1272 | 0.3928 | 0.0000 | | 672 | 5 | 5 |  | 0.0140 | 0.0188 | 0.0119 | 0.0030 | 0.9370 | 0.0153 | | 673 | 5 | 5 |  | 0.1130 | 0.3001 | 0.0777 | 0.0038 | 0.4867 | 0.0187 | | 674 | 5 | 5 |  | 0.0383 | 0.0108 | 0.0228 | 0.0049 | 0.9232 | 0.0000 | | 675 | 5 | 4 | * | 0.0244 | 0.0251 | 0.1464 | 0.7974 | 0.0066 | 0.0001 | | 676 | 5 | 4 | * | 0.2810 | 0.0645 | 0.2033 | 0.3769 | 0.0736 | 0.0007 | | 677 | 5 | 1 | * | 0.3880 | 0.3108 | 0.0616 | 0.1227 | 0.0905 | 0.0264 | | 678 | 5 | 2 | * | 0.2923 | 0.4000 | 0.0657 | 0.1564 | 0.0519 | 0.0337 | | 679 | 5 | 4 | * | 0.0179 | 0.0242 | 0.1469 | 0.8070 | 0.0038 | 0.0001 | | 680 | 5 | 1 | * | 0.7063 | 0.0659 | 0.0892 | 0.0385 | 0.1001 | 0.0000 | | 681 | 5 | 4 | * | 0.0339 | 0.0527 | 0.2952 | 0.3558 | 0.2600 | 0.0024 | | 682 | 5 | 1 | * | 0.7216 | 0.0464 | 0.0596 | 0.0250 | 0.1474 | 0.0000 | | 683 | 5 | 5 |  | 0.1504 | 0.0191 | 0.0139 | 0.0057 | 0.8109 | 0.0000 | | 684 | 5 | 5 |  | 0.0213 | 0.0684 | 0.0647 | 0.0212 | 0.5967 | 0.2276 | | 685 | 5 | 5 |  | 0.0151 | 0.0034 | 0.0218 | 0.0292 | 0.9305 | 0.0000 | | 686 | 5 | 5 |  | 0.2120 | 0.0388 | 0.0262 | 0.0099 | 0.7131 | 0.0001 | | 687 | 5 | 5 |  | 0.0231 | 0.0036 | 0.0064 | 0.0014 | 0.9655 | 0.0000 | | 688 | 5 | 5 |  | 0.0211 | 0.0090 | 0.0525 | 0.0698 | 0.8475 | 0.0001 | | 689 | 5 | 5 |  | 0.0055 | 0.0019 | 0.0013 | 0.0003 | 0.9909 | 0.0001 | | 690 | 5 | 1 | * | 0.7238 | 0.0209 | 0.0262 | 0.0099 | 0.2192 | 0.0000 | | 691 | 5 | 5 |  | 0.0472 | 0.0013 | 0.0068 | 0.0015 | 0.9432 | 0.0000 | | 692 | 5 | 5 |  | 0.2924 | 0.1898 | 0.1267 | 0.0509 | 0.3381 | 0.0022 | | 693 | 5 | 5 |  | 0.0776 | 0.0054 | 0.0324 | 0.0081 | 0.8766 | 0.0000 | | 694 | 5 | 5 |  | 0.0092 | 0.0047 | 0.0030 | 0.0006 | 0.9818 | 0.0007 | | 695 | 5 | 5 |  | 0.2216 | 0.0067 | 0.0190 | 0.0342 | 0.7185 | 0.0000 | | 696 | 5 | 5 |  | 0.2740 | 0.0980 | 0.0673 | 0.0299 | 0.5303 | 0.0005 | | 697 | 5 | 4 | * | 0.0520 | 0.0204 | 0.1244 | 0.7598 | 0.0433 | 0.0000 | | 698 | 5 | 5 |  | 0.0871 | 0.0493 | 0.0840 | 0.0169 | 0.7626 | 0.0001 | | 699 | 5 | 1 | * | 0.3894 | 0.0335 | 0.0911 | 0.1670 | 0.3190 | 0.0000 | | 700 | 5 | 5 |  | 0.1402 | 0.1407 | 0.2527 | 0.0446 | 0.4213 | 0.0006 | | 701 | 5 | 5 |  | 0.0331 | 0.0287 | 0.1684 | 0.2130 | 0.5566 | 0.0002 | | 702 | 5 | 4 | * | 0.0249 | 0.0178 | 0.1310 | 0.8158 | 0.0106 | 0.0000 | | 703 | 5 | 5 |  | 0.3241 | 0.0110 | 0.0313 | 0.0496 | 0.5840 | 0.0000 | | 704 | 5 | 5 |  | 0.0191 | 0.0066 | 0.0034 | 0.0006 | 0.9702 | 0.0000 | | 705 | 5 | 2 | * | 0.2074 | 0.3983 | 0.0725 | 0.1866 | 0.0320 | 0.1032 | | 706 | 5 | 1 | * | 0.3593 | 0.3320 | 0.0839 | 0.0155 | 0.2039 | 0.0054 | | 707 | 5 | 1 | * | 0.4950 | 0.1223 | 0.0369 | 0.0171 | 0.3286 | 0.0002 | | 708 | 5 | 2 | * | 0.2860 | 0.4722 | 0.0222 | 0.0637 | 0.1559 | 0.0000 | | 709 | 5 | 2 | * | 0.1855 | 0.6426 | 0.0319 | 0.0903 | 0.0497 | 0.0000 | | 710 | 5 | 5 |  | 0.0702 | 0.0351 | 0.2004 | 0.0831 | 0.6111 | 0.0000 | | 711 | 5 | 5 |  | 0.4263 | 0.0794 | 0.0248 | 0.0127 | 0.4567 | 0.0002 | | 712 | 5 | 2 | * | 0.2443 | 0.4740 | 0.1543 | 0.0786 | 0.0484 | 0.0004 | | 713 | 5 | 5 |  | 0.0283 | 0.0016 | 0.0035 | 0.0019 | 0.9637 | 0.0009 | | 714 | 5 | 1 | * | 0.4798 | 0.1915 | 0.0722 | 0.0356 | 0.2208 | 0.0001 | | 715 | 5 | 2 | * | 0.1738 | 0.2969 | 0.0554 | 0.1906 | 0.2831 | 0.0002 | | 716 | 5 | 5 |  | 0.0415 | 0.0024 | 0.0093 | 0.0052 | 0.9416 | 0.0000 | | 717 | 5 | 5 |  | 0.2476 | 0.1990 | 0.0430 | 0.1144 | 0.3953 | 0.0007 | | 718 | 5 | 5 |  | 0.0597 | 0.0221 | 0.1238 | 0.0492 | 0.7451 | 0.0000 | | 719 | 5 | 3 | * | 0.1320 | 0.2452 | 0.4260 | 0.1775 | 0.0192 | 0.0001 | | 720 | 5 | 5 |  | 0.0324 | 0.0012 | 0.0045 | 0.0024 | 0.9595 | 0.0000 | | 721 | 5 | 5 |  | 0.2730 | 0.1403 | 0.0254 | 0.0580 | 0.5028 | 0.0006 | | 722 | 5 | 1 | * | 0.4098 | 0.2533 | 0.0900 | 0.0542 | 0.1921 | 0.0006 | | 723 | 5 | 5 |  | 0.1700 | 0.2028 | 0.0348 | 0.1173 | 0.4747 | 0.0003 | | 724 | 5 | 5 |  | 0.0587 | 0.0046 | 0.0183 | 0.0095 | 0.9088 | 0.0000 | | 725 | 5 | 3 | * | 0.1758 | 0.2208 | 0.4014 | 0.1737 | 0.0282 | 0.0000 | | 726 | 5 | 5 |  | 0.0397 | 0.0032 | 0.0072 | 0.0039 | 0.9448 | 0.0011 | | 727 | 5 | 2 | * | 0.3122 | 0.4131 | 0.1278 | 0.0678 | 0.0785 | 0.0004 | | 728 | 5 | 2 | * | 0.2513 | 0.5646 | 0.0284 | 0.0770 | 0.0788 | 0.0000 | | 729 | 5 | 5 |  | 0.3410 | 0.1617 | 0.0457 | 0.0085 | 0.4360 | 0.0071 | | 730 | 5 | 2 | * | 0.2772 | 0.5089 | 0.0891 | 0.0115 | 0.1120 | 0.0013 | | 731 | 5 | 5 |  | 0.3145 | 0.2575 | 0.0147 | 0.0493 | 0.3640 | 0.0000 | | 732 | 5 | 5 |  | 0.0726 | 0.0084 | 0.0433 | 0.0260 | 0.8496 | 0.0000 | | 733 | 5 | 5 |  | 0.3471 | 0.0324 | 0.0101 | 0.0052 | 0.6051 | 0.0001 | | 734 | 5 | 1 | * | 0.3905 | 0.3641 | 0.0941 | 0.0452 | 0.1059 | 0.0003 | | 735 | 5 | 3 | * | 0.2402 | 0.2165 | 0.3387 | 0.1494 | 0.0551 | 0.0001 | | 736 | 5 | 5 |  | 0.0181 | 0.0005 | 0.0013 | 0.0007 | 0.9790 | 0.0004 | | 737 | 5 | 2 | * | 0.2305 | 0.2818 | 0.0659 | 0.1570 | 0.2642 | 0.0007 | | 738 | 5 | 5 |  | 0.0321 | 0.0009 | 0.0042 | 0.0024 | 0.9604 | 0.0000 | | 739 | 5 | 2 | * | 0.1391 | 0.3560 | 0.0832 | 0.2713 | 0.1502 | 0.0001 | | 740 | 5 | 5 |  | 0.0437 | 0.0100 | 0.0734 | 0.0307 | 0.8421 | 0.0000 | | 741 | 5 | 2 | * | 0.1775 | 0.4910 | 0.1952 | 0.1095 | 0.0264 | 0.0003 | | 742 | 5 | 3 | * | 0.0783 | 0.2021 | 0.4750 | 0.2339 | 0.0106 | 0.0001 | | 743 | 5 | 5 |  | 0.1979 | 0.0478 | 0.0107 | 0.0247 | 0.7186 | 0.0004 | | 744 | 5 | 5 |  | 0.0495 | 0.0404 | 0.2685 | 0.1217 | 0.5198 | 0.0001 | | 745 | 5 | 5 |  | 0.1278 | 0.0593 | 0.0105 | 0.0378 | 0.7645 | 0.0001 | | 746 | 5 | 5 |  | 0.0515 | 0.0131 | 0.0662 | 0.0245 | 0.8446 | 0.0000 | | 747 | 5 | 3 | * | 0.2610 | 0.1610 | 0.3354 | 0.1545 | 0.0880 | 0.0001 | | 748 | 5 | 5 |  | 0.0503 | 0.0060 | 0.0158 | 0.0091 | 0.9172 | 0.0016 | | 749 | 5 | 2 | * | 0.2458 | 0.4813 | 0.1575 | 0.0310 | 0.0800 | 0.0046 | | 750 | 5 | 1 | * | 0.3723 | 0.2515 | 0.0547 | 0.0096 | 0.3061 | 0.0058 | | 751 | 5 | 2 | * | 0.3121 | 0.3760 | 0.0156 | 0.0445 | 0.2518 | 0.0000 | | 752 | 5 | 5 |  | 0.2842 | 0.1010 | 0.0165 | 0.0352 | 0.5628 | 0.0004 | | 753 | 5 | 5 |  | 0.3841 | 0.0506 | 0.0134 | 0.0075 | 0.5443 | 0.0001 | | 754 | 5 | 5 |  | 0.1598 | 0.1345 | 0.0203 | 0.0648 | 0.6203 | 0.0004 | | 755 | 5 | 5 |  | 0.0238 | 0.0010 | 0.0020 | 0.0010 | 0.9716 | 0.0005 | | 756 | 6 | 2 | * | 0.1438 | 0.5420 | 0.0566 | 0.1014 | 0.1073 | 0.0489 | | 757 | 6 | 6 |  | 0.0149 | 0.0332 | 0.0740 | 0.0344 | 0.3510 | 0.4926 | | 758 | 6 | 6 |  | 0.1043 | 0.0976 | 0.0996 | 0.0136 | 0.1551 | 0.5298 | | 759 | 6 | 5 | * | 0.0758 | 0.0325 | 0.0112 | 0.0135 | 0.8665 | 0.0005 | | 760 | 6 | 6 |  | 0.0086 | 0.0395 | 0.2114 | 0.2906 | 0.0029 | 0.4470 | | 761 | 6 | 2 | * | 0.3013 | 0.6275 | 0.0168 | 0.0352 | 0.0177 | 0.0013 | | 762 | 6 | 6 |  | 0.1012 | 0.0079 | 0.0688 | 0.0748 | 0.2496 | 0.4976 | | 763 | 6 | 3 | * | 0.0423 | 0.1555 | 0.3641 | 0.2751 | 0.0244 | 0.1386 | | 764 | 6 | 6 |  | 0.0266 | 0.0326 | 0.0408 | 0.0451 | 0.0762 | 0.7787 | | 765 | 6 | 2 | * | 0.3575 | 0.5513 | 0.0157 | 0.0402 | 0.0346 | 0.0008 | | 766 | 6 | 5 | * | 0.1907 | 0.2832 | 0.0256 | 0.0415 | 0.4394 | 0.0196 | | 767 | 6 | 2 | * | 0.3597 | 0.5123 | 0.0114 | 0.0283 | 0.0807 | 0.0076 | | 768 | 6 | 6 |  | 0.0017 | 0.0075 | 0.0023 | 0.0202 | 0.0199 | 0.9484 | | 769 | 6 | 6 |  | 0.0074 | 0.0312 | 0.1557 | 0.1882 | 0.0026 | 0.6150 | | 770 | 6 | 6 |  | 0.0224 | 0.0305 | 0.0371 | 0.0532 | 0.0756 | 0.7812 | | 771 | 6 | 6 |  | 0.0020 | 0.0071 | 0.0090 | 0.0120 | 0.0019 | 0.9680 | | 772 | 6 | 2 | * | 0.2203 | 0.7045 | 0.0202 | 0.0474 | 0.0068 | 0.0007 | | 773 | 6 | 6 |  | 0.0933 | 0.0081 | 0.0721 | 0.0992 | 0.2607 | 0.4666 | | 774 | 6 | 3 | * | 0.0530 | 0.1652 | 0.3737 | 0.2362 | 0.0246 | 0.1472 | | 775 | 6 | 5 | * | 0.0626 | 0.0308 | 0.0112 | 0.0168 | 0.8781 | 0.0004 | | 776 | 6 | 6 |  | 0.0287 | 0.0537 | 0.0773 | 0.0909 | 0.0405 | 0.7088 | | 777 | 6 | 6 |  | 0.0008 | 0.0058 | 0.0023 | 0.0188 | 0.0042 | 0.9680 | | 778 | 6 | 6 |  | 0.0131 | 0.0331 | 0.0777 | 0.0421 | 0.3730 | 0.4610 | | 779 | 6 | 6 |  | 0.1126 | 0.0879 | 0.0935 | 0.0110 | 0.1348 | 0.5603 | | 780 | 6 | 2 | * | 0.0854 | 0.6591 | 0.0603 | 0.0884 | 0.0185 | 0.0882 | | 781 | 6 | 5 | * | 0.1226 | 0.0042 | 0.0345 | 0.0405 | 0.7654 | 0.0328 | | 782 | 6 | 6 |  | 0.0153 | 0.0358 | 0.0378 | 0.0052 | 0.0059 | 0.9001 | | 783 | 6 | 6 |  | 0.0018 | 0.0072 | 0.0023 | 0.0174 | 0.0180 | 0.9533 | | 784 | 6 | 6 |  | 0.0006 | 0.0067 | 0.0401 | 0.0747 | 0.0002 | 0.8777 | | 785 | 6 | 5 | * | 0.0681 | 0.0576 | 0.0390 | 0.1101 | 0.7221 | 0.0031 | | 786 | 6 | 3 | * | 0.0125 | 0.1191 | 0.3258 | 0.2582 | 0.0022 | 0.2821 | | 787 | 6 | 6 |  | 0.0071 | 0.0240 | 0.0557 | 0.0306 | 0.1523 | 0.7304 | | 788 | 6 | 6 |  | 0.0379 | 0.0243 | 0.0305 | 0.0402 | 0.2935 | 0.5736 | | 789 | 6 | 3 | * | 0.0262 | 0.1378 | 0.3570 | 0.2792 | 0.0076 | 0.1923 | | 790 | 6 | 6 |  | 0.0001 | 0.0025 | 0.0009 | 0.0072 | 0.0005 | 0.9889 | | 791 | 6 | 5 | * | 0.0239 | 0.0060 | 0.0022 | 0.0032 | 0.9645 | 0.0001 | | 792 | 6 | 2 | * | 0.1602 | 0.5439 | 0.0556 | 0.0850 | 0.1009 | 0.0544 | | 793 | 6 | 6 |  | 0.0872 | 0.0092 | 0.0793 | 0.1264 | 0.1412 | 0.5567 | | 794 | 6 | 6 |  | 0.0253 | 0.0058 | 0.0517 | 0.0718 | 0.0291 | 0.8163 | | 795 | 6 | 6 |  | 0.0068 | 0.0129 | 0.0035 | 0.0289 | 0.2970 | 0.6509 | | 796 | 6 | 5 | * | 0.0928 | 0.1796 | 0.0630 | 0.1060 | 0.4202 | 0.1383 | | 797 | 6 | 6 |  | 0.0068 | 0.0278 | 0.0493 | 0.0233 | 0.1115 | 0.7813 | | 798 | 6 | 5 | * | 0.1183 | 0.0446 | 0.0448 | 0.0057 | 0.6606 | 0.1260 | | 799 | 6 | 4 | * | 0.0302 | 0.0566 | 0.2592 | 0.3182 | 0.0375 | 0.2981 | | 800 | 6 | 2 | * | 0.1044 | 0.6317 | 0.0469 | 0.0930 | 0.0522 | 0.0719 | | 801 | 6 | 6 |  | 0.0962 | 0.1215 | 0.1146 | 0.0148 | 0.0772 | 0.5757 | | 802 | 6 | 6 |  | 0.0030 | 0.0189 | 0.1122 | 0.1773 | 0.0010 | 0.6876 | | 803 | 6 | 3 | * | 0.1066 | 0.1492 | 0.3441 | 0.2269 | 0.1449 | 0.0282 | | 804 | 6 | 1 | * | 0.4799 | 0.3601 | 0.0089 | 0.0203 | 0.1301 | 0.0007 | | 805 | 6 | 5 | * | 0.0099 | 0.0079 | 0.0148 | 0.0069 | 0.9503 | 0.0101 | | 806 | 6 | 6 |  | 0.0001 | 0.0001 | 0.0000 | 0.0000 | 0.0002 | 0.9996 | | 807 | 6 | 6 |  | 0.0000 | 0.0000 | 0.0001 | 0.0001 | 0.0003 | 0.9993 | | 808 | 6 | 6 |  | 0.0000 | 0.0000 | 0.0000 | 0.0000 | 0.0000 | 1.0000 | | 809 | 6 | 6 |  | 0.0000 | 0.0002 | 0.0000 | 0.0002 | 0.0001 | 0.9994 | | 810 | 6 | 6 |  | 0.0000 | 0.0000 | 0.0000 | 0.0000 | 0.0000 | 1.0000 | | 811 | 6 | 6 |  | 0.0000 | 0.0000 | 0.0000 | 0.0000 | 0.0000 | 1.0000 | | 812 | 6 | 6 |  | 0.0000 | 0.0000 | 0.0000 | 0.0000 | 0.0001 | 0.9998 | | 813 | 6 | 6 |  | 0.0000 | 0.0000 | 0.0000 | 0.0000 | 0.0000 | 1.0000 | | 814 | 6 | 6 |  | 0.0001 | 0.0037 | 0.0010 | 0.0010 | 0.0007 | 0.9935 | | 815 | 6 | 6 |  | 0.0003 | 0.0004 | 0.0000 | 0.0003 | 0.0046 | 0.9943 | | 816 | 6 | 6 |  | 0.0000 | 0.0000 | 0.0000 | 0.0000 | 0.0000 | 0.9999 | | 817 | 6 | 6 |  | 0.0000 | 0.0000 | 0.0001 | 0.0001 | 0.0000 | 0.9998 | | 818 | 6 | 6 |  | 0.0000 | 0.0001 | 0.0000 | 0.0000 | 0.0000 | 0.9999 | | 819 | 6 | 6 |  | 0.0000 | 0.0000 | 0.0000 | 0.0000 | 0.0000 | 0.9999 | | 820 | 6 | 6 |  | 0.0000 | 0.0000 | 0.0000 | 0.0000 | 0.0000 | 1.0000 | | 821 | 6 | 6 |  | 0.0001 | 0.0001 | 0.0000 | 0.0000 | 0.0002 | 0.9996 | | 822 | 6 | 6 |  | 0.0000 | 0.0003 | 0.0000 | 0.0002 | 0.0000 | 0.9995 | | 823 | 6 | 6 |  | 0.0005 | 0.0088 | 0.0025 | 0.0025 | 0.0096 | 0.9760 | | 824 | 6 | 6 |  | 0.0000 | 0.0002 | 0.0000 | 0.0002 | 0.0001 | 0.9995 | | 825 | 6 | 6 |  | 0.0000 | 0.0000 | 0.0000 | 0.0000 | 0.0000 | 1.0000 | | 826 | 6 | 6 |  | 0.0000 | 0.0000 | 0.0000 | 0.0000 | 0.0000 | 0.9999 | | 827 | 6 | 6 |  | 0.0000 | 0.0000 | 0.0000 | 0.0000 | 0.0000 | 1.0000 | | 828 | 6 | 6 |  | 0.0001 | 0.0031 | 0.0011 | 0.0010 | 0.0005 | 0.9943 | | 829 | 6 | 6 |  | 0.0000 | 0.0011 | 0.0003 | 0.0002 | 0.0000 | 0.9983 | | 830 | 6 | 6 |  | 0.0000 | 0.0000 | 0.0000 | 0.0000 | 0.0000 | 1.0000 | | 831 | 6 | 6 |  | 0.0000 | 0.0000 | 0.0001 | 0.0001 | 0.0000 | 0.9998 | | 832 | 6 | 6 |  | 0.0001 | 0.0049 | 0.0014 | 0.0013 | 0.0006 | 0.9917 | | 833 | 6 | 6 |  | 0.0000 | 0.0000 | 0.0000 | 0.0000 | 0.0001 | 0.9999 | | 834 | 6 | 6 |  | 0.0000 | 0.0003 | 0.0000 | 0.0002 | 0.0001 | 0.9994 | | 835 | 6 | 6 |  | 0.0006 | 0.0002 | 0.0000 | 0.0000 | 0.0073 | 0.9919 | | 836 | 6 | 6 |  | 0.0002 | 0.0004 | 0.0000 | 0.0000 | 0.0004 | 0.9989 | | 837 | 6 | 6 |  | 0.0000 | 0.0000 | 0.0000 | 0.0000 | 0.0000 | 1.0000 | | 838 | 6 | 6 |  | 0.0001 | 0.0032 | 0.0011 | 0.0010 | 0.0009 | 0.9937 | | 839 | 6 | 6 |  | 0.0000 | 0.0000 | 0.0000 | 0.0000 | 0.0000 | 1.0000 | | 840 | 6 | 6 |  | 0.0000 | 0.0002 | 0.0000 | 0.0001 | 0.0001 | 0.9996 | | 841 | 6 | 6 |  | 0.0000 | 0.0000 | 0.0000 | 0.0000 | 0.0000 | 1.0000 | | 842 | 6 | 6 |  | 0.0000 | 0.0000 | 0.0000 | 0.0000 | 0.0000 | 1.0000 | | 843 | 6 | 6 |  | 0.0000 | 0.0000 | 0.0000 | 0.0000 | 0.0000 | 0.9999 | | 844 | 6 | 6 |  | 0.0000 | 0.0000 | 0.0001 | 0.0001 | 0.0001 | 0.9998 | | 845 | 6 | 6 |  | 0.0000 | 0.0000 | 0.0000 | 0.0000 | 0.0000 | 1.0000 | | 846 | 6 | 6 |  | 0.0000 | 0.0014 | 0.0003 | 0.0004 | 0.0001 | 0.9979 | | 847 | 6 | 6 |  | 0.0000 | 0.0000 | 0.0000 | 0.0000 | 0.0000 | 0.9999 | | 848 | 6 | 6 |  | 0.0000 | 0.0000 | 0.0000 | 0.0000 | 0.0000 | 1.0000 | | 849 | 6 | 6 |  | 0.0011 | 0.0189 | 0.0047 | 0.0052 | 0.0131 | 0.9570 | | 850 | 6 | 6 |  | 0.0000 | 0.0000 | 0.0000 | 0.0000 | 0.0000 | 1.0000 | | 851 | 6 | 6 |  | 0.0002 | 0.0002 | 0.0000 | 0.0002 | 0.0000 | 0.9994 | | 852 | 6 | 6 |  | 0.0004 | 0.0002 | 0.0000 | 0.0000 | 0.0002 | 0.9991 | | 853 | 6 | 6 |  | 0.0000 | 0.0000 | 0.0000 | 0.0000 | 0.0000 | 1.0000 | | 854 | 6 | 6 |  | 0.0004 | 0.0004 | 0.0001 | 0.0003 | 0.0000 | 0.9988 | | 855 | 6 | 6 |  | 0.0004 | 0.0001 | 0.0000 | 0.0000 | 0.0005 | 0.9990 | | 856 | 6 | 6 |  | 0.0003 | 0.0003 | 0.0001 | 0.0002 | 0.0000 | 0.9992 | | 857 | 6 | 6 |  | 0.0000 | 0.0000 | 0.0000 | 0.0000 | 0.0000 | 1.0000 | | 858 | 6 | 6 |  | 0.0000 | 0.0001 | 0.0000 | 0.0000 | 0.0000 | 0.9999 | | 859 | 6 | 6 |  | 0.0000 | 0.0000 | 0.0000 | 0.0000 | 0.0000 | 1.0000 | | 860 | 6 | 6 |  | 0.0003 | 0.0002 | 0.0000 | 0.0000 | 0.0002 | 0.9994 | | 861 | 6 | 6 |  | 0.0000 | 0.0000 | 0.0000 | 0.0000 | 0.0000 | 1.0000 | | 862 | 6 | 6 |  | 0.0000 | 0.0000 | 0.0000 | 0.0000 | 0.0000 | 1.0000 | | 863 | 6 | 6 |  | 0.0000 | 0.0000 | 0.0000 | 0.0000 | 0.0000 | 1.0000 | | 864 | 6 | 6 |  | 0.0002 | 0.0001 | 0.0000 | 0.0001 | 0.0000 | 0.9996 | | 865 | 6 | 6 |  | 0.0002 | 0.0001 | 0.0000 | 0.0000 | 0.0002 | 0.9995 | | 866 | 6 | 6 |  | 0.0001 | 0.0001 | 0.0000 | 0.0001 | 0.0000 | 0.9998 | | 867 | 6 | 6 |  | 0.0000 | 0.0000 | 0.0000 | 0.0000 | 0.0000 | 1.0000 | | 868 | 6 | 6 |  | 0.0003 | 0.0004 | 0.0001 | 0.0002 | 0.0000 | 0.9989 | | 869 | 6 | 6 |  | 0.0000 | 0.0001 | 0.0000 | 0.0000 | 0.0000 | 0.9998 | | 870 | 6 | 6 |  | 0.0003 | 0.0003 | 0.0000 | 0.0000 | 0.0000 | 0.9994 | | 871 | 6 | 6 |  | 0.0000 | 0.0000 | 0.0000 | 0.0000 | 0.0000 | 1.0000 | | 872 | 6 | 6 |  | 0.0002 | 0.0003 | 0.0000 | 0.0001 | 0.0000 | 0.9994 | | 873 | 6 | 6 |  | 0.0007 | 0.0011 | 0.0002 | 0.0007 | 0.0000 | 0.9974 | | 874 | 6 | 6 |  | 0.0000 | 0.0000 | 0.0000 | 0.0000 | 0.0000 | 1.0000 | | 875 | 6 | 6 |  | 0.0000 | 0.0005 | 0.0003 | 0.0007 | 0.0000 | 0.9984 | | 876 | 6 | 6 |  | 0.0000 | 0.0000 | 0.0000 | 0.0000 | 0.0000 | 0.9999 | | 877 | 6 | 6 |  | 0.0000 | 0.0001 | 0.0001 | 0.0006 | 0.0000 | 0.9993 | | | --- | --- | --- | --- | --- | --- | --- | --- | --- | --- | --- | --- | --- | --- | --- | --- | --- | --- | --- | --- | --- | --- | --- | --- | --- | --- | --- | --- | --- | --- | --- | --- | --- | --- | --- | --- | --- | --- | --- | --- | --- | --- | --- | --- | --- | --- | --- | --- | --- | --- | --- | --- | --- | --- | --- | --- | --- | --- | --- | --- | --- | --- | --- | --- | --- | --- | --- | --- | --- | --- | --- | --- | --- | --- | --- | --- | --- | --- | --- | --- | --- | --- | --- | --- | --- | --- | --- | --- | --- | --- | --- | --- | --- | --- | --- | --- | --- | --- | --- | --- | --- | --- | --- | --- | --- | --- | --- | --- | --- | --- | --- | --- | --- | --- | --- | --- | --- | --- | --- | --- | --- | --- | --- | --- | --- | --- | --- | --- | --- | --- | --- | --- | --- | --- | --- | --- | --- | --- | --- | --- | --- | --- | --- | --- | --- | --- | --- | --- | --- | --- | --- | --- | --- | --- | --- | --- | --- | --- | --- | --- | --- | --- | --- | --- | --- | --- | --- | --- | --- | --- | --- | --- | --- | --- | --- | --- | --- | --- | --- | --- | --- | --- | --- | --- | --- | --- | --- | --- | --- | --- | --- | --- | --- | --- | --- | --- | --- | --- | --- | --- | --- | --- | --- | --- | --- | --- | --- | --- | --- | --- | --- | --- | --- | --- | --- | --- | --- | --- | --- | --- | --- | --- | --- | --- | --- | --- | --- | --- | --- | --- | --- | --- | --- | --- | --- | --- | --- | --- | --- | --- | --- | --- | --- | --- | --- | --- | --- | --- | --- | --- | --- | --- | --- | --- | --- | --- | --- | --- | --- | --- | --- | --- | --- | --- | --- | --- | --- | --- | --- | --- | --- | --- | --- | --- | --- | --- | --- | --- | --- | --- | --- | --- | --- | --- | --- | --- | --- | --- | --- | --- | --- | --- | --- | --- | --- | --- | --- | --- | --- | --- | --- | --- | --- | --- | --- | --- | --- | --- | --- | --- | --- | --- | --- | --- | --- | --- | --- | --- | --- | --- | --- | --- | --- | --- | --- | --- | --- | --- | --- | --- | --- | --- | --- | --- | --- | --- | --- | --- | --- | --- | --- | --- | --- | --- | --- | --- | --- | --- | --- | --- | --- | --- | --- | --- | --- | --- | --- | --- | --- | --- | --- | --- | --- | --- | --- | --- | --- | --- | --- | --- | --- | --- | --- | --- | --- | --- | --- | --- | --- | --- | --- | --- | --- | --- | --- | --- | --- | --- | --- | --- | --- | --- | --- | --- | --- | --- | --- | --- | --- | --- | --- | --- | --- | --- | --- | --- | --- | --- | --- | --- | --- | --- | --- | --- | --- | --- | --- | --- | --- | --- | --- | --- | --- | --- | --- | --- | --- | --- | --- | --- | --- | --- | --- | --- | --- | --- | --- | --- | --- | --- | --- | --- | --- | --- | --- | --- | --- | --- | --- | --- | --- | --- | --- | --- | --- | --- | --- | --- | --- | --- | --- | --- | --- | --- | --- | --- | --- | --- | --- | --- | --- | --- | --- | --- | --- | --- | --- | --- | --- | --- | --- | --- | --- | --- | --- | --- | --- | --- | --- | --- | --- | --- | --- | --- | --- | --- | --- | --- | --- | --- | --- | --- | --- | --- | --- | --- | --- | --- | --- | --- | --- | --- | --- | --- | --- | --- | --- | --- | --- | --- | --- | --- | --- | --- | --- | --- | --- | --- | --- | --- | --- | --- | --- | --- | --- | --- | --- | --- | --- | --- | --- | --- | --- | --- | --- | --- | --- | --- | --- | --- | --- | --- | --- | --- | --- | --- | --- | --- | --- | --- | --- | --- | --- | --- | --- | --- | --- | --- | --- | --- | --- | --- | --- | --- | --- | --- | --- | --- | --- | --- | --- | --- | --- | --- | --- | --- | --- | --- | --- | --- | --- | --- | --- | --- | --- | --- | --- | --- | --- | --- | --- | --- | --- | --- | --- | --- | --- | --- | --- | --- | --- | --- | --- | --- | --- | --- | --- | --- | --- | --- | --- | --- | --- | --- | --- | --- | --- | --- | --- | --- | --- | --- | --- | --- | --- | --- | --- | --- | --- | --- | --- | --- | --- | --- | --- | --- | --- | --- | --- | --- | --- | --- | --- | --- | --- | --- | --- | --- | --- | --- | --- | --- | --- | --- | --- | --- | --- | --- | --- | --- | --- | --- | --- | --- | --- | --- | --- | --- | --- | --- | --- | --- | --- | --- | --- | --- | --- | --- | --- | --- | --- | --- | --- | --- | --- | --- | --- | --- | --- | --- | --- | --- | --- | --- | --- | --- | --- | --- | --- | --- | --- | --- | --- | --- | --- | --- | --- | --- | --- | --- | --- | --- | --- | --- | --- | --- | --- | --- | --- | --- | --- | --- | --- | --- | --- | --- | --- | --- | --- | --- | --- | --- | --- | --- | --- | --- | --- | --- | --- | --- | --- | --- | --- | --- | --- | --- | --- | --- | --- | --- | --- | --- | --- | --- | --- | --- | --- | --- | --- | --- | --- | --- | --- | --- | --- | --- | --- | --- | --- | --- | --- | --- | --- | --- | --- | --- | --- | --- | --- | --- | --- | --- | --- | --- | --- | --- | --- | --- | --- | --- | --- | --- | --- | --- | --- | --- | --- | --- | --- | --- | --- | --- | --- | --- | --- | --- | --- | --- | --- | --- | --- | --- | --- | --- | --- | --- | --- | --- | --- | --- | --- | --- | --- | --- | --- | --- | --- | --- | --- | --- | --- | --- | --- | --- | --- | --- | --- | --- | --- | --- | --- | --- | --- | --- | --- | --- | --- | --- | --- | --- | --- | --- | --- | --- | --- | --- | --- | --- | --- | --- | --- | --- | --- | --- | --- | --- | --- | --- | --- | --- | --- | --- | --- | --- | --- | --- | --- | --- | --- | --- | --- | --- | --- | --- | --- | --- | --- | --- | --- | --- | --- | --- | --- | --- | --- | --- | --- | --- | --- | --- | --- | --- | --- | --- | --- | --- | --- | --- | --- | --- | --- | --- | --- | --- | --- | --- | --- | --- | --- | --- | --- | --- | --- | --- | --- | --- | --- | --- | --- | --- | --- | --- | --- | --- | --- | --- | --- | --- | --- | --- | --- | --- | --- | --- | --- | --- | --- | --- | --- | --- | --- | --- | --- | --- | --- | --- | --- | --- | --- | --- | --- | --- | --- | --- | --- | --- | --- | --- | --- | --- | --- | --- | --- | --- | --- | --- | --- | --- | --- | --- | --- | --- | --- | --- | --- | --- | --- | --- | --- | --- | --- | --- | --- | --- | --- | --- | --- | --- | --- | --- | --- | --- | --- | --- | --- | --- | --- | --- | --- | --- | --- | --- | --- | --- | --- | --- | --- | --- | --- | --- | --- | --- | --- | --- | --- | --- | --- | --- | --- | --- | --- | --- | --- | --- | --- | --- | --- | --- | --- | --- | --- | --- | --- | --- | --- | --- | --- | --- | --- | --- | --- | --- | --- | --- | --- | --- | --- | --- | --- | --- | --- | --- | --- | --- | --- | --- | --- | --- | --- | --- | --- | --- | --- | --- | --- | --- | --- | --- | --- | --- | --- | --- | --- | --- | --- | --- | --- | --- | --- | --- | --- | --- | --- | --- | --- | --- | --- | --- | --- | --- | --- | --- | --- | --- | --- | --- | --- | --- | --- | --- | --- | --- | --- | --- | --- | --- | --- | --- | --- | --- | --- | --- | --- | --- | --- | --- | --- | --- | --- | --- | --- | --- | --- | --- | --- | --- | --- | --- | --- | --- | --- | --- | --- | --- | --- | --- | --- | --- | --- | --- | --- | --- | --- | --- | --- | --- | --- | --- | --- | --- | --- | --- | --- | --- | --- | --- | --- | --- | --- | --- | --- | --- | --- | --- | --- | --- | --- | --- | --- | --- | --- | --- | --- | --- | --- | --- | --- | --- | --- | --- | --- | --- | --- | --- | --- | --- | --- | --- | --- | --- | --- | --- | --- | --- | --- | --- | --- | --- | --- | --- | --- | --- | --- | --- | --- | --- | --- | --- | --- | --- | --- | --- | --- | --- | --- | --- | --- | --- | --- | --- | --- | --- | --- | --- | --- | --- | --- | --- | --- | --- | --- | --- | --- | --- | --- | --- | --- | --- | --- | --- | --- | --- | --- | --- | --- | --- | --- | --- | --- | --- | --- | --- | --- | --- | --- | --- | --- | --- | --- | --- | --- | --- | --- | --- | --- | --- | --- | --- | --- | --- | --- | --- | --- | --- | --- | --- | --- | --- | --- | --- | --- | --- | --- | --- | --- | --- | --- | --- | --- | --- | --- | --- | --- | --- | --- | --- | --- | --- | --- | --- | --- | --- | --- | --- | --- | --- | --- | --- | --- | --- | --- | --- | --- | --- | --- | --- | --- | --- | --- | --- | --- | --- | --- | --- | --- | --- | --- | --- | --- | --- | --- | --- | --- | --- | --- | --- | --- | --- | --- | --- | --- | --- | --- | --- | --- | --- | --- | --- | --- | --- | --- | --- | --- | --- | --- | --- | --- | --- | --- | --- | --- | --- | --- | --- | --- | --- | --- | --- | --- | --- | --- | --- | --- | --- | --- | --- | --- | --- | --- | --- | --- | --- | --- | --- | --- | --- | --- | --- | --- | --- | --- | --- | --- | --- | --- | --- | --- | --- | --- | --- | --- | --- | --- | --- | --- | --- | --- | --- | --- | --- | --- | --- | --- | --- | --- | --- | --- | --- | --- | --- | --- | --- | --- | --- | --- | --- | --- | --- | --- | --- | --- | --- | --- | --- | --- | --- | --- | --- | --- | --- | --- | --- | --- | --- | --- | --- | --- | --- | --- | --- | --- | --- | --- | --- | --- | --- | --- | --- | --- | --- | --- | --- | --- | --- | --- | --- | --- | --- | --- | --- | --- | --- | --- | --- | --- | --- | --- | --- | --- | --- | --- | --- | --- | --- | --- | --- | --- | --- | --- | --- | --- | --- | --- | --- | --- | --- | --- | --- | --- | --- | --- | --- | --- | --- | --- | --- | --- | --- | --- | --- | --- | --- | --- | --- | --- | --- | --- | --- | --- | --- | --- | --- | --- | --- | --- | --- | --- | --- | --- | --- | --- | --- | --- | --- | --- | --- | --- | --- | --- | --- | --- | --- | --- | --- | --- | --- | --- | --- | --- | --- | --- | --- | --- | --- | --- | --- | --- | --- | --- | --- | --- | --- | --- | --- | --- | --- | --- | --- | --- | --- | --- | --- | --- | --- | --- | --- | --- | --- | --- | --- | --- | --- | --- | --- | --- | --- | --- | --- | --- | --- | --- | --- | --- | --- | --- | --- | --- | --- | --- | --- | --- | --- | --- | --- | --- | --- | --- | --- | --- | --- | --- | --- | --- | --- | --- | --- | --- | --- | --- | --- | --- | --- | --- | --- | --- | --- | --- | --- | --- | --- | --- | --- | --- | --- | --- | --- | --- | --- | --- | --- | --- | --- | --- | --- | --- | --- | --- | --- | --- | --- | --- | --- | --- | --- | --- | --- | --- | --- | --- | --- | --- | --- | --- | --- | --- | --- | --- | --- | --- | --- | --- | --- | --- | --- | --- | --- | --- | --- | --- | --- | --- | --- | --- | --- | --- | --- | --- | --- | --- | --- | --- | --- | --- | --- | --- | --- | --- | --- | --- | --- | --- | --- | --- | --- | --- | --- | --- | --- | --- | --- | --- | --- | --- | --- | --- | --- | --- | --- | --- | --- | --- | --- | --- | --- | --- | --- | --- | --- | --- | --- | --- | --- | --- | --- | --- | --- | --- | --- | --- | --- | --- | --- | --- | --- | --- | --- | --- | --- | --- | --- | --- | --- | --- | --- | --- | --- | --- | --- | --- | --- | --- | --- | --- | --- | --- | --- | --- | --- | --- | --- | --- | --- | --- | --- | --- | --- | --- | --- | --- | --- | --- | --- | --- | --- | --- | --- | --- | --- | --- | --- | --- | --- | --- | --- | --- | --- | --- | --- | --- | --- | --- | --- | --- | --- | --- | --- | --- | --- | --- | --- | --- | --- | --- | --- | --- | --- | --- | --- | --- | --- | --- | --- | --- | --- | --- | --- | --- | --- | --- | --- | --- | --- | --- | --- | --- | --- | --- | --- | --- | --- | --- | --- | --- | --- | --- | --- | --- | --- | --- | --- | --- | --- | --- | --- | --- | --- | --- | --- | --- | --- | --- | --- | --- | --- | --- | --- | --- | --- | --- | --- | --- | --- | --- | --- | --- | --- | --- | --- | --- | --- | --- | --- | --- | --- | --- | --- | --- | --- | --- | --- | --- | --- | --- | --- | --- | --- | --- | --- | --- | --- | --- | --- | --- | --- | --- | --- | --- | --- | --- | --- | --- | --- | --- | --- | --- | --- | --- | --- | --- | --- | --- | --- | --- | --- | --- | --- | --- | --- | --- | --- | --- | --- | --- | --- | --- | --- | --- | --- | --- | --- | --- | --- | --- | --- | --- | --- | --- | --- | --- | --- | --- | --- | --- | --- | --- | --- | --- | --- | --- | --- | --- | --- | --- | --- | --- | --- | --- | --- | --- | --- | --- | --- | --- | --- | --- | --- | --- | --- | --- | --- | --- | --- | --- | --- | --- | --- | --- | --- | --- | --- | --- | --- | --- | --- | --- | --- | --- | --- | --- | --- | --- | --- | --- | --- | --- | --- | --- | --- | --- | --- | --- | --- | --- | --- | --- | --- | --- | --- | --- | --- | --- | --- | --- | --- | --- | --- | --- | --- | --- | --- | --- | --- | --- | --- | --- | --- | --- | --- | --- | --- | --- | --- | --- | --- | --- | --- | --- | --- | --- | --- | --- | --- | --- | --- | --- | --- | --- | --- | --- | --- | --- | --- | --- | --- | --- | --- | --- | --- | --- | --- | --- | --- | --- | --- | --- | --- | --- | --- | --- | --- | --- | --- | --- | --- | --- | --- | --- | --- | --- | --- | --- | --- | --- | --- | --- | --- | --- | --- | --- | --- | --- | --- | --- | --- | --- | --- | --- | --- | --- | --- | --- | --- | --- | --- | --- | --- | --- | --- | --- | --- | --- | --- | --- | --- | --- | --- | --- | --- | --- | --- | --- | --- | --- | --- | --- | --- | --- | --- | --- | --- | --- | --- | --- | --- | --- | --- | --- | --- | --- | --- | --- | --- | --- | --- | --- | --- | --- | --- | --- | --- | --- | --- | --- | --- | --- | --- | --- | --- | --- | --- | --- | --- | --- | --- | --- | --- | --- | --- | --- | --- | --- | --- | --- | --- | --- | --- | --- | --- | --- | --- | --- | --- | --- | --- | --- | --- | --- | --- | --- | --- | --- | --- | --- | --- | --- | --- | --- | --- | --- | --- | --- | --- | --- | --- | --- | --- | --- | --- | --- | --- | --- | --- | --- | --- | --- | --- | --- | --- | --- | --- | --- | --- | --- | --- | --- | --- | --- | --- | --- | --- | --- | --- | --- | --- | --- | --- | --- | --- | --- | --- | --- | --- | --- | --- | --- | --- | --- | --- | --- | --- | --- | --- | --- | --- | --- | --- | --- | --- | --- | --- | --- | --- | --- | --- | --- | --- | --- | --- | --- | --- | --- | --- | --- | --- | --- | --- | --- | --- | --- | --- | --- | --- | --- | --- | --- | --- | --- | --- | --- | --- | --- | --- | --- | --- | --- | --- | --- | --- | --- | --- | --- | --- | --- | --- | --- | --- | --- | --- | --- | --- | --- | --- | --- | --- | --- | --- | --- | --- | --- | --- | --- | --- | --- | --- | --- | --- | --- | --- | --- | --- | --- | --- | --- | --- | --- | --- | --- | --- | --- | --- | --- | --- | --- | --- | --- | --- | --- | --- | --- | --- | --- | --- | --- | --- | --- | --- | --- | --- | --- | --- | --- | --- | --- | --- | --- | --- | --- | --- | --- | --- | --- | --- | --- | --- | --- | --- | --- | --- | --- | --- | --- | --- | --- | --- | --- | --- | --- | --- | --- | --- | --- | --- | --- | --- | --- | --- | --- | --- | --- | --- | --- | --- | --- | --- | --- | --- | --- | --- | --- | --- | --- | --- | --- | --- | --- | --- | --- | --- | --- | --- | --- | --- | --- | --- | --- | --- | --- | --- | --- | --- | --- | --- | --- | --- | --- | --- | --- | --- | --- | --- | --- | --- | --- | --- | --- | --- | --- | --- | --- | --- | --- | --- | --- | --- | --- | --- | --- | --- | --- | --- | --- | --- | --- | --- | --- | --- | --- | --- | --- | --- | --- | --- | --- | --- | --- | --- | --- | --- | --- | --- | --- | --- | --- | --- | --- | --- | --- | --- | --- | --- | --- | --- | --- | --- | --- | --- | --- | --- | --- | --- | --- | --- | --- | --- | --- | --- | --- | --- | --- | --- | --- | --- | --- | --- | --- | --- | --- | --- | --- | --- | --- | --- | --- | --- | --- | --- | --- | --- | --- | --- | --- | --- | --- | --- | --- | --- | --- | --- | --- | --- | --- | --- | --- | --- | --- | --- | --- | --- | --- | --- | --- | --- | --- | --- | --- | --- | --- | --- | --- | --- | --- | --- | --- | --- | --- | --- | --- | --- | --- | --- | --- | --- | --- | --- | --- | --- | --- | --- | --- | --- | --- | --- | --- | --- | --- | --- | --- | --- | --- | --- | --- | --- | --- | --- | --- | --- | --- | --- | --- | --- | --- | --- | --- | --- | --- | --- | --- | --- | --- | --- | --- | --- | --- | --- | --- | --- | --- | --- | --- | --- | --- | --- | --- | --- | --- | --- | --- | --- | --- | --- | --- | --- | --- | --- | --- | --- | --- | --- | --- | --- | --- | --- | --- | --- | --- | --- | --- | --- | --- | --- | --- | --- | --- | --- | --- | --- | --- | --- | --- | --- | --- | --- | --- | --- | --- | --- | --- | --- | --- | --- | --- | --- | --- | --- | --- | --- | --- | --- | --- | --- | --- | --- | --- | --- | --- | --- | --- | --- | --- | --- | --- | --- | --- | --- | --- | --- | --- | --- | --- | --- | --- | --- | --- | --- | --- | --- | --- | --- | --- | --- | --- | --- | --- | --- | --- | --- | --- | --- | --- | --- | --- | --- | --- | --- | --- | --- | --- | --- | --- | --- | --- | --- | --- | --- | --- | --- | --- | --- | --- | --- | --- | --- | --- | --- | --- | --- | --- | --- | --- | --- | --- | --- | --- | --- | --- | --- | --- | --- | --- | --- | --- | --- | --- | --- | --- | --- | --- | --- | --- | --- | --- | --- | --- | --- | --- | --- | --- | --- | --- | --- | --- | --- | --- | --- | --- | --- | --- | --- | --- | --- | --- | --- | --- | --- | --- | --- | --- | --- | --- | --- | --- | --- | --- | --- | --- | --- | --- | --- | --- | --- | --- | --- | --- | --- | --- | --- | --- | --- | --- | --- | --- | --- | --- | --- | --- | --- | --- | --- | --- | --- | --- | --- | --- | --- | --- | --- | --- | --- | --- | --- | --- | --- | --- | --- | --- | --- | --- | --- | --- | --- | --- | --- | --- | --- | --- | --- | --- | --- | --- | --- | --- | --- | --- | --- | --- | --- | --- | --- | --- | --- | --- | --- | --- | --- | --- | --- | --- | --- | --- | --- | --- | --- | --- | --- | --- | --- | --- | --- | --- | --- | --- | --- | --- | --- | --- | --- | --- | --- | --- | --- | --- | --- | --- | --- | --- | --- | --- | --- | --- | --- | --- | --- | --- | --- | --- | --- | --- | --- | --- | --- | --- | --- | --- | --- | --- | --- | --- | --- | --- | --- | --- | --- | --- | --- | --- | --- | --- | --- | --- | --- | --- | --- | --- | --- | --- | --- | --- | --- | --- | --- | --- | --- | --- | --- | --- | --- | --- | --- | --- | --- | --- | --- | --- | --- | --- | --- | --- | --- | --- | --- | --- | --- | --- | --- | --- | --- | --- | --- | --- | --- | --- | --- | --- | --- | --- | --- | --- | --- | --- | --- | --- | --- | --- | --- | --- | --- | --- | --- | --- | --- | --- | --- | --- | --- | --- | --- | --- | --- | --- | --- | --- | --- | --- | --- | --- | --- | --- | --- | --- | --- | --- | --- | --- | --- | --- | --- | --- | --- | --- | --- | --- | --- | --- | --- | --- | --- | --- | --- | --- | --- | --- | --- | --- | --- | --- | --- | --- | --- | --- | --- | --- | --- | --- | --- | --- | --- | --- | --- | --- | --- | --- | --- | --- | --- | --- | --- | --- | --- | --- | --- | --- | --- | --- | --- | --- | --- | --- | --- | --- | --- | --- | --- | --- | --- | --- | --- | --- | --- | --- | --- | --- | --- | --- | --- | --- | --- | --- | --- | --- | --- | --- | --- | --- | --- | --- | --- | --- | --- | --- | --- | --- | --- | --- | --- | --- | --- | --- | --- | --- | --- | --- | --- | --- | --- | --- | --- | --- | --- | --- | --- | --- | --- | --- | --- | --- | --- | --- | --- | --- | --- | --- | --- | --- | --- | --- | --- | --- | --- | --- | --- | --- | --- | --- | --- | --- | --- | --- | --- | --- | --- | --- | --- | --- | --- | --- | --- | --- | --- | --- | --- | --- | --- | --- | --- | --- | --- | --- | --- | --- | --- | --- | --- | --- | --- | --- | --- | --- | --- | --- | --- | --- | --- | --- | --- | --- | --- | --- | --- | --- | --- | --- | --- | --- | --- | --- | --- | --- | --- | --- | --- | --- | --- | --- | --- | --- | --- | --- | --- | --- | --- | --- | --- | --- | --- | --- | --- | --- | --- | --- | --- | --- | --- | --- | --- | --- | --- | --- | --- | --- | --- | --- | --- | --- | --- | --- | --- | --- | --- | --- | --- | --- | --- | --- | --- | --- | --- | --- | --- | --- | --- | --- | --- | --- | --- | --- | --- | --- | --- | --- | --- | --- | --- | --- | --- | --- | --- | --- | --- | --- | --- | --- | --- | --- | --- | --- | --- | --- | --- | --- | --- | --- | --- | --- | --- | --- | --- | --- | --- | --- | --- | --- | --- | --- | --- | --- | --- | --- | --- | --- | --- | --- | --- | --- | --- | --- | --- | --- | --- | --- | --- | --- | --- | --- | --- | --- | --- | --- | --- | --- | --- | --- | --- | --- | --- | --- | --- | --- | --- | --- | --- | --- | --- | --- | --- | --- | --- | --- | --- | --- | --- | --- | --- | --- | --- | --- | --- | --- | --- | --- | --- | --- | --- | --- | --- | --- | --- | --- | --- | --- | --- | --- | --- | --- | --- | --- | --- | --- | --- | --- | --- | --- | --- | --- | --- | --- | --- | --- | --- | --- | --- | --- | --- | --- | --- | --- | --- | --- | --- | --- | --- | --- | --- | --- | --- | --- | --- | --- | --- | --- | --- | --- | --- | --- | --- | --- | --- | --- | --- | --- | --- | --- | --- | --- | --- | --- | --- | --- | --- | --- | --- | --- | --- | --- | --- | --- | --- | --- | --- | --- | --- | --- | --- | --- | --- | --- | --- | --- | --- | --- | --- | --- | --- | --- | --- | --- | --- | --- | --- | --- | --- | --- | --- | --- | --- | --- | --- | --- | --- | --- | --- | --- | --- | --- | --- | --- | --- | --- | --- | --- | --- | --- | --- | --- | --- | --- | --- | --- | --- | --- | --- | --- | --- | --- | --- | --- | --- | --- | --- | --- | --- | --- | --- | --- | --- | --- | --- | --- | --- | --- | --- | --- | --- | --- | --- | --- | --- | --- | --- | --- | --- | --- | --- | --- | --- | --- | --- | --- | --- | --- | --- | --- | --- | --- | --- | --- | --- | --- | --- | --- | --- | --- | --- | --- | --- | --- | --- | --- | --- | --- | --- | --- | --- | --- | --- | --- | --- | --- | --- | --- | --- | --- | --- | --- | --- | --- | --- | --- | --- | --- | --- | --- | --- | --- | --- | --- | --- | --- | --- | --- | --- | --- | --- | --- | --- | --- | --- | --- | --- | --- | --- | --- | --- | --- | --- | --- | --- | --- | --- | --- | --- | --- | --- | --- | --- | --- | --- | --- | --- | --- | --- | --- | --- | --- | --- | --- | --- | --- | --- | --- | --- | --- | --- | --- | --- | --- | --- | --- | --- | --- | --- | --- | --- | --- | --- | --- | --- | --- | --- | --- | --- | --- | --- | --- | --- | --- | --- | --- | --- | --- | --- | --- | --- | --- | --- | --- | --- | --- | --- | --- | --- | --- | --- | --- | --- | --- | --- | --- | --- | --- | --- | --- | --- | --- | --- | --- | --- | --- | --- | --- | --- | --- | --- | --- | --- | --- | --- | --- | --- | --- | --- | --- | --- | --- | --- | --- | --- | --- | --- | --- | --- | --- | --- | --- | --- | --- | --- | --- | --- | --- | --- | --- | --- | --- | --- | --- | --- | --- | --- | --- | --- | --- | --- | --- | --- | --- | --- | --- | --- | --- | --- | --- | --- | --- | --- | --- | --- | --- | --- | --- | --- | --- | --- | --- | --- | --- | --- | --- | --- | --- | --- | --- | --- | --- | --- | --- | --- | --- | --- | --- | --- | --- | --- | --- | --- | --- | --- | --- | --- | --- | --- | --- | --- | --- | --- | --- | --- | --- | --- | --- | --- | --- | --- | --- | --- | --- | --- | --- | --- | --- | --- | --- | --- | --- | --- | --- | --- | --- | --- | --- | --- | --- | --- | --- | --- | --- | --- | --- | --- | --- | --- | --- | --- | --- | --- | --- | --- | --- | --- | --- | --- | --- | --- | --- | --- | --- | --- | --- | --- | --- | --- | --- | --- | --- | --- | --- | --- | --- | --- | --- | --- | --- | --- | --- | --- | --- | --- | --- | --- | --- | --- | --- | --- | --- | --- | --- | --- | --- | --- | --- | --- | --- | --- | --- | --- | --- | --- | --- | --- | --- | --- | --- | --- | --- | --- | --- | --- | --- | --- | --- | --- | --- | --- | --- | --- | --- | --- | --- | --- | --- | --- | --- | --- | --- | --- | --- | --- | --- | --- | --- | --- | --- | --- | --- | --- | --- | --- | --- | --- | --- | --- | --- | --- | --- | --- | --- | --- | --- | --- | --- | --- | --- | --- | --- | --- | --- | --- | --- | --- | --- | --- | --- | --- | --- | --- | --- | --- | --- | --- | --- | --- | --- | --- | --- | --- | --- | --- | --- | --- | --- | --- | --- | --- | --- | --- | --- | --- | --- | --- | --- | --- | --- | --- | --- | --- | --- | --- | --- | --- | --- | --- | --- | --- | --- | --- | --- | --- | --- | --- | --- | --- | --- | --- | --- | --- | --- | --- | --- | --- | --- | --- | --- | --- | --- | --- | --- | --- | --- | --- | --- | --- | --- | --- | --- | --- | --- | --- | --- | --- | --- | --- | --- | --- | --- | --- | --- | --- | --- | --- | --- | --- | --- | --- | --- | --- | --- | --- | --- | --- | --- | --- | --- | --- | --- | --- | --- | --- | --- | --- | --- | --- | --- | --- | --- | --- | --- | --- | --- | --- | --- | --- | --- | --- | --- | --- | --- | --- | --- | --- | --- | --- | --- | --- | --- | --- | --- | --- | --- | --- | --- | --- | --- | --- | --- | --- | --- | --- | --- | --- | --- | --- | --- | --- | --- | --- | --- | --- | --- | --- | --- | --- | --- | --- | --- | --- | --- | --- | --- | --- | --- | --- | --- | --- | --- | --- | --- | --- | --- | --- | --- | --- | --- | --- | --- | --- | --- | --- | --- | --- | --- | --- | --- | --- | --- | --- | --- | --- | --- | --- | --- | --- | --- | --- | --- | --- | --- | --- | --- | --- | --- | --- | --- | --- | --- | --- | --- | --- | --- | --- | --- | --- | --- | --- | --- | --- | --- | --- | --- | --- | --- | --- | --- | --- | --- | --- | --- | --- | --- | --- | --- | --- | --- | --- | --- | --- | --- | --- | --- | --- | --- | --- | --- | --- | --- | --- | --- | --- | --- | --- | --- | --- | --- | --- | --- | --- | --- | --- | --- | --- | --- | --- | --- | --- | --- | --- | --- | --- | --- | --- | --- | --- | --- | --- | --- | --- | --- | --- | --- | --- | --- | --- | --- | --- | --- | --- | --- | --- | --- | --- | --- | --- | --- | --- | --- | --- | --- | --- | --- | --- | --- | --- | --- | --- | --- | --- | --- | --- | --- | --- | --- | --- | --- | --- | --- | --- | --- | --- | --- | --- | --- | --- | --- | --- | --- | --- | --- | --- | --- | --- | --- | --- | --- | --- | --- | --- | --- | --- | --- | --- | --- | --- | --- | --- | --- | --- | --- | --- | --- | --- | --- | --- | --- | --- | --- | --- | --- | --- | --- | --- | --- | --- | --- | --- | --- | --- | --- | --- | --- | --- | --- | --- | --- | --- | --- | --- | --- | --- | --- | --- | --- | --- | --- | --- | --- | --- | --- | --- | --- | --- | --- | --- | --- | --- | --- | --- | --- | --- | --- | --- | --- | --- | --- | --- | --- | --- | --- | --- | --- | --- | --- | --- | --- | --- | --- | --- | --- | --- | --- | --- | --- | --- | --- | --- | --- | --- | --- | --- | --- | --- | --- | --- | --- | --- | --- | --- | --- | --- | --- | --- | --- | --- | --- | --- | --- | --- | --- | --- | --- | --- | --- | --- | --- | --- | --- | --- | --- | --- | --- | --- | --- | --- | --- | --- | --- | --- | --- | --- | --- | --- | --- | --- | --- | --- | --- | --- | --- | --- | --- | --- | --- | --- | --- | --- | --- | --- | --- | --- | --- | --- | --- | --- | --- | --- | --- | --- | --- | --- | --- | --- | --- | --- | --- | --- | --- | --- | --- | --- | --- | --- | --- | --- | --- | --- | --- | --- | --- | --- | --- | --- | --- | --- | --- | --- | --- | --- | --- | --- | --- | --- | --- | --- | --- | --- | --- | --- | --- | --- | --- | --- | --- | --- | --- | --- | --- | --- | --- | --- | --- | --- | --- | --- | --- | --- | --- | --- | --- | --- | --- | --- | --- | --- | --- | --- | --- | --- | --- | --- | --- | --- | --- | --- | --- | --- | --- | --- | --- | --- | --- | --- | --- | --- | --- | --- | --- | --- | --- | --- | --- | --- | --- | --- | --- | --- | --- | --- | --- | --- | --- | --- | --- | --- | --- | --- | --- | --- | --- | --- | --- | --- | --- | --- | --- | --- | --- | --- | --- | --- | --- | --- | --- | --- | --- | --- | --- | --- | --- | --- | --- | --- | --- | --- | --- | --- | --- | --- | --- | --- | --- | --- | --- | --- | --- | --- | --- | --- | --- | --- | --- | --- | --- | --- | --- | --- | --- | --- | --- | --- | --- | --- | --- | --- | --- | --- | --- | --- | --- | --- | --- | --- | --- | --- | --- | --- | --- | --- | --- | --- | --- | --- | --- | --- | --- | --- | --- | --- | --- | --- | --- | --- | --- | --- | --- | --- | --- | --- | --- | --- | --- | --- | --- | --- | --- | --- | --- | --- | --- | --- | --- | --- | --- | --- | --- | --- | --- | --- | --- | --- | --- | --- | --- | --- | --- | --- | --- | --- | --- | --- | --- | --- | --- | --- | --- | --- | --- | --- | --- | --- | --- | --- | --- | --- | --- | --- | --- | --- | --- | --- | --- | --- | --- | --- | --- | --- | --- | --- | --- | --- | --- | --- | --- | --- | --- | --- | --- | --- | --- | --- | --- | --- | --- | --- | --- | --- | --- | --- | --- | --- | --- | --- | --- | --- | --- | --- | --- | --- | --- | --- | --- | --- | --- | --- | --- | --- | --- | --- | --- | --- | --- | --- | --- | --- | --- | --- | --- | --- | --- | --- | --- | --- | --- | --- | --- | --- | --- | --- | --- | --- | --- | --- | --- | --- | --- | --- | --- | --- | --- | --- | --- | --- | --- | --- | --- | --- | --- | --- | --- | --- | --- | --- | --- | --- | --- | --- | --- | --- | --- | --- | --- | --- | --- | --- | --- | --- | --- | --- | --- | --- | --- | --- | --- | --- | --- | --- | --- | --- | --- | --- | --- | --- | --- | --- | --- | --- | --- | --- | --- | --- | --- | --- | --- | --- | --- | --- | --- | --- | --- | --- | --- | --- | --- | --- | --- | --- | --- | --- | --- | --- | --- | --- | --- | --- | --- | --- | --- | --- | --- | --- | --- | --- | --- | --- | --- | --- | --- | --- | --- | --- | --- | --- | --- | --- | --- | --- | --- | --- | --- | --- | --- | --- | --- | --- | --- | --- | --- | --- | --- | --- | --- | --- | --- | --- | --- | --- | --- | --- | --- | --- | --- | --- | --- | --- | --- | --- | --- | --- | --- | --- | --- | --- | --- | --- | --- | --- | --- | --- | --- | --- | --- | --- | --- | --- | --- | --- | --- | --- | --- | --- | --- | --- | --- | --- | --- | --- | --- | --- | --- | --- | --- | --- | --- | --- | --- | --- | --- | --- | --- | --- | --- | --- | --- | --- | --- | --- | --- | --- | --- | --- | --- | --- | --- | --- | --- | --- | --- | --- | --- | --- | --- | --- | --- | --- | --- | --- | --- | --- | --- | --- | --- | --- | --- | --- | --- | --- | --- | --- | --- | --- | --- | --- | --- | --- | --- | --- | --- | --- | --- | --- | --- | --- | --- | --- | --- | --- | --- | --- | --- | --- | --- | --- | --- | --- | --- | --- | --- | --- | --- | --- | --- | --- | --- | --- | --- | --- | --- | --- | --- | --- | --- | --- | --- | --- | --- | --- | --- | --- | --- | --- | --- | --- | --- | --- | --- | --- | --- | --- | --- | --- | --- | --- | --- | --- | --- | --- | --- | --- | --- | --- | --- | --- | --- | --- | --- | --- | --- | --- | --- | --- | --- | --- | --- | --- | --- | --- | --- | --- | --- | --- | --- | --- | --- | --- | --- | --- | --- | --- | --- | --- | --- | --- | --- | --- | --- | --- | --- | --- | --- | --- | --- | --- | --- | --- | --- | --- | --- | --- | --- | --- | --- | --- | --- | --- | --- | --- | --- | --- | --- | --- | --- | --- | --- | --- | --- | --- | --- | --- | --- | --- | --- | --- | --- | --- | --- | --- | --- | --- | --- | --- | --- | --- | --- | --- | --- | --- | --- | --- | --- | --- | --- | --- | --- | --- | --- | --- | --- | --- | --- | --- | --- | --- | --- | --- | --- | --- | --- | --- | --- | --- | --- | --- | --- | --- | --- | --- | --- | --- | --- | --- | --- | --- | --- | --- | --- | --- | --- | --- | --- | --- | --- | --- | --- | --- | --- | --- | --- | --- | --- | --- | --- | --- | --- | --- | --- | --- | --- | --- | --- | --- | --- | --- | --- | --- | --- | --- | --- | --- | --- | --- | --- | --- | --- | --- | --- | --- | --- | --- | --- | --- | --- | --- | --- | --- | --- | --- | --- | --- | --- | --- | --- | --- | --- | --- | --- | --- | --- | --- | --- | --- | --- | --- | --- | --- | --- | --- | --- | --- | --- | --- | --- | --- | --- | --- | --- | --- | --- | --- | --- | --- | --- | --- | --- | --- | --- | --- | --- | --- | --- | --- | --- | --- | --- | --- | --- | --- | --- | --- | --- | --- | --- | --- | --- | --- | --- | --- | --- | --- | --- | --- | --- | --- | --- | --- | --- | --- | --- | --- | --- | --- | --- | --- | --- | --- | --- | --- | --- | --- | --- | --- | --- | --- | --- | --- | --- | --- | --- | --- | --- | --- | --- | --- | --- | --- | --- | --- | --- | --- | --- | --- | --- | --- | --- | --- | --- | --- | --- | --- | --- | --- | --- | --- | --- | --- | --- | --- | --- | --- | --- | --- | --- | --- | --- | --- | --- | --- | --- | --- | --- | --- | --- | --- | --- | --- | --- | --- | --- | --- | --- | --- | --- | --- | --- | --- | --- | --- | --- | --- | --- | --- | --- | --- | --- | --- | --- | --- | --- | --- | --- | --- | --- | --- | --- | --- | --- | --- | --- | --- | --- | --- | --- | --- | --- | --- | --- | --- | --- | --- | --- | --- | --- | --- | --- | --- | --- | --- | --- | --- | --- | --- | --- | --- | --- | --- | --- | --- | --- | --- | --- | --- | --- | --- | --- | --- | --- | --- | --- | --- | --- | --- | --- | --- | --- | --- | --- | --- | --- | --- | --- | --- | --- | --- | --- | --- | --- | --- | --- | --- | --- | --- | --- | --- | --- | --- | --- | --- | --- | --- | --- | --- | --- | --- | --- | --- | --- | --- | --- | --- | --- | --- | --- | --- | --- | --- | --- | --- | --- | --- | --- | --- | --- | --- | --- | --- | --- | --- | --- | --- | --- | --- | --- | --- | --- | --- | --- | --- | --- | --- | --- | --- | --- | --- | --- | --- | --- | --- | --- | --- | --- | --- | --- | --- | --- | --- | --- | --- | --- | --- | --- | --- | --- | --- | --- | --- | --- | --- | --- | --- | --- | --- | --- | --- | --- | --- | --- | --- | --- | --- | --- | --- | --- | --- | --- | --- | --- | --- | --- | --- | --- | --- | --- | --- | --- | --- | --- | --- | --- | --- | --- | --- | --- | --- | --- | --- | --- | --- | --- | --- | --- | --- | --- | --- | --- | --- | --- | --- | --- | --- | --- | --- | --- | --- | --- | --- | --- | --- | --- | --- | --- | --- | --- | --- | --- | --- | --- | --- | --- | --- | --- | --- | --- | --- | --- | --- | --- | --- | --- | --- | --- | --- | --- | --- | --- | --- | --- | --- | --- | --- | --- | --- | --- | --- | --- | --- | --- | --- | --- | --- | --- | --- | --- | --- | --- | --- | --- | --- | --- | --- | --- | --- | --- | --- | --- | --- | --- | --- | --- | --- | --- | --- | --- | --- | --- | --- | --- | --- | --- | --- | --- | --- | --- | --- | --- | --- | --- | --- | --- | --- | --- | --- | --- | --- | --- | --- | --- | --- | --- | --- | --- | --- | --- | --- | --- | --- | --- | --- | --- | --- | --- | --- | --- | --- | --- | --- | --- | --- | --- | --- | --- | --- | --- | --- | --- | --- | --- | --- | --- | --- | --- | --- | --- | --- | --- | --- | --- | --- | --- | --- | --- | --- | --- | --- | --- | --- | --- | --- | --- | --- | --- | --- | --- | --- | --- | --- | --- | --- | --- | --- | --- | --- | --- | --- | --- | --- | --- | --- | --- | --- | --- | --- | --- | --- | --- | --- | --- | --- | --- | --- | --- | --- | --- | --- | --- | --- | --- | --- | --- | --- | --- | --- | --- | --- | --- | --- | --- | --- | --- | --- | --- | --- | --- | --- | --- | --- | --- | --- | --- | --- | --- | --- | --- | --- | --- | --- | --- | --- | --- | --- | --- | --- | --- | --- | --- | --- | --- | --- | --- | --- | --- | --- | --- | --- | --- | --- | --- | --- | --- | --- | --- | --- | --- | --- | --- | --- | --- | --- | --- | --- | --- | --- | --- | --- | --- | --- | --- | --- | --- | --- | --- | --- | --- | --- | --- | --- | --- | --- | --- | --- | --- | --- | --- | --- | --- | --- | --- | --- | --- | --- | --- | --- | --- | --- | --- | --- | --- | --- | --- | --- | --- | --- | --- | --- | --- | --- | --- | --- | --- | --- | --- | --- | --- | --- | --- | --- | --- | --- | --- | --- | --- | --- | --- | --- | --- | --- | --- | --- | --- | --- | --- | --- | --- | --- | --- | --- | --- | --- | --- | --- | --- | --- | --- | --- | --- | --- | --- | --- | --- | --- | --- | --- | --- | --- | --- | --- | --- | --- | --- | --- | --- | --- | --- | --- | --- | --- | --- | --- | --- | --- | --- | --- | --- | --- | --- | --- | --- | --- | --- | --- | --- | --- | --- | --- | --- | --- | --- | --- | --- | --- | --- | --- | --- | --- | --- | --- | --- | --- | --- | --- | --- | --- | --- | --- | --- | --- | --- | --- | --- | --- | --- | --- | --- | --- | --- | --- | --- | --- | --- | --- | --- | --- | --- | --- | --- | --- | --- | --- | --- | --- | --- | --- | --- | --- | --- | --- | --- | --- | --- | --- | --- | --- | --- | --- | --- | --- | --- | --- | --- | --- | --- | --- | --- | --- | --- | --- | --- | --- | --- | --- | --- | --- | --- | --- | --- | --- | --- | --- | --- | --- | --- | --- | --- | --- | --- | --- | --- | --- | --- | --- | --- | --- | --- | --- | --- | --- | --- | --- | --- | --- | --- | --- | --- | --- | --- | --- | --- | --- | --- | --- | --- | --- | --- | --- | --- | --- | --- | --- | --- | --- | --- | --- | --- | --- | --- | --- | --- | --- | --- | --- | --- | --- | --- | --- | --- | --- | --- | --- | --- | --- | --- | --- | --- | --- | --- | --- | --- | --- | --- | --- | --- | --- | --- | --- | --- | --- | --- | --- | --- | --- | --- | --- | --- | --- | --- | --- | --- | --- | --- | --- | --- | --- | --- | --- | --- | --- | --- | --- | --- | --- | --- | --- | --- | --- | --- | --- | --- | --- | --- | --- | --- | --- | --- | --- | --- | --- | --- | --- | --- | --- | --- | --- | --- | --- | --- | --- | --- | --- | --- | --- | --- | --- | --- | --- | --- | --- | --- | --- | --- | --- | --- | --- | --- | --- | --- | --- | --- | --- | --- | --- | --- | --- | --- | --- | --- | --- | --- | --- | --- | --- | --- | --- | --- | --- | --- | --- | --- | --- | --- | --- | --- | --- | --- | --- | --- | --- | --- | --- | --- | --- | --- | --- | --- | --- | --- | --- | --- | --- | --- | --- | --- | --- | --- | --- | --- | --- | --- | --- | --- | --- | --- | --- | --- | --- | --- | --- | --- | --- | --- | --- | --- | --- | --- | --- | --- | --- | --- | --- | --- | --- | --- | --- | --- | --- | --- | --- | --- | --- | --- | --- | --- | --- | --- | --- | --- | --- | --- | --- | --- | --- | --- | --- | --- | --- | --- | --- | --- | --- | --- | --- | --- | --- | --- | --- | --- | --- | --- | --- | --- | --- | --- | --- | --- | --- | --- | --- | --- | --- | --- | --- | --- | --- | --- | --- | --- | --- | --- | --- | --- | --- | --- | --- | --- | --- | --- | --- | --- | --- | --- | --- | --- | --- | --- | --- | --- | --- | --- | --- | --- | --- | --- | --- | --- | --- | --- | --- | --- | --- | --- | --- | --- | --- | --- | --- | --- | --- | --- | --- | --- | --- | --- | --- | --- | --- | --- | --- | --- | --- | --- | --- | --- | --- | --- | --- | --- | --- | --- | --- | --- | --- | --- | --- | --- | --- | --- | --- | --- | --- | --- | --- | --- | --- | --- | --- | --- | --- | --- | --- | --- | --- | --- | --- | --- | --- | --- | --- | --- | --- | --- | --- | --- | --- | --- | --- | --- | --- | --- | --- | --- | --- | --- | --- | --- | --- | --- | --- | --- | --- | --- | --- | --- | --- | --- | --- | --- | --- | --- | --- | --- | --- | --- | --- | --- | --- | --- | --- | --- | --- | --- | --- | --- | --- | --- | --- | --- | --- | --- | --- | --- | --- | --- | --- | --- | --- | --- | --- | --- | --- | --- | --- | --- | --- | --- | --- | --- | --- | --- | --- | --- | --- | --- | --- | --- | --- | --- | --- | --- | --- | --- | --- | --- | --- | --- | --- | --- | --- | --- | --- | --- | --- | --- | --- | --- | --- | --- | --- | --- | --- | --- | --- | --- | --- | --- | --- | --- | --- | --- | --- | --- | --- | --- | --- | --- | --- | --- | --- | --- | --- | --- | --- | --- | --- | --- | --- | --- | --- | --- | --- | --- | --- | --- | --- | --- | --- | --- | --- | --- | --- | --- | --- | --- | --- | --- | --- | --- | --- | --- | --- | --- | --- | --- | --- | --- | --- | --- | --- | --- | --- | --- | --- | --- | --- | --- | --- | --- | --- | --- | --- | --- | --- | --- | --- | --- | --- | --- | --- | --- | --- | --- | --- | --- | --- | --- | --- | --- | --- | --- | --- | --- | --- | --- | --- | --- | --- | --- | --- | --- | --- | --- | --- | --- | --- | --- | --- | --- | --- | --- | --- | --- | --- | --- | --- | --- | --- | --- | --- | --- | --- | --- | --- | --- | --- | --- | --- | --- | --- | --- | --- | --- | --- | --- | --- | --- | --- | --- | --- | --- | --- | --- | --- | --- | --- | --- | --- | --- | --- | --- | --- | --- | --- | --- | --- | --- | --- | --- | --- | --- | --- | --- | --- | --- | --- | --- | --- | --- | --- | --- | --- | --- | --- | --- | --- | --- | --- | --- | --- | --- | --- | --- | --- | --- | --- | --- | --- | --- | --- | --- | --- | --- | --- | --- | --- | --- | --- | --- | --- | --- | --- | --- | --- | --- | --- | --- | --- | --- | --- | --- | --- | --- | --- | --- | --- | --- | --- | --- | --- | --- | --- | --- | --- | --- | --- | --- | --- | --- | --- | --- | --- | --- | --- | --- | --- | --- | --- | --- | --- | --- | --- | --- | --- | --- | --- | --- | --- | --- | --- | --- | --- | --- | --- | --- | --- | --- | --- | --- | --- | --- | --- | --- | --- | --- | --- | --- | --- | --- | --- | --- | --- | --- | --- | --- | --- | --- | --- | --- | --- | --- | --- | --- | --- | --- | --- | --- | --- | --- | --- | --- | --- | --- | --- | --- | --- | --- | --- | --- | --- | --- | --- | --- | --- | --- | --- | --- | --- | --- | --- | --- | --- | --- | --- | --- | --- | --- | --- | --- | --- | --- | --- | --- | --- | --- | --- | --- | --- | --- | --- | --- | --- | --- | --- | --- | --- | --- | --- | --- | --- | --- | --- | --- | --- | --- | --- | --- | --- | --- | --- | --- | --- | --- | --- | --- | --- | --- | --- | --- | --- | --- | --- | --- | --- | --- | --- | --- | --- | --- | --- | --- | --- | --- | --- | --- | --- | --- | --- | --- | --- | --- | --- | --- | --- | --- | --- | --- | --- | --- | --- | --- | --- | --- | --- | --- | --- | --- | --- | --- | --- | --- | --- | --- | --- | --- | --- | --- | --- | --- | --- | --- | --- | --- | --- | --- | --- | --- | --- | --- | --- | --- | --- | --- | --- | --- | --- | --- | --- | --- | --- | --- | --- | --- | --- | --- | --- | --- | --- | --- | --- | --- | --- | --- | --- | --- | --- | --- | --- | --- | --- | --- | --- | --- | --- | --- | --- | --- | --- | --- | --- | --- | --- | --- | --- | --- | --- | --- | --- | --- | --- | --- | --- | --- | --- | --- | --- | --- | --- | --- | --- | --- | --- | --- | --- | --- | --- | --- | --- | --- | --- | --- | --- | --- | --- | --- | --- | --- | --- | --- | --- | --- | --- | --- | --- | --- | --- | --- | --- | --- | --- | --- | --- | --- | --- | --- | --- | --- | --- | --- | --- | --- | --- | --- | --- | --- | --- | --- | --- | --- | --- | --- | --- | --- | --- | --- | --- | --- | --- | --- | --- | --- | --- | --- | --- | --- | --- | --- | --- | --- | --- | --- | --- | --- | --- | --- | --- | --- | --- | --- | --- | --- | --- | --- | --- | --- | --- | --- | --- | --- | --- | --- | --- | --- | --- | --- | --- | --- | --- | --- | --- | --- | --- | --- | --- | --- | --- | --- | --- | --- | --- | --- | --- | --- | --- | --- | --- | --- | --- | --- | --- | --- | --- | --- | --- | --- | --- | --- | --- | --- | --- | --- | --- | --- | --- | --- | --- | --- | --- | --- | --- | --- | --- | --- | --- | --- | --- | --- | --- | --- | --- | --- | --- | --- | --- | --- | --- | --- | --- | --- | --- | --- | --- | --- | --- | --- | --- | --- | --- | --- | --- | --- | --- | --- | --- | --- | --- | --- | --- | --- | --- | --- | --- | --- | --- | --- | --- | --- | --- | --- | --- | --- | --- | --- | --- | --- | --- | --- | --- | --- | --- | --- | --- | --- | --- | --- | --- | --- | --- | --- | --- | --- | --- | --- | --- | --- | --- | --- | --- | --- | --- | --- | --- | --- | --- | --- | --- | --- | --- | --- | --- | --- | --- | --- | --- | --- | --- | --- | --- | --- | --- | --- | --- | --- | --- | --- | --- | --- | --- | --- | --- | --- | --- | --- | --- | --- | --- | --- | --- | --- | --- | --- | --- | --- | --- | --- | --- | --- | --- | --- | --- | --- | --- | --- | --- | --- | --- | --- | --- | --- | --- | --- | --- | --- | --- | --- | --- | --- | --- | --- | --- | --- | --- | --- | --- | --- | --- | --- | --- | --- | --- | --- | --- | --- | --- | --- | --- | --- | --- | --- | --- | --- | --- | --- | --- | --- | --- | --- | --- | --- | --- | --- | --- | --- | --- | --- | --- | --- | --- | --- | --- | --- | --- | --- | --- | --- | --- | --- | --- | --- | --- | --- | --- | --- | --- | --- | --- | --- | --- | --- | --- | --- | --- | --- | --- | --- | --- | --- | --- | --- | --- | --- | --- | --- | --- | --- | --- | --- | --- | --- | --- | --- | --- | --- | --- | --- | --- | --- | --- | --- | --- | --- | --- | --- | --- | --- | --- | --- | --- | --- | --- | --- | --- | --- | --- | --- | --- | --- | --- | --- | --- | --- | --- | --- | --- | --- | --- | --- | --- | --- | --- | --- | --- | --- | --- | --- | --- | --- | --- | --- | --- | --- | --- | --- | --- | --- | --- | --- | --- | --- | --- | --- | --- | --- | --- | --- | --- | --- | --- | --- | --- | --- | --- | --- | --- | --- | --- | --- | --- | --- | --- | --- | --- | --- | --- | --- | --- | --- | --- | --- | --- | --- | --- | --- | --- | --- | --- | --- | --- | --- | --- | --- | --- | --- | --- | --- | --- | --- | --- | --- | --- | --- | --- | --- | --- | --- | --- | --- | --- | --- | --- | --- | --- | --- | --- | --- | --- | --- | --- | --- | --- | --- | --- | --- | --- | --- | --- | --- | --- | --- | --- | --- | --- | --- | --- | --- | --- | --- | --- | --- | --- | --- | --- | --- | --- | --- | --- | --- | --- | --- | --- | --- | --- | --- | --- | --- | --- | --- | --- | --- | --- | --- | --- | --- | --- | --- | --- | --- | --- | --- | --- | --- | --- | --- | --- | --- | --- | --- | --- | --- | --- | --- | --- | --- | --- | --- | --- | --- | --- | --- | --- | --- | --- | --- | --- | --- | --- | --- | --- | --- | --- | --- | --- | --- | --- | --- | --- | --- | --- | --- | --- | --- | --- | --- | --- | --- | --- | --- | --- | --- | --- | --- | --- | --- | --- | --- | --- | --- | --- | --- | --- | --- | --- | --- | --- | --- | --- | --- | --- | --- | --- | --- | --- | --- | --- | --- | --- | --- | --- | --- | --- | --- | --- | --- | --- | --- | --- | --- | --- | --- | --- | --- | --- | --- | --- | --- | --- | --- | --- | --- | --- | --- | --- | --- | --- | --- | --- | --- | --- | --- | --- | --- | --- | --- | --- | --- | --- | --- | --- | --- | --- | --- | --- | --- | --- | --- | --- | --- | --- | --- | --- | --- | --- | --- | --- | --- | --- | --- | --- | --- | --- | --- | --- | --- | --- | --- | --- | --- | --- | --- | --- | --- | --- | --- | --- | --- | --- | --- | --- | --- | --- | --- | --- | --- | --- | --- | --- | --- | --- | --- | --- | --- | --- | --- | --- | --- | --- | --- | --- | --- | --- | --- | --- | --- | --- | --- | --- | --- | --- | --- | --- | --- | --- | --- | --- | --- | --- | --- | --- | --- | --- | --- | --- | --- | --- | --- | --- | --- | --- | --- | --- | --- | --- | --- | --- | --- | --- | --- | --- | --- | --- | --- | --- | --- | --- | --- | --- | --- | --- | --- | --- | --- | --- | --- | --- | --- | --- | --- | --- | --- | --- | --- | --- | --- | --- | --- | --- | --- | --- | --- | --- | --- | --- | --- | --- | --- | --- | --- | --- | --- | --- | --- | --- | --- | --- | --- | --- | --- | --- | --- | --- | --- | --- | --- | --- | --- | --- | --- | --- | --- | --- | --- | --- | --- | --- | --- | --- | --- | --- | --- | --- | --- | --- | --- | --- | --- | --- | --- | --- | --- | --- | --- | --- | --- | --- | --- | --- | --- | --- | --- | --- | --- | --- | --- | --- | --- | --- | --- | --- | --- | --- | --- | --- | --- | --- | --- | --- | --- | --- | --- | --- | --- | --- | --- | --- | --- | --- | --- | --- | --- | --- | --- | --- | --- | --- | --- | --- | --- | --- | --- | --- | --- | --- | --- | --- | --- | --- | --- | --- | --- | --- | --- | --- | --- | --- | --- | --- | --- | --- | --- | --- | --- | --- | --- | --- | --- | --- | --- | --- | --- | --- | --- | --- | --- | --- | --- | --- | --- | --- | --- | --- | --- | --- | --- | --- | --- | --- | --- | --- | --- | --- | --- | --- | --- | --- | --- | --- | --- | --- | --- | --- | --- | --- | --- | --- | --- | --- | --- | --- | --- | --- | --- | --- | --- | --- | --- | --- | --- | --- | --- | --- | --- | --- | --- | --- | --- | --- | --- | --- | --- | --- | --- | --- | --- | --- | --- | --- | --- | --- | --- | --- | --- | --- | --- | --- | --- | --- | --- | --- | --- | --- | --- | --- | --- | --- | --- | --- | --- | --- | --- | --- | --- | --- | --- | --- | --- | --- | --- | --- | --- | --- | --- | --- | --- | --- | --- | --- | --- | --- | --- | --- | --- | --- | --- | --- | --- | --- | --- | --- | --- | --- | --- | --- | --- | --- | --- | --- | --- | --- | --- | --- | --- | --- | --- | --- | --- | --- | --- | --- | --- | --- | --- | --- | --- | --- | --- | --- | --- | --- | --- | --- | --- | --- | --- | --- | --- | --- | --- | --- | --- | --- | --- | --- | --- | --- | --- | --- | --- | --- | --- | --- | --- | --- | --- | --- | --- | --- | --- | --- | --- | --- | --- | --- | --- | --- | --- | --- | --- | --- | --- | --- | --- | --- | --- | --- | --- | --- | --- | --- | --- | --- | --- | --- | --- | --- | --- | --- | --- | --- | --- | --- | --- | --- | --- | --- | --- | --- | --- | --- | --- | --- | --- | --- | --- | --- | --- | --- | --- | --- | --- | --- | --- | --- | --- | --- | --- | --- | --- | --- | --- | --- | --- | --- | --- | --- | --- | --- | --- | --- | --- | --- | --- | --- | --- | --- | --- | --- | --- | --- | --- | --- | --- | --- | --- | --- | --- | --- | --- | --- | --- | --- | --- | --- | --- | --- | --- | --- | --- | --- | --- | --- | --- | --- | --- | --- | --- | --- | --- | --- | --- | --- | --- | --- | --- | --- | --- | --- | --- | --- | --- | --- | --- | --- | --- | --- | --- | --- | --- | --- | --- | --- | --- | --- | --- | --- | --- | --- | --- | --- | --- | --- | --- | --- | --- | --- | --- | --- | --- | --- | --- | --- | --- | --- | --- | --- | --- | --- | --- | --- | --- | --- | --- | --- | --- | --- | --- | --- | --- | --- | --- | --- | --- | --- | --- | --- | --- | --- | --- | --- | --- | --- | --- | --- | --- | --- | --- | --- | --- | --- | --- | --- | --- | --- | --- | --- | --- | --- | --- | --- | --- | --- | --- | --- | --- | --- | --- | --- | --- | --- | --- | --- | --- | --- | --- | --- | --- | --- | --- | --- | --- | --- | --- | --- | --- | --- | --- | --- | --- | --- | --- | --- | --- | --- | --- | --- | --- | --- | --- | --- | --- | --- | --- | --- | --- | --- | --- | --- | --- | --- | --- | --- | --- | --- | --- | --- | --- | --- | --- | --- | --- | --- | --- | --- | --- | --- | --- | --- | --- | --- | --- | --- | --- | --- | --- | --- | --- | --- | --- | --- | --- | --- | --- | --- | --- | --- | --- | --- | --- | --- | --- | --- | --- | --- | --- | --- | --- | --- | --- | --- | --- | --- | --- | --- | --- | --- | --- | --- | --- | --- | --- | --- | --- | --- | --- | --- | --- | --- | --- | --- | --- | --- | --- | --- | --- | --- | --- | --- | --- | --- | --- | --- | --- | --- | --- | --- | --- | --- | --- | --- | --- | --- | --- | --- | --- | --- | --- | --- | --- | --- | --- | --- | --- | --- | --- | --- | --- | --- | --- | --- | --- | --- | --- | --- | --- | --- | --- | --- | --- | --- | --- | --- | --- | --- | --- | --- | --- | --- | --- | --- | --- | --- | --- | --- | --- | --- | --- | --- | --- | --- | --- | --- | --- | --- | --- | --- | --- | --- | --- | --- | --- | --- | --- | --- | --- | --- | --- | --- | --- | --- | --- | --- | --- | --- | --- | --- | --- | --- | --- | --- | --- | --- | --- | --- | --- | --- | --- | --- | --- | --- | --- | --- | --- | --- | --- | --- | --- | --- | --- | --- | --- | --- | --- | --- | --- | --- | --- | --- | --- | --- | --- | --- | --- | --- | --- | --- | --- | --- | --- | --- | --- | --- | --- | --- | --- | --- | --- | --- | --- | --- | --- | --- | --- | --- | --- | --- | --- | --- | --- | --- | --- | --- | --- | --- | --- | --- | --- | --- | --- | --- | --- | --- | --- | --- | --- | --- | --- | --- | --- | --- | --- | --- | --- | --- | --- | --- | --- | --- | --- | --- | --- | --- | --- | --- | --- | --- | --- | --- | --- | --- | --- | --- | --- | --- | --- | --- | --- | --- | --- | --- | --- | --- | --- | --- | --- | --- | --- | --- | --- | --- | --- | --- | --- | --- | --- | --- | --- | --- | --- | --- | --- | --- | --- | --- | --- | --- | --- | --- | --- | --- | --- | --- | --- | --- | --- | --- | --- | --- | --- | --- | --- | --- | --- | --- | --- | --- | --- | --- | --- | --- | --- | --- | --- | --- | --- | --- | --- | --- | --- | --- | --- | --- | --- | --- | --- | --- | --- | --- | --- | --- | --- | --- | --- | --- | --- | --- | --- | --- | --- | --- | --- | --- | --- | --- | --- | --- | --- | --- | --- | --- | --- | --- | --- | --- | --- | --- | --- | --- | --- | --- | --- | --- | --- | --- | --- | --- | --- | --- | --- | --- | --- | --- | --- | --- | --- | --- | --- | --- | --- | --- | --- | --- | --- | --- | --- | --- | --- | --- | --- | --- | --- | --- | --- | | | | | *** Misclassified observation** | | --- | | | --- | --- | | |  | | | | | | **Discriminant Analysis Results** | | --- | | | --- | --- | | | | **The DISCRIM Procedure Classification Summary for Calibration Data: WORK.SORTTEMPTABLESORTED Resubstitution Summary using Linear Discriminant Function** | | --- | | | --- | --- | | | | **Number of Observations and Percent Classified into DIST** | | | | | | | | | --- | --- | --- | --- | --- | --- | --- | --- | | **From DIST** | **1** | **2** | **3** | **4** | **5** | **6** | **Total** | | 1 | | 81 | | --- | | 48.80 | | | 27 | | --- | | 16.27 | | | 23 | | --- | | 13.86 | | | 13 | | --- | | 7.83 | | | 22 | | --- | | 13.25 | | | 0 | | --- | | 0.00 | | | 166 | | --- | | 100.00 | | | 2 | | 23 | | --- | | 14.47 | | | 99 | | --- | | 62.26 | | | 4 | | --- | | 2.52 | | | 24 | | --- | | 15.09 | | | 3 | | --- | | 1.89 | | | 6 | | --- | | 3.77 | | | 159 | | --- | | 100.00 | | | 3 | | 25 | | --- | | 19.08 | | | 27 | | --- | | 20.61 | | | 45 | | --- | | 34.35 | | | 25 | | --- | | 19.08 | | | 9 | | --- | | 6.87 | | | 0 | | --- | | 0.00 | | | 131 | | --- | | 100.00 | | | 4 | | 13 | | --- | | 8.50 | | | 27 | | --- | | 17.65 | | | 18 | | --- | | 11.76 | | | 89 | | --- | | 58.17 | | | 6 | | --- | | 3.92 | | | 0 | | --- | | 0.00 | | | 153 | | --- | | 100.00 | | | 5 | | 19 | | --- | | 13.01 | | | 16 | | --- | | 10.96 | | | 6 | | --- | | 4.11 | | | 11 | | --- | | 7.53 | | | 92 | | --- | | 63.01 | | | 2 | | --- | | 1.37 | | | 146 | | --- | | 100.00 | | | 6 | | 1 | | --- | | 0.82 | | | 8 | | --- | | 6.56 | | | 5 | | --- | | 4.10 | | | 1 | | --- | | 0.82 | | | 9 | | --- | | 7.38 | | | 98 | | --- | | 80.33 | | | 122 | | --- | | 100.00 | | | Total | | 162 | | --- | | 18.47 | | | 204 | | --- | | 23.26 | | | 101 | | --- | | 11.52 | | | 163 | | --- | | 18.59 | | | 141 | | --- | | 16.08 | | | 106 | | --- | | 12.09 | | | 877 | | --- | | 100.00 | | | Priors | | 0.18928 | | --- | |  | | | 0.1813 | | --- | |  | | | 0.14937 | | --- | |  | | | 0.17446 | | --- | |  | | | 0.16648 | | --- | |  | | | 0.13911 | | --- | |  | | |  | | --- | |  | | | | --- | --- | --- | --- | --- | --- | --- | --- | --- | --- | --- | --- | --- | --- | --- | --- | --- | --- | --- | --- | --- | --- | --- | --- | --- | --- | --- | --- | --- | --- | --- | --- | --- | --- | --- | --- | --- | --- | --- | --- | --- | --- | --- | --- | --- | --- | --- | --- | --- | --- | --- | --- | --- | --- | --- | --- | --- | --- | --- | --- | --- | --- | --- | --- | --- | --- | --- | --- | --- | --- | --- | --- | --- | --- | --- | --- | --- | --- | --- | --- | --- | --- | --- | --- | --- | --- | --- | --- | --- | --- | --- | --- | --- | --- | --- | --- | --- | --- | --- | --- | --- | --- | --- | --- | --- | --- | --- | --- | --- | --- | --- | --- | --- | --- | --- | --- | --- | --- | --- | --- | --- | --- | --- | --- | --- | --- | --- | --- | --- | --- | --- | --- | --- | --- | --- | --- | --- | --- | --- | --- | --- | --- | --- | --- | --- | --- | --- | --- | --- | --- | --- | --- | --- | --- | --- | --- | --- | --- | --- | --- | --- | --- | --- | --- | --- | --- | --- | --- | --- | --- | --- | --- | --- | --- | --- | --- | --- | --- | --- | --- | --- | --- | --- | --- | --- | --- | --- | --- | --- | --- | --- | --- | --- | | | **Error Count Estimates for DIST** | | | | | | | | | --- | --- | --- | --- | --- | --- | --- | --- | |  | **1** | **2** | **3** | **4** | **5** | **6** | **Total** | | Rate | 0.5120 | 0.3774 | 0.6565 | 0.4183 | 0.3699 | 0.1967 | 0.4253 | | Priors | 0.1893 | 0.1813 | 0.1494 | 0.1745 | 0.1665 | 0.1391 |  | | | |  | | | | | | **Discriminant Analysis Results** | | --- | | | --- | --- | | | | **The DISCRIM Procedure Classification Results for Calibration Data: WORK.SORTTEMPTABLESORTED Resubstitution Results using Linear Discriminant Function** | | --- | | | --- | --- | | | | **Number of Observations and Average Posterior Probabilities Classified into DIST** | | | | | | | | --- | --- | --- | --- | --- | --- | --- | | **From DIST** | **1** | **2** | **3** | **4** | **5** | **6** | | 1 | | 81 | | --- | | 0.5302 | | | 27 | | --- | | 0.4798 | | | 23 | | --- | | 0.4012 | | | 13 | | --- | | 0.4260 | | | 22 | | --- | | 0.5265 | | | 0 | | --- | | . | | | 2 | | 23 | | --- | | 0.3856 | | | 99 | | --- | | 0.5931 | | | 4 | | --- | | 0.2983 | | | 24 | | --- | | 0.4991 | | | 3 | | --- | | 0.5326 | | | 6 | | --- | | 0.4373 | | | 3 | | 25 | | --- | | 0.5289 | | | 27 | | --- | | 0.4147 | | | 45 | | --- | | 0.4933 | | | 25 | | --- | | 0.5220 | | | 9 | | --- | | 0.3788 | | | 0 | | --- | | . | | | 4 | | 13 | | --- | | 0.3955 | | | 27 | | --- | | 0.4277 | | | 18 | | --- | | 0.4010 | | | 89 | | --- | | 0.5268 | | | 6 | | --- | | 0.5507 | | | 0 | | --- | | . | | | 5 | | 19 | | --- | | 0.4812 | | | 16 | | --- | | 0.4305 | | | 6 | | --- | | 0.3877 | | | 11 | | --- | | 0.5639 | | | 92 | | --- | | 0.7126 | | | 2 | | --- | | 0.3836 | | | 6 | | 1 | | --- | | 0.4799 | | | 8 | | --- | | 0.5965 | | | 5 | | --- | | 0.3530 | | | 1 | | --- | | 0.3182 | | | 9 | | --- | | 0.7408 | | | 98 | | --- | | 0.9202 | | | Total | | 162 | | --- | | 0.4926 | | | 204 | | --- | | 0.5200 | | | 101 | | --- | | 0.4349 | | | 163 | | --- | | 0.5152 | | | 141 | | --- | | 0.6534 | | | 106 | | --- | | 0.8828 | | | Priors | | 0.18928 | | --- | |  | | | 0.1813 | | --- | |  | | | 0.14937 | | --- | |  | | | 0.17446 | | --- | |  | | | 0.16648 | | --- | |  | | | 0.13911 | | --- | |  | | | | --- | --- | --- | --- | --- | --- | --- | --- | --- | --- | --- | --- | --- | --- | --- | --- | --- | --- | --- | --- | --- | --- | --- | --- | --- | --- | --- | --- | --- | --- | --- | --- | --- | --- | --- | --- | --- | --- | --- | --- | --- | --- | --- | --- | --- | --- | --- | --- | --- | --- | --- | --- | --- | --- | --- | --- | --- | --- | --- | --- | --- | --- | --- | --- | --- | --- | --- | --- | --- | --- | --- | --- | --- | --- | --- | --- | --- | --- | --- | --- | --- | --- | --- | --- | --- | --- | --- | --- | --- | --- | --- | --- | --- | --- | --- | --- | --- | --- | --- | --- | --- | --- | --- | --- | --- | --- | --- | --- | --- | --- | --- | --- | --- | --- | --- | --- | --- | --- | --- | --- | --- | --- | --- | --- | --- | --- | --- | --- | --- | --- | --- | --- | --- | --- | --- | --- | --- | --- | --- | --- | --- | --- | --- | --- | --- | --- | --- | --- | --- | --- | --- | --- | --- | --- | --- | --- | --- | --- | --- | --- | --- | --- | --- | --- | --- | --- | --- | | | **Posterior Probability Error Rate Estimates for DIST** | | | | | | | | | --- | --- | --- | --- | --- | --- | --- | --- | | **Estimate** | **1** | **2** | **3** | **4** | **5** | **6** | **Total** | | Stratified | 0.5193 | 0.3329 | 0.6647 | 0.4511 | 0.3690 | 0.2330 | 0.4305 | | Unstratified | 0.5193 | 0.3329 | 0.6647 | 0.4511 | 0.3690 | 0.2330 | 0.4305 | | Priors | 0.1893 | 0.1813 | 0.1494 | 0.1745 | 0.1665 | 0.1391 |  | | | |  | | | | | | **Discriminant Analysis Results** | | --- | | | --- | --- | | | | **The DISCRIM Procedure Classification Results for Calibration Data: WORK.SORTTEMPTABLESORTED Cross-validation Results using Linear Discriminant Function** | | --- | | | --- | --- | | | | **Posterior Probability of Membership in DIST** | | | | | | | | | | | --- | --- | --- | --- | --- | --- | --- | --- | --- | --- | | **Obs** | **From DIST** | **Classified into DIST** | | **1** | **2** | **3** | **4** | **5** | **6** | | 1 | 1 | 1 |  | 0.6839 | 0.1517 | 0.0713 | 0.0357 | 0.0573 | 0.0000 | | 2 | 1 | 1 |  | 0.5074 | 0.2276 | 0.1227 | 0.1111 | 0.0312 | 0.0000 | | 3 | 1 | 2 | * | 0.2516 | 0.4906 | 0.1156 | 0.0976 | 0.0439 | 0.0007 | | 4 | 1 | 1 |  | 0.4093 | 0.0536 | 0.0917 | 0.0975 | 0.3477 | 0.0002 | | 5 | 1 | 3 | * | 0.1007 | 0.2197 | 0.4300 | 0.0890 | 0.1454 | 0.0152 | | 6 | 1 | 2 | * | 0.2691 | 0.2779 | 0.0709 | 0.1183 | 0.2460 | 0.0177 | | 7 | 1 | 4 | * | 0.1272 | 0.1093 | 0.1651 | 0.4956 | 0.1029 | 0.0000 | | 8 | 1 | 1 |  | 0.3140 | 0.1598 | 0.2444 | 0.1276 | 0.1539 | 0.0002 | | 9 | 1 | 3 | * | 0.3125 | 0.1283 | 0.4124 | 0.0958 | 0.0509 | 0.0000 | | 10 | 1 | 1 |  | 0.7084 | 0.0598 | 0.0386 | 0.0137 | 0.1795 | 0.0000 | | 11 | 1 | 3 | * | 0.1000 | 0.2142 | 0.4398 | 0.0881 | 0.1434 | 0.0145 | | 12 | 1 | 1 |  | 0.2761 | 0.2634 | 0.0813 | 0.1195 | 0.2446 | 0.0152 | | 13 | 1 | 4 | * | 0.2003 | 0.2277 | 0.2800 | 0.2851 | 0.0067 | 0.0002 | | 14 | 1 | 2 | * | 0.3517 | 0.3524 | 0.1223 | 0.0878 | 0.0856 | 0.0001 | | 15 | 1 | 5 | * | 0.4138 | 0.0285 | 0.0249 | 0.0184 | 0.5144 | 0.0000 | | 16 | 1 | 1 |  | 0.3480 | 0.2781 | 0.0636 | 0.0626 | 0.2412 | 0.0065 | | 17 | 1 | 5 | * | 0.3503 | 0.0448 | 0.1029 | 0.1211 | 0.3807 | 0.0002 | | 18 | 1 | 3 | * | 0.0990 | 0.2371 | 0.3650 | 0.0884 | 0.1819 | 0.0286 | | 19 | 1 | 2 | * | 0.1355 | 0.4971 | 0.1568 | 0.1933 | 0.0163 | 0.0009 | | 20 | 1 | 2 | * | 0.2532 | 0.4845 | 0.1197 | 0.0979 | 0.0439 | 0.0007 | | 21 | 1 | 4 | * | 0.0719 | 0.0910 | 0.1562 | 0.6355 | 0.0454 | 0.0000 | | 22 | 1 | 1 |  | 0.7326 | 0.1402 | 0.0294 | 0.0174 | 0.0803 | 0.0000 | | 23 | 1 | 5 | * | 0.3676 | 0.0561 | 0.0859 | 0.1014 | 0.3886 | 0.0003 | | 24 | 1 | 2 | * | 0.2391 | 0.2956 | 0.1567 | 0.1998 | 0.0951 | 0.0137 | | 25 | 1 | 1 |  | 0.5085 | 0.2198 | 0.1303 | 0.1107 | 0.0308 | 0.0000 | | 26 | 1 | 2 | * | 0.2215 | 0.5147 | 0.1279 | 0.0958 | 0.0392 | 0.0009 | | 27 | 1 | 1 |  | 0.7437 | 0.1437 | 0.0264 | 0.0127 | 0.0735 | 0.0000 | | 28 | 1 | 1 |  | 0.3165 | 0.1620 | 0.2838 | 0.1847 | 0.0530 | 0.0000 | | 29 | 1 | 1 |  | 0.5306 | 0.2781 | 0.1096 | 0.0514 | 0.0301 | 0.0002 | | 30 | 1 | 3 | * | 0.2001 | 0.1194 | 0.5011 | 0.1532 | 0.0261 | 0.0000 | | 31 | 1 | 3 | * | 0.1410 | 0.2945 | 0.4135 | 0.0679 | 0.0557 | 0.0274 | | 32 | 1 | 1 |  | 0.3634 | 0.1114 | 0.1547 | 0.0690 | 0.3015 | 0.0001 | | 33 | 1 | 3 | * | 0.1273 | 0.2645 | 0.4253 | 0.0967 | 0.0581 | 0.0280 | | 34 | 1 | 3 | * | 0.3005 | 0.1223 | 0.3705 | 0.1431 | 0.0636 | 0.0000 | | 35 | 1 | 1 |  | 0.5763 | 0.1879 | 0.1022 | 0.1015 | 0.0321 | 0.0000 | | 36 | 1 | 1 |  | 0.3399 | 0.1045 | 0.2165 | 0.2097 | 0.1281 | 0.0013 | | 37 | 1 | 4 | * | 0.1830 | 0.0987 | 0.1714 | 0.4114 | 0.1355 | 0.0000 | | 38 | 1 | 3 | * | 0.1903 | 0.1054 | 0.5360 | 0.1441 | 0.0242 | 0.0000 | | 39 | 1 | 3 | * | 0.1632 | 0.2049 | 0.3776 | 0.0808 | 0.1623 | 0.0111 | | 40 | 1 | 2 | * | 0.2074 | 0.3397 | 0.1314 | 0.1648 | 0.1333 | 0.0234 | | 41 | 1 | 1 |  | 0.3409 | 0.3022 | 0.0555 | 0.0679 | 0.2213 | 0.0121 | | 42 | 1 | 1 |  | 0.5404 | 0.2817 | 0.1231 | 0.0412 | 0.0133 | 0.0002 | | 43 | 1 | 3 | * | 0.0900 | 0.2325 | 0.3297 | 0.1206 | 0.1925 | 0.0347 | | 44 | 1 | 2 | * | 0.1355 | 0.5468 | 0.1289 | 0.1715 | 0.0162 | 0.0011 | | 45 | 1 | 4 | * | 0.0874 | 0.1211 | 0.1470 | 0.5925 | 0.0519 | 0.0001 | | 46 | 1 | 1 |  | 0.5499 | 0.2627 | 0.1385 | 0.0363 | 0.0126 | 0.0001 | | 47 | 1 | 5 | * | 0.3598 | 0.1944 | 0.0325 | 0.0411 | 0.3701 | 0.0021 | | 48 | 1 | 1 |  | 0.6341 | 0.0589 | 0.0263 | 0.0382 | 0.2425 | 0.0001 | | 49 | 1 | 4 | * | 0.2316 | 0.0932 | 0.2548 | 0.3120 | 0.1082 | 0.0001 | | 50 | 1 | 2 | * | 0.3378 | 0.4305 | 0.0454 | 0.0318 | 0.1538 | 0.0007 | | 51 | 1 | 3 | * | 0.1269 | 0.0813 | 0.3233 | 0.2565 | 0.2117 | 0.0003 | | 52 | 1 | 5 | * | 0.2654 | 0.0222 | 0.0740 | 0.0096 | 0.6280 | 0.0007 | | 53 | 1 | 1 |  | 0.4537 | 0.1684 | 0.2362 | 0.0782 | 0.0633 | 0.0003 | | 54 | 1 | 5 | * | 0.1336 | 0.0459 | 0.2129 | 0.1522 | 0.4552 | 0.0001 | | 55 | 1 | 5 | * | 0.3117 | 0.0447 | 0.0144 | 0.0113 | 0.5398 | 0.0781 | | 56 | 1 | 1 |  | 0.5417 | 0.0196 | 0.0292 | 0.0195 | 0.3898 | 0.0003 | | 57 | 1 | 1 |  | 0.5174 | 0.0458 | 0.3052 | 0.0581 | 0.0735 | 0.0000 | | 58 | 1 | 1 |  | 0.3648 | 0.1399 | 0.0373 | 0.1795 | 0.2784 | 0.0000 | | 59 | 1 | 5 | * | 0.2742 | 0.0517 | 0.0096 | 0.0169 | 0.6472 | 0.0004 | | 60 | 1 | 5 | * | 0.4194 | 0.0146 | 0.0076 | 0.0126 | 0.5459 | 0.0000 | | 61 | 1 | 5 | * | 0.1338 | 0.0469 | 0.2072 | 0.1529 | 0.4591 | 0.0001 | | 62 | 1 | 1 |  | 0.3603 | 0.0773 | 0.1710 | 0.2400 | 0.1513 | 0.0000 | | 63 | 1 | 5 | * | 0.2909 | 0.1007 | 0.0131 | 0.0106 | 0.5846 | 0.0001 | | 64 | 1 | 5 | * | 0.2293 | 0.0140 | 0.0436 | 0.0045 | 0.7084 | 0.0003 | | 65 | 1 | 1 |  | 0.3074 | 0.1218 | 0.2703 | 0.1337 | 0.1665 | 0.0003 | | 66 | 1 | 5 | * | 0.0833 | 0.0272 | 0.1348 | 0.1318 | 0.6229 | 0.0001 | | 67 | 1 | 5 | * | 0.2978 | 0.0325 | 0.0149 | 0.0143 | 0.6300 | 0.0106 | | 68 | 1 | 5 | * | 0.4690 | 0.0165 | 0.0210 | 0.0119 | 0.4813 | 0.0003 | | 69 | 1 | 1 |  | 0.4683 | 0.0351 | 0.3439 | 0.0692 | 0.0835 | 0.0000 | | 70 | 1 | 1 |  | 0.3392 | 0.1414 | 0.0355 | 0.2397 | 0.2443 | 0.0000 | | 71 | 1 | 1 |  | 0.3510 | 0.1236 | 0.0399 | 0.1683 | 0.3172 | 0.0000 | | 72 | 1 | 5 | * | 0.1624 | 0.0601 | 0.0158 | 0.0439 | 0.7158 | 0.0021 | | 73 | 1 | 1 |  | 0.7085 | 0.0586 | 0.0250 | 0.0459 | 0.1619 | 0.0000 | | 74 | 1 | 4 | * | 0.1787 | 0.1047 | 0.2667 | 0.4019 | 0.0480 | 0.0001 | | 75 | 1 | 2 | * | 0.3225 | 0.4173 | 0.0711 | 0.0963 | 0.0904 | 0.0024 | | 76 | 1 | 5 | * | 0.2192 | 0.0262 | 0.0727 | 0.0175 | 0.6562 | 0.0082 | | 77 | 1 | 3 | * | 0.1906 | 0.1436 | 0.3769 | 0.2231 | 0.0638 | 0.0019 | | 78 | 1 | 5 | * | 0.1212 | 0.0487 | 0.2399 | 0.2166 | 0.3734 | 0.0002 | | 79 | 1 | 5 | * | 0.3623 | 0.0755 | 0.0308 | 0.0299 | 0.4210 | 0.0806 | | 80 | 1 | 1 |  | 0.5556 | 0.0249 | 0.0357 | 0.0226 | 0.3604 | 0.0008 | | 81 | 1 | 3 | * | 0.3875 | 0.0474 | 0.4126 | 0.0991 | 0.0534 | 0.0000 | | 82 | 1 | 1 |  | 0.4523 | 0.1647 | 0.2423 | 0.0777 | 0.0626 | 0.0003 | | 83 | 1 | 2 | * | 0.2435 | 0.4922 | 0.0808 | 0.1178 | 0.0503 | 0.0154 | | 84 | 1 | 1 |  | 0.6406 | 0.1261 | 0.0530 | 0.1087 | 0.0713 | 0.0004 | | 85 | 1 | 4 | * | 0.1108 | 0.1108 | 0.3242 | 0.4348 | 0.0194 | 0.0001 | | 86 | 1 | 2 | * | 0.1561 | 0.6205 | 0.0965 | 0.0901 | 0.0341 | 0.0027 | | 87 | 1 | 5 | * | 0.2508 | 0.0282 | 0.0452 | 0.0120 | 0.6593 | 0.0045 | | 88 | 1 | 3 | * | 0.0995 | 0.1332 | 0.4300 | 0.3213 | 0.0133 | 0.0027 | | 89 | 1 | 3 | * | 0.1255 | 0.0774 | 0.3384 | 0.2520 | 0.2064 | 0.0003 | | 90 | 1 | 1 |  | 0.3122 | 0.0938 | 0.0286 | 0.0264 | 0.2697 | 0.2693 | | 91 | 1 | 1 |  | 0.6214 | 0.0464 | 0.0523 | 0.0344 | 0.2437 | 0.0018 | | 92 | 1 | 3 | * | 0.2652 | 0.0539 | 0.5310 | 0.1264 | 0.0234 | 0.0000 | | 93 | 1 | 2 | * | 0.2514 | 0.2952 | 0.0488 | 0.2657 | 0.1383 | 0.0005 | | 94 | 1 | 5 | * | 0.3005 | 0.0683 | 0.0274 | 0.0363 | 0.4447 | 0.1228 | | 95 | 1 | 1 |  | 0.4974 | 0.0240 | 0.0349 | 0.0218 | 0.4201 | 0.0018 | | 96 | 1 | 5 | * | 0.3170 | 0.0436 | 0.0155 | 0.0114 | 0.5400 | 0.0725 | | 97 | 1 | 3 | * | 0.3870 | 0.0507 | 0.3994 | 0.1027 | 0.0601 | 0.0000 | | 98 | 1 | 4 | * | 0.2923 | 0.1346 | 0.0303 | 0.2969 | 0.2459 | 0.0000 | | 99 | 1 | 2 | * | 0.2455 | 0.4469 | 0.1008 | 0.1420 | 0.0520 | 0.0129 | | 100 | 1 | 1 |  | 0.6336 | 0.1376 | 0.0456 | 0.1096 | 0.0732 | 0.0005 | | 101 | 1 | 1 |  | 0.3072 | 0.0942 | 0.0274 | 0.0261 | 0.2676 | 0.2775 | | 102 | 1 | 1 |  | 0.5853 | 0.0420 | 0.0608 | 0.0455 | 0.2645 | 0.0020 | | 103 | 1 | 3 | * | 0.2308 | 0.0485 | 0.5410 | 0.1592 | 0.0204 | 0.0000 | | 104 | 1 | 3 | * | 0.2202 | 0.0733 | 0.3226 | 0.2878 | 0.0960 | 0.0001 | | 105 | 1 | 2 | * | 0.3436 | 0.4208 | 0.0489 | 0.0321 | 0.1540 | 0.0007 | | 106 | 1 | 5 | * | 0.2654 | 0.0222 | 0.0740 | 0.0096 | 0.6280 | 0.0007 | | 107 | 1 | 1 |  | 0.9317 | 0.0191 | 0.0346 | 0.0081 | 0.0065 | 0.0000 | | 108 | 1 | 1 |  | 0.7444 | 0.0186 | 0.0519 | 0.0299 | 0.1550 | 0.0002 | | 109 | 1 | 1 |  | 0.4002 | 0.1259 | 0.2690 | 0.1103 | 0.0945 | 0.0000 | | 110 | 1 | 2 | * | 0.2702 | 0.6561 | 0.0010 | 0.0064 | 0.0664 | 0.0000 | | 111 | 1 | 3 | * | 0.3225 | 0.1214 | 0.3829 | 0.1529 | 0.0202 | 0.0000 | | 112 | 1 | 4 | * | 0.2870 | 0.1419 | 0.0985 | 0.3867 | 0.0859 | 0.0000 | | 113 | 1 | 2 | * | 0.4019 | 0.4573 | 0.0576 | 0.0728 | 0.0104 | 0.0000 | | 114 | 1 | 3 | * | 0.3177 | 0.0984 | 0.3726 | 0.1913 | 0.0199 | 0.0000 | | 115 | 1 | 1 |  | 0.3863 | 0.2154 | 0.0282 | 0.0095 | 0.3461 | 0.0144 | | 116 | 1 | 2 | * | 0.2446 | 0.4736 | 0.0169 | 0.0152 | 0.2489 | 0.0009 | | 117 | 1 | 4 | * | 0.3604 | 0.0454 | 0.0905 | 0.4034 | 0.1003 | 0.0000 | | 118 | 1 | 1 |  | 0.4212 | 0.3385 | 0.0667 | 0.0602 | 0.1132 | 0.0001 | | 119 | 1 | 2 | * | 0.2846 | 0.3766 | 0.0139 | 0.0130 | 0.3116 | 0.0003 | | 120 | 1 | 1 |  | 0.8592 | 0.0149 | 0.0903 | 0.0241 | 0.0115 | 0.0000 | | 121 | 1 | 1 |  | 0.5080 | 0.0953 | 0.1960 | 0.0706 | 0.1301 | 0.0000 | | 122 | 1 | 2 | * | 0.3268 | 0.5028 | 0.0007 | 0.0043 | 0.1654 | 0.0000 | | 123 | 1 | 1 |  | 0.7992 | 0.0161 | 0.0342 | 0.0150 | 0.1354 | 0.0001 | | 124 | 1 | 1 |  | 0.4848 | 0.1152 | 0.0508 | 0.0866 | 0.2625 | 0.0000 | | 125 | 1 | 1 |  | 0.7443 | 0.0183 | 0.0534 | 0.0298 | 0.1540 | 0.0002 | | 126 | 1 | 1 |  | 0.4675 | 0.3677 | 0.0667 | 0.0753 | 0.0228 | 0.0000 | | 127 | 1 | 1 |  | 0.3764 | 0.0596 | 0.3409 | 0.1833 | 0.0399 | 0.0000 | | 128 | 1 | 1 |  | 0.4143 | 0.1520 | 0.0255 | 0.0129 | 0.3797 | 0.0157 | | 129 | 1 | 1 |  | 0.4967 | 0.0431 | 0.0906 | 0.2813 | 0.0883 | 0.0000 | | 130 | 1 | 1 |  | 0.4241 | 0.2411 | 0.0444 | 0.0449 | 0.2453 | 0.0002 | | 131 | 1 | 1 |  | 0.8720 | 0.0189 | 0.0747 | 0.0215 | 0.0129 | 0.0000 | | 132 | 1 | 1 |  | 0.7479 | 0.0238 | 0.0486 | 0.0196 | 0.1596 | 0.0004 | | 133 | 1 | 1 |  | 0.3482 | 0.1519 | 0.2967 | 0.1244 | 0.0789 | 0.0000 | | 134 | 1 | 2 | * | 0.2474 | 0.6894 | 0.0010 | 0.0063 | 0.0558 | 0.0000 | | 135 | 1 | 1 |  | 0.6762 | 0.2238 | 0.0733 | 0.0234 | 0.0033 | 0.0000 | | 136 | 1 | 1 |  | 0.4604 | 0.1890 | 0.0787 | 0.1571 | 0.1148 | 0.0000 | | 137 | 1 | 2 | * | 0.3961 | 0.4077 | 0.0753 | 0.1043 | 0.0166 | 0.0000 | | 138 | 1 | 1 |  | 0.7394 | 0.1778 | 0.0519 | 0.0209 | 0.0100 | 0.0000 | | 139 | 1 | 1 |  | 0.3817 | 0.1913 | 0.0265 | 0.0113 | 0.3777 | 0.0115 | | 140 | 1 | 2 | * | 0.2268 | 0.4891 | 0.0100 | 0.0107 | 0.2627 | 0.0008 | | 141 | 1 | 1 |  | 0.3873 | 0.2543 | 0.0528 | 0.0584 | 0.2471 | 0.0002 | | 142 | 1 | 1 |  | 0.9127 | 0.0267 | 0.0461 | 0.0106 | 0.0038 | 0.0000 | | 143 | 1 | 1 |  | 0.6438 | 0.0082 | 0.0958 | 0.1608 | 0.0786 | 0.0127 | | 144 | 1 | 4 | * | 0.2881 | 0.1370 | 0.1046 | 0.3854 | 0.0849 | 0.0000 | | 145 | 1 | 2 | * | 0.2179 | 0.7343 | 0.0009 | 0.0048 | 0.0422 | 0.0000 | | 146 | 1 | 1 |  | 0.5798 | 0.2739 | 0.0806 | 0.0620 | 0.0037 | 0.0000 | | 147 | 1 | 4 | * | 0.2392 | 0.2550 | 0.1192 | 0.3426 | 0.0441 | 0.0000 | | 148 | 1 | 2 | * | 0.3614 | 0.4919 | 0.0667 | 0.0702 | 0.0098 | 0.0000 | | 149 | 1 | 3 | * | 0.1798 | 0.1240 | 0.4397 | 0.2464 | 0.0103 | 0.0000 | | 150 | 1 | 5 | * | 0.3227 | 0.2499 | 0.0359 | 0.0155 | 0.3636 | 0.0125 | | 151 | 1 | 1 |  | 0.6776 | 0.1976 | 0.0787 | 0.0415 | 0.0045 | 0.0000 | | 152 | 1 | 2 | * | 0.1632 | 0.5349 | 0.0148 | 0.0142 | 0.2715 | 0.0015 | | 153 | 1 | 4 | * | 0.2165 | 0.0542 | 0.0817 | 0.5331 | 0.1144 | 0.0000 | | 154 | 1 | 2 | * | 0.3947 | 0.4540 | 0.0537 | 0.0585 | 0.0391 | 0.0000 | | 155 | 1 | 1 |  | 0.8749 | 0.0197 | 0.0706 | 0.0217 | 0.0131 | 0.0000 | | 156 | 1 | 1 |  | 0.7476 | 0.0247 | 0.0459 | 0.0198 | 0.1616 | 0.0004 | | 157 | 1 | 1 |  | 0.3588 | 0.1862 | 0.2363 | 0.1321 | 0.0867 | 0.0000 | | 158 | 1 | 1 |  | 0.6787 | 0.1941 | 0.0812 | 0.0415 | 0.0045 | 0.0000 | | 159 | 1 | 2 | * | 0.2394 | 0.6984 | 0.0009 | 0.0062 | 0.0551 | 0.0000 | | 160 | 1 | 1 |  | 0.6729 | 0.2314 | 0.0689 | 0.0234 | 0.0034 | 0.0000 | | 161 | 1 | 3 | * | 0.2385 | 0.1829 | 0.3724 | 0.1604 | 0.0459 | 0.0000 | | 162 | 1 | 1 |  | 0.3903 | 0.1116 | 0.3031 | 0.1059 | 0.0890 | 0.0000 | | 163 | 1 | 2 | * | 0.2786 | 0.6467 | 0.0011 | 0.0065 | 0.0672 | 0.0000 | | 164 | 1 | 1 |  | 0.4577 | 0.1953 | 0.0739 | 0.1573 | 0.1158 | 0.0000 | | 165 | 1 | 2 | * | 0.4080 | 0.4463 | 0.0619 | 0.0734 | 0.0104 | 0.0000 | | 166 | 1 | 3 | * | 0.3202 | 0.1011 | 0.3649 | 0.1934 | 0.0203 | 0.0000 | | 167 | 2 | 2 |  | 0.0267 | 0.4705 | 0.1550 | 0.3420 | 0.0050 | 0.0009 | | 168 | 2 | 2 |  | 0.1206 | 0.5350 | 0.2072 | 0.1334 | 0.0037 | 0.0001 | | 169 | 2 | 4 | * | 0.0631 | 0.0495 | 0.1004 | 0.7555 | 0.0275 | 0.0040 | | 170 | 2 | 4 | * | 0.1800 | 0.2462 | 0.2332 | 0.3118 | 0.0287 | 0.0001 | | 171 | 2 | 4 | * | 0.1203 | 0.1466 | 0.1298 | 0.5375 | 0.0615 | 0.0043 | | 172 | 2 | 2 |  | 0.1480 | 0.4520 | 0.2314 | 0.1333 | 0.0330 | 0.0022 | | 173 | 2 | 1 | * | 0.4427 | 0.1269 | 0.2253 | 0.0500 | 0.1551 | 0.0000 | | 174 | 2 | 3 | * | 0.1942 | 0.2160 | 0.2831 | 0.2675 | 0.0391 | 0.0001 | | 175 | 2 | 5 | * | 0.3212 | 0.1260 | 0.0231 | 0.0133 | 0.5165 | 0.0000 | | 176 | 2 | 2 |  | 0.0781 | 0.8683 | 0.0170 | 0.0196 | 0.0168 | 0.0001 | | 177 | 2 | 2 |  | 0.0464 | 0.5624 | 0.1369 | 0.2469 | 0.0061 | 0.0013 | | 178 | 2 | 2 |  | 0.1690 | 0.4988 | 0.1768 | 0.1441 | 0.0108 | 0.0005 | | 179 | 2 | 2 |  | 0.2855 | 0.4173 | 0.1636 | 0.0663 | 0.0671 | 0.0003 | | 180 | 2 | 2 |  | 0.1061 | 0.4458 | 0.0772 | 0.0906 | 0.2795 | 0.0008 | | 181 | 2 | 2 |  | 0.1372 | 0.3191 | 0.2536 | 0.2561 | 0.0340 | 0.0001 | | 182 | 2 | 4 | * | 0.1498 | 0.1455 | 0.1699 | 0.4646 | 0.0668 | 0.0033 | | 183 | 2 | 2 |  | 0.3684 | 0.3697 | 0.0915 | 0.0450 | 0.1245 | 0.0009 | | 184 | 2 | 1 | * | 0.4912 | 0.1165 | 0.1742 | 0.0300 | 0.1881 | 0.0000 | | 185 | 2 | 2 |  | 0.2187 | 0.5001 | 0.1754 | 0.0699 | 0.0349 | 0.0010 | | 186 | 2 | 2 |  | 0.1545 | 0.3683 | 0.3061 | 0.1338 | 0.0344 | 0.0029 | | 187 | 2 | 2 |  | 0.0900 | 0.8367 | 0.0169 | 0.0194 | 0.0365 | 0.0004 | | 188 | 2 | 2 |  | 0.2232 | 0.3899 | 0.2531 | 0.0761 | 0.0576 | 0.0001 | | 189 | 2 | 2 |  | 0.0238 | 0.4320 | 0.2001 | 0.3417 | 0.0023 | 0.0001 | | 190 | 2 | 2 |  | 0.1140 | 0.5686 | 0.1968 | 0.1182 | 0.0024 | 0.0000 | | 191 | 2 | 2 |  | 0.0815 | 0.6226 | 0.1072 | 0.1021 | 0.0866 | 0.0000 | | 192 | 2 | 5 | * | 0.3291 | 0.1095 | 0.0198 | 0.0086 | 0.5330 | 0.0000 | | 193 | 2 | 4 | * | 0.0569 | 0.0966 | 0.1453 | 0.6822 | 0.0185 | 0.0005 | | 194 | 2 | 2 |  | 0.1338 | 0.3246 | 0.2497 | 0.2758 | 0.0156 | 0.0004 | | 195 | 2 | 2 |  | 0.1051 | 0.4803 | 0.2521 | 0.1386 | 0.0229 | 0.0010 | | 196 | 2 | 1 | * | 0.3351 | 0.1333 | 0.2863 | 0.0772 | 0.1680 | 0.0000 | | 197 | 2 | 1 | * | 0.4189 | 0.2316 | 0.0259 | 0.0156 | 0.3081 | 0.0000 | | 198 | 2 | 2 |  | 0.0707 | 0.8952 | 0.0094 | 0.0113 | 0.0134 | 0.0001 | | 199 | 2 | 2 |  | 0.1032 | 0.5192 | 0.2147 | 0.1384 | 0.0233 | 0.0012 | | 200 | 2 | 2 |  | 0.0640 | 0.8097 | 0.0542 | 0.0711 | 0.0009 | 0.0002 | | 201 | 2 | 4 | * | 0.0712 | 0.0944 | 0.1362 | 0.6814 | 0.0113 | 0.0055 | | 202 | 2 | 4 | * | 0.0763 | 0.1396 | 0.1239 | 0.6426 | 0.0134 | 0.0042 | | 203 | 2 | 2 |  | 0.1480 | 0.4520 | 0.2314 | 0.1333 | 0.0330 | 0.0022 | | 204 | 2 | 2 |  | 0.1308 | 0.5547 | 0.1958 | 0.0957 | 0.0198 | 0.0032 | | 205 | 2 | 1 | * | 0.4347 | 0.2067 | 0.2577 | 0.0463 | 0.0545 | 0.0001 | | 206 | 2 | 1 | * | 0.4022 | 0.2204 | 0.0281 | 0.0208 | 0.3284 | 0.0000 | | 207 | 2 | 2 |  | 0.0506 | 0.9027 | 0.0146 | 0.0194 | 0.0121 | 0.0005 | | 208 | 2 | 2 |  | 0.0807 | 0.6285 | 0.1031 | 0.1014 | 0.0863 | 0.0000 | | 209 | 2 | 2 |  | 0.1197 | 0.3352 | 0.1881 | 0.3215 | 0.0354 | 0.0001 | | 210 | 2 | 4 | * | 0.0569 | 0.1002 | 0.1372 | 0.6864 | 0.0188 | 0.0005 | | 211 | 2 | 4 | * | 0.1800 | 0.2462 | 0.2332 | 0.3118 | 0.0287 | 0.0001 | | 212 | 2 | 2 |  | 0.0189 | 0.5424 | 0.1522 | 0.2852 | 0.0011 | 0.0003 | | 213 | 2 | 1 | * | 0.3388 | 0.1431 | 0.2657 | 0.0789 | 0.1735 | 0.0000 | | 214 | 2 | 1 | * | 0.3770 | 0.2442 | 0.0452 | 0.0265 | 0.3071 | 0.0000 | | 215 | 2 | 2 |  | 0.0708 | 0.8879 | 0.0107 | 0.0157 | 0.0149 | 0.0001 | | 216 | 2 | 2 |  | 0.2231 | 0.4707 | 0.2004 | 0.0703 | 0.0346 | 0.0009 | | 217 | 2 | 2 |  | 0.0192 | 0.5291 | 0.1633 | 0.2871 | 0.0011 | 0.0003 | | 218 | 2 | 4 | * | 0.1203 | 0.1466 | 0.1298 | 0.5375 | 0.0615 | 0.0043 | | 219 | 2 | 2 |  | 0.1293 | 0.5192 | 0.2211 | 0.1085 | 0.0176 | 0.0042 | | 220 | 2 | 1 | * | 0.4008 | 0.1815 | 0.2915 | 0.0622 | 0.0639 | 0.0000 | | 221 | 2 | 1 | * | 0.3963 | 0.2533 | 0.0476 | 0.0264 | 0.2764 | 0.0000 | | 222 | 2 | 2 |  | 0.0511 | 0.9067 | 0.0144 | 0.0176 | 0.0090 | 0.0011 | | 223 | 2 | 2 |  | 0.1204 | 0.5889 | 0.1941 | 0.0940 | 0.0026 | 0.0000 | | 224 | 2 | 2 |  | 0.0756 | 0.5672 | 0.1152 | 0.1386 | 0.1034 | 0.0000 | | 225 | 2 | 2 |  | 0.1232 | 0.4951 | 0.2441 | 0.1340 | 0.0037 | 0.0000 | | 226 | 2 | 4 | * | 0.0627 | 0.0541 | 0.0864 | 0.7636 | 0.0283 | 0.0050 | | 227 | 2 | 1 | * | 0.4427 | 0.1269 | 0.2253 | 0.0500 | 0.1551 | 0.0000 | | 228 | 2 | 1 | * | 0.3947 | 0.3387 | 0.0551 | 0.1844 | 0.0269 | 0.0002 | | 229 | 2 | 3 | * | 0.1222 | 0.1991 | 0.3164 | 0.2515 | 0.1098 | 0.0010 | | 230 | 2 | 2 |  | 0.0887 | 0.4404 | 0.1314 | 0.2300 | 0.0787 | 0.0309 | | 231 | 2 | 2 |  | 0.2779 | 0.5179 | 0.0643 | 0.1281 | 0.0118 | 0.0000 | | 232 | 2 | 1 | * | 0.3285 | 0.1380 | 0.0521 | 0.2749 | 0.1470 | 0.0595 | | 233 | 2 | 2 |  | 0.2538 | 0.5910 | 0.0750 | 0.0251 | 0.0545 | 0.0006 | | 234 | 2 | 2 |  | 0.0381 | 0.9025 | 0.0152 | 0.0299 | 0.0142 | 0.0001 | | 235 | 2 | 4 | * | 0.2986 | 0.1189 | 0.1003 | 0.4151 | 0.0669 | 0.0003 | | 236 | 2 | 2 |  | 0.2896 | 0.3737 | 0.0763 | 0.0394 | 0.2210 | 0.0000 | | 237 | 2 | 2 |  | 0.2512 | 0.5967 | 0.0722 | 0.0249 | 0.0543 | 0.0006 | | 238 | 2 | 2 |  | 0.1978 | 0.6516 | 0.0793 | 0.0394 | 0.0316 | 0.0003 | | 239 | 2 | 6 | * | 0.0534 | 0.3180 | 0.0519 | 0.1162 | 0.0302 | 0.4304 | | 240 | 2 | 2 |  | 0.0970 | 0.7400 | 0.0895 | 0.0473 | 0.0258 | 0.0003 | | 241 | 2 | 2 |  | 0.3059 | 0.3815 | 0.0755 | 0.0296 | 0.2075 | 0.0000 | | 242 | 2 | 2 |  | 0.1385 | 0.6925 | 0.0284 | 0.0787 | 0.0602 | 0.0017 | | 243 | 2 | 2 |  | 0.1736 | 0.5327 | 0.0668 | 0.0254 | 0.2012 | 0.0003 | | 244 | 2 | 1 | * | 0.3562 | 0.3317 | 0.0487 | 0.2353 | 0.0280 | 0.0002 | | 245 | 2 | 2 |  | 0.0769 | 0.4150 | 0.1117 | 0.2821 | 0.0785 | 0.0359 | | 246 | 2 | 4 | * | 0.2996 | 0.1127 | 0.1096 | 0.4122 | 0.0657 | 0.0002 | | 247 | 2 | 2 |  | 0.2365 | 0.4749 | 0.0606 | 0.2151 | 0.0129 | 0.0000 | | 248 | 2 | 4 | * | 0.2813 | 0.1275 | 0.0437 | 0.3338 | 0.1450 | 0.0687 | | 249 | 2 | 2 |  | 0.0348 | 0.8978 | 0.0136 | 0.0387 | 0.0150 | 0.0001 | | 250 | 2 | 4 | * | 0.2506 | 0.1074 | 0.0824 | 0.4946 | 0.0647 | 0.0003 | | 251 | 2 | 2 |  | 0.2672 | 0.3778 | 0.0691 | 0.0514 | 0.2345 | 0.0000 | | 252 | 2 | 2 |  | 0.2339 | 0.6083 | 0.0659 | 0.0329 | 0.0582 | 0.0007 | | 253 | 2 | 2 |  | 0.1823 | 0.6605 | 0.0717 | 0.0515 | 0.0336 | 0.0004 | | 254 | 2 | 6 | * | 0.0351 | 0.2511 | 0.0431 | 0.1352 | 0.0250 | 0.5105 | | 255 | 2 | 4 | * | 0.1079 | 0.1916 | 0.2739 | 0.3140 | 0.1116 | 0.0011 | | 256 | 2 | 2 |  | 0.0866 | 0.7114 | 0.0886 | 0.0836 | 0.0294 | 0.0004 | | 257 | 2 | 2 |  | 0.1100 | 0.6172 | 0.0982 | 0.1465 | 0.0272 | 0.0009 | | 258 | 2 | 2 |  | 0.1310 | 0.6702 | 0.0286 | 0.1042 | 0.0642 | 0.0019 | | 259 | 2 | 2 |  | 0.1587 | 0.5419 | 0.0602 | 0.0334 | 0.2056 | 0.0003 | | 260 | 2 | 4 | * | 0.2511 | 0.1056 | 0.0850 | 0.4937 | 0.0643 | 0.0003 | | 261 | 2 | 2 |  | 0.2412 | 0.5917 | 0.0740 | 0.0336 | 0.0588 | 0.0007 | | 262 | 2 | 2 |  | 0.2134 | 0.5799 | 0.1224 | 0.0502 | 0.0338 | 0.0002 | | 263 | 2 | 2 |  | 0.1219 | 0.6256 | 0.1109 | 0.1146 | 0.0262 | 0.0007 | | 264 | 2 | 6 | * | 0.0552 | 0.2953 | 0.0698 | 0.1640 | 0.0334 | 0.3824 | | 265 | 2 | 3 | * | 0.1226 | 0.2038 | 0.3085 | 0.2531 | 0.1110 | 0.0010 | | 266 | 2 | 2 |  | 0.1003 | 0.7384 | 0.0933 | 0.0426 | 0.0252 | 0.0003 | | 267 | 2 | 2 |  | 0.1188 | 0.5628 | 0.1369 | 0.1533 | 0.0276 | 0.0007 | | 268 | 2 | 2 |  | 0.1023 | 0.7240 | 0.0239 | 0.0803 | 0.0688 | 0.0007 | | 269 | 2 | 2 |  | 0.1776 | 0.5904 | 0.0503 | 0.0233 | 0.1583 | 0.0001 | | 270 | 2 | 1 | * | 0.3264 | 0.2947 | 0.0518 | 0.2987 | 0.0282 | 0.0002 | | 271 | 2 | 1 | * | 0.3597 | 0.3047 | 0.0581 | 0.2494 | 0.0278 | 0.0002 | | 272 | 2 | 3 | * | 0.1230 | 0.2085 | 0.3007 | 0.2547 | 0.1121 | 0.0011 | | 273 | 2 | 2 |  | 0.0982 | 0.7345 | 0.0933 | 0.0478 | 0.0259 | 0.0003 | | 274 | 2 | 2 |  | 0.1252 | 0.6073 | 0.1241 | 0.1165 | 0.0263 | 0.0007 | | 275 | 2 | 2 |  | 0.1356 | 0.7058 | 0.0256 | 0.0726 | 0.0586 | 0.0018 | | 276 | 2 | 2 |  | 0.1673 | 0.5099 | 0.0729 | 0.0343 | 0.2153 | 0.0003 | | 277 | 2 | 6 | * | 0.0560 | 0.3211 | 0.0576 | 0.1210 | 0.0312 | 0.4130 | | 278 | 2 | 2 |  | 0.2803 | 0.5124 | 0.0668 | 0.1287 | 0.0119 | 0.0000 | | 279 | 2 | 1 | * | 0.3303 | 0.1364 | 0.0540 | 0.2755 | 0.1467 | 0.0572 | | 280 | 2 | 2 |  | 0.2936 | 0.3640 | 0.0819 | 0.0396 | 0.2209 | 0.0000 | | 281 | 2 | 2 |  | 0.0394 | 0.8987 | 0.0167 | 0.0307 | 0.0145 | 0.0001 | | 282 | 2 | 2 |  | 0.2060 | 0.6287 | 0.0925 | 0.0405 | 0.0320 | 0.0003 | | 283 | 2 | 4 | * | 0.0513 | 0.1474 | 0.2491 | 0.5354 | 0.0169 | 0.0000 | | 284 | 2 | 1 | * | 0.3749 | 0.2901 | 0.0682 | 0.2393 | 0.0274 | 0.0001 | | 285 | 2 | 2 |  | 0.0917 | 0.4143 | 0.1565 | 0.2336 | 0.0784 | 0.0256 | | 286 | 2 | 2 |  | 0.2827 | 0.5068 | 0.0693 | 0.1294 | 0.0119 | 0.0000 | | 287 | 2 | 1 | * | 0.4828 | 0.2773 | 0.1340 | 0.0186 | 0.0864 | 0.0009 | | 288 | 2 | 1 | * | 0.3371 | 0.1296 | 0.0618 | 0.2773 | 0.1454 | 0.0488 | | 289 | 2 | 2 |  | 0.0408 | 0.8946 | 0.0183 | 0.0315 | 0.0148 | 0.0001 | | 290 | 2 | 4 | * | 0.2480 | 0.1166 | 0.0706 | 0.4981 | 0.0663 | 0.0004 | | 291 | 2 | 2 |  | 0.2571 | 0.4010 | 0.0577 | 0.0504 | 0.2338 | 0.0000 | | 292 | 2 | 2 |  | 0.1039 | 0.3575 | 0.2991 | 0.2260 | 0.0135 | 0.0000 | | 293 | 2 | 2 |  | 0.2251 | 0.6382 | 0.0520 | 0.0314 | 0.0527 | 0.0006 | | 294 | 2 | 2 |  | 0.1823 | 0.6605 | 0.0717 | 0.0515 | 0.0336 | 0.0004 | | 295 | 2 | 6 | * | 0.0470 | 0.3077 | 0.0396 | 0.1041 | 0.0276 | 0.4739 | | 296 | 2 | 4 | * | 0.1244 | 0.2472 | 0.2422 | 0.2646 | 0.1201 | 0.0015 | | 297 | 2 | 2 |  | 0.0850 | 0.7885 | 0.0589 | 0.0429 | 0.0243 | 0.0004 | | 298 | 2 | 2 |  | 0.1161 | 0.6545 | 0.0917 | 0.1111 | 0.0259 | 0.0009 | | 299 | 2 | 2 |  | 0.1310 | 0.6702 | 0.0286 | 0.1042 | 0.0642 | 0.0019 | | 300 | 2 | 2 |  | 0.1504 | 0.5600 | 0.0492 | 0.0319 | 0.2081 | 0.0004 | | 301 | 2 | 2 |  | 0.1086 | 0.4153 | 0.0609 | 0.0449 | 0.2493 | 0.1211 | | 302 | 2 | 4 | * | 0.0583 | 0.0553 | 0.3431 | 0.5185 | 0.0244 | 0.0004 | | 303 | 2 | 2 |  | 0.2529 | 0.4102 | 0.0536 | 0.0499 | 0.2334 | 0.0000 | | 304 | 2 | 1 | * | 0.2521 | 0.2237 | 0.1981 | 0.2372 | 0.0889 | 0.0000 | | 305 | 2 | 5 | * | 0.2380 | 0.1060 | 0.0479 | 0.0132 | 0.5754 | 0.0195 | | 306 | 2 | 2 |  | 0.0927 | 0.4034 | 0.1674 | 0.2346 | 0.0781 | 0.0237 | | 307 | 2 | 1 | * | 0.6247 | 0.2137 | 0.0689 | 0.0419 | 0.0508 | 0.0000 | | 308 | 2 | 2 |  | 0.1875 | 0.4678 | 0.1423 | 0.0777 | 0.1246 | 0.0002 | | 309 | 2 | 4 | * | 0.0517 | 0.1638 | 0.2179 | 0.5490 | 0.0176 | 0.0000 | | 310 | 2 | 4 | * | 0.2673 | 0.1192 | 0.0734 | 0.4712 | 0.0686 | 0.0004 | | 311 | 2 | 2 |  | 0.0415 | 0.8925 | 0.0191 | 0.0319 | 0.0149 | 0.0001 | | 312 | 2 | 2 |  | 0.2214 | 0.6347 | 0.0541 | 0.0317 | 0.0572 | 0.0009 | | 313 | 2 | 6 | * | 0.0435 | 0.2816 | 0.0415 | 0.1340 | 0.0283 | 0.4711 | | 314 | 2 | 2 |  | 0.2289 | 0.6191 | 0.0609 | 0.0325 | 0.0578 | 0.0008 | | 315 | 2 | 2 |  | 0.1219 | 0.6256 | 0.1109 | 0.1146 | 0.0262 | 0.0007 | | 316 | 2 | 2 |  | 0.0803 | 0.8049 | 0.0496 | 0.0411 | 0.0236 | 0.0004 | | 317 | 2 | 2 |  | 0.1013 | 0.7330 | 0.0794 | 0.0633 | 0.0225 | 0.0005 | | 318 | 2 | 2 |  | 0.0876 | 0.7890 | 0.0529 | 0.0402 | 0.0298 | 0.0006 | | 319 | 2 | 2 |  | 0.1429 | 0.5037 | 0.0575 | 0.0443 | 0.2513 | 0.0002 | | 320 | 2 | 2 |  | 0.1142 | 0.4390 | 0.0566 | 0.0339 | 0.2367 | 0.1195 | | 321 | 2 | 2 |  | 0.2873 | 0.4956 | 0.0746 | 0.1306 | 0.0119 | 0.0000 | | 322 | 2 | 1 | * | 0.3387 | 0.1279 | 0.0639 | 0.2776 | 0.1450 | 0.0469 | | 323 | 2 | 2 |  | 0.1326 | 0.6661 | 0.0298 | 0.1052 | 0.0645 | 0.0018 | | 324 | 2 | 4 | * | 0.2687 | 0.1161 | 0.0841 | 0.4642 | 0.0665 | 0.0003 | | 325 | 2 | 2 |  | 0.2612 | 0.3918 | 0.0620 | 0.0508 | 0.2342 | 0.0000 | | 326 | 3 | 2 | * | 0.1934 | 0.4438 | 0.1585 | 0.1609 | 0.0433 | 0.0000 | | 327 | 3 | 4 | * | 0.0640 | 0.0207 | 0.4308 | 0.4815 | 0.0030 | 0.0000 | | 328 | 3 | 2 | * | 0.2206 | 0.3838 | 0.1940 | 0.1630 | 0.0387 | 0.0000 | | 329 | 3 | 4 | * | 0.0512 | 0.2568 | 0.2477 | 0.3352 | 0.1034 | 0.0056 | | 330 | 3 | 2 | * | 0.1079 | 0.3925 | 0.2113 | 0.2542 | 0.0333 | 0.0009 | | 331 | 3 | 1 | * | 0.5029 | 0.2075 | 0.1388 | 0.0534 | 0.0966 | 0.0009 | | 332 | 3 | 5 | * | 0.1161 | 0.0811 | 0.2426 | 0.2633 | 0.2801 | 0.0167 | | 333 | 3 | 3 |  | 0.0522 | 0.0551 | 0.4868 | 0.4030 | 0.0030 | 0.0000 | | 334 | 3 | 2 | * | 0.1752 | 0.4301 | 0.1446 | 0.2053 | 0.0448 | 0.0000 | | 335 | 3 | 4 | * | 0.0539 | 0.0188 | 0.3543 | 0.5701 | 0.0030 | 0.0000 | | 336 | 3 | 4 | * | 0.0436 | 0.2388 | 0.2057 | 0.4041 | 0.1014 | 0.0064 | | 337 | 3 | 2 | * | 0.0975 | 0.3789 | 0.1781 | 0.3106 | 0.0340 | 0.0010 | | 338 | 3 | 1 | * | 0.4776 | 0.2149 | 0.1290 | 0.0718 | 0.1055 | 0.0011 | | 339 | 3 | 4 | * | 0.1001 | 0.0763 | 0.2043 | 0.3215 | 0.2784 | 0.0193 | | 340 | 3 | 3 |  | 0.0678 | 0.0636 | 0.6018 | 0.2639 | 0.0029 | 0.0000 | | 341 | 3 | 2 | * | 0.1770 | 0.3953 | 0.1462 | 0.2386 | 0.0428 | 0.0000 | | 342 | 3 | 4 | * | 0.0809 | 0.0282 | 0.4434 | 0.4442 | 0.0034 | 0.0000 | | 343 | 3 | 2 | * | 0.0668 | 0.3153 | 0.2839 | 0.2225 | 0.1062 | 0.0053 | | 344 | 3 | 2 | * | 0.1284 | 0.4116 | 0.2307 | 0.1954 | 0.0331 | 0.0007 | | 345 | 3 | 1 | * | 0.5241 | 0.2140 | 0.1313 | 0.0398 | 0.0900 | 0.0008 | | 346 | 3 | 5 | * | 0.1329 | 0.0902 | 0.2605 | 0.2153 | 0.2848 | 0.0163 | | 347 | 3 | 4 | * | 0.0638 | 0.0207 | 0.4295 | 0.4830 | 0.0029 | 0.0000 | | 348 | 3 | 4 | * | 0.0512 | 0.2568 | 0.2477 | 0.3352 | 0.1034 | 0.0056 | | 349 | 3 | 3 |  | 0.0516 | 0.0534 | 0.4950 | 0.3971 | 0.0029 | 0.0000 | | 350 | 3 | 2 | * | 0.1146 | 0.4007 | 0.2028 | 0.2470 | 0.0341 | 0.0008 | | 351 | 3 | 1 | * | 0.5029 | 0.2075 | 0.1388 | 0.0534 | 0.0966 | 0.0009 | | 352 | 3 | 5 | * | 0.1161 | 0.0811 | 0.2426 | 0.2633 | 0.2801 | 0.0167 | | 353 | 3 | 3 |  | 0.0522 | 0.0551 | 0.4868 | 0.4030 | 0.0030 | 0.0000 | | 354 | 3 | 2 | * | 0.1743 | 0.4362 | 0.1398 | 0.2049 | 0.0449 | 0.0000 | | 355 | 3 | 4 | * | 0.1070 | 0.0802 | 0.1986 | 0.3040 | 0.2896 | 0.0205 | | 356 | 3 | 4 | * | 0.0512 | 0.2421 | 0.2694 | 0.3314 | 0.1011 | 0.0049 | | 357 | 3 | 4 | * | 0.1137 | 0.0695 | 0.2626 | 0.2796 | 0.2576 | 0.0170 | | 358 | 3 | 2 | * | 0.1019 | 0.3708 | 0.2340 | 0.2604 | 0.0321 | 0.0008 | | 359 | 3 | 1 | * | 0.5034 | 0.2037 | 0.1429 | 0.0532 | 0.0960 | 0.0008 | | 360 | 3 | 3 |  | 0.2207 | 0.0235 | 0.3848 | 0.1853 | 0.1857 | 0.0000 | | 361 | 3 | 2 | * | 0.0105 | 0.4578 | 0.1707 | 0.3558 | 0.0051 | 0.0000 | | 362 | 3 | 2 | * | 0.0390 | 0.4566 | 0.0991 | 0.3545 | 0.0508 | 0.0001 | | 363 | 3 | 5 | * | 0.2184 | 0.0214 | 0.2390 | 0.1019 | 0.4193 | 0.0000 | | 364 | 3 | 4 | * | 0.0122 | 0.0086 | 0.3648 | 0.6002 | 0.0142 | 0.0000 | | 365 | 3 | 3 |  | 0.0846 | 0.0858 | 0.6470 | 0.1405 | 0.0421 | 0.0000 | | 366 | 3 | 4 | * | 0.1277 | 0.0412 | 0.1361 | 0.6725 | 0.0224 | 0.0000 | | 367 | 3 | 1 | * | 0.6229 | 0.1949 | 0.0969 | 0.0738 | 0.0115 | 0.0000 | | 368 | 3 | 3 |  | 0.1649 | 0.0232 | 0.5140 | 0.2120 | 0.0858 | 0.0000 | | 369 | 3 | 2 | * | 0.0070 | 0.4527 | 0.1701 | 0.3662 | 0.0040 | 0.0000 | | 370 | 3 | 4 | * | 0.0057 | 0.0053 | 0.3132 | 0.6703 | 0.0055 | 0.0000 | | 371 | 3 | 2 | * | 0.0272 | 0.4514 | 0.1140 | 0.3851 | 0.0223 | 0.0000 | | 372 | 3 | 4 | * | 0.0081 | 0.0073 | 0.3582 | 0.6196 | 0.0068 | 0.0000 | | 373 | 3 | 3 |  | 0.0552 | 0.0761 | 0.7027 | 0.1492 | 0.0169 | 0.0000 | | 374 | 3 | 4 | * | 0.0860 | 0.0379 | 0.1445 | 0.7207 | 0.0109 | 0.0000 | | 375 | 3 | 2 | * | 0.0475 | 0.4943 | 0.0793 | 0.2379 | 0.1405 | 0.0005 | | 376 | 3 | 1 | * | 0.5200 | 0.2670 | 0.1188 | 0.0821 | 0.0121 | 0.0000 | | 377 | 3 | 5 | * | 0.2184 | 0.0214 | 0.2390 | 0.1019 | 0.4193 | 0.0000 | | 378 | 3 | 2 | * | 0.0143 | 0.5381 | 0.1673 | 0.2698 | 0.0104 | 0.0001 | | 379 | 3 | 3 |  | 0.0539 | 0.0748 | 0.7061 | 0.1479 | 0.0173 | 0.0000 | | 380 | 3 | 2 | * | 0.0475 | 0.4943 | 0.0793 | 0.2379 | 0.1405 | 0.0005 | | 381 | 3 | 4 | * | 0.0183 | 0.0111 | 0.4024 | 0.5356 | 0.0324 | 0.0001 | | 382 | 3 | 3 |  | 0.1115 | 0.1001 | 0.5607 | 0.1016 | 0.1259 | 0.0001 | | 383 | 3 | 4 | * | 0.1902 | 0.0538 | 0.1406 | 0.5540 | 0.0613 | 0.0000 | | 384 | 3 | 1 | * | 0.6778 | 0.1832 | 0.0755 | 0.0447 | 0.0187 | 0.0000 | | 385 | 3 | 3 |  | 0.0846 | 0.0858 | 0.6470 | 0.1405 | 0.0421 | 0.0000 | | 386 | 3 | 4 | * | 0.1277 | 0.0412 | 0.1361 | 0.6725 | 0.0224 | 0.0000 | | 387 | 3 | 1 | * | 0.6229 | 0.1949 | 0.0969 | 0.0738 | 0.0115 | 0.0000 | | 388 | 3 | 3 |  | 0.1649 | 0.0232 | 0.5140 | 0.2120 | 0.0858 | 0.0000 | | 389 | 3 | 2 | * | 0.0070 | 0.4527 | 0.1701 | 0.3662 | 0.0040 | 0.0000 | | 390 | 3 | 1 | * | 0.4975 | 0.3167 | 0.1086 | 0.0635 | 0.0136 | 0.0000 | | 391 | 3 | 4 | * | 0.0045 | 0.0046 | 0.3189 | 0.6671 | 0.0048 | 0.0000 | | 392 | 3 | 3 |  | 0.0552 | 0.0761 | 0.7027 | 0.1492 | 0.0169 | 0.0000 | | 393 | 3 | 4 | * | 0.0660 | 0.0288 | 0.1243 | 0.7716 | 0.0093 | 0.0000 | | 394 | 3 | 1 | * | 0.5067 | 0.2933 | 0.1161 | 0.0707 | 0.0132 | 0.0000 | | 395 | 3 | 3 |  | 0.0118 | 0.0124 | 0.5159 | 0.4518 | 0.0082 | 0.0000 | | 396 | 3 | 3 |  | 0.0523 | 0.0681 | 0.7237 | 0.1402 | 0.0157 | 0.0000 | | 397 | 3 | 4 | * | 0.0859 | 0.0372 | 0.1486 | 0.7175 | 0.0108 | 0.0000 | | 398 | 3 | 3 |  | 0.1110 | 0.0960 | 0.5642 | 0.1060 | 0.1227 | 0.0001 | | 399 | 3 | 1 | * | 0.5326 | 0.2380 | 0.1187 | 0.0998 | 0.0109 | 0.0000 | | 400 | 3 | 5 | * | 0.2195 | 0.0224 | 0.2273 | 0.1032 | 0.4275 | 0.0001 | | 401 | 3 | 2 | * | 0.0125 | 0.4942 | 0.1511 | 0.3318 | 0.0103 | 0.0001 | | 402 | 3 | 4 | * | 0.1858 | 0.0506 | 0.1383 | 0.5666 | 0.0587 | 0.0000 | | 403 | 3 | 2 | * | 0.0272 | 0.4514 | 0.1140 | 0.3851 | 0.0223 | 0.0000 | | 404 | 3 | 4 | * | 0.0180 | 0.0105 | 0.4177 | 0.5224 | 0.0314 | 0.0001 | | 405 | 3 | 3 |  | 0.1101 | 0.0970 | 0.5692 | 0.1000 | 0.1235 | 0.0001 | | 406 | 3 | 4 | * | 0.1904 | 0.0560 | 0.1327 | 0.5585 | 0.0623 | 0.0000 | | 407 | 3 | 1 | * | 0.6715 | 0.1998 | 0.0645 | 0.0450 | 0.0192 | 0.0000 | | 408 | 3 | 2 | * | 0.0272 | 0.4514 | 0.1140 | 0.3851 | 0.0223 | 0.0000 | | 409 | 3 | 4 | * | 0.0073 | 0.0067 | 0.3681 | 0.6116 | 0.0063 | 0.0000 | | 410 | 3 | 3 |  | 0.0493 | 0.0621 | 0.7403 | 0.1330 | 0.0153 | 0.0000 | | 411 | 3 | 3 |  | 0.0666 | 0.1451 | 0.4160 | 0.3141 | 0.0573 | 0.0008 | | 412 | 3 | 1 | * | 0.4649 | 0.0179 | 0.1238 | 0.0412 | 0.3518 | 0.0003 | | 413 | 3 | 3 |  | 0.0436 | 0.0533 | 0.4747 | 0.3680 | 0.0354 | 0.0249 | | 414 | 3 | 3 |  | 0.0412 | 0.0595 | 0.5314 | 0.2900 | 0.0430 | 0.0349 | | 415 | 3 | 1 | * | 0.4179 | 0.0109 | 0.1264 | 0.0517 | 0.3919 | 0.0012 | | 416 | 3 | 3 |  | 0.0577 | 0.1517 | 0.4061 | 0.3339 | 0.0503 | 0.0004 | | 417 | 3 | 1 | * | 0.5417 | 0.0726 | 0.3120 | 0.0491 | 0.0246 | 0.0000 | | 418 | 3 | 3 |  | 0.0341 | 0.0572 | 0.5146 | 0.3377 | 0.0354 | 0.0210 | | 419 | 3 | 1 | * | 0.4258 | 0.0156 | 0.1475 | 0.0545 | 0.3564 | 0.0003 | | 420 | 3 | 3 |  | 0.1419 | 0.2187 | 0.2966 | 0.2194 | 0.1231 | 0.0004 | | 421 | 3 | 2 | * | 0.0823 | 0.4239 | 0.2614 | 0.2157 | 0.0157 | 0.0011 | | 422 | 3 | 3 |  | 0.0164 | 0.0411 | 0.4855 | 0.4401 | 0.0115 | 0.0054 | | 423 | 3 | 3 |  | 0.0481 | 0.1389 | 0.3993 | 0.3731 | 0.0404 | 0.0002 | | 424 | 3 | 1 | * | 0.4616 | 0.0729 | 0.3737 | 0.0681 | 0.0237 | 0.0000 | | 425 | 3 | 2 | * | 0.0887 | 0.3754 | 0.2753 | 0.2403 | 0.0184 | 0.0019 | | 426 | 3 | 3 |  | 0.0305 | 0.0584 | 0.4980 | 0.3715 | 0.0309 | 0.0107 | | 427 | 3 | 1 | * | 0.3867 | 0.0149 | 0.1724 | 0.0694 | 0.3565 | 0.0001 | | 428 | 3 | 3 |  | 0.1687 | 0.2254 | 0.2668 | 0.2226 | 0.1162 | 0.0002 | | 429 | 3 | 2 | * | 0.0629 | 0.3390 | 0.2807 | 0.3043 | 0.0126 | 0.0005 | | 430 | 3 | 3 |  | 0.0568 | 0.0673 | 0.4938 | 0.3151 | 0.0401 | 0.0269 | | 431 | 3 | 1 | * | 0.4364 | 0.0142 | 0.1310 | 0.0413 | 0.3765 | 0.0005 | | 432 | 3 | 3 |  | 0.0789 | 0.1370 | 0.4281 | 0.2891 | 0.0654 | 0.0015 | | 433 | 3 | 1 | * | 0.6597 | 0.0690 | 0.2204 | 0.0233 | 0.0276 | 0.0001 | | 434 | 3 | 3 |  | 0.0391 | 0.0465 | 0.4953 | 0.3004 | 0.0431 | 0.0756 | | 435 | 3 | 5 | * | 0.3597 | 0.0103 | 0.1126 | 0.0391 | 0.4780 | 0.0003 | | 436 | 3 | 3 |  | 0.1644 | 0.2008 | 0.2879 | 0.1787 | 0.1673 | 0.0010 | | 437 | 3 | 2 | * | 0.1062 | 0.3386 | 0.2974 | 0.2333 | 0.0207 | 0.0038 | | 438 | 3 | 3 |  | 0.1422 | 0.1980 | 0.2994 | 0.2168 | 0.1430 | 0.0006 | | 439 | 3 | 3 |  | 0.0981 | 0.1435 | 0.4320 | 0.2437 | 0.0802 | 0.0024 | | 440 | 3 | 1 | * | 0.7072 | 0.0682 | 0.1788 | 0.0153 | 0.0305 | 0.0001 | | 441 | 3 | 3 |  | 0.0469 | 0.0516 | 0.4836 | 0.2555 | 0.0559 | 0.1065 | | 442 | 3 | 5 | * | 0.4021 | 0.0093 | 0.0907 | 0.0290 | 0.4671 | 0.0018 | | 443 | 3 | 3 |  | 0.1905 | 0.1987 | 0.2414 | 0.1343 | 0.2329 | 0.0023 | | 444 | 3 | 2 | * | 0.1169 | 0.3491 | 0.2794 | 0.2229 | 0.0263 | 0.0053 | | 445 | 3 | 3 |  | 0.0544 | 0.3151 | 0.3212 | 0.2952 | 0.0134 | 0.0007 | | 446 | 3 | 3 |  | 0.0784 | 0.0465 | 0.4673 | 0.1541 | 0.0767 | 0.1770 | | 447 | 3 | 1 | * | 0.6131 | 0.0722 | 0.2587 | 0.0307 | 0.0252 | 0.0000 | | 448 | 3 | 3 |  | 0.0268 | 0.0543 | 0.5072 | 0.3720 | 0.0286 | 0.0112 | | 449 | 3 | 3 |  | 0.1423 | 0.1945 | 0.2937 | 0.2278 | 0.1411 | 0.0007 | | 450 | 3 | 3 |  | 0.0620 | 0.1109 | 0.4210 | 0.3456 | 0.0591 | 0.0014 | | 451 | 3 | 5 | * | 0.4082 | 0.0125 | 0.1200 | 0.0421 | 0.4164 | 0.0008 | | 452 | 3 | 3 |  | 0.1734 | 0.2354 | 0.2720 | 0.1496 | 0.1684 | 0.0012 | | 453 | 3 | 3 |  | 0.1004 | 0.1556 | 0.4053 | 0.2520 | 0.0839 | 0.0028 | | 454 | 3 | 1 | * | 0.6751 | 0.0578 | 0.2138 | 0.0224 | 0.0307 | 0.0001 | | 455 | 3 | 1 | * | 0.5487 | 0.0707 | 0.3089 | 0.0474 | 0.0243 | 0.0000 | | 456 | 3 | 3 |  | 0.0273 | 0.0503 | 0.4872 | 0.3799 | 0.0324 | 0.0229 | | 457 | 4 | 4 |  | 0.2896 | 0.1119 | 0.0801 | 0.4205 | 0.0974 | 0.0004 | | 458 | 4 | 4 |  | 0.0252 | 0.0837 | 0.1738 | 0.6744 | 0.0418 | 0.0011 | | 459 | 4 | 4 |  | 0.0046 | 0.0035 | 0.3778 | 0.6100 | 0.0041 | 0.0000 | | 460 | 4 | 4 |  | 0.0098 | 0.0885 | 0.0819 | 0.7787 | 0.0116 | 0.0297 | | 461 | 4 | 4 |  | 0.0252 | 0.0837 | 0.1738 | 0.6744 | 0.0418 | 0.0011 | | 462 | 4 | 1 | * | 0.4081 | 0.0826 | 0.2886 | 0.2073 | 0.0134 | 0.0000 | | 463 | 4 | 2 | * | 0.2025 | 0.4734 | 0.0559 | 0.1971 | 0.0703 | 0.0008 | | 464 | 4 | 4 |  | 0.0163 | 0.0152 | 0.2100 | 0.7423 | 0.0162 | 0.0000 | | 465 | 4 | 4 |  | 0.2985 | 0.1120 | 0.0762 | 0.3788 | 0.1340 | 0.0004 | | 466 | 4 | 4 |  | 0.0242 | 0.0195 | 0.2618 | 0.6606 | 0.0339 | 0.0000 | | 467 | 4 | 4 |  | 0.0052 | 0.0038 | 0.3889 | 0.5959 | 0.0062 | 0.0000 | | 468 | 4 | 4 |  | 0.0103 | 0.0851 | 0.0842 | 0.7775 | 0.0165 | 0.0264 | | 469 | 4 | 4 |  | 0.0318 | 0.1010 | 0.1931 | 0.6073 | 0.0657 | 0.0012 | | 470 | 4 | 1 | * | 0.4255 | 0.0820 | 0.2855 | 0.1884 | 0.0186 | 0.0000 | | 471 | 4 | 2 | * | 0.2129 | 0.4658 | 0.0561 | 0.1692 | 0.0954 | 0.0007 | | 472 | 4 | 4 |  | 0.3294 | 0.1021 | 0.0738 | 0.3332 | 0.1599 | 0.0017 | | 473 | 4 | 4 |  | 0.0255 | 0.0192 | 0.2877 | 0.6038 | 0.0633 | 0.0005 | | 474 | 4 | 2 | * | 0.1583 | 0.6121 | 0.0525 | 0.1517 | 0.0219 | 0.0036 | | 475 | 4 | 4 |  | 0.0062 | 0.0038 | 0.4091 | 0.5728 | 0.0081 | 0.0000 | | 476 | 4 | 4 |  | 0.0191 | 0.1409 | 0.1012 | 0.5483 | 0.0265 | 0.1640 | | 477 | 4 | 4 |  | 0.0487 | 0.1222 | 0.2163 | 0.5148 | 0.0907 | 0.0072 | | 478 | 4 | 1 | * | 0.4676 | 0.0759 | 0.2677 | 0.1665 | 0.0223 | 0.0000 | | 479 | 4 | 2 | * | 0.2331 | 0.4366 | 0.0521 | 0.1584 | 0.1170 | 0.0029 | | 480 | 4 | 4 |  | 0.2403 | 0.0961 | 0.0771 | 0.5110 | 0.0751 | 0.0003 | | 481 | 4 | 4 |  | 0.0046 | 0.0035 | 0.3778 | 0.6100 | 0.0041 | 0.0000 | | 482 | 4 | 4 |  | 0.0034 | 0.0026 | 0.3343 | 0.6568 | 0.0028 | 0.0000 | | 483 | 4 | 4 |  | 0.0082 | 0.0651 | 0.1040 | 0.8020 | 0.0080 | 0.0127 | | 484 | 4 | 4 |  | 0.0137 | 0.0488 | 0.1277 | 0.7850 | 0.0242 | 0.0006 | | 485 | 4 | 4 |  | 0.0357 | 0.0335 | 0.3009 | 0.5411 | 0.0881 | 0.0007 | | 486 | 4 | 1 | * | 0.3426 | 0.0720 | 0.3055 | 0.2693 | 0.0106 | 0.0000 | | 487 | 4 | 2 | * | 0.1817 | 0.4405 | 0.0583 | 0.2603 | 0.0587 | 0.0005 | | 488 | 4 | 4 |  | 0.2869 | 0.1220 | 0.0687 | 0.4219 | 0.1000 | 0.0005 | | 489 | 4 | 4 |  | 0.0216 | 0.0217 | 0.2270 | 0.6996 | 0.0301 | 0.0001 | | 490 | 4 | 4 |  | 0.0046 | 0.0035 | 0.3778 | 0.6100 | 0.0041 | 0.0000 | | 491 | 4 | 4 |  | 0.0098 | 0.0885 | 0.0819 | 0.7787 | 0.0116 | 0.0297 | | 492 | 4 | 1 | * | 0.4081 | 0.0826 | 0.2886 | 0.2073 | 0.0134 | 0.0000 | | 493 | 4 | 2 | * | 0.2025 | 0.4734 | 0.0559 | 0.1971 | 0.0703 | 0.0008 | | 494 | 4 | 1 | * | 0.4709 | 0.0462 | 0.2368 | 0.2004 | 0.0457 | 0.0000 | | 495 | 4 | 2 | * | 0.1006 | 0.4813 | 0.0607 | 0.3303 | 0.0248 | 0.0023 | | 496 | 4 | 1 | * | 0.3544 | 0.0698 | 0.0532 | 0.2424 | 0.2802 | 0.0001 | | 497 | 4 | 4 |  | 0.0160 | 0.0212 | 0.2953 | 0.6528 | 0.0146 | 0.0000 | | 498 | 4 | 4 |  | 0.0110 | 0.0025 | 0.3378 | 0.6156 | 0.0331 | 0.0000 | | 499 | 4 | 4 |  | 0.0083 | 0.1382 | 0.1095 | 0.6892 | 0.0037 | 0.0511 | | 500 | 4 | 4 |  | 0.0419 | 0.0811 | 0.1578 | 0.5773 | 0.1414 | 0.0005 | | 501 | 4 | 4 |  | 0.0181 | 0.1319 | 0.1425 | 0.6650 | 0.0156 | 0.0269 | | 502 | 4 | 3 | * | 0.3326 | 0.1372 | 0.3546 | 0.1708 | 0.0048 | 0.0000 | | 503 | 4 | 2 | * | 0.2071 | 0.5290 | 0.0526 | 0.1134 | 0.0975 | 0.0004 | | 504 | 4 | 4 |  | 0.2955 | 0.1207 | 0.0622 | 0.3370 | 0.1834 | 0.0013 | | 505 | 4 | 4 |  | 0.0243 | 0.0224 | 0.2049 | 0.7197 | 0.0285 | 0.0001 | | 506 | 4 | 4 |  | 0.0051 | 0.0031 | 0.4273 | 0.5570 | 0.0075 | 0.0000 | | 507 | 4 | 4 |  | 0.0241 | 0.2214 | 0.0954 | 0.4390 | 0.0333 | 0.1868 | | 508 | 4 | 4 |  | 0.0437 | 0.1446 | 0.1823 | 0.5197 | 0.1041 | 0.0057 | | 509 | 4 | 1 | * | 0.5171 | 0.0862 | 0.2300 | 0.1447 | 0.0220 | 0.0000 | | 510 | 4 | 2 | * | 0.1772 | 0.4554 | 0.0522 | 0.2561 | 0.0585 | 0.0006 | | 511 | 4 | 4 |  | 0.0212 | 0.0193 | 0.2565 | 0.6745 | 0.0284 | 0.0001 | | 512 | 4 | 1 | * | 0.3319 | 0.1692 | 0.0600 | 0.3255 | 0.1127 | 0.0006 | | 513 | 4 | 3 | * | 0.0839 | 0.2078 | 0.3704 | 0.3094 | 0.0283 | 0.0002 | | 514 | 4 | 4 |  | 0.1407 | 0.1010 | 0.2956 | 0.2994 | 0.1623 | 0.0010 | | 515 | 4 | 2 | * | 0.1657 | 0.3444 | 0.3263 | 0.1591 | 0.0045 | 0.0000 | | 516 | 4 | 4 |  | 0.0356 | 0.1841 | 0.0704 | 0.6208 | 0.0882 | 0.0008 | | 517 | 4 | 2 | * | 0.0721 | 0.4181 | 0.0691 | 0.2919 | 0.1461 | 0.0028 | | 518 | 4 | 4 |  | 0.1440 | 0.0724 | 0.2869 | 0.3292 | 0.1671 | 0.0003 | | 519 | 4 | 2 | * | 0.0653 | 0.4484 | 0.0738 | 0.3412 | 0.0649 | 0.0064 | | 520 | 4 | 4 |  | 0.0148 | 0.0115 | 0.4320 | 0.5047 | 0.0368 | 0.0001 | | 521 | 4 | 4 |  | 0.0507 | 0.3471 | 0.0560 | 0.4776 | 0.0683 | 0.0002 | | 522 | 4 | 4 |  | 0.1154 | 0.2925 | 0.2193 | 0.3700 | 0.0028 | 0.0000 | | 523 | 4 | 3 | * | 0.1309 | 0.2209 | 0.3968 | 0.2439 | 0.0073 | 0.0003 | | 524 | 4 | 4 |  | 0.0528 | 0.1511 | 0.3832 | 0.3979 | 0.0149 | 0.0001 | | 525 | 4 | 4 |  | 0.0987 | 0.0499 | 0.3140 | 0.3947 | 0.1406 | 0.0021 | | 526 | 4 | 1 | * | 0.3738 | 0.3109 | 0.0719 | 0.1023 | 0.1411 | 0.0000 | | 527 | 4 | 2 | * | 0.0855 | 0.3565 | 0.0789 | 0.3133 | 0.1613 | 0.0045 | | 528 | 4 | 3 | * | 0.1814 | 0.2918 | 0.3334 | 0.1881 | 0.0052 | 0.0000 | | 529 | 4 | 2 | * | 0.1943 | 0.4520 | 0.1400 | 0.1959 | 0.0176 | 0.0002 | | 530 | 4 | 2 | * | 0.3028 | 0.3423 | 0.0962 | 0.1387 | 0.1198 | 0.0001 | | 531 | 4 | 2 | * | 0.0999 | 0.3968 | 0.0624 | 0.2590 | 0.1809 | 0.0009 | | 532 | 4 | 4 |  | 0.0140 | 0.0084 | 0.4131 | 0.5274 | 0.0370 | 0.0000 | | 533 | 4 | 4 |  | 0.0532 | 0.2659 | 0.0546 | 0.5460 | 0.0802 | 0.0001 | | 534 | 4 | 3 | * | 0.1188 | 0.1507 | 0.3646 | 0.3376 | 0.0257 | 0.0025 | | 535 | 4 | 2 | * | 0.2141 | 0.3667 | 0.1743 | 0.2406 | 0.0043 | 0.0000 | | 536 | 4 | 3 | * | 0.0486 | 0.1041 | 0.4150 | 0.4082 | 0.0238 | 0.0003 | | 537 | 4 | 3 | * | 0.0397 | 0.2264 | 0.4174 | 0.3073 | 0.0067 | 0.0024 | | 538 | 4 | 3 | * | 0.1361 | 0.2234 | 0.3906 | 0.2419 | 0.0077 | 0.0003 | | 539 | 4 | 4 |  | 0.0123 | 0.0054 | 0.4183 | 0.5274 | 0.0365 | 0.0001 | | 540 | 4 | 4 |  | 0.1019 | 0.2948 | 0.2256 | 0.3762 | 0.0015 | 0.0000 | | 541 | 4 | 4 |  | 0.0381 | 0.2122 | 0.0581 | 0.5880 | 0.1028 | 0.0009 | | 542 | 4 | 3 | * | 0.0471 | 0.2314 | 0.4565 | 0.2562 | 0.0069 | 0.0018 | | 543 | 4 | 4 |  | 0.0097 | 0.0060 | 0.4075 | 0.5373 | 0.0393 | 0.0003 | | 544 | 4 | 4 |  | 0.1432 | 0.2679 | 0.2310 | 0.3520 | 0.0055 | 0.0003 | | 545 | 4 | 3 | * | 0.0928 | 0.3478 | 0.3887 | 0.1694 | 0.0010 | 0.0002 | | 546 | 4 | 4 |  | 0.0158 | 0.0063 | 0.4597 | 0.4768 | 0.0414 | 0.0001 | | 547 | 4 | 4 |  | 0.0137 | 0.0179 | 0.3860 | 0.5717 | 0.0105 | 0.0001 | | 548 | 4 | 2 | * | 0.0604 | 0.5268 | 0.0507 | 0.3386 | 0.0229 | 0.0006 | | 549 | 4 | 4 |  | 0.0959 | 0.3286 | 0.2195 | 0.3548 | 0.0010 | 0.0001 | | 550 | 4 | 2 | * | 0.0764 | 0.4954 | 0.0695 | 0.2353 | 0.1215 | 0.0019 | | 551 | 4 | 1 | * | 0.3247 | 0.2621 | 0.1062 | 0.1586 | 0.1481 | 0.0003 | | 552 | 4 | 4 |  | 0.1305 | 0.1094 | 0.2975 | 0.3211 | 0.1408 | 0.0007 | | 553 | 4 | 2 | * | 0.2817 | 0.3675 | 0.1089 | 0.1563 | 0.0856 | 0.0000 | | 554 | 4 | 2 | * | 0.0468 | 0.4309 | 0.0549 | 0.4058 | 0.0615 | 0.0001 | | 555 | 4 | 3 | * | 0.1371 | 0.3256 | 0.3584 | 0.1769 | 0.0020 | 0.0000 | | 556 | 4 | 2 | * | 0.1126 | 0.3347 | 0.0819 | 0.2520 | 0.2164 | 0.0024 | | 557 | 4 | 4 |  | 0.0146 | 0.0133 | 0.4637 | 0.4817 | 0.0266 | 0.0000 | | 558 | 4 | 3 | * | 0.1771 | 0.2141 | 0.4124 | 0.1863 | 0.0099 | 0.0001 | | 559 | 4 | 4 |  | 0.0612 | 0.1790 | 0.3556 | 0.3853 | 0.0188 | 0.0001 | | 560 | 4 | 1 | * | 0.3822 | 0.0308 | 0.2194 | 0.2575 | 0.1101 | 0.0000 | | 561 | 4 | 4 |  | 0.1295 | 0.2729 | 0.2200 | 0.3560 | 0.0214 | 0.0002 | | 562 | 4 | 4 |  | 0.1672 | 0.0818 | 0.2506 | 0.3633 | 0.1371 | 0.0000 | | 563 | 4 | 4 |  | 0.1120 | 0.0391 | 0.3183 | 0.3994 | 0.1309 | 0.0003 | | 564 | 4 | 4 |  | 0.1025 | 0.0558 | 0.3382 | 0.4441 | 0.0590 | 0.0003 | | 565 | 4 | 4 |  | 0.0326 | 0.0686 | 0.2276 | 0.6500 | 0.0137 | 0.0075 | | 566 | 4 | 4 |  | 0.1048 | 0.1002 | 0.3069 | 0.4403 | 0.0477 | 0.0001 | | 567 | 4 | 3 | * | 0.0579 | 0.1461 | 0.4044 | 0.3877 | 0.0036 | 0.0004 | | 568 | 4 | 4 |  | 0.0886 | 0.1389 | 0.3686 | 0.3920 | 0.0109 | 0.0011 | | 569 | 4 | 4 |  | 0.0251 | 0.0276 | 0.2560 | 0.6823 | 0.0087 | 0.0002 | | 570 | 4 | 4 |  | 0.0249 | 0.0851 | 0.2606 | 0.6234 | 0.0030 | 0.0030 | | 571 | 4 | 4 |  | 0.1439 | 0.1026 | 0.2793 | 0.3966 | 0.0777 | 0.0000 | | 572 | 4 | 5 | * | 0.2071 | 0.1607 | 0.0194 | 0.0356 | 0.5744 | 0.0027 | | 573 | 4 | 3 | * | 0.1129 | 0.2665 | 0.4854 | 0.1102 | 0.0240 | 0.0010 | | 574 | 4 | 4 |  | 0.1668 | 0.2481 | 0.2207 | 0.3274 | 0.0370 | 0.0001 | | 575 | 4 | 3 | * | 0.0876 | 0.2971 | 0.4989 | 0.1003 | 0.0140 | 0.0021 | | 576 | 4 | 4 |  | 0.0401 | 0.0286 | 0.2628 | 0.6532 | 0.0151 | 0.0001 | | 577 | 4 | 4 |  | 0.1397 | 0.0346 | 0.2820 | 0.3374 | 0.2061 | 0.0002 | | 578 | 4 | 4 |  | 0.1016 | 0.0284 | 0.2218 | 0.5410 | 0.1071 | 0.0001 | | 579 | 4 | 4 |  | 0.2220 | 0.2310 | 0.1841 | 0.3098 | 0.0530 | 0.0000 | | 580 | 4 | 2 | * | 0.1233 | 0.4159 | 0.2083 | 0.1414 | 0.1111 | 0.0000 | | 581 | 4 | 4 |  | 0.0806 | 0.1041 | 0.3323 | 0.4648 | 0.0181 | 0.0001 | | 582 | 4 | 5 | * | 0.1620 | 0.0277 | 0.1913 | 0.2219 | 0.3970 | 0.0002 | | 583 | 4 | 4 |  | 0.1025 | 0.0653 | 0.1856 | 0.5351 | 0.1081 | 0.0033 | | 584 | 4 | 1 | * | 0.4084 | 0.0237 | 0.1709 | 0.2088 | 0.1882 | 0.0000 | | 585 | 4 | 5 | * | 0.1523 | 0.0933 | 0.0111 | 0.0183 | 0.7235 | 0.0014 | | 586 | 4 | 4 |  | 0.0834 | 0.3264 | 0.2526 | 0.3300 | 0.0074 | 0.0003 | | 587 | 4 | 4 |  | 0.1975 | 0.1148 | 0.2775 | 0.3418 | 0.0679 | 0.0005 | | 588 | 4 | 2 | * | 0.0921 | 0.5027 | 0.2023 | 0.1507 | 0.0522 | 0.0000 | | 589 | 4 | 5 | * | 0.1632 | 0.2516 | 0.1394 | 0.1002 | 0.3456 | 0.0000 | | 590 | 4 | 1 | * | 0.3962 | 0.0155 | 0.1355 | 0.1878 | 0.2650 | 0.0000 | | 591 | 4 | 5 | * | 0.0816 | 0.0322 | 0.0037 | 0.0075 | 0.8750 | 0.0001 | | 592 | 4 | 4 |  | 0.1813 | 0.0477 | 0.3699 | 0.3876 | 0.0135 | 0.0001 | | 593 | 4 | 2 | * | 0.2243 | 0.4205 | 0.0503 | 0.0822 | 0.2135 | 0.0092 | | 594 | 4 | 3 | * | 0.1489 | 0.2671 | 0.4431 | 0.0891 | 0.0510 | 0.0008 | | 595 | 4 | 4 |  | 0.0475 | 0.0249 | 0.2606 | 0.6424 | 0.0246 | 0.0001 | | 596 | 4 | 2 | * | 0.0598 | 0.3481 | 0.2582 | 0.3312 | 0.0022 | 0.0006 | | 597 | 4 | 4 |  | 0.3095 | 0.0367 | 0.2830 | 0.3120 | 0.0588 | 0.0000 | | 598 | 4 | 4 |  | 0.0245 | 0.0745 | 0.2610 | 0.6290 | 0.0050 | 0.0059 | | 599 | 4 | 3 | * | 0.0780 | 0.3808 | 0.4470 | 0.0831 | 0.0055 | 0.0056 | | 600 | 4 | 2 | * | 0.0872 | 0.4128 | 0.2547 | 0.1911 | 0.0541 | 0.0001 | | 601 | 4 | 3 | * | 0.0634 | 0.0701 | 0.4398 | 0.4047 | 0.0206 | 0.0015 | | 602 | 4 | 4 |  | 0.0433 | 0.0608 | 0.2154 | 0.6527 | 0.0234 | 0.0043 | | 603 | 4 | 4 |  | 0.0580 | 0.1136 | 0.3466 | 0.4682 | 0.0135 | 0.0001 | | 604 | 4 | 3 | * | 0.2143 | 0.1896 | 0.3437 | 0.0827 | 0.1695 | 0.0002 | | 605 | 4 | 5 | * | 0.1815 | 0.2605 | 0.0342 | 0.0682 | 0.4327 | 0.0229 | | 606 | 4 | 4 |  | 0.1157 | 0.1306 | 0.3648 | 0.3690 | 0.0193 | 0.0005 | | 607 | 4 | 4 |  | 0.0142 | 0.0260 | 0.2748 | 0.6828 | 0.0015 | 0.0006 | | 608 | 4 | 2 | * | 0.1552 | 0.3563 | 0.1957 | 0.1157 | 0.1771 | 0.0000 | | 609 | 4 | 4 |  | 0.0592 | 0.1291 | 0.3783 | 0.4253 | 0.0060 | 0.0022 | | 610 | 5 | 5 |  | 0.2283 | 0.0076 | 0.0057 | 0.0110 | 0.7472 | 0.0002 | | 611 | 5 | 1 | * | 0.3517 | 0.0340 | 0.0424 | 0.2246 | 0.3397 | 0.0076 | | 612 | 5 | 5 |  | 0.0708 | 0.0277 | 0.1166 | 0.1406 | 0.6443 | 0.0000 | | 613 | 5 | 5 |  | 0.0229 | 0.1777 | 0.0098 | 0.0469 | 0.7424 | 0.0003 | | 614 | 5 | 5 |  | 0.0489 | 0.0081 | 0.0394 | 0.0466 | 0.8570 | 0.0000 | | 615 | 5 | 5 |  | 0.4187 | 0.0296 | 0.0490 | 0.0132 | 0.4892 | 0.0004 | | 616 | 5 | 5 |  | 0.0182 | 0.0833 | 0.0047 | 0.0200 | 0.8738 | 0.0001 | | 617 | 5 | 5 |  | 0.0754 | 0.0263 | 0.1236 | 0.3502 | 0.3978 | 0.0268 | | 618 | 5 | 5 |  | 0.0155 | 0.0617 | 0.0030 | 0.0120 | 0.9077 | 0.0000 | | 619 | 5 | 5 |  | 0.1454 | 0.1129 | 0.0258 | 0.0290 | 0.6869 | 0.0000 | | 620 | 5 | 4 | * | 0.0621 | 0.0636 | 0.2677 | 0.3670 | 0.2397 | 0.0000 | | 621 | 5 | 5 |  | 0.1185 | 0.0016 | 0.0012 | 0.0024 | 0.8763 | 0.0000 | | 622 | 5 | 5 |  | 0.3184 | 0.0152 | 0.0116 | 0.0201 | 0.6342 | 0.0005 | | 623 | 5 | 1 | * | 0.3587 | 0.0484 | 0.0532 | 0.2694 | 0.2517 | 0.0186 | | 624 | 5 | 1 | * | 0.4255 | 0.0779 | 0.1160 | 0.0347 | 0.3350 | 0.0109 | | 625 | 5 | 2 | * | 0.0263 | 0.4138 | 0.0268 | 0.1527 | 0.3793 | 0.0010 | | 626 | 5 | 5 |  | 0.1800 | 0.0043 | 0.0037 | 0.0073 | 0.8047 | 0.0001 | | 627 | 5 | 5 |  | 0.1233 | 0.0709 | 0.0163 | 0.0207 | 0.7686 | 0.0000 | | 628 | 5 | 5 |  | 0.0704 | 0.0199 | 0.0758 | 0.0859 | 0.7480 | 0.0000 | | 629 | 5 | 1 | * | 0.3697 | 0.1478 | 0.1868 | 0.0479 | 0.1152 | 0.1326 | | 630 | 5 | 5 |  | 0.1181 | 0.0350 | 0.0098 | 0.0119 | 0.8251 | 0.0000 | | 631 | 5 | 5 |  | 0.1767 | 0.1887 | 0.0335 | 0.0321 | 0.5689 | 0.0000 | | 632 | 5 | 5 |  | 0.0791 | 0.0188 | 0.0995 | 0.2780 | 0.5164 | 0.0081 | | 633 | 5 | 6 | * | 0.0397 | 0.0283 | 0.1070 | 0.3402 | 0.0715 | 0.4135 | | 634 | 5 | 5 |  | 0.1130 | 0.0014 | 0.0010 | 0.0018 | 0.8827 | 0.0000 | | 635 | 5 | 5 |  | 0.1241 | 0.0700 | 0.0169 | 0.0208 | 0.7681 | 0.0000 | | 636 | 5 | 4 | * | 0.0682 | 0.0342 | 0.1490 | 0.3960 | 0.2786 | 0.0740 | | 637 | 5 | 1 | * | 0.5139 | 0.0402 | 0.0280 | 0.0454 | 0.3664 | 0.0061 | | 638 | 5 | 5 |  | 0.0256 | 0.2754 | 0.0152 | 0.0694 | 0.6138 | 0.0007 | | 639 | 5 | 5 |  | 0.3815 | 0.0530 | 0.0724 | 0.0229 | 0.4658 | 0.0043 | | 640 | 5 | 5 |  | 0.3907 | 0.0274 | 0.0518 | 0.0172 | 0.5125 | 0.0004 | | 641 | 5 | 6 | * | 0.0397 | 0.0283 | 0.1070 | 0.3402 | 0.0715 | 0.4135 | | 642 | 5 | 5 |  | 0.0656 | 0.0434 | 0.1931 | 0.2872 | 0.4107 | 0.0000 | | 643 | 5 | 2 | * | 0.2113 | 0.3448 | 0.0559 | 0.0494 | 0.3381 | 0.0006 | | 644 | 5 | 5 |  | 0.0629 | 0.0101 | 0.0614 | 0.2354 | 0.6255 | 0.0046 | | 645 | 5 | 5 |  | 0.2912 | 0.0179 | 0.0244 | 0.1234 | 0.5422 | 0.0009 | | 646 | 5 | 1 | * | 0.4235 | 0.1068 | 0.1459 | 0.0438 | 0.2467 | 0.0333 | | 647 | 5 | 5 |  | 0.0712 | 0.0267 | 0.1241 | 0.1403 | 0.6377 | 0.0000 | | 648 | 5 | 5 |  | 0.0229 | 0.1777 | 0.0098 | 0.0469 | 0.7424 | 0.0003 | | 649 | 5 | 5 |  | 0.3499 | 0.0505 | 0.0703 | 0.0220 | 0.5003 | 0.0070 | | 650 | 5 | 5 |  | 0.0182 | 0.0833 | 0.0047 | 0.0200 | 0.8738 | 0.0001 | | 651 | 5 | 4 | * | 0.2424 | 0.0693 | 0.0722 | 0.4343 | 0.0825 | 0.0994 | | 652 | 5 | 5 |  | 0.0750 | 0.0272 | 0.1161 | 0.3508 | 0.4018 | 0.0291 | | 653 | 5 | 5 |  | 0.0155 | 0.0617 | 0.0030 | 0.0120 | 0.9077 | 0.0000 | | 654 | 5 | 4 | * | 0.3052 | 0.0574 | 0.0679 | 0.3518 | 0.1729 | 0.0448 | | 655 | 5 | 5 |  | 0.2689 | 0.0699 | 0.0496 | 0.0207 | 0.5908 | 0.0002 | | 656 | 5 | 1 | * | 0.5328 | 0.1598 | 0.2094 | 0.0782 | 0.0192 | 0.0007 | | 657 | 5 | 5 |  | 0.0313 | 0.0198 | 0.1190 | 0.1583 | 0.6715 | 0.0001 | | 658 | 5 | 1 | * | 0.4685 | 0.1632 | 0.0228 | 0.0478 | 0.2939 | 0.0038 | | 659 | 5 | 2 | * | 0.1032 | 0.3705 | 0.0588 | 0.1434 | 0.0071 | 0.3171 | | 660 | 5 | 5 |  | 0.0754 | 0.0745 | 0.0152 | 0.0006 | 0.8329 | 0.0014 | | 661 | 5 | 5 |  | 0.0773 | 0.0302 | 0.0576 | 0.0121 | 0.8227 | 0.0000 | | 662 | 5 | 4 | * | 0.0149 | 0.0336 | 0.1641 | 0.7854 | 0.0015 | 0.0005 | | 663 | 5 | 5 |  | 0.0129 | 0.0120 | 0.0078 | 0.0019 | 0.9599 | 0.0055 | | 664 | 5 | 1 | * | 0.6819 | 0.0863 | 0.1260 | 0.0522 | 0.0535 | 0.0001 | | 665 | 5 | 5 |  | 0.0164 | 0.0052 | 0.0017 | 0.0001 | 0.9765 | 0.0000 | | 666 | 5 | 5 |  | 0.1264 | 0.0115 | 0.0681 | 0.0161 | 0.7779 | 0.0000 | | 667 | 5 | 5 |  | 0.1403 | 0.0178 | 0.1116 | 0.0271 | 0.7033 | 0.0001 | | 668 | 5 | 5 |  | 0.0630 | 0.0425 | 0.0094 | 0.0004 | 0.8843 | 0.0004 | | 669 | 5 | 2 | * | 0.3548 | 0.3597 | 0.0585 | 0.1335 | 0.0759 | 0.0175 | | 670 | 5 | 3 | * | 0.1725 | 0.0603 | 0.3715 | 0.0988 | 0.2948 | 0.0022 | | 671 | 5 | 1 | * | 0.3959 | 0.0226 | 0.0712 | 0.1301 | 0.3802 | 0.0000 | | 672 | 5 | 5 |  | 0.0149 | 0.0206 | 0.0129 | 0.0031 | 0.9298 | 0.0187 | | 673 | 5 | 5 |  | 0.1180 | 0.3264 | 0.0825 | 0.0038 | 0.4478 | 0.0215 | | 674 | 5 | 5 |  | 0.0398 | 0.0111 | 0.0239 | 0.0051 | 0.9201 | 0.0000 | | 675 | 5 | 4 | * | 0.0230 | 0.0241 | 0.1443 | 0.8032 | 0.0053 | 0.0001 | | 676 | 5 | 4 | * | 0.2812 | 0.0642 | 0.2051 | 0.3827 | 0.0660 | 0.0007 | | 677 | 5 | 1 | * | 0.3899 | 0.3138 | 0.0617 | 0.1235 | 0.0841 | 0.0270 | | 678 | 5 | 2 | * | 0.2921 | 0.4036 | 0.0655 | 0.1572 | 0.0470 | 0.0347 | | 679 | 5 | 4 | * | 0.0167 | 0.0231 | 0.1443 | 0.8128 | 0.0029 | 0.0001 | | 680 | 5 | 1 | * | 0.7130 | 0.0660 | 0.0895 | 0.0385 | 0.0930 | 0.0000 | | 681 | 5 | 4 | * | 0.0337 | 0.0531 | 0.3024 | 0.3654 | 0.2430 | 0.0024 | | 682 | 5 | 1 | * | 0.7298 | 0.0465 | 0.0598 | 0.0250 | 0.1389 | 0.0000 | | 683 | 5 | 5 |  | 0.1572 | 0.0198 | 0.0144 | 0.0058 | 0.8028 | 0.0000 | | 684 | 5 | 5 |  | 0.0213 | 0.0720 | 0.0683 | 0.0218 | 0.5389 | 0.2778 | | 685 | 5 | 5 |  | 0.0158 | 0.0035 | 0.0236 | 0.0319 | 0.9251 | 0.0000 | | 686 | 5 | 5 |  | 0.2198 | 0.0400 | 0.0269 | 0.0102 | 0.7031 | 0.0001 | | 687 | 5 | 5 |  | 0.0241 | 0.0037 | 0.0066 | 0.0014 | 0.9642 | 0.0000 | | 688 | 5 | 5 |  | 0.0219 | 0.0094 | 0.0564 | 0.0755 | 0.8368 | 0.0001 | | 689 | 5 | 5 |  | 0.0058 | 0.0020 | 0.0013 | 0.0003 | 0.9905 | 0.0002 | | 690 | 5 | 1 | * | 0.7357 | 0.0209 | 0.0263 | 0.0099 | 0.2073 | 0.0000 | | 691 | 5 | 5 |  | 0.0498 | 0.0013 | 0.0071 | 0.0015 | 0.9403 | 0.0000 | | 692 | 5 | 5 |  | 0.2969 | 0.1934 | 0.1289 | 0.0516 | 0.3270 | 0.0022 | | 693 | 5 | 5 |  | 0.0815 | 0.0056 | 0.0342 | 0.0084 | 0.8705 | 0.0000 | | 694 | 5 | 5 |  | 0.0096 | 0.0050 | 0.0032 | 0.0006 | 0.9808 | 0.0008 | | 695 | 5 | 5 |  | 0.2306 | 0.0068 | 0.0196 | 0.0355 | 0.7076 | 0.0000 | | 696 | 5 | 5 |  | 0.2806 | 0.1004 | 0.0689 | 0.0304 | 0.5192 | 0.0005 | | 697 | 5 | 4 | * | 0.0504 | 0.0198 | 0.1236 | 0.7687 | 0.0375 | 0.0000 | | 698 | 5 | 5 |  | 0.0902 | 0.0513 | 0.0879 | 0.0174 | 0.7531 | 0.0001 | | 699 | 5 | 1 | * | 0.3962 | 0.0338 | 0.0926 | 0.1704 | 0.3069 | 0.0000 | | 700 | 5 | 5 |  | 0.1433 | 0.1451 | 0.2622 | 0.0456 | 0.4032 | 0.0006 | | 701 | 5 | 5 |  | 0.0336 | 0.0295 | 0.1769 | 0.2245 | 0.5353 | 0.0002 | | 702 | 5 | 4 | * | 0.0235 | 0.0170 | 0.1290 | 0.8219 | 0.0086 | 0.0000 | | 703 | 5 | 5 |  | 0.3339 | 0.0111 | 0.0320 | 0.0509 | 0.5721 | 0.0000 | | 704 | 5 | 5 |  | 0.0198 | 0.0068 | 0.0035 | 0.0006 | 0.9693 | 0.0000 | | 705 | 5 | 2 | * | 0.2053 | 0.4008 | 0.0719 | 0.1870 | 0.0279 | 0.1072 | | 706 | 5 | 1 | * | 0.3636 | 0.3380 | 0.0848 | 0.0154 | 0.1926 | 0.0056 | | 707 | 5 | 1 | * | 0.5014 | 0.1238 | 0.0372 | 0.0172 | 0.3202 | 0.0002 | | 708 | 5 | 2 | * | 0.2890 | 0.4875 | 0.0219 | 0.0642 | 0.1374 | 0.0000 | | 709 | 5 | 2 | * | 0.1822 | 0.6568 | 0.0310 | 0.0896 | 0.0404 | 0.0000 | | 710 | 5 | 5 |  | 0.0713 | 0.0358 | 0.2058 | 0.0851 | 0.6020 | 0.0000 | | 711 | 5 | 5 |  | 0.4327 | 0.0805 | 0.0251 | 0.0128 | 0.4488 | 0.0002 | | 712 | 5 | 2 | * | 0.2432 | 0.4811 | 0.1549 | 0.0784 | 0.0420 | 0.0004 | | 713 | 5 | 5 |  | 0.0298 | 0.0017 | 0.0036 | 0.0019 | 0.9620 | 0.0010 | | 714 | 5 | 1 | * | 0.4838 | 0.1933 | 0.0728 | 0.0358 | 0.2142 | 0.0001 | | 715 | 5 | 2 | * | 0.1758 | 0.3033 | 0.0561 | 0.1945 | 0.2701 | 0.0002 | | 716 | 5 | 5 |  | 0.0433 | 0.0024 | 0.0096 | 0.0054 | 0.9392 | 0.0000 | | 717 | 5 | 5 |  | 0.2529 | 0.2044 | 0.0437 | 0.1174 | 0.3809 | 0.0007 | | 718 | 5 | 5 |  | 0.0610 | 0.0226 | 0.1279 | 0.0506 | 0.7380 | 0.0000 | | 719 | 5 | 3 | * | 0.1299 | 0.2458 | 0.4300 | 0.1779 | 0.0163 | 0.0001 | | 720 | 5 | 5 |  | 0.0338 | 0.0012 | 0.0046 | 0.0025 | 0.9578 | 0.0000 | | 721 | 5 | 5 |  | 0.2800 | 0.1442 | 0.0258 | 0.0594 | 0.4899 | 0.0006 | | 722 | 5 | 1 | * | 0.4184 | 0.2602 | 0.0915 | 0.0549 | 0.1745 | 0.0006 | | 723 | 5 | 5 |  | 0.1737 | 0.2090 | 0.0355 | 0.1207 | 0.4609 | 0.0003 | | 724 | 5 | 5 |  | 0.0612 | 0.0048 | 0.0191 | 0.0099 | 0.9051 | 0.0000 | | 725 | 5 | 3 | * | 0.1741 | 0.2215 | 0.4054 | 0.1744 | 0.0245 | 0.0000 | | 726 | 5 | 5 |  | 0.0417 | 0.0033 | 0.0075 | 0.0041 | 0.9422 | 0.0012 | | 727 | 5 | 2 | * | 0.3133 | 0.4206 | 0.1287 | 0.0679 | 0.0692 | 0.0004 | | 728 | 5 | 2 | * | 0.2503 | 0.5786 | 0.0277 | 0.0768 | 0.0665 | 0.0000 | | 729 | 5 | 5 |  | 0.3499 | 0.1662 | 0.0466 | 0.0086 | 0.4212 | 0.0075 | | 730 | 5 | 2 | * | 0.2782 | 0.5177 | 0.0895 | 0.0113 | 0.1020 | 0.0014 | | 731 | 5 | 5 |  | 0.3263 | 0.2701 | 0.0149 | 0.0507 | 0.3380 | 0.0000 | | 732 | 5 | 5 |  | 0.0755 | 0.0087 | 0.0454 | 0.0272 | 0.8432 | 0.0000 | | 733 | 5 | 5 |  | 0.3562 | 0.0330 | 0.0102 | 0.0052 | 0.5952 | 0.0001 | | 734 | 5 | 1 | * | 0.3942 | 0.3715 | 0.0947 | 0.0451 | 0.0941 | 0.0003 | | 735 | 5 | 3 | * | 0.2398 | 0.2180 | 0.3426 | 0.1503 | 0.0493 | 0.0001 | | 736 | 5 | 5 |  | 0.0191 | 0.0005 | 0.0013 | 0.0007 | 0.9779 | 0.0005 | | 737 | 5 | 2 | * | 0.2335 | 0.2878 | 0.0668 | 0.1601 | 0.2511 | 0.0007 | | 738 | 5 | 5 |  | 0.0337 | 0.0009 | 0.0044 | 0.0025 | 0.9586 | 0.0000 | | 739 | 5 | 2 | * | 0.1393 | 0.3612 | 0.0837 | 0.2753 | 0.1404 | 0.0001 | | 740 | 5 | 5 |  | 0.0447 | 0.0102 | 0.0761 | 0.0317 | 0.8372 | 0.0000 | | 741 | 5 | 2 | * | 0.1753 | 0.4968 | 0.1958 | 0.1093 | 0.0224 | 0.0003 | | 742 | 5 | 3 | * | 0.0762 | 0.2016 | 0.4789 | 0.2344 | 0.0088 | 0.0001 | | 743 | 5 | 5 |  | 0.2042 | 0.0492 | 0.0109 | 0.0253 | 0.7100 | 0.0004 | | 744 | 5 | 5 |  | 0.0501 | 0.0411 | 0.2762 | 0.1248 | 0.5078 | 0.0001 | | 745 | 5 | 5 |  | 0.1340 | 0.0623 | 0.0108 | 0.0397 | 0.7530 | 0.0001 | | 746 | 5 | 5 |  | 0.0529 | 0.0134 | 0.0687 | 0.0252 | 0.8398 | 0.0000 | | 747 | 5 | 3 | * | 0.2616 | 0.1621 | 0.3396 | 0.1558 | 0.0807 | 0.0001 | | 748 | 5 | 5 |  | 0.0528 | 0.0062 | 0.0166 | 0.0095 | 0.9131 | 0.0017 | | 749 | 5 | 2 | * | 0.2459 | 0.4867 | 0.1584 | 0.0308 | 0.0735 | 0.0047 | | 750 | 5 | 1 | * | 0.3796 | 0.2576 | 0.0555 | 0.0096 | 0.2915 | 0.0061 | | 751 | 5 | 2 | * | 0.3197 | 0.3916 | 0.0156 | 0.0451 | 0.2279 | 0.0000 | | 752 | 5 | 5 |  | 0.2919 | 0.1038 | 0.0168 | 0.0359 | 0.5513 | 0.0004 | | 753 | 5 | 5 |  | 0.3921 | 0.0515 | 0.0135 | 0.0075 | 0.5353 | 0.0001 | | 754 | 5 | 5 |  | 0.1649 | 0.1397 | 0.0208 | 0.0671 | 0.6072 | 0.0004 | | 755 | 5 | 5 |  | 0.0251 | 0.0010 | 0.0021 | 0.0010 | 0.9702 | 0.0006 | | 756 | 6 | 2 | * | 0.1458 | 0.5487 | 0.0569 | 0.1020 | 0.1081 | 0.0385 | | 757 | 6 | 5 | * | 0.0166 | 0.0366 | 0.0850 | 0.0386 | 0.4122 | 0.4110 | | 758 | 6 | 6 |  | 0.1127 | 0.1045 | 0.1074 | 0.0144 | 0.1674 | 0.4935 | | 759 | 6 | 5 | * | 0.0755 | 0.0318 | 0.0110 | 0.0133 | 0.8680 | 0.0003 | | 760 | 6 | 6 |  | 0.0090 | 0.0414 | 0.2248 | 0.3089 | 0.0030 | 0.4129 | | 761 | 6 | 2 | * | 0.3037 | 0.6275 | 0.0163 | 0.0344 | 0.0172 | 0.0009 | | 762 | 6 | 6 |  | 0.1141 | 0.0084 | 0.0769 | 0.0833 | 0.2824 | 0.4348 | | 763 | 6 | 3 | * | 0.0430 | 0.1575 | 0.3684 | 0.2786 | 0.0247 | 0.1278 | | 764 | 6 | 6 |  | 0.0276 | 0.0338 | 0.0423 | 0.0468 | 0.0790 | 0.7706 | | 765 | 6 | 2 | * | 0.3607 | 0.5504 | 0.0152 | 0.0393 | 0.0338 | 0.0005 | | 766 | 6 | 5 | * | 0.1920 | 0.2832 | 0.0254 | 0.0412 | 0.4428 | 0.0154 | | 767 | 6 | 2 | * | 0.3632 | 0.5125 | 0.0111 | 0.0277 | 0.0801 | 0.0055 | | 768 | 6 | 6 |  | 0.0019 | 0.0087 | 0.0027 | 0.0240 | 0.0237 | 0.9390 | | 769 | 6 | 6 |  | 0.0079 | 0.0331 | 0.1676 | 0.2024 | 0.0028 | 0.5862 | | 770 | 6 | 6 |  | 0.0233 | 0.0317 | 0.0387 | 0.0553 | 0.0786 | 0.7723 | | 771 | 6 | 6 |  | 0.0021 | 0.0074 | 0.0095 | 0.0126 | 0.0020 | 0.9664 | | 772 | 6 | 2 | * | 0.2215 | 0.7057 | 0.0196 | 0.0463 | 0.0065 | 0.0005 | | 773 | 6 | 6 |  | 0.1050 | 0.0085 | 0.0805 | 0.1107 | 0.2952 | 0.4000 | | 774 | 6 | 3 | * | 0.0538 | 0.1675 | 0.3789 | 0.2396 | 0.0250 | 0.1351 | | 775 | 6 | 5 | * | 0.0622 | 0.0302 | 0.0110 | 0.0165 | 0.8797 | 0.0003 | | 776 | 6 | 6 |  | 0.0297 | 0.0554 | 0.0797 | 0.0938 | 0.0418 | 0.6996 | | 777 | 6 | 6 |  | 0.0009 | 0.0066 | 0.0027 | 0.0220 | 0.0049 | 0.9630 | | 778 | 6 | 5 | * | 0.0145 | 0.0363 | 0.0889 | 0.0471 | 0.4371 | 0.3761 | | 779 | 6 | 6 |  | 0.1233 | 0.0951 | 0.1021 | 0.0118 | 0.1472 | 0.5205 | | 780 | 6 | 2 | * | 0.0871 | 0.6763 | 0.0610 | 0.0895 | 0.0184 | 0.0677 | | 781 | 6 | 5 | * | 0.1226 | 0.0039 | 0.0337 | 0.0395 | 0.7774 | 0.0229 | | 782 | 6 | 6 |  | 0.0170 | 0.0397 | 0.0421 | 0.0057 | 0.0065 | 0.8890 | | 783 | 6 | 6 |  | 0.0020 | 0.0083 | 0.0026 | 0.0205 | 0.0213 | 0.9453 | | 784 | 6 | 6 |  | 0.0007 | 0.0077 | 0.0480 | 0.0895 | 0.0002 | 0.8539 | | 785 | 6 | 5 | * | 0.0681 | 0.0573 | 0.0389 | 0.1100 | 0.7231 | 0.0025 | | 786 | 6 | 3 | * | 0.0129 | 0.1228 | 0.3381 | 0.2676 | 0.0023 | 0.2563 | | 787 | 6 | 6 |  | 0.0084 | 0.0285 | 0.0688 | 0.0371 | 0.1908 | 0.6664 | | 788 | 6 | 6 |  | 0.0396 | 0.0252 | 0.0318 | 0.0419 | 0.3057 | 0.5557 | | 789 | 6 | 3 | * | 0.0267 | 0.1403 | 0.3642 | 0.2849 | 0.0077 | 0.1762 | | 790 | 6 | 6 |  | 0.0002 | 0.0028 | 0.0010 | 0.0081 | 0.0005 | 0.9876 | | 791 | 6 | 5 | * | 0.0232 | 0.0056 | 0.0021 | 0.0030 | 0.9661 | 0.0001 | | 792 | 6 | 2 | * | 0.1627 | 0.5510 | 0.0559 | 0.0854 | 0.1016 | 0.0433 | | 793 | 6 | 6 |  | 0.1009 | 0.0100 | 0.0914 | 0.1457 | 0.1634 | 0.4887 | | 794 | 6 | 6 |  | 0.0309 | 0.0067 | 0.0638 | 0.0885 | 0.0354 | 0.7746 | | 795 | 6 | 6 |  | 0.0078 | 0.0146 | 0.0039 | 0.0336 | 0.3604 | 0.5797 | | 796 | 6 | 5 | * | 0.0941 | 0.1818 | 0.0639 | 0.1074 | 0.4247 | 0.1281 | | 797 | 6 | 6 |  | 0.0081 | 0.0333 | 0.0608 | 0.0281 | 0.1389 | 0.7308 | | 798 | 6 | 5 | * | 0.1211 | 0.0446 | 0.0453 | 0.0057 | 0.6820 | 0.1014 | | 799 | 6 | 4 | * | 0.0310 | 0.0580 | 0.2656 | 0.3261 | 0.0385 | 0.2808 | | 800 | 6 | 2 | * | 0.1062 | 0.6443 | 0.0472 | 0.0938 | 0.0524 | 0.0560 | | 801 | 6 | 6 |  | 0.1044 | 0.1309 | 0.1243 | 0.0158 | 0.0834 | 0.5411 | | 802 | 6 | 6 |  | 0.0032 | 0.0206 | 0.1253 | 0.1980 | 0.0010 | 0.6518 | | 803 | 6 | 3 | * | 0.1071 | 0.1495 | 0.3455 | 0.2278 | 0.1455 | 0.0245 | | 804 | 6 | 1 | * | 0.4852 | 0.3573 | 0.0086 | 0.0196 | 0.1289 | 0.0004 | | 805 | 6 | 5 | * | 0.0090 | 0.0070 | 0.0137 | 0.0062 | 0.9585 | 0.0056 | | 806 | 6 | 6 |  | 0.0001 | 0.0001 | 0.0000 | 0.0000 | 0.0002 | 0.9996 | | 807 | 6 | 6 |  | 0.0000 | 0.0000 | 0.0002 | 0.0002 | 0.0004 | 0.9993 | | 808 | 6 | 6 |  | 0.0000 | 0.0000 | 0.0000 | 0.0000 | 0.0000 | 1.0000 | | 809 | 6 | 6 |  | 0.0000 | 0.0002 | 0.0000 | 0.0002 | 0.0001 | 0.9994 | | 810 | 6 | 6 |  | 0.0000 | 0.0000 | 0.0000 | 0.0000 | 0.0000 | 1.0000 | | 811 | 6 | 6 |  | 0.0000 | 0.0000 | 0.0000 | 0.0000 | 0.0000 | 1.0000 | | 812 | 6 | 6 |  | 0.0000 | 0.0000 | 0.0000 | 0.0000 | 0.0001 | 0.9998 | | 813 | 6 | 6 |  | 0.0000 | 0.0000 | 0.0000 | 0.0000 | 0.0000 | 1.0000 | | 814 | 6 | 6 |  | 0.0001 | 0.0041 | 0.0012 | 0.0011 | 0.0007 | 0.9928 | | 815 | 6 | 6 |  | 0.0003 | 0.0005 | 0.0000 | 0.0003 | 0.0057 | 0.9931 | | 816 | 6 | 6 |  | 0.0000 | 0.0000 | 0.0000 | 0.0000 | 0.0000 | 0.9999 | | 817 | 6 | 6 |  | 0.0000 | 0.0000 | 0.0001 | 0.0001 | 0.0001 | 0.9998 | | 818 | 6 | 6 |  | 0.0000 | 0.0001 | 0.0000 | 0.0000 | 0.0000 | 0.9999 | | 819 | 6 | 6 |  | 0.0000 | 0.0000 | 0.0000 | 0.0000 | 0.0000 | 0.9999 | | 820 | 6 | 6 |  | 0.0000 | 0.0000 | 0.0000 | 0.0000 | 0.0000 | 1.0000 | | 821 | 6 | 6 |  | 0.0001 | 0.0001 | 0.0000 | 0.0000 | 0.0002 | 0.9996 | | 822 | 6 | 6 |  | 0.0000 | 0.0003 | 0.0000 | 0.0002 | 0.0000 | 0.9994 | | 823 | 6 | 6 |  | 0.0006 | 0.0098 | 0.0028 | 0.0028 | 0.0107 | 0.9733 | | 824 | 6 | 6 |  | 0.0000 | 0.0002 | 0.0000 | 0.0002 | 0.0001 | 0.9994 | | 825 | 6 | 6 |  | 0.0000 | 0.0000 | 0.0000 | 0.0000 | 0.0000 | 1.0000 | | 826 | 6 | 6 |  | 0.0000 | 0.0000 | 0.0000 | 0.0000 | 0.0000 | 0.9999 | | 827 | 6 | 6 |  | 0.0000 | 0.0000 | 0.0000 | 0.0000 | 0.0000 | 1.0000 | | 828 | 6 | 6 |  | 0.0001 | 0.0034 | 0.0012 | 0.0011 | 0.0005 | 0.9936 | | 829 | 6 | 6 |  | 0.0000 | 0.0013 | 0.0003 | 0.0003 | 0.0000 | 0.9981 | | 830 | 6 | 6 |  | 0.0000 | 0.0000 | 0.0000 | 0.0000 | 0.0000 | 1.0000 | | 831 | 6 | 6 |  | 0.0000 | 0.0000 | 0.0001 | 0.0001 | 0.0000 | 0.9998 | | 832 | 6 | 6 |  | 0.0001 | 0.0056 | 0.0016 | 0.0015 | 0.0006 | 0.9906 | | 833 | 6 | 6 |  | 0.0000 | 0.0000 | 0.0000 | 0.0000 | 0.0001 | 0.9999 | | 834 | 6 | 6 |  | 0.0000 | 0.0003 | 0.0000 | 0.0002 | 0.0002 | 0.9993 | | 835 | 6 | 6 |  | 0.0007 | 0.0003 | 0.0000 | 0.0000 | 0.0090 | 0.9899 | | 836 | 6 | 6 |  | 0.0003 | 0.0004 | 0.0000 | 0.0000 | 0.0004 | 0.9988 | | 837 | 6 | 6 |  | 0.0000 | 0.0000 | 0.0000 | 0.0000 | 0.0000 | 1.0000 | | 838 | 6 | 6 |  | 0.0001 | 0.0035 | 0.0013 | 0.0011 | 0.0010 | 0.9930 | | 839 | 6 | 6 |  | 0.0000 | 0.0000 | 0.0000 | 0.0000 | 0.0000 | 1.0000 | | 840 | 6 | 6 |  | 0.0000 | 0.0002 | 0.0000 | 0.0001 | 0.0001 | 0.9995 | | 841 | 6 | 6 |  | 0.0000 | 0.0000 | 0.0000 | 0.0000 | 0.0000 | 1.0000 | | 842 | 6 | 6 |  | 0.0000 | 0.0000 | 0.0000 | 0.0000 | 0.0000 | 1.0000 | | 843 | 6 | 6 |  | 0.0000 | 0.0000 | 0.0000 | 0.0000 | 0.0000 | 0.9999 | | 844 | 6 | 6 |  | 0.0000 | 0.0000 | 0.0001 | 0.0001 | 0.0001 | 0.9998 | | 845 | 6 | 6 |  | 0.0000 | 0.0000 | 0.0000 | 0.0000 | 0.0000 | 1.0000 | | 846 | 6 | 6 |  | 0.0000 | 0.0016 | 0.0003 | 0.0005 | 0.0001 | 0.9976 | | 847 | 6 | 6 |  | 0.0000 | 0.0000 | 0.0000 | 0.0000 | 0.0000 | 0.9999 | | 848 | 6 | 6 |  | 0.0000 | 0.0000 | 0.0000 | 0.0000 | 0.0000 | 1.0000 | | 849 | 6 | 6 |  | 0.0012 | 0.0210 | 0.0052 | 0.0058 | 0.0147 | 0.9521 | | 850 | 6 | 6 |  | 0.0000 | 0.0000 | 0.0000 | 0.0000 | 0.0000 | 1.0000 | | 851 | 6 | 6 |  | 0.0002 | 0.0002 | 0.0000 | 0.0002 | 0.0000 | 0.9993 | | 852 | 6 | 6 |  | 0.0005 | 0.0003 | 0.0000 | 0.0000 | 0.0003 | 0.9989 | | 853 | 6 | 6 |  | 0.0000 | 0.0000 | 0.0000 | 0.0000 | 0.0000 | 1.0000 | | 854 | 6 | 6 |  | 0.0004 | 0.0005 | 0.0001 | 0.0004 | 0.0000 | 0.9986 | | 855 | 6 | 6 |  | 0.0005 | 0.0001 | 0.0000 | 0.0000 | 0.0007 | 0.9987 | | 856 | 6 | 6 |  | 0.0003 | 0.0003 | 0.0001 | 0.0002 | 0.0000 | 0.9990 | | 857 | 6 | 6 |  | 0.0000 | 0.0000 | 0.0000 | 0.0000 | 0.0000 | 1.0000 | | 858 | 6 | 6 |  | 0.0000 | 0.0001 | 0.0000 | 0.0000 | 0.0000 | 0.9999 | | 859 | 6 | 6 |  | 0.0000 | 0.0000 | 0.0000 | 0.0000 | 0.0000 | 1.0000 | | 860 | 6 | 6 |  | 0.0004 | 0.0002 | 0.0000 | 0.0000 | 0.0002 | 0.9992 | | 861 | 6 | 6 |  | 0.0000 | 0.0000 | 0.0000 | 0.0000 | 0.0000 | 1.0000 | | 862 | 6 | 6 |  | 0.0000 | 0.0000 | 0.0000 | 0.0000 | 0.0000 | 1.0000 | | 863 | 6 | 6 |  | 0.0000 | 0.0000 | 0.0000 | 0.0000 | 0.0000 | 1.0000 | | 864 | 6 | 6 |  | 0.0002 | 0.0001 | 0.0000 | 0.0001 | 0.0000 | 0.9995 | | 865 | 6 | 6 |  | 0.0003 | 0.0002 | 0.0000 | 0.0000 | 0.0002 | 0.9993 | | 866 | 6 | 6 |  | 0.0001 | 0.0001 | 0.0000 | 0.0001 | 0.0000 | 0.9997 | | 867 | 6 | 6 |  | 0.0000 | 0.0000 | 0.0000 | 0.0000 | 0.0000 | 1.0000 | | 868 | 6 | 6 |  | 0.0004 | 0.0005 | 0.0001 | 0.0002 | 0.0000 | 0.9987 | | 869 | 6 | 6 |  | 0.0000 | 0.0002 | 0.0000 | 0.0000 | 0.0000 | 0.9998 | | 870 | 6 | 6 |  | 0.0003 | 0.0004 | 0.0000 | 0.0000 | 0.0001 | 0.9992 | | 871 | 6 | 6 |  | 0.0000 | 0.0000 | 0.0000 | 0.0000 | 0.0000 | 1.0000 | | 872 | 6 | 6 |  | 0.0003 | 0.0003 | 0.0000 | 0.0001 | 0.0000 | 0.9992 | | 873 | 6 | 6 |  | 0.0008 | 0.0013 | 0.0002 | 0.0008 | 0.0000 | 0.9968 | | 874 | 6 | 6 |  | 0.0000 | 0.0000 | 0.0000 | 0.0000 | 0.0000 | 1.0000 | | 875 | 6 | 6 |  | 0.0000 | 0.0007 | 0.0004 | 0.0008 | 0.0000 | 0.9981 | | 876 | 6 | 6 |  | 0.0000 | 0.0000 | 0.0000 | 0.0000 | 0.0000 | 0.9999 | | 877 | 6 | 6 |  | 0.0000 | 0.0001 | 0.0001 | 0.0008 | 0.0000 | 0.9991 | | | --- | --- | --- | --- | --- | --- | --- | --- | --- | --- | --- | --- | --- | --- | --- | --- | --- | --- | --- | --- | --- | --- | --- | --- | --- | --- | --- | --- | --- | --- | --- | --- | --- | --- | --- | --- | --- | --- | --- | --- | --- | --- | --- | --- | --- | --- | --- | --- | --- | --- | --- | --- | --- | --- | --- | --- | --- | --- | --- | --- | --- | --- | --- | --- | --- | --- | --- | --- | --- | --- | --- | --- | --- | --- | --- | --- | --- | --- | --- | --- | --- | --- | --- | --- | --- | --- | --- | --- | --- | --- | --- | --- | --- | --- | --- | --- | --- | --- | --- | --- | --- | --- | --- | --- | --- | --- | --- | --- | --- | --- | --- | --- | --- | --- | --- | --- | --- | --- | --- | --- | --- | --- | --- | --- | --- | --- | --- | --- | --- | --- | --- | --- | --- | --- | --- | --- | --- | --- | --- | --- | --- | --- | --- | --- | --- | --- | --- | --- | --- | --- | --- | --- | --- | --- | --- | --- | --- | --- | --- | --- | --- | --- | --- | --- | --- | --- | --- | --- | --- | --- | --- | --- | --- | --- | --- | --- | --- | --- | --- | --- | --- | --- | --- | --- | --- | --- | --- | --- | --- | --- | --- | --- | --- | --- | --- | --- | --- | --- | --- | --- | --- | --- | --- | --- | --- | --- | --- | --- | --- | --- | --- | --- | --- | --- | --- | --- | --- | --- | --- | --- | --- | --- | --- | --- | --- | --- | --- | --- | --- | --- | --- | --- | --- | --- | --- | --- | --- | --- | --- | --- | --- | --- | --- | --- | --- | --- | --- | --- | --- | --- | --- | --- | --- | --- | --- | --- | --- | --- | --- | --- | --- | --- | --- | --- | --- | --- | --- | --- | --- | --- | --- | --- | --- | --- | --- | --- | --- | --- | --- | --- | --- | --- | --- | --- | --- | --- | --- | --- | --- | --- | --- | --- | --- | --- | --- | --- | --- | --- | --- | --- | --- | --- | --- | --- | --- | --- | --- | --- | --- | --- | --- | --- | --- | --- | --- | --- | --- | --- | --- | --- | --- | --- | --- | --- | --- | --- | --- | --- | --- | --- | --- | --- | --- | --- | --- | --- | --- | --- | --- | --- | --- | --- | --- | --- | --- | --- | --- | --- | --- | --- | --- | --- | --- | --- | --- | --- | --- | --- | --- | --- | --- | --- | --- | --- | --- | --- | --- | --- | --- | --- | --- | --- | --- | --- | --- | --- | --- | --- | --- | --- | --- | --- | --- | --- | --- | --- | --- | --- | --- | --- | --- | --- | --- | --- | --- | --- | --- | --- | --- | --- | --- | --- | --- | --- | --- | --- | --- | --- | --- | --- | --- | --- | --- | --- | --- | --- | --- | --- | --- | --- | --- | --- | --- | --- | --- | --- | --- | --- | --- | --- | --- | --- | --- | --- | --- | --- | --- | --- | --- | --- | --- | --- | --- | --- | --- | --- | --- | --- | --- | --- | --- | --- | --- | --- | --- | --- | --- | --- | --- | --- | --- | --- | --- | --- | --- | --- | --- | --- | --- | --- | --- | --- | --- | --- | --- | --- | --- | --- | --- | --- | --- | --- | --- | --- | --- | --- | --- | --- | --- | --- | --- | --- | --- | --- | --- | --- | --- | --- | --- | --- | --- | --- | --- | --- | --- | --- | --- | --- | --- | --- | --- | --- | --- | --- | --- | --- | --- | --- | --- | --- | --- | --- | --- | --- | --- | --- | --- | --- | --- | --- | --- | --- | --- | --- | --- | --- | --- | --- | --- | --- | --- | --- | --- | --- | --- | --- | --- | --- | --- | --- | --- | --- | --- | --- | --- | --- | --- | --- | --- | --- | --- | --- | --- | --- | --- | --- | --- | --- | --- | --- | --- | --- | --- | --- | --- | --- | --- | --- | --- | --- | --- | --- | --- | --- | --- | --- | --- | --- | --- | --- | --- | --- | --- | --- | --- | --- | --- | --- | --- | --- | --- | --- | --- | --- | --- | --- | --- | --- | --- | --- | --- | --- | --- | --- | --- | --- | --- | --- | --- | --- | --- | --- | --- | --- | --- | --- | --- | --- | --- | --- | --- | --- | --- | --- | --- | --- | --- | --- | --- | --- | --- | --- | --- | --- | --- | --- | --- | --- | --- | --- | --- | --- | --- | --- | --- | --- | --- | --- | --- | --- | --- | --- | --- | --- | --- | --- | --- | --- | --- | --- | --- | --- | --- | --- | --- | --- | --- | --- | --- | --- | --- | --- | --- | --- | --- | --- | --- | --- | --- | --- | --- | --- | --- | --- | --- | --- | --- | --- | --- | --- | --- | --- | --- | --- | --- | --- | --- | --- | --- | --- | --- | --- | --- | --- | --- | --- | --- | --- | --- | --- | --- | --- | --- | --- | --- | --- | --- | --- | --- | --- | --- | --- | --- | --- | --- | --- | --- | --- | --- | --- | --- | --- | --- | --- | --- | --- | --- | --- | --- | --- | --- | --- | --- | --- | --- | --- | --- | --- | --- | --- | --- | --- | --- | --- | --- | --- | --- | --- | --- | --- | --- | --- | --- | --- | --- | --- | --- | --- | --- | --- | --- | --- | --- | --- | --- | --- | --- | --- | --- | --- | --- | --- | --- | --- | --- | --- | --- | --- | --- | --- | --- | --- | --- | --- | --- | --- | --- | --- | --- | --- | --- | --- | --- | --- | --- | --- | --- | --- | --- | --- | --- | --- | --- | --- | --- | --- | --- | --- | --- | --- | --- | --- | --- | --- | --- | --- | --- | --- | --- | --- | --- | --- | --- | --- | --- | --- | --- | --- | --- | --- | --- | --- | --- | --- | --- | --- | --- | --- | --- | --- | --- | --- | --- | --- | --- | --- | --- | --- | --- | --- | --- | --- | --- | --- | --- | --- | --- | --- | --- | --- | --- | --- | --- | --- | --- | --- | --- | --- | --- | --- | --- | --- | --- | --- | --- | --- | --- | --- | --- | --- | --- | --- | --- | --- | --- | --- | --- | --- | --- | --- | --- | --- | --- | --- | --- | --- | --- | --- | --- | --- | --- | --- | --- | --- | --- | --- | --- | --- | --- | --- | --- | --- | --- | --- | --- | --- | --- | --- | --- | --- | --- | --- | --- | --- | --- | --- | --- | --- | --- | --- | --- | --- | --- | --- | --- | --- | --- | --- | --- | --- | --- | --- | --- | --- | --- | --- | --- | --- | --- | --- | --- | --- | --- | --- | --- | --- | --- | --- | --- | --- | --- | --- | --- | --- | --- | --- | --- | --- | --- | --- | --- | --- | --- | --- | --- | --- | --- | --- | --- | --- | --- | --- | --- | --- | --- | --- | --- | --- | --- | --- | --- | --- | --- | --- | --- | --- | --- | --- | --- | --- | --- | --- | --- | --- | --- | --- | --- | --- | --- | --- | --- | --- | --- | --- | --- | --- | --- | --- | --- | --- | --- | --- | --- | --- | --- | --- | --- | --- | --- | --- | --- | --- | --- | --- | --- | --- | --- | --- | --- | --- | --- | --- | --- | --- | --- | --- | --- | --- | --- | --- | --- | --- | --- | --- | --- | --- | --- | --- | --- | --- | --- | --- | --- | --- | --- | --- | --- | --- | --- | --- | --- | --- | --- | --- | --- | --- | --- | --- | --- | --- | --- | --- | --- | --- | --- | --- | --- | --- | --- | --- | --- | --- | --- | --- | --- | --- | --- | --- | --- | --- | --- | --- | --- | --- | --- | --- | --- | --- | --- | --- | --- | --- | --- | --- | --- | --- | --- | --- | --- | --- | --- | --- | --- | --- | --- | --- | --- | --- | --- | --- | --- | --- | --- | --- | --- | --- | --- | --- | --- | --- | --- | --- | --- | --- | --- | --- | --- | --- | --- | --- | --- | --- | --- | --- | --- | --- | --- | --- | --- | --- | --- | --- | --- | --- | --- | --- | --- | --- | --- | --- | --- | --- | --- | --- | --- | --- | --- | --- | --- | --- | --- | --- | --- | --- | --- | --- | --- | --- | --- | --- | --- | --- | --- | --- | --- | --- | --- | --- | --- | --- | --- | --- | --- | --- | --- | --- | --- | --- | --- | --- | --- | --- | --- | --- | --- | --- | --- | --- | --- | --- | --- | --- | --- | --- | --- | --- | --- | --- | --- | --- | --- | --- | --- | --- | --- | --- | --- | --- | --- | --- | --- | --- | --- | --- | --- | --- | --- | --- | --- | --- | --- | --- | --- | --- | --- | --- | --- | --- | --- | --- | --- | --- | --- | --- | --- | --- | --- | --- | --- | --- | --- | --- | --- | --- | --- | --- | --- | --- | --- | --- | --- | --- | --- | --- | --- | --- | --- | --- | --- | --- | --- | --- | --- | --- | --- | --- | --- | --- | --- | --- | --- | --- | --- | --- | --- | --- | --- | --- | --- | --- | --- | --- | --- | --- | --- | --- | --- | --- | --- | --- | --- | --- | --- | --- | --- | --- | --- | --- | --- | --- | --- | --- | --- | --- | --- | --- | --- | --- | --- | --- | --- | --- | --- | --- | --- | --- | --- | --- | --- | --- | --- | --- | --- | --- | --- | --- | --- | --- | --- | --- | --- | --- | --- | --- | --- | --- | --- | --- | --- | --- | --- | --- | --- | --- | --- | --- | --- | --- | --- | --- | --- | --- | --- | --- | --- | --- | --- | --- | --- | --- | --- | --- | --- | --- | --- | --- | --- | --- | --- | --- | --- | --- | --- | --- | --- | --- | --- | --- | --- | --- | --- | --- | --- | --- | --- | --- | --- | --- | --- | --- | --- | --- | --- | --- | --- | --- | --- | --- | --- | --- | --- | --- | --- | --- | --- | --- | --- | --- | --- | --- | --- | --- | --- | --- | --- | --- | --- | --- | --- | --- | --- | --- | --- | --- | --- | --- | --- | --- | --- | --- | --- | --- | --- | --- | --- | --- | --- | --- | --- | --- | --- | --- | --- | --- | --- | --- | --- | --- | --- | --- | --- | --- | --- | --- | --- | --- | --- | --- | --- | --- | --- | --- | --- | --- | --- | --- | --- | --- | --- | --- | --- | --- | --- | --- | --- | --- | --- | --- | --- | --- | --- | --- | --- | --- | --- | --- | --- | --- | --- | --- | --- | --- | --- | --- | --- | --- | --- | --- | --- | --- | --- | --- | --- | --- | --- | --- | --- | --- | --- | --- | --- | --- | --- | --- | --- | --- | --- | --- | --- | --- | --- | --- | --- | --- | --- | --- | --- | --- | --- | --- | --- | --- | --- | --- | --- | --- | --- | --- | --- | --- | --- | --- | --- | --- | --- | --- | --- | --- | --- | --- | --- | --- | --- | --- | --- | --- | --- | --- | --- | --- | --- | --- | --- | --- | --- | --- | --- | --- | --- | --- | --- | --- | --- | --- | --- | --- | --- | --- | --- | --- | --- | --- | --- | --- | --- | --- | --- | --- | --- | --- | --- | --- | --- | --- | --- | --- | --- | --- | --- | --- | --- | --- | --- | --- | --- | --- | --- | --- | --- | --- | --- | --- | --- | --- | --- | --- | --- | --- | --- | --- | --- | --- | --- | --- | --- | --- | --- | --- | --- | --- | --- | --- | --- | --- | --- | --- | --- | --- | --- | --- | --- | --- | --- | --- | --- | --- | --- | --- | --- | --- | --- | --- | --- | --- | --- | --- | --- | --- | --- | --- | --- | --- | --- | --- | --- | --- | --- | --- | --- | --- | --- | --- | --- | --- | --- | --- | --- | --- | --- | --- | --- | --- | --- | --- | --- | --- | --- | --- | --- | --- | --- | --- | --- | --- | --- | --- | --- | --- | --- | --- | --- | --- | --- | --- | --- | --- | --- | --- | --- | --- | --- | --- | --- | --- | --- | --- | --- | --- | --- | --- | --- | --- | --- | --- | --- | --- | --- | --- | --- | --- | --- | --- | --- | --- | --- | --- | --- | --- | --- | --- | --- | --- | --- | --- | --- | --- | --- | --- | --- | --- | --- | --- | --- | --- | --- | --- | --- | --- | --- | --- | --- | --- | --- | --- | --- | --- | --- | --- | --- | --- | --- | --- | --- | --- | --- | --- | --- | --- | --- | --- | --- | --- | --- | --- | --- | --- | --- | --- | --- | --- | --- | --- | --- | --- | --- | --- | --- | --- | --- | --- | --- | --- | --- | --- | --- | --- | --- | --- | --- | --- | --- | --- | --- | --- | --- | --- | --- | --- | --- | --- | --- | --- | --- | --- | --- | --- | --- | --- | --- | --- | --- | --- | --- | --- | --- | --- | --- | --- | --- | --- | --- | --- | --- | --- | --- | --- | --- | --- | --- | --- | --- | --- | --- | --- | --- | --- | --- | --- | --- | --- | --- | --- | --- | --- | --- | --- | --- | --- | --- | --- | --- | --- | --- | --- | --- | --- | --- | --- | --- | --- | --- | --- | --- | --- | --- | --- | --- | --- | --- | --- | --- | --- | --- | --- | --- | --- | --- | --- | --- | --- | --- | --- | --- | --- | --- | --- | --- | --- | --- | --- | --- | --- | --- | --- | --- | --- | --- | --- | --- | --- | --- | --- | --- | --- | --- | --- | --- | --- | --- | --- | --- | --- | --- | --- | --- | --- | --- | --- | --- | --- | --- | --- | --- | --- | --- | --- | --- | --- | --- | --- | --- | --- | --- | --- | --- | --- | --- | --- | --- | --- | --- | --- | --- | --- | --- | --- | --- | --- | --- | --- | --- | --- | --- | --- | --- | --- | --- | --- | --- | --- | --- | --- | --- | --- | --- | --- | --- | --- | --- | --- | --- | --- | --- | --- | --- | --- | --- | --- | --- | --- | --- | --- | --- | --- | --- | --- | --- | --- | --- | --- | --- | --- | --- | --- | --- | --- | --- | --- | --- | --- | --- | --- | --- | --- | --- | --- | --- | --- | --- | --- | --- | --- | --- | --- | --- | --- | --- | --- | --- | --- | --- | --- | --- | --- | --- | --- | --- | --- | --- | --- | --- | --- | --- | --- | --- | --- | --- | --- | --- | --- | --- | --- | --- | --- | --- | --- | --- | --- | --- | --- | --- | --- | --- | --- | --- | --- | --- | --- | --- | --- | --- | --- | --- | --- | --- | --- | --- | --- | --- | --- | --- | --- | --- | --- | --- | --- | --- | --- | --- | --- | --- | --- | --- | --- | --- | --- | --- | --- | --- | --- | --- | --- | --- | --- | --- | --- | --- | --- | --- | --- | --- | --- | --- | --- | --- | --- | --- | --- | --- | --- | --- | --- | --- | --- | --- | --- | --- | --- | --- | --- | --- | --- | --- | --- | --- | --- | --- | --- | --- | --- | --- | --- | --- | --- | --- | --- | --- | --- | --- | --- | --- | --- | --- | --- | --- | --- | --- | --- | --- | --- | --- | --- | --- | --- | --- | --- | --- | --- | --- | --- | --- | --- | --- | --- | --- | --- | --- | --- | --- | --- | --- | --- | --- | --- | --- | --- | --- | --- | --- | --- | --- | --- | --- | --- | --- | --- | --- | --- | --- | --- | --- | --- | --- | --- | --- | --- | --- | --- | --- | --- | --- | --- | --- | --- | --- | --- | --- | --- | --- | --- | --- | --- | --- | --- | --- | --- | --- | --- | --- | --- | --- | --- | --- | --- | --- | --- | --- | --- | --- | --- | --- | --- | --- | --- | --- | --- | --- | --- | --- | --- | --- | --- | --- | --- | --- | --- | --- | --- | --- | --- | --- | --- | --- | --- | --- | --- | --- | --- | --- | --- | --- | --- | --- | --- | --- | --- | --- | --- | --- | --- | --- | --- | --- | --- | --- | --- | --- | --- | --- | --- | --- | --- | --- | --- | --- | --- | --- | --- | --- | --- | --- | --- | --- | --- | --- | --- | --- | --- | --- | --- | --- | --- | --- | --- | --- | --- | --- | --- | --- | --- | --- | --- | --- | --- | --- | --- | --- | --- | --- | --- | --- | --- | --- | --- | --- | --- | --- | --- | --- | --- | --- | --- | --- | --- | --- | --- | --- | --- | --- | --- | --- | --- | --- | --- | --- | --- | --- | --- | --- | --- | --- | --- | --- | --- | --- | --- | --- | --- | --- | --- | --- | --- | --- | --- | --- | --- | --- | --- | --- | --- | --- | --- | --- | --- | --- | --- | --- | --- | --- | --- | --- | --- | --- | --- | --- | --- | --- | --- | --- | --- | --- | --- | --- | --- | --- | --- | --- | --- | --- | --- | --- | --- | --- | --- | --- | --- | --- | --- | --- | --- | --- | --- | --- | --- | --- | --- | --- | --- | --- | --- | --- | --- | --- | --- | --- | --- | --- | --- | --- | --- | --- | --- | --- | --- | --- | --- | --- | --- | --- | --- | --- | --- | --- | --- | --- | --- | --- | --- | --- | --- | --- | --- | --- | --- | --- | --- | --- | --- | --- | --- | --- | --- | --- | --- | --- | --- | --- | --- | --- | --- | --- | --- | --- | --- | --- | --- | --- | --- | --- | --- | --- | --- | --- | --- | --- | --- | --- | --- | --- | --- | --- | --- | --- | --- | --- | --- | --- | --- | --- | --- | --- | --- | --- | --- | --- | --- | --- | --- | --- | --- | --- | --- | --- | --- | --- | --- | --- | --- | --- | --- | --- | --- | --- | --- | --- | --- | --- | --- | --- | --- | --- | --- | --- | --- | --- | --- | --- | --- | --- | --- | --- | --- | --- | --- | --- | --- | --- | --- | --- | --- | --- | --- | --- | --- | --- | --- | --- | --- | --- | --- | --- | --- | --- | --- | --- | --- | --- | --- | --- | --- | --- | --- | --- | --- | --- | --- | --- | --- | --- | --- | --- | --- | --- | --- | --- | --- | --- | --- | --- | --- | --- | --- | --- | --- | --- | --- | --- | --- | --- | --- | --- | --- | --- | --- | --- | --- | --- | --- | --- | --- | --- | --- | --- | --- | --- | --- | --- | --- | --- | --- | --- | --- | --- | --- | --- | --- | --- | --- | --- | --- | --- | --- | --- | --- | --- | --- | --- | --- | --- | --- | --- | --- | --- | --- | --- | --- | --- | --- | --- | --- | --- | --- | --- | --- | --- | --- | --- | --- | --- | --- | --- | --- | --- | --- | --- | --- | --- | --- | --- | --- | --- | --- | --- | --- | --- | --- | --- | --- | --- | --- | --- | --- | --- | --- | --- | --- | --- | --- | --- | --- | --- | --- | --- | --- | --- | --- | --- | --- | --- | --- | --- | --- | --- | --- | --- | --- | --- | --- | --- | --- | --- | --- | --- | --- | --- | --- | --- | --- | --- | --- | --- | --- | --- | --- | --- | --- | --- | --- | --- | --- | --- | --- | --- | --- | --- | --- | --- | --- | --- | --- | --- | --- | --- | --- | --- | --- | --- | --- | --- | --- | --- | --- | --- | --- | --- | --- | --- | --- | --- | --- | --- | --- | --- | --- | --- | --- | --- | --- | --- | --- | --- | --- | --- | --- | --- | --- | --- | --- | --- | --- | --- | --- | --- | --- | --- | --- | --- | --- | --- | --- | --- | --- | --- | --- | --- | --- | --- | --- | --- | --- | --- | --- | --- | --- | --- | --- | --- | --- | --- | --- | --- | --- | --- | --- | --- | --- | --- | --- | --- | --- | --- | --- | --- | --- | --- | --- | --- | --- | --- | --- | --- | --- | --- | --- | --- | --- | --- | --- | --- | --- | --- | --- | --- | --- | --- | --- | --- | --- | --- | --- | --- | --- | --- | --- | --- | --- | --- | --- | --- | --- | --- | --- | --- | --- | --- | --- | --- | --- | --- | --- | --- | --- | --- | --- | --- | --- | --- | --- | --- | --- | --- | --- | --- | --- | --- | --- | --- | --- | --- | --- | --- | --- | --- | --- | --- | --- | --- | --- | --- | --- | --- | --- | --- | --- | --- | --- | --- | --- | --- | --- | --- | --- | --- | --- | --- | --- | --- | --- | --- | --- | --- | --- | --- | --- | --- | --- | --- | --- | --- | --- | --- | --- | --- | --- | --- | --- | --- | --- | --- | --- | --- | --- | --- | --- | --- | --- | --- | --- | --- | --- | --- | --- | --- | --- | --- | --- | --- | --- | --- | --- | --- | --- | --- | --- | --- | --- | --- | --- | --- | --- | --- | --- | --- | --- | --- | --- | --- | --- | --- | --- | --- | --- | --- | --- | --- | --- | --- | --- | --- | --- | --- | --- | --- | --- | --- | --- | --- | --- | --- | --- | --- | --- | --- | --- | --- | --- | --- | --- | --- | --- | --- | --- | --- | --- | --- | --- | --- | --- | --- | --- | --- | --- | --- | --- | --- | --- | --- | --- | --- | --- | --- | --- | --- | --- | --- | --- | --- | --- | --- | --- | --- | --- | --- | --- | --- | --- | --- | --- | --- | --- | --- | --- | --- | --- | --- | --- | --- | --- | --- | --- | --- | --- | --- | --- | --- | --- | --- | --- | --- | --- | --- | --- | --- | --- | --- | --- | --- | --- | --- | --- | --- | --- | --- | --- | --- | --- | --- | --- | --- | --- | --- | --- | --- | --- | --- | --- | --- | --- | --- | --- | --- | --- | --- | --- | --- | --- | --- | --- | --- | --- | --- | --- | --- | --- | --- | --- | --- | --- | --- | --- | --- | --- | --- | --- | --- | --- | --- | --- | --- | --- | --- | --- | --- | --- | --- | --- | --- | --- | --- | --- | --- | --- | --- | --- | --- | --- | --- | --- | --- | --- | --- | --- | --- | --- | --- | --- | --- | --- | --- | --- | --- | --- | --- | --- | --- | --- | --- | --- | --- | --- | --- | --- | --- | --- | --- | --- | --- | --- | --- | --- | --- | --- | --- | --- | --- | --- | --- | --- | --- | --- | --- | --- | --- | --- | --- | --- | --- | --- | --- | --- | --- | --- | --- | --- | --- | --- | --- | --- | --- | --- | --- | --- | --- | --- | --- | --- | --- | --- | --- | --- | --- | --- | --- | --- | --- | --- | --- | --- | --- | --- | --- | --- | --- | --- | --- | --- | --- | --- | --- | --- | --- | --- | --- | --- | --- | --- | --- | --- | --- | --- | --- | --- | --- | --- | --- | --- | --- | --- | --- | --- | --- | --- | --- | --- | --- | --- | --- | --- | --- | --- | --- | --- | --- | --- | --- | --- | --- | --- | --- | --- | --- | --- | --- | --- | --- | --- | --- | --- | --- | --- | --- | --- | --- | --- | --- | --- | --- | --- | --- | --- | --- | --- | --- | --- | --- | --- | --- | --- | --- | --- | --- | --- | --- | --- | --- | --- | --- | --- | --- | --- | --- | --- | --- | --- | --- | --- | --- | --- | --- | --- | --- | --- | --- | --- | --- | --- | --- | --- | --- | --- | --- | --- | --- | --- | --- | --- | --- | --- | --- | --- | --- | --- | --- | --- | --- | --- | --- | --- | --- | --- | --- | --- | --- | --- | --- | --- | --- | --- | --- | --- | --- | --- | --- | --- | --- | --- | --- | --- | --- | --- | --- | --- | --- | --- | --- | --- | --- | --- | --- | --- | --- | --- | --- | --- | --- | --- | --- | --- | --- | --- | --- | --- | --- | --- | --- | --- | --- | --- | --- | --- | --- | --- | --- | --- | --- | --- | --- | --- | --- | --- | --- | --- | --- | --- | --- | --- | --- | --- | --- | --- | --- | --- | --- | --- | --- | --- | --- | --- | --- | --- | --- | --- | --- | --- | --- | --- | --- | --- | --- | --- | --- | --- | --- | --- | --- | --- | --- | --- | --- | --- | --- | --- | --- | --- | --- | --- | --- | --- | --- | --- | --- | --- | --- | --- | --- | --- | --- | --- | --- | --- | --- | --- | --- | --- | --- | --- | --- | --- | --- | --- | --- | --- | --- | --- | --- | --- | --- | --- | --- | --- | --- | --- | --- | --- | --- | --- | --- | --- | --- | --- | --- | --- | --- | --- | --- | --- | --- | --- | --- | --- | --- | --- | --- | --- | --- | --- | --- | --- | --- | --- | --- | --- | --- | --- | --- | --- | --- | --- | --- | --- | --- | --- | --- | --- | --- | --- | --- | --- | --- | --- | --- | --- | --- | --- | --- | --- | --- | --- | --- | --- | --- | --- | --- | --- | --- | --- | --- | --- | --- | --- | --- | --- | --- | --- | --- | --- | --- | --- | --- | --- | --- | --- | --- | --- | --- | --- | --- | --- | --- | --- | --- | --- | --- | --- | --- | --- | --- | --- | --- | --- | --- | --- | --- | --- | --- | --- | --- | --- | --- | --- | --- | --- | --- | --- | --- | --- | --- | --- | --- | --- | --- | --- | --- | --- | --- | --- | --- | --- | --- | --- | --- | --- | --- | --- | --- | --- | --- | --- | --- | --- | --- | --- | --- | --- | --- | --- | --- | --- | --- | --- | --- | --- | --- | --- | --- | --- | --- | --- | --- | --- | --- | --- | --- | --- | --- | --- | --- | --- | --- | --- | --- | --- | --- | --- | --- | --- | --- | --- | --- | --- | --- | --- | --- | --- | --- | --- | --- | --- | --- | --- | --- | --- | --- | --- | --- | --- | --- | --- | --- | --- | --- | --- | --- | --- | --- | --- | --- | --- | --- | --- | --- | --- | --- | --- | --- | --- | --- | --- | --- | --- | --- | --- | --- | --- | --- | --- | --- | --- | --- | --- | --- | --- | --- | --- | --- | --- | --- | --- | --- | --- | --- | --- | --- | --- | --- | --- | --- | --- | --- | --- | --- | --- | --- | --- | --- | --- | --- | --- | --- | --- | --- | --- | --- | --- | --- | --- | --- | --- | --- | --- | --- | --- | --- | --- | --- | --- | --- | --- | --- | --- | --- | --- | --- | --- | --- | --- | --- | --- | --- | --- | --- | --- | --- | --- | --- | --- | --- | --- | --- | --- | --- | --- | --- | --- | --- | --- | --- | --- | --- | --- | --- | --- | --- | --- | --- | --- | --- | --- | --- | --- | --- | --- | --- | --- | --- | --- | --- | --- | --- | --- | --- | --- | --- | --- | --- | --- | --- | --- | --- | --- | --- | --- | --- | --- | --- | --- | --- | --- | --- | --- | --- | --- | --- | --- | --- | --- | --- | --- | --- | --- | --- | --- | --- | --- | --- | --- | --- | --- | --- | --- | --- | --- | --- | --- | --- | --- | --- | --- | --- | --- | --- | --- | --- | --- | --- | --- | --- | --- | --- | --- | --- | --- | --- | --- | --- | --- | --- | --- | --- | --- | --- | --- | --- | --- | --- | --- | --- | --- | --- | --- | --- | --- | --- | --- | --- | --- | --- | --- | --- | --- | --- | --- | --- | --- | --- | --- | --- | --- | --- | --- | --- | --- | --- | --- | --- | --- | --- | --- | --- | --- | --- | --- | --- | --- | --- | --- | --- | --- | --- | --- | --- | --- | --- | --- | --- | --- | --- | --- | --- | --- | --- | --- | --- | --- | --- | --- | --- | --- | --- | --- | --- | --- | --- | --- | --- | --- | --- | --- | --- | --- | --- | --- | --- | --- | --- | --- | --- | --- | --- | --- | --- | --- | --- | --- | --- | --- | --- | --- | --- | --- | --- | --- | --- | --- | --- | --- | --- | --- | --- | --- | --- | --- | --- | --- | --- | --- | --- | --- | --- | --- | --- | --- | --- | --- | --- | --- | --- | --- | --- | --- | --- | --- | --- | --- | --- | --- | --- | --- | --- | --- | --- | --- | --- | --- | --- | --- | --- | --- | --- | --- | --- | --- | --- | --- | --- | --- | --- | --- | --- | --- | --- | --- | --- | --- | --- | --- | --- | --- | --- | --- | --- | --- | --- | --- | --- | --- | --- | --- | --- | --- | --- | --- | --- | --- | --- | --- | --- | --- | --- | --- | --- | --- | --- | --- | --- | --- | --- | --- | --- | --- | --- | --- | --- | --- | --- | --- | --- | --- | --- | --- | --- | --- | --- | --- | --- | --- | --- | --- | --- | --- | --- | --- | --- | --- | --- | --- | --- | --- | --- | --- | --- | --- | --- | --- | --- | --- | --- | --- | --- | --- | --- | --- | --- | --- | --- | --- | --- | --- | --- | --- | --- | --- | --- | --- | --- | --- | --- | --- | --- | --- | --- | --- | --- | --- | --- | --- | --- | --- | --- | --- | --- | --- | --- | --- | --- | --- | --- | --- | --- | --- | --- | --- | --- | --- | --- | --- | --- | --- | --- | --- | --- | --- | --- | --- | --- | --- | --- | --- | --- | --- | --- | --- | --- | --- | --- | --- | --- | --- | --- | --- | --- | --- | --- | --- | --- | --- | --- | --- | --- | --- | --- | --- | --- | --- | --- | --- | --- | --- | --- | --- | --- | --- | --- | --- | --- | --- | --- | --- | --- | --- | --- | --- | --- | --- | --- | --- | --- | --- | --- | --- | --- | --- | --- | --- | --- | --- | --- | --- | --- | --- | --- | --- | --- | --- | --- | --- | --- | --- | --- | --- | --- | --- | --- | --- | --- | --- | --- | --- | --- | --- | --- | --- | --- | --- | --- | --- | --- | --- | --- | --- | --- | --- | --- | --- | --- | --- | --- | --- | --- | --- | --- | --- | --- | --- | --- | --- | --- | --- | --- | --- | --- | --- | --- | --- | --- | --- | --- | --- | --- | --- | --- | --- | --- | --- | --- | --- | --- | --- | --- | --- | --- | --- | --- | --- | --- | --- | --- | --- | --- | --- | --- | --- | --- | --- | --- | --- | --- | --- | --- | --- | --- | --- | --- | --- | --- | --- | --- | --- | --- | --- | --- | --- | --- | --- | --- | --- | --- | --- | --- | --- | --- | --- | --- | --- | --- | --- | --- | --- | --- | --- | --- | --- | --- | --- | --- | --- | --- | --- | --- | --- | --- | --- | --- | --- | --- | --- | --- | --- | --- | --- | --- | --- | --- | --- | --- | --- | --- | --- | --- | --- | --- | --- | --- | --- | --- | --- | --- | --- | --- | --- | --- | --- | --- | --- | --- | --- | --- | --- | --- | --- | --- | --- | --- | --- | --- | --- | --- | --- | --- | --- | --- | --- | --- | --- | --- | --- | --- | --- | --- | --- | --- | --- | --- | --- | --- | --- | --- | --- | --- | --- | --- | --- | --- | --- | --- | --- | --- | --- | --- | --- | --- | --- | --- | --- | --- | --- | --- | --- | --- | --- | --- | --- | --- | --- | --- | --- | --- | --- | --- | --- | --- | --- | --- | --- | --- | --- | --- | --- | --- | --- | --- | --- | --- | --- | --- | --- | --- | --- | --- | --- | --- | --- | --- | --- | --- | --- | --- | --- | --- | --- | --- | --- | --- | --- | --- | --- | --- | --- | --- | --- | --- | --- | --- | --- | --- | --- | --- | --- | --- | --- | --- | --- | --- | --- | --- | --- | --- | --- | --- | --- | --- | --- | --- | --- | --- | --- | --- | --- | --- | --- | --- | --- | --- | --- | --- | --- | --- | --- | --- | --- | --- | --- | --- | --- | --- | --- | --- | --- | --- | --- | --- | --- | --- | --- | --- | --- | --- | --- | --- | --- | --- | --- | --- | --- | --- | --- | --- | --- | --- | --- | --- | --- | --- | --- | --- | --- | --- | --- | --- | --- | --- | --- | --- | --- | --- | --- | --- | --- | --- | --- | --- | --- | --- | --- | --- | --- | --- | --- | --- | --- | --- | --- | --- | --- | --- | --- | --- | --- | --- | --- | --- | --- | --- | --- | --- | --- | --- | --- | --- | --- | --- | --- | --- | --- | --- | --- | --- | --- | --- | --- | --- | --- | --- | --- | --- | --- | --- | --- | --- | --- | --- | --- | --- | --- | --- | --- | --- | --- | --- | --- | --- | --- | --- | --- | --- | --- | --- | --- | --- | --- | --- | --- | --- | --- | --- | --- | --- | --- | --- | --- | --- | --- | --- | --- | --- | --- | --- | --- | --- | --- | --- | --- | --- | --- | --- | --- | --- | --- | --- | --- | --- | --- | --- | --- | --- | --- | --- | --- | --- | --- | --- | --- | --- | --- | --- | --- | --- | --- | --- | --- | --- | --- | --- | --- | --- | --- | --- | --- | --- | --- | --- | --- | --- | --- | --- | --- | --- | --- | --- | --- | --- | --- | --- | --- | --- | --- | --- | --- | --- | --- | --- | --- | --- | --- | --- | --- | --- | --- | --- | --- | --- | --- | --- | --- | --- | --- | --- | --- | --- | --- | --- | --- | --- | --- | --- | --- | --- | --- | --- | --- | --- | --- | --- | --- | --- | --- | --- | --- | --- | --- | --- | --- | --- | --- | --- | --- | --- | --- | --- | --- | --- | --- | --- | --- | --- | --- | --- | --- | --- | --- | --- | --- | --- | --- | --- | --- | --- | --- | --- | --- | --- | --- | --- | --- | --- | --- | --- | --- | --- | --- | --- | --- | --- | --- | --- | --- | --- | --- | --- | --- | --- | --- | --- | --- | --- | --- | --- | --- | --- | --- | --- | --- | --- | --- | --- | --- | --- | --- | --- | --- | --- | --- | --- | --- | --- | --- | --- | --- | --- | --- | --- | --- | --- | --- | --- | --- | --- | --- | --- | --- | --- | --- | --- | --- | --- | --- | --- | --- | --- | --- | --- | --- | --- | --- | --- | --- | --- | --- | --- | --- | --- | --- | --- | --- | --- | --- | --- | --- | --- | --- | --- | --- | --- | --- | --- | --- | --- | --- | --- | --- | --- | --- | --- | --- | --- | --- | --- | --- | --- | --- | --- | --- | --- | --- | --- | --- | --- | --- | --- | --- | --- | --- | --- | --- | --- | --- | --- | --- | --- | --- | --- | --- | --- | --- | --- | --- | --- | --- | --- | --- | --- | --- | --- | --- | --- | --- | --- | --- | --- | --- | --- | --- | --- | --- | --- | --- | --- | --- | --- | --- | --- | --- | --- | --- | --- | --- | --- | --- | --- | --- | --- | --- | --- | --- | --- | --- | --- | --- | --- | --- | --- | --- | --- | --- | --- | --- | --- | --- | --- | --- | --- | --- | --- | --- | --- | --- | --- | --- | --- | --- | --- | --- | --- | --- | --- | --- | --- | --- | --- | --- | --- | --- | --- | --- | --- | --- | --- | --- | --- | --- | --- | --- | --- | --- | --- | --- | --- | --- | --- | --- | --- | --- | --- | --- | --- | --- | --- | --- | --- | --- | --- | --- | --- | --- | --- | --- | --- | --- | --- | --- | --- | --- | --- | --- | --- | --- | --- | --- | --- | --- | --- | --- | --- | --- | --- | --- | --- | --- | --- | --- | --- | --- | --- | --- | --- | --- | --- | --- | --- | --- | --- | --- | --- | --- | --- | --- | --- | --- | --- | --- | --- | --- | --- | --- | --- | --- | --- | --- | --- | --- | --- | --- | --- | --- | --- | --- | --- | --- | --- | --- | --- | --- | --- | --- | --- | --- | --- | --- | --- | --- | --- | --- | --- | --- | --- | --- | --- | --- | --- | --- | --- | --- | --- | --- | --- | --- | --- | --- | --- | --- | --- | --- | --- | --- | --- | --- | --- | --- | --- | --- | --- | --- | --- | --- | --- | --- | --- | --- | --- | --- | --- | --- | --- | --- | --- | --- | --- | --- | --- | --- | --- | --- | --- | --- | --- | --- | --- | --- | --- | --- | --- | --- | --- | --- | --- | --- | --- | --- | --- | --- | --- | --- | --- | --- | --- | --- | --- | --- | --- | --- | --- | --- | --- | --- | --- | --- | --- | --- | --- | --- | --- | --- | --- | --- | --- | --- | --- | --- | --- | --- | --- | --- | --- | --- | --- | --- | --- | --- | --- | --- | --- | --- | --- | --- | --- | --- | --- | --- | --- | --- | --- | --- | --- | --- | --- | --- | --- | --- | --- | --- | --- | --- | --- | --- | --- | --- | --- | --- | --- | --- | --- | --- | --- | --- | --- | --- | --- | --- | --- | --- | --- | --- | --- | --- | --- | --- | --- | --- | --- | --- | --- | --- | --- | --- | --- | --- | --- | --- | --- | --- | --- | --- | --- | --- | --- | --- | --- | --- | --- | --- | --- | --- | --- | --- | --- | --- | --- | --- | --- | --- | --- | --- | --- | --- | --- | --- | --- | --- | --- | --- | --- | --- | --- | --- | --- | --- | --- | --- | --- | --- | --- | --- | --- | --- | --- | --- | --- | --- | --- | --- | --- | --- | --- | --- | --- | --- | --- | --- | --- | --- | --- | --- | --- | --- | --- | --- | --- | --- | --- | --- | --- | --- | --- | --- | --- | --- | --- | --- | --- | --- | --- | --- | --- | --- | --- | --- | --- | --- | --- | --- | --- | --- | --- | --- | --- | --- | --- | --- | --- | --- | --- | --- | --- | --- | --- | --- | --- | --- | --- | --- | --- | --- | --- | --- | --- | --- | --- | --- | --- | --- | --- | --- | --- | --- | --- | --- | --- | --- | --- | --- | --- | --- | --- | --- | --- | --- | --- | --- | --- | --- | --- | --- | --- | --- | --- | --- | --- | --- | --- | --- | --- | --- | --- | --- | --- | --- | --- | --- | --- | --- | --- | --- | --- | --- | --- | --- | --- | --- | --- | --- | --- | --- | --- | --- | --- | --- | --- | --- | --- | --- | --- | --- | --- | --- | --- | --- | --- | --- | --- | --- | --- | --- | --- | --- | --- | --- | --- | --- | --- | --- | --- | --- | --- | --- | --- | --- | --- | --- | --- | --- | --- | --- | --- | --- | --- | --- | --- | --- | --- | --- | --- | --- | --- | --- | --- | --- | --- | --- | --- | --- | --- | --- | --- | --- | --- | --- | --- | --- | --- | --- | --- | --- | --- | --- | --- | --- | --- | --- | --- | --- | --- | --- | --- | --- | --- | --- | --- | --- | --- | --- | --- | --- | --- | --- | --- | --- | --- | --- | --- | --- | --- | --- | --- | --- | --- | --- | --- | --- | --- | --- | --- | --- | --- | --- | --- | --- | --- | --- | --- | --- | --- | --- | --- | --- | --- | --- | --- | --- | --- | --- | --- | --- | --- | --- | --- | --- | --- | --- | --- | --- | --- | --- | --- | --- | --- | --- | --- | --- | --- | --- | --- | --- | --- | --- | --- | --- | --- | --- | --- | --- | --- | --- | --- | --- | --- | --- | --- | --- | --- | --- | --- | --- | --- | --- | --- | --- | --- | --- | --- | --- | --- | --- | --- | --- | --- | --- | --- | --- | --- | --- | --- | --- | --- | --- | --- | --- | --- | --- | --- | --- | --- | --- | --- | --- | --- | --- | --- | --- | --- | --- | --- | --- | --- | --- | --- | --- | --- | --- | --- | --- | --- | --- | --- | --- | --- | --- | --- | --- | --- | --- | --- | --- | --- | --- | --- | --- | --- | --- | --- | --- | --- | --- | --- | --- | --- | --- | --- | --- | --- | --- | --- | --- | --- | --- | --- | --- | --- | --- | --- | --- | --- | --- | --- | --- | --- | --- | --- | --- | --- | --- | --- | --- | --- | --- | --- | --- | --- | --- | --- | --- | --- | --- | --- | --- | --- | --- | --- | --- | --- | --- | --- | --- | --- | --- | --- | --- | --- | --- | --- | --- | --- | --- | --- | --- | --- | --- | --- | --- | --- | --- | --- | --- | --- | --- | --- | --- | --- | --- | --- | --- | --- | --- | --- | --- | --- | --- | --- | --- | --- | --- | --- | --- | --- | --- | --- | --- | --- | --- | --- | --- | --- | --- | --- | --- | --- | --- | --- | --- | --- | --- | --- | --- | --- | --- | --- | --- | --- | --- | --- | --- | --- | --- | --- | --- | --- | --- | --- | --- | --- | --- | --- | --- | --- | --- | --- | --- | --- | --- | --- | --- | --- | --- | --- | --- | --- | --- | --- | --- | --- | --- | --- | --- | --- | --- | --- | --- | --- | --- | --- | --- | --- | --- | --- | --- | --- | --- | --- | --- | --- | --- | --- | --- | --- | --- | --- | --- | --- | --- | --- | --- | --- | --- | --- | --- | --- | --- | --- | --- | --- | --- | --- | --- | --- | --- | --- | --- | --- | --- | --- | --- | --- | --- | --- | --- | --- | --- | --- | --- | --- | --- | --- | --- | --- | --- | --- | --- | --- | --- | --- | --- | --- | --- | --- | --- | --- | --- | --- | --- | --- | --- | --- | --- | --- | --- | --- | --- | --- | --- | --- | --- | --- | --- | --- | --- | --- | --- | --- | --- | --- | --- | --- | --- | --- | --- | --- | --- | --- | --- | --- | --- | --- | --- | --- | --- | --- | --- | --- | --- | --- | --- | --- | --- | --- | --- | --- | --- | --- | --- | --- | --- | --- | --- | --- | --- | --- | --- | --- | --- | --- | --- | --- | --- | --- | --- | --- | --- | --- | --- | --- | --- | --- | --- | --- | --- | --- | --- | --- | --- | --- | --- | --- | --- | --- | --- | --- | --- | --- | --- | --- | --- | --- | --- | --- | --- | --- | --- | --- | --- | --- | --- | --- | --- | --- | --- | --- | --- | --- | --- | --- | --- | --- | --- | --- | --- | --- | --- | --- | --- | --- | --- | --- | --- | --- | --- | --- | --- | --- | --- | --- | --- | --- | --- | --- | --- | --- | --- | --- | --- | --- | --- | --- | --- | --- | --- | --- | --- | --- | --- | --- | --- | --- | --- | --- | --- | --- | --- | --- | --- | --- | --- | --- | --- | --- | --- | --- | --- | --- | --- | --- | --- | --- | --- | --- | --- | --- | --- | --- | --- | --- | --- | --- | --- | --- | --- | --- | --- | --- | --- | --- | --- | --- | --- | --- | --- | --- | --- | --- | --- | --- | --- | --- | --- | --- | --- | --- | --- | --- | --- | --- | --- | --- | --- | --- | --- | --- | --- | --- | --- | --- | --- | --- | --- | --- | --- | --- | --- | --- | --- | --- | --- | --- | --- | --- | --- | --- | --- | --- | --- | --- | --- | --- | --- | --- | --- | --- | --- | --- | --- | --- | --- | --- | --- | --- | --- | --- | --- | --- | --- | --- | --- | --- | --- | --- | --- | --- | --- | --- | --- | --- | --- | --- | --- | --- | --- | --- | --- | --- | --- | --- | --- | --- | --- | --- | --- | --- | --- | --- | --- | --- | --- | --- | --- | --- | --- | --- | --- | --- | --- | --- | --- | --- | --- | --- | --- | --- | --- | --- | --- | --- | --- | --- | --- | --- | --- | --- | --- | --- | --- | --- | --- | --- | --- | --- | --- | --- | --- | --- | --- | --- | --- | --- | --- | --- | --- | --- | --- | --- | --- | --- | --- | --- | --- | --- | --- | --- | --- | --- | --- | --- | --- | --- | --- | --- | --- | --- | --- | --- | --- | --- | --- | --- | --- | --- | --- | --- | --- | --- | --- | --- | --- | --- | --- | --- | --- | --- | --- | --- | --- | --- | --- | --- | --- | --- | --- | --- | --- | --- | --- | --- | --- | --- | --- | --- | --- | --- | --- | --- | --- | --- | --- | --- | --- | --- | --- | --- | --- | --- | --- | --- | --- | --- | --- | --- | --- | --- | --- | --- | --- | --- | --- | --- | --- | --- | --- | --- | --- | --- | --- | --- | --- | --- | --- | --- | --- | --- | --- | --- | --- | --- | --- | --- | --- | --- | --- | --- | --- | --- | --- | --- | --- | --- | --- | --- | --- | --- | --- | --- | --- | --- | --- | --- | --- | --- | --- | --- | --- | --- | --- | --- | --- | --- | --- | --- | --- | --- | --- | --- | --- | --- | --- | --- | --- | --- | --- | --- | --- | --- | --- | --- | --- | --- | --- | --- | --- | --- | --- | --- | --- | --- | --- | --- | --- | --- | --- | --- | --- | --- | --- | --- | --- | --- | --- | --- | --- | --- | --- | --- | --- | --- | --- | --- | --- | --- | --- | --- | --- | --- | --- | --- | --- | --- | --- | --- | --- | --- | --- | --- | --- | --- | --- | --- | --- | --- | --- | --- | --- | --- | --- | --- | --- | --- | --- | --- | --- | --- | --- | --- | --- | --- | --- | --- | --- | --- | --- | --- | --- | --- | --- | --- | --- | --- | --- | --- | --- | --- | --- | --- | --- | --- | --- | --- | --- | --- | --- | --- | --- | --- | --- | --- | --- | --- | --- | --- | --- | --- | --- | --- | --- | --- | --- | --- | --- | --- | --- | --- | --- | --- | --- | --- | --- | --- | --- | --- | --- | --- | --- | --- | --- | --- | --- | --- | --- | --- | --- | --- | --- | --- | --- | --- | --- | --- | --- | --- | --- | --- | --- | --- | --- | --- | --- | --- | --- | --- | --- | --- | --- | --- | --- | --- | --- | --- | --- | --- | --- | --- | --- | --- | --- | --- | --- | --- | --- | --- | --- | --- | --- | --- | --- | --- | --- | --- | --- | --- | --- | --- | --- | --- | --- | --- | --- | --- | --- | --- | --- | --- | --- | --- | --- | --- | --- | --- | --- | --- | --- | --- | --- | --- | --- | --- | --- | --- | --- | --- | --- | --- | --- | --- | --- | --- | --- | --- | --- | --- | --- | --- | --- | --- | --- | --- | --- | --- | --- | --- | --- | --- | --- | --- | --- | --- | --- | --- | --- | --- | --- | --- | --- | --- | --- | --- | --- | --- | --- | --- | --- | --- | --- | --- | --- | --- | --- | --- | --- | --- | --- | --- | --- | --- | --- | --- | --- | --- | --- | --- | --- | --- | --- | --- | --- | --- | --- | --- | --- | --- | --- | --- | --- | --- | --- | --- | --- | --- | --- | --- | --- | --- | --- | --- | --- | --- | --- | --- | --- | --- | --- | --- | --- | --- | --- | --- | --- | --- | --- | --- | --- | --- | --- | --- | --- | --- | --- | --- | --- | --- | --- | --- | --- | --- | --- | --- | --- | --- | --- | --- | --- | --- | --- | --- | --- | --- | --- | --- | --- | --- | --- | --- | --- | --- | --- | --- | --- | --- | --- | --- | --- | --- | --- | --- | --- | --- | --- | --- | --- | --- | --- | --- | --- | --- | --- | --- | --- | --- | --- | --- | --- | --- | --- | --- | --- | --- | --- | --- | --- | --- | --- | --- | --- | --- | --- | --- | --- | --- | --- | --- | --- | --- | --- | --- | --- | --- | --- | --- | --- | --- | --- | --- | --- | --- | --- | --- | --- | --- | --- | --- | --- | --- | --- | --- | --- | --- | --- | --- | --- | --- | --- | --- | --- | --- | --- | --- | --- | --- | --- | --- | --- | --- | --- | --- | --- | --- | --- | --- | --- | --- | --- | --- | --- | --- | --- | --- | --- | --- | --- | --- | --- | --- | --- | --- | --- | --- | --- | --- | --- | --- | --- | --- | --- | --- | --- | --- | --- | --- | --- | --- | --- | --- | --- | --- | --- | --- | --- | --- | --- | --- | --- | --- | --- | --- | --- | --- | --- | --- | --- | --- | --- | --- | --- | --- | --- | --- | --- | --- | --- | --- | --- | --- | --- | --- | --- | --- | --- | --- | --- | --- | --- | --- | --- | --- | --- | --- | --- | --- | --- | --- | --- | --- | --- | --- | --- | --- | --- | --- | --- | --- | --- | --- | --- | --- | --- | --- | --- | --- | --- | --- | --- | --- | --- | --- | --- | --- | --- | --- | --- | --- | --- | --- | --- | --- | --- | --- | --- | --- | --- | --- | --- | --- | --- | --- | --- | --- | --- | --- | --- | --- | --- | --- | --- | --- | --- | --- | --- | --- | --- | --- | --- | --- | --- | --- | --- | --- | --- | --- | --- | --- | --- | --- | --- | --- | --- | --- | --- | --- | --- | --- | --- | --- | --- | --- | --- | --- | --- | --- | --- | --- | --- | --- | --- | --- | --- | --- | --- | --- | --- | --- | --- | --- | --- | --- | --- | --- | --- | --- | --- | --- | --- | --- | --- | --- | --- | --- | --- | --- | --- | --- | --- | --- | --- | --- | --- | --- | --- | --- | --- | --- | --- | --- | --- | --- | --- | --- | --- | --- | --- | --- | --- | --- | --- | --- | --- | --- | --- | --- | --- | --- | --- | --- | --- | --- | --- | --- | --- | --- | --- | --- | --- | --- | --- | --- | --- | --- | --- | --- | --- | --- | --- | --- | --- | --- | --- | --- | --- | --- | --- | --- | --- | --- | --- | --- | --- | --- | --- | --- | --- | --- | --- | --- | --- | --- | --- | --- | --- | --- | --- | --- | --- | --- | --- | --- | --- | --- | --- | --- | --- | --- | --- | --- | --- | --- | --- | --- | --- | --- | --- | --- | --- | --- | --- | --- | --- | --- | --- | --- | --- | --- | --- | --- | --- | --- | --- | --- | --- | --- | --- | --- | --- | --- | --- | --- | --- | --- | --- | --- | --- | --- | --- | --- | --- | --- | --- | --- | --- | --- | --- | --- | --- | --- | --- | --- | --- | --- | --- | --- | --- | --- | --- | --- | --- | --- | --- | --- | --- | --- | --- | --- | --- | --- | --- | --- | --- | --- | --- | --- | --- | --- | --- | --- | --- | --- | --- | --- | --- | --- | --- | --- | --- | --- | --- | --- | --- | --- | --- | --- | --- | --- | --- | --- | --- | --- | --- | --- | --- | --- | --- | --- | --- | --- | --- | --- | --- | --- | --- | --- | --- | --- | --- | --- | --- | --- | --- | --- | --- | --- | --- | --- | --- | --- | --- | --- | --- | --- | --- | --- | --- | --- | --- | --- | --- | --- | --- | --- | --- | --- | --- | --- | --- | --- | --- | --- | --- | --- | --- | --- | --- | --- | --- | --- | --- | --- | --- | --- | --- | --- | --- | --- | --- | --- | --- | --- | --- | --- | --- | --- | --- | --- | --- | --- | --- | --- | --- | --- | --- | --- | --- | --- | --- | --- | --- | --- | --- | --- | --- | --- | --- | --- | --- | --- | --- | --- | --- | --- | --- | --- | --- | --- | --- | --- | --- | --- | --- | --- | --- | --- | --- | --- | --- | --- | --- | --- | --- | --- | --- | --- | --- | --- | --- | --- | --- | --- | --- | --- | --- | --- | --- | --- | --- | --- | --- | --- | --- | --- | --- | --- | --- | --- | --- | --- | --- | --- | --- | --- | --- | --- | --- | --- | --- | --- | --- | --- | --- | --- | --- | --- | --- | --- | --- | --- | --- | --- | --- | --- | --- | --- | --- | --- | --- | --- | --- | --- | --- | --- | --- | --- | --- | --- | --- | --- | --- | --- | --- | --- | --- | --- | --- | --- | --- | --- | --- | --- | --- | --- | --- | --- | --- | --- | --- | --- | --- | --- | --- | --- | --- | --- | --- | --- | --- | --- | --- | --- | --- | --- | --- | --- | --- | --- | --- | --- | --- | --- | --- | --- | --- | --- | --- | --- | --- | --- | --- | --- | --- | --- | --- | --- | --- | --- | --- | --- | --- | --- | --- | --- | --- | --- | --- | --- | --- | --- | --- | --- | --- | --- | --- | --- | --- | --- | --- | --- | --- | --- | --- | --- | --- | --- | --- | --- | --- | --- | --- | --- | --- | --- | --- | --- | --- | --- | --- | --- | --- | --- | --- | --- | --- | --- | --- | --- | --- | --- | --- | --- | --- | --- | --- | --- | --- | --- | --- | --- | --- | --- | --- | --- | --- | --- | --- | --- | --- | --- | --- | --- | --- | --- | --- | --- | --- | --- | --- | --- | --- | --- | --- | --- | --- | --- | --- | --- | --- | --- | --- | --- | --- | --- | --- | --- | --- | --- | --- | --- | --- | --- | --- | --- | --- | --- | --- | --- | --- | --- | --- | --- | --- | --- | --- | --- | --- | --- | --- | --- | --- | --- | --- | --- | --- | --- | --- | --- | --- | --- | --- | --- | --- | --- | --- | --- | --- | --- | --- | --- | --- | --- | --- | --- | --- | --- | --- | --- | --- | --- | --- | --- | --- | --- | --- | --- | --- | --- | --- | --- | --- | --- | --- | --- | --- | --- | --- | --- | --- | --- | --- | --- | --- | --- | --- | --- | --- | --- | --- | --- | --- | --- | --- | --- | --- | --- | --- | --- | --- | --- | --- | --- | --- | --- | --- | --- | --- | --- | --- | --- | --- | --- | --- | --- | --- | --- | --- | --- | --- | --- | --- | --- | --- | --- | --- | --- | --- | --- | --- | --- | --- | --- | --- | --- | --- | --- | --- | --- | --- | --- | --- | --- | --- | --- | --- | --- | --- | --- | --- | --- | --- | --- | --- | --- | --- | --- | --- | --- | --- | --- | --- | --- | --- | --- | --- | --- | --- | --- | --- | --- | --- | --- | --- | --- | --- | --- | --- | --- | --- | --- | --- | --- | --- | --- | --- | --- | --- | --- | --- | --- | --- | --- | --- | --- | --- | --- | --- | --- | --- | --- | --- | --- | --- | --- | --- | --- | --- | --- | --- | --- | --- | --- | --- | --- | --- | --- | --- | --- | --- | --- | --- | --- | --- | --- | --- | --- | --- | --- | --- | --- | --- | --- | --- | --- | --- | --- | --- | --- | --- | --- | --- | --- | --- | --- | --- | --- | --- | --- | --- | --- | --- | --- | --- | --- | --- | --- | --- | --- | --- | --- | --- | --- | --- | --- | --- | --- | --- | --- | --- | --- | --- | --- | --- | --- | --- | --- | --- | --- | --- | --- | --- | --- | --- | --- | --- | --- | --- | --- | --- | --- | --- | --- | --- | --- | --- | --- | --- | --- | --- | --- | --- | --- | --- | --- | --- | --- | --- | --- | --- | --- | --- | --- | --- | --- | --- | --- | --- | --- | --- | --- | --- | --- | --- | --- | --- | --- | --- | --- | --- | --- | --- | --- | --- | --- | --- | --- | --- | --- | --- | --- | --- | --- | --- | --- | --- | --- | --- | --- | --- | --- | --- | --- | --- | --- | --- | --- | --- | --- | --- | --- | --- | --- | --- | --- | --- | --- | --- | --- | --- | --- | --- | --- | --- | --- | --- | --- | --- | --- | --- | --- | --- | --- | --- | --- | --- | --- | --- | --- | --- | --- | --- | --- | --- | --- | --- | --- | --- | --- | --- | --- | --- | --- | --- | --- | --- | --- | --- | --- | --- | --- | --- | --- | --- | --- | --- | --- | --- | --- | --- | --- | --- | --- | --- | --- | --- | --- | --- | --- | --- | --- | --- | --- | --- | --- | --- | --- | --- | --- | --- | --- | --- | --- | --- | --- | --- | --- | --- | --- | --- | --- | --- | --- | --- | --- | --- | --- | --- | --- | --- | --- | --- | --- | --- | --- | --- | --- | --- | --- | --- | --- | --- | --- | --- | --- | --- | --- | --- | --- | --- | --- | --- | --- | --- | --- | --- | --- | --- | --- | --- | --- | --- | --- | --- | --- | --- | --- | --- | --- | --- | --- | --- | --- | --- | --- | --- | --- | --- | --- | --- | --- | --- | --- | --- | --- | --- | --- | --- | --- | --- | --- | --- | --- | --- | --- | --- | --- | --- | --- | --- | --- | --- | --- | --- | --- | --- | --- | --- | --- | --- | --- | --- | --- | --- | --- | --- | --- | --- | --- | --- | --- | --- | --- | --- | --- | --- | --- | --- | --- | --- | --- | --- | --- | --- | --- | --- | --- | --- | --- | --- | --- | --- | --- | --- | --- | --- | --- | --- | --- | --- | --- | --- | --- | --- | --- | --- | --- | --- | --- | --- | --- | --- | --- | --- | --- | --- | --- | --- | --- | --- | --- | --- | --- | --- | --- | --- | --- | --- | --- | --- | --- | --- | --- | --- | --- | --- | --- | --- | --- | --- | --- | --- | --- | --- | --- | --- | --- | --- | --- | --- | --- | --- | --- | --- | --- | --- | --- | --- | --- | --- | --- | --- | --- | --- | --- | --- | --- | --- | --- | --- | --- | --- | --- | --- | --- | --- | --- | --- | --- | --- | --- | --- | --- | --- | --- | --- | --- | --- | --- | --- | --- | --- | --- | --- | --- | --- | --- | --- | --- | --- | --- | --- | --- | --- | --- | --- | --- | --- | --- | --- | --- | --- | --- | --- | --- | --- | --- | --- | --- | --- | --- | --- | --- | --- | --- | --- | --- | --- | --- | --- | --- | --- | --- | --- | --- | --- | --- | --- | --- | --- | --- | --- | --- | --- | --- | --- | --- | --- | --- | --- | --- | --- | --- | --- | --- | --- | --- | --- | --- | --- | --- | --- | --- | --- | --- | --- | --- | --- | --- | --- | --- | --- | --- | --- | --- | --- | --- | --- | --- | --- | --- | --- | --- | --- | --- | --- | --- | --- | --- | --- | --- | --- | --- | --- | --- | --- | --- | --- | --- | --- | --- | --- | --- | --- | --- | --- | --- | --- | --- | --- | --- | --- | --- | --- | --- | --- | --- | --- | --- | --- | --- | --- | --- | --- | --- | --- | --- | --- | --- | --- | --- | --- | --- | --- | --- | --- | --- | --- | --- | --- | --- | --- | --- | --- | --- | --- | --- | --- | --- | --- | --- | --- | --- | --- | --- | --- | --- | --- | --- | --- | --- | --- | --- | --- | --- | --- | --- | --- | --- | --- | --- | --- | --- | --- | --- | --- | --- | --- | --- | --- | --- | --- | --- | --- | --- | --- | --- | --- | --- | --- | --- | --- | --- | --- | --- | --- | --- | --- | --- | --- | --- | --- | --- | --- | --- | --- | --- | --- | --- | --- | --- | --- | --- | --- | --- | --- | --- | --- | --- | --- | --- | --- | --- | --- | --- | --- | --- | --- | --- | --- | --- | --- | --- | --- | --- | --- | --- | --- | --- | --- | --- | --- | --- | --- | --- | --- | --- | --- | --- | --- | --- | --- | --- | --- | --- | --- | --- | --- | --- | --- | --- | --- | --- | --- | --- | --- | --- | --- | --- | --- | --- | --- | --- | --- | --- | --- | --- | --- | --- | --- | --- | --- | --- | --- | --- | --- | --- | --- | --- | --- | --- | --- | --- | --- | --- | --- | --- | --- | --- | --- | --- | --- | --- | --- | --- | --- | --- | --- | --- | --- | --- | --- | --- | --- | --- | --- | --- | --- | --- | --- | --- | --- | --- | --- | --- | --- | --- | --- | --- | --- | --- | --- | --- | --- | --- | --- | --- | --- | --- | --- | --- | --- | --- | --- | --- | --- | --- | --- | --- | --- | --- | --- | --- | --- | --- | --- | --- | --- | --- | --- | --- | --- | --- | --- | --- | --- | --- | --- | --- | --- | --- | --- | --- | --- | --- | --- | --- | --- | --- | --- | --- | --- | --- | --- | --- | --- | --- | --- | --- | --- | --- | --- | --- | --- | --- | --- | --- | --- | --- | --- | --- | --- | --- | --- | --- | --- | --- | | | | | *** Misclassified observation** | | --- | | | --- | --- | | |  | | | | | | **Discriminant Analysis Results** | | --- | | | --- | --- | | | | **The DISCRIM Procedure Classification Summary for Calibration Data: WORK.SORTTEMPTABLESORTED Cross-validation Summary using Linear Discriminant Function** | | --- | | | --- | --- | | | | **Number of Observations and Percent Classified into DIST** | | | | | | | | | --- | --- | --- | --- | --- | --- | --- | --- | | **From DIST** | **1** | **2** | **3** | **4** | **5** | **6** | **Total** | | 1 | | 72 | | --- | | 43.37 | | | 31 | | --- | | 18.67 | | | 25 | | --- | | 15.06 | | | 14 | | --- | | 8.43 | | | 24 | | --- | | 14.46 | | | 0 | | --- | | 0.00 | | | 166 | | --- | | 100.00 | | | 2 | | 23 | | --- | | 14.47 | | | 99 | | --- | | 62.26 | | | 4 | | --- | | 2.52 | | | 24 | | --- | | 15.09 | | | 3 | | --- | | 1.89 | | | 6 | | --- | | 3.77 | | | 159 | | --- | | 100.00 | | | 3 | | 25 | | --- | | 19.08 | | | 27 | | --- | | 20.61 | | | 44 | | --- | | 33.59 | | | 26 | | --- | | 19.85 | | | 9 | | --- | | 6.87 | | | 0 | | --- | | 0.00 | | | 131 | | --- | | 100.00 | | | 4 | | 14 | | --- | | 9.15 | | | 28 | | --- | | 18.30 | | | 19 | | --- | | 12.42 | | | 86 | | --- | | 56.21 | | | 6 | | --- | | 3.92 | | | 0 | | --- | | 0.00 | | | 153 | | --- | | 100.00 | | | 5 | | 21 | | --- | | 14.38 | | | 18 | | --- | | 12.33 | | | 6 | | --- | | 4.11 | | | 11 | | --- | | 7.53 | | | 88 | | --- | | 60.27 | | | 2 | | --- | | 1.37 | | | 146 | | --- | | 100.00 | | | 6 | | 1 | | --- | | 0.82 | | | 8 | | --- | | 6.56 | | | 5 | | --- | | 4.10 | | | 1 | | --- | | 0.82 | | | 11 | | --- | | 9.02 | | | 96 | | --- | | 78.69 | | | 122 | | --- | | 100.00 | | | Total | | 156 | | --- | | 17.79 | | | 211 | | --- | | 24.06 | | | 103 | | --- | | 11.74 | | | 162 | | --- | | 18.47 | | | 141 | | --- | | 16.08 | | | 104 | | --- | | 11.86 | | | 877 | | --- | | 100.00 | | | Priors | | 0.18928 | | --- | |  | | | 0.1813 | | --- | |  | | | 0.14937 | | --- | |  | | | 0.17446 | | --- | |  | | | 0.16648 | | --- | |  | | | 0.13911 | | --- | |  | | |  | | --- | |  | | | | --- | --- | --- | --- | --- | --- | --- | --- | --- | --- | --- | --- | --- | --- | --- | --- | --- | --- | --- | --- | --- | --- | --- | --- | --- | --- | --- | --- | --- | --- | --- | --- | --- | --- | --- | --- | --- | --- | --- | --- | --- | --- | --- | --- | --- | --- | --- | --- | --- | --- | --- | --- | --- | --- | --- | --- | --- | --- | --- | --- | --- | --- | --- | --- | --- | --- | --- | --- | --- | --- | --- | --- | --- | --- | --- | --- | --- | --- | --- | --- | --- | --- | --- | --- | --- | --- | --- | --- | --- | --- | --- | --- | --- | --- | --- | --- | --- | --- | --- | --- | --- | --- | --- | --- | --- | --- | --- | --- | --- | --- | --- | --- | --- | --- | --- | --- | --- | --- | --- | --- | --- | --- | --- | --- | --- | --- | --- | --- | --- | --- | --- | --- | --- | --- | --- | --- | --- | --- | --- | --- | --- | --- | --- | --- | --- | --- | --- | --- | --- | --- | --- | --- | --- | --- | --- | --- | --- | --- | --- | --- | --- | --- | --- | --- | --- | --- | --- | --- | --- | --- | --- | --- | --- | --- | --- | --- | --- | --- | --- | --- | --- | --- | --- | --- | --- | --- | --- | --- | --- | --- | --- | --- | --- | | | **Error Count Estimates for DIST** | | | | | | | | | --- | --- | --- | --- | --- | --- | --- | --- | |  | **1** | **2** | **3** | **4** | **5** | **6** | **Total** | | Rate | 0.5663 | 0.3774 | 0.6641 | 0.4379 | 0.3973 | 0.2131 | 0.4470 | | Priors | 0.1893 | 0.1813 | 0.1494 | 0.1745 | 0.1665 | 0.1391 |  | | | |  | | | | | | **Discriminant Analysis Results** | | --- | | | --- | --- | | | | **The DISCRIM Procedure Classification Results for Calibration Data: WORK.SORTTEMPTABLESORTED Cross-validation Results using Linear Discriminant Function** | | --- | | | --- | --- | | | | **Number of Observations and Average Posterior Probabilities Classified into DIST** | | | | | | | | --- | --- | --- | --- | --- | --- | --- | | **From DIST** | **1** | **2** | **3** | **4** | **5** | **6** | | 1 | | 72 | | --- | | 0.5348 | | | 31 | | --- | | 0.4833 | | | 25 | | --- | | 0.4084 | | | 14 | | --- | | 0.4226 | | | 24 | | --- | | 0.5316 | | | 0 | | --- | | . | | | 2 | | 23 | | --- | | 0.3907 | | | 99 | | --- | | 0.5826 | | | 4 | | --- | | 0.3022 | | | 24 | | --- | | 0.5058 | | | 3 | | --- | | 0.5416 | | | 6 | | --- | | 0.4469 | | | 3 | | 25 | | --- | | 0.5401 | | | 27 | | --- | | 0.4215 | | | 44 | | --- | | 0.4815 | | | 26 | | --- | | 0.5289 | | | 9 | | --- | | 0.3858 | | | 0 | | --- | | . | | | 4 | | 14 | | --- | | 0.4008 | | | 28 | | --- | | 0.4323 | | | 19 | | --- | | 0.4064 | | | 86 | | --- | | 0.5227 | | | 6 | | --- | | 0.5580 | | | 0 | | --- | | . | | | 5 | | 21 | | --- | | 0.4775 | | | 18 | | --- | | 0.4313 | | | 6 | | --- | | 0.3947 | | | 11 | | --- | | 0.5717 | | | 88 | | --- | | 0.7161 | | | 2 | | --- | | 0.4135 | | | 6 | | 1 | | --- | | 0.4852 | | | 8 | | --- | | 0.6021 | | | 5 | | --- | | 0.3590 | | | 1 | | --- | | 0.3261 | | | 11 | | --- | | 0.6883 | | | 96 | | --- | | 0.9216 | | | Total | | 156 | | --- | | 0.4944 | | | 211 | | --- | | 0.5153 | | | 103 | | --- | | 0.4319 | | | 162 | | --- | | 0.5147 | | | 141 | | --- | | 0.6510 | | | 104 | | --- | | 0.8844 | | | Priors | | 0.18928 | | --- | |  | | | 0.1813 | | --- | |  | | | 0.14937 | | --- | |  | | | 0.17446 | | --- | |  | | | 0.16648 | | --- | |  | | | 0.13911 | | --- | |  | | | | --- | --- | --- | --- | --- | --- | --- | --- | --- | --- | --- | --- | --- | --- | --- | --- | --- | --- | --- | --- | --- | --- | --- | --- | --- | --- | --- | --- | --- | --- | --- | --- | --- | --- | --- | --- | --- | --- | --- | --- | --- | --- | --- | --- | --- | --- | --- | --- | --- | --- | --- | --- | --- | --- | --- | --- | --- | --- | --- | --- | --- | --- | --- | --- | --- | --- | --- | --- | --- | --- | --- | --- | --- | --- | --- | --- | --- | --- | --- | --- | --- | --- | --- | --- | --- | --- | --- | --- | --- | --- | --- | --- | --- | --- | --- | --- | --- | --- | --- | --- | --- | --- | --- | --- | --- | --- | --- | --- | --- | --- | --- | --- | --- | --- | --- | --- | --- | --- | --- | --- | --- | --- | --- | --- | --- | --- | --- | --- | --- | --- | --- | --- | --- | --- | --- | --- | --- | --- | --- | --- | --- | --- | --- | --- | --- | --- | --- | --- | --- | --- | --- | --- | --- | --- | --- | --- | --- | --- | --- | --- | --- | --- | --- | --- | --- | --- | --- | | | **Posterior Probability Error Rate Estimates for DIST** | | | | | | | | | --- | --- | --- | --- | --- | --- | --- | --- | | **Estimate** | **1** | **2** | **3** | **4** | **5** | **6** | **Total** | | Stratified | 0.5354 | 0.3162 | 0.6604 | 0.4551 | 0.3713 | 0.2461 | 0.4328 | | Unstratified | 0.5354 | 0.3162 | 0.6604 | 0.4551 | 0.3713 | 0.2461 | 0.4328 | | Priors | 0.1893 | 0.1813 | 0.1494 | 0.1745 | 0.1665 | 0.1391 |  | | | |  | | | |
| --- | --- | --- | --- | --- | --- | --- | --- | --- | --- | --- | --- | --- | --- | --- | --- | --- | --- | --- | --- | --- | --- | --- | --- | --- | --- | --- | --- | --- | --- | --- | --- | --- | --- | --- | --- | --- | --- | --- | --- | --- | --- | --- | --- | --- | --- | --- | --- | --- | --- | --- | --- | --- | --- | --- | --- | --- | --- | --- | --- | --- | --- | --- | --- | --- | --- | --- | --- | --- | --- | --- | --- | --- | --- | --- | --- | --- | --- | --- | --- | --- | --- | --- | --- | --- | --- | --- | --- | --- | --- | --- | --- | --- | --- | --- | --- | --- | --- | --- | --- | --- | --- | --- | --- | --- | --- | --- | --- | --- | --- | --- | --- | --- | --- | --- | --- | --- | --- | --- | --- | --- | --- | --- | --- | --- | --- | --- | --- | --- | --- | --- | --- | --- | --- | --- | --- | --- | --- | --- | --- | --- | --- | --- | --- | --- | --- | --- | --- | --- | --- | --- | --- | --- | --- | --- | --- | --- | --- | --- | --- | --- | --- | --- | --- | --- | --- | --- | --- | --- | --- | --- | --- | --- | --- | --- | --- | --- | --- | --- | --- | --- | --- | --- | --- | --- | --- | --- | --- | --- | --- | --- | --- | --- | --- | --- | --- | --- | --- | --- | --- | --- | --- | --- | --- | --- | --- | --- | --- | --- | --- | --- | --- | --- | --- | --- | --- | --- | --- | --- | --- | --- | --- | --- | --- | --- | --- | --- | --- | --- | --- | --- | --- | --- | --- | --- | --- | --- | --- | --- | --- | --- | --- | --- | --- | --- | --- | --- | --- | --- | --- | --- | --- | --- | --- | --- | --- | --- | --- | --- | --- | --- | --- | --- | --- | --- | --- | --- | --- | --- | --- | --- | --- | --- | --- | --- | --- | --- | --- | --- | --- | --- | --- | --- | --- | --- | --- | --- | --- | --- | --- | --- | --- | --- | --- | --- | --- | --- | --- | --- | --- | --- | --- | --- | --- | --- | --- | --- | --- | --- | --- | --- | --- | --- | --- | --- | --- | --- | --- | --- | --- | --- | --- | --- | --- | --- | --- | --- | --- | --- | --- | --- | --- | --- | --- | --- | --- | --- | --- | --- | --- | --- | --- | --- | --- | --- | --- | --- | --- | --- | --- | --- | --- | --- | --- | --- | --- | --- | --- | --- | --- | --- | --- | --- | --- | --- | --- | --- | --- | --- | --- | --- | --- | --- | --- | --- | --- | --- | --- | --- | --- | --- | --- | --- | --- | --- | --- | --- | --- | --- | --- | --- | --- | --- | --- | --- | --- | --- | --- | --- | --- | --- | --- | --- | --- | --- | --- | --- | --- | --- | --- | --- | --- | --- | --- | --- | --- | --- | --- | --- | --- | --- | --- | --- | --- | --- | --- | --- | --- | --- | --- | --- | --- | --- | --- | --- | --- | --- | --- | --- | --- | --- | --- | --- | --- | --- | --- | --- | --- | --- | --- | --- | --- | --- | --- | --- | --- | --- | --- | --- | --- | --- | --- | --- | --- | --- | --- | --- | --- | --- | --- | --- | --- | --- | --- | --- | --- | --- | --- | --- | --- | --- | --- | --- | --- | --- | --- | --- | --- | --- | --- | --- | --- | --- | --- | --- | --- | --- | --- | --- | --- | --- | --- | --- | --- | --- | --- | --- | --- | --- | --- | --- | --- | --- | --- | --- | --- | --- | --- | --- | --- | --- | --- | --- | --- | --- | --- | --- | --- | --- | --- | --- | --- | --- | --- | --- | --- | --- | --- | --- | --- | --- | --- | --- | --- | --- | --- | --- | --- | --- | --- | --- | --- | --- | --- | --- | --- | --- | --- | --- | --- | --- | --- | --- | --- | --- | --- | --- | --- | --- | --- | --- | --- | --- | --- | --- | --- | --- | --- | --- | --- | --- | --- | --- | --- | --- | --- | --- | --- | --- | --- | --- | --- | --- | --- | --- | --- | --- | --- | --- | --- | --- | --- | --- | --- | --- | --- | --- | --- | --- | --- | --- | --- | --- | --- | --- | --- | --- | --- | --- | --- | --- | --- | --- | --- | --- | --- | --- | --- | --- | --- | --- | --- | --- | --- | --- | --- | --- | --- | --- | --- | --- | --- | --- | --- | --- | --- | --- | --- | --- | --- | --- | --- | --- | --- | --- | --- | --- | --- | --- | --- | --- | --- | --- | --- | --- | --- | --- | --- | --- | --- | --- | --- | --- | --- | --- | --- | --- | --- | --- | --- | --- | --- | --- | --- | --- | --- | --- | --- | --- | --- | --- | --- | --- | --- | --- | --- | --- | --- | --- | --- | --- | --- | --- | --- | --- | --- | --- | --- | --- | --- | --- | --- | --- | --- | --- | --- | --- | --- | --- | --- | --- | --- | --- | --- | --- | --- | --- | --- | --- | --- | --- | --- | --- | --- | --- | --- | --- | --- | --- | --- | --- | --- | --- | --- | --- | --- | --- | --- | --- | --- | --- | --- | --- | --- | --- | --- | --- | --- | --- | --- | --- | --- | --- | --- | --- | --- | --- | --- | --- | --- | --- | --- | --- | --- | --- | --- | --- | --- | --- | --- | --- | --- | --- | --- | --- | --- | --- | --- | --- | --- | --- | --- | --- | --- | --- | --- | --- | --- | --- | --- | --- | --- | --- | --- | --- | --- | --- | --- | --- | --- | --- | --- | --- | --- | --- | --- | --- | --- | --- | --- | --- | --- | --- | --- | --- | --- | --- | --- | --- | --- | --- | --- | --- | --- | --- | --- | --- | --- | --- | --- | --- | --- | --- | --- | --- | --- | --- | --- | --- | --- | --- | --- | --- | --- | --- | --- | --- | --- | --- | --- | --- | --- | --- | --- | --- | --- | --- | --- | --- | --- | --- | --- | --- | --- | --- | --- | --- | --- | --- | --- | --- | --- | --- | --- | --- | --- | --- | --- | --- | --- | --- | --- | --- | --- | --- | --- | --- | --- | --- | --- | --- | --- | --- | --- | --- | --- | --- | --- | --- | --- | --- | --- | --- | --- | --- | --- | --- | --- | --- | --- | --- | --- | --- | --- | --- | --- | --- | --- | --- | --- | --- | --- | --- | --- | --- | --- | --- | --- | --- | --- | --- | --- | --- | --- | --- | --- | --- | --- | --- | --- | --- | --- | --- | --- | --- | --- | --- | --- | --- | --- | --- | --- | --- | --- | --- | --- | --- | --- | --- | --- | --- | --- | --- | --- | --- | --- | --- | --- | --- | --- | --- | --- | --- | --- | --- | --- | --- | --- | --- | --- | --- | --- | --- | --- | --- | --- | --- | --- | --- | --- | --- | --- | --- | --- | --- | --- | --- | --- | --- | --- | --- | --- | --- | --- | --- | --- | --- | --- | --- | --- | --- | --- | --- | --- | --- | --- | --- | --- | --- | --- | --- | --- | --- | --- | --- | --- | --- | --- | --- | --- | --- | --- | --- | --- | --- | --- | --- | --- | --- | --- | --- | --- | --- | --- | --- | --- | --- | --- | --- | --- | --- | --- | --- | --- | --- | --- | --- | --- | --- | --- | --- | --- | --- | --- | --- | --- | --- | --- | --- | --- | --- | --- | --- | --- | --- | --- | --- | --- | --- | --- | --- | --- | --- | --- | --- | --- | --- | --- | --- | --- | --- | --- | --- | --- | --- | --- | --- | --- | --- | --- | --- | --- | --- | --- | --- | --- | --- | --- | --- | --- | --- | --- | --- | --- | --- | --- | --- | --- | --- | --- | --- | --- | --- | --- | --- | --- | --- | --- | --- | --- | --- | --- | --- | --- | --- | --- | --- | --- | --- | --- | --- | --- | --- | --- | --- | --- | --- | --- | --- | --- | --- | --- | --- | --- | --- | --- | --- | --- | --- | --- | --- | --- | --- | --- | --- | --- | --- | --- | --- | --- | --- | --- | --- | --- | --- | --- | --- | --- | --- | --- | --- | --- | --- | --- | --- | --- | --- | --- | --- | --- | --- | --- | --- | --- | --- | --- | --- | --- | --- | --- | --- | --- | --- | --- | --- | --- | --- | --- | --- | --- | --- | --- | --- | --- | --- | --- | --- | --- | --- | --- | --- | --- | --- | --- | --- | --- | --- | --- | --- | --- | --- | --- | --- | --- | --- | --- | --- | --- | --- | --- | --- | --- | --- | --- | --- | --- | --- | --- | --- | --- | --- | --- | --- | --- | --- | --- | --- | --- | --- | --- | --- | --- | --- | --- | --- | --- | --- | --- | --- | --- | --- | --- | --- | --- | --- | --- | --- | --- | --- | --- | --- | --- | --- | --- | --- | --- | --- | --- | --- | --- | --- | --- | --- | --- | --- | --- | --- | --- | --- | --- | --- | --- | --- | --- | --- | --- | --- | --- | --- | --- | --- | --- | --- | --- | --- | --- | --- | --- | --- | --- | --- | --- | --- | --- | --- | --- | --- | --- | --- | --- | --- | --- | --- | --- | --- | --- | --- | --- | --- | --- | --- | --- | --- | --- | --- | --- | --- | --- | --- | --- | --- | --- | --- | --- | --- | --- | --- | --- | --- | --- | --- | --- | --- | --- | --- | --- | --- | --- | --- | --- | --- | --- | --- | --- | --- | --- | --- | --- | --- | --- | --- | --- | --- | --- | --- | --- | --- | --- | --- | --- | --- | --- | --- | --- | --- | --- | --- | --- | --- | --- | --- | --- | --- | --- | --- | --- | --- | --- | --- | --- | --- | --- | --- | --- | --- | --- | --- | --- | --- | --- | --- | --- | --- | --- | --- | --- | --- | --- | --- | --- | --- | --- | --- | --- | --- | --- | --- | --- | --- | --- | --- | --- | --- | --- | --- | --- | --- | --- | --- | --- | --- | --- | --- | --- | --- | --- | --- | --- | --- | --- | --- | --- | --- | --- | --- | --- | --- | --- | --- | --- | --- | --- | --- | --- | --- | --- | --- | --- | --- | --- | --- | --- | --- | --- | --- | --- | --- | --- | --- | --- | --- | --- | --- | --- | --- | --- | --- | --- | --- | --- | --- | --- | --- | --- | --- | --- | --- | --- | --- | --- | --- | --- | --- | --- | --- | --- | --- | --- | --- | --- | --- | --- | --- | --- | --- | --- | --- | --- | --- | --- | --- | --- | --- | --- | --- | --- | --- | --- | --- | --- | --- | --- | --- | --- | --- | --- | --- | --- | --- | --- | --- | --- | --- | --- | --- | --- | --- | --- | --- | --- | --- | --- | --- | --- | --- | --- | --- | --- | --- | --- | --- | --- | --- | --- | --- | --- | --- | --- | --- | --- | --- | --- | --- | --- | --- | --- | --- | --- | --- | --- | --- | --- | --- | --- | --- | --- | --- | --- | --- | --- | --- | --- | --- | --- | --- | --- | --- | --- | --- | --- | --- | --- | --- | --- | --- | --- | --- | --- | --- | --- | --- | --- | --- | --- | --- | --- | --- | --- | --- | --- | --- | --- | --- | --- | --- | --- | --- | --- | --- | --- | --- | --- | --- | --- | --- | --- | --- | --- | --- | --- | --- | --- | --- | --- | --- | --- | --- | --- | --- | --- | --- | --- | --- | --- | --- | --- | --- | --- | --- | --- | --- | --- | --- | --- | --- | --- | --- | --- | --- | --- | --- | --- | --- | --- | --- | --- | --- | --- | --- | --- | --- | --- | --- | --- | --- | --- | --- | --- | --- | --- | --- | --- | --- | --- | --- | --- | --- | --- | --- | --- | --- | --- | --- | --- | --- | --- | --- | --- | --- | --- | --- | --- | --- | --- | --- | --- | --- | --- | --- | --- | --- | --- | --- | --- | --- | --- | --- | --- | --- | --- | --- | --- | --- | --- | --- | --- | --- | --- | --- | --- | --- | --- | --- | --- | --- | --- | --- | --- | --- | --- | --- | --- | --- | --- | --- | --- | --- | --- | --- | --- | --- | --- | --- | --- | --- | --- | --- | --- | --- | --- | --- | --- | --- | --- | --- | --- | --- | --- | --- | --- | --- | --- | --- | --- | --- | --- | --- | --- | --- | --- | --- | --- | --- | --- | --- | --- | --- | --- | --- | --- | --- | --- | --- | --- | --- | --- | --- | --- | --- | --- | --- | --- | --- | --- | --- | --- | --- | --- | --- | --- | --- | --- | --- | --- | --- | --- | --- | --- | --- | --- | --- | --- | --- | --- | --- | --- | --- | --- | --- | --- | --- | --- | --- | --- | --- | --- | --- | --- | --- | --- | --- | --- | --- | --- | --- | --- | --- | --- | --- | --- | --- | --- | --- | --- | --- | --- | --- | --- | --- | --- | --- | --- | --- | --- | --- | --- | --- | --- | --- | --- | --- | --- | --- | --- | --- | --- | --- | --- | --- | --- | --- | --- | --- | --- | --- | --- | --- | --- | --- | --- | --- | --- | --- | --- | --- | --- | --- | --- | --- | --- | --- | --- | --- | --- | --- | --- | --- | --- | --- | --- | --- | --- | --- | --- | --- | --- | --- | --- | --- | --- | --- | --- | --- | --- | --- | --- | --- | --- | --- | --- | --- | --- | --- | --- | --- | --- | --- | --- | --- | --- | --- | --- | --- | --- | --- | --- | --- | --- | --- | --- | --- | --- | --- | --- | --- | --- | --- | --- | --- | --- | --- | --- | --- | --- | --- | --- | --- | --- | --- | --- | --- | --- | --- | --- | --- | --- | --- | --- | --- | --- | --- | --- | --- | --- | --- | --- | --- | --- | --- | --- | --- | --- | --- | --- | --- | --- | --- | --- | --- | --- | --- | --- | --- | --- | --- | --- | --- | --- | --- | --- | --- | --- | --- | --- | --- | --- | --- | --- | --- | --- | --- | --- | --- | --- | --- | --- | --- | --- | --- | --- | --- | --- | --- | --- | --- | --- | --- | --- | --- | --- | --- | --- | --- | --- | --- | --- | --- | --- | --- | --- | --- | --- | --- | --- | --- | --- | --- | --- | --- | --- | --- | --- | --- | --- | --- | --- | --- | --- | --- | --- | --- | --- | --- | --- | --- | --- | --- | --- | --- | --- | --- | --- | --- | --- | --- | --- | --- | --- | --- | --- | --- | --- | --- | --- | --- | --- | --- | --- | --- | --- | --- | --- | --- | --- | --- | --- | --- | --- | --- | --- | --- | --- | --- | --- | --- | --- | --- | --- | --- | --- | --- | --- | --- | --- | --- | --- | --- | --- | --- | --- | --- | --- | --- | --- | --- | --- | --- | --- | --- | --- | --- | --- | --- | --- | --- | --- | --- | --- | --- | --- | --- | --- | --- | --- | --- | --- | --- | --- | --- | --- | --- | --- | --- | --- | --- | --- | --- | --- | --- | --- | --- | --- | --- | --- | --- | --- | --- | --- | --- | --- | --- | --- | --- | --- | --- | --- | --- | --- | --- | --- | --- | --- | --- | --- | --- | --- | --- | --- | --- | --- | --- | --- | --- | --- | --- | --- | --- | --- | --- | --- | --- | --- | --- | --- | --- | --- | --- | --- | --- | --- | --- | --- | --- | --- | --- | --- | --- | --- | --- | --- | --- | --- | --- | --- | --- | --- | --- | --- | --- | --- | --- | --- | --- | --- | --- | --- | --- | --- | --- | --- | --- | --- | --- | --- | --- | --- | --- | --- | --- | --- | --- | --- | --- | --- | --- | --- | --- | --- | --- | --- | --- | --- | --- | --- | --- | --- | --- | --- | --- | --- | --- | --- | --- | --- | --- | --- | --- | --- | --- | --- | --- | --- | --- | --- | --- | --- | --- | --- | --- | --- | --- | --- | --- | --- | --- | --- | --- | --- | --- | --- | --- | --- | --- | --- | --- | --- | --- | --- | --- | --- | --- | --- | --- | --- | --- | --- | --- | --- | --- | --- | --- | --- | --- | --- | --- | --- | --- | --- | --- | --- | --- | --- | --- | --- | --- | --- | --- | --- | --- | --- | --- | --- | --- | --- | --- | --- | --- | --- | --- | --- | --- | --- | --- | --- | --- | --- | --- | --- | --- | --- | --- | --- | --- | --- | --- | --- | --- | --- | --- | --- | --- | --- | --- | --- | --- | --- | --- | --- | --- | --- | --- | --- | --- | --- | --- | --- | --- | --- | --- | --- | --- | --- | --- | --- | --- | --- | --- | --- | --- | --- | --- | --- | --- | --- | --- | --- | --- | --- | --- | --- | --- | --- | --- | --- | --- | --- | --- | --- | --- | --- | --- | --- | --- | --- | --- | --- | --- | --- | --- | --- | --- | --- | --- | --- | --- | --- | --- | --- | --- | --- | --- | --- | --- | --- | --- | --- | --- | --- | --- | --- | --- | --- | --- | --- | --- | --- | --- | --- | --- | --- | --- | --- | --- | --- | --- | --- | --- | --- | --- | --- | --- | --- | --- | --- | --- | --- | --- | --- | --- | --- | --- | --- | --- | --- | --- | --- | --- | --- | --- | --- | --- | --- | --- | --- | --- | --- | --- | --- | --- | --- | --- | --- | --- | --- | --- | --- | --- | --- | --- | --- | --- | --- | --- | --- | --- | --- | --- | --- | --- | --- | --- | --- | --- | --- | --- | --- | --- | --- | --- | --- | --- | --- | --- | --- | --- | --- | --- | --- | --- | --- | --- | --- | --- | --- | --- | --- | --- | --- | --- | --- | --- | --- | --- | --- | --- | --- | --- | --- | --- | --- | --- | --- | --- | --- | --- | --- | --- | --- | --- | --- | --- | --- | --- | --- | --- | --- | --- | --- | --- | --- | --- | --- | --- | --- | --- | --- | --- | --- | --- | --- | --- | --- | --- | --- | --- | --- | --- | --- | --- | --- | --- | --- | --- | --- | --- | --- | --- | --- | --- | --- | --- | --- | --- | --- | --- | --- | --- | --- | --- | --- | --- | --- | --- | --- | --- | --- | --- | --- | --- | --- | --- | --- | --- | --- | --- | --- | --- | --- | --- | --- | --- | --- | --- | --- | --- | --- | --- | --- | --- | --- | --- | --- | --- | --- | --- | --- | --- | --- | --- | --- | --- | --- | --- | --- | --- | --- | --- | --- | --- | --- | --- | --- | --- | --- | --- | --- | --- | --- | --- | --- | --- | --- | --- | --- | --- | --- | --- | --- | --- | --- | --- | --- | --- | --- | --- | --- | --- | --- | --- | --- | --- | --- | --- | --- | --- | --- | --- | --- | --- | --- | --- | --- | --- | --- | --- | --- | --- | --- | --- | --- | --- | --- | --- | --- | --- | --- | --- | --- | --- | --- | --- | --- | --- | --- | --- | --- | --- | --- | --- | --- | --- | --- | --- | --- | --- | --- | --- | --- | --- | --- | --- | --- | --- | --- | --- | --- | --- | --- | --- | --- | --- | --- | --- | --- | --- | --- | --- | --- | --- | --- | --- | --- | --- | --- | --- | --- | --- | --- | --- | --- | --- | --- | --- | --- | --- | --- | --- | --- | --- | --- | --- | --- | --- | --- | --- | --- | --- | --- | --- | --- | --- | --- | --- | --- | --- | --- | --- | --- | --- | --- | --- | --- | --- | --- | --- | --- | --- | --- | --- | --- | --- | --- | --- | --- | --- | --- | --- | --- | --- | --- | --- | --- | --- | --- | --- | --- | --- | --- | --- | --- | --- | --- | --- | --- | --- | --- | --- | --- | --- | --- | --- | --- | --- | --- | --- | --- | --- | --- | --- | --- | --- | --- | --- | --- | --- | --- | --- | --- | --- | --- | --- | --- | --- | --- | --- | --- | --- | --- | --- | --- | --- | --- | --- | --- | --- | --- | --- | --- | --- | --- | --- | --- | --- | --- | --- | --- | --- | --- | --- | --- | --- | --- | --- | --- | --- | --- | --- | --- | --- | --- | --- | --- | --- | --- | --- | --- | --- | --- | --- | --- | --- | --- | --- | --- | --- | --- | --- | --- | --- | --- | --- | --- | --- | --- | --- | --- | --- | --- | --- | --- | --- | --- | --- | --- | --- | --- | --- | --- | --- | --- | --- | --- | --- | --- | --- | --- | --- | --- | --- | --- | --- | --- | --- | --- | --- | --- | --- | --- | --- | --- | --- | --- | --- | --- | --- | --- | --- | --- | --- | --- | --- | --- | --- | --- | --- | --- | --- | --- | --- | --- | --- | --- | --- | --- | --- | --- | --- | --- | --- | --- | --- | --- | --- | --- | --- | --- | --- | --- | --- | --- | --- | --- | --- | --- | --- | --- | --- | --- | --- | --- | --- | --- | --- | --- | --- | --- | --- | --- | --- | --- | --- | --- | --- | --- | --- | --- | --- | --- | --- | --- | --- | --- | --- | --- | --- | --- | --- | --- | --- | --- | --- | --- | --- | --- | --- | --- | --- | --- | --- | --- | --- | --- | --- | --- | --- | --- | --- | --- | --- | --- | --- | --- | --- | --- | --- | --- | --- | --- | --- | --- | --- | --- | --- | --- | --- | --- | --- | --- | --- | --- | --- | --- | --- | --- | --- | --- | --- | --- | --- | --- | --- | --- | --- | --- | --- | --- | --- | --- | --- | --- | --- | --- | --- | --- | --- | --- | --- | --- | --- | --- | --- | --- | --- | --- | --- | --- | --- | --- | --- | --- | --- | --- | --- | --- | --- | --- | --- | --- | --- | --- | --- | --- | --- | --- | --- | --- | --- | --- | --- | --- | --- | --- | --- | --- | --- | --- | --- | --- | --- | --- | --- | --- | --- | --- | --- | --- | --- | --- | --- | --- | --- | --- | --- | --- | --- | --- | --- | --- | --- | --- | --- | --- | --- | --- | --- | --- | --- | --- | --- | --- | --- | --- | --- | --- | --- | --- | --- | --- | --- | --- | --- | --- | --- | --- | --- | --- | --- | --- | --- | --- | --- | --- | --- | --- | --- | --- | --- | --- | --- | --- | --- | --- | --- | --- | --- | --- | --- | --- | --- | --- | --- | --- | --- | --- | --- | --- | --- | --- | --- | --- | --- | --- | --- | --- | --- | --- | --- | --- | --- | --- | --- | --- | --- | --- | --- | --- | --- | --- | --- | --- | --- | --- | --- | --- | --- | --- | --- | --- | --- | --- | --- | --- | --- | --- | --- | --- | --- | --- | --- | --- | --- | --- | --- | --- | --- | --- | --- | --- | --- | --- | --- | --- | --- | --- | --- | --- | --- | --- | --- | --- | --- | --- | --- | --- | --- | --- | --- | --- | --- | --- | --- | --- | --- | --- | --- | --- | --- | --- | --- | --- | --- | --- | --- | --- | --- | --- | --- | --- | --- | --- | --- | --- | --- | --- | --- | --- | --- | --- | --- | --- | --- | --- | --- | --- | --- | --- | --- | --- | --- | --- | --- | --- | --- | --- | --- | --- | --- | --- | --- | --- | --- | --- | --- | --- | --- | --- | --- | --- | --- | --- | --- | --- | --- | --- | --- | --- | --- | --- | --- | --- | --- | --- | --- | --- | --- | --- | --- | --- | --- | --- | --- | --- | --- | --- | --- | --- | --- | --- | --- | --- | --- | --- | --- | --- | --- | --- | --- | --- | --- | --- | --- | --- | --- | --- | --- | --- | --- | --- | --- | --- | --- | --- | --- | --- | --- | --- | --- | --- | --- | --- | --- | --- | --- | --- | --- | --- | --- | --- | --- | --- | --- | --- | --- | --- | --- | --- | --- | --- | --- | --- | --- | --- | --- | --- | --- | --- | --- | --- | --- | --- | --- | --- | --- | --- | --- | --- | --- | --- | --- | --- | --- | --- | --- | --- | --- | --- | --- | --- | --- | --- | --- | --- | --- | --- | --- | --- | --- | --- | --- | --- | --- | --- | --- | --- | --- | --- | --- | --- | --- | --- | --- | --- | --- | --- | --- | --- | --- | --- | --- | --- | --- | --- | --- | --- | --- | --- | --- | --- | --- | --- | --- | --- | --- | --- | --- | --- | --- | --- | --- | --- | --- | --- | --- | --- | --- | --- | --- | --- | --- | --- | --- | --- | --- | --- | --- | --- | --- | --- | --- | --- | --- | --- | --- | --- | --- | --- | --- | --- | --- | --- | --- | --- | --- | --- | --- | --- | --- | --- | --- | --- | --- | --- | --- | --- | --- | --- | --- | --- | --- | --- | --- | --- | --- | --- | --- | --- | --- | --- | --- | --- | --- | --- | --- | --- | --- | --- | --- | --- | --- | --- | --- | --- | --- | --- | --- | --- | --- | --- | --- | --- | --- | --- | --- | --- | --- | --- | --- | --- | --- | --- | --- | --- | --- | --- | --- | --- | --- | --- | --- | --- | --- | --- | --- | --- | --- | --- | --- | --- | --- | --- | --- | --- | --- | --- | --- | --- | --- | --- | --- | --- | --- | --- | --- | --- | --- | --- | --- | --- | --- | --- | --- | --- | --- | --- | --- | --- | --- | --- | --- | --- | --- | --- | --- | --- | --- | --- | --- | --- | --- | --- | --- | --- | --- | --- | --- | --- | --- | --- | --- | --- | --- | --- | --- | --- | --- | --- | --- | --- | --- | --- | --- | --- | --- | --- | --- | --- | --- | --- | --- | --- | --- | --- | --- | --- | --- | --- | --- | --- | --- | --- | --- | --- | --- | --- | --- | --- | --- | --- | --- | --- | --- | --- | --- | --- | --- | --- | --- | --- | --- | --- | --- | --- | --- | --- | --- | --- | --- | --- | --- | --- | --- | --- | --- | --- | --- | --- | --- | --- | --- | --- | --- | --- | --- | --- | --- | --- | --- | --- | --- | --- | --- | --- | --- | --- | --- | --- | --- | --- | --- | --- | --- | --- | --- | --- | --- | --- | --- | --- | --- | --- | --- | --- | --- | --- | --- | --- | --- | --- | --- | --- | --- | --- | --- | --- | --- | --- | --- | --- | --- | --- | --- | --- | --- | --- | --- | --- | --- | --- | --- | --- | --- | --- | --- | --- | --- | --- | --- | --- | --- | --- | --- | --- | --- | --- | --- | --- | --- | --- | --- | --- | --- | --- | --- | --- | --- | --- | --- | --- | --- | --- | --- | --- | --- | --- | --- | --- | --- | --- | --- | --- | --- | --- | --- | --- | --- | --- | --- | --- | --- | --- | --- | --- | --- | --- | --- | --- | --- | --- | --- | --- | --- | --- | --- | --- | --- | --- | --- | --- | --- | --- | --- | --- | --- | --- | --- | --- | --- | --- | --- | --- | --- | --- | --- | --- | --- | --- | --- | --- | --- | --- | --- | --- | --- | --- | --- | --- | --- | --- | --- | --- | --- | --- | --- | --- | --- | --- | --- | --- | --- | --- | --- | --- | --- | --- | --- | --- | --- | --- | --- | --- | --- | --- | --- | --- | --- | --- | --- | --- | --- | --- | --- | --- | --- | --- | --- | --- | --- | --- | --- | --- | --- | --- | --- | --- | --- | --- | --- | --- | --- | --- | --- | --- | --- | --- | --- | --- | --- | --- | --- | --- | --- | --- | --- | --- | --- | --- | --- | --- | --- | --- | --- | --- | --- | --- | --- | --- | --- | --- | --- | --- | --- | --- | --- | --- | --- | --- | --- | --- | --- | --- | --- | --- | --- | --- | --- | --- | --- | --- | --- | --- | --- | --- | --- | --- | --- | --- | --- | --- | --- | --- | --- | --- | --- | --- | --- | --- | --- | --- | --- | --- | --- | --- | --- | --- | --- | --- | --- | --- | --- | --- | --- | --- | --- | --- | --- | --- | --- | --- | --- | --- | --- | --- | --- | --- | --- | --- | --- | --- | --- | --- | --- | --- | --- | --- | --- | --- | --- | --- | --- | --- | --- | --- | --- | --- | --- | --- | --- | --- | --- | --- | --- | --- | --- | --- | --- | --- | --- | --- | --- | --- | --- | --- | --- | --- | --- | --- | --- | --- | --- | --- | --- | --- | --- | --- | --- | --- | --- | --- | --- | --- | --- | --- | --- | --- | --- | --- | --- | --- | --- | --- | --- | --- | --- | --- | --- | --- | --- | --- | --- | --- | --- | --- | --- | --- | --- | --- | --- | --- | --- | --- | --- | --- | --- | --- | --- | --- | --- | --- | --- | --- | --- | --- | --- | --- | --- | --- | --- | --- | --- | --- | --- | --- | --- | --- | --- | --- | --- | --- | --- | --- | --- | --- | --- | --- | --- | --- | --- | --- | --- | --- | --- | --- | --- | --- | --- | --- | --- | --- | --- | --- | --- | --- | --- | --- | --- | --- | --- | --- | --- | --- | --- | --- | --- | --- | --- | --- | --- | --- | --- | --- | --- | --- | --- | --- | --- | --- | --- | --- | --- | --- | --- | --- | --- | --- | --- | --- | --- | --- | --- | --- | --- | --- | --- | --- | --- | --- | --- | --- | --- | --- | --- | --- | --- | --- | --- | --- | --- | --- | --- | --- | --- | --- | --- | --- | --- | --- | --- | --- | --- | --- | --- | --- | --- | --- | --- | --- | --- | --- | --- | --- | --- | --- | --- | --- | --- | --- | --- | --- | --- | --- | --- | --- | --- | --- | --- | --- | --- | --- | --- | --- | --- | --- | --- | --- | --- | --- | --- | --- | --- | --- | --- | --- | --- | --- | --- | --- | --- | --- | --- | --- | --- | --- | --- | --- | --- | --- | --- | --- | --- | --- | --- | --- | --- | --- | --- | --- | --- | --- | --- | --- | --- | --- | --- | --- | --- | --- | --- | --- | --- | --- | --- | --- | --- | --- | --- | --- | --- | --- | --- | --- | --- | --- | --- | --- | --- | --- | --- | --- | --- | --- | --- | --- | --- | --- | --- | --- | --- | --- | --- | --- | --- | --- | --- | --- | --- | --- | --- | --- | --- | --- | --- | --- | --- | --- | --- | --- | --- | --- | --- | --- | --- | --- | --- | --- | --- | --- | --- | --- | --- | --- | --- | --- | --- | --- | --- | --- | --- | --- | --- | --- | --- | --- | --- | --- | --- | --- | --- | --- | --- | --- | --- | --- | --- | --- | --- | --- | --- | --- | --- | --- | --- | --- | --- | --- | --- | --- | --- | --- | --- | --- | --- | --- | --- | --- | --- | --- | --- | --- | --- | --- | --- | --- | --- | --- | --- | --- | --- | --- | --- | --- | --- | --- | --- | --- | --- | --- | --- | --- | --- | --- | --- | --- | --- | --- | --- | --- | --- | --- | --- | --- | --- | --- | --- | --- | --- | --- | --- | --- | --- | --- | --- | --- | --- | --- | --- | --- | --- | --- | --- | --- | --- | --- | --- | --- | --- | --- | --- | --- | --- | --- | --- | --- | --- | --- | --- | --- | --- | --- | --- | --- | --- | --- | --- | --- | --- | --- | --- | --- | --- | --- | --- | --- | --- | --- | --- | --- | --- | --- | --- | --- | --- | --- | --- | --- | --- | --- | --- | --- | --- | --- | --- | --- | --- | --- | --- | --- | --- | --- | --- | --- | --- | --- | --- | --- | --- | --- | --- | --- | --- | --- | --- | --- | --- | --- | --- | --- | --- | --- | --- | --- | --- | --- | --- | --- | --- | --- | --- | --- | --- | --- | --- | --- | --- | --- | --- | --- | --- | --- | --- | --- | --- | --- | --- | --- | --- | --- | --- | --- | --- | --- | --- | --- | --- | --- | --- | --- | --- | --- | --- | --- | --- | --- | --- | --- | --- | --- | --- | --- | --- | --- | --- | --- | --- | --- | --- | --- | --- | --- | --- | --- | --- | --- | --- | --- | --- | --- | --- | --- | --- | --- | --- | --- | --- | --- | --- | --- | --- | --- | --- | --- | --- | --- | --- | --- | --- | --- | --- | --- | --- | --- | --- | --- | --- | --- | --- | --- | --- | --- | --- | --- | --- | --- | --- | --- | --- | --- | --- | --- | --- | --- | --- | --- | --- | --- | --- | --- | --- | --- | --- | --- | --- | --- | --- | --- | --- | --- | --- | --- | --- | --- | --- | --- | --- | --- | --- | --- | --- | --- | --- | --- | --- | --- | --- | --- | --- | --- | --- | --- | --- | --- | --- | --- | --- | --- | --- | --- | --- | --- | --- | --- | --- | --- | --- | --- | --- | --- | --- | --- | --- | --- | --- | --- | --- | --- | --- | --- | --- | --- | --- | --- | --- | --- | --- | --- | --- | --- | --- | --- | --- | --- | --- | --- | --- | --- | --- | --- | --- | --- | --- | --- | --- | --- | --- | --- | --- | --- | --- | --- | --- | --- | --- | --- | --- | --- | --- | --- | --- | --- | --- | --- | --- | --- | --- | --- | --- | --- | --- | --- | --- | --- | --- | --- | --- | --- | --- | --- | --- | --- | --- | --- | --- | --- | --- | --- | --- | --- | --- | --- | --- | --- | --- | --- | --- | --- | --- | --- | --- | --- | --- | --- | --- | --- | --- | --- | --- | --- | --- | --- | --- | --- | --- | --- | --- | --- | --- | --- | --- | --- | --- | --- | --- | --- | --- | --- | --- | --- | --- | --- | --- | --- | --- | --- | --- | --- | --- | --- | --- | --- | --- | --- | --- | --- | --- | --- | --- | --- | --- | --- | --- | --- | --- | --- | --- | --- | --- | --- | --- | --- | --- | --- | --- | --- | --- | --- | --- | --- | --- | --- | --- | --- | --- | --- | --- | --- | --- | --- | --- | --- | --- | --- | --- | --- | --- | --- | --- | --- | --- | --- | --- | --- | --- | --- | --- | --- | --- | --- | --- | --- | --- | --- | --- | --- | --- | --- | --- | --- | --- | --- | --- | --- | --- | --- | --- | --- | --- | --- | --- | --- | --- | --- | --- | --- | --- | --- | --- | --- | --- | --- | --- | --- | --- | --- | --- | --- | --- | --- | --- | --- | --- | --- | --- | --- | --- | --- | --- | --- | --- | --- | --- | --- | --- | --- | --- | --- | --- | --- | --- | --- | --- | --- | --- | --- | --- | --- | --- | --- | --- | --- | --- | --- | --- | --- | --- | --- | --- | --- | --- | --- | --- | --- | --- | --- | --- | --- | --- | --- | --- | --- | --- | --- | --- | --- | --- | --- | --- | --- | --- | --- | --- | --- | --- | --- | --- | --- | --- | --- | --- | --- | --- | --- | --- | --- | --- | --- | --- | --- | --- | --- | --- | --- | --- | --- | --- | --- | --- | --- | --- | --- | --- | --- | --- | --- | --- | --- | --- | --- | --- | --- | --- | --- | --- | --- | --- | --- | --- | --- | --- | --- | --- | --- | --- | --- | --- | --- | --- | --- | --- | --- | --- | --- | --- | --- | --- | --- | --- | --- | --- | --- | --- | --- | --- | --- | --- | --- | --- | --- | --- | --- | --- | --- | --- | --- | --- | --- | --- | --- | --- | --- | --- | --- | --- | --- | --- | --- | --- | --- | --- | --- | --- | --- | --- | --- | --- | --- | --- | --- | --- | --- | --- | --- | --- | --- | --- | --- | --- | --- | --- | --- | --- | --- | --- | --- | --- | --- | --- | --- | --- | --- | --- | --- | --- | --- | --- | --- | --- | --- | --- | --- | --- | --- | --- | --- | --- | --- | --- | --- | --- | --- | --- | --- | --- | --- | --- | --- | --- | --- | --- | --- | --- | --- | --- | --- | --- | --- | --- | --- | --- | --- | --- | --- | --- | --- | --- | --- | --- | --- | --- | --- | --- | --- | --- | --- | --- | --- | --- | --- | --- | --- | --- | --- | --- | --- | --- | --- | --- | --- | --- | --- | --- | --- | --- | --- | --- | --- | --- | --- | --- | --- | --- | --- | --- | --- | --- | --- | --- | --- | --- | --- | --- | --- | --- | --- | --- | --- | --- | --- | --- | --- | --- | --- | --- | --- | --- | --- | --- | --- | --- | --- | --- | --- | --- | --- | --- | --- | --- | --- | --- | --- | --- | --- | --- | --- | --- | --- | --- | --- | --- | --- | --- | --- | --- | --- | --- | --- | --- | --- | --- | --- | --- | --- | --- | --- | --- | --- | --- | --- | --- | --- | --- | --- | --- | --- | --- | --- | --- | --- | --- | --- | --- | --- | --- | --- | --- | --- | --- | --- | --- | --- | --- | --- | --- | --- | --- | --- | --- | --- | --- | --- | --- | --- | --- | --- | --- | --- | --- | --- | --- | --- | --- | --- | --- | --- | --- | --- | --- | --- | --- | --- | --- | --- | --- | --- | --- | --- | --- | --- | --- | --- | --- | --- | --- | --- | --- | --- | --- | --- | --- | --- | --- | --- | --- | --- | --- | --- | --- | --- | --- | --- | --- | --- | --- | --- | --- | --- | --- | --- | --- | --- | --- | --- | --- | --- | --- | --- | --- | --- | --- | --- | --- | --- | --- | --- | --- | --- | --- | --- | --- | --- | --- | --- | --- | --- | --- | --- | --- | --- | --- | --- | --- | --- | --- | --- | --- | --- | --- | --- | --- | --- | --- | --- | --- | --- | --- | --- | --- | --- | --- | --- | --- | --- | --- | --- | --- | --- | --- | --- | --- | --- | --- | --- | --- | --- | --- | --- | --- | --- | --- | --- | --- | --- | --- | --- | --- | --- | --- | --- | --- | --- | --- | --- | --- | --- | --- | --- | --- | --- | --- | --- | --- | --- | --- | --- | --- | --- | --- | --- | --- | --- | --- | --- | --- | --- | --- | --- | --- | --- | --- | --- | --- | --- | --- | --- | --- | --- | --- | --- | --- | --- | --- | --- | --- | --- | --- | --- | --- | --- | --- | --- | --- | --- | --- | --- | --- | --- | --- | --- | --- | --- | --- | --- | --- | --- | --- | --- | --- | --- | --- | --- | --- | --- | --- | --- | --- | --- | --- | --- | --- | --- | --- | --- | --- | --- | --- | --- | --- | --- | --- | --- | --- | --- | --- | --- | --- | --- | --- | --- | --- | --- | --- | --- | --- | --- | --- | --- | --- | --- | --- | --- | --- | --- | --- | --- | --- | --- | --- | --- | --- | --- | --- | --- | --- | --- | --- | --- | --- | --- | --- | --- | --- | --- | --- | --- | --- | --- | --- | --- | --- | --- | --- | --- | --- | --- | --- | --- | --- | --- | --- | --- | --- | --- | --- | --- | --- | --- | --- | --- | --- | --- | --- | --- | --- | --- | --- | --- | --- | --- | --- | --- | --- | --- | --- | --- | --- | --- | --- | --- | --- | --- | --- | --- | --- | --- | --- | --- | --- | --- | --- | --- | --- | --- | --- | --- | --- | --- | --- | --- | --- | --- | --- | --- | --- | --- | --- | --- | --- | --- | --- | --- | --- | --- | --- | --- | --- | --- | --- | --- | --- | --- | --- | --- | --- | --- | --- | --- | --- | --- | --- | --- | --- | --- | --- | --- | --- | --- | --- | --- | --- | --- | --- | --- | --- | --- | --- | --- | --- | --- | --- | --- | --- | --- | --- | --- | --- | --- | --- | --- | --- | --- | --- | --- | --- | --- | --- | --- | --- | --- | --- | --- | --- | --- | --- | --- | --- | --- | --- | --- | --- | --- | --- | --- | --- | --- | --- | --- | --- | --- | --- | --- | --- | --- | --- | --- | --- | --- | --- | --- | --- | --- | --- | --- | --- | --- | --- | --- | --- | --- | --- | --- | --- | --- | --- | --- | --- | --- | --- | --- | --- | --- | --- | --- | --- | --- | --- | --- | --- | --- | --- | --- | --- | --- | --- | --- | --- | --- | --- | --- | --- | --- | --- | --- | --- | --- | --- | --- | --- | --- | --- | --- | --- | --- | --- | --- | --- | --- | --- | --- | --- | --- | --- | --- | --- | --- | --- | --- | --- | --- | --- | --- | --- | --- | --- | --- | --- | --- | --- | --- | --- | --- | --- | --- | --- | --- | --- | --- | --- | --- | --- | --- | --- | --- | --- | --- | --- | --- | --- | --- | --- | --- | --- | --- | --- | --- | --- | --- | --- | --- | --- | --- | --- | --- | --- | --- | --- | --- | --- | --- | --- | --- | --- | --- | --- | --- | --- | --- | --- | --- | --- | --- | --- | --- | --- | --- | --- | --- | --- | --- | --- | --- | --- | --- | --- | --- | --- | --- | --- | --- | --- | --- | --- | --- | --- | --- | --- | --- | --- | --- | --- | --- | --- | --- | --- | --- | --- | --- | --- | --- | --- | --- | --- | --- | --- | --- | --- | --- | --- | --- | --- | --- | --- | --- | --- | --- | --- | --- | --- | --- | --- | --- | --- | --- | --- | --- | --- | --- | --- | --- | --- | --- | --- | --- | --- | --- | --- | --- | --- | --- | --- | --- | --- | --- | --- | --- | --- | --- | --- | --- | --- | --- | --- | --- | --- | --- | --- | --- | --- | --- | --- | --- | --- | --- | --- | --- | --- | --- | --- | --- | --- | --- | --- | --- | --- | --- | --- | --- | --- | --- | --- | --- | --- | --- | --- | --- | --- | --- | --- | --- | --- | --- | --- | --- | --- | --- | --- | --- | --- | --- | --- | --- | --- | --- | --- | --- | --- | --- | --- | --- | --- | --- | --- | --- | --- | --- | --- | --- | --- | --- | --- | --- | --- | --- | --- | --- | --- | --- | --- | --- | --- | --- | --- | --- | --- | --- | --- | --- | --- | --- | --- | --- | --- | --- | --- | --- | --- | --- | --- | --- | --- | --- | --- | --- | --- | --- | --- | --- | --- | --- | --- | --- | --- | --- | --- | --- | --- | --- | --- | --- | --- | --- | --- | --- | --- | --- | --- | --- | --- | --- | --- | --- | --- | --- | --- | --- | --- | --- | --- | --- | --- | --- | --- | --- | --- | --- | --- | --- | --- | --- | --- | --- | --- | --- | --- | --- | --- | --- | --- | --- | --- | --- | --- | --- | --- | --- | --- | --- | --- | --- | --- | --- | --- | --- | --- | --- | --- | --- | --- | --- | --- | --- | --- | --- | --- | --- | --- | --- | --- | --- | --- | --- | --- | --- | --- | --- | --- | --- | --- | --- | --- | --- | --- | --- | --- | --- | --- | --- | --- | --- | --- | --- | --- | --- | --- | --- | --- | --- | --- | --- | --- | --- | --- | --- | --- | --- | --- | --- | --- | --- | --- | --- | --- | --- | --- | --- | --- | --- | --- | --- | --- | --- | --- | --- | --- | --- | --- | --- | --- | --- | --- | --- | --- | --- | --- | --- | --- | --- | --- | --- | --- | --- | --- | --- | --- | --- | --- | --- | --- | --- | --- | --- | --- | --- | --- | --- | --- | --- | --- | --- | --- | --- | --- | --- | --- | --- | --- | --- | --- | --- | --- | --- | --- | --- | --- | --- | --- | --- | --- | --- | --- | --- | --- | --- | --- | --- | --- | --- | --- | --- | --- | --- | --- | --- | --- | --- | --- | --- | --- | --- | --- | --- | --- | --- | --- | --- | --- | --- | --- | --- | --- | --- | --- | --- | --- | --- | --- | --- | --- | --- | --- | --- | --- | --- | --- | --- | --- | --- | --- | --- | --- | --- | --- | --- | --- | --- | --- | --- | --- | --- | --- | --- | --- | --- | --- | --- | --- | --- | --- | --- | --- | --- | --- | --- | --- | --- | --- | --- | --- | --- | --- | --- | --- | --- | --- | --- | --- | --- | --- | --- | --- | --- | --- | --- | --- | --- | --- | --- | --- | --- | --- | --- | --- | --- | --- | --- | --- | --- | --- | --- | --- | --- | --- | --- | --- | --- | --- | --- | --- | --- | --- | --- | --- | --- | --- | --- | --- | --- | --- | --- | --- | --- | --- | --- | --- | --- | --- | --- | --- | --- | --- | --- | --- | --- | --- | --- | --- | --- | --- | --- | --- | --- | --- | --- | --- | --- | --- | --- | --- | --- | --- | --- | --- | --- | --- | --- | --- | --- | --- | --- | --- | --- | --- | --- | --- | --- | --- | --- | --- | --- | --- | --- | --- | --- | --- | --- | --- | --- | --- | --- | --- | --- | --- | --- | --- | --- | --- | --- | --- | --- | --- | --- | --- | --- | --- | --- | --- | --- | --- | --- | --- | --- | --- | --- | --- | --- | --- | --- | --- | --- | --- | --- | --- | --- | --- | --- | --- | --- | --- | --- | --- | --- | --- | --- | --- | --- | --- | --- | --- | --- | --- | --- | --- | --- | --- | --- | --- | --- | --- | --- | --- | --- | --- | --- | --- | --- | --- | --- | --- | --- | --- | --- | --- | --- | --- | --- | --- | --- | --- | --- | --- | --- | --- | --- | --- | --- | --- | --- | --- | --- | --- | --- | --- | --- | --- | --- | --- | --- | --- | --- | --- | --- | --- | --- | --- | --- | --- | --- | --- | --- | --- | --- | --- | --- | --- | --- | --- | --- | --- | --- | --- | --- | --- | --- | --- | --- | --- | --- | --- | --- | --- | --- | --- | --- | --- | --- | --- | --- | --- | --- | --- | --- | --- | --- | --- | --- | --- | --- | --- | --- | --- | --- | --- | --- | --- | --- | --- | --- | --- | --- | --- | --- | --- | --- | --- | --- | --- | --- | --- | --- | --- | --- | --- | --- | --- | --- | --- | --- | --- | --- | --- | --- | --- | --- | --- | --- | --- | --- | --- | --- | --- | --- | --- | --- | --- | --- | --- | --- | --- | --- | --- | --- | --- | --- | --- | --- | --- | --- | --- | --- | --- | --- | --- | --- | --- | --- | --- | --- | --- | --- | --- | --- | --- | --- | --- | --- | --- | --- | --- | --- | --- | --- | --- | --- | --- | --- | --- | --- | --- | --- | --- | --- | --- | --- | --- | --- | --- | --- | --- | --- | --- | --- | --- | --- | --- | --- | --- | --- | --- | --- | --- | --- | --- | --- | --- | --- | --- | --- | --- | --- | --- | --- | --- | --- | --- | --- | --- | --- | --- | --- | --- | --- | --- | --- | --- | --- | --- | --- | --- | --- | --- | --- | --- | --- | --- | --- | --- | --- | --- | --- | --- | --- | --- | --- | --- | --- | --- | --- | --- | --- | --- | --- | --- | --- | --- | --- | --- | --- | --- | --- | --- | --- | --- | --- | --- | --- | --- | --- | --- | --- | --- | --- | --- | --- | --- | --- | --- | --- | --- | --- | --- | --- | --- | --- | --- | --- | --- | --- | --- | --- | --- | --- | --- | --- | --- | --- | --- | --- | --- | --- | --- | --- | --- | --- | --- | --- | --- | --- | --- | --- | --- | --- | --- | --- | --- | --- | --- | --- | --- | --- | --- | --- | --- | --- | --- | --- | --- | --- | --- | --- | --- | --- | --- | --- | --- | --- | --- | --- | --- | --- | --- | --- | --- | --- | --- | --- | --- | --- | --- | --- | --- | --- | --- | --- | --- | --- | --- | --- | --- | --- | --- | --- | --- | --- | --- | --- | --- | --- | --- | --- | --- | --- | --- | --- | --- | --- | --- | --- | --- | --- | --- | --- | --- | --- | --- | --- | --- | --- | --- | --- | --- | --- | --- | --- | --- | --- | --- | --- | --- | --- | --- | --- | --- | --- | --- | --- | --- | --- | --- | --- | --- | --- | --- | --- | --- | --- | --- | --- | --- | --- | --- | --- | --- | --- | --- | --- | --- | --- | --- | --- | --- | --- | --- | --- | --- | --- | --- | --- | --- | --- | --- | --- | --- | --- | --- | --- | --- | --- | --- | --- | --- | --- | --- | --- | --- | --- | --- | --- | --- | --- | --- | --- | --- | --- | --- | --- | --- | --- | --- | --- | --- | --- | --- | --- | --- | --- | --- | --- | --- | --- | --- | --- | --- | --- | --- | --- | --- | --- | --- | --- | --- | --- | --- | --- | --- | --- | --- | --- | --- | --- | --- | --- | --- | --- | --- | --- | --- | --- | --- | --- | --- | --- | --- | --- | --- | --- | --- | --- | --- | --- | --- | --- | --- | --- | --- | --- | --- | --- | --- | --- | --- | --- | --- | --- | --- | --- | --- | --- | --- | --- | --- | --- | --- | --- | --- | --- | --- | --- | --- | --- | --- | --- | --- | --- | --- | --- | --- | --- | --- | --- | --- | --- | --- | --- | --- | --- | --- | --- | --- | --- | --- | --- | --- | --- | --- | --- | --- | --- | --- | --- | --- | --- | --- | --- | --- | --- | --- | --- | --- | --- | --- | --- | --- | --- | --- | --- | --- | --- | --- | --- | --- | --- | --- | --- | --- | --- | --- | --- | --- | --- | --- | --- | --- | --- | --- | --- | --- | --- | --- | --- | --- | --- | --- | --- | --- | --- | --- | --- | --- | --- | --- | --- | --- | --- | --- | --- | --- | --- | --- | --- | --- | --- | --- | --- | --- | --- | --- | --- | --- | --- | --- | --- | --- | --- | --- | --- | --- | --- | --- | --- | --- | --- | --- | --- | --- | --- | --- | --- | --- | --- | --- | --- | --- | --- | --- | --- | --- | --- | --- | --- | --- | --- | --- | --- | --- | --- | --- | --- | --- | --- | --- | --- | --- | --- | --- | --- | --- | --- | --- | --- | --- | --- | --- | --- | --- | --- | --- | --- | --- | --- | --- | --- | --- | --- | --- | --- | --- | --- | --- | --- | --- | --- | --- | --- | --- | --- | --- | --- | --- | --- | --- | --- | --- | --- | --- | --- | --- | --- | --- | --- | --- | --- | --- | --- | --- | --- | --- | --- | --- | --- | --- | --- | --- | --- | --- | --- | --- | --- | --- | --- | --- | --- | --- | --- | --- | --- | --- | --- | --- | --- | --- | --- | --- | --- | --- | --- | --- | --- | --- | --- | --- | --- | --- | --- | --- | --- | --- | --- | --- | --- | --- | --- | --- | --- | --- | --- | --- | --- | --- | --- | --- | --- | --- | --- | --- | --- | --- | --- | --- | --- | --- | --- | --- | --- | --- | --- | --- | --- | --- | --- | --- | --- | --- | --- | --- | --- | --- | --- | --- | --- | --- | --- | --- | --- | --- | --- | --- | --- | --- | --- | --- | --- | --- | --- | --- | --- | --- | --- | --- | --- | --- | --- | --- | --- | --- | --- | --- | --- | --- | --- | --- | --- | --- | --- | --- | --- | --- | --- | --- | --- | --- | --- | --- | --- | --- | --- | --- | --- | --- | --- | --- | --- | --- | --- | --- | --- | --- | --- | --- | --- | --- | --- | --- | --- | --- | --- | --- | --- | --- | --- | --- | --- | --- | --- | --- | --- | --- | --- | --- | --- | --- | --- | --- | --- | --- | --- | --- | --- | --- | --- | --- | --- | --- | --- | --- | --- | --- | --- | --- | --- | --- | --- | --- | --- | --- | --- | --- | --- | --- | --- | --- | --- | --- | --- | --- | --- | --- | --- | --- | --- | --- | --- | --- | --- | --- | --- | --- | --- | --- | --- | --- | --- | --- | --- | --- | --- | --- | --- | --- | --- | --- | --- | --- | --- | --- | --- | --- | --- | --- | --- | --- | --- | --- | --- | --- | --- | --- | --- | --- | --- | --- | --- | --- | --- | --- | --- | --- | --- | --- | --- | --- | --- | --- | --- | --- | --- | --- | --- | --- | --- | --- | --- | --- | --- | --- | --- | --- | --- | --- | --- | --- | --- | --- | --- | --- | --- | --- | --- | --- | --- | --- | --- | --- | --- | --- | --- | --- | --- | --- | --- | --- | --- | --- | --- | --- | --- | --- | --- | --- | --- | --- | --- | --- | --- | --- | --- | --- | --- | --- | --- | --- | --- | --- | --- | --- | --- | --- | --- | --- | --- | --- | --- | --- | --- | --- | --- | --- | --- | --- | --- | --- | --- | --- | --- | --- | --- | --- | --- | --- | --- | --- | --- | --- | --- | --- | --- | --- | --- | --- | --- | --- | --- | --- | --- | --- | --- | --- | --- | --- | --- | --- | --- | --- | --- | --- | --- | --- | --- | --- | --- | --- | --- | --- | --- | --- | --- | --- | --- | --- | --- | --- | --- | --- | --- | --- | --- | --- | --- | --- | --- | --- | --- | --- | --- | --- | --- | --- | --- | --- | --- | --- | --- | --- | --- | --- | --- | --- | --- | --- | --- | --- | --- | --- | --- | --- | --- | --- | --- | --- | --- | --- | --- | --- | --- | --- | --- | --- | --- | --- | --- | --- | --- | --- | --- | --- | --- | --- | --- | --- | --- | --- | --- | --- | --- | --- | --- | --- | --- | --- | --- | --- | --- | --- | --- | --- | --- | --- | --- | --- | --- | --- | --- | --- | --- | --- | --- | --- | --- | --- | --- | --- | --- | --- | --- | --- | --- | --- | --- | --- | --- | --- | --- | --- | --- | --- | --- | --- | --- | --- | --- | --- | --- | --- | --- | --- | --- | --- | --- | --- | --- | --- | --- | --- | --- | --- | --- | --- | --- | --- | --- | --- | --- | --- | --- | --- | --- | --- | --- | --- | --- | --- | --- | --- | --- | --- | --- | --- | --- | --- | --- | --- | --- | --- | --- | --- | --- | --- | --- | --- | --- | --- | --- | --- | --- | --- | --- | --- | --- | --- | --- | --- | --- | --- | --- | --- | --- | --- | --- | --- | --- | --- | --- | --- | --- | --- | --- | --- | --- | --- | --- | --- | --- | --- | --- | --- | --- | --- | --- | --- | --- | --- | --- | --- | --- | --- | --- | --- | --- | --- | --- | --- | --- | --- | --- | --- | --- | --- | --- | --- | --- | --- | --- | --- | --- | --- | --- | --- | --- | --- | --- | --- | --- | --- | --- | --- | --- | --- | --- | --- | --- | --- | --- | --- | --- | --- | --- | --- | --- | --- | --- | --- | --- | --- | --- | --- | --- | --- | --- | --- | --- | --- | --- | --- | --- | --- | --- | --- | --- | --- | --- | --- | --- | --- | --- | --- | --- | --- | --- | --- | --- | --- | --- | --- | --- | --- | --- | --- | --- | --- | --- | --- | --- | --- | --- | --- | --- | --- | --- | --- | --- | --- | --- | --- | --- | --- | --- | --- | --- | --- | --- | --- | --- | --- | --- | --- | --- | --- | --- | --- | --- | --- | --- | --- | --- | --- | --- | --- | --- | --- | --- | --- | --- | --- | --- | --- | --- | --- | --- | --- | --- | --- | --- | --- | --- | --- | --- | --- | --- | --- | --- | --- | --- | --- | --- | --- | --- | --- | --- | --- | --- | --- | --- | --- | --- | --- | --- | --- | --- | --- | --- | --- | --- | --- | --- | --- | --- | --- | --- | --- | --- | --- | --- | --- | --- | --- | --- | --- | --- | --- | --- | --- | --- | --- | --- | --- | --- | --- | --- | --- | --- | --- | --- | --- | --- | --- | --- | --- | --- | --- | --- | --- | --- | --- | --- | --- | --- | --- | --- | --- | --- | --- | --- | --- | --- | --- | --- | --- | --- | --- | --- | --- | --- | --- | --- | --- | --- | --- | --- | --- | --- | --- | --- | --- | --- | --- | --- | --- | --- | --- | --- | --- | --- | --- | --- | --- | --- | --- | --- | --- | --- | --- | --- | --- | --- | --- | --- | --- | --- | --- | --- | --- | --- | --- | --- | --- | --- | --- | --- | --- | --- | --- | --- | --- | --- | --- | --- | --- | --- | --- | --- | --- | --- | --- | --- | --- | --- | --- | --- | --- | --- | --- | --- | --- | --- | --- | --- | --- | --- | --- | --- | --- | --- | --- | --- | --- | --- | --- | --- | --- | --- | --- | --- | --- | --- | --- | --- | --- | --- | --- | --- | --- | --- | --- | --- | --- | --- | --- | --- | --- | --- | --- | --- | --- | --- | --- | --- | --- | --- | --- | --- | --- | --- | --- | --- | --- | --- | --- | --- | --- | --- | --- | --- | --- | --- | --- | --- | --- | --- | --- | --- | --- | --- | --- | --- | --- | --- | --- | --- | --- | --- | --- | --- | --- | --- | --- | --- | --- | --- | --- | --- | --- | --- | --- | --- | --- | --- | --- | --- | --- | --- | --- | --- | --- | --- | --- | --- | --- | --- | --- | --- | --- | --- | --- | --- | --- | --- | --- | --- | --- | --- | --- | --- | --- | --- | --- | --- | --- | --- | --- | --- | --- | --- | --- | --- | --- | --- | --- | --- | --- | --- | --- | --- | --- | --- | --- | --- | --- | --- | --- | --- | --- | --- | --- | --- | --- | --- | --- | --- | --- | --- | --- | --- | --- | --- | --- | --- | --- | --- | --- | --- | --- | --- | --- | --- | --- | --- | --- | --- | --- | --- | --- | --- | --- | --- | --- | --- | --- | --- | --- | --- | --- | --- | --- | --- | --- | --- | --- | --- | --- | --- | --- | --- | --- | --- | --- | --- | --- | --- | --- | --- | --- | --- | --- | --- | --- | --- | --- | --- | --- | --- | --- | --- | --- | --- | --- | --- | --- | --- | --- | --- | --- | --- | --- | --- | --- | --- | --- | --- | --- | --- | --- | --- | --- | --- | --- | --- | --- | --- | --- | --- | --- | --- | --- | --- | --- | --- | --- | --- | --- | --- | --- | --- | --- | --- | --- | --- | --- | --- | --- | --- | --- | --- | --- | --- | --- | --- | --- | --- | --- | --- | --- | --- | --- | --- | --- | --- | --- | --- | --- | --- | --- | --- | --- | --- | --- | --- | --- | --- | --- | --- | --- | --- | --- | --- | --- | --- | --- | --- | --- | --- | --- | --- | --- | --- | --- | --- | --- | --- | --- | --- | --- | --- | --- | --- | --- | --- | --- | --- | --- | --- | --- | --- | --- | --- | --- | --- | --- | --- | --- | --- | --- | --- | --- | --- | --- | --- | --- | --- | --- | --- | --- | --- | --- | --- | --- | --- | --- | --- | --- | --- | --- | --- | --- | --- | --- | --- | --- | --- | --- | --- | --- | --- | --- | --- | --- | --- | --- | --- | --- | --- | --- | --- | --- | --- | --- | --- | --- | --- | --- | --- | --- | --- | --- | --- | --- | --- | --- | --- | --- | --- | --- | --- | --- | --- | --- | --- | --- | --- | --- | --- | --- | --- | --- | --- | --- | --- | --- | --- | --- | --- | --- | --- | --- | --- | --- | --- | --- | --- | --- | --- | --- | --- | --- | --- | --- | --- | --- | --- | --- | --- | --- | --- | --- | --- | --- | --- | --- | --- | --- | --- | --- | --- | --- | --- | --- | --- | --- | --- | --- | --- | --- | --- | --- | --- | --- | --- | --- | --- | --- | --- | --- | --- | --- | --- | --- | --- | --- | --- | --- | --- | --- | --- | --- | --- | --- | --- | --- | --- | --- | --- | --- | --- | --- | --- | --- | --- | --- | --- | --- | --- | --- | --- | --- | --- | --- | --- | --- | --- | --- | --- | --- | --- | --- | --- | --- | --- | --- | --- | --- | --- | --- | --- | --- | --- | --- | --- | --- | --- | --- | --- | --- | --- | --- | --- | --- | --- | --- | --- | --- | --- | --- | --- | --- | --- | --- | --- | --- | --- | --- | --- | --- | --- | --- | --- | --- | --- | --- | --- | --- | --- | --- | --- | --- | --- | --- | --- | --- | --- | --- | --- | --- | --- | --- | --- | --- | --- | --- | --- | --- | --- | --- | --- | --- | --- | --- | --- | --- | --- | --- | --- | --- | --- | --- | --- | --- | --- | --- | --- | --- | --- | --- | --- | --- | --- | --- | --- | --- | --- | --- | --- | --- | --- | --- | --- | --- | --- | --- | --- | --- | --- | --- | --- | --- | --- | --- | --- | --- | --- | --- | --- | --- | --- | --- | --- | --- | --- | --- | --- | --- | --- | --- | --- | --- | --- | --- | --- | --- | --- | --- | --- | --- | --- | --- | --- | --- | --- | --- | --- | --- | --- | --- | --- | --- | --- | --- | --- | --- | --- | --- | --- | --- | --- | --- | --- | --- | --- | --- | --- | --- | --- | --- | --- | --- | --- | --- | --- | --- | --- | --- | --- | --- | --- | --- | --- | --- | --- | --- | --- | --- | --- | --- | --- | --- | --- | --- | --- | --- | --- | --- | --- | --- | --- | --- | --- | --- | --- | --- | --- | --- | --- | --- | --- | --- | --- | --- | --- | --- | --- | --- | --- | --- | --- | --- | --- | --- | --- | --- | --- | --- | --- | --- | --- | --- | --- | --- | --- | --- | --- | --- | --- | --- | --- | --- | --- | --- | --- | --- | --- | --- | --- | --- | --- | --- | --- | --- | --- | --- | --- | --- | --- | --- | --- | --- | --- | --- | --- | --- | --- | --- | --- | --- | --- | --- | --- | --- | --- | --- | --- | --- | --- | --- | --- | --- | --- | --- | --- | --- | --- | --- | --- | --- | --- | --- | --- | --- | --- | --- | --- | --- | --- | --- | --- | --- | --- | --- | --- | --- | --- | --- | --- | --- | --- | --- | --- | --- | --- | --- | --- | --- | --- | --- | --- | --- | --- | --- | --- | --- | --- | --- | --- | --- | --- | --- | --- | --- | --- | --- | --- | --- | --- | --- | --- | --- | --- | --- | --- | --- | --- | --- | --- | --- | --- | --- | --- | --- | --- | --- | --- | --- | --- | --- | --- | --- | --- | --- | --- | --- | --- | --- | --- | --- | --- | --- | --- | --- | --- | --- | --- | --- | --- | --- | --- | --- | --- | --- | --- | --- | --- | --- | --- | --- | --- | --- | --- | --- | --- | --- | --- | --- | --- | --- | --- | --- | --- | --- | --- | --- | --- | --- | --- | --- | --- | --- | --- | --- | --- | --- | --- | --- | --- | --- | --- | --- | --- | --- | --- | --- | --- | --- | --- | --- | --- | --- | --- | --- | --- | --- | --- | --- | --- | --- | --- | --- | --- | --- | --- | --- | --- | --- | --- | --- | --- | --- | --- | --- | --- | --- | --- | --- | --- | --- | --- | --- | --- | --- | --- | --- | --- | --- | --- | --- | --- | --- | --- | --- | --- | --- | --- | --- | --- | --- | --- | --- | --- | --- | --- | --- | --- | --- | --- | --- | --- | --- | --- | --- | --- | --- | --- | --- | --- | --- | --- | --- | --- | --- | --- | --- | --- | --- | --- | --- | --- | --- | --- | --- | --- | --- | --- | --- | --- | --- | --- | --- | --- | --- | --- | --- | --- | --- | --- | --- | --- | --- | --- | --- | --- | --- | --- | --- | --- | --- | --- | --- | --- | --- | --- | --- | --- | --- | --- | --- | --- | --- | --- | --- | --- | --- | --- | --- | --- | --- | --- | --- | --- | --- | --- | --- | --- | --- | --- | --- | --- | --- | --- | --- | --- | --- | --- | --- | --- | --- | --- | --- | --- | --- | --- | --- | --- | --- | --- | --- | --- | --- | --- | --- | --- | --- | --- | --- | --- | --- | --- | --- | --- | --- | --- | --- | --- | --- | --- | --- | --- | --- | --- | --- | --- | --- | --- | --- | --- | --- | --- | --- | --- | --- | --- | --- | --- | --- | --- | --- | --- | --- | --- | --- | --- | --- | --- | --- | --- | --- | --- | --- | --- | --- | --- | --- | --- | --- | --- | --- | --- | --- | --- | --- | --- | --- | --- | --- | --- | --- | --- | --- | --- | --- | --- | --- | --- | --- | --- | --- | --- | --- | --- | --- | --- | --- | --- | --- | --- | --- | --- | --- | --- | --- | --- | --- | --- | --- | --- | --- | --- | --- | --- | --- | --- | --- | --- | --- | --- | --- | --- | --- | --- | --- | --- | --- | --- | --- | --- | --- | --- | --- | --- | --- | --- | --- | --- | --- | --- | --- | --- | --- | --- | --- | --- | --- | --- | --- | --- | --- | --- | --- | --- | --- | --- | --- | --- | --- | --- | --- | --- | --- | --- | --- | --- | --- | --- | --- | --- | --- | --- | --- | --- | --- | --- | --- | --- | --- | --- | --- | --- | --- | --- | --- | --- | --- | --- | --- | --- | --- | --- | --- | --- | --- | --- | --- | --- | --- | --- | --- | --- | --- | --- | --- | --- | --- | --- | --- | --- | --- | --- | --- | --- | --- | --- | --- | --- | --- | --- | --- | --- | --- | --- | --- | --- | --- | --- | --- | --- | --- | --- | --- | --- | --- | --- | --- | --- | --- | --- | --- | --- | --- | --- | --- | --- | --- | --- | --- | --- | --- | --- | --- | --- | --- | --- | --- | --- | --- | --- | --- | --- | --- | --- | --- | --- | --- | --- | --- | --- | --- | --- | --- | --- | --- | --- | --- | --- | --- | --- | --- | --- | --- | --- | --- | --- | --- | --- | --- | --- | --- | --- | --- | --- | --- | --- | --- | --- | --- | --- | --- | --- | --- | --- | --- | --- | --- | --- | --- | --- | --- | --- | --- | --- | --- | --- | --- | --- | --- | --- | --- | --- | --- | --- | --- | --- | --- | --- | --- | --- | --- | --- | --- | --- | --- | --- | --- | --- | --- | --- | --- | --- | --- | --- | --- | --- | --- | --- | --- | --- | --- | --- | --- | --- | --- | --- | --- | --- | --- | --- | --- | --- | --- | --- | --- | --- | --- | --- | --- | --- | --- | --- | --- | --- | --- | --- | --- | --- | --- | --- | --- | --- | --- | --- | --- | --- | --- | --- | --- | --- | --- | --- | --- | --- | --- | --- | --- | --- | --- | --- | --- | --- | --- | --- | --- | --- | --- | --- | --- | --- | --- | --- | --- | --- | --- | --- | --- | --- | --- | --- | --- | --- | --- | --- | --- | --- | --- | --- | --- | --- | --- | --- | --- | --- | --- | --- | --- | --- | --- | --- | --- | --- | --- | --- | --- | --- | --- | --- | --- | --- | --- | --- | --- | --- | --- | --- | --- | --- | --- | --- | --- | --- | --- | --- | --- | --- | --- | --- | --- | --- | --- | --- | --- | --- | --- | --- | --- | --- | --- | --- | --- | --- | --- | --- | --- | --- | --- | --- | --- | --- | --- | --- | --- | --- | --- | --- | --- | --- | --- | --- | --- | --- | --- | --- | --- | --- | --- | --- | --- | --- | --- | --- | --- | --- | --- | --- | --- | --- | --- | --- | --- | --- | --- | --- | --- | --- | --- | --- | --- | --- | --- | --- | --- | --- | --- | --- | --- | --- | --- | --- | --- | --- | --- | --- | --- | --- | --- | --- | --- | --- | --- | --- | --- | --- | --- | --- | --- | --- | --- | --- | --- | --- | --- | --- | --- | --- | --- | --- | --- | --- | --- | --- | --- | --- | --- | --- | --- | --- | --- | --- | --- | --- | --- | --- | --- | --- | --- | --- | --- | --- | --- | --- | --- | --- | --- | --- | --- | --- | --- | --- | --- | --- | --- | --- | --- | --- | --- | --- | --- | --- | --- | --- | --- | --- | --- | --- | --- | --- | --- | --- | --- | --- | --- | --- | --- | --- | --- | --- | --- | --- | --- | --- | --- | --- | --- | --- | --- | --- | --- | --- | --- | --- | --- | --- | --- | --- | --- | --- | --- | --- | --- | --- | --- | --- | --- | --- | --- | --- | --- | --- | --- | --- | --- | --- | --- | --- | --- | --- | --- | --- | --- | --- | --- | --- | --- | --- | --- | --- | --- | --- | --- | --- | --- | --- | --- | --- | --- | --- | --- | --- | --- | --- | --- | --- | --- | --- | --- | --- | --- | --- | --- | --- | --- | --- | --- | --- | --- | --- | --- | --- | --- | --- | --- | --- | --- | --- | --- | --- | --- | --- | --- | --- | --- | --- | --- | --- | --- | --- | --- | --- | --- | --- | --- | --- | --- | --- | --- | --- | --- | --- | --- | --- | --- | --- | --- | --- | --- | --- | --- | --- | --- | --- | --- | --- | --- | --- | --- | --- | --- | --- | --- | --- | --- | --- | --- | --- | --- | --- | --- | --- | --- | --- | --- | --- | --- | --- | --- | --- | --- | --- | --- | --- | --- | --- | --- | --- | --- | --- | --- | --- | --- | --- | --- | --- | --- | --- | --- | --- | --- | --- | --- | --- | --- | --- | --- | --- | --- | --- | --- | --- | --- | --- | --- | --- | --- | --- | --- | --- | --- | --- | --- | --- | --- | --- | --- | --- | --- | --- | --- | --- | --- | --- | --- | --- | --- | --- | --- | --- | --- | --- | --- | --- | --- | --- | --- | --- | --- | --- | --- | --- | --- | --- | --- | --- | --- | --- | --- | --- | --- | --- | --- | --- | --- | --- | --- | --- | --- | --- | --- | --- | --- | --- | --- | --- | --- | --- | --- | --- | --- | --- | --- | --- | --- | --- | --- | --- | --- | --- | --- | --- | --- | --- | --- | --- | --- | --- | --- | --- | --- | --- | --- | --- | --- | --- | --- | --- | --- | --- | --- | --- | --- | --- | --- | --- | --- | --- | --- | --- | --- | --- | --- | --- | --- | --- | --- | --- | --- | --- | --- | --- | --- | --- | --- | --- | --- | --- | --- | --- | --- | --- | --- | --- | --- | --- | --- | --- | --- | --- | --- | --- | --- | --- | --- | --- | --- | --- | --- | --- | --- | --- | --- | --- | --- | --- | --- | --- | --- | --- | --- | --- | --- | --- | --- | --- | --- | --- | --- | --- | --- | --- | --- | --- | --- | --- | --- | --- | --- | --- | --- | --- | --- | --- | --- | --- | --- | --- | --- | --- | --- | --- | --- | --- | --- | --- | --- | --- | --- | --- | --- | --- | --- | --- | --- | --- | --- | --- | --- | --- | --- | --- | --- | --- | --- | --- | --- | --- | --- | --- | --- | --- | --- | --- | --- | --- | --- | --- | --- | --- | --- | --- | --- | --- | --- | --- | --- | --- | --- | --- | --- | --- | --- | --- | --- | --- | --- | --- | --- | --- | --- | --- | --- | --- | --- | --- | --- | --- | --- | --- | --- | --- | --- | --- | --- | --- | --- | --- | --- | --- | --- | --- | --- | --- | --- | --- | --- | --- | --- | --- | --- | --- | --- | --- | --- | --- | --- | --- | --- | --- | --- | --- | --- | --- | --- | --- | --- | --- | --- | --- | --- | --- | --- | --- | --- | --- | --- | --- | --- | --- | --- | --- | --- | --- | --- | --- | --- | --- | --- | --- | --- | --- | --- | --- | --- | --- | --- | --- | --- | --- | --- | --- | --- | --- | --- | --- | --- | --- | --- | --- | --- | --- | --- | --- | --- | --- | --- | --- | --- | --- | --- | --- | --- | --- | --- | --- | --- | --- | --- | --- | --- | --- | --- | --- | --- | --- | --- | --- | --- | --- | --- | --- | --- | --- | --- | --- | --- | --- | --- | --- | --- | --- | --- | --- | --- | --- | --- | --- | --- | --- | --- | --- | --- | --- | --- | --- | --- | --- | --- | --- | --- | --- | --- | --- | --- | --- | --- | --- | --- | --- | --- | --- | --- | --- | --- | --- | --- | --- | --- | --- | --- | --- | --- | --- | --- | --- | --- | --- | --- | --- | --- | --- | --- | --- | --- | --- | --- | --- | --- | --- | --- | --- | --- | --- | --- | --- | --- | --- | --- | --- | --- | --- | --- | --- | --- | --- | --- | --- | --- | --- | --- | --- | --- | --- | --- | --- | --- | --- | --- | --- | --- | --- | --- | --- | --- | --- | --- | --- | --- | --- | --- | --- | --- | --- | --- | --- | --- | --- | --- | --- | --- | --- | --- | --- | --- | --- | --- | --- | --- | --- | --- | --- | --- | --- | --- | --- | --- | --- | --- | --- | --- | --- | --- | --- | --- | --- | --- | --- | --- | --- | --- | --- | --- | --- | --- | --- | --- | --- | --- | --- | --- | --- | --- | --- | --- | --- | --- | --- | --- | --- | --- | --- | --- | --- | --- | --- | --- | --- | --- | --- | --- | --- | --- | --- | --- | --- | --- | --- | --- | --- | --- | --- | --- | --- | --- | --- | --- | --- | --- | --- | --- | --- | --- | --- | --- | --- | --- | --- | --- | --- | --- | --- | --- | --- | --- | --- | --- | --- | --- | --- | --- | --- | --- | --- | --- | --- | --- | --- | --- | --- | --- | --- | --- | --- | --- | --- | --- | --- | --- | --- | --- | --- | --- | --- | --- | --- | --- | --- | --- | --- | --- | --- | --- | --- | --- | --- | --- | --- | --- | --- | --- | --- | --- | --- | --- | --- | --- | --- | --- | --- | --- | --- | --- | --- | --- | --- | --- | --- | --- | --- | --- | --- | --- | --- | --- | --- | --- | --- | --- | --- | --- | --- | --- | --- | --- | --- | --- | --- | --- | --- | --- | --- | --- | --- | --- | --- | --- | --- | --- | --- | --- | --- | --- | --- | --- | --- | --- | --- | --- | --- | --- | --- | --- | --- | --- | --- | --- | --- | --- | --- | --- | --- | --- | --- | --- | --- | --- | --- | --- | --- | --- | --- | --- | --- | --- | --- | --- | --- | --- | --- | --- | --- | --- | --- | --- | --- | --- | --- | --- | --- | --- | --- | --- | --- | --- | --- | --- | --- | --- | --- | --- | --- | --- | --- | --- | --- | --- | --- | --- | --- | --- | --- | --- | --- | --- | --- | --- | --- | --- | --- | --- | --- | --- | --- | --- | --- | --- | --- | --- | --- | --- | --- | --- | --- | --- | --- | --- | --- | --- | --- | --- | --- | --- | --- | --- | --- | --- | --- | --- | --- | --- | --- | --- | --- | --- | --- | --- | --- | --- | --- | --- | --- | --- | --- | --- | --- | --- | --- | --- | --- | --- | --- | --- | --- | --- | --- | --- | --- | --- | --- | --- | --- | --- | --- | --- | --- | --- | --- | --- | --- | --- | --- | --- | --- | --- | --- | --- | --- | --- | --- | --- | --- | --- | --- | --- | --- | --- | --- | --- | --- | --- | --- | --- | --- | --- | --- | --- | --- | --- | --- | --- | --- | --- | --- | --- | --- | --- | --- | --- | --- | --- | --- | --- | --- | --- | --- | --- | --- | --- | --- | --- | --- | --- | --- | --- | --- | --- | --- | --- | --- | --- | --- | --- | --- | --- | --- | --- | --- | --- | --- | --- | --- | --- | --- | --- | --- | --- | --- | --- | --- | --- | --- | --- | --- | --- | --- | --- | --- | --- | --- | --- | --- | --- | --- | --- | --- | --- | --- | --- | --- | --- | --- | --- | --- | --- | --- | --- | --- | --- | --- | --- | --- | --- | --- | --- | --- | --- | --- | --- | --- | --- | --- | --- | --- | --- | --- | --- | --- | --- | --- | --- | --- | --- | --- | --- | --- | --- | --- | --- | --- | --- | --- | --- | --- | --- | --- | --- | --- | --- | --- | --- | --- | --- | --- | --- | --- | --- | --- | --- | --- | --- | --- | --- | --- | --- | --- | --- | --- | --- | --- | --- | --- | --- | --- | --- | --- | --- | --- | --- | --- | --- | --- | --- | --- | --- | --- | --- | --- | --- | --- | --- | --- | --- | --- | --- | --- | --- | --- | --- | --- | --- | --- | --- | --- | --- | --- | --- | --- | --- | --- | --- | --- | --- | --- | --- | --- | --- | --- | --- | --- | --- | --- | --- | --- | --- | --- | --- | --- | --- | --- | --- | --- | --- | --- | --- | --- | --- | --- | --- | --- | --- | --- | --- | --- | --- | --- | --- | --- | --- | --- | --- | --- | --- | --- | --- | --- | --- | --- | --- | --- | --- | --- | --- | --- | --- | --- | --- | --- | --- | --- | --- | --- | --- | --- | --- | --- | --- | --- | --- | --- | --- | --- | --- | --- | --- | --- | --- | --- | --- | --- | --- | --- | --- | --- | --- | --- | --- | --- | --- | --- | --- | --- | --- | --- | --- | --- | --- | --- | --- | --- | --- | --- | --- | --- | --- | --- | --- | --- | --- | --- | --- | --- | --- | --- | --- | --- | --- | --- | --- | --- | --- | --- | --- | --- | --- | --- | --- | --- | --- | --- | --- | --- | --- | --- | --- | --- | --- | --- | --- | --- | --- | --- | --- | --- | --- | --- | --- | --- | --- | --- | --- | --- | --- | --- | --- | --- | --- | --- | --- | --- | --- | --- | --- | --- | --- | --- | --- | --- | --- | --- | --- | --- | --- | --- | --- | --- | --- | --- | --- | --- | --- | --- | --- | --- | --- | --- | --- | --- | --- | --- | --- | --- | --- | --- | --- | --- | --- | --- | --- | --- | --- | --- | --- | --- | --- | --- | --- | --- | --- | --- | --- | --- | --- | --- | --- | --- | --- | --- | --- | --- | --- | --- | --- | --- | --- | --- | --- | --- | --- | --- | --- | --- | --- | --- | --- | --- | --- | --- | --- | --- | --- | --- | --- | --- | --- | --- | --- | --- | --- | --- | --- | --- | --- | --- | --- | --- | --- | --- | --- | --- | --- | --- | --- | --- | --- | --- | --- | --- | --- | --- | --- | --- | --- | --- | --- | --- | --- | --- | --- | --- | --- | --- | --- | --- | --- | --- | --- | --- | --- | --- | --- | --- | --- | --- | --- | --- | --- | --- | --- | --- | --- | --- | --- | --- | --- | --- | --- | --- | --- | --- | --- | --- | --- | --- | --- | --- | --- | --- | --- | --- | --- | --- | --- | --- | --- | --- | --- | --- | --- | --- | --- | --- | --- | --- | --- | --- | --- | --- | --- | --- | --- | --- | --- | --- | --- | --- | --- | --- | --- | --- | --- | --- | --- | --- | --- | --- | --- | --- | --- | --- | --- | --- | --- | --- | --- | --- | --- | --- | --- | --- | --- | --- | --- | --- | --- | --- | --- | --- | --- | --- | --- | --- | --- | --- | --- | --- | --- | --- | --- | --- | --- | --- | --- | --- | --- | --- | --- | --- | --- | --- | --- | --- | --- | --- | --- | --- | --- | --- | --- | --- | --- | --- | --- | --- | --- | --- | --- | --- | --- | --- | --- | --- | --- | --- | --- | --- | --- | --- | --- | --- | --- | --- | --- | --- | --- | --- | --- | --- | --- | --- | --- | --- | --- | --- | --- | --- | --- | --- | --- | --- | --- | --- | --- | --- | --- | --- | --- | --- | --- | --- | --- | --- | --- | --- | --- | --- | --- | --- | --- | --- | --- | --- | --- | --- | --- | --- | --- | --- | --- | --- | --- | --- | --- | --- | --- | --- | --- | --- | --- | --- | --- | --- | --- | --- | --- | --- | --- | --- | --- | --- | --- | --- | --- | --- | --- | --- | --- | --- | --- | --- | --- | --- | --- | --- | --- | --- | --- | --- | --- | --- | --- | --- | --- | --- | --- | --- | --- | --- | --- | --- | --- | --- | --- | --- | --- | --- | --- | --- | --- | --- | --- | --- | --- | --- | --- | --- | --- | --- | --- | --- | --- | --- | --- | --- | --- | --- | --- | --- | --- | --- | --- | --- | --- | --- | --- | --- | --- | --- | --- | --- | --- | --- | --- | --- | --- | --- | --- | --- | --- | --- | --- | --- | --- | --- | --- | --- | --- | --- | --- | --- | --- | --- | --- | --- | --- | --- | --- | --- | --- | --- | --- | --- | --- | --- | --- | --- | --- | --- | --- | --- | --- | --- | --- | --- | --- | --- | --- | --- | --- | --- | --- | --- | --- | --- | --- | --- | --- | --- | --- | --- | --- | --- | --- | --- | --- | --- | --- | --- | --- | --- | --- | --- | --- | --- | --- | --- | --- | --- | --- | --- | --- | --- | --- | --- | --- | --- | --- | --- | --- | --- | --- | --- | --- | --- | --- | --- | --- | --- | --- | --- | --- | --- | --- | --- | --- | --- | --- | --- | --- | --- | --- | --- | --- | --- | --- | --- | --- | --- | --- | --- | --- | --- | --- | --- | --- | --- | --- | --- | --- | --- | --- | --- | --- | --- | --- | --- | --- | --- | --- | --- | --- | --- | --- | --- | --- | --- | --- | --- | --- | --- | --- | --- | --- | --- | --- | --- | --- | --- | --- | --- | --- | --- | --- | --- | --- | --- | --- | --- | --- | --- | --- | --- | --- | --- | --- | --- | --- | --- | --- | --- | --- | --- | --- | --- | --- | --- | --- | --- | --- | --- | --- | --- | --- | --- | --- | --- | --- | --- | --- | --- | --- | --- | --- | --- | --- | --- | --- | --- | --- | --- | --- | --- | --- | --- | --- | --- | --- | --- | --- | --- | --- | --- | --- | --- | --- | --- | --- | --- | --- | --- | --- | --- | --- | --- | --- | --- | --- | --- | --- | --- | --- | --- | --- | --- | --- | --- | --- | --- | --- | --- | --- | --- | --- | --- | --- | --- | --- | --- | --- | --- | --- | --- | --- | --- | --- | --- | --- | --- | --- | --- | --- | --- | --- | --- | --- | --- | --- | --- | --- | --- | --- | --- | --- | --- | --- | --- | --- | --- | --- | --- | --- | --- | --- | --- | --- | --- | --- | --- | --- | --- | --- | --- | --- | --- | --- | --- | --- | --- | --- | --- | --- | --- | --- | --- | --- | --- | --- | --- | --- | --- | --- | --- | --- | --- | --- | --- | --- | --- | --- | --- | --- | --- | --- | --- | --- | --- | --- | --- | --- | --- | --- | --- | --- | --- | --- | --- | --- | --- | --- | --- | --- | --- | --- | --- | --- | --- | --- | --- | --- | --- | --- | --- | --- | --- | --- | --- | --- | --- | --- | --- | --- | --- | --- | --- | --- | --- | --- | --- | --- | --- | --- | --- | --- | --- | --- | --- | --- | --- | --- | --- | --- | --- | --- | --- | --- | --- | --- | --- | --- | --- | --- | --- | --- | --- | --- | --- | --- | --- | --- | --- | --- | --- | --- | --- | --- | --- | --- | --- | --- | --- | --- | --- | --- | --- | --- | --- | --- | --- | --- | --- | --- | --- | --- | --- | --- | --- | --- | --- | --- | --- | --- | --- | --- | --- | --- | --- | --- | --- | --- | --- | --- | --- | --- | --- | --- | --- | --- | --- | --- | --- | --- | --- | --- | --- | --- | --- | --- | --- | --- | --- | --- | --- | --- | --- | --- | --- | --- | --- | --- | --- | --- | --- | --- | --- | --- | --- | --- | --- | --- | --- | --- | --- | --- | --- | --- | --- | --- | --- | --- | --- | --- | --- | --- | --- | --- | --- | --- | --- | --- | --- | --- | --- | --- | --- | --- | --- | --- | --- | --- | --- | --- | --- | --- | --- | --- | --- | --- | --- | --- | --- | --- | --- | --- | --- | --- | --- | --- | --- | --- | --- | --- | --- | --- | --- | --- | --- | --- | --- | --- | --- | --- | --- | --- | --- | --- | --- | --- | --- | --- | --- | --- | --- | --- | --- | --- | --- | --- | --- | --- | --- | --- | --- | --- | --- | --- | --- | --- | --- | --- | --- | --- | --- | --- | --- | --- | --- | --- | --- | --- | --- | --- | --- | --- | --- | --- | --- | --- | --- | --- | --- | --- | --- | --- | --- | --- | --- | --- | --- | --- | --- | --- | --- | --- | --- | --- | --- | --- | --- | --- | --- | --- | --- | --- | --- | --- | --- | --- | --- | --- | --- | --- | --- | --- | --- | --- | --- | --- | --- | --- | --- | --- | --- | --- | --- | --- | --- | --- | --- | --- | --- | --- | --- | --- | --- | --- | --- | --- | --- | --- | --- | --- | --- | --- | --- | --- | --- | --- | --- | --- | --- | --- | --- | --- | --- | --- | --- | --- | --- | --- | --- | --- | --- | --- | --- | --- | --- | --- | --- | --- | --- | --- | --- | --- | --- | --- | --- | --- | --- | --- | --- | --- | --- | --- | --- | --- | --- | --- | --- | --- | --- | --- | --- | --- | --- | --- | --- | --- | --- | --- | --- | --- | --- | --- | --- | --- | --- | --- | --- | --- | --- | --- | --- | --- | --- | --- | --- | --- | --- | --- | --- | --- | --- | --- | --- | --- | --- | --- | --- | --- | --- | --- | --- | --- | --- | --- | --- | --- | --- | --- | --- | --- | --- | --- | --- | --- | --- | --- | --- | --- | --- | --- | --- | --- | --- | --- | --- | --- | --- | --- | --- | --- | --- | --- | --- | --- | --- | --- | --- | --- | --- | --- | --- | --- | --- | --- | --- | --- | --- | --- | --- | --- | --- | --- | --- | --- | --- | --- | --- | --- | --- | --- | --- | --- | --- | --- | --- | --- | --- | --- | --- | --- | --- | --- | --- | --- | --- | --- | --- | --- | --- | --- | --- | --- | --- | --- | --- | --- | --- | --- | --- | --- | --- | --- | --- | --- | --- | --- | --- | --- | --- | --- | --- | --- | --- | --- | --- | --- | --- | --- | --- | --- | --- | --- | --- | --- | --- | --- | --- | --- | --- | --- | --- | --- | --- | --- | --- | --- | --- | --- | --- | --- | --- | --- | --- | --- | --- | --- | --- | --- | --- | --- | --- | --- | --- | --- | --- | --- | --- | --- | --- | --- | --- | --- | --- | --- | --- | --- | --- | --- | --- | --- | --- | --- | --- | --- | --- | --- | --- | --- | --- | --- | --- | --- | --- | --- | --- | --- | --- | --- | --- | --- | --- | --- | --- | --- | --- | --- | --- | --- | --- | --- | --- | --- | --- | --- | --- | --- | --- | --- | --- | --- | --- | --- | --- | --- | --- | --- | --- | --- | --- | --- | --- | --- | --- | --- | --- | --- | --- | --- | --- | --- | --- | --- | --- | --- | --- | --- | --- | --- | --- | --- | --- | --- | --- | --- | --- | --- | --- | --- | --- | --- | --- | --- | --- | --- | --- | --- | --- | --- | --- | --- | --- | --- | --- | --- | --- | --- | --- | --- | --- | --- | --- | --- | --- | --- | --- | --- | --- | --- | --- | --- | --- | --- | --- | --- | --- | --- | --- | --- | --- | --- | --- | --- | --- | --- | --- | --- | --- | --- | --- | --- | --- | --- | --- | --- | --- | --- | --- | --- | --- | --- | --- | --- | --- | --- | --- | --- | --- | --- | --- | --- | --- | --- | --- | --- | --- | --- | --- | --- | --- | --- | --- | --- | --- | --- | --- | --- | --- | --- | --- | --- | --- | --- | --- | --- | --- | --- | --- | --- | --- | --- | --- | --- | --- | --- | --- | --- | --- | --- | --- | --- | --- | --- | --- | --- | --- | --- | --- | --- | --- | --- | --- | --- | --- | --- | --- | --- | --- | --- | --- | --- | --- | --- | --- | --- | --- | --- | --- | --- | --- | --- | --- | --- | --- | --- | --- | --- | --- | --- | --- | --- | --- | --- | --- | --- | --- | --- | --- | --- | --- | --- | --- | --- | --- | --- | --- | --- | --- | --- | --- | --- | --- | --- | --- | --- | --- | --- | --- | --- | --- | --- | --- | --- | --- | --- | --- | --- | --- | --- | --- | --- | --- | --- | --- | --- | --- | --- | --- | --- | --- | --- | --- | --- | --- | --- | --- | --- | --- | --- | --- | --- | --- | --- | --- | --- | --- | --- | --- | --- | --- | --- | --- | --- | --- | --- | --- | --- | --- | --- | --- | --- | --- | --- | --- | --- | --- | --- | --- | --- | --- | --- | --- | --- | --- | --- | --- | --- | --- | --- | --- | --- | --- | --- | --- | --- | --- | --- | --- | --- | --- | --- | --- | --- | --- | --- | --- | --- | --- | --- | --- | --- | --- | --- | --- | --- | --- | --- | --- | --- | --- | --- | --- | --- | --- | --- | --- | --- | --- | --- | --- | --- | --- | --- | --- | --- | --- | --- | --- | --- | --- | --- | --- | --- | --- | --- | --- | --- | --- | --- | --- | --- | --- | --- | --- | --- | --- | --- | --- | --- | --- | --- | --- | --- | --- | --- | --- | --- | --- | --- | --- | --- | --- | --- | --- | --- | --- | --- | --- | --- | --- | --- | --- | --- | --- | --- | --- | --- | --- | --- | --- | --- | --- | --- | --- | --- | --- | --- | --- | --- | --- | --- | --- | --- | --- | --- | --- | --- | --- | --- | --- | --- | --- | --- | --- | --- | --- | --- | --- | --- | --- | --- | --- | --- | --- | --- | --- | --- | --- | --- | --- | --- | --- | --- | --- | --- | --- | --- | --- | --- | --- | --- | --- | --- | --- | --- | --- | --- | --- | --- | --- | --- | --- | --- | --- | --- | --- | --- | --- | --- | --- | --- | --- | --- | --- | --- | --- | --- | --- | --- | --- | --- | --- | --- | --- | --- | --- | --- | --- | --- | --- | --- | --- | --- | --- | --- | --- | --- | --- | --- | --- | --- | --- | --- | --- | --- | --- | --- | --- | --- | --- | --- | --- | --- | --- | --- | --- | --- | --- | --- | --- | --- | --- | --- | --- | --- | --- | --- | --- | --- | --- | --- | --- | --- | --- | --- | --- | --- | --- | --- | --- | --- | --- | --- | --- | --- | --- | --- | --- | --- | --- | --- | --- | --- | --- | --- | --- | --- | --- | --- | --- | --- | --- | --- | --- | --- | --- | --- | --- | --- | --- | --- | --- | --- | --- | --- | --- | --- | --- | --- | --- | --- | --- | --- | --- | --- | --- | --- | --- | --- | --- | --- | --- | --- | --- | --- | --- | --- | --- | --- | --- | --- | --- | --- | --- | --- | --- | --- | --- | --- | --- | --- | --- | --- | --- | --- | --- | --- | --- | --- | --- | --- | --- | --- | --- | --- | --- | --- | --- | --- | --- | --- | --- | --- | --- | --- | --- | --- | --- | --- | --- | --- | --- | --- | --- | --- | --- | --- | --- | --- | --- | --- | --- | --- | --- | --- | --- | --- | --- | --- | --- | --- | --- | --- | --- | --- | --- | --- | --- | --- | --- | --- | --- | --- | --- | --- | --- | --- | --- | --- | --- | --- | --- | --- | --- | --- | --- | --- | --- | --- | --- | --- | --- | --- | --- | --- | --- | --- | --- | --- | --- | --- | --- | --- | --- | --- | --- | --- | --- | --- | --- | --- | --- | --- | --- | --- | --- | --- | --- | --- | --- | --- | --- | --- | --- | --- | --- | --- | --- | --- | --- | --- | --- | --- | --- | --- | --- | --- | --- | --- | --- | --- | --- | --- | --- | --- | --- | --- | --- | --- | --- | --- | --- | --- | --- | --- | --- | --- | --- | --- | --- | --- | --- | --- | --- | --- | --- | --- | --- | --- | --- | --- | --- | --- | --- | --- | --- | --- | --- | --- | --- | --- | --- | --- | --- | --- | --- | --- | --- | --- | --- | --- | --- | --- | --- | --- | --- | --- | --- | --- | --- | --- | --- | --- | --- | --- | --- | --- | --- | --- | --- | --- | --- | --- | --- | --- | --- | --- | --- | --- | --- | --- | --- | --- | --- | --- | --- | --- | --- | --- | --- | --- | --- | --- | --- | --- | --- | --- | --- | --- | --- | --- | --- | --- | --- | --- | --- | --- | --- | --- | --- | --- | --- | --- | --- | --- | --- | --- | --- | --- | --- | --- | --- | --- | --- | --- | --- | --- | --- | --- | --- | --- | --- | --- | --- | --- | --- | --- | --- | --- | --- | --- | --- | --- | --- | --- | --- | --- | --- | --- | --- | --- | --- | --- | --- | --- | --- | --- | --- | --- | --- | --- | --- | --- | --- | --- | --- | --- | --- | --- | --- | --- | --- | --- | --- | --- | --- | --- | --- | --- | --- | --- | --- | --- | --- | --- | --- | --- | --- | --- | --- | --- | --- | --- | --- | --- | --- | --- | --- | --- | --- | --- | --- | --- | --- | --- | --- | --- | --- | --- | --- | --- | --- | --- | --- | --- | --- | --- | --- | --- | --- | --- | --- | --- | --- | --- | --- | --- | --- | --- | --- | --- | --- | --- | --- | --- | --- | --- | --- | --- | --- | --- | --- | --- | --- | --- | --- | --- | --- | --- | --- | --- | --- | --- | --- | --- | --- | --- | --- | --- | --- | --- | --- | --- | --- | --- | --- | --- | --- | --- | --- | --- | --- | --- | --- | --- | --- | --- | --- | --- | --- | --- | --- | --- | --- | --- | --- | --- | --- | --- | --- | --- | --- | --- | --- | --- | --- | --- | --- | --- | --- | --- | --- | --- | --- | --- | --- | --- | --- | --- | --- | --- | --- | --- | --- | --- | --- | --- | --- | --- | --- | --- | --- | --- | --- | --- | --- | --- | --- | --- | --- | --- | --- | --- | --- | --- | --- | --- | --- | --- | --- | --- | --- | --- | --- | --- | --- | --- | --- | --- | --- | --- | --- | --- | --- | --- | --- | --- | --- | --- | --- | --- | --- | --- | --- | --- | --- | --- | --- | --- | --- | --- | --- | --- | --- | --- | --- | --- | --- | --- | --- | --- | --- | --- | --- | --- | --- | --- | --- | --- | --- | --- | --- | --- | --- | --- | --- | --- | --- | --- | --- | --- | --- | --- | --- | --- | --- | --- | --- | --- | --- | --- | --- | --- | --- | --- | --- | --- | --- | --- | --- | --- | --- | --- | --- | --- | --- | --- | --- | --- | --- | --- | --- | --- | --- | --- | --- | --- | --- | --- | --- | --- | --- | --- | --- | --- | --- | --- | --- | --- | --- | --- | --- | --- | --- | --- | --- | --- | --- | --- | --- | --- | --- | --- | --- | --- | --- | --- | --- | --- | --- | --- | --- | --- | --- | --- | --- | --- | --- | --- | --- | --- | --- | --- | --- | --- | --- | --- | --- | --- | --- | --- | --- | --- | --- | --- | --- | --- | --- | --- | --- | --- | --- | --- | --- | --- | --- | --- | --- | --- | --- | --- | --- | --- | --- | --- | --- | --- | --- | --- | --- | --- | --- | --- | --- | --- | --- | --- | --- | --- | --- | --- | --- | --- | --- | --- | --- | --- | --- | --- | --- | --- | --- | --- | --- | --- | --- | --- | --- | --- | --- | --- | --- | --- | --- | --- | --- | --- | --- | --- | --- | --- | --- | --- | --- | --- | --- | --- | --- | --- | --- | --- | --- | --- | --- | --- | --- | --- | --- | --- | --- | --- | --- | --- | --- | --- | --- | --- | --- | --- | --- | --- | --- | --- | --- | --- | --- | --- | --- | --- | --- | --- | --- | --- | --- | --- | --- | --- | --- | --- | --- | --- | --- | --- | --- | --- | --- | --- | --- | --- | --- | --- | --- | --- | --- | --- | --- | --- | --- | --- | --- | --- | --- | --- | --- | --- | --- | --- | --- | --- | --- | --- | --- | --- | --- | --- | --- | --- | --- | --- | --- | --- | --- | --- | --- | --- | --- | --- | --- | --- | --- | --- | --- | --- | --- | --- | --- | --- | --- | --- | --- | --- | --- | --- | --- | --- | --- | --- | --- | --- | --- | --- | --- | --- | --- | --- | --- | --- | --- | --- | --- | --- | --- | --- | --- | --- | --- | --- | --- | --- | --- | --- | --- | --- | --- | --- | --- | --- | --- | --- | --- | --- | --- | --- | --- | --- | --- | --- | --- | --- | --- | --- | --- | --- | --- | --- | --- | --- | --- | --- | --- | --- | --- | --- | --- | --- | --- | --- | --- | --- | --- | --- | --- | --- | --- | --- | --- | --- | --- | --- | --- | --- | --- | --- | --- | --- | --- | --- | --- | --- | --- | --- | --- | --- | --- | --- | --- | --- | --- | --- | --- | --- | --- | --- | --- | --- | --- | --- | --- | --- | --- | --- | --- | --- | --- | --- | --- | --- | --- | --- | --- | --- | --- | --- | --- | --- | --- | --- | --- | --- | --- | --- | --- | --- | --- | --- | --- | --- | --- | --- | --- | --- | --- | --- | --- | --- | --- | --- | --- | --- | --- | --- | --- | --- | --- | --- | --- | --- | --- | --- | --- | --- | --- | --- | --- | --- | --- | --- | --- | --- | --- | --- | --- | --- | --- | --- | --- | --- | --- | --- | --- | --- | --- | --- | --- | --- | --- | --- | --- | --- | --- | --- | --- | --- | --- | --- | --- | --- | --- | --- | --- | --- | --- | --- | --- | --- | --- | --- | --- | --- | --- | --- | --- | --- | --- | --- | --- | --- | --- | --- | --- | --- | --- | --- | --- | --- | --- | --- | --- | --- | --- | --- | --- | --- | --- | --- | --- | --- | --- | --- | --- | --- | --- | --- | --- | --- | --- | --- | --- | --- | --- | --- | --- | --- | --- | --- | --- | --- | --- | --- | --- | --- | --- | --- | --- | --- | --- | --- | --- | --- | --- | --- | --- | --- | --- | --- | --- | --- | --- | --- | --- | --- | --- | --- | --- | --- | --- | --- | --- | --- | --- | --- | --- | --- | --- | --- | --- | --- | --- | --- | --- | --- | --- | --- | --- | --- | --- | --- | --- | --- | --- | --- | --- | --- | --- | --- | --- | --- | --- | --- | --- | --- | --- | --- | --- | --- | --- | --- | --- | --- | --- | --- | --- | --- | --- | --- | --- | --- | --- | --- | --- | --- | --- | --- | --- | --- | --- | --- | --- | --- | --- | --- | --- | --- | --- | --- | --- | --- | --- | --- | --- | --- | --- | --- | --- | --- | --- | --- | --- | --- | --- | --- | --- | --- | --- | --- | --- | --- | --- | --- | --- | --- | --- | --- | --- | --- | --- | --- | --- | --- | --- | --- | --- | --- | --- | --- | --- | --- | --- | --- | --- | --- | --- | --- | --- | --- | --- | --- | --- | --- | --- | --- | --- | --- | --- | --- | --- | --- | --- | --- | --- | --- | --- | --- | --- | --- | --- | --- | --- | --- | --- | --- | --- | --- | --- | --- | --- | --- | --- | --- | --- | --- | --- | --- | --- | --- | --- | --- | --- | --- | --- | --- | --- | --- | --- | --- | --- | --- | --- | --- | --- | --- | --- | --- | --- | --- | --- | --- | --- | --- | --- | --- | --- | --- | --- | --- | --- | --- | --- | --- | --- | --- | --- | --- | --- | --- | --- | --- | --- | --- | --- | --- | --- | --- | --- | --- | --- | --- | --- | --- | --- | --- | --- | --- | --- | --- | --- | --- | --- | --- | --- | --- | --- | --- | --- | --- | --- | --- | --- | --- | --- | --- | --- | --- | --- | --- | --- | --- | --- | --- | --- | --- | --- | --- | --- | --- | --- | --- | --- | --- | --- | --- | --- | --- | --- | --- | --- | --- | --- | --- | --- | --- | --- | --- | --- | --- | --- | --- | --- | --- | --- | --- | --- | --- | --- | --- | --- | --- | --- | --- | --- | --- | --- | --- | --- | --- | --- | --- | --- | --- | --- | --- | --- | --- | --- | --- | --- | --- | --- | --- | --- | --- | --- | --- | --- | --- | --- | --- | --- | --- | --- | --- | --- | --- | --- | --- | --- | --- | --- | --- | --- | --- | --- | --- | --- | --- | --- | --- | --- | --- | --- | --- | --- | --- | --- | --- | --- | --- | --- | --- | --- | --- | --- | --- | --- | --- | --- | --- | --- | --- | --- | --- | --- | --- | --- | --- | --- | --- | --- | --- | --- | --- | --- | --- | --- | --- | --- | --- | --- | --- | --- | --- | --- | --- | --- | --- | --- | --- | --- | --- | --- | --- | --- | --- | --- | --- | --- | --- | --- | --- | --- | --- | --- | --- | --- | --- | --- | --- | --- | --- | --- | --- | --- | --- | --- | --- | --- | --- | --- | --- | --- | --- | --- | --- | --- | --- | --- | --- | --- | --- | --- | --- | --- | --- | --- | --- | --- | --- | --- | --- | --- | --- | --- | --- | --- | --- | --- | --- | --- | --- | --- | --- | --- | --- | --- | --- | --- | --- | --- | --- | --- | --- | --- | --- | --- | --- | --- | --- | --- | --- | --- | --- | --- | --- | --- | --- | --- | --- | --- | --- | --- | --- | --- | --- | --- | --- | --- | --- | --- | --- | --- | --- | --- | --- | --- | --- | --- | --- | --- | --- | --- | --- | --- | --- | --- | --- | --- | --- | --- | --- | --- | --- | --- | --- | --- | --- | --- | --- | --- | --- | --- | --- | --- | --- | --- | --- | --- | --- | --- | --- | --- | --- | --- | --- | --- | --- | --- | --- | --- | --- | --- | --- | --- | --- | --- | --- | --- | --- | --- | --- | --- | --- | --- | --- | --- | --- | --- | --- | --- | --- | --- | --- | --- | --- | --- | --- | --- | --- | --- | --- | --- | --- | --- | --- | --- | --- | --- | --- | --- | --- | --- | --- | --- | --- | --- | --- | --- | --- | --- | --- | --- | --- | --- | --- | --- | --- | --- | --- | --- | --- | --- | --- | --- | --- | --- | --- | --- | --- | --- | --- | --- | --- | --- | --- | --- | --- | --- | --- | --- | --- | --- | --- | --- | --- | --- | --- | --- | --- | --- | --- | --- | --- | --- | --- | --- | --- | --- | --- | --- | --- | --- | --- | --- | --- | --- | --- | --- | --- | --- | --- | --- | --- | --- | --- | --- | --- | --- | --- | --- | --- | --- | --- | --- | --- | --- | --- | --- | --- | --- | --- | --- | --- | --- | --- | --- | --- | --- | --- | --- | --- | --- | --- | --- | --- | --- | --- | --- | --- | --- | --- | --- | --- | --- | --- | --- | --- | --- | --- | --- | --- | --- | --- | --- | --- | --- | --- | --- | --- | --- | --- | --- | --- | --- | --- | --- | --- | --- | --- | --- | --- | --- | --- | --- | --- | --- | --- | --- | --- | --- | --- | --- | --- | --- | --- | --- | --- | --- | --- | --- | --- | --- | --- | --- | --- | --- | --- | --- | --- | --- | --- | --- | --- | --- | --- | --- | --- | --- | --- | --- | --- | --- | --- | --- | --- | --- | --- | --- | --- | --- | --- | --- | --- | --- | --- | --- | --- | --- | --- | --- | --- | --- | --- | --- | --- | --- | --- | --- | --- | --- | --- | --- | --- | --- | --- | --- | --- | --- | --- | --- | --- | --- | --- | --- | --- | --- | --- | --- | --- | --- | --- | --- | --- | --- | --- | --- | --- | --- | --- | --- | --- | --- | --- | --- | --- | --- | --- | --- | --- | --- | --- | --- | --- | --- | --- | --- | --- | --- | --- | --- | --- | --- | --- | --- | --- | --- | --- | --- | --- | --- | --- | --- | --- | --- | --- | --- | --- | --- | --- | --- | --- | --- | --- | --- | --- | --- | --- | --- | --- | --- | --- | --- | --- | --- | --- | --- | --- | --- | --- | --- | --- | --- | --- | --- | --- | --- | --- | --- | --- | --- | --- | --- | --- | --- | --- | --- | --- | --- | --- | --- | --- | --- | --- | --- | --- | --- | --- | --- | --- | --- | --- | --- | --- | --- | --- | --- | --- | --- | --- | --- | --- | --- | --- | --- | --- | --- | --- | --- | --- | --- | --- | --- | --- | --- | --- | --- | --- | --- | --- | --- | --- | --- | --- | --- | --- | --- | --- | --- | --- | --- | --- | --- | --- | --- | --- | --- | --- | --- | --- | --- | --- | --- | --- | --- | --- | --- | --- | --- | --- | --- | --- | --- | --- | --- | --- | --- | --- | --- | --- | --- | --- | --- | --- | --- | --- | --- | --- | --- | --- | --- | --- | --- | --- | --- | --- | --- | --- | --- | --- | --- | --- | --- | --- | --- | --- | --- | --- | --- | --- | --- | --- | --- | --- | --- | --- | --- | --- | --- | --- | --- | --- | --- | --- | --- | --- | --- | --- | --- | --- | --- | --- | --- | --- | --- | --- | --- | --- | --- | --- | --- | --- | --- | --- | --- | --- | --- | --- | --- | --- | --- | --- | --- | --- | --- | --- | --- | --- | --- | --- | --- | --- | --- | --- | --- | --- | --- | --- | --- | --- | --- | --- | --- | --- | --- | --- | --- | --- | --- | --- | --- | --- | --- | --- | --- | --- | --- | --- | --- | --- | --- | --- | --- | --- | --- | --- | --- | --- | --- | --- | --- | --- | --- | --- | --- | --- | --- | --- | --- | --- | --- | --- | --- | --- | --- | --- | --- | --- | --- | --- | --- | --- | --- | --- | --- | --- | --- | --- | --- | --- | --- | --- | --- | --- | --- | --- | --- | --- | --- | --- | --- | --- | --- | --- | --- | --- | --- | --- | --- | --- | --- | --- | --- | --- | --- | --- | --- | --- | --- | --- | --- | --- | --- | --- | --- | --- | --- | --- | --- | --- | --- | --- | --- | --- | --- | --- | --- | --- | --- | --- | --- | --- | --- | --- | --- | --- | --- | --- | --- | --- | --- | --- | --- | --- | --- | --- | --- | --- | --- | --- | --- | --- | --- | --- | --- | --- | --- | --- | --- | --- | --- | --- | --- | --- | --- | --- | --- | --- | --- | --- | --- | --- | --- | --- | --- | --- | --- | --- | --- | --- | --- | --- | --- | --- | --- | --- | --- | --- | --- | --- | --- | --- | --- | --- | --- | --- | --- | --- | --- | --- | --- | --- | --- | --- | --- | --- | --- | --- | --- | --- | --- | --- | --- | --- | --- | --- | --- | --- | --- | --- | --- | --- | --- | --- | --- | --- | --- | --- | --- | --- | --- | --- | --- | --- | --- | --- | --- | --- | --- | --- | --- | --- | --- | --- | --- | --- | --- | --- | --- | --- | --- | --- | --- | --- | --- | --- | --- | --- | --- | --- | --- | --- | --- | --- | --- | --- | --- | --- | --- | --- | --- | --- | --- | --- | --- | --- | --- | --- | --- | --- | --- | --- | --- | --- | --- | --- | --- | --- | --- | --- | --- | --- | --- | --- | --- | --- | --- | --- | --- | --- | --- | --- | --- | --- | --- | --- | --- | --- | --- | --- | --- | --- | --- | --- | --- | --- | --- | --- | --- | --- | --- | --- | --- | --- | --- | --- | --- | --- | --- | --- | --- | --- | --- | --- | --- | --- | --- | --- | --- | --- | --- | --- | --- | --- | --- | --- | --- | --- | --- | --- | --- | --- | --- | --- | --- | --- | --- | --- | --- | --- | --- | --- | --- | --- | --- | --- | --- | --- | --- | --- | --- | --- | --- | --- | --- | --- | --- | --- | --- | --- | --- | --- | --- | --- | --- | --- | --- | --- | --- | --- | --- | --- | --- | --- | --- | --- | --- | --- | --- | --- | --- | --- | --- | --- | --- | --- | --- | --- | --- | --- | --- | --- | --- | --- | --- | --- | --- | --- | --- | --- | --- | --- | --- | --- | --- | --- | --- | --- | --- | --- | --- | --- | --- | --- | --- | --- | --- | --- | --- | --- | --- | --- | --- | --- | --- | --- | --- | --- | --- | --- | --- | --- | --- | --- | --- | --- | --- | --- | --- | --- | --- | --- | --- | --- | --- | --- | --- | --- | --- | --- | --- | --- | --- | --- | --- | --- | --- | --- | --- | --- | --- | --- | --- | --- | --- | --- | --- | --- | --- | --- | --- | --- | --- | --- | --- | --- | --- | --- | --- | --- | --- | --- | --- | --- | --- | --- | --- | --- | --- | --- | --- | --- | --- | --- | --- | --- | --- | --- | --- | --- | --- | --- | --- | --- | --- | --- | --- | --- | --- | --- | --- | --- | --- | --- | --- | --- | --- | --- | --- | --- | --- | --- | --- | --- | --- | --- | --- | --- | --- | --- | --- | --- | --- | --- | --- | --- | --- | --- | --- | --- | --- | --- | --- | --- | --- | --- | --- | --- | --- | --- | --- | --- | --- | --- | --- | --- | --- | --- | --- | --- | --- | --- | --- | --- | --- | --- | --- | --- | --- | --- | --- | --- | --- | --- | --- | --- | --- | --- | --- | --- | --- | --- | --- | --- | --- | --- | --- | --- | --- | --- | --- | --- | --- | --- | --- | --- | --- | --- | --- | --- | --- | --- | --- | --- | --- | --- | --- | --- | --- | --- | --- | --- | --- | --- | --- | --- | --- | --- | --- | --- | --- | --- | --- | --- | --- | --- | --- | --- | --- | --- | --- | --- | --- | --- | --- | --- | --- | --- | --- | --- | --- | --- | --- | --- | --- | --- | --- | --- | --- | --- | --- | --- | --- | --- | --- | --- | --- | --- | --- | --- | --- | --- | --- | --- | --- | --- | --- | --- | --- | --- | --- | --- | --- | --- | --- | --- | --- | --- | --- | --- | --- | --- | --- | --- | --- | --- | --- | --- | --- | --- | --- | --- | --- | --- | --- | --- | --- | --- | --- | --- | --- | --- | --- | --- | --- | --- | --- | --- | --- | --- | --- | --- | --- | --- | --- | --- | --- | --- | --- | --- | --- | --- | --- | --- | --- | --- | --- | --- | --- | --- | --- | --- | --- | --- | --- | --- | --- | --- | --- | --- | --- | --- | --- | --- | --- | --- | --- | --- | --- | --- | --- | --- | --- | --- | --- | --- | --- | --- | --- | --- | --- | --- | --- | --- | --- | --- | --- | --- | --- | --- | --- | --- | --- | --- | --- | --- | --- | --- | --- | --- | --- | --- | --- | --- | --- | --- | --- | --- | --- | --- | --- | --- | --- | --- | --- | --- | --- | --- | --- | --- | --- | --- | --- | --- | --- | --- | --- | --- | --- | --- | --- | --- | --- | --- | --- | --- | --- | --- | --- | --- | --- | --- | --- | --- | --- | --- | --- | --- | --- | --- | --- | --- | --- | --- | --- | --- | --- | --- | --- | --- | --- | --- | --- | --- | --- | --- | --- | --- | --- | --- | --- | --- | --- | --- | --- | --- | --- | --- | --- | --- | --- | --- | --- | --- | --- | --- | --- | --- | --- | --- | --- | --- | --- | --- | --- | --- | --- | --- | --- | --- | --- | --- | --- | --- | --- | --- | --- | --- | --- | --- | --- | --- | --- | --- | --- | --- | --- | --- | --- | --- | --- | --- | --- | --- | --- | --- | --- | --- | --- | --- | --- | --- | --- | --- | --- | --- | --- | --- | --- | --- | --- | --- | --- | --- | --- | --- | --- | --- | --- | --- | --- | --- | --- | --- | --- | --- | --- | --- | --- | --- | --- | --- | --- | --- | --- | --- | --- | --- | --- | --- | --- | --- | --- | --- | --- | --- | --- | --- | --- | --- | --- | --- | --- | --- | --- | --- | --- | --- | --- | --- | --- | --- | --- | --- | --- | --- | --- | --- | --- | --- | --- | --- | --- | --- | --- | --- | --- | --- | --- | --- | --- | --- | --- | --- | --- | --- | --- | --- | --- | --- | --- | --- | --- | --- | --- | --- | --- | --- | --- | --- | --- | --- | --- | --- | --- | --- | --- | --- | --- | --- | --- | --- | --- | --- | --- | --- | --- | --- | --- | --- | --- | --- | --- | --- | --- | --- | --- | --- | --- | --- | --- | --- | --- | --- | --- | --- | --- | --- | --- | --- | --- | --- | --- | --- | --- | --- | --- | --- | --- | --- | --- | --- | --- | --- | --- | --- | --- | --- | --- | --- | --- | --- | --- | --- | --- | --- | --- | --- | --- | --- | --- | --- | --- | --- | --- | --- | --- | --- | --- | --- | --- | --- | --- | --- | --- | --- | --- | --- | --- | --- | --- | --- | --- | --- | --- | --- | --- | --- | --- | --- | --- | --- | --- | --- | --- | --- | --- | --- | --- | --- | --- | --- | --- | --- | --- | --- | --- | --- | --- | --- | --- | --- | --- | --- | --- | --- | --- | --- | --- | --- | --- | --- | --- | --- | --- | --- | --- | --- | --- | --- | --- | --- | --- | --- | --- | --- | --- | --- | --- | --- | --- | --- | --- | --- | --- | --- | --- | --- | --- | --- | --- | --- | --- | --- | --- | --- | --- | --- | --- | --- | --- | --- | --- | --- | --- | --- | --- | --- | --- | --- | --- | --- | --- | --- | --- | --- | --- | --- | --- | --- | --- | --- | --- | --- | --- | --- | --- | --- | --- | --- | --- | --- | --- | --- | --- | --- | --- | --- | --- | --- | --- | --- | --- | --- | --- | --- | --- | --- | --- | --- | --- | --- | --- | --- | --- | --- | --- | --- | --- | --- | --- | --- | --- | --- | --- | --- | --- | --- | --- | --- | --- | --- | --- | --- | --- | --- | --- | --- | --- | --- | --- | --- | --- | --- | --- | --- | --- | --- | --- | --- | --- | --- | --- | --- | --- | --- | --- | --- | --- | --- | --- | --- | --- | --- | --- | --- | --- | --- | --- | --- | --- | --- | --- | --- | --- | --- | --- | --- | --- | --- | --- | --- | --- | --- | --- | --- | --- | --- | --- | --- | --- | --- | --- | --- | --- | --- | --- | --- | --- | --- | --- | --- | --- | --- | --- | --- | --- | --- | --- | --- | --- | --- | --- | --- | --- | --- | --- | --- | --- | --- | --- | --- | --- | --- | --- | --- | --- | --- | --- | --- | --- | --- | --- | --- | --- | --- | --- | --- | --- | --- | --- | --- | --- | --- | --- | --- | --- | --- | --- | --- | --- | --- | --- | --- | --- | --- | --- | --- | --- | --- | --- | --- | --- | --- | --- | --- | --- | --- | --- | --- | --- | --- | --- | --- | --- | --- | --- | --- | --- | --- | --- | --- | --- | --- | --- | --- | --- | --- | --- | --- | --- | --- | --- | --- | --- | --- | --- | --- | --- | --- | --- | --- | --- | --- | --- | --- | --- | --- | --- | --- | --- | --- | --- | --- | --- | --- | --- | --- | --- | --- | --- | --- | --- | --- | --- | --- | --- | --- | --- | --- | --- | --- | --- | --- | --- | --- | --- | --- | --- | --- | --- | --- | --- | --- | --- | --- | --- | --- | --- | --- | --- | --- | --- | --- | --- | --- | --- | --- | --- | --- | --- | --- | --- | --- | --- | --- | --- | --- | --- | --- | --- | --- | --- | --- | --- | --- | --- | --- | --- | --- | --- | --- | --- | --- | --- | --- | --- | --- | --- | --- | --- | --- | --- | --- | --- | --- | --- | --- | --- | --- | --- | --- | --- | --- | --- | --- | --- | --- | --- | --- | --- | --- | --- | --- | --- | --- | --- | --- | --- | --- | --- | --- | --- | --- | --- | --- | --- | --- | --- | --- | --- | --- | --- | --- | --- | --- | --- | --- | --- | --- | --- | --- | --- | --- | --- | --- | --- | --- | --- | --- | --- | --- | --- | --- | --- | --- | --- | --- | --- | --- | --- | --- | --- | --- | --- | --- | --- | --- | --- | --- | --- | --- | --- | --- | --- | --- | --- | --- | --- | --- | --- | --- | --- | --- | --- | --- | --- | --- | --- | --- | --- | --- | --- | --- | --- | --- | --- | --- | --- | --- | --- | --- | --- | --- | --- | --- | --- | --- | --- | --- | --- | --- | --- | --- | --- | --- | --- | --- | --- | --- | --- | --- | --- | --- | --- | --- | --- | --- | --- | --- | --- | --- | --- | --- | --- | --- | --- | --- | --- | --- | --- | --- | --- | --- | --- | --- | --- | --- | --- | --- | --- | --- | --- | --- | --- | --- | --- | --- | --- | --- | --- | --- | --- | --- | --- | --- | --- | --- | --- | --- | --- | --- | --- | --- | --- | --- | --- | --- | --- | --- | --- | --- | --- | --- | --- | --- | --- | --- | --- | --- | --- | --- | --- | --- | --- | --- | --- | --- | --- | --- | --- | --- | --- | --- | --- | --- | --- | --- | --- | --- | --- | --- | --- | --- | --- | --- | --- | --- | --- | --- | --- | --- | --- | --- | --- | --- | --- | --- | --- | --- | --- | --- | --- | --- | --- | --- | --- | --- | --- | --- | --- | --- | --- | --- | --- | --- | --- | --- | --- | --- | --- | --- | --- | --- | --- | --- | --- | --- | --- | --- | --- | --- | --- | --- | --- | --- | --- | --- | --- | --- | --- | --- | --- | --- | --- | --- | --- | --- | --- | --- | --- | --- | --- | --- | --- | --- | --- | --- | --- | --- | --- | --- | --- | --- | --- | --- | --- | --- | --- | --- | --- | --- | --- | --- | --- | --- | --- | --- | --- | --- | --- | --- | --- | --- | --- | --- | --- | --- | --- | --- | --- | --- | --- | --- | --- | --- | --- | --- | --- | --- | --- | --- | --- | --- | --- | --- | --- | --- | --- | --- | --- | --- | --- | --- | --- | --- | --- | --- | --- | --- | --- | --- | --- | --- | --- | --- | --- | --- | --- | --- | --- | --- | --- | --- | --- | --- | --- | --- | --- | --- | --- | --- | --- | --- | --- | --- | --- | --- | --- | --- | --- | --- | --- | --- | --- | --- | --- | --- | --- | --- | --- | --- | --- | --- | --- | --- | --- | --- | --- | --- | --- | --- | --- | --- | --- | --- | --- | --- | --- | --- | --- | --- | --- | --- | --- | --- | --- | --- | --- | --- | --- | --- | --- | --- | --- | --- | --- | --- | --- | --- | --- | --- | --- | --- | --- | --- | --- | --- | --- | --- | --- | --- | --- | --- | --- | --- | --- | --- | --- | --- | --- | --- | --- | --- | --- | --- | --- | --- | --- | --- | --- | --- | --- | --- | --- | --- | --- | --- | --- | --- | --- | --- | --- | --- | --- | --- | --- | --- | --- | --- | --- | --- | --- | --- | --- | --- | --- | --- | --- | --- | --- | --- | --- | --- | --- | --- | --- | --- | --- | --- | --- | --- | --- | --- | --- | --- | --- | --- | --- | --- | --- | --- | --- | --- | --- | --- | --- | --- | --- | --- | --- | --- | --- | --- | --- | --- | --- | --- | --- | --- | --- | --- | --- | --- | --- | --- | --- | --- | --- | --- | --- | --- | --- | --- | --- | --- | --- | --- | --- | --- | --- | --- | --- | --- | --- | --- | --- | --- | --- | --- | --- | --- | --- | --- | --- | --- | --- | --- | --- | --- | --- | --- | --- | --- | --- | --- | --- | --- | --- | --- | --- | --- | --- | --- | --- | --- | --- | --- | --- | --- | --- | --- | --- | --- | --- | --- | --- | --- | --- | --- | --- | --- | --- | --- | --- | --- | --- | --- | --- | --- | --- | --- | --- | --- | --- | --- | --- | --- | --- | --- | --- | --- | --- | --- | --- | --- | --- | --- | --- | --- | --- | --- | --- | --- | --- | --- | --- | --- | --- | --- | --- | --- | --- | --- | --- | --- | --- | --- | --- | --- | --- | --- | --- | --- | --- | --- | --- | --- | --- | --- | --- | --- | --- | --- | --- | --- | --- | --- | --- | --- | --- | --- | --- | --- | --- | --- | --- | --- | --- | --- | --- | --- | --- | --- | --- | --- | --- | --- | --- | --- | --- | --- | --- | --- | --- | --- | --- | --- | --- | --- | --- | --- | --- | --- | --- | --- | --- | --- | --- | --- | --- | --- | --- | --- | --- | --- | --- | --- | --- | --- | --- | --- | --- | --- | --- | --- | --- | --- | --- | --- | --- | --- | --- | --- | --- | --- | --- | --- | --- | --- | --- | --- | --- | --- | --- | --- | --- | --- | --- | --- | --- | --- | --- | --- | --- | --- | --- | --- | --- | --- | --- | --- | --- | --- | --- | --- | --- | --- | --- | --- | --- | --- | --- | --- | --- | --- | --- | --- | --- | --- | --- | --- | --- | --- | --- | --- | --- | --- | --- | --- | --- | --- | --- | --- | --- | --- | --- | --- | --- | --- | --- | --- | --- | --- | --- | --- | --- | --- | --- | --- | --- | --- | --- | --- | --- | --- | --- | --- | --- | --- | --- | --- | --- | --- | --- | --- | --- | --- | --- | --- | --- | --- | --- | --- | --- | --- | --- | --- | --- | --- | --- | --- | --- | --- | --- | --- | --- | --- | --- | --- | --- | --- | --- | --- | --- | --- | --- | --- | --- | --- | --- | --- | --- | --- | --- | --- | --- | --- | --- | --- | --- | --- | --- | --- | --- | --- | --- | --- | --- | --- | --- | --- | --- | --- | --- | --- | --- | --- | --- | --- | --- | --- | --- | --- | --- | --- | --- | --- | --- | --- | --- | --- | --- | --- | --- | --- | --- | --- | --- | --- | --- | --- | --- | --- | --- | --- | --- | --- | --- | --- | --- | --- | --- | --- | --- | --- | --- | --- | --- | --- | --- | --- | --- | --- | --- | --- | --- | --- | --- | --- | --- | --- | --- | --- | --- | --- | --- | --- | --- | --- | --- | --- | --- | --- | --- | --- | --- | --- | --- | --- | --- | --- | --- | --- | --- | --- | --- | --- | --- | --- | --- | --- | --- | --- | --- | --- | --- | --- | --- | --- | --- | --- | --- | --- | --- | --- | --- | --- | --- | --- | --- | --- | --- | --- | --- | --- | --- | --- | --- | --- | --- | --- | --- | --- | --- | --- | --- | --- | --- | --- | --- | --- | --- | --- | --- | --- | --- | --- | --- | --- | --- | --- | --- | --- | --- | --- | --- | --- | --- | --- | --- | --- | --- | --- | --- | --- | --- | --- | --- | --- | --- | --- | --- | --- | --- | --- | --- | --- | --- | --- | --- | --- | --- | --- | --- | --- | --- | --- | --- | --- | --- | --- | --- | --- | --- | --- | --- | --- | --- | --- | --- | --- | --- | --- | --- | --- | --- | --- | --- | --- | --- | --- | --- | --- | --- | --- | --- | --- | --- | --- | --- | --- | --- | --- | --- | --- | --- | --- | --- | --- | --- | --- | --- | --- | --- | --- | --- | --- | --- | --- | --- | --- | --- | --- | --- | --- | --- | --- | --- | --- | --- | --- | --- | --- | --- | --- | --- | --- | --- | --- | --- | --- | --- | --- | --- | --- | --- | --- | --- | --- | --- | --- | --- | --- | --- | --- | --- | --- | --- | --- | --- | --- | --- | --- | --- | --- | --- | --- | --- | --- | --- | --- | --- | --- | --- | --- | --- | --- | --- | --- | --- | --- | --- | --- | --- | --- | --- | --- | --- | --- | --- | --- | --- | --- | --- | --- | --- | --- | --- | --- | --- | --- | --- | --- | --- | --- | --- | --- | --- | --- | --- | --- | --- | --- | --- | --- | --- | --- | --- | --- | --- | --- | --- | --- | --- | --- | --- | --- | --- | --- | --- | --- | --- | --- | --- | --- | --- | --- | --- | --- | --- | --- | --- | --- | --- | --- | --- | --- | --- | --- | --- | --- | --- | --- | --- | --- | --- | --- | --- | --- | --- | --- | --- | --- | --- | --- | --- | --- | --- | --- | --- | --- | --- | --- | --- | --- | --- | --- | --- | --- | --- | --- | --- | --- | --- | --- | --- | --- | --- | --- | --- | --- | --- | --- | --- | --- | --- | --- | --- | --- | --- | --- | --- | --- | --- | --- | --- | --- | --- | --- | --- | --- | --- | --- | --- | --- | --- | --- | --- | --- | --- | --- | --- | --- | --- | --- | --- | --- | --- | --- | --- | --- | --- | --- | --- | --- | --- | --- | --- | --- | --- | --- | --- | --- | --- | --- | --- | --- | --- | --- | --- | --- | --- | --- | --- | --- | --- | --- | --- | --- | --- | --- | --- | --- | --- | --- | --- | --- | --- | --- | --- | --- | --- | --- | --- | --- | --- | --- | --- | --- | --- | --- | --- | --- | --- | --- | --- | --- | --- | --- | --- | --- | --- | --- | --- | --- | --- | --- | --- | --- | --- | --- | --- | --- | --- | --- | --- | --- | --- | --- | --- | --- | --- | --- | --- | --- | --- | --- | --- | --- | --- | --- | --- | --- | --- | --- | --- | --- | --- | --- | --- | --- | --- | --- | --- | --- | --- | --- | --- | --- | --- | --- | --- | --- | --- | --- | --- | --- | --- | --- | --- | --- | --- | --- | --- | --- | --- | --- | --- | --- | --- | --- | --- | --- | --- | --- | --- | --- | --- | --- | --- | --- | --- | --- | --- | --- | --- | --- | --- | --- | --- | --- | --- | --- | --- | --- | --- | --- | --- | --- | --- | --- | --- | --- | --- | --- | --- | --- | --- | --- | --- | --- | --- | --- | --- | --- | --- | --- | --- | --- | --- | --- | --- | --- | --- | --- | --- | --- | --- | --- | --- | --- | --- | --- | --- | --- | --- | --- | --- | --- | --- | --- | --- | --- | --- | --- | --- | --- | --- | --- | --- | --- | --- | --- | --- | --- | --- | --- | --- | --- | --- | --- | --- | --- | --- | --- | --- | --- | --- | --- | --- | --- | --- | --- | --- | --- | --- | --- | --- | --- | --- | --- | --- | --- | --- | --- | --- | --- | --- | --- | --- | --- | --- | --- | --- | --- | --- | --- | --- | --- | --- | --- | --- | --- | --- | --- | --- | --- | --- | --- | --- | --- | --- | --- | --- | --- | --- | --- | --- | --- | --- | --- | --- | --- | --- | --- | --- | --- | --- | --- | --- | --- | --- | --- | --- | --- | --- | --- | --- | --- | --- | --- | --- | --- | --- | --- | --- | --- | --- | --- | --- | --- | --- | --- | --- | --- | --- | --- | --- | --- | --- | --- | --- | --- | --- | --- | --- | --- | --- | --- | --- | --- | --- | --- | --- | --- | --- | --- | --- | --- | --- | --- | --- | --- | --- | --- | --- | --- | --- | --- | --- | --- | --- | --- | --- | --- | --- | --- | --- | --- | --- | --- | --- | --- | --- | --- | --- | --- | --- | --- | --- | --- | --- | --- | --- | --- | --- | --- | --- | --- | --- | --- | --- | --- | --- | --- | --- | --- | --- | --- | --- | --- | --- | --- | --- | --- | --- | --- | --- | --- | --- | --- | --- | --- | --- | --- | --- | --- | --- | --- | --- | --- | --- | --- | --- | --- | --- | --- | --- | --- | --- | --- | --- | --- | --- | --- | --- | --- | --- | --- | --- | --- | --- | --- | --- | --- | --- | --- | --- | --- | --- | --- | --- | --- | --- | --- | --- | --- | --- | --- | --- | --- | --- | --- | --- | --- | --- | --- | --- | --- | --- | --- | --- | --- | --- | --- | --- | --- | --- | --- | --- | --- | --- | --- | --- | --- | --- | --- | --- | --- | --- | --- | --- | --- | --- | --- | --- | --- | --- | --- | --- | --- | --- | --- | --- | --- | --- | --- | --- | --- | --- | --- | --- | --- | --- | --- | --- | --- | --- | --- | --- | --- | --- | --- | --- | --- | --- | --- | --- | --- | --- | --- | --- | --- | --- | --- | --- | --- | --- | --- | --- | --- | --- | --- | --- | --- | --- | --- | --- | --- | --- | --- | --- | --- | --- | --- | --- | --- | --- | --- | --- | --- | --- | --- | --- | --- | --- | --- | --- | --- | --- | --- | --- | --- | --- | --- | --- | --- | --- | --- | --- | --- | --- | --- | --- | --- | --- | --- | --- | --- | --- | --- | --- | --- | --- | --- | --- | --- | --- | --- | --- | --- | --- | --- | --- | --- | --- | --- | --- | --- | --- | --- | --- | --- | --- | --- | --- | --- | --- | --- | --- | --- | --- | --- | --- | --- | --- | --- | --- | --- | --- | --- | --- | --- | --- | --- | --- | --- | --- | --- | --- | --- | --- | --- | --- | --- | --- | --- | --- | --- | --- | --- | --- | --- | --- | --- | --- | --- | --- | --- | --- | --- | --- | --- | --- | --- | --- | --- | --- | --- | --- | --- | --- | --- | --- | --- | --- | --- | --- | --- | --- | --- | --- | --- | --- | --- | --- | --- | --- | --- | --- | --- | --- | --- | --- | --- | --- | --- | --- | --- | --- | --- | --- | --- | --- | --- | --- | --- | --- | --- | --- | --- | --- | --- | --- | --- | --- | --- | --- | --- | --- | --- | --- | --- | --- | --- | --- | --- | --- | --- | --- | --- | --- | --- | --- | --- | --- | --- | --- | --- | --- | --- | --- | --- | --- | --- | --- | --- | --- | --- | --- | --- | --- | --- | --- | --- | --- | --- | --- | --- | --- | --- | --- | --- | --- | --- | --- | --- | --- | --- | --- | --- | --- | --- | --- | --- | --- | --- | --- | --- | --- | --- | --- | --- | --- | --- | --- | --- | --- | --- | --- | --- | --- | --- | --- | --- | --- | --- | --- | --- | --- | --- | --- | --- | --- | --- | --- | --- | --- | --- | --- | --- | --- | --- | --- | --- | --- | --- | --- | --- | --- | --- | --- | --- | --- | --- | --- | --- | --- | --- | --- | --- | --- | --- | --- | --- | --- | --- | --- | --- | --- | --- | --- | --- | --- | --- | --- | --- | --- | --- | --- | --- | --- | --- | --- | --- | --- | --- | --- | --- | --- | --- | --- | --- | --- | --- | --- | --- | --- | --- | --- | --- | --- | --- | --- | --- | --- | --- | --- | --- | --- | --- | --- | --- | --- | --- | --- | --- | --- | --- | --- | --- | --- | --- | --- | --- | --- | --- | --- | --- | --- | --- | --- | --- | --- | --- | --- | --- | --- | --- | --- | --- | --- | --- | --- | --- | --- | --- | --- | --- | --- | --- | --- | --- | --- | --- | --- | --- | --- | --- | --- | --- | --- | --- | --- | --- | --- | --- | --- | --- | --- | --- | --- | --- | --- | --- | --- | --- | --- | --- | --- | --- | --- | --- | --- | --- | --- | --- | --- | --- | --- | --- | --- | --- | --- | --- | --- | --- | --- | --- | --- | --- | --- | --- | --- | --- | --- | --- | --- | --- | --- | --- | --- | --- | --- | --- | --- | --- | --- | --- | --- | --- | --- | --- | --- | --- | --- | --- | --- | --- | --- | --- | --- | --- | --- | --- | --- | --- | --- | --- | --- | --- | --- | --- | --- | --- | --- | --- | --- | --- | --- | --- | --- | --- | --- | --- | --- | --- | --- | --- | --- | --- | --- | --- | --- | --- | --- | --- | --- | --- | --- | --- | --- | --- | --- | --- | --- | --- | --- | --- | --- | --- | --- | --- | --- | --- | --- | --- | --- | --- | --- | --- | --- | --- | --- | --- | --- | --- | --- | --- | --- | --- | --- | --- | --- | --- | --- | --- | --- | --- | --- | --- | --- | --- | --- | --- | --- | --- | --- | --- | --- | --- | --- | --- | --- | --- | --- | --- | --- | --- | --- | --- | --- | --- | --- | --- | --- | --- | --- | --- | --- | --- | --- | --- | --- | --- | --- | --- | --- | --- | --- | --- | --- | --- | --- | --- | --- | --- | --- | --- | --- | --- | --- | --- | --- | --- | --- | --- | --- | --- | --- | --- | --- | --- | --- | --- | --- | --- | --- | --- | --- | --- | --- | --- | --- | --- | --- | --- | --- | --- | --- | --- | --- | --- | --- | --- | --- | --- | --- | --- | --- | --- | --- | --- | --- | --- | --- | --- | --- | --- | --- | --- | --- | --- | --- | --- | --- | --- | --- | --- | --- | --- | --- | --- | --- | --- | --- | --- | --- | --- | --- | --- | --- | --- | --- | --- | --- | --- | --- | --- | --- | --- | --- | --- | --- | --- | --- | --- | --- | --- | --- | --- | --- | --- | --- | --- | --- | --- | --- | --- | --- | --- | --- | --- | --- | --- | --- | --- | --- | --- | --- | --- | --- | --- | --- | --- | --- | --- | --- | --- | --- | --- | --- | --- | --- |
