# Supplementary material for: Morpho-biometric characterization of indigenous chicken ecotypes in north-western Ethiopia
Source: PLoS One. 2023 Jun 2;18(6):e0286299. doi: 10.1371/journal.pone.0286299 (PMC10237646; doi:10.1371/journal.pone.0286299)
Supplement: S5 File — (DOCX) [file pone.0286299.s005.docx]

| \| \| \| **Discriminant Analysis Results** \| \| --- \| \| \| --- \| --- \| \| \| \| **The DISCRIM Procedure** \| \| --- \| \| \| --- \| --- \| \| \| \| Total Sample Size \| 323 \| DF Total \| 322 \| \| --- \| --- \| --- \| --- \| \| Variables \| 11 \| DF Within Classes \| 317 \| \| Classes \| 6 \| DF Between Classes \| 5 \| \| \| --- \| --- \| --- \| --- \| --- \| --- \| --- \| --- \| --- \| --- \| --- \| --- \| --- \| \| \| Number of Observations Read \| 323 \| \| --- \| --- \| \| Number of Observations Used \| 323 \| \| \| \| **Class Level Information** \| \| \| \| \| \| \| --- \| --- \| --- \| --- \| --- \| --- \| \| **DIST** \| **Variable Name** \| **Frequency** \| **Weight** \| **Proportion** \| **Prior Probability** \| \| 1 \| 1 \| 50 \| 50.0000 \| 0.154799 \| 0.154799 \| \| 2 \| 2 \| 63 \| 63.0000 \| 0.195046 \| 0.195046 \| \| 3 \| 3 \| 43 \| 43.0000 \| 0.133127 \| 0.133127 \| \| 4 \| 4 \| 55 \| 55.0000 \| 0.170279 \| 0.170279 \| \| 5 \| 5 \| 60 \| 60.0000 \| 0.185759 \| 0.185759 \| \| 6 \| 6 \| 52 \| 52.0000 \| 0.160991 \| 0.160991 \| \| \| \| **Pooled Covariance Matrix Information** \| \| \| --- \| --- \| \| **Covariance Matrix Rank** \| **Natural Log of the Determinant of the Covariance Matrix** \| \| 11 \| -3.00864 \| \| \| \|  \| \| \| \| --- \| --- \| --- \| --- \| --- \| --- \| --- \| --- \| --- \| --- \| --- \| --- \| --- \| --- \| --- \| --- \| --- \| --- \| --- \| --- \| --- \| --- \| --- \| --- \| --- \| --- \| --- \| --- \| --- \| --- \| --- \| --- \| --- \| --- \| --- \| --- \| --- \| --- \| --- \| --- \| --- \| --- \| --- \| --- \| --- \| --- \| --- \| --- \| --- \| --- \| --- \| --- \| --- \| --- \| --- \| --- \| --- \| --- \| --- \| --- \| --- \| --- \| --- \| --- \| --- \| --- \| --- \| --- \| --- \| --- \| --- \| --- \| --- \| --- \| --- \| --- \| --- \| --- \| --- \| --- \| --- \| --- \| \| \| \| **Discriminant Analysis Results** \| \| --- \| \| \| --- \| --- \| \| \| \| **The DISCRIM Procedure** \| \| --- \| \| \| --- \| --- \| \| \| \| **Generalized Squared Distance to DIST** \| \| \| \| \| \| \| \| --- \| --- \| --- \| --- \| --- \| --- \| --- \| \| **From DIST** \| **1** \| **2** \| **3** \| **4** \| **5** \| **6** \| \| 1 \| 3.73126 \| 6.88817 \| 8.57625 \| 5.67951 \| 8.42633 \| 12.40055 \| \| 2 \| 7.35039 \| 3.26904 \| 8.17548 \| 9.65200 \| 12.18385 \| 15.69982 \| \| 3 \| 8.27461 \| 7.41162 \| 4.03290 \| 12.05359 \| 9.50651 \| 16.42457 \| \| 4 \| 5.87013 \| 9.38040 \| 12.54585 \| 3.54064 \| 11.81147 \| 15.20377 \| \| 5 \| 8.79098 \| 12.08627 \| 10.17279 \| 11.98549 \| 3.36662 \| 12.38932 \| \| 6 \| 12.47899 \| 15.31603 \| 16.80466 \| 15.09159 \| 12.10312 \| 3.65282 \| \| \| --- \| --- \| --- \| --- \| --- \| --- \| --- \| --- \| --- \| --- \| --- \| --- \| --- \| --- \| --- \| --- \| --- \| --- \| --- \| --- \| --- \| --- \| --- \| --- \| --- \| --- \| --- \| --- \| --- \| --- \| --- \| --- \| --- \| --- \| --- \| --- \| --- \| --- \| --- \| --- \| --- \| --- \| --- \| --- \| --- \| --- \| --- \| --- \| --- \| --- \| --- \| --- \| --- \| --- \| --- \| --- \| --- \| \| \|  \| \| \| \| \| \| **Discriminant Analysis Results** \| \| --- \| \| \| --- \| --- \| \| \| \| **The DISCRIM Procedure** \| \| --- \| \| \| --- \| --- \| \| \| \| **Univariate Test Statistics** \| \| \| \| \| \| \| \| \| --- \| --- \| --- \| --- \| --- \| --- \| --- \| --- \| \| **F Statistics, Num DF=5, Den DF=317** \| \| \| \| \| \| \| \| \| **Variable** \| **Total Standard Deviation** \| **Pooled Standard Deviation** \| **Between Standard Deviation** \| **R-Square** \| **R-Square / (1-RSq)** \| **F Value** \| **Pr > F** \| \| WS \| 3.4428 \| 2.7969 \| 2.2286 \| 0.3503 \| 0.5391 \| 34.18 \| <.0001 \| \| SL \| 1.0723 \| 0.7781 \| 0.8140 \| 0.4816 \| 0.9291 \| 58.90 \| <.0001 \| \| BL \| 3.4240 \| 2.9017 \| 2.0270 \| 0.2930 \| 0.4143 \| 26.27 \| <.0001 \| \| BKL \| 0.3298 \| 0.3201 \| 0.0972 \| 0.0726 \| 0.0783 \| 4.97 \| 0.0002 \| \| NL \| 1.3061 \| 1.2273 \| 0.5163 \| 0.1306 \| 0.1503 \| 9.53 \| <.0001 \| \| CL \| 1.8485 \| 1.6644 \| 0.9084 \| 0.2019 \| 0.2529 \| 16.03 \| <.0001 \| \| SC \| 0.8611 \| 0.7454 \| 0.4824 \| 0.2623 \| 0.3556 \| 22.55 \| <.0001 \| \| CC \| 2.4867 \| 2.0270 \| 1.5995 \| 0.3459 \| 0.5287 \| 33.52 \| <.0001 \| \| TC \| 1.5834 \| 1.4399 \| 0.7468 \| 0.1859 \| 0.2284 \| 14.48 \| <.0001 \| \| CH \| 1.5228 \| 1.4594 \| 0.5155 \| 0.0958 \| 0.1060 \| 6.72 \| <.0001 \| \| BW \| 0.3212 \| 0.2706 \| 0.1930 \| 0.3017 \| 0.4320 \| 27.39 \| <.0001 \| \| \| --- \| --- \| --- \| --- \| --- \| --- \| --- \| --- \| --- \| --- \| --- \| --- \| --- \| --- \| --- \| --- \| --- \| --- \| --- \| --- \| --- \| --- \| --- \| --- \| --- \| --- \| --- \| --- \| --- \| --- \| --- \| --- \| --- \| --- \| --- \| --- \| --- \| --- \| --- \| --- \| --- \| --- \| --- \| --- \| --- \| --- \| --- \| --- \| --- \| --- \| --- \| --- \| --- \| --- \| --- \| --- \| --- \| --- \| --- \| --- \| --- \| --- \| --- \| --- \| --- \| --- \| --- \| --- \| --- \| --- \| --- \| --- \| --- \| --- \| --- \| --- \| --- \| --- \| --- \| --- \| --- \| --- \| --- \| --- \| --- \| --- \| --- \| --- \| --- \| --- \| --- \| --- \| --- \| --- \| --- \| --- \| --- \| --- \| --- \| --- \| --- \| --- \| --- \| --- \| --- \| --- \| --- \| --- \| --- \| --- \| --- \| --- \| --- \| \| \| **Average R-Square** \| \| \| --- \| --- \| \| Unweighted \| 0.247417 \| \| Weighted by Variance \| 0.2897028 \| \| \| \| **Multivariate Statistics and F Approximations** \| \| \| \| \| \| \| --- \| --- \| --- \| --- \| --- \| --- \| \| **S=5 M=2.5 N=152.5** \| \| \| \| \| \| \| **Statistic** \| **Value** \| **F Value** \| **Num DF** \| **Den DF** \| **Pr > F** \| \| Wilks' Lambda \| 0.10441015 \| 16.30 \| 55 \| 1424.6 \| <.0001 \| \| Pillai's Trace \| 1.69858167 \| 14.55 \| 55 \| 1555 \| <.0001 \| \| Hotelling-Lawley Trace \| 3.15320810 \| 17.52 \| 55 \| 1012.2 \| <.0001 \| \| Roy's Greatest Root \| 1.33592326 \| 37.77 \| 11 \| 311 \| <.0001 \| \| **NOTE: F Statistic for Roy's Greatest Root is an upper bound.** \| \| \| \| \| \| \| \| \|  \| \| \| \| \| \| **Discriminant Analysis Results** \| \| --- \| \| \| --- \| --- \| \| \| \| **The DISCRIM Procedure Canonical Discriminant Analysis** \| \| --- \| \| \| --- \| --- \| \| \| \|  \| **Canonical Correlation** \| **Adjusted Canonical Correlation** \| **Approximate Standard Error** \| **Squared Canonical Correlation** \| **Eigenvalues of Inv(E)*H = CanRsq/(1-CanRsq)** \| \| \| \| **Test of H0: The canonical correlations in the current row and all that follow are zero** \| \| \| \| \| \| --- \| --- \| --- \| --- \| --- \| --- \| --- \| --- \| --- \| --- \| --- \| --- \| --- \| --- \| \| **Eigenvalue** \| **Difference** \| **Proportion** \| **Cumulative** \| **Likelihood Ratio** \| **Approximate F Value** \| **Num DF** \| **Den DF** \| **Pr > F** \| \| 1 \| 0.756243 \| 0.739359 \| 0.023857 \| 0.571904 \| 1.3359 \| 0.5129 \| 0.4237 \| 0.4237 \| 0.10441015 \| 16.30 \| 55 \| 1424.6 \| <.0001 \| \| 2 \| 0.671903 \| 0.649113 \| 0.030569 \| 0.451453 \| 0.8230 \| 0.1554 \| 0.2610 \| 0.6847 \| 0.24389411 \| 13.18 \| 40 \| 1169.8 \| <.0001 \| \| 3 \| 0.632725 \| 0.632133 \| 0.033418 \| 0.400342 \| 0.6676 \| 0.4361 \| 0.2117 \| 0.8964 \| 0.44461869 \| 10.70 \| 27 \| 903.08 \| <.0001 \| \| 4 \| 0.433564 \| 0.413367 \| 0.045252 \| 0.187978 \| 0.2315 \| 0.1363 \| 0.0734 \| 0.9698 \| 0.74145320 \| 6.25 \| 16 \| 620 \| <.0001 \| \| 5 \| 0.294797 \| 0.276669 \| 0.050885 \| 0.086905 \| 0.0952 \|  \| 0.0302 \| 1.0000 \| 0.91309464 \| 4.23 \| 7 \| 311 \| 0.0002 \| \| \| --- \| --- \| --- \| --- \| --- \| --- \| --- \| --- \| --- \| --- \| --- \| --- \| --- \| --- \| --- \| --- \| --- \| --- \| --- \| --- \| --- \| --- \| --- \| --- \| --- \| --- \| --- \| --- \| --- \| --- \| --- \| --- \| --- \| --- \| --- \| --- \| --- \| --- \| --- \| --- \| --- \| --- \| --- \| --- \| --- \| --- \| --- \| --- \| --- \| --- \| --- \| --- \| --- \| --- \| --- \| --- \| --- \| --- \| --- \| --- \| --- \| --- \| --- \| --- \| --- \| --- \| --- \| --- \| --- \| --- \| --- \| --- \| --- \| --- \| --- \| --- \| --- \| --- \| --- \| --- \| --- \| --- \| --- \| --- \| --- \| --- \| --- \| --- \| --- \| --- \| --- \| --- \| --- \| --- \| \| \|  \| \| \| \| \| \| **Discriminant Analysis Results** \| \| --- \| \| \| --- \| --- \| \| \| \| **The DISCRIM Procedure Canonical Discriminant Analysis** \| \| --- \| \| \| --- \| --- \| \| \| \| **Total Canonical Structure** \| \| \| \| \| \| \| --- \| --- \| --- \| --- \| --- \| --- \| \| **Variable** \| **Can1** \| **Can2** \| **Can3** \| **Can4** \| **Can5** \| \| WS \| 0.699298 \| 0.312982 \| -0.148843 \| -0.265641 \| -0.221087 \| \| SL \| 0.864159 \| 0.272224 \| -0.178664 \| 0.210116 \| -0.008979 \| \| BL \| 0.559735 \| 0.479023 \| -0.071182 \| -0.208001 \| 0.014980 \| \| BKL \| 0.098289 \| 0.291930 \| -0.203572 \| -0.219126 \| 0.186366 \| \| NL \| 0.286830 \| -0.257944 \| 0.245880 \| -0.352088 \| -0.263466 \| \| CL \| 0.338936 \| -0.302981 \| -0.441006 \| 0.288229 \| 0.119141 \| \| SC \| 0.021365 \| 0.490005 \| -0.602981 \| 0.206709 \| 0.029442 \| \| CC \| 0.714473 \| 0.243230 \| -0.055403 \| -0.235007 \| 0.423736 \| \| TC \| 0.536501 \| -0.080136 \| -0.111796 \| -0.250637 \| 0.136075 \| \| CH \| 0.284404 \| -0.181671 \| -0.016960 \| 0.428602 \| 0.002804 \| \| BW \| 0.593058 \| 0.445777 \| 0.132943 \| -0.092852 \| 0.156914 \| \| \| --- \| --- \| --- \| --- \| --- \| --- \| --- \| --- \| --- \| --- \| --- \| --- \| --- \| --- \| --- \| --- \| --- \| --- \| --- \| --- \| --- \| --- \| --- \| --- \| --- \| --- \| --- \| --- \| --- \| --- \| --- \| --- \| --- \| --- \| --- \| --- \| --- \| --- \| --- \| --- \| --- \| --- \| --- \| --- \| --- \| --- \| --- \| --- \| --- \| --- \| --- \| --- \| --- \| --- \| --- \| --- \| --- \| --- \| --- \| --- \| --- \| --- \| --- \| --- \| --- \| --- \| --- \| --- \| --- \| --- \| --- \| --- \| --- \| --- \| --- \| --- \| --- \| --- \| --- \| \| \| **Between Canonical Structure** \| \| \| \| \| \| \| --- \| --- \| --- \| --- \| --- \| --- \| \| **Variable** \| **Can1** \| **Can2** \| **Can3** \| **Can4** \| **Can5** \| \| WS \| 0.893549 \| 0.355320 \| -0.159125 \| -0.194600 \| -0.110124 \| \| SL \| 0.941677 \| 0.263561 \| -0.162891 \| 0.131268 \| -0.003814 \| \| BL \| 0.782071 \| 0.594654 \| -0.083212 \| -0.166617 \| 0.008159 \| \| BKL \| 0.275800 \| 0.727804 \| -0.477927 \| -0.352513 \| 0.203853 \| \| NL \| 0.600162 \| -0.479529 \| 0.430448 \| -0.422364 \| -0.214897 \| \| CL \| 0.570509 \| -0.453112 \| -0.621074 \| 0.278147 \| 0.078175 \| \| SC \| 0.031546 \| 0.642819 \| -0.744904 \| 0.174982 \| 0.016946 \| \| CC \| 0.918745 \| 0.277889 \| -0.059606 \| -0.173253 \| 0.212406 \| \| TC \| 0.940921 \| -0.124870 \| -0.164045 \| -0.252011 \| 0.093030 \| \| CH \| 0.694865 \| -0.394363 \| -0.034669 \| 0.600360 \| 0.002670 \| \| BW \| 0.816533 \| 0.545304 \| 0.153143 \| -0.073292 \| 0.084217 \| \| \| \| **Pooled Within Canonical Structure** \| \| \| \| \| \| \| --- \| --- \| --- \| --- \| --- \| --- \| \| **Variable** \| **Can1** \| **Can2** \| **Can3** \| **Can4** \| **Can5** \| \| WS \| 0.567635 \| 0.287582 \| -0.142994 \| -0.296972 \| -0.262094 \| \| SL \| 0.785310 \| 0.280034 \| -0.192161 \| 0.262978 \| -0.011917 \| \| BL \| 0.435541 \| 0.421928 \| -0.065554 \| -0.222907 \| 0.017023 \| \| BKL \| 0.066780 \| 0.224523 \| -0.163698 \| -0.205046 \| 0.184926 \| \| NL \| 0.201276 \| -0.204894 \| 0.204207 \| -0.340277 \| -0.270010 \| \| CL \| 0.248226 \| -0.251178 \| -0.382257 \| 0.290723 \| 0.127432 \| \| SC \| 0.016276 \| 0.422546 \| -0.543654 \| 0.216875 \| 0.032756 \| \| CC \| 0.577993 \| 0.222736 \| -0.053045 \| -0.261837 \| 0.500633 \| \| TC \| 0.389056 \| -0.065782 \| -0.095951 \| -0.250322 \| 0.144114 \| \| CH \| 0.195693 \| -0.141502 \| -0.013812 \| 0.406170 \| 0.002818 \| \| BW \| 0.464350 \| 0.395095 \| 0.123196 \| -0.100127 \| 0.179431 \| \| \| \|  \| \| \| \| \| \| **Discriminant Analysis Results** \| \| --- \| \| \| --- \| --- \| \| \| \| **The DISCRIM Procedure Canonical Discriminant Analysis** \| \| --- \| \| \| --- \| --- \| \| \| \| **Total-Sample Standardized Canonical Coefficients** \| \| \| \| \| \| \| --- \| --- \| --- \| --- \| --- \| --- \| \| **Variable** \| **Can1** \| **Can2** \| **Can3** \| **Can4** \| **Can5** \| \| WS \| 0.273973269 \| 0.135667102 \| -0.787719067 \| -0.544494395 \| -1.170480154 \| \| SL \| 1.436685918 \| -0.319597776 \| 0.070845145 \| 0.726305621 \| -0.344541239 \| \| BL \| -0.093371266 \| 0.637055192 \| 0.056574731 \| -0.398101316 \| 0.171546209 \| \| BKL \| -0.263749362 \| 0.105988854 \| -0.138596727 \| -0.486959329 \| 0.297694463 \| \| NL \| 0.143097224 \| -0.354988892 \| 0.371797629 \| -0.369672845 \| -0.373307616 \| \| CL \| -0.317686720 \| -0.498071196 \| -0.688546903 \| 0.439831448 \| 0.366984081 \| \| SC \| -0.388548054 \| 0.601071900 \| -0.834882796 \| 0.053588010 \| -0.220937403 \| \| CC \| 0.693210973 \| -0.330326856 \| -0.302068121 \| -0.601297736 \| 1.359606308 \| \| TC \| 0.266744940 \| -0.744146007 \| -0.403568072 \| -0.611458675 \| -0.114206935 \| \| CH \| 0.174996967 \| -0.000307684 \| 0.530979113 \| 0.353349761 \| -0.058299513 \| \| BW \| -0.761636539 \| 1.192865163 \| 1.513301437 \| 1.073746828 \| 0.143047311 \| \| \| --- \| --- \| --- \| --- \| --- \| --- \| --- \| --- \| --- \| --- \| --- \| --- \| --- \| --- \| --- \| --- \| --- \| --- \| --- \| --- \| --- \| --- \| --- \| --- \| --- \| --- \| --- \| --- \| --- \| --- \| --- \| --- \| --- \| --- \| --- \| --- \| --- \| --- \| --- \| --- \| --- \| --- \| --- \| --- \| --- \| --- \| --- \| --- \| --- \| --- \| --- \| --- \| --- \| --- \| --- \| --- \| --- \| --- \| --- \| --- \| --- \| --- \| --- \| --- \| --- \| --- \| --- \| --- \| --- \| --- \| --- \| --- \| --- \| --- \| --- \| --- \| --- \| --- \| --- \| \| \| **Pooled Within-Class Standardized Canonical Coefficients** \| \| \| \| \| \| \| --- \| --- \| --- \| --- \| --- \| --- \| \| **Variable** \| **Can1** \| **Can2** \| **Can3** \| **Can4** \| **Can5** \| \| WS \| 0.222572108 \| 0.110214084 \| -0.639932113 \| -0.442339742 \| -0.950881944 \| \| SL \| 1.042518108 \| -0.231913228 \| 0.051408137 \| 0.527037088 \| -0.250013226 \| \| BL \| -0.079129058 \| 0.539883195 \| 0.047945212 \| -0.337377692 \| 0.145379736 \| \| BKL \| -0.255985399 \| 0.102868872 \| -0.134516870 \| -0.472624759 \| 0.288931262 \| \| NL \| 0.134472179 \| -0.333592283 \| 0.349387890 \| -0.347391175 \| -0.350806864 \| \| CL \| -0.286048050 \| -0.448467894 \| -0.619973976 \| 0.396028289 \| 0.330435848 \| \| SC \| -0.336338182 \| 0.520304832 \| -0.722698155 \| 0.046387297 \| -0.191249663 \| \| CC \| 0.565064187 \| -0.269262726 \| -0.246227893 \| -0.490142006 \| 1.108269868 \| \| TC \| 0.242562905 \| -0.676684689 \| -0.366982195 \| -0.556026264 \| -0.103853388 \| \| CH \| 0.167710263 \| -0.000294873 \| 0.508869658 \| 0.338636620 \| -0.055871978 \| \| BW \| -0.641458219 \| 1.004643453 \| 1.274518218 \| 0.904320752 \| 0.120475934 \| \| \| \| **Raw Canonical Coefficients** \| \| \| \| \| \| \| --- \| --- \| --- \| --- \| --- \| --- \| \| **Variable** \| **Can1** \| **Can2** \| **Can3** \| **Can4** \| **Can5** \| \| WS \| 0.079578040 \| 0.039405750 \| -0.228800203 \| -0.158153374 \| -0.339976659 \| \| SL \| 1.339769958 \| -0.298038349 \| 0.066066073 \| 0.677310496 \| -0.321299176 \| \| BL \| -0.027269841 \| 0.186057176 \| 0.016523113 \| -0.116268743 \| 0.050101473 \| \| BKL \| -0.799607449 \| 0.321325811 \| -0.420182914 \| -1.476311847 \| 0.902518621 \| \| NL \| 0.109563933 \| -0.271801074 \| 0.284670864 \| -0.283044000 \| -0.285827002 \| \| CL \| -0.171859098 \| -0.269441752 \| -0.372483463 \| 0.237935775 \| 0.198527509 \| \| SC \| -0.451236320 \| 0.698048721 \| -0.969582620 \| 0.062233889 \| -0.256583399 \| \| CC \| 0.278770873 \| -0.132839077 \| -0.121474987 \| -0.241808485 \| 0.546757989 \| \| TC \| 0.168459862 \| -0.469957306 \| -0.254869021 \| -0.386160067 \| -0.072126146 \| \| CH \| 0.114916330 \| -0.000202049 \| 0.348681307 \| 0.232036352 \| -0.038283898 \| \| BW \| -2.370928112 \| 3.713316528 \| 4.710815112 \| 3.342508412 \| 0.445297559 \| \| \| \| **Class Means on Canonical Variables** \| \| \| \| \| \| \| --- \| --- \| --- \| --- \| --- \| --- \| \| **DIST** \| **Can1** \| **Can2** \| **Can3** \| **Can4** \| **Can5** \| \| 1 \| -0.409426988 \| -0.396343568 \| -0.361686068 \| -0.141024748 \| -0.673109244 \| \| 2 \| -1.117947758 \| -0.011460927 \| 1.012044091 \| 0.597834419 \| 0.058975330 \| \| 3 \| -0.772562866 \| 1.162682213 \| 0.471992964 \| -0.934116556 \| 0.137385689 \| \| 4 \| -0.641952266 \| -1.281614831 \| -0.894644305 \| -0.150269229 \| 0.335340464 \| \| 5 \| 0.735914179 \| 1.198690025 \| -0.958098596 \| 0.412144125 \| 0.063147941 \| \| 6 \| 2.216822794 \| -0.594013542 \| 0.783099630 \| -0.132868442 \| 0.034612112 \| \| \| \|  \| \| \| \| \| \| **Discriminant Analysis Results** \| \| --- \| \| \| --- \| --- \| \| \| \| **The DISCRIM Procedure** \| \| --- \| \| \| --- \| --- \| \| \| \| **Linear Discriminant Function for DIST** \| \| \| \| \| \| \| \| --- \| --- \| --- \| --- \| --- \| --- \| --- \| \| **Variable** \| **1** \| **2** \| **3** \| **4** \| **5** \| **6** \| \| Constant \| -412.29092 \| -372.35219 \| -412.57247 \| -409.61734 \| -430.20680 \| -442.10070 \| \| WS \| 8.46322 \| 7.74195 \| 8.15490 \| 8.19039 \| 8.41588 \| 8.16060 \| \| SL \| 5.02344 \| 4.31545 \| 3.32977 \| 4.61027 \| 6.18126 \| 8.45469 \| \| BL \| 3.55664 \| 3.62104 \| 4.00320 \| 3.44106 \| 3.78489 \| 3.50167 \| \| BKL \| 26.10347 \| 25.78640 \| 28.44683 \| 27.15267 \| 25.79861 \| 24.08566 \| \| NL \| 8.88595 \| 8.67639 \| 8.65256 \| 8.66375 \| 8.04111 \| 9.34871 \| \| CL \| -4.28027 \| -4.45276 \| -4.97626 \| -3.60526 \| -4.40694 \| -4.96233 \| \| SC \| 6.25710 \| 5.37167 \| 6.44359 \| 6.00148 \| 7.27747 \| 3.64301 \| \| CC \| 11.43736 \| 11.24345 \| 11.66268 \| 12.10849 \| 11.88600 \| 12.44165 \| \| TC \| 3.26202 \| 2.27355 \| 2.50350 \| 3.70556 \| 2.59066 \| 3.45137 \| \| CH \| 0.84008 \| 1.38099 \| 0.87367 \| 0.58696 \| 0.86359 \| 1.51589 \| \| BW \| -171.88744 \| -159.51137 \| -163.60001 \| -176.71594 \| -169.31286 \| -173.11282 \| \| \| --- \| --- \| --- \| --- \| --- \| --- \| --- \| --- \| --- \| --- \| --- \| --- \| --- \| --- \| --- \| --- \| --- \| --- \| --- \| --- \| --- \| --- \| --- \| --- \| --- \| --- \| --- \| --- \| --- \| --- \| --- \| --- \| --- \| --- \| --- \| --- \| --- \| --- \| --- \| --- \| --- \| --- \| --- \| --- \| --- \| --- \| --- \| --- \| --- \| --- \| --- \| --- \| --- \| --- \| --- \| --- \| --- \| --- \| --- \| --- \| --- \| --- \| --- \| --- \| --- \| --- \| --- \| --- \| --- \| --- \| --- \| --- \| --- \| --- \| --- \| --- \| --- \| --- \| --- \| --- \| --- \| --- \| --- \| --- \| --- \| --- \| --- \| --- \| --- \| --- \| --- \| --- \| --- \| --- \| --- \| --- \| --- \| --- \| --- \| \| \|  \| \| \| \| \| \| **Discriminant Analysis Results** \| \| --- \| \| \| --- \| --- \| \| \| \| **The DISCRIM Procedure Classification Results for Calibration Data: WORK.SORTTEMPTABLESORTED Resubstitution Results using Linear Discriminant Function** \| \| --- \| \| \| --- \| --- \| \| \| \| **Posterior Probability of Membership in DIST** \| \| \| \| \| \| \| \| \| \| \| --- \| --- \| --- \| --- \| --- \| --- \| --- \| --- \| --- \| --- \| \| **Obs** \| **From DIST** \| **Classified into DIST** \| \| **1** \| **2** \| **3** \| **4** \| **5** \| **6** \| \| 1 \| 1 \| 1 \|  \| 0.6847 \| 0.0158 \| 0.0443 \| 0.1719 \| 0.0825 \| 0.0008 \| \| 2 \| 1 \| 1 \|  \| 0.3813 \| 0.2446 \| 0.0052 \| 0.3678 \| 0.0007 \| 0.0004 \| \| 3 \| 1 \| 1 \|  \| 0.8043 \| 0.0613 \| 0.0296 \| 0.0976 \| 0.0065 \| 0.0006 \| \| 4 \| 1 \| 1 \|  \| 0.7039 \| 0.0911 \| 0.0595 \| 0.1439 \| 0.0015 \| 0.0001 \| \| 5 \| 1 \| 4 \| * \| 0.4367 \| 0.0114 \| 0.0188 \| 0.4573 \| 0.0692 \| 0.0066 \| \| 6 \| 1 \| 4 \| * \| 0.4138 \| 0.1484 \| 0.0015 \| 0.4326 \| 0.0003 \| 0.0034 \| \| 7 \| 1 \| 4 \| * \| 0.3545 \| 0.0065 \| 0.0242 \| 0.5315 \| 0.0804 \| 0.0030 \| \| 8 \| 1 \| 1 \|  \| 0.7507 \| 0.0200 \| 0.0046 \| 0.2220 \| 0.0023 \| 0.0004 \| \| 9 \| 1 \| 1 \|  \| 0.5973 \| 0.0146 \| 0.0290 \| 0.2750 \| 0.0834 \| 0.0007 \| \| 10 \| 1 \| 1 \|  \| 0.7337 \| 0.1410 \| 0.0376 \| 0.0828 \| 0.0047 \| 0.0002 \| \| 11 \| 1 \| 1 \|  \| 0.6229 \| 0.0159 \| 0.0389 \| 0.2733 \| 0.0488 \| 0.0002 \| \| 12 \| 1 \| 1 \|  \| 0.7785 \| 0.0113 \| 0.0026 \| 0.2030 \| 0.0036 \| 0.0011 \| \| 13 \| 1 \| 1 \|  \| 0.7262 \| 0.1081 \| 0.0558 \| 0.1045 \| 0.0052 \| 0.0002 \| \| 14 \| 1 \| 1 \|  \| 0.6908 \| 0.0942 \| 0.0652 \| 0.1465 \| 0.0028 \| 0.0004 \| \| 15 \| 1 \| 1 \|  \| 0.4759 \| 0.3842 \| 0.0673 \| 0.0703 \| 0.0015 \| 0.0008 \| \| 16 \| 1 \| 4 \| * \| 0.3666 \| 0.0043 \| 0.0049 \| 0.3705 \| 0.1612 \| 0.0924 \| \| 17 \| 1 \| 4 \| * \| 0.3317 \| 0.0040 \| 0.0119 \| 0.5018 \| 0.1361 \| 0.0144 \| \| 18 \| 1 \| 1 \|  \| 0.7794 \| 0.0901 \| 0.0275 \| 0.0950 \| 0.0074 \| 0.0005 \| \| 19 \| 1 \| 1 \|  \| 0.7444 \| 0.1386 \| 0.0450 \| 0.0594 \| 0.0112 \| 0.0013 \| \| 20 \| 1 \| 1 \|  \| 0.5214 \| 0.3533 \| 0.0497 \| 0.0702 \| 0.0051 \| 0.0002 \| \| 21 \| 1 \| 1 \|  \| 0.4524 \| 0.1887 \| 0.0014 \| 0.3459 \| 0.0006 \| 0.0110 \| \| 22 \| 1 \| 1 \|  \| 0.6118 \| 0.0256 \| 0.0581 \| 0.1825 \| 0.1185 \| 0.0035 \| \| 23 \| 1 \| 1 \|  \| 0.6721 \| 0.0224 \| 0.0547 \| 0.1377 \| 0.1120 \| 0.0012 \| \| 24 \| 1 \| 2 \| * \| 0.1830 \| 0.6399 \| 0.0265 \| 0.1202 \| 0.0279 \| 0.0026 \| \| 25 \| 1 \| 6 \| * \| 0.0309 \| 0.0041 \| 0.0025 \| 0.0104 \| 0.3229 \| 0.6292 \| \| 26 \| 1 \| 4 \| * \| 0.3467 \| 0.1023 \| 0.0122 \| 0.4650 \| 0.0621 \| 0.0117 \| \| 27 \| 1 \| 2 \| * \| 0.2872 \| 0.3527 \| 0.0669 \| 0.2715 \| 0.0216 \| 0.0001 \| \| 28 \| 1 \| 4 \| * \| 0.1180 \| 0.0023 \| 0.0050 \| 0.8502 \| 0.0202 \| 0.0043 \| \| 29 \| 1 \| 1 \|  \| 0.3628 \| 0.0004 \| 0.0112 \| 0.3300 \| 0.1516 \| 0.1440 \| \| 30 \| 1 \| 4 \| * \| 0.4065 \| 0.1268 \| 0.0058 \| 0.4198 \| 0.0301 \| 0.0110 \| \| 31 \| 1 \| 6 \| * \| 0.0368 \| 0.0042 \| 0.0023 \| 0.0143 \| 0.1537 \| 0.7886 \| \| 32 \| 1 \| 5 \| * \| 0.1637 \| 0.1007 \| 0.2861 \| 0.0064 \| 0.3625 \| 0.0805 \| \| 33 \| 1 \| 1 \|  \| 0.4530 \| 0.3279 \| 0.0040 \| 0.1435 \| 0.0642 \| 0.0074 \| \| 34 \| 1 \| 1 \|  \| 0.4957 \| 0.0097 \| 0.0937 \| 0.0611 \| 0.1579 \| 0.1820 \| \| 35 \| 1 \| 4 \| * \| 0.2250 \| 0.0157 \| 0.0075 \| 0.6950 \| 0.0206 \| 0.0362 \| \| 36 \| 1 \| 4 \| * \| 0.1400 \| 0.0020 \| 0.0037 \| 0.8118 \| 0.0390 \| 0.0035 \| \| 37 \| 1 \| 5 \| * \| 0.0728 \| 0.0080 \| 0.0311 \| 0.0288 \| 0.5501 \| 0.3092 \| \| 38 \| 1 \| 2 \| * \| 0.2281 \| 0.3577 \| 0.0815 \| 0.3231 \| 0.0096 \| 0.0000 \| \| 39 \| 1 \| 3 \| * \| 0.1268 \| 0.1915 \| 0.3056 \| 0.0201 \| 0.2731 \| 0.0829 \| \| 40 \| 1 \| 3 \| * \| 0.1551 \| 0.2840 \| 0.3868 \| 0.0761 \| 0.0976 \| 0.0004 \| \| 41 \| 1 \| 2 \| * \| 0.1453 \| 0.7792 \| 0.0153 \| 0.0161 \| 0.0262 \| 0.0178 \| \| 42 \| 1 \| 5 \| * \| 0.1035 \| 0.1375 \| 0.2182 \| 0.0132 \| 0.4053 \| 0.1224 \| \| 43 \| 1 \| 2 \| * \| 0.2077 \| 0.3196 \| 0.2816 \| 0.1273 \| 0.0628 \| 0.0010 \| \| 44 \| 1 \| 2 \| * \| 0.1789 \| 0.4819 \| 0.2118 \| 0.1104 \| 0.0169 \| 0.0001 \| \| 45 \| 1 \| 2 \| * \| 0.1302 \| 0.7899 \| 0.0191 \| 0.0076 \| 0.0412 \| 0.0122 \| \| 46 \| 1 \| 5 \| * \| 0.1802 \| 0.1541 \| 0.2129 \| 0.0251 \| 0.3353 \| 0.0924 \| \| 47 \| 1 \| 2 \| * \| 0.2355 \| 0.6690 \| 0.0211 \| 0.0310 \| 0.0255 \| 0.0179 \| \| 48 \| 1 \| 2 \| * \| 0.1877 \| 0.4507 \| 0.2237 \| 0.1044 \| 0.0330 \| 0.0005 \| \| 49 \| 1 \| 2 \| * \| 0.1177 \| 0.8412 \| 0.0096 \| 0.0166 \| 0.0088 \| 0.0061 \| \| 50 \| 1 \| 5 \| * \| 0.2218 \| 0.1238 \| 0.1726 \| 0.0543 \| 0.2631 \| 0.1643 \| \| 51 \| 2 \| 2 \|  \| 0.1500 \| 0.5466 \| 0.0051 \| 0.2960 \| 0.0020 \| 0.0002 \| \| 52 \| 2 \| 2 \|  \| 0.1793 \| 0.5451 \| 0.0665 \| 0.2090 \| 0.0001 \| 0.0000 \| \| 53 \| 2 \| 1 \| * \| 0.3488 \| 0.2586 \| 0.2545 \| 0.1033 \| 0.0265 \| 0.0083 \| \| 54 \| 2 \| 2 \|  \| 0.0000 \| 0.8989 \| 0.0000 \| 0.0000 \| 0.0000 \| 0.1011 \| \| 55 \| 2 \| 4 \| * \| 0.2540 \| 0.2773 \| 0.0045 \| 0.4528 \| 0.0084 \| 0.0030 \| \| 56 \| 2 \| 2 \|  \| 0.0147 \| 0.8772 \| 0.1047 \| 0.0008 \| 0.0012 \| 0.0014 \| \| 57 \| 2 \| 2 \|  \| 0.2674 \| 0.4550 \| 0.0569 \| 0.2198 \| 0.0008 \| 0.0001 \| \| 58 \| 2 \| 1 \| * \| 0.3754 \| 0.1731 \| 0.0739 \| 0.3748 \| 0.0021 \| 0.0007 \| \| 59 \| 2 \| 1 \| * \| 0.3996 \| 0.2148 \| 0.2617 \| 0.0873 \| 0.0346 \| 0.0020 \| \| 60 \| 2 \| 1 \| * \| 0.3534 \| 0.2172 \| 0.1087 \| 0.3176 \| 0.0027 \| 0.0004 \| \| 61 \| 2 \| 2 \|  \| 0.0429 \| 0.8176 \| 0.1084 \| 0.0079 \| 0.0190 \| 0.0043 \| \| 62 \| 2 \| 4 \| * \| 0.1799 \| 0.3951 \| 0.0039 \| 0.4149 \| 0.0055 \| 0.0007 \| \| 63 \| 2 \| 2 \|  \| 0.2666 \| 0.3724 \| 0.0878 \| 0.2722 \| 0.0008 \| 0.0001 \| \| 64 \| 2 \| 2 \|  \| 0.2884 \| 0.3692 \| 0.2625 \| 0.0714 \| 0.0065 \| 0.0020 \| \| 65 \| 2 \| 2 \|  \| 0.0067 \| 0.8394 \| 0.1521 \| 0.0011 \| 0.0005 \| 0.0003 \| \| 66 \| 2 \| 2 \|  \| 0.0508 \| 0.9377 \| 0.0011 \| 0.0102 \| 0.0002 \| 0.0000 \| \| 67 \| 2 \| 3 \| * \| 0.1715 \| 0.0693 \| 0.6305 \| 0.0536 \| 0.0644 \| 0.0109 \| \| 68 \| 2 \| 2 \|  \| 0.1468 \| 0.7519 \| 0.0090 \| 0.0513 \| 0.0148 \| 0.0262 \| \| 69 \| 2 \| 1 \| * \| 0.3966 \| 0.1238 \| 0.1878 \| 0.0782 \| 0.2090 \| 0.0046 \| \| 70 \| 2 \| 2 \|  \| 0.2209 \| 0.4167 \| 0.0345 \| 0.1813 \| 0.1450 \| 0.0018 \| \| 71 \| 2 \| 2 \|  \| 0.1309 \| 0.7742 \| 0.0085 \| 0.0483 \| 0.0133 \| 0.0248 \| \| 72 \| 2 \| 2 \|  \| 0.1284 \| 0.7760 \| 0.0035 \| 0.0131 \| 0.0746 \| 0.0044 \| \| 73 \| 2 \| 2 \|  \| 0.0908 \| 0.8463 \| 0.0086 \| 0.0266 \| 0.0109 \| 0.0169 \| \| 74 \| 2 \| 1 \| * \| 0.3675 \| 0.0870 \| 0.3047 \| 0.1556 \| 0.0527 \| 0.0325 \| \| 75 \| 2 \| 1 \| * \| 0.5656 \| 0.0963 \| 0.0599 \| 0.1204 \| 0.1455 \| 0.0122 \| \| 76 \| 2 \| 2 \|  \| 0.1205 \| 0.7952 \| 0.0032 \| 0.0126 \| 0.0632 \| 0.0054 \| \| 77 \| 2 \| 2 \|  \| 0.1145 \| 0.7122 \| 0.0391 \| 0.0939 \| 0.0396 \| 0.0006 \| \| 78 \| 2 \| 2 \|  \| 0.1018 \| 0.8531 \| 0.0027 \| 0.0143 \| 0.0259 \| 0.0022 \| \| 79 \| 2 \| 2 \|  \| 0.0207 \| 0.9555 \| 0.0030 \| 0.0135 \| 0.0014 \| 0.0060 \| \| 80 \| 2 \| 1 \| * \| 0.3423 \| 0.1691 \| 0.0259 \| 0.2722 \| 0.1884 \| 0.0022 \| \| 81 \| 2 \| 3 \| * \| 0.1162 \| 0.0225 \| 0.7203 \| 0.0497 \| 0.0861 \| 0.0052 \| \| 82 \| 2 \| 1 \| * \| 0.3546 \| 0.0378 \| 0.2453 \| 0.1167 \| 0.2414 \| 0.0042 \| \| 83 \| 2 \| 2 \|  \| 0.0953 \| 0.6862 \| 0.0293 \| 0.1581 \| 0.0237 \| 0.0073 \| \| 84 \| 2 \| 2 \|  \| 0.2991 \| 0.4453 \| 0.0174 \| 0.1555 \| 0.0467 \| 0.0360 \| \| 85 \| 2 \| 4 \| * \| 0.2386 \| 0.1195 \| 0.0360 \| 0.3071 \| 0.2967 \| 0.0021 \| \| 86 \| 2 \| 2 \|  \| 0.2599 \| 0.4045 \| 0.0083 \| 0.0331 \| 0.2812 \| 0.0130 \| \| 87 \| 2 \| 2 \|  \| 0.1911 \| 0.6586 \| 0.0328 \| 0.0761 \| 0.0318 \| 0.0096 \| \| 88 \| 2 \| 3 \| * \| 0.1317 \| 0.0855 \| 0.6891 \| 0.0306 \| 0.0557 \| 0.0074 \| \| 89 \| 2 \| 1 \| * \| 0.3267 \| 0.1893 \| 0.2341 \| 0.0506 \| 0.1958 \| 0.0036 \| \| 90 \| 2 \| 2 \|  \| 0.0532 \| 0.8711 \| 0.0066 \| 0.0484 \| 0.0063 \| 0.0144 \| \| 91 \| 2 \| 1 \| * \| 0.3613 \| 0.2898 \| 0.0139 \| 0.2392 \| 0.0496 \| 0.0462 \| \| 92 \| 2 \| 1 \| * \| 0.3620 \| 0.2533 \| 0.0104 \| 0.3267 \| 0.0221 \| 0.0256 \| \| 93 \| 2 \| 2 \|  \| 0.0429 \| 0.9085 \| 0.0069 \| 0.0267 \| 0.0056 \| 0.0094 \| \| 94 \| 2 \| 1 \| * \| 0.3655 \| 0.1875 \| 0.0213 \| 0.2667 \| 0.1001 \| 0.0590 \| \| 95 \| 2 \| 2 \|  \| 0.0074 \| 0.9737 \| 0.0171 \| 0.0008 \| 0.0007 \| 0.0003 \| \| 96 \| 2 \| 2 \|  \| 0.0938 \| 0.4356 \| 0.0442 \| 0.4221 \| 0.0023 \| 0.0020 \| \| 97 \| 2 \| 2 \|  \| 0.0063 \| 0.9733 \| 0.0191 \| 0.0004 \| 0.0007 \| 0.0001 \| \| 98 \| 2 \| 2 \|  \| 0.0011 \| 0.9253 \| 0.0733 \| 0.0001 \| 0.0003 \| 0.0000 \| \| 99 \| 2 \| 2 \|  \| 0.0118 \| 0.9433 \| 0.0379 \| 0.0066 \| 0.0001 \| 0.0002 \| \| 100 \| 2 \| 2 \|  \| 0.0146 \| 0.6507 \| 0.3209 \| 0.0004 \| 0.0132 \| 0.0002 \| \| 101 \| 2 \| 2 \|  \| 0.0006 \| 0.9602 \| 0.0390 \| 0.0000 \| 0.0001 \| 0.0000 \| \| 102 \| 2 \| 2 \|  \| 0.0042 \| 0.7394 \| 0.2554 \| 0.0007 \| 0.0002 \| 0.0000 \| \| 103 \| 2 \| 2 \|  \| 0.0326 \| 0.5206 \| 0.4158 \| 0.0009 \| 0.0298 \| 0.0002 \| \| 104 \| 2 \| 2 \|  \| 0.0256 \| 0.9260 \| 0.0410 \| 0.0069 \| 0.0004 \| 0.0000 \| \| 105 \| 2 \| 2 \|  \| 0.0930 \| 0.5591 \| 0.0569 \| 0.2868 \| 0.0025 \| 0.0016 \| \| 106 \| 2 \| 2 \|  \| 0.0087 \| 0.8469 \| 0.1417 \| 0.0027 \| 0.0000 \| 0.0000 \| \| 107 \| 2 \| 2 \|  \| 0.0092 \| 0.9488 \| 0.0382 \| 0.0035 \| 0.0001 \| 0.0001 \| \| 108 \| 2 \| 2 \|  \| 0.0201 \| 0.5729 \| 0.3867 \| 0.0005 \| 0.0197 \| 0.0002 \| \| 109 \| 2 \| 2 \|  \| 0.0005 \| 0.9558 \| 0.0436 \| 0.0000 \| 0.0001 \| 0.0000 \| \| 110 \| 2 \| 2 \|  \| 0.0033 \| 0.7400 \| 0.2562 \| 0.0004 \| 0.0002 \| 0.0000 \| \| 111 \| 2 \| 2 \|  \| 0.0049 \| 0.9108 \| 0.0812 \| 0.0031 \| 0.0000 \| 0.0000 \| \| 112 \| 2 \| 2 \|  \| 0.0235 \| 0.9212 \| 0.0506 \| 0.0041 \| 0.0005 \| 0.0000 \| \| 113 \| 2 \| 2 \|  \| 0.0090 \| 0.9485 \| 0.0386 \| 0.0037 \| 0.0001 \| 0.0001 \| \| 114 \| 3 \| 2 \| * \| 0.0515 \| 0.7349 \| 0.0225 \| 0.1741 \| 0.0034 \| 0.0136 \| \| 115 \| 3 \| 3 \|  \| 0.0378 \| 0.0151 \| 0.8782 \| 0.0477 \| 0.0211 \| 0.0000 \| \| 116 \| 3 \| 3 \|  \| 0.1376 \| 0.1611 \| 0.6392 \| 0.0196 \| 0.0328 \| 0.0096 \| \| 117 \| 3 \| 6 \| * \| 0.0528 \| 0.0064 \| 0.1801 \| 0.0393 \| 0.2108 \| 0.5106 \| \| 118 \| 3 \| 3 \|  \| 0.0020 \| 0.0016 \| 0.6633 \| 0.0000 \| 0.3329 \| 0.0001 \| \| 119 \| 3 \| 3 \|  \| 0.1342 \| 0.1698 \| 0.6080 \| 0.0109 \| 0.0564 \| 0.0207 \| \| 120 \| 3 \| 5 \| * \| 0.0267 \| 0.0350 \| 0.1259 \| 0.0006 \| 0.6471 \| 0.1648 \| \| 121 \| 3 \| 3 \|  \| 0.1236 \| 0.2706 \| 0.5892 \| 0.0124 \| 0.0040 \| 0.0001 \| \| 122 \| 3 \| 2 \| * \| 0.0454 \| 0.7242 \| 0.0221 \| 0.2047 \| 0.0013 \| 0.0022 \| \| 123 \| 3 \| 5 \| * \| 0.0255 \| 0.0448 \| 0.1531 \| 0.0004 \| 0.6334 \| 0.1428 \| \| 124 \| 3 \| 3 \|  \| 0.1661 \| 0.1846 \| 0.5963 \| 0.0285 \| 0.0202 \| 0.0045 \| \| 125 \| 3 \| 3 \|  \| 0.1074 \| 0.0154 \| 0.4708 \| 0.1085 \| 0.1542 \| 0.1437 \| \| 126 \| 3 \| 3 \|  \| 0.0026 \| 0.0018 \| 0.7269 \| 0.0000 \| 0.2686 \| 0.0001 \| \| 127 \| 3 \| 3 \|  \| 0.1283 \| 0.2020 \| 0.6333 \| 0.0163 \| 0.0174 \| 0.0028 \| \| 128 \| 3 \| 5 \| * \| 0.0542 \| 0.0372 \| 0.1338 \| 0.0018 \| 0.6411 \| 0.1318 \| \| 129 \| 3 \| 3 \|  \| 0.1352 \| 0.2739 \| 0.5700 \| 0.0183 \| 0.0026 \| 0.0000 \| \| 130 \| 3 \| 3 \|  \| 0.1187 \| 0.0108 \| 0.3760 \| 0.1700 \| 0.1517 \| 0.1729 \| \| 131 \| 3 \| 2 \| * \| 0.0467 \| 0.8121 \| 0.0273 \| 0.0957 \| 0.0040 \| 0.0143 \| \| 132 \| 3 \| 3 \|  \| 0.0388 \| 0.0167 \| 0.8784 \| 0.0279 \| 0.0381 \| 0.0001 \| \| 133 \| 3 \| 6 \| * \| 0.0182 \| 0.0021 \| 0.0431 \| 0.0071 \| 0.1611 \| 0.7684 \| \| 134 \| 3 \| 3 \|  \| 0.0016 \| 0.0014 \| 0.5228 \| 0.0000 \| 0.4739 \| 0.0002 \| \| 135 \| 3 \| 3 \|  \| 0.0474 \| 0.0180 \| 0.8496 \| 0.0716 \| 0.0134 \| 0.0000 \| \| 136 \| 3 \| 5 \| * \| 0.0160 \| 0.0396 \| 0.1041 \| 0.0001 \| 0.6046 \| 0.2355 \| \| 137 \| 3 \| 3 \|  \| 0.1308 \| 0.3205 \| 0.5339 \| 0.0076 \| 0.0070 \| 0.0002 \| \| 138 \| 3 \| 3 \|  \| 0.0338 \| 0.0234 \| 0.8937 \| 0.0381 \| 0.0109 \| 0.0000 \| \| 139 \| 3 \| 3 \|  \| 0.0933 \| 0.2999 \| 0.5969 \| 0.0064 \| 0.0034 \| 0.0001 \| \| 140 \| 3 \| 2 \| * \| 0.0339 \| 0.8236 \| 0.0234 \| 0.1167 \| 0.0011 \| 0.0014 \| \| 141 \| 3 \| 3 \|  \| 0.0884 \| 0.0077 \| 0.5953 \| 0.0010 \| 0.3075 \| 0.0001 \| \| 142 \| 3 \| 3 \|  \| 0.0170 \| 0.0561 \| 0.9003 \| 0.0139 \| 0.0127 \| 0.0000 \| \| 143 \| 3 \| 3 \|  \| 0.0468 \| 0.0279 \| 0.8436 \| 0.0013 \| 0.0804 \| 0.0001 \| \| 144 \| 3 \| 2 \| * \| 0.0330 \| 0.7528 \| 0.2079 \| 0.0059 \| 0.0005 \| 0.0000 \| \| 145 \| 3 \| 3 \|  \| 0.0624 \| 0.0202 \| 0.8163 \| 0.0017 \| 0.0993 \| 0.0001 \| \| 146 \| 3 \| 3 \|  \| 0.0031 \| 0.1268 \| 0.8666 \| 0.0021 \| 0.0015 \| 0.0000 \| \| 147 \| 3 \| 2 \| * \| 0.0083 \| 0.8841 \| 0.1050 \| 0.0026 \| 0.0001 \| 0.0000 \| \| 148 \| 3 \| 3 \|  \| 0.0277 \| 0.0419 \| 0.8866 \| 0.0258 \| 0.0179 \| 0.0000 \| \| 149 \| 3 \| 2 \| * \| 0.1030 \| 0.5728 \| 0.3056 \| 0.0173 \| 0.0012 \| 0.0000 \| \| 150 \| 3 \| 2 \| * \| 0.0474 \| 0.7090 \| 0.2378 \| 0.0054 \| 0.0004 \| 0.0001 \| \| 151 \| 3 \| 3 \|  \| 0.0457 \| 0.0449 \| 0.8317 \| 0.0275 \| 0.0498 \| 0.0004 \| \| 152 \| 3 \| 3 \|  \| 0.0352 \| 0.0140 \| 0.8070 \| 0.0009 \| 0.1425 \| 0.0004 \| \| 153 \| 3 \| 2 \| * \| 0.1399 \| 0.6117 \| 0.2306 \| 0.0148 \| 0.0021 \| 0.0009 \| \| 154 \| 3 \| 3 \|  \| 0.0450 \| 0.0143 \| 0.7942 \| 0.0015 \| 0.1441 \| 0.0008 \| \| 155 \| 3 \| 3 \|  \| 0.1560 \| 0.3899 \| 0.4404 \| 0.0114 \| 0.0021 \| 0.0002 \| \| 156 \| 3 \| 3 \|  \| 0.0406 \| 0.0364 \| 0.8553 \| 0.0187 \| 0.0487 \| 0.0003 \| \| 157 \| 4 \| 4 \|  \| 0.1361 \| 0.0422 \| 0.0030 \| 0.6710 \| 0.1307 \| 0.0170 \| \| 158 \| 4 \| 4 \|  \| 0.1771 \| 0.0600 \| 0.0346 \| 0.7130 \| 0.0147 \| 0.0006 \| \| 159 \| 4 \| 4 \|  \| 0.0763 \| 0.0084 \| 0.0127 \| 0.7374 \| 0.1642 \| 0.0010 \| \| 160 \| 4 \| 4 \|  \| 0.1383 \| 0.0145 \| 0.0234 \| 0.8060 \| 0.0172 \| 0.0005 \| \| 161 \| 4 \| 4 \|  \| 0.1262 \| 0.0317 \| 0.0084 \| 0.7043 \| 0.1262 \| 0.0032 \| \| 162 \| 4 \| 4 \|  \| 0.1670 \| 0.0437 \| 0.0389 \| 0.7331 \| 0.0167 \| 0.0006 \| \| 163 \| 4 \| 4 \|  \| 0.1867 \| 0.1067 \| 0.0278 \| 0.6690 \| 0.0091 \| 0.0007 \| \| 164 \| 4 \| 4 \|  \| 0.0943 \| 0.0102 \| 0.0081 \| 0.6012 \| 0.2847 \| 0.0015 \| \| 165 \| 4 \| 4 \|  \| 0.1674 \| 0.0658 \| 0.0321 \| 0.7230 \| 0.0111 \| 0.0005 \| \| 166 \| 4 \| 4 \|  \| 0.1040 \| 0.0205 \| 0.0068 \| 0.7237 \| 0.1413 \| 0.0038 \| \| 167 \| 4 \| 4 \|  \| 0.1094 \| 0.0507 \| 0.0018 \| 0.8347 \| 0.0018 \| 0.0017 \| \| 168 \| 4 \| 4 \|  \| 0.3409 \| 0.0610 \| 0.0026 \| 0.5877 \| 0.0038 \| 0.0039 \| \| 169 \| 4 \| 4 \|  \| 0.1723 \| 0.0623 \| 0.0026 \| 0.7570 \| 0.0031 \| 0.0026 \| \| 170 \| 4 \| 4 \|  \| 0.2083 \| 0.0140 \| 0.0018 \| 0.7683 \| 0.0049 \| 0.0027 \| \| 171 \| 4 \| 4 \|  \| 0.3083 \| 0.0704 \| 0.0023 \| 0.6120 \| 0.0031 \| 0.0038 \| \| 172 \| 4 \| 4 \|  \| 0.1188 \| 0.0190 \| 0.0014 \| 0.8541 \| 0.0064 \| 0.0002 \| \| 173 \| 4 \| 4 \|  \| 0.2575 \| 0.0558 \| 0.0019 \| 0.6786 \| 0.0026 \| 0.0036 \| \| 174 \| 4 \| 4 \|  \| 0.0637 \| 0.0338 \| 0.0011 \| 0.8996 \| 0.0009 \| 0.0009 \| \| 175 \| 4 \| 4 \|  \| 0.0909 \| 0.0063 \| 0.0006 \| 0.8991 \| 0.0017 \| 0.0013 \| \| 176 \| 4 \| 4 \|  \| 0.2112 \| 0.0622 \| 0.0018 \| 0.7201 \| 0.0020 \| 0.0027 \| \| 177 \| 4 \| 4 \|  \| 0.0674 \| 0.0126 \| 0.0009 \| 0.9156 \| 0.0034 \| 0.0001 \| \| 178 \| 4 \| 4 \|  \| 0.1638 \| 0.0101 \| 0.0012 \| 0.8186 \| 0.0036 \| 0.0028 \| \| 179 \| 4 \| 4 \|  \| 0.2431 \| 0.0098 \| 0.0013 \| 0.7278 \| 0.0083 \| 0.0097 \| \| 180 \| 4 \| 4 \|  \| 0.4270 \| 0.0855 \| 0.0032 \| 0.4736 \| 0.0053 \| 0.0053 \| \| 181 \| 4 \| 4 \|  \| 0.1943 \| 0.0240 \| 0.0023 \| 0.7679 \| 0.0112 \| 0.0003 \| \| 182 \| 4 \| 4 \|  \| 0.0892 \| 0.0463 \| 0.0015 \| 0.8599 \| 0.0014 \| 0.0016 \| \| 183 \| 4 \| 4 \|  \| 0.1577 \| 0.0232 \| 0.0015 \| 0.8083 \| 0.0090 \| 0.0003 \| \| 184 \| 4 \| 4 \|  \| 0.1382 \| 0.0533 \| 0.0020 \| 0.8015 \| 0.0026 \| 0.0025 \| \| 185 \| 4 \| 4 \|  \| 0.0949 \| 0.0161 \| 0.0011 \| 0.8828 \| 0.0049 \| 0.0002 \| \| 186 \| 4 \| 4 \|  \| 0.1192 \| 0.0057 \| 0.0007 \| 0.8696 \| 0.0023 \| 0.0025 \| \| 187 \| 4 \| 4 \|  \| 0.3264 \| 0.0055 \| 0.0027 \| 0.6635 \| 0.0019 \| 0.0002 \| \| 188 \| 4 \| 4 \|  \| 0.0593 \| 0.0018 \| 0.0021 \| 0.9005 \| 0.0276 \| 0.0086 \| \| 189 \| 4 \| 4 \|  \| 0.1522 \| 0.2172 \| 0.0276 \| 0.4600 \| 0.0460 \| 0.0971 \| \| 190 \| 4 \| 1 \| * \| 0.4189 \| 0.1200 \| 0.2385 \| 0.2129 \| 0.0097 \| 0.0001 \| \| 191 \| 4 \| 1 \| * \| 0.5145 \| 0.0503 \| 0.0046 \| 0.4228 \| 0.0030 \| 0.0049 \| \| 192 \| 4 \| 1 \| * \| 0.4120 \| 0.1499 \| 0.2775 \| 0.1492 \| 0.0113 \| 0.0002 \| \| 193 \| 4 \| 4 \|  \| 0.3740 \| 0.0082 \| 0.0036 \| 0.6101 \| 0.0037 \| 0.0003 \| \| 194 \| 4 \| 1 \| * \| 0.3637 \| 0.2364 \| 0.3128 \| 0.0647 \| 0.0217 \| 0.0007 \| \| 195 \| 4 \| 4 \|  \| 0.1237 \| 0.2437 \| 0.0273 \| 0.3191 \| 0.0666 \| 0.2196 \| \| 196 \| 4 \| 4 \|  \| 0.2921 \| 0.0072 \| 0.0039 \| 0.6959 \| 0.0008 \| 0.0000 \| \| 197 \| 4 \| 4 \|  \| 0.0610 \| 0.0028 \| 0.0029 \| 0.9061 \| 0.0235 \| 0.0038 \| \| 198 \| 4 \| 4 \|  \| 0.4392 \| 0.0370 \| 0.0032 \| 0.5180 \| 0.0013 \| 0.0013 \| \| 199 \| 4 \| 4 \|  \| 0.0750 \| 0.0115 \| 0.0102 \| 0.8771 \| 0.0250 \| 0.0011 \| \| 200 \| 4 \| 4 \|  \| 0.4662 \| 0.0128 \| 0.0049 \| 0.5094 \| 0.0053 \| 0.0014 \| \| 201 \| 4 \| 4 \|  \| 0.3643 \| 0.0173 \| 0.0029 \| 0.6141 \| 0.0012 \| 0.0002 \| \| 202 \| 4 \| 4 \|  \| 0.0915 \| 0.0082 \| 0.0076 \| 0.7043 \| 0.1434 \| 0.0450 \| \| 203 \| 4 \| 1 \| * \| 0.4064 \| 0.1794 \| 0.2938 \| 0.1048 \| 0.0152 \| 0.0004 \| \| 204 \| 4 \| 6 \| * \| 0.0651 \| 0.2220 \| 0.0196 \| 0.0946 \| 0.0944 \| 0.5042 \| \| 205 \| 4 \| 4 \|  \| 0.1252 \| 0.2676 \| 0.0396 \| 0.5102 \| 0.0306 \| 0.0269 \| \| 206 \| 4 \| 4 \|  \| 0.3538 \| 0.0253 \| 0.0034 \| 0.6164 \| 0.0009 \| 0.0002 \| \| 207 \| 4 \| 4 \|  \| 0.2462 \| 0.0030 \| 0.0019 \| 0.7480 \| 0.0009 \| 0.0001 \| \| 208 \| 4 \| 4 \|  \| 0.0807 \| 0.0057 \| 0.0058 \| 0.8091 \| 0.0827 \| 0.0160 \| \| 209 \| 4 \| 4 \|  \| 0.4582 \| 0.0355 \| 0.0040 \| 0.4985 \| 0.0023 \| 0.0015 \| \| 210 \| 4 \| 1 \| * \| 0.3878 \| 0.1813 \| 0.2612 \| 0.1603 \| 0.0092 \| 0.0001 \| \| 211 \| 4 \| 6 \| * \| 0.0978 \| 0.2498 \| 0.0261 \| 0.1958 \| 0.0874 \| 0.3431 \| \| 212 \| 5 \| 5 \|  \| 0.0580 \| 0.0010 \| 0.0035 \| 0.0011 \| 0.8879 \| 0.0485 \| \| 213 \| 5 \| 5 \|  \| 0.1834 \| 0.2407 \| 0.0582 \| 0.0475 \| 0.4660 \| 0.0042 \| \| 214 \| 5 \| 5 \|  \| 0.0547 \| 0.0040 \| 0.0194 \| 0.0172 \| 0.8758 \| 0.0289 \| \| 215 \| 5 \| 5 \|  \| 0.1818 \| 0.3291 \| 0.0598 \| 0.0472 \| 0.3807 \| 0.0014 \| \| 216 \| 5 \| 5 \|  \| 0.0344 \| 0.0005 \| 0.0022 \| 0.0007 \| 0.8137 \| 0.1485 \| \| 217 \| 5 \| 5 \|  \| 0.0544 \| 0.0020 \| 0.0207 \| 0.0256 \| 0.8636 \| 0.0337 \| \| 218 \| 5 \| 5 \|  \| 0.0291 \| 0.0003 \| 0.0020 \| 0.0007 \| 0.8387 \| 0.1291 \| \| 219 \| 5 \| 5 \|  \| 0.1486 \| 0.0888 \| 0.0559 \| 0.0518 \| 0.6522 \| 0.0027 \| \| 220 \| 5 \| 5 \|  \| 0.0648 \| 0.0043 \| 0.0128 \| 0.0219 \| 0.8491 \| 0.0471 \| \| 221 \| 5 \| 5 \|  \| 0.0423 \| 0.0005 \| 0.0020 \| 0.0010 \| 0.7745 \| 0.1797 \| \| 222 \| 5 \| 5 \|  \| 0.0853 \| 0.0043 \| 0.0131 \| 0.0360 \| 0.8161 \| 0.0451 \| \| 223 \| 5 \| 5 \|  \| 0.0283 \| 0.0003 \| 0.0033 \| 0.0007 \| 0.8430 \| 0.1245 \| \| 224 \| 5 \| 5 \|  \| 0.2267 \| 0.2561 \| 0.0556 \| 0.0763 \| 0.3810 \| 0.0043 \| \| 225 \| 5 \| 5 \|  \| 0.1051 \| 0.0081 \| 0.0172 \| 0.0345 \| 0.8058 \| 0.0293 \| \| 226 \| 5 \| 5 \|  \| 0.1965 \| 0.1792 \| 0.0603 \| 0.0590 \| 0.5007 \| 0.0043 \| \| 227 \| 5 \| 3 \| * \| 0.1878 \| 0.0140 \| 0.4593 \| 0.0280 \| 0.3068 \| 0.0041 \| \| 228 \| 5 \| 5 \|  \| 0.0295 \| 0.0330 \| 0.0056 \| 0.0078 \| 0.9180 \| 0.0062 \| \| 229 \| 5 \| 3 \| * \| 0.1459 \| 0.0110 \| 0.4704 \| 0.0204 \| 0.3473 \| 0.0049 \| \| 230 \| 5 \| 3 \| * \| 0.2050 \| 0.0185 \| 0.4409 \| 0.0293 \| 0.2989 \| 0.0072 \| \| 231 \| 5 \| 5 \|  \| 0.0754 \| 0.0357 \| 0.0180 \| 0.2341 \| 0.6174 \| 0.0194 \| \| 232 \| 5 \| 5 \|  \| 0.0107 \| 0.0008 \| 0.0063 \| 0.0038 \| 0.9135 \| 0.0647 \| \| 233 \| 5 \| 5 \|  \| 0.0269 \| 0.0014 \| 0.0064 \| 0.0101 \| 0.8254 \| 0.1298 \| \| 234 \| 5 \| 5 \|  \| 0.0168 \| 0.0050 \| 0.0032 \| 0.0058 \| 0.9617 \| 0.0076 \| \| 235 \| 5 \| 5 \|  \| 0.0296 \| 0.0157 \| 0.0038 \| 0.0092 \| 0.9323 \| 0.0095 \| \| 236 \| 5 \| 5 \|  \| 0.0966 \| 0.0445 \| 0.0182 \| 0.3266 \| 0.4907 \| 0.0234 \| \| 237 \| 5 \| 3 \| * \| 0.0996 \| 0.0428 \| 0.4460 \| 0.0239 \| 0.3877 \| 0.0001 \| \| 238 \| 5 \| 5 \|  \| 0.0183 \| 0.0009 \| 0.0055 \| 0.0053 \| 0.8993 \| 0.0708 \| \| 239 \| 5 \| 3 \| * \| 0.1447 \| 0.0078 \| 0.4162 \| 0.0202 \| 0.4059 \| 0.0052 \| \| 240 \| 5 \| 5 \|  \| 0.3063 \| 0.0359 \| 0.2486 \| 0.0323 \| 0.3535 \| 0.0233 \| \| 241 \| 5 \| 3 \| * \| 0.1195 \| 0.0803 \| 0.5119 \| 0.0195 \| 0.2688 \| 0.0000 \| \| 242 \| 5 \| 5 \|  \| 0.0581 \| 0.0093 \| 0.0131 \| 0.2175 \| 0.6827 \| 0.0194 \| \| 243 \| 5 \| 5 \|  \| 0.0379 \| 0.0032 \| 0.0065 \| 0.0111 \| 0.7213 \| 0.2201 \| \| 244 \| 5 \| 5 \|  \| 0.0099 \| 0.0003 \| 0.0028 \| 0.0026 \| 0.8691 \| 0.1153 \| \| 245 \| 5 \| 3 \| * \| 0.1858 \| 0.1356 \| 0.4418 \| 0.0541 \| 0.1826 \| 0.0000 \| \| 246 \| 5 \| 5 \|  \| 0.0214 \| 0.0118 \| 0.0042 \| 0.0064 \| 0.9492 \| 0.0069 \| \| 247 \| 5 \| 5 \|  \| 0.1063 \| 0.0776 \| 0.0187 \| 0.3063 \| 0.4637 \| 0.0275 \| \| 248 \| 5 \| 5 \|  \| 0.1256 \| 0.0319 \| 0.3367 \| 0.0264 \| 0.4792 \| 0.0001 \| \| 249 \| 5 \| 3 \| * \| 0.1570 \| 0.1224 \| 0.4599 \| 0.0407 \| 0.2199 \| 0.0000 \| \| 250 \| 5 \| 5 \|  \| 0.0643 \| 0.0250 \| 0.0172 \| 0.2395 \| 0.6382 \| 0.0157 \| \| 251 \| 5 \| 5 \|  \| 0.0453 \| 0.0489 \| 0.0076 \| 0.0125 \| 0.8765 \| 0.0092 \| \| 252 \| 5 \| 5 \|  \| 0.0013 \| 0.0021 \| 0.4263 \| 0.0000 \| 0.5677 \| 0.0025 \| \| 253 \| 5 \| 5 \|  \| 0.1206 \| 0.1042 \| 0.0265 \| 0.0239 \| 0.7193 \| 0.0055 \| \| 254 \| 5 \| 5 \|  \| 0.0173 \| 0.0094 \| 0.0462 \| 0.0009 \| 0.9041 \| 0.0221 \| \| 255 \| 5 \| 5 \|  \| 0.0857 \| 0.0014 \| 0.0003 \| 0.0800 \| 0.8304 \| 0.0022 \| \| 256 \| 5 \| 5 \|  \| 0.0226 \| 0.0161 \| 0.0554 \| 0.0011 \| 0.8879 \| 0.0169 \| \| 257 \| 5 \| 5 \|  \| 0.0763 \| 0.0247 \| 0.0204 \| 0.0177 \| 0.8594 \| 0.0015 \| \| 258 \| 5 \| 3 \| * \| 0.0010 \| 0.0058 \| 0.7109 \| 0.0000 \| 0.2809 \| 0.0013 \| \| 259 \| 5 \| 5 \|  \| 0.0104 \| 0.0030 \| 0.0195 \| 0.0005 \| 0.9561 \| 0.0105 \| \| 260 \| 5 \| 5 \|  \| 0.0797 \| 0.0013 \| 0.0003 \| 0.0625 \| 0.8546 \| 0.0016 \| \| 261 \| 5 \| 5 \|  \| 0.0007 \| 0.0006 \| 0.2521 \| 0.0000 \| 0.7437 \| 0.0029 \| \| 262 \| 5 \| 5 \|  \| 0.0098 \| 0.0021 \| 0.0161 \| 0.0005 \| 0.9576 \| 0.0139 \| \| 263 \| 5 \| 5 \|  \| 0.0534 \| 0.0006 \| 0.0002 \| 0.0461 \| 0.8972 \| 0.0025 \| \| 264 \| 5 \| 5 \|  \| 0.1045 \| 0.0023 \| 0.0003 \| 0.0934 \| 0.7963 \| 0.0032 \| \| 265 \| 5 \| 3 \| * \| 0.0012 \| 0.0033 \| 0.5666 \| 0.0000 \| 0.4276 \| 0.0012 \| \| 266 \| 5 \| 5 \|  \| 0.0345 \| 0.0094 \| 0.0109 \| 0.0066 \| 0.9365 \| 0.0022 \| \| 267 \| 5 \| 5 \|  \| 0.0063 \| 0.0014 \| 0.0112 \| 0.0003 \| 0.9625 \| 0.0183 \| \| 268 \| 5 \| 5 \|  \| 0.0452 \| 0.0140 \| 0.0131 \| 0.0076 \| 0.9188 \| 0.0013 \| \| 269 \| 5 \| 5 \|  \| 0.0010 \| 0.0012 \| 0.3985 \| 0.0000 \| 0.5983 \| 0.0010 \| \| 270 \| 5 \| 5 \|  \| 0.1172 \| 0.0042 \| 0.0003 \| 0.0928 \| 0.7827 \| 0.0029 \| \| 271 \| 5 \| 5 \|  \| 0.1111 \| 0.0617 \| 0.0251 \| 0.0226 \| 0.7756 \| 0.0038 \| \| 272 \| 6 \| 5 \| * \| 0.0137 \| 0.0020 \| 0.0061 \| 0.0001 \| 0.5716 \| 0.4065 \| \| 273 \| 6 \| 6 \|  \| 0.0931 \| 0.0037 \| 0.0002 \| 0.0433 \| 0.1177 \| 0.7421 \| \| 274 \| 6 \| 5 \| * \| 0.0100 \| 0.0016 \| 0.0052 \| 0.0001 \| 0.5662 \| 0.4170 \| \| 275 \| 6 \| 5 \| * \| 0.0258 \| 0.0021 \| 0.0010 \| 0.0073 \| 0.8838 \| 0.0801 \| \| 276 \| 6 \| 5 \| * \| 0.0839 \| 0.0765 \| 0.0194 \| 0.0868 \| 0.4056 \| 0.3279 \| \| 277 \| 6 \| 5 \| * \| 0.0580 \| 0.0941 \| 0.0249 \| 0.0493 \| 0.5208 \| 0.2528 \| \| 278 \| 6 \| 5 \| * \| 0.0307 \| 0.0072 \| 0.0024 \| 0.0063 \| 0.9175 \| 0.0359 \| \| 279 \| 6 \| 6 \|  \| 0.0311 \| 0.0009 \| 0.0001 \| 0.0154 \| 0.0767 \| 0.8759 \| \| 280 \| 6 \| 6 \|  \| 0.0127 \| 0.0034 \| 0.0232 \| 0.0206 \| 0.3496 \| 0.5905 \| \| 281 \| 6 \| 5 \| * \| 0.0142 \| 0.0030 \| 0.0063 \| 0.0001 \| 0.5254 \| 0.4510 \| \| 282 \| 6 \| 6 \|  \| 0.0754 \| 0.0018 \| 0.0001 \| 0.0335 \| 0.1134 \| 0.7758 \| \| 283 \| 6 \| 5 \| * \| 0.0175 \| 0.0014 \| 0.0006 \| 0.0046 \| 0.8555 \| 0.1203 \| \| 284 \| 6 \| 6 \|  \| 0.0084 \| 0.0023 \| 0.0179 \| 0.0142 \| 0.3627 \| 0.5945 \| \| 285 \| 6 \| 5 \| * \| 0.0088 \| 0.0007 \| 0.0072 \| 0.0001 \| 0.7407 \| 0.2425 \| \| 286 \| 6 \| 6 \|  \| 0.0178 \| 0.0100 \| 0.0323 \| 0.0237 \| 0.2726 \| 0.6436 \| \| 287 \| 6 \| 5 \| * \| 0.0502 \| 0.0664 \| 0.0275 \| 0.0450 \| 0.5523 \| 0.2585 \| \| 288 \| 6 \| 5 \| * \| 0.0134 \| 0.0008 \| 0.0011 \| 0.0039 \| 0.9357 \| 0.0452 \| \| 289 \| 6 \| 5 \| * \| 0.0867 \| 0.2289 \| 0.0199 \| 0.0684 \| 0.3315 \| 0.2646 \| \| 290 \| 6 \| 5 \| * \| 0.0422 \| 0.0471 \| 0.0474 \| 0.0400 \| 0.6692 \| 0.1542 \| \| 291 \| 6 \| 5 \| * \| 0.0505 \| 0.0083 \| 0.0020 \| 0.0107 \| 0.8750 \| 0.0535 \| \| 292 \| 6 \| 6 \|  \| 0.0100 \| 0.0019 \| 0.0304 \| 0.0184 \| 0.4566 \| 0.4827 \| \| 293 \| 6 \| 5 \| * \| 0.0230 \| 0.0051 \| 0.0070 \| 0.0001 \| 0.5552 \| 0.4096 \| \| 294 \| 6 \| 6 \|  \| 0.0151 \| 0.0081 \| 0.0401 \| 0.0214 \| 0.3619 \| 0.5534 \| \| 295 \| 6 \| 6 \|  \| 0.0518 \| 0.0018 \| 0.0001 \| 0.0230 \| 0.1035 \| 0.8198 \| \| 296 \| 6 \| 6 \|  \| 0.1306 \| 0.0060 \| 0.0002 \| 0.0594 \| 0.1190 \| 0.6848 \| \| 297 \| 6 \| 6 \|  \| 0.0080 \| 0.0002 \| 0.0001 \| 0.0003 \| 0.0001 \| 0.9912 \| \| 298 \| 6 \| 6 \|  \| 0.1321 \| 0.2359 \| 0.0134 \| 0.2037 \| 0.1087 \| 0.3062 \| \| 299 \| 6 \| 6 \|  \| 0.0067 \| 0.0003 \| 0.0001 \| 0.0002 \| 0.0001 \| 0.9926 \| \| 300 \| 6 \| 6 \|  \| 0.1113 \| 0.1535 \| 0.0110 \| 0.1676 \| 0.1066 \| 0.4498 \| \| 301 \| 6 \| 2 \| * \| 0.1142 \| 0.3341 \| 0.0144 \| 0.2123 \| 0.0814 \| 0.2436 \| \| 302 \| 6 \| 6 \|  \| 0.0086 \| 0.0001 \| 0.0003 \| 0.0004 \| 0.0002 \| 0.9904 \| \| 303 \| 6 \| 6 \|  \| 0.0079 \| 0.0003 \| 0.0001 \| 0.0003 \| 0.0001 \| 0.9914 \| \| 304 \| 6 \| 6 \|  \| 0.1054 \| 0.0884 \| 0.0137 \| 0.1759 \| 0.1724 \| 0.4442 \| \| 305 \| 6 \| 6 \|  \| 0.0274 \| 0.0015 \| 0.0006 \| 0.0010 \| 0.0002 \| 0.9692 \| \| 306 \| 6 \| 6 \|  \| 0.0056 \| 0.0002 \| 0.0001 \| 0.0002 \| 0.0001 \| 0.9939 \| \| 307 \| 6 \| 6 \|  \| 0.1074 \| 0.1359 \| 0.0127 \| 0.1753 \| 0.1116 \| 0.4571 \| \| 308 \| 6 \| 6 \|  \| 0.0295 \| 0.0017 \| 0.0007 \| 0.0011 \| 0.0002 \| 0.9668 \| \| 309 \| 6 \| 6 \|  \| 0.1004 \| 0.1161 \| 0.0061 \| 0.1336 \| 0.1394 \| 0.5044 \| \| 310 \| 6 \| 6 \|  \| 0.0047 \| 0.0001 \| 0.0002 \| 0.0002 \| 0.0001 \| 0.9947 \| \| 311 \| 6 \| 6 \|  \| 0.0000 \| 0.0000 \| 0.0000 \| 0.0000 \| 0.0000 \| 0.9999 \| \| 312 \| 6 \| 6 \|  \| 0.0000 \| 0.0000 \| 0.0000 \| 0.0000 \| 0.0000 \| 0.9999 \| \| 313 \| 6 \| 6 \|  \| 0.0000 \| 0.0000 \| 0.0000 \| 0.0000 \| 0.0000 \| 0.9999 \| \| 314 \| 6 \| 6 \|  \| 0.0001 \| 0.0001 \| 0.0000 \| 0.0000 \| 0.0000 \| 0.9998 \| \| 315 \| 6 \| 6 \|  \| 0.0000 \| 0.0001 \| 0.0001 \| 0.0000 \| 0.0000 \| 0.9998 \| \| 316 \| 6 \| 6 \|  \| 0.0000 \| 0.0000 \| 0.0000 \| 0.0000 \| 0.0000 \| 1.0000 \| \| 317 \| 6 \| 6 \|  \| 0.0001 \| 0.0000 \| 0.0000 \| 0.0001 \| 0.0000 \| 0.9999 \| \| 318 \| 6 \| 6 \|  \| 0.0000 \| 0.0000 \| 0.0000 \| 0.0000 \| 0.0000 \| 0.9999 \| \| 319 \| 6 \| 6 \|  \| 0.0001 \| 0.0002 \| 0.0000 \| 0.0000 \| 0.0000 \| 0.9996 \| \| 320 \| 6 \| 6 \|  \| 0.0000 \| 0.0001 \| 0.0001 \| 0.0000 \| 0.0000 \| 0.9998 \| \| 321 \| 6 \| 6 \|  \| 0.0001 \| 0.0001 \| 0.0000 \| 0.0000 \| 0.0000 \| 0.9998 \| \| 322 \| 6 \| 6 \|  \| 0.0050 \| 0.0002 \| 0.0001 \| 0.0060 \| 0.0000 \| 0.9887 \| \| 323 \| 6 \| 6 \|  \| 0.0018 \| 0.0017 \| 0.0005 \| 0.0010 \| 0.0001 \| 0.9950 \| \| \| --- \| --- \| --- \| --- \| --- \| --- \| --- \| --- \| --- \| --- \| --- \| --- \| --- \| --- \| --- \| --- \| --- \| --- \| --- \| --- \| --- \| --- \| --- \| --- \| --- \| --- \| --- \| --- \| --- \| --- \| --- \| --- \| --- \| --- \| --- \| --- \| --- \| --- \| --- \| --- \| --- \| --- \| --- \| --- \| --- \| --- \| --- \| --- \| --- \| --- \| --- \| --- \| --- \| --- \| --- \| --- \| --- \| --- \| --- \| --- \| --- \| --- \| --- \| --- \| --- \| --- \| --- \| --- \| --- \| --- \| --- \| --- \| --- \| --- \| --- \| --- \| --- \| --- \| --- \| --- \| --- \| --- \| --- \| --- \| --- \| --- \| --- \| --- \| --- \| --- \| --- \| --- \| --- \| --- \| --- \| --- \| --- \| --- \| --- \| --- \| --- \| --- \| --- \| --- \| --- \| --- \| --- \| --- \| --- \| --- \| --- \| --- \| --- \| --- \| --- \| --- \| --- \| --- \| --- \| --- \| --- \| --- \| --- \| --- \| --- \| --- \| --- \| --- \| --- \| --- \| --- \| --- \| --- \| --- \| --- \| --- \| --- \| --- \| --- \| --- \| --- \| --- \| --- \| --- \| --- \| --- \| --- \| --- \| --- \| --- \| --- \| --- \| --- \| --- \| --- \| --- \| --- \| --- \| --- \| --- \| --- \| --- \| --- \| --- \| --- \| --- \| --- \| --- \| --- \| --- \| --- \| --- \| --- \| --- \| --- \| --- \| --- \| --- \| --- \| --- \| --- \| --- \| --- \| --- \| --- \| --- \| --- \| --- \| --- \| --- \| --- \| --- \| --- \| --- \| --- \| --- \| --- \| --- \| --- \| --- \| --- \| --- \| --- \| --- \| --- \| --- \| --- \| --- \| --- \| --- \| --- \| --- \| --- \| --- \| --- \| --- \| --- \| --- \| --- \| --- \| --- \| --- \| --- \| --- \| --- \| --- \| --- \| --- \| --- \| --- \| --- \| --- \| --- \| --- \| --- \| --- \| --- \| --- \| --- \| --- \| --- \| --- \| --- \| --- \| --- \| --- \| --- \| --- \| --- \| --- \| --- \| --- \| --- \| --- \| --- \| --- \| --- \| --- \| --- \| --- \| --- \| --- \| --- \| --- \| --- \| --- \| --- \| --- \| --- \| --- \| --- \| --- \| --- \| --- \| --- \| --- \| --- \| --- \| --- \| --- \| --- \| --- \| --- \| --- \| --- \| --- \| --- \| --- \| --- \| --- \| --- \| --- \| --- \| --- \| --- \| --- \| --- \| --- \| --- \| --- \| --- \| --- \| --- \| --- \| --- \| --- \| --- \| --- \| --- \| --- \| --- \| --- \| --- \| --- \| --- \| --- \| --- \| --- \| --- \| --- \| --- \| --- \| --- \| --- \| --- \| --- \| --- \| --- \| --- \| --- \| --- \| --- \| --- \| --- \| --- \| --- \| --- \| --- \| --- \| --- \| --- \| --- \| --- \| --- \| --- \| --- \| --- \| --- \| --- \| --- \| --- \| --- \| --- \| --- \| --- \| --- \| --- \| --- \| --- \| --- \| --- \| --- \| --- \| --- \| --- \| --- \| --- \| --- \| --- \| --- \| --- \| --- \| --- \| --- \| --- \| --- \| --- \| --- \| --- \| --- \| --- \| --- \| --- \| --- \| --- \| --- \| --- \| --- \| --- \| --- \| --- \| --- \| --- \| --- \| --- \| --- \| --- \| --- \| --- \| --- \| --- \| --- \| --- \| --- \| --- \| --- \| --- \| --- \| --- \| --- \| --- \| --- \| --- \| --- \| --- \| --- \| --- \| --- \| --- \| --- \| --- \| --- \| --- \| --- \| --- \| --- \| --- \| --- \| --- \| --- \| --- \| --- \| --- \| --- \| --- \| --- \| --- \| --- \| --- \| --- \| --- \| --- \| --- \| --- \| --- \| --- \| --- \| --- \| --- \| --- \| --- \| --- \| --- \| --- \| --- \| --- \| --- \| --- \| --- \| --- \| --- \| --- \| --- \| --- \| --- \| --- \| --- \| --- \| --- \| --- \| --- \| --- \| --- \| --- \| --- \| --- \| --- \| --- \| --- \| --- \| --- \| --- \| --- \| --- \| --- \| --- \| --- \| --- \| --- \| --- \| --- \| --- \| --- \| --- \| --- \| --- \| --- \| --- \| --- \| --- \| --- \| --- \| --- \| --- \| --- \| --- \| --- \| --- \| --- \| --- \| --- \| --- \| --- \| --- \| --- \| --- \| --- \| --- \| --- \| --- \| --- \| --- \| --- \| --- \| --- \| --- \| --- \| --- \| --- \| --- \| --- \| --- \| --- \| --- \| --- \| --- \| --- \| --- \| --- \| --- \| --- \| --- \| --- \| --- \| --- \| --- \| --- \| --- \| --- \| --- \| --- \| --- \| --- \| --- \| --- \| --- \| --- \| --- \| --- \| --- \| --- \| --- \| --- \| --- \| --- \| --- \| --- \| --- \| --- \| --- \| --- \| --- \| --- \| --- \| --- \| --- \| --- \| --- \| --- \| --- \| --- \| --- \| --- \| --- \| --- \| --- \| --- \| --- \| --- \| --- \| --- \| --- \| --- \| --- \| --- \| --- \| --- \| --- \| --- \| --- \| --- \| --- \| --- \| --- \| --- \| --- \| --- \| --- \| --- \| --- \| --- \| --- \| --- \| --- \| --- \| --- \| --- \| --- \| --- \| --- \| --- \| --- \| --- \| --- \| --- \| --- \| --- \| --- \| --- \| --- \| --- \| --- \| --- \| --- \| --- \| --- \| --- \| --- \| --- \| --- \| --- \| --- \| --- \| --- \| --- \| --- \| --- \| --- \| --- \| --- \| --- \| --- \| --- \| --- \| --- \| --- \| --- \| --- \| --- \| --- \| --- \| --- \| --- \| --- \| --- \| --- \| --- \| --- \| --- \| --- \| --- \| --- \| --- \| --- \| --- \| --- \| --- \| --- \| --- \| --- \| --- \| --- \| --- \| --- \| --- \| --- \| --- \| --- \| --- \| --- \| --- \| --- \| --- \| --- \| --- \| --- \| --- \| --- \| --- \| --- \| --- \| --- \| --- \| --- \| --- \| --- \| --- \| --- \| --- \| --- \| --- \| --- \| --- \| --- \| --- \| --- \| --- \| --- \| --- \| --- \| --- \| --- \| --- \| --- \| --- \| --- \| --- \| --- \| --- \| --- \| --- \| --- \| --- \| --- \| --- \| --- \| --- \| --- \| --- \| --- \| --- \| --- \| --- \| --- \| --- \| --- \| --- \| --- \| --- \| --- \| --- \| --- \| --- \| --- \| --- \| --- \| --- \| --- \| --- \| --- \| --- \| --- \| --- \| --- \| --- \| --- \| --- \| --- \| --- \| --- \| --- \| --- \| --- \| --- \| --- \| --- \| --- \| --- \| --- \| --- \| --- \| --- \| --- \| --- \| --- \| --- \| --- \| --- \| --- \| --- \| --- \| --- \| --- \| --- \| --- \| --- \| --- \| --- \| --- \| --- \| --- \| --- \| --- \| --- \| --- \| --- \| --- \| --- \| --- \| --- \| --- \| --- \| --- \| --- \| --- \| --- \| --- \| --- \| --- \| --- \| --- \| --- \| --- \| --- \| --- \| --- \| --- \| --- \| --- \| --- \| --- \| --- \| --- \| --- \| --- \| --- \| --- \| --- \| --- \| --- \| --- \| --- \| --- \| --- \| --- \| --- \| --- \| --- \| --- \| --- \| --- \| --- \| --- \| --- \| --- \| --- \| --- \| --- \| --- \| --- \| --- \| --- \| --- \| --- \| --- \| --- \| --- \| --- \| --- \| --- \| --- \| --- \| --- \| --- \| --- \| --- \| --- \| --- \| --- \| --- \| --- \| --- \| --- \| --- \| --- \| --- \| --- \| --- \| --- \| --- \| --- \| --- \| --- \| --- \| --- \| --- \| --- \| --- \| --- \| --- \| --- \| --- \| --- \| --- \| --- \| --- \| --- \| --- \| --- \| --- \| --- \| --- \| --- \| --- \| --- \| --- \| --- \| --- \| --- \| --- \| --- \| --- \| --- \| --- \| --- \| --- \| --- \| --- \| --- \| --- \| --- \| --- \| --- \| --- \| --- \| --- \| --- \| --- \| --- \| --- \| --- \| --- \| --- \| --- \| --- \| --- \| --- \| --- \| --- \| --- \| --- \| --- \| --- \| --- \| --- \| --- \| --- \| --- \| --- \| --- \| --- \| --- \| --- \| --- \| --- \| --- \| --- \| --- \| --- \| --- \| --- \| --- \| --- \| --- \| --- \| --- \| --- \| --- \| --- \| --- \| --- \| --- \| --- \| --- \| --- \| --- \| --- \| --- \| --- \| --- \| --- \| --- \| --- \| --- \| --- \| --- \| --- \| --- \| --- \| --- \| --- \| --- \| --- \| --- \| --- \| --- \| --- \| --- \| --- \| --- \| --- \| --- \| --- \| --- \| --- \| --- \| --- \| --- \| --- \| --- \| --- \| --- \| --- \| --- \| --- \| --- \| --- \| --- \| --- \| --- \| --- \| --- \| --- \| --- \| --- \| --- \| --- \| --- \| --- \| --- \| --- \| --- \| --- \| --- \| --- \| --- \| --- \| --- \| --- \| --- \| --- \| --- \| --- \| --- \| --- \| --- \| --- \| --- \| --- \| --- \| --- \| --- \| --- \| --- \| --- \| --- \| --- \| --- \| --- \| --- \| --- \| --- \| --- \| --- \| --- \| --- \| --- \| --- \| --- \| --- \| --- \| --- \| --- \| --- \| --- \| --- \| --- \| --- \| --- \| --- \| --- \| --- \| --- \| --- \| --- \| --- \| --- \| --- \| --- \| --- \| --- \| --- \| --- \| --- \| --- \| --- \| --- \| --- \| --- \| --- \| --- \| --- \| --- \| --- \| --- \| --- \| --- \| --- \| --- \| --- \| --- \| --- \| --- \| --- \| --- \| --- \| --- \| --- \| --- \| --- \| --- \| --- \| --- \| --- \| --- \| --- \| --- \| --- \| --- \| --- \| --- \| --- \| --- \| --- \| --- \| --- \| --- \| --- \| --- \| --- \| --- \| --- \| --- \| --- \| --- \| --- \| --- \| --- \| --- \| --- \| --- \| --- \| --- \| --- \| --- \| --- \| --- \| --- \| --- \| --- \| --- \| --- \| --- \| --- \| --- \| --- \| --- \| --- \| --- \| --- \| --- \| --- \| --- \| --- \| --- \| --- \| --- \| --- \| --- \| --- \| --- \| --- \| --- \| --- \| --- \| --- \| --- \| --- \| --- \| --- \| --- \| --- \| --- \| --- \| --- \| --- \| --- \| --- \| --- \| --- \| --- \| --- \| --- \| --- \| --- \| --- \| --- \| --- \| --- \| --- \| --- \| --- \| --- \| --- \| --- \| --- \| --- \| --- \| --- \| --- \| --- \| --- \| --- \| --- \| --- \| --- \| --- \| --- \| --- \| --- \| --- \| --- \| --- \| --- \| --- \| --- \| --- \| --- \| --- \| --- \| --- \| --- \| --- \| --- \| --- \| --- \| --- \| --- \| --- \| --- \| --- \| --- \| --- \| --- \| --- \| --- \| --- \| --- \| --- \| --- \| --- \| --- \| --- \| --- \| --- \| --- \| --- \| --- \| --- \| --- \| --- \| --- \| --- \| --- \| --- \| --- \| --- \| --- \| --- \| --- \| --- \| --- \| --- \| --- \| --- \| --- \| --- \| --- \| --- \| --- \| --- \| --- \| --- \| --- \| --- \| --- \| --- \| --- \| --- \| --- \| --- \| --- \| --- \| --- \| --- \| --- \| --- \| --- \| --- \| --- \| --- \| --- \| --- \| --- \| --- \| --- \| --- \| --- \| --- \| --- \| --- \| --- \| --- \| --- \| --- \| --- \| --- \| --- \| --- \| --- \| --- \| --- \| --- \| --- \| --- \| --- \| --- \| --- \| --- \| --- \| --- \| --- \| --- \| --- \| --- \| --- \| --- \| --- \| --- \| --- \| --- \| --- \| --- \| --- \| --- \| --- \| --- \| --- \| --- \| --- \| --- \| --- \| --- \| --- \| --- \| --- \| --- \| --- \| --- \| --- \| --- \| --- \| --- \| --- \| --- \| --- \| --- \| --- \| --- \| --- \| --- \| --- \| --- \| --- \| --- \| --- \| --- \| --- \| --- \| --- \| --- \| --- \| --- \| --- \| --- \| --- \| --- \| --- \| --- \| --- \| --- \| --- \| --- \| --- \| --- \| --- \| --- \| --- \| --- \| --- \| --- \| --- \| --- \| --- \| --- \| --- \| --- \| --- \| --- \| --- \| --- \| --- \| --- \| --- \| --- \| --- \| --- \| --- \| --- \| --- \| --- \| --- \| --- \| --- \| --- \| --- \| --- \| --- \| --- \| --- \| --- \| --- \| --- \| --- \| --- \| --- \| --- \| --- \| --- \| --- \| --- \| --- \| --- \| --- \| --- \| --- \| --- \| --- \| --- \| --- \| --- \| --- \| --- \| --- \| --- \| --- \| --- \| --- \| --- \| --- \| --- \| --- \| --- \| --- \| --- \| --- \| --- \| --- \| --- \| --- \| --- \| --- \| --- \| --- \| --- \| --- \| --- \| --- \| --- \| --- \| --- \| --- \| --- \| --- \| --- \| --- \| --- \| --- \| --- \| --- \| --- \| --- \| --- \| --- \| --- \| --- \| --- \| --- \| --- \| --- \| --- \| --- \| --- \| --- \| --- \| --- \| --- \| --- \| --- \| --- \| --- \| --- \| --- \| --- \| --- \| --- \| --- \| --- \| --- \| --- \| --- \| --- \| --- \| --- \| --- \| --- \| --- \| --- \| --- \| --- \| --- \| --- \| --- \| --- \| --- \| --- \| --- \| --- \| --- \| --- \| --- \| --- \| --- \| --- \| --- \| --- \| --- \| --- \| --- \| --- \| --- \| --- \| --- \| --- \| --- \| --- \| --- \| --- \| --- \| --- \| --- \| --- \| --- \| --- \| --- \| --- \| --- \| --- \| --- \| --- \| --- \| --- \| --- \| --- \| --- \| --- \| --- \| --- \| --- \| --- \| --- \| --- \| --- \| --- \| --- \| --- \| --- \| --- \| --- \| --- \| --- \| --- \| --- \| --- \| --- \| --- \| --- \| --- \| --- \| --- \| --- \| --- \| --- \| --- \| --- \| --- \| --- \| --- \| --- \| --- \| --- \| --- \| --- \| --- \| --- \| --- \| --- \| --- \| --- \| --- \| --- \| --- \| --- \| --- \| --- \| --- \| --- \| --- \| --- \| --- \| --- \| --- \| --- \| --- \| --- \| --- \| --- \| --- \| --- \| --- \| --- \| --- \| --- \| --- \| --- \| --- \| --- \| --- \| --- \| --- \| --- \| --- \| --- \| --- \| --- \| --- \| --- \| --- \| --- \| --- \| --- \| --- \| --- \| --- \| --- \| --- \| --- \| --- \| --- \| --- \| --- \| --- \| --- \| --- \| --- \| --- \| --- \| --- \| --- \| --- \| --- \| --- \| --- \| --- \| --- \| --- \| --- \| --- \| --- \| --- \| --- \| --- \| --- \| --- \| --- \| --- \| --- \| --- \| --- \| --- \| --- \| --- \| --- \| --- \| --- \| --- \| --- \| --- \| --- \| --- \| --- \| --- \| --- \| --- \| --- \| --- \| --- \| --- \| --- \| --- \| --- \| --- \| --- \| --- \| --- \| --- \| --- \| --- \| --- \| --- \| --- \| --- \| --- \| --- \| --- \| --- \| --- \| --- \| --- \| --- \| --- \| --- \| --- \| --- \| --- \| --- \| --- \| --- \| --- \| --- \| --- \| --- \| --- \| --- \| --- \| --- \| --- \| --- \| --- \| --- \| --- \| --- \| --- \| --- \| --- \| --- \| --- \| --- \| --- \| --- \| --- \| --- \| --- \| --- \| --- \| --- \| --- \| --- \| --- \| --- \| --- \| --- \| --- \| --- \| --- \| --- \| --- \| --- \| --- \| --- \| --- \| --- \| --- \| --- \| --- \| --- \| --- \| --- \| --- \| --- \| --- \| --- \| --- \| --- \| --- \| --- \| --- \| --- \| --- \| --- \| --- \| --- \| --- \| --- \| --- \| --- \| --- \| --- \| --- \| --- \| --- \| --- \| --- \| --- \| --- \| --- \| --- \| --- \| --- \| --- \| --- \| --- \| --- \| --- \| --- \| --- \| --- \| --- \| --- \| --- \| --- \| --- \| --- \| --- \| --- \| --- \| --- \| --- \| --- \| --- \| --- \| --- \| --- \| --- \| --- \| --- \| --- \| --- \| --- \| --- \| --- \| --- \| --- \| --- \| --- \| --- \| --- \| --- \| --- \| --- \| --- \| --- \| --- \| --- \| --- \| --- \| --- \| --- \| --- \| --- \| --- \| --- \| --- \| --- \| --- \| --- \| --- \| --- \| --- \| --- \| --- \| --- \| --- \| --- \| --- \| --- \| --- \| --- \| --- \| --- \| --- \| --- \| --- \| --- \| --- \| --- \| --- \| --- \| --- \| --- \| --- \| --- \| --- \| --- \| --- \| --- \| --- \| --- \| --- \| --- \| --- \| --- \| --- \| --- \| --- \| --- \| --- \| --- \| --- \| --- \| --- \| --- \| --- \| --- \| --- \| --- \| --- \| --- \| --- \| --- \| --- \| --- \| --- \| --- \| --- \| --- \| --- \| --- \| --- \| --- \| --- \| --- \| --- \| --- \| --- \| --- \| --- \| --- \| --- \| --- \| --- \| --- \| --- \| --- \| --- \| --- \| --- \| --- \| --- \| --- \| --- \| --- \| --- \| --- \| --- \| --- \| --- \| --- \| --- \| --- \| --- \| --- \| --- \| --- \| --- \| --- \| --- \| --- \| --- \| --- \| --- \| --- \| --- \| --- \| --- \| --- \| --- \| --- \| --- \| --- \| --- \| --- \| --- \| --- \| --- \| --- \| --- \| --- \| --- \| --- \| --- \| --- \| --- \| --- \| --- \| --- \| --- \| --- \| --- \| --- \| --- \| --- \| --- \| --- \| --- \| --- \| --- \| --- \| --- \| --- \| --- \| --- \| --- \| --- \| --- \| --- \| --- \| --- \| --- \| --- \| --- \| --- \| --- \| --- \| --- \| --- \| --- \| --- \| --- \| --- \| --- \| --- \| --- \| --- \| --- \| --- \| --- \| --- \| --- \| --- \| --- \| --- \| --- \| --- \| --- \| --- \| --- \| --- \| --- \| --- \| --- \| --- \| --- \| --- \| --- \| --- \| --- \| --- \| --- \| --- \| --- \| --- \| --- \| --- \| --- \| --- \| --- \| --- \| --- \| --- \| --- \| --- \| --- \| --- \| --- \| --- \| --- \| --- \| --- \| --- \| --- \| --- \| --- \| --- \| --- \| --- \| --- \| --- \| --- \| --- \| --- \| --- \| --- \| --- \| --- \| --- \| --- \| --- \| --- \| --- \| --- \| --- \| --- \| --- \| --- \| --- \| --- \| --- \| --- \| --- \| --- \| --- \| --- \| --- \| --- \| --- \| --- \| --- \| --- \| --- \| --- \| --- \| --- \| --- \| --- \| --- \| --- \| --- \| --- \| --- \| --- \| --- \| --- \| --- \| --- \| --- \| --- \| --- \| --- \| --- \| --- \| --- \| --- \| --- \| --- \| --- \| --- \| --- \| --- \| --- \| --- \| --- \| --- \| --- \| --- \| --- \| --- \| --- \| --- \| --- \| --- \| --- \| --- \| --- \| --- \| --- \| --- \| --- \| --- \| --- \| --- \| --- \| --- \| --- \| --- \| --- \| --- \| --- \| --- \| --- \| --- \| --- \| --- \| --- \| --- \| --- \| --- \| --- \| --- \| --- \| --- \| --- \| --- \| --- \| --- \| --- \| --- \| --- \| --- \| --- \| --- \| --- \| --- \| --- \| --- \| --- \| --- \| --- \| --- \| --- \| --- \| --- \| --- \| --- \| --- \| --- \| --- \| --- \| --- \| --- \| --- \| --- \| --- \| --- \| --- \| --- \| --- \| --- \| --- \| --- \| --- \| --- \| --- \| --- \| --- \| --- \| --- \| --- \| --- \| --- \| --- \| --- \| --- \| --- \| --- \| --- \| --- \| --- \| --- \| --- \| --- \| --- \| --- \| --- \| --- \| --- \| --- \| --- \| --- \| --- \| --- \| --- \| --- \| --- \| --- \| --- \| --- \| --- \| --- \| --- \| --- \| --- \| --- \| --- \| --- \| --- \| --- \| --- \| --- \| --- \| --- \| --- \| --- \| --- \| --- \| --- \| --- \| --- \| --- \| --- \| --- \| --- \| --- \| --- \| --- \| --- \| --- \| --- \| --- \| --- \| --- \| --- \| --- \| --- \| --- \| --- \| --- \| --- \| --- \| --- \| --- \| --- \| --- \| --- \| --- \| --- \| --- \| --- \| --- \| --- \| --- \| --- \| --- \| --- \| --- \| --- \| --- \| --- \| --- \| --- \| --- \| --- \| --- \| --- \| --- \| --- \| --- \| --- \| --- \| --- \| --- \| --- \| --- \| --- \| --- \| --- \| --- \| --- \| --- \| --- \| --- \| --- \| --- \| --- \| --- \| --- \| --- \| --- \| --- \| --- \| --- \| --- \| --- \| --- \| --- \| --- \| --- \| --- \| --- \| --- \| --- \| --- \| --- \| --- \| --- \| --- \| --- \| --- \| --- \| --- \| --- \| --- \| --- \| --- \| --- \| --- \| --- \| --- \| --- \| --- \| --- \| --- \| --- \| --- \| --- \| --- \| --- \| --- \| --- \| --- \| --- \| --- \| --- \| --- \| --- \| --- \| --- \| --- \| --- \| --- \| --- \| --- \| --- \| --- \| --- \| --- \| --- \| --- \| --- \| --- \| --- \| --- \| --- \| --- \| --- \| --- \| --- \| --- \| --- \| --- \| --- \| --- \| --- \| --- \| --- \| --- \| --- \| --- \| --- \| --- \| --- \| --- \| --- \| --- \| --- \| --- \| --- \| --- \| --- \| --- \| --- \| --- \| --- \| --- \| --- \| --- \| --- \| --- \| --- \| --- \| --- \| --- \| --- \| --- \| --- \| --- \| --- \| --- \| --- \| --- \| --- \| --- \| --- \| --- \| --- \| --- \| --- \| --- \| --- \| --- \| --- \| --- \| --- \| --- \| --- \| --- \| --- \| --- \| --- \| --- \| --- \| --- \| --- \| --- \| --- \| --- \| --- \| --- \| --- \| --- \| --- \| --- \| --- \| --- \| --- \| --- \| --- \| --- \| --- \| --- \| --- \| --- \| --- \| --- \| --- \| --- \| --- \| --- \| --- \| --- \| --- \| --- \| --- \| --- \| --- \| --- \| --- \| --- \| --- \| --- \| --- \| --- \| --- \| --- \| --- \| --- \| --- \| --- \| --- \| --- \| --- \| --- \| --- \| --- \| --- \| --- \| --- \| --- \| --- \| --- \| --- \| --- \| --- \| --- \| --- \| --- \| --- \| --- \| --- \| --- \| --- \| --- \| --- \| --- \| --- \| --- \| --- \| --- \| --- \| --- \| --- \| --- \| --- \| --- \| --- \| --- \| --- \| --- \| --- \| --- \| --- \| --- \| --- \| --- \| --- \| --- \| --- \| --- \| --- \| --- \| --- \| --- \| --- \| --- \| --- \| --- \| --- \| --- \| --- \| --- \| --- \| --- \| --- \| --- \| --- \| --- \| --- \| --- \| --- \| --- \| --- \| --- \| --- \| --- \| --- \| --- \| --- \| --- \| --- \| --- \| --- \| --- \| --- \| --- \| --- \| --- \| --- \| --- \| --- \| --- \| --- \| --- \| --- \| --- \| --- \| --- \| --- \| --- \| --- \| --- \| --- \| --- \| --- \| --- \| --- \| --- \| --- \| --- \| --- \| --- \| --- \| --- \| --- \| --- \| --- \| --- \| --- \| --- \| --- \| --- \| --- \| --- \| --- \| --- \| --- \| --- \| --- \| --- \| --- \| --- \| --- \| --- \| --- \| --- \| --- \| --- \| --- \| --- \| --- \| --- \| --- \| --- \| --- \| --- \| --- \| --- \| --- \| --- \| --- \| --- \| --- \| --- \| --- \| --- \| --- \| --- \| --- \| --- \| --- \| --- \| --- \| --- \| --- \| --- \| --- \| --- \| --- \| --- \| --- \| --- \| --- \| --- \| --- \| --- \| --- \| --- \| --- \| --- \| --- \| --- \| --- \| --- \| --- \| --- \| --- \| --- \| --- \| --- \| --- \| --- \| --- \| --- \| --- \| --- \| --- \| --- \| --- \| --- \| --- \| --- \| --- \| --- \| --- \| --- \| --- \| --- \| --- \| --- \| --- \| --- \| --- \| --- \| --- \| --- \| --- \| --- \| --- \| --- \| --- \| --- \| --- \| --- \| --- \| --- \| --- \| --- \| --- \| --- \| --- \| --- \| --- \| --- \| --- \| --- \| --- \| --- \| --- \| --- \| --- \| --- \| --- \| --- \| --- \| --- \| --- \| --- \| --- \| --- \| --- \| --- \| --- \| --- \| --- \| --- \| --- \| --- \| --- \| --- \| --- \| --- \| --- \| --- \| --- \| --- \| --- \| --- \| --- \| --- \| --- \| --- \| --- \| --- \| --- \| --- \| --- \| --- \| --- \| --- \| --- \| --- \| --- \| --- \| --- \| --- \| --- \| --- \| --- \| --- \| --- \| --- \| --- \| --- \| --- \| --- \| --- \| --- \| --- \| --- \| --- \| --- \| --- \| --- \| --- \| --- \| --- \| --- \| --- \| --- \| --- \| --- \| --- \| --- \| --- \| --- \| --- \| --- \| --- \| --- \| --- \| --- \| --- \| --- \| --- \| --- \| --- \| --- \| --- \| --- \| --- \| --- \| --- \| --- \| --- \| --- \| --- \| --- \| --- \| --- \| --- \| --- \| --- \| --- \| --- \| --- \| --- \| --- \| --- \| --- \| --- \| --- \| --- \| --- \| --- \| --- \| --- \| --- \| --- \| --- \| --- \| --- \| --- \| --- \| --- \| --- \| --- \| --- \| --- \| --- \| --- \| --- \| --- \| --- \| --- \| --- \| --- \| --- \| --- \| --- \| --- \| --- \| --- \| --- \| --- \| --- \| --- \| --- \| --- \| --- \| --- \| --- \| --- \| --- \| --- \| --- \| --- \| --- \| --- \| --- \| --- \| --- \| --- \| --- \| --- \| --- \| --- \| --- \| --- \| --- \| --- \| --- \| --- \| --- \| --- \| --- \| --- \| --- \| --- \| --- \| --- \| --- \| --- \| --- \| --- \| --- \| --- \| --- \| --- \| --- \| --- \| --- \| --- \| --- \| --- \| --- \| --- \| --- \| --- \| --- \| --- \| --- \| --- \| --- \| --- \| --- \| --- \| --- \| --- \| --- \| --- \| --- \| --- \| --- \| --- \| --- \| --- \| --- \| --- \| --- \| --- \| --- \| --- \| --- \| --- \| --- \| --- \| --- \| --- \| --- \| --- \| --- \| --- \| --- \| --- \| --- \| --- \| --- \| --- \| --- \| --- \| --- \| --- \| --- \| --- \| --- \| --- \| --- \| --- \| --- \| --- \| --- \| --- \| --- \| --- \| --- \| --- \| --- \| --- \| --- \| --- \| --- \| --- \| --- \| --- \| --- \| --- \| --- \| --- \| --- \| --- \| --- \| --- \| --- \| --- \| --- \| --- \| --- \| --- \| --- \| --- \| --- \| --- \| --- \| --- \| --- \| --- \| --- \| --- \| --- \| --- \| --- \| --- \| --- \| --- \| --- \| --- \| --- \| --- \| --- \| --- \| --- \| --- \| --- \| --- \| --- \| --- \| --- \| --- \| --- \| --- \| --- \| --- \| --- \| --- \| --- \| --- \| --- \| --- \| --- \| --- \| --- \| --- \| --- \| --- \| --- \| --- \| --- \| --- \| --- \| --- \| --- \| --- \| --- \| --- \| --- \| --- \| --- \| --- \| --- \| --- \| --- \| --- \| --- \| --- \| --- \| --- \| --- \| --- \| --- \| --- \| --- \| --- \| --- \| --- \| --- \| --- \| --- \| --- \| --- \| --- \| --- \| --- \| --- \| --- \| --- \| --- \| --- \| --- \| --- \| --- \| --- \| --- \| --- \| --- \| --- \| --- \| --- \| --- \| --- \| --- \| --- \| --- \| --- \| --- \| --- \| --- \| --- \| --- \| --- \| --- \| --- \| --- \| --- \| --- \| --- \| --- \| --- \| --- \| --- \| --- \| --- \| --- \| --- \| --- \| --- \| --- \| --- \| --- \| --- \| --- \| --- \| --- \| --- \| --- \| --- \| --- \| --- \| --- \| --- \| --- \| --- \| --- \| --- \| --- \| --- \| --- \| --- \| --- \| --- \| --- \| --- \| --- \| \| \| \| \| *** Misclassified observation** \| \| --- \| \| \| --- \| --- \| \| \|  \| \| \| \| \| \| **Discriminant Analysis Results** \| \| --- \| \| \| --- \| --- \| \| \| \| **The DISCRIM Procedure Classification Summary for Calibration Data: WORK.SORTTEMPTABLESORTED Resubstitution Summary using Linear Discriminant Function** \| \| --- \| \| \| --- \| --- \| \| \| \| **Number of Observations and Percent Classified into DIST** \| \| \| \| \| \| \| \| \| --- \| --- \| --- \| --- \| --- \| --- \| --- \| --- \| \| **From DIST** \| **1** \| **2** \| **3** \| **4** \| **5** \| **6** \| **Total** \| \| 1 \| \| 21 \| \| --- \| \| 42.00 \| \| \| 10 \| \| --- \| \| 20.00 \| \| \| 2 \| \| --- \| \| 4.00 \| \| \| 10 \| \| --- \| \| 20.00 \| \| \| 5 \| \| --- \| \| 10.00 \| \| \| 2 \| \| --- \| \| 4.00 \| \| \| 50 \| \| --- \| \| 100.00 \| \| \| 2 \| \| 13 \| \| --- \| \| 20.63 \| \| \| 44 \| \| --- \| \| 69.84 \| \| \| 3 \| \| --- \| \| 4.76 \| \| \| 3 \| \| --- \| \| 4.76 \| \| \| 0 \| \| --- \| \| 0.00 \| \| \| 0 \| \| --- \| \| 0.00 \| \| \| 63 \| \| --- \| \| 100.00 \| \| \| 3 \| \| 0 \| \| --- \| \| 0.00 \| \| \| 9 \| \| --- \| \| 20.93 \| \| \| 28 \| \| --- \| \| 65.12 \| \| \| 0 \| \| --- \| \| 0.00 \| \| \| 4 \| \| --- \| \| 9.30 \| \| \| 2 \| \| --- \| \| 4.65 \| \| \| 43 \| \| --- \| \| 100.00 \| \| \| 4 \| \| 6 \| \| --- \| \| 10.91 \| \| \| 0 \| \| --- \| \| 0.00 \| \| \| 0 \| \| --- \| \| 0.00 \| \| \| 47 \| \| --- \| \| 85.45 \| \| \| 0 \| \| --- \| \| 0.00 \| \| \| 2 \| \| --- \| \| 3.64 \| \| \| 55 \| \| --- \| \| 100.00 \| \| \| 5 \| \| 0 \| \| --- \| \| 0.00 \| \| \| 0 \| \| --- \| \| 0.00 \| \| \| 10 \| \| --- \| \| 16.67 \| \| \| 0 \| \| --- \| \| 0.00 \| \| \| 50 \| \| --- \| \| 83.33 \| \| \| 0 \| \| --- \| \| 0.00 \| \| \| 60 \| \| --- \| \| 100.00 \| \| \| 6 \| \| 0 \| \| --- \| \| 0.00 \| \| \| 1 \| \| --- \| \| 1.92 \| \| \| 0 \| \| --- \| \| 0.00 \| \| \| 0 \| \| --- \| \| 0.00 \| \| \| 15 \| \| --- \| \| 28.85 \| \| \| 36 \| \| --- \| \| 69.23 \| \| \| 52 \| \| --- \| \| 100.00 \| \| \| Total \| \| 40 \| \| --- \| \| 12.38 \| \| \| 64 \| \| --- \| \| 19.81 \| \| \| 43 \| \| --- \| \| 13.31 \| \| \| 60 \| \| --- \| \| 18.58 \| \| \| 74 \| \| --- \| \| 22.91 \| \| \| 42 \| \| --- \| \| 13.00 \| \| \| 323 \| \| --- \| \| 100.00 \| \| \| Priors \| \| 0.1548 \| \| --- \| \|  \| \| \| 0.19505 \| \| --- \| \|  \| \| \| 0.13313 \| \| --- \| \|  \| \| \| 0.17028 \| \| --- \| \|  \| \| \| 0.18576 \| \| --- \| \|  \| \| \| 0.16099 \| \| --- \| \|  \| \| \|  \| \| --- \| \|  \| \| \| \| --- \| --- \| --- \| --- \| --- \| --- \| --- \| --- \| --- \| --- \| --- \| --- \| --- \| --- \| --- \| --- \| --- \| --- \| --- \| --- \| --- \| --- \| --- \| --- \| --- \| --- \| --- \| --- \| --- \| --- \| --- \| --- \| --- \| --- \| --- \| --- \| --- \| --- \| --- \| --- \| --- \| --- \| --- \| --- \| --- \| --- \| --- \| --- \| --- \| --- \| --- \| --- \| --- \| --- \| --- \| --- \| --- \| --- \| --- \| --- \| --- \| --- \| --- \| --- \| --- \| --- \| --- \| --- \| --- \| --- \| --- \| --- \| --- \| --- \| --- \| --- \| --- \| --- \| --- \| --- \| --- \| --- \| --- \| --- \| --- \| --- \| --- \| --- \| --- \| --- \| --- \| --- \| --- \| --- \| --- \| --- \| --- \| --- \| --- \| --- \| --- \| --- \| --- \| --- \| --- \| --- \| --- \| --- \| --- \| --- \| --- \| --- \| --- \| --- \| --- \| --- \| --- \| --- \| --- \| --- \| --- \| --- \| --- \| --- \| --- \| --- \| --- \| --- \| --- \| --- \| --- \| --- \| --- \| --- \| --- \| --- \| --- \| --- \| --- \| --- \| --- \| --- \| --- \| --- \| --- \| --- \| --- \| --- \| --- \| --- \| --- \| --- \| --- \| --- \| --- \| --- \| --- \| --- \| --- \| --- \| --- \| --- \| --- \| --- \| --- \| --- \| --- \| --- \| --- \| --- \| --- \| --- \| --- \| --- \| --- \| --- \| --- \| --- \| --- \| --- \| --- \| --- \| --- \| --- \| --- \| --- \| --- \| --- \| --- \| --- \| --- \| --- \| --- \| \| \| **Error Count Estimates for DIST** \| \| \| \| \| \| \| \| \| --- \| --- \| --- \| --- \| --- \| --- \| --- \| --- \| \|  \| **1** \| **2** \| **3** \| **4** \| **5** \| **6** \| **Total** \| \| Rate \| 0.5800 \| 0.3016 \| 0.3488 \| 0.1455 \| 0.1667 \| 0.3077 \| 0.3003 \| \| Priors \| 0.1548 \| 0.1950 \| 0.1331 \| 0.1703 \| 0.1858 \| 0.1610 \|  \| \| \| \|  \| \| \| \| \| \| **Discriminant Analysis Results** \| \| --- \| \| \| --- \| --- \| \| \| \| **The DISCRIM Procedure Classification Results for Calibration Data: WORK.SORTTEMPTABLESORTED Resubstitution Results using Linear Discriminant Function** \| \| --- \| \| \| --- \| --- \| \| \| \| **Number of Observations and Average Posterior Probabilities Classified into DIST** \| \| \| \| \| \| \| \| --- \| --- \| --- \| --- \| --- \| --- \| --- \| \| **From DIST** \| **1** \| **2** \| **3** \| **4** \| **5** \| **6** \| \| 1 \| \| 21 \| \| --- \| \| 0.6211 \| \| \| 10 \| \| --- \| \| 0.5682 \| \| \| 2 \| \| --- \| \| 0.3462 \| \| \| 10 \| \| --- \| \| 0.5536 \| \| \| 5 \| \| --- \| \| 0.3833 \| \| \| 2 \| \| --- \| \| 0.7089 \| \| \| 2 \| \| 13 \| \| --- \| \| 0.3784 \| \| \| 44 \| \| --- \| \| 0.7492 \| \| \| 3 \| \| --- \| \| 0.6799 \| \| \| 3 \| \| --- \| \| 0.3916 \| \| \| 0 \| \| --- \| \| . \| \| \| 0 \| \| --- \| \| . \| \| \| 3 \| \| 0 \| \| --- \| \| . \| \| \| 9 \| \| --- \| \| 0.7361 \| \| \| 28 \| \| --- \| \| 0.7023 \| \| \| 0 \| \| --- \| \| . \| \| \| 4 \| \| --- \| \| 0.6316 \| \| \| 2 \| \| --- \| \| 0.6395 \| \| \| 4 \| \| 6 \| \| --- \| \| 0.4172 \| \| \| 0 \| \| --- \| \| . \| \| \| 0 \| \| --- \| \| . \| \| \| 47 \| \| --- \| \| 0.7144 \| \| \| 0 \| \| --- \| \| . \| \| \| 2 \| \| --- \| \| 0.4237 \| \| \| 5 \| \| 0 \| \| --- \| \| . \| \| \| 0 \| \| --- \| \| . \| \| \| 10 \| \| --- \| \| 0.4924 \| \| \| 0 \| \| --- \| \| . \| \| \| 50 \| \| --- \| \| 0.7658 \| \| \| 0 \| \| --- \| \| . \| \| \| 6 \| \| 0 \| \| --- \| \| . \| \| \| 1 \| \| --- \| \| 0.3341 \| \| \| 0 \| \| --- \| \| . \| \| \| 0 \| \| --- \| \| . \| \| \| 15 \| \| --- \| \| 0.6604 \| \| \| 36 \| \| --- \| \| 0.8277 \| \| \| Total \| \| 40 \| \| --- \| \| 0.5116 \| \| \| 64 \| \| --- \| \| 0.7126 \| \| \| 43 \| \| --- \| \| 0.6353 \| \| \| 60 \| \| --- \| \| 0.6715 \| \| \| 74 \| \| --- \| \| 0.7113 \| \| \| 42 \| \| --- \| \| 0.7938 \| \| \| Priors \| \| 0.1548 \| \| --- \| \|  \| \| \| 0.19505 \| \| --- \| \|  \| \| \| 0.13313 \| \| --- \| \|  \| \| \| 0.17028 \| \| --- \| \|  \| \| \| 0.18576 \| \| --- \| \|  \| \| \| 0.16099 \| \| --- \| \|  \| \| \| \| --- \| --- \| --- \| --- \| --- \| --- \| --- \| --- \| --- \| --- \| --- \| --- \| --- \| --- \| --- \| --- \| --- \| --- \| --- \| --- \| --- \| --- \| --- \| --- \| --- \| --- \| --- \| --- \| --- \| --- \| --- \| --- \| --- \| --- \| --- \| --- \| --- \| --- \| --- \| --- \| --- \| --- \| --- \| --- \| --- \| --- \| --- \| --- \| --- \| --- \| --- \| --- \| --- \| --- \| --- \| --- \| --- \| --- \| --- \| --- \| --- \| --- \| --- \| --- \| --- \| --- \| --- \| --- \| --- \| --- \| --- \| --- \| --- \| --- \| --- \| --- \| --- \| --- \| --- \| --- \| --- \| --- \| --- \| --- \| --- \| --- \| --- \| --- \| --- \| --- \| --- \| --- \| --- \| --- \| --- \| --- \| --- \| --- \| --- \| --- \| --- \| --- \| --- \| --- \| --- \| --- \| --- \| --- \| --- \| --- \| --- \| --- \| --- \| --- \| --- \| --- \| --- \| --- \| --- \| --- \| --- \| --- \| --- \| --- \| --- \| --- \| --- \| --- \| --- \| --- \| --- \| --- \| --- \| --- \| --- \| --- \| --- \| --- \| --- \| --- \| --- \| --- \| --- \| --- \| --- \| --- \| --- \| --- \| --- \| --- \| --- \| --- \| --- \| --- \| --- \| --- \| --- \| --- \| --- \| --- \| --- \| --- \| --- \| --- \| --- \| --- \| --- \| \| \| **Posterior Probability Error Rate Estimates for DIST** \| \| \| \| \| \| \| \| \| --- \| --- \| --- \| --- \| --- \| --- \| --- \| --- \| \| **Estimate** \| **1** \| **2** \| **3** \| **4** \| **5** \| **6** \| **Total** \| \| Stratified \| 0.5907 \| 0.2761 \| 0.3647 \| 0.2675 \| 0.1227 \| 0.3588 \| 0.3199 \| \| Unstratified \| 0.5907 \| 0.2761 \| 0.3647 \| 0.2675 \| 0.1227 \| 0.3588 \| 0.3199 \| \| Priors \| 0.1548 \| 0.1950 \| 0.1331 \| 0.1703 \| 0.1858 \| 0.1610 \|  \| \| \| \|  \| \| \| \| \| \| **Discriminant Analysis Results** \| \| --- \| \| \| --- \| --- \| \| \| \| **The DISCRIM Procedure Classification Results for Calibration Data: WORK.SORTTEMPTABLESORTED Cross-validation Results using Linear Discriminant Function** \| \| --- \| \| \| --- \| --- \| \| \| \| **Posterior Probability of Membership in DIST** \| \| \| \| \| \| \| \| \| \| \| --- \| --- \| --- \| --- \| --- \| --- \| --- \| --- \| --- \| --- \| \| **Obs** \| **From DIST** \| **Classified into DIST** \| \| **1** \| **2** \| **3** \| **4** \| **5** \| **6** \| \| 1 \| 1 \| 1 \|  \| 0.6653 \| 0.0164 \| 0.0472 \| 0.1820 \| 0.0883 \| 0.0008 \| \| 2 \| 1 \| 4 \| * \| 0.3399 \| 0.2620 \| 0.0052 \| 0.3917 \| 0.0007 \| 0.0004 \| \| 3 \| 1 \| 1 \|  \| 0.7850 \| 0.0676 \| 0.0327 \| 0.1070 \| 0.0070 \| 0.0007 \| \| 4 \| 1 \| 1 \|  \| 0.6772 \| 0.0994 \| 0.0656 \| 0.1563 \| 0.0015 \| 0.0001 \| \| 5 \| 1 \| 4 \| * \| 0.4150 \| 0.0115 \| 0.0193 \| 0.4753 \| 0.0720 \| 0.0069 \| \| 6 \| 1 \| 4 \| * \| 0.3577 \| 0.1614 \| 0.0014 \| 0.4756 \| 0.0003 \| 0.0036 \| \| 7 \| 1 \| 4 \| * \| 0.3287 \| 0.0065 \| 0.0248 \| 0.5533 \| 0.0837 \| 0.0031 \| \| 8 \| 1 \| 1 \|  \| 0.7203 \| 0.0216 \| 0.0049 \| 0.2505 \| 0.0024 \| 0.0004 \| \| 9 \| 1 \| 1 \|  \| 0.5667 \| 0.0151 \| 0.0309 \| 0.2961 \| 0.0905 \| 0.0007 \| \| 10 \| 1 \| 1 \|  \| 0.7041 \| 0.1584 \| 0.0419 \| 0.0905 \| 0.0050 \| 0.0002 \| \| 11 \| 1 \| 1 \|  \| 0.5879 \| 0.0166 \| 0.0424 \| 0.2995 \| 0.0534 \| 0.0002 \| \| 12 \| 1 \| 1 \|  \| 0.7467 \| 0.0121 \| 0.0027 \| 0.2335 \| 0.0038 \| 0.0012 \| \| 13 \| 1 \| 1 \|  \| 0.6960 \| 0.1207 \| 0.0627 \| 0.1148 \| 0.0055 \| 0.0002 \| \| 14 \| 1 \| 1 \|  \| 0.6690 \| 0.1010 \| 0.0705 \| 0.1563 \| 0.0028 \| 0.0004 \| \| 15 \| 1 \| 1 \|  \| 0.4460 \| 0.4084 \| 0.0709 \| 0.0724 \| 0.0014 \| 0.0008 \| \| 16 \| 1 \| 4 \| * \| 0.3286 \| 0.0041 \| 0.0049 \| 0.3899 \| 0.1718 \| 0.1007 \| \| 17 \| 1 \| 4 \| * \| 0.3025 \| 0.0039 \| 0.0121 \| 0.5236 \| 0.1427 \| 0.0151 \| \| 18 \| 1 \| 1 \|  \| 0.7565 \| 0.1003 \| 0.0305 \| 0.1042 \| 0.0080 \| 0.0006 \| \| 19 \| 1 \| 1 \|  \| 0.7244 \| 0.1507 \| 0.0487 \| 0.0630 \| 0.0119 \| 0.0014 \| \| 20 \| 1 \| 1 \|  \| 0.4823 \| 0.3857 \| 0.0533 \| 0.0734 \| 0.0052 \| 0.0002 \| \| 21 \| 1 \| 1 \|  \| 0.3927 \| 0.2096 \| 0.0014 \| 0.3834 \| 0.0006 \| 0.0123 \| \| 22 \| 1 \| 1 \|  \| 0.5961 \| 0.0263 \| 0.0606 \| 0.1893 \| 0.1241 \| 0.0036 \| \| 23 \| 1 \| 1 \|  \| 0.6529 \| 0.0232 \| 0.0581 \| 0.1449 \| 0.1197 \| 0.0012 \| \| 24 \| 1 \| 2 \| * \| 0.1560 \| 0.6660 \| 0.0266 \| 0.1207 \| 0.0281 \| 0.0026 \| \| 25 \| 1 \| 6 \| * \| 0.0112 \| 0.0025 \| 0.0016 \| 0.0066 \| 0.2949 \| 0.6832 \| \| 26 \| 1 \| 4 \| * \| 0.3169 \| 0.1062 \| 0.0124 \| 0.4872 \| 0.0649 \| 0.0123 \| \| 27 \| 1 \| 2 \| * \| 0.2512 \| 0.3737 \| 0.0700 \| 0.2829 \| 0.0221 \| 0.0001 \| \| 28 \| 1 \| 4 \| * \| 0.0949 \| 0.0021 \| 0.0047 \| 0.8741 \| 0.0200 \| 0.0043 \| \| 29 \| 1 \| 4 \| * \| 0.2682 \| 0.0003 \| 0.0112 \| 0.3657 \| 0.1737 \| 0.1808 \| \| 30 \| 1 \| 4 \| * \| 0.3749 \| 0.1331 \| 0.0059 \| 0.4431 \| 0.0313 \| 0.0117 \| \| 31 \| 1 \| 6 \| * \| 0.0151 \| 0.0027 \| 0.0015 \| 0.0096 \| 0.1335 \| 0.8375 \| \| 32 \| 1 \| 5 \| * \| 0.1393 \| 0.1013 \| 0.2949 \| 0.0060 \| 0.3745 \| 0.0840 \| \| 33 \| 1 \| 1 \|  \| 0.4016 \| 0.3633 \| 0.0040 \| 0.1532 \| 0.0699 \| 0.0081 \| \| 34 \| 1 \| 1 \|  \| 0.4348 \| 0.0097 \| 0.1031 \| 0.0635 \| 0.1759 \| 0.2130 \| \| 35 \| 1 \| 4 \| * \| 0.1959 \| 0.0155 \| 0.0074 \| 0.7223 \| 0.0208 \| 0.0381 \| \| 36 \| 1 \| 4 \| * \| 0.1180 \| 0.0019 \| 0.0036 \| 0.8338 \| 0.0393 \| 0.0035 \| \| 37 \| 1 \| 5 \| * \| 0.0288 \| 0.0054 \| 0.0257 \| 0.0207 \| 0.5657 \| 0.3537 \| \| 38 \| 1 \| 2 \| * \| 0.1878 \| 0.3801 \| 0.0855 \| 0.3371 \| 0.0095 \| 0.0000 \| \| 39 \| 1 \| 3 \| * \| 0.0956 \| 0.1937 \| 0.3193 \| 0.0186 \| 0.2844 \| 0.0884 \| \| 40 \| 1 \| 3 \| * \| 0.0951 \| 0.2989 \| 0.4327 \| 0.0716 \| 0.1014 \| 0.0004 \| \| 41 \| 1 \| 2 \| * \| 0.1077 \| 0.8190 \| 0.0147 \| 0.0147 \| 0.0256 \| 0.0184 \| \| 42 \| 1 \| 5 \| * \| 0.0746 \| 0.1361 \| 0.2244 \| 0.0118 \| 0.4225 \| 0.1306 \| \| 43 \| 1 \| 2 \| * \| 0.1402 \| 0.3479 \| 0.3173 \| 0.1281 \| 0.0657 \| 0.0009 \| \| 44 \| 1 \| 2 \| * \| 0.1112 \| 0.5309 \| 0.2339 \| 0.1078 \| 0.0160 \| 0.0001 \| \| 45 \| 1 \| 2 \| * \| 0.0870 \| 0.8361 \| 0.0180 \| 0.0064 \| 0.0402 \| 0.0123 \| \| 46 \| 1 \| 5 \| * \| 0.1494 \| 0.1568 \| 0.2211 \| 0.0241 \| 0.3505 \| 0.0980 \| \| 47 \| 1 \| 2 \| * \| 0.1894 \| 0.7150 \| 0.0212 \| 0.0299 \| 0.0256 \| 0.0189 \| \| 48 \| 1 \| 2 \| * \| 0.1227 \| 0.4940 \| 0.2472 \| 0.1025 \| 0.0330 \| 0.0005 \| \| 49 \| 1 \| 2 \| * \| 0.0904 \| 0.8708 \| 0.0092 \| 0.0153 \| 0.0084 \| 0.0060 \| \| 50 \| 1 \| 5 \| * \| 0.1920 \| 0.1262 \| 0.1791 \| 0.0539 \| 0.2744 \| 0.1745 \| \| 51 \| 2 \| 2 \|  \| 0.1653 \| 0.4817 \| 0.0051 \| 0.3456 \| 0.0021 \| 0.0002 \| \| 52 \| 2 \| 2 \|  \| 0.1950 \| 0.4994 \| 0.0714 \| 0.2342 \| 0.0001 \| 0.0000 \| \| 53 \| 2 \| 1 \| * \| 0.3669 \| 0.2188 \| 0.2685 \| 0.1089 \| 0.0280 \| 0.0089 \| \| 54 \| 2 \| 6 \| * \| 0.0000 \| 0.0000 \| 0.0000 \| 0.0000 \| 0.0000 \| 1.0000 \| \| 55 \| 2 \| 4 \| * \| 0.2647 \| 0.2263 \| 0.0041 \| 0.4931 \| 0.0086 \| 0.0031 \| \| 56 \| 2 \| 2 \|  \| 0.0156 \| 0.8637 \| 0.1171 \| 0.0008 \| 0.0013 \| 0.0016 \| \| 57 \| 2 \| 2 \|  \| 0.2798 \| 0.4279 \| 0.0589 \| 0.2324 \| 0.0008 \| 0.0001 \| \| 58 \| 2 \| 4 \| * \| 0.3851 \| 0.1462 \| 0.0744 \| 0.3915 \| 0.0021 \| 0.0007 \| \| 59 \| 2 \| 1 \| * \| 0.4162 \| 0.1819 \| 0.2728 \| 0.0907 \| 0.0363 \| 0.0021 \| \| 60 \| 2 \| 1 \| * \| 0.3695 \| 0.1742 \| 0.1115 \| 0.3417 \| 0.0027 \| 0.0004 \| \| 61 \| 2 \| 2 \|  \| 0.0459 \| 0.8014 \| 0.1185 \| 0.0084 \| 0.0210 \| 0.0048 \| \| 62 \| 2 \| 4 \| * \| 0.1903 \| 0.3390 \| 0.0037 \| 0.4607 \| 0.0057 \| 0.0007 \| \| 63 \| 2 \| 2 \|  \| 0.2791 \| 0.3394 \| 0.0911 \| 0.2895 \| 0.0008 \| 0.0001 \| \| 64 \| 2 \| 2 \|  \| 0.3067 \| 0.3275 \| 0.2810 \| 0.0759 \| 0.0068 \| 0.0021 \| \| 65 \| 2 \| 2 \|  \| 0.0070 \| 0.8119 \| 0.1792 \| 0.0012 \| 0.0005 \| 0.0003 \| \| 66 \| 2 \| 2 \|  \| 0.0670 \| 0.9186 \| 0.0011 \| 0.0131 \| 0.0002 \| 0.0000 \| \| 67 \| 2 \| 3 \| * \| 0.1685 \| 0.0479 \| 0.6529 \| 0.0528 \| 0.0666 \| 0.0113 \| \| 68 \| 2 \| 2 \|  \| 0.1609 \| 0.7269 \| 0.0093 \| 0.0565 \| 0.0164 \| 0.0301 \| \| 69 \| 2 \| 1 \| * \| 0.4015 \| 0.1110 \| 0.1900 \| 0.0793 \| 0.2136 \| 0.0047 \| \| 70 \| 2 \| 2 \|  \| 0.2306 \| 0.3854 \| 0.0353 \| 0.1918 \| 0.1551 \| 0.0019 \| \| 71 \| 2 \| 2 \|  \| 0.1438 \| 0.7505 \| 0.0089 \| 0.0534 \| 0.0148 \| 0.0287 \| \| 72 \| 2 \| 2 \|  \| 0.1470 \| 0.7392 \| 0.0036 \| 0.0145 \| 0.0905 \| 0.0052 \| \| 73 \| 2 \| 2 \|  \| 0.0995 \| 0.8306 \| 0.0090 \| 0.0293 \| 0.0121 \| 0.0194 \| \| 74 \| 2 \| 1 \| * \| 0.3748 \| 0.0605 \| 0.3133 \| 0.1609 \| 0.0550 \| 0.0354 \| \| 75 \| 2 \| 1 \| * \| 0.5763 \| 0.0780 \| 0.0592 \| 0.1224 \| 0.1514 \| 0.0126 \| \| 76 \| 2 \| 2 \|  \| 0.1385 \| 0.7609 \| 0.0033 \| 0.0140 \| 0.0769 \| 0.0064 \| \| 77 \| 2 \| 2 \|  \| 0.1235 \| 0.6871 \| 0.0417 \| 0.1031 \| 0.0439 \| 0.0006 \| \| 78 \| 2 \| 2 \|  \| 0.1188 \| 0.8280 \| 0.0028 \| 0.0163 \| 0.0315 \| 0.0026 \| \| 79 \| 2 \| 2 \|  \| 0.0222 \| 0.9515 \| 0.0031 \| 0.0148 \| 0.0015 \| 0.0069 \| \| 80 \| 2 \| 1 \| * \| 0.3489 \| 0.1455 \| 0.0256 \| 0.2812 \| 0.1966 \| 0.0022 \| \| 81 \| 2 \| 3 \| * \| 0.1054 \| 0.0106 \| 0.7438 \| 0.0461 \| 0.0890 \| 0.0051 \| \| 82 \| 2 \| 1 \| * \| 0.3547 \| 0.0295 \| 0.2457 \| 0.1172 \| 0.2485 \| 0.0043 \| \| 83 \| 2 \| 2 \|  \| 0.1040 \| 0.6504 \| 0.0314 \| 0.1792 \| 0.0267 \| 0.0084 \| \| 84 \| 2 \| 2 \|  \| 0.3101 \| 0.4230 \| 0.0177 \| 0.1623 \| 0.0490 \| 0.0380 \| \| 85 \| 2 \| 4 \| * \| 0.2403 \| 0.1040 \| 0.0357 \| 0.3133 \| 0.3046 \| 0.0021 \| \| 86 \| 2 \| 2 \|  \| 0.2757 \| 0.3532 \| 0.0081 \| 0.0345 \| 0.3141 \| 0.0143 \| \| 87 \| 2 \| 2 \|  \| 0.1996 \| 0.6429 \| 0.0339 \| 0.0798 \| 0.0336 \| 0.0102 \| \| 88 \| 2 \| 3 \| * \| 0.1290 \| 0.0620 \| 0.7141 \| 0.0298 \| 0.0574 \| 0.0076 \| \| 89 \| 2 \| 1 \| * \| 0.3323 \| 0.1730 \| 0.2384 \| 0.0514 \| 0.2012 \| 0.0037 \| \| 90 \| 2 \| 2 \|  \| 0.0576 \| 0.8587 \| 0.0069 \| 0.0535 \| 0.0069 \| 0.0164 \| \| 91 \| 2 \| 1 \| * \| 0.3708 \| 0.2685 \| 0.0139 \| 0.2472 \| 0.0514 \| 0.0482 \| \| 92 \| 2 \| 1 \| * \| 0.3705 \| 0.2331 \| 0.0104 \| 0.3367 \| 0.0227 \| 0.0266 \| \| 93 \| 2 \| 2 \|  \| 0.0461 \| 0.9008 \| 0.0072 \| 0.0292 \| 0.0061 \| 0.0106 \| \| 94 \| 2 \| 1 \| * \| 0.3749 \| 0.1585 \| 0.0209 \| 0.2777 \| 0.1050 \| 0.0629 \| \| 95 \| 2 \| 2 \|  \| 0.0079 \| 0.9713 \| 0.0190 \| 0.0008 \| 0.0007 \| 0.0003 \| \| 96 \| 2 \| 4 \| * \| 0.0980 \| 0.3849 \| 0.0458 \| 0.4668 \| 0.0023 \| 0.0021 \| \| 97 \| 2 \| 2 \|  \| 0.0067 \| 0.9706 \| 0.0214 \| 0.0004 \| 0.0008 \| 0.0002 \| \| 98 \| 2 \| 2 \|  \| 0.0010 \| 0.9101 \| 0.0884 \| 0.0000 \| 0.0004 \| 0.0000 \| \| 99 \| 2 \| 2 \|  \| 0.0124 \| 0.9392 \| 0.0410 \| 0.0071 \| 0.0001 \| 0.0002 \| \| 100 \| 2 \| 2 \|  \| 0.0151 \| 0.6026 \| 0.3670 \| 0.0004 \| 0.0147 \| 0.0002 \| \| 101 \| 2 \| 2 \|  \| 0.0006 \| 0.9523 \| 0.0469 \| 0.0000 \| 0.0001 \| 0.0000 \| \| 102 \| 2 \| 2 \|  \| 0.0042 \| 0.7128 \| 0.2820 \| 0.0007 \| 0.0002 \| 0.0000 \| \| 103 \| 2 \| 2 \|  \| 0.0337 \| 0.4706 \| 0.4616 \| 0.0009 \| 0.0330 \| 0.0002 \| \| 104 \| 2 \| 2 \|  \| 0.0269 \| 0.9220 \| 0.0434 \| 0.0073 \| 0.0004 \| 0.0000 \| \| 105 \| 2 \| 2 \|  \| 0.0993 \| 0.5142 \| 0.0607 \| 0.3214 \| 0.0027 \| 0.0017 \| \| 106 \| 2 \| 2 \|  \| 0.0093 \| 0.8138 \| 0.1740 \| 0.0029 \| 0.0000 \| 0.0000 \| \| 107 \| 2 \| 2 \|  \| 0.0096 \| 0.9451 \| 0.0414 \| 0.0037 \| 0.0001 \| 0.0001 \| \| 108 \| 2 \| 2 \|  \| 0.0207 \| 0.5196 \| 0.4371 \| 0.0005 \| 0.0219 \| 0.0002 \| \| 109 \| 2 \| 2 \|  \| 0.0005 \| 0.9451 \| 0.0543 \| 0.0000 \| 0.0001 \| 0.0000 \| \| 110 \| 2 \| 2 \|  \| 0.0032 \| 0.7116 \| 0.2845 \| 0.0004 \| 0.0002 \| 0.0000 \| \| 111 \| 2 \| 2 \|  \| 0.0052 \| 0.8929 \| 0.0985 \| 0.0034 \| 0.0000 \| 0.0000 \| \| 112 \| 2 \| 2 \|  \| 0.0248 \| 0.9160 \| 0.0544 \| 0.0043 \| 0.0006 \| 0.0000 \| \| 113 \| 2 \| 2 \|  \| 0.0094 \| 0.9448 \| 0.0417 \| 0.0039 \| 0.0001 \| 0.0001 \| \| 114 \| 3 \| 2 \| * \| 0.0479 \| 0.7412 \| 0.0121 \| 0.1818 \| 0.0029 \| 0.0141 \| \| 115 \| 3 \| 3 \|  \| 0.0447 \| 0.0171 \| 0.8532 \| 0.0599 \| 0.0250 \| 0.0000 \| \| 116 \| 3 \| 3 \|  \| 0.1411 \| 0.1650 \| 0.6300 \| 0.0203 \| 0.0337 \| 0.0100 \| \| 117 \| 3 \| 6 \| * \| 0.0499 \| 0.0055 \| 0.1197 \| 0.0393 \| 0.2129 \| 0.5727 \| \| 118 \| 3 \| 5 \| * \| 0.0020 \| 0.0015 \| 0.4908 \| 0.0000 \| 0.5055 \| 0.0001 \| \| 119 \| 3 \| 3 \|  \| 0.1383 \| 0.1749 \| 0.5958 \| 0.0113 \| 0.0582 \| 0.0215 \| \| 120 \| 3 \| 5 \| * \| 0.0249 \| 0.0325 \| 0.0848 \| 0.0005 \| 0.6763 \| 0.1810 \| \| 121 \| 3 \| 3 \|  \| 0.1293 \| 0.2832 \| 0.5703 \| 0.0131 \| 0.0041 \| 0.0001 \| \| 122 \| 3 \| 2 \| * \| 0.0421 \| 0.7285 \| 0.0122 \| 0.2139 \| 0.0011 \| 0.0022 \| \| 123 \| 3 \| 5 \| * \| 0.0238 \| 0.0420 \| 0.1010 \| 0.0003 \| 0.6725 \| 0.1604 \| \| 124 \| 3 \| 3 \|  \| 0.1702 \| 0.1891 \| 0.5860 \| 0.0293 \| 0.0207 \| 0.0047 \| \| 125 \| 3 \| 3 \|  \| 0.1180 \| 0.0156 \| 0.3898 \| 0.1260 \| 0.1745 \| 0.1760 \| \| 126 \| 3 \| 3 \|  \| 0.0027 \| 0.0018 \| 0.5946 \| 0.0000 \| 0.4008 \| 0.0001 \| \| 127 \| 3 \| 3 \|  \| 0.1310 \| 0.2061 \| 0.6255 \| 0.0168 \| 0.0178 \| 0.0029 \| \| 128 \| 3 \| 5 \| * \| 0.0531 \| 0.0356 \| 0.0993 \| 0.0017 \| 0.6685 \| 0.1419 \| \| 129 \| 3 \| 3 \|  \| 0.1427 \| 0.2892 \| 0.5460 \| 0.0194 \| 0.0026 \| 0.0000 \| \| 130 \| 3 \| 3 \|  \| 0.1275 \| 0.0103 \| 0.2851 \| 0.1968 \| 0.1680 \| 0.2122 \| \| 131 \| 3 \| 2 \| * \| 0.0438 \| 0.8237 \| 0.0159 \| 0.0983 \| 0.0035 \| 0.0149 \| \| 132 \| 3 \| 3 \|  \| 0.0442 \| 0.0185 \| 0.8601 \| 0.0330 \| 0.0441 \| 0.0001 \| \| 133 \| 3 \| 6 \| * \| 0.0152 \| 0.0015 \| 0.0236 \| 0.0062 \| 0.1489 \| 0.8045 \| \| 134 \| 3 \| 5 \| * \| 0.0014 \| 0.0011 \| 0.3130 \| 0.0000 \| 0.6843 \| 0.0002 \| \| 135 \| 3 \| 3 \|  \| 0.0565 \| 0.0204 \| 0.8159 \| 0.0914 \| 0.0158 \| 0.0000 \| \| 136 \| 3 \| 5 \| * \| 0.0137 \| 0.0347 \| 0.0576 \| 0.0001 \| 0.6261 \| 0.2678 \| \| 137 \| 3 \| 3 \|  \| 0.1361 \| 0.3335 \| 0.5151 \| 0.0079 \| 0.0072 \| 0.0002 \| \| 138 \| 3 \| 3 \|  \| 0.0398 \| 0.0269 \| 0.8730 \| 0.0476 \| 0.0127 \| 0.0000 \| \| 139 \| 3 \| 3 \|  \| 0.0982 \| 0.3167 \| 0.5748 \| 0.0068 \| 0.0035 \| 0.0001 \| \| 140 \| 3 \| 2 \| * \| 0.0315 \| 0.8326 \| 0.0137 \| 0.1199 \| 0.0009 \| 0.0013 \| \| 141 \| 3 \| 3 \|  \| 0.1052 \| 0.0079 \| 0.4910 \| 0.0010 \| 0.3948 \| 0.0001 \| \| 142 \| 3 \| 3 \|  \| 0.0191 \| 0.0640 \| 0.8864 \| 0.0162 \| 0.0143 \| 0.0000 \| \| 143 \| 3 \| 3 \|  \| 0.0557 \| 0.0321 \| 0.8117 \| 0.0015 \| 0.0989 \| 0.0001 \| \| 144 \| 3 \| 2 \| * \| 0.0332 \| 0.7825 \| 0.1779 \| 0.0060 \| 0.0004 \| 0.0000 \| \| 145 \| 3 \| 3 \|  \| 0.0748 \| 0.0230 \| 0.7772 \| 0.0019 \| 0.1230 \| 0.0001 \| \| 146 \| 3 \| 3 \|  \| 0.0033 \| 0.1540 \| 0.8386 \| 0.0024 \| 0.0016 \| 0.0000 \| \| 147 \| 3 \| 2 \| * \| 0.0077 \| 0.9153 \| 0.0745 \| 0.0024 \| 0.0000 \| 0.0000 \| \| 148 \| 3 \| 3 \|  \| 0.0313 \| 0.0472 \| 0.8707 \| 0.0304 \| 0.0204 \| 0.0000 \| \| 149 \| 3 \| 2 \| * \| 0.1065 \| 0.5972 \| 0.2770 \| 0.0181 \| 0.0012 \| 0.0000 \| \| 150 \| 3 \| 2 \| * \| 0.0482 \| 0.7393 \| 0.2066 \| 0.0055 \| 0.0004 \| 0.0001 \| \| 151 \| 3 \| 3 \|  \| 0.0503 \| 0.0490 \| 0.8138 \| 0.0310 \| 0.0555 \| 0.0005 \| \| 152 \| 3 \| 3 \|  \| 0.0403 \| 0.0154 \| 0.7720 \| 0.0010 \| 0.1710 \| 0.0004 \| \| 153 \| 3 \| 2 \| * \| 0.1437 \| 0.6313 \| 0.2067 \| 0.0153 \| 0.0020 \| 0.0009 \| \| 154 \| 3 \| 3 \|  \| 0.0510 \| 0.0156 \| 0.7607 \| 0.0016 \| 0.1701 \| 0.0009 \| \| 155 \| 3 \| 2 \| * \| 0.1650 \| 0.4136 \| 0.4070 \| 0.0121 \| 0.0021 \| 0.0002 \| \| 156 \| 3 \| 3 \|  \| 0.0448 \| 0.0397 \| 0.8397 \| 0.0211 \| 0.0545 \| 0.0003 \| \| 157 \| 4 \| 4 \|  \| 0.1643 \| 0.0531 \| 0.0035 \| 0.5654 \| 0.1894 \| 0.0243 \| \| 158 \| 4 \| 4 \|  \| 0.1825 \| 0.0623 \| 0.0361 \| 0.7031 \| 0.0153 \| 0.0007 \| \| 159 \| 4 \| 4 \|  \| 0.0841 \| 0.0092 \| 0.0149 \| 0.6865 \| 0.2041 \| 0.0012 \| \| 160 \| 4 \| 4 \|  \| 0.1439 \| 0.0151 \| 0.0248 \| 0.7976 \| 0.0181 \| 0.0005 \| \| 161 \| 4 \| 4 \|  \| 0.1430 \| 0.0366 \| 0.0098 \| 0.6479 \| 0.1590 \| 0.0038 \| \| 162 \| 4 \| 4 \|  \| 0.1722 \| 0.0454 \| 0.0407 \| 0.7236 \| 0.0175 \| 0.0006 \| \| 163 \| 4 \| 4 \|  \| 0.1973 \| 0.1149 \| 0.0301 \| 0.6473 \| 0.0097 \| 0.0007 \| \| 164 \| 4 \| 4 \|  \| 0.1019 \| 0.0109 \| 0.0092 \| 0.5169 \| 0.3596 \| 0.0016 \| \| 165 \| 4 \| 4 \|  \| 0.1746 \| 0.0694 \| 0.0342 \| 0.7095 \| 0.0117 \| 0.0006 \| \| 166 \| 4 \| 4 \|  \| 0.1173 \| 0.0233 \| 0.0080 \| 0.6678 \| 0.1791 \| 0.0045 \| \| 167 \| 4 \| 4 \|  \| 0.1191 \| 0.0566 \| 0.0020 \| 0.8186 \| 0.0019 \| 0.0019 \| \| 168 \| 4 \| 4 \|  \| 0.3547 \| 0.0639 \| 0.0027 \| 0.5706 \| 0.0040 \| 0.0042 \| \| 169 \| 4 \| 4 \|  \| 0.1859 \| 0.0684 \| 0.0028 \| 0.7368 \| 0.0033 \| 0.0028 \| \| 170 \| 4 \| 4 \|  \| 0.2140 \| 0.0144 \| 0.0019 \| 0.7619 \| 0.0051 \| 0.0029 \| \| 171 \| 4 \| 4 \|  \| 0.3224 \| 0.0742 \| 0.0024 \| 0.5936 \| 0.0033 \| 0.0041 \| \| 172 \| 4 \| 4 \|  \| 0.1251 \| 0.0201 \| 0.0015 \| 0.8463 \| 0.0068 \| 0.0002 \| \| 173 \| 4 \| 4 \|  \| 0.2711 \| 0.0593 \| 0.0020 \| 0.6611 \| 0.0027 \| 0.0038 \| \| 174 \| 4 \| 4 \|  \| 0.0705 \| 0.0387 \| 0.0012 \| 0.8875 \| 0.0010 \| 0.0010 \| \| 175 \| 4 \| 4 \|  \| 0.0938 \| 0.0065 \| 0.0007 \| 0.8959 \| 0.0018 \| 0.0014 \| \| 176 \| 4 \| 4 \|  \| 0.2242 \| 0.0668 \| 0.0019 \| 0.7021 \| 0.0021 \| 0.0030 \| \| 177 \| 4 \| 4 \|  \| 0.0712 \| 0.0134 \| 0.0009 \| 0.9107 \| 0.0037 \| 0.0001 \| \| 178 \| 4 \| 4 \|  \| 0.1681 \| 0.0104 \| 0.0012 \| 0.8137 \| 0.0037 \| 0.0029 \| \| 179 \| 4 \| 4 \|  \| 0.2522 \| 0.0102 \| 0.0013 \| 0.7174 \| 0.0087 \| 0.0102 \| \| 180 \| 4 \| 4 \|  \| 0.4437 \| 0.0895 \| 0.0033 \| 0.4524 \| 0.0055 \| 0.0056 \| \| 181 \| 4 \| 4 \|  \| 0.2038 \| 0.0253 \| 0.0024 \| 0.7563 \| 0.0119 \| 0.0004 \| \| 182 \| 4 \| 4 \|  \| 0.0982 \| 0.0525 \| 0.0016 \| 0.8443 \| 0.0015 \| 0.0019 \| \| 183 \| 4 \| 4 \|  \| 0.1656 \| 0.0245 \| 0.0016 \| 0.7984 \| 0.0095 \| 0.0003 \| \| 184 \| 4 \| 4 \|  \| 0.1499 \| 0.0590 \| 0.0021 \| 0.7835 \| 0.0028 \| 0.0028 \| \| 185 \| 4 \| 4 \|  \| 0.0996 \| 0.0170 \| 0.0012 \| 0.8768 \| 0.0052 \| 0.0002 \| \| 186 \| 4 \| 4 \|  \| 0.1231 \| 0.0059 \| 0.0007 \| 0.8653 \| 0.0024 \| 0.0027 \| \| 187 \| 4 \| 4 \|  \| 0.3697 \| 0.0058 \| 0.0030 \| 0.6194 \| 0.0020 \| 0.0002 \| \| 188 \| 4 \| 4 \|  \| 0.0675 \| 0.0020 \| 0.0024 \| 0.8831 \| 0.0341 \| 0.0109 \| \| 189 \| 4 \| 4 \|  \| 0.1600 \| 0.2411 \| 0.0305 \| 0.4052 \| 0.0509 \| 0.1124 \| \| 190 \| 4 \| 1 \| * \| 0.4271 \| 0.1236 \| 0.2485 \| 0.1908 \| 0.0099 \| 0.0001 \| \| 191 \| 4 \| 1 \| * \| 0.5381 \| 0.0527 \| 0.0048 \| 0.3962 \| 0.0031 \| 0.0052 \| \| 192 \| 4 \| 1 \| * \| 0.4168 \| 0.1535 \| 0.2868 \| 0.1312 \| 0.0115 \| 0.0002 \| \| 193 \| 4 \| 4 \|  \| 0.4154 \| 0.0087 \| 0.0040 \| 0.5675 \| 0.0040 \| 0.0004 \| \| 194 \| 4 \| 1 \| * \| 0.3636 \| 0.2397 \| 0.3190 \| 0.0550 \| 0.0219 \| 0.0007 \| \| 195 \| 4 \| 2 \| * \| 0.1243 \| 0.2637 \| 0.0293 \| 0.2577 \| 0.0722 \| 0.2529 \| \| 196 \| 4 \| 4 \|  \| 0.3398 \| 0.0079 \| 0.0044 \| 0.6469 \| 0.0009 \| 0.0000 \| \| 197 \| 4 \| 4 \|  \| 0.0669 \| 0.0030 \| 0.0032 \| 0.8954 \| 0.0272 \| 0.0044 \| \| 198 \| 4 \| 4 \|  \| 0.4681 \| 0.0393 \| 0.0034 \| 0.4865 \| 0.0013 \| 0.0014 \| \| 199 \| 4 \| 4 \|  \| 0.0809 \| 0.0125 \| 0.0114 \| 0.8659 \| 0.0281 \| 0.0012 \| \| 200 \| 4 \| 1 \| * \| 0.5094 \| 0.0135 \| 0.0053 \| 0.4646 \| 0.0057 \| 0.0015 \| \| 201 \| 4 \| 4 \|  \| 0.3914 \| 0.0183 \| 0.0031 \| 0.5858 \| 0.0012 \| 0.0002 \| \| 202 \| 4 \| 4 \|  \| 0.0988 \| 0.0088 \| 0.0085 \| 0.6624 \| 0.1682 \| 0.0534 \| \| 203 \| 4 \| 1 \| * \| 0.4089 \| 0.1828 \| 0.3016 \| 0.0910 \| 0.0154 \| 0.0004 \| \| 204 \| 4 \| 6 \| * \| 0.0567 \| 0.2181 \| 0.0188 \| 0.0595 \| 0.0942 \| 0.5527 \| \| 205 \| 4 \| 4 \|  \| 0.1322 \| 0.3004 \| 0.0443 \| 0.4583 \| 0.0338 \| 0.0308 \| \| 206 \| 4 \| 4 \|  \| 0.3811 \| 0.0271 \| 0.0036 \| 0.5872 \| 0.0009 \| 0.0002 \| \| 207 \| 4 \| 4 \|  \| 0.2848 \| 0.0031 \| 0.0021 \| 0.7089 \| 0.0010 \| 0.0001 \| \| 208 \| 4 \| 4 \|  \| 0.0877 \| 0.0061 \| 0.0065 \| 0.7850 \| 0.0961 \| 0.0186 \| \| 209 \| 4 \| 1 \| * \| 0.4829 \| 0.0373 \| 0.0042 \| 0.4716 \| 0.0024 \| 0.0015 \| \| 210 \| 4 \| 1 \| * \| 0.3926 \| 0.1862 \| 0.2707 \| 0.1411 \| 0.0094 \| 0.0001 \| \| 211 \| 4 \| 6 \| * \| 0.0925 \| 0.2597 \| 0.0267 \| 0.1417 \| 0.0917 \| 0.3878 \| \| 212 \| 5 \| 5 \|  \| 0.0775 \| 0.0012 \| 0.0041 \| 0.0013 \| 0.8443 \| 0.0715 \| \| 213 \| 5 \| 5 \|  \| 0.1896 \| 0.2498 \| 0.0602 \| 0.0494 \| 0.4465 \| 0.0044 \| \| 214 \| 5 \| 5 \|  \| 0.0613 \| 0.0044 \| 0.0216 \| 0.0196 \| 0.8597 \| 0.0333 \| \| 215 \| 5 \| 5 \|  \| 0.1888 \| 0.3460 \| 0.0620 \| 0.0494 \| 0.3525 \| 0.0014 \| \| 216 \| 5 \| 5 \|  \| 0.0422 \| 0.0005 \| 0.0024 \| 0.0008 \| 0.7360 \| 0.2181 \| \| 217 \| 5 \| 5 \|  \| 0.0630 \| 0.0022 \| 0.0239 \| 0.0306 \| 0.8397 \| 0.0407 \| \| 218 \| 5 \| 5 \|  \| 0.0356 \| 0.0003 \| 0.0022 \| 0.0008 \| 0.7726 \| 0.1886 \| \| 219 \| 5 \| 5 \|  \| 0.1534 \| 0.0920 \| 0.0578 \| 0.0537 \| 0.6404 \| 0.0028 \| \| 220 \| 5 \| 5 \|  \| 0.0724 \| 0.0047 \| 0.0140 \| 0.0249 \| 0.8297 \| 0.0543 \| \| 221 \| 5 \| 5 \|  \| 0.0516 \| 0.0005 \| 0.0021 \| 0.0011 \| 0.6816 \| 0.2631 \| \| 222 \| 5 \| 5 \|  \| 0.0957 \| 0.0048 \| 0.0144 \| 0.0411 \| 0.7920 \| 0.0520 \| \| 223 \| 5 \| 5 \|  \| 0.0345 \| 0.0003 \| 0.0037 \| 0.0008 \| 0.7804 \| 0.1804 \| \| 224 \| 5 \| 5 \|  \| 0.2335 \| 0.2647 \| 0.0572 \| 0.0791 \| 0.3611 \| 0.0044 \| \| 225 \| 5 \| 5 \|  \| 0.1187 \| 0.0091 \| 0.0190 \| 0.0395 \| 0.7800 \| 0.0336 \| \| 226 \| 5 \| 5 \|  \| 0.2029 \| 0.1858 \| 0.0623 \| 0.0612 \| 0.4833 \| 0.0045 \| \| 227 \| 5 \| 3 \| * \| 0.2002 \| 0.0145 \| 0.5146 \| 0.0297 \| 0.2369 \| 0.0041 \| \| 228 \| 5 \| 5 \|  \| 0.0329 \| 0.0384 \| 0.0061 \| 0.0088 \| 0.9068 \| 0.0070 \| \| 229 \| 5 \| 3 \| * \| 0.1563 \| 0.0115 \| 0.5374 \| 0.0216 \| 0.2682 \| 0.0050 \| \| 230 \| 5 \| 3 \| * \| 0.2174 \| 0.0194 \| 0.4852 \| 0.0310 \| 0.2397 \| 0.0074 \| \| 231 \| 5 \| 5 \|  \| 0.0901 \| 0.0453 \| 0.0205 \| 0.3288 \| 0.4913 \| 0.0240 \| \| 232 \| 5 \| 5 \|  \| 0.0121 \| 0.0009 \| 0.0072 \| 0.0044 \| 0.8918 \| 0.0835 \| \| 233 \| 5 \| 5 \|  \| 0.0310 \| 0.0015 \| 0.0072 \| 0.0120 \| 0.7796 \| 0.1688 \| \| 234 \| 5 \| 5 \|  \| 0.0179 \| 0.0054 \| 0.0033 \| 0.0062 \| 0.9589 \| 0.0082 \| \| 235 \| 5 \| 5 \|  \| 0.0326 \| 0.0177 \| 0.0040 \| 0.0102 \| 0.9248 \| 0.0107 \| \| 236 \| 5 \| 4 \| * \| 0.1089 \| 0.0530 \| 0.0193 \| 0.4310 \| 0.3607 \| 0.0271 \| \| 237 \| 5 \| 3 \| * \| 0.1054 \| 0.0465 \| 0.4980 \| 0.0255 \| 0.3245 \| 0.0001 \| \| 238 \| 5 \| 5 \|  \| 0.0213 \| 0.0010 \| 0.0062 \| 0.0063 \| 0.8722 \| 0.0931 \| \| 239 \| 5 \| 3 \| * \| 0.1597 \| 0.0082 \| 0.4944 \| 0.0221 \| 0.3102 \| 0.0053 \| \| 240 \| 5 \| 1 \| * \| 0.3316 \| 0.0386 \| 0.2723 \| 0.0347 \| 0.2980 \| 0.0249 \| \| 241 \| 5 \| 3 \| * \| 0.1234 \| 0.0862 \| 0.5582 \| 0.0201 \| 0.2121 \| 0.0000 \| \| 242 \| 5 \| 5 \|  \| 0.0707 \| 0.0113 \| 0.0152 \| 0.3176 \| 0.5605 \| 0.0248 \| \| 243 \| 5 \| 5 \|  \| 0.0427 \| 0.0035 \| 0.0071 \| 0.0127 \| 0.6537 \| 0.2803 \| \| 244 \| 5 \| 5 \|  \| 0.0114 \| 0.0003 \| 0.0032 \| 0.0030 \| 0.8157 \| 0.1663 \| \| 245 \| 5 \| 3 \| * \| 0.1905 \| 0.1471 \| 0.4809 \| 0.0566 \| 0.1248 \| 0.0000 \| \| 246 \| 5 \| 5 \|  \| 0.0232 \| 0.0131 \| 0.0045 \| 0.0071 \| 0.9444 \| 0.0077 \| \| 247 \| 5 \| 4 \| * \| 0.1191 \| 0.0943 \| 0.0197 \| 0.3988 \| 0.3362 \| 0.0319 \| \| 248 \| 5 \| 5 \|  \| 0.1354 \| 0.0348 \| 0.3749 \| 0.0286 \| 0.4261 \| 0.0001 \| \| 249 \| 5 \| 3 \| * \| 0.1618 \| 0.1327 \| 0.5021 \| 0.0426 \| 0.1608 \| 0.0000 \| \| 250 \| 5 \| 5 \|  \| 0.0773 \| 0.0317 \| 0.0198 \| 0.3471 \| 0.5045 \| 0.0195 \| \| 251 \| 5 \| 5 \|  \| 0.0513 \| 0.0579 \| 0.0084 \| 0.0143 \| 0.8575 \| 0.0106 \| \| 252 \| 5 \| 3 \| * \| 0.0012 \| 0.0022 \| 0.5080 \| 0.0000 \| 0.4860 \| 0.0026 \| \| 253 \| 5 \| 5 \|  \| 0.1316 \| 0.1164 \| 0.0286 \| 0.0263 \| 0.6912 \| 0.0059 \| \| 254 \| 5 \| 5 \|  \| 0.0193 \| 0.0109 \| 0.0541 \| 0.0009 \| 0.8885 \| 0.0262 \| \| 255 \| 5 \| 5 \|  \| 0.1083 \| 0.0016 \| 0.0002 \| 0.1086 \| 0.7787 \| 0.0025 \| \| 256 \| 5 \| 5 \|  \| 0.0254 \| 0.0190 \| 0.0654 \| 0.0012 \| 0.8690 \| 0.0200 \| \| 257 \| 5 \| 5 \|  \| 0.0842 \| 0.0277 \| 0.0224 \| 0.0197 \| 0.8444 \| 0.0016 \| \| 258 \| 5 \| 3 \| * \| 0.0008 \| 0.0056 \| 0.7897 \| 0.0000 \| 0.2027 \| 0.0012 \| \| 259 \| 5 \| 5 \|  \| 0.0116 \| 0.0034 \| 0.0226 \| 0.0005 \| 0.9494 \| 0.0124 \| \| 260 \| 5 \| 5 \|  \| 0.0998 \| 0.0014 \| 0.0003 \| 0.0831 \| 0.8135 \| 0.0018 \| \| 261 \| 5 \| 5 \|  \| 0.0006 \| 0.0007 \| 0.3258 \| 0.0000 \| 0.6697 \| 0.0032 \| \| 262 \| 5 \| 5 \|  \| 0.0112 \| 0.0024 \| 0.0191 \| 0.0005 \| 0.9495 \| 0.0172 \| \| 263 \| 5 \| 5 \|  \| 0.0679 \| 0.0007 \| 0.0002 \| 0.0631 \| 0.8652 \| 0.0030 \| \| 264 \| 5 \| 5 \|  \| 0.1318 \| 0.0026 \| 0.0003 \| 0.1262 \| 0.7354 \| 0.0038 \| \| 265 \| 5 \| 3 \| * \| 0.0011 \| 0.0033 \| 0.6499 \| 0.0000 \| 0.3444 \| 0.0012 \| \| 266 \| 5 \| 5 \|  \| 0.0383 \| 0.0106 \| 0.0120 \| 0.0074 \| 0.9293 \| 0.0024 \| \| 267 \| 5 \| 5 \|  \| 0.0070 \| 0.0016 \| 0.0132 \| 0.0003 \| 0.9549 \| 0.0229 \| \| 268 \| 5 \| 5 \|  \| 0.0502 \| 0.0158 \| 0.0144 \| 0.0085 \| 0.9097 \| 0.0014 \| \| 269 \| 5 \| 5 \|  \| 0.0009 \| 0.0012 \| 0.4847 \| 0.0000 \| 0.5122 \| 0.0011 \| \| 270 \| 5 \| 5 \|  \| 0.1500 \| 0.0049 \| 0.0003 \| 0.1271 \| 0.7144 \| 0.0034 \| \| 271 \| 5 \| 5 \|  \| 0.1211 \| 0.0684 \| 0.0271 \| 0.0247 \| 0.7546 \| 0.0041 \| \| 272 \| 6 \| 5 \| * \| 0.0142 \| 0.0021 \| 0.0069 \| 0.0001 \| 0.7120 \| 0.2648 \| \| 273 \| 6 \| 6 \|  \| 0.1103 \| 0.0042 \| 0.0002 \| 0.0520 \| 0.1396 \| 0.6938 \| \| 274 \| 6 \| 5 \| * \| 0.0103 \| 0.0016 \| 0.0058 \| 0.0000 \| 0.7178 \| 0.2644 \| \| 275 \| 6 \| 5 \| * \| 0.0251 \| 0.0020 \| 0.0009 \| 0.0071 \| 0.9092 \| 0.0557 \| \| 276 \| 6 \| 5 \| * \| 0.0892 \| 0.0829 \| 0.0209 \| 0.0940 \| 0.4377 \| 0.2754 \| \| 277 \| 6 \| 5 \| * \| 0.0605 \| 0.1022 \| 0.0268 \| 0.0528 \| 0.5632 \| 0.1945 \| \| 278 \| 6 \| 5 \| * \| 0.0297 \| 0.0070 \| 0.0023 \| 0.0061 \| 0.9307 \| 0.0242 \| \| 279 \| 6 \| 6 \|  \| 0.0381 \| 0.0010 \| 0.0001 \| 0.0193 \| 0.0957 \| 0.8458 \| \| 280 \| 6 \| 6 \|  \| 0.0142 \| 0.0039 \| 0.0283 \| 0.0245 \| 0.4281 \| 0.5010 \| \| 281 \| 6 \| 5 \| * \| 0.0151 \| 0.0032 \| 0.0073 \| 0.0001 \| 0.6706 \| 0.3037 \| \| 282 \| 6 \| 6 \|  \| 0.0907 \| 0.0021 \| 0.0001 \| 0.0409 \| 0.1370 \| 0.7293 \| \| 283 \| 6 \| 5 \| * \| 0.0169 \| 0.0013 \| 0.0006 \| 0.0044 \| 0.8946 \| 0.0821 \| \| 284 \| 6 \| 6 \|  \| 0.0092 \| 0.0026 \| 0.0218 \| 0.0167 \| 0.4465 \| 0.5031 \| \| 285 \| 6 \| 5 \| * \| 0.0076 \| 0.0006 \| 0.0072 \| 0.0000 \| 0.8766 \| 0.1080 \| \| 286 \| 6 \| 6 \|  \| 0.0205 \| 0.0120 \| 0.0406 \| 0.0288 \| 0.3388 \| 0.5593 \| \| 287 \| 6 \| 5 \| * \| 0.0523 \| 0.0728 \| 0.0301 \| 0.0487 \| 0.6090 \| 0.1872 \| \| 288 \| 6 \| 5 \| * \| 0.0117 \| 0.0006 \| 0.0009 \| 0.0035 \| 0.9604 \| 0.0228 \| \| 289 \| 6 \| 5 \| * \| 0.0906 \| 0.2447 \| 0.0211 \| 0.0725 \| 0.3501 \| 0.2211 \| \| 290 \| 6 \| 5 \| * \| 0.0410 \| 0.0496 \| 0.0515 \| 0.0415 \| 0.7328 \| 0.0836 \| \| 291 \| 6 \| 5 \| * \| 0.0498 \| 0.0081 \| 0.0020 \| 0.0106 \| 0.8919 \| 0.0376 \| \| 292 \| 6 \| 5 \| * \| 0.0107 \| 0.0020 \| 0.0373 \| 0.0214 \| 0.5633 \| 0.3653 \| \| 293 \| 6 \| 5 \| * \| 0.0247 \| 0.0055 \| 0.0079 \| 0.0001 \| 0.6862 \| 0.2756 \| \| 294 \| 6 \| 6 \|  \| 0.0168 \| 0.0093 \| 0.0492 \| 0.0252 \| 0.4397 \| 0.4597 \| \| 295 \| 6 \| 6 \|  \| 0.0626 \| 0.0021 \| 0.0001 \| 0.0283 \| 0.1265 \| 0.7804 \| \| 296 \| 6 \| 6 \|  \| 0.1555 \| 0.0069 \| 0.0002 \| 0.0717 \| 0.1409 \| 0.6246 \| \| 297 \| 6 \| 6 \|  \| 0.0121 \| 0.0003 \| 0.0002 \| 0.0003 \| 0.0001 \| 0.9869 \| \| 298 \| 6 \| 2 \| * \| 0.1439 \| 0.2728 \| 0.0146 \| 0.2340 \| 0.1170 \| 0.2177 \| \| 299 \| 6 \| 6 \|  \| 0.0101 \| 0.0004 \| 0.0001 \| 0.0002 \| 0.0001 \| 0.9891 \| \| 300 \| 6 \| 6 \|  \| 0.1255 \| 0.1804 \| 0.0125 \| 0.1967 \| 0.1195 \| 0.3653 \| \| 301 \| 6 \| 2 \| * \| 0.1203 \| 0.3854 \| 0.0154 \| 0.2405 \| 0.0841 \| 0.1544 \| \| 302 \| 6 \| 6 \|  \| 0.0130 \| 0.0002 \| 0.0005 \| 0.0005 \| 0.0002 \| 0.9856 \| \| 303 \| 6 \| 6 \|  \| 0.0118 \| 0.0003 \| 0.0002 \| 0.0003 \| 0.0001 \| 0.9872 \| \| 304 \| 6 \| 6 \|  \| 0.1149 \| 0.0981 \| 0.0151 \| 0.1961 \| 0.1886 \| 0.3872 \| \| 305 \| 6 \| 6 \|  \| 0.0424 \| 0.0021 \| 0.0009 \| 0.0014 \| 0.0002 \| 0.9530 \| \| 306 \| 6 \| 6 \|  \| 0.0082 \| 0.0002 \| 0.0002 \| 0.0002 \| 0.0001 \| 0.9912 \| \| 307 \| 6 \| 6 \|  \| 0.1193 \| 0.1558 \| 0.0143 \| 0.2010 \| 0.1236 \| 0.3860 \| \| 308 \| 6 \| 6 \|  \| 0.0457 \| 0.0025 \| 0.0010 \| 0.0015 \| 0.0003 \| 0.9491 \| \| 309 \| 6 \| 6 \|  \| 0.1185 \| 0.1445 \| 0.0071 \| 0.1662 \| 0.1652 \| 0.3985 \| \| 310 \| 6 \| 6 \|  \| 0.0070 \| 0.0001 \| 0.0002 \| 0.0002 \| 0.0001 \| 0.9924 \| \| 311 \| 6 \| 6 \|  \| 0.0000 \| 0.0000 \| 0.0000 \| 0.0000 \| 0.0000 \| 0.9999 \| \| 312 \| 6 \| 6 \|  \| 0.0000 \| 0.0000 \| 0.0000 \| 0.0000 \| 0.0000 \| 0.9999 \| \| 313 \| 6 \| 6 \|  \| 0.0000 \| 0.0000 \| 0.0000 \| 0.0000 \| 0.0000 \| 0.9999 \| \| 314 \| 6 \| 6 \|  \| 0.0001 \| 0.0001 \| 0.0000 \| 0.0000 \| 0.0000 \| 0.9998 \| \| 315 \| 6 \| 6 \|  \| 0.0000 \| 0.0001 \| 0.0001 \| 0.0000 \| 0.0000 \| 0.9997 \| \| 316 \| 6 \| 6 \|  \| 0.0000 \| 0.0000 \| 0.0000 \| 0.0000 \| 0.0000 \| 1.0000 \| \| 317 \| 6 \| 6 \|  \| 0.0001 \| 0.0000 \| 0.0000 \| 0.0001 \| 0.0000 \| 0.9998 \| \| 318 \| 6 \| 6 \|  \| 0.0000 \| 0.0000 \| 0.0000 \| 0.0000 \| 0.0000 \| 0.9999 \| \| 319 \| 6 \| 6 \|  \| 0.0001 \| 0.0003 \| 0.0000 \| 0.0000 \| 0.0000 \| 0.9995 \| \| 320 \| 6 \| 6 \|  \| 0.0000 \| 0.0001 \| 0.0001 \| 0.0000 \| 0.0000 \| 0.9997 \| \| 321 \| 6 \| 6 \|  \| 0.0001 \| 0.0001 \| 0.0000 \| 0.0000 \| 0.0000 \| 0.9998 \| \| 322 \| 6 \| 6 \|  \| 0.0080 \| 0.0003 \| 0.0002 \| 0.0110 \| 0.0000 \| 0.9805 \| \| 323 \| 6 \| 6 \|  \| 0.0023 \| 0.0023 \| 0.0006 \| 0.0013 \| 0.0002 \| 0.9933 \| \| \| --- \| --- \| --- \| --- \| --- \| --- \| --- \| --- \| --- \| --- \| --- \| --- \| --- \| --- \| --- \| --- \| --- \| --- \| --- \| --- \| --- \| --- \| --- \| --- \| --- \| --- \| --- \| --- \| --- \| --- \| --- \| --- \| --- \| --- \| --- \| --- \| --- \| --- \| --- \| --- \| --- \| --- \| --- \| --- \| --- \| --- \| --- \| --- \| --- \| --- \| --- \| --- \| --- \| --- \| --- \| --- \| --- \| --- \| --- \| --- \| --- \| --- \| --- \| --- \| --- \| --- \| --- \| --- \| --- \| --- \| --- \| --- \| --- \| --- \| --- \| --- \| --- \| --- \| --- \| --- \| --- \| --- \| --- \| --- \| --- \| --- \| --- \| --- \| --- \| --- \| --- \| --- \| --- \| --- \| --- \| --- \| --- \| --- \| --- \| --- \| --- \| --- \| --- \| --- \| --- \| --- \| --- \| --- \| --- \| --- \| --- \| --- \| --- \| --- \| --- \| --- \| --- \| --- \| --- \| --- \| --- \| --- \| --- \| --- \| --- \| --- \| --- \| --- \| --- \| --- \| --- \| --- \| --- \| --- \| --- \| --- \| --- \| --- \| --- \| --- \| --- \| --- \| --- \| --- \| --- \| --- \| --- \| --- \| --- \| --- \| --- \| --- \| --- \| --- \| --- \| --- \| --- \| --- \| --- \| --- \| --- \| --- \| --- \| --- \| --- \| --- \| --- \| --- \| --- \| --- \| --- \| --- \| --- \| --- \| --- \| --- \| --- \| --- \| --- \| --- \| --- \| --- \| --- \| --- \| --- \| --- \| --- \| --- \| --- \| --- \| --- \| --- \| --- \| --- \| --- \| --- \| --- \| --- \| --- \| --- \| --- \| --- \| --- \| --- \| --- \| --- \| --- \| --- \| --- \| --- \| --- \| --- \| --- \| --- \| --- \| --- \| --- \| --- \| --- \| --- \| --- \| --- \| --- \| --- \| --- \| --- \| --- \| --- \| --- \| --- \| --- \| --- \| --- \| --- \| --- \| --- \| --- \| --- \| --- \| --- \| --- \| --- \| --- \| --- \| --- \| --- \| --- \| --- \| --- \| --- \| --- \| --- \| --- \| --- \| --- \| --- \| --- \| --- \| --- \| --- \| --- \| --- \| --- \| --- \| --- \| --- \| --- \| --- \| --- \| --- \| --- \| --- \| --- \| --- \| --- \| --- \| --- \| --- \| --- \| --- \| --- \| --- \| --- \| --- \| --- \| --- \| --- \| --- \| --- \| --- \| --- \| --- \| --- \| --- \| --- \| --- \| --- \| --- \| --- \| --- \| --- \| --- \| --- \| --- \| --- \| --- \| --- \| --- \| --- \| --- \| --- \| --- \| --- \| --- \| --- \| --- \| --- \| --- \| --- \| --- \| --- \| --- \| --- \| --- \| --- \| --- \| --- \| --- \| --- \| --- \| --- \| --- \| --- \| --- \| --- \| --- \| --- \| --- \| --- \| --- \| --- \| --- \| --- \| --- \| --- \| --- \| --- \| --- \| --- \| --- \| --- \| --- \| --- \| --- \| --- \| --- \| --- \| --- \| --- \| --- \| --- \| --- \| --- \| --- \| --- \| --- \| --- \| --- \| --- \| --- \| --- \| --- \| --- \| --- \| --- \| --- \| --- \| --- \| --- \| --- \| --- \| --- \| --- \| --- \| --- \| --- \| --- \| --- \| --- \| --- \| --- \| --- \| --- \| --- \| --- \| --- \| --- \| --- \| --- \| --- \| --- \| --- \| --- \| --- \| --- \| --- \| --- \| --- \| --- \| --- \| --- \| --- \| --- \| --- \| --- \| --- \| --- \| --- \| --- \| --- \| --- \| --- \| --- \| --- \| --- \| --- \| --- \| --- \| --- \| --- \| --- \| --- \| --- \| --- \| --- \| --- \| --- \| --- \| --- \| --- \| --- \| --- \| --- \| --- \| --- \| --- \| --- \| --- \| --- \| --- \| --- \| --- \| --- \| --- \| --- \| --- \| --- \| --- \| --- \| --- \| --- \| --- \| --- \| --- \| --- \| --- \| --- \| --- \| --- \| --- \| --- \| --- \| --- \| --- \| --- \| --- \| --- \| --- \| --- \| --- \| --- \| --- \| --- \| --- \| --- \| --- \| --- \| --- \| --- \| --- \| --- \| --- \| --- \| --- \| --- \| --- \| --- \| --- \| --- \| --- \| --- \| --- \| --- \| --- \| --- \| --- \| --- \| --- \| --- \| --- \| --- \| --- \| --- \| --- \| --- \| --- \| --- \| --- \| --- \| --- \| --- \| --- \| --- \| --- \| --- \| --- \| --- \| --- \| --- \| --- \| --- \| --- \| --- \| --- \| --- \| --- \| --- \| --- \| --- \| --- \| --- \| --- \| --- \| --- \| --- \| --- \| --- \| --- \| --- \| --- \| --- \| --- \| --- \| --- \| --- \| --- \| --- \| --- \| --- \| --- \| --- \| --- \| --- \| --- \| --- \| --- \| --- \| --- \| --- \| --- \| --- \| --- \| --- \| --- \| --- \| --- \| --- \| --- \| --- \| --- \| --- \| --- \| --- \| --- \| --- \| --- \| --- \| --- \| --- \| --- \| --- \| --- \| --- \| --- \| --- \| --- \| --- \| --- \| --- \| --- \| --- \| --- \| --- \| --- \| --- \| --- \| --- \| --- \| --- \| --- \| --- \| --- \| --- \| --- \| --- \| --- \| --- \| --- \| --- \| --- \| --- \| --- \| --- \| --- \| --- \| --- \| --- \| --- \| --- \| --- \| --- \| --- \| --- \| --- \| --- \| --- \| --- \| --- \| --- \| --- \| --- \| --- \| --- \| --- \| --- \| --- \| --- \| --- \| --- \| --- \| --- \| --- \| --- \| --- \| --- \| --- \| --- \| --- \| --- \| --- \| --- \| --- \| --- \| --- \| --- \| --- \| --- \| --- \| --- \| --- \| --- \| --- \| --- \| --- \| --- \| --- \| --- \| --- \| --- \| --- \| --- \| --- \| --- \| --- \| --- \| --- \| --- \| --- \| --- \| --- \| --- \| --- \| --- \| --- \| --- \| --- \| --- \| --- \| --- \| --- \| --- \| --- \| --- \| --- \| --- \| --- \| --- \| --- \| --- \| --- \| --- \| --- \| --- \| --- \| --- \| --- \| --- \| --- \| --- \| --- \| --- \| --- \| --- \| --- \| --- \| --- \| --- \| --- \| --- \| --- \| --- \| --- \| --- \| --- \| --- \| --- \| --- \| --- \| --- \| --- \| --- \| --- \| --- \| --- \| --- \| --- \| --- \| --- \| --- \| --- \| --- \| --- \| --- \| --- \| --- \| --- \| --- \| --- \| --- \| --- \| --- \| --- \| --- \| --- \| --- \| --- \| --- \| --- \| --- \| --- \| --- \| --- \| --- \| --- \| --- \| --- \| --- \| --- \| --- \| --- \| --- \| --- \| --- \| --- \| --- \| --- \| --- \| --- \| --- \| --- \| --- \| --- \| --- \| --- \| --- \| --- \| --- \| --- \| --- \| --- \| --- \| --- \| --- \| --- \| --- \| --- \| --- \| --- \| --- \| --- \| --- \| --- \| --- \| --- \| --- \| --- \| --- \| --- \| --- \| --- \| --- \| --- \| --- \| --- \| --- \| --- \| --- \| --- \| --- \| --- \| --- \| --- \| --- \| --- \| --- \| --- \| --- \| --- \| --- \| --- \| --- \| --- \| --- \| --- \| --- \| --- \| --- \| --- \| --- \| --- \| --- \| --- \| --- \| --- \| --- \| --- \| --- \| --- \| --- \| --- \| --- \| --- \| --- \| --- \| --- \| --- \| --- \| --- \| --- \| --- \| --- \| --- \| --- \| --- \| --- \| --- \| --- \| --- \| --- \| --- \| --- \| --- \| --- \| --- \| --- \| --- \| --- \| --- \| --- \| --- \| --- \| --- \| --- \| --- \| --- \| --- \| --- \| --- \| --- \| --- \| --- \| --- \| --- \| --- \| --- \| --- \| --- \| --- \| --- \| --- \| --- \| --- \| --- \| --- \| --- \| --- \| --- \| --- \| --- \| --- \| --- \| --- \| --- \| --- \| --- \| --- \| --- \| --- \| --- \| --- \| --- \| --- \| --- \| --- \| --- \| --- \| --- \| --- \| --- \| --- \| --- \| --- \| --- \| --- \| --- \| --- \| --- \| --- \| --- \| --- \| --- \| --- \| --- \| --- \| --- \| --- \| --- \| --- \| --- \| --- \| --- \| --- \| --- \| --- \| --- \| --- \| --- \| --- \| --- \| --- \| --- \| --- \| --- \| --- \| --- \| --- \| --- \| --- \| --- \| --- \| --- \| --- \| --- \| --- \| --- \| --- \| --- \| --- \| --- \| --- \| --- \| --- \| --- \| --- \| --- \| --- \| --- \| --- \| --- \| --- \| --- \| --- \| --- \| --- \| --- \| --- \| --- \| --- \| --- \| --- \| --- \| --- \| --- \| --- \| --- \| --- \| --- \| --- \| --- \| --- \| --- \| --- \| --- \| --- \| --- \| --- \| --- \| --- \| --- \| --- \| --- \| --- \| --- \| --- \| --- \| --- \| --- \| --- \| --- \| --- \| --- \| --- \| --- \| --- \| --- \| --- \| --- \| --- \| --- \| --- \| --- \| --- \| --- \| --- \| --- \| --- \| --- \| --- \| --- \| --- \| --- \| --- \| --- \| --- \| --- \| --- \| --- \| --- \| --- \| --- \| --- \| --- \| --- \| --- \| --- \| --- \| --- \| --- \| --- \| --- \| --- \| --- \| --- \| --- \| --- \| --- \| --- \| --- \| --- \| --- \| --- \| --- \| --- \| --- \| --- \| --- \| --- \| --- \| --- \| --- \| --- \| --- \| --- \| --- \| --- \| --- \| --- \| --- \| --- \| --- \| --- \| --- \| --- \| --- \| --- \| --- \| --- \| --- \| --- \| --- \| --- \| --- \| --- \| --- \| --- \| --- \| --- \| --- \| --- \| --- \| --- \| --- \| --- \| --- \| --- \| --- \| --- \| --- \| --- \| --- \| --- \| --- \| --- \| --- \| --- \| --- \| --- \| --- \| --- \| --- \| --- \| --- \| --- \| --- \| --- \| --- \| --- \| --- \| --- \| --- \| --- \| --- \| --- \| --- \| --- \| --- \| --- \| --- \| --- \| --- \| --- \| --- \| --- \| --- \| --- \| --- \| --- \| --- \| --- \| --- \| --- \| --- \| --- \| --- \| --- \| --- \| --- \| --- \| --- \| --- \| --- \| --- \| --- \| --- \| --- \| --- \| --- \| --- \| --- \| --- \| --- \| --- \| --- \| --- \| --- \| --- \| --- \| --- \| --- \| --- \| --- \| --- \| --- \| --- \| --- \| --- \| --- \| --- \| --- \| --- \| --- \| --- \| --- \| --- \| --- \| --- \| --- \| --- \| --- \| --- \| --- \| --- \| --- \| --- \| --- \| --- \| --- \| --- \| --- \| --- \| --- \| --- \| --- \| --- \| --- \| --- \| --- \| --- \| --- \| --- \| --- \| --- \| --- \| --- \| --- \| --- \| --- \| --- \| --- \| --- \| --- \| --- \| --- \| --- \| --- \| --- \| --- \| --- \| --- \| --- \| --- \| --- \| --- \| --- \| --- \| --- \| --- \| --- \| --- \| --- \| --- \| --- \| --- \| --- \| --- \| --- \| --- \| --- \| --- \| --- \| --- \| --- \| --- \| --- \| --- \| --- \| --- \| --- \| --- \| --- \| --- \| --- \| --- \| --- \| --- \| --- \| --- \| --- \| --- \| --- \| --- \| --- \| --- \| --- \| --- \| --- \| --- \| --- \| --- \| --- \| --- \| --- \| --- \| --- \| --- \| --- \| --- \| --- \| --- \| --- \| --- \| --- \| --- \| --- \| --- \| --- \| --- \| --- \| --- \| --- \| --- \| --- \| --- \| --- \| --- \| --- \| --- \| --- \| --- \| --- \| --- \| --- \| --- \| --- \| --- \| --- \| --- \| --- \| --- \| --- \| --- \| --- \| --- \| --- \| --- \| --- \| --- \| --- \| --- \| --- \| --- \| --- \| --- \| --- \| --- \| --- \| --- \| --- \| --- \| --- \| --- \| --- \| --- \| --- \| --- \| --- \| --- \| --- \| --- \| --- \| --- \| --- \| --- \| --- \| --- \| --- \| --- \| --- \| --- \| --- \| --- \| --- \| --- \| --- \| --- \| --- \| --- \| --- \| --- \| --- \| --- \| --- \| --- \| --- \| --- \| --- \| --- \| --- \| --- \| --- \| --- \| --- \| --- \| --- \| --- \| --- \| --- \| --- \| --- \| --- \| --- \| --- \| --- \| --- \| --- \| --- \| --- \| --- \| --- \| --- \| --- \| --- \| --- \| --- \| --- \| --- \| --- \| --- \| --- \| --- \| --- \| --- \| --- \| --- \| --- \| --- \| --- \| --- \| --- \| --- \| --- \| --- \| --- \| --- \| --- \| --- \| --- \| --- \| --- \| --- \| --- \| --- \| --- \| --- \| --- \| --- \| --- \| --- \| --- \| --- \| --- \| --- \| --- \| --- \| --- \| --- \| --- \| --- \| --- \| --- \| --- \| --- \| --- \| --- \| --- \| --- \| --- \| --- \| --- \| --- \| --- \| --- \| --- \| --- \| --- \| --- \| --- \| --- \| --- \| --- \| --- \| --- \| --- \| --- \| --- \| --- \| --- \| --- \| --- \| --- \| --- \| --- \| --- \| --- \| --- \| --- \| --- \| --- \| --- \| --- \| --- \| --- \| --- \| --- \| --- \| --- \| --- \| --- \| --- \| --- \| --- \| --- \| --- \| --- \| --- \| --- \| --- \| --- \| --- \| --- \| --- \| --- \| --- \| --- \| --- \| --- \| --- \| --- \| --- \| --- \| --- \| --- \| --- \| --- \| --- \| --- \| --- \| --- \| --- \| --- \| --- \| --- \| --- \| --- \| --- \| --- \| --- \| --- \| --- \| --- \| --- \| --- \| --- \| --- \| --- \| --- \| --- \| --- \| --- \| --- \| --- \| --- \| --- \| --- \| --- \| --- \| --- \| --- \| --- \| --- \| --- \| --- \| --- \| --- \| --- \| --- \| --- \| --- \| --- \| --- \| --- \| --- \| --- \| --- \| --- \| --- \| --- \| --- \| --- \| --- \| --- \| --- \| --- \| --- \| --- \| --- \| --- \| --- \| --- \| --- \| --- \| --- \| --- \| --- \| --- \| --- \| --- \| --- \| --- \| --- \| --- \| --- \| --- \| --- \| --- \| --- \| --- \| --- \| --- \| --- \| --- \| --- \| --- \| --- \| --- \| --- \| --- \| --- \| --- \| --- \| --- \| --- \| --- \| --- \| --- \| --- \| --- \| --- \| --- \| --- \| --- \| --- \| --- \| --- \| --- \| --- \| --- \| --- \| --- \| --- \| --- \| --- \| --- \| --- \| --- \| --- \| --- \| --- \| --- \| --- \| --- \| --- \| --- \| --- \| --- \| --- \| --- \| --- \| --- \| --- \| --- \| --- \| --- \| --- \| --- \| --- \| --- \| --- \| --- \| --- \| --- \| --- \| --- \| --- \| --- \| --- \| --- \| --- \| --- \| --- \| --- \| --- \| --- \| --- \| --- \| --- \| --- \| --- \| --- \| --- \| --- \| --- \| --- \| --- \| --- \| --- \| --- \| --- \| --- \| --- \| --- \| --- \| --- \| --- \| --- \| --- \| --- \| --- \| --- \| --- \| --- \| --- \| --- \| --- \| --- \| --- \| --- \| --- \| --- \| --- \| --- \| --- \| --- \| --- \| --- \| --- \| --- \| --- \| --- \| --- \| --- \| --- \| --- \| --- \| --- \| --- \| --- \| --- \| --- \| --- \| --- \| --- \| --- \| --- \| --- \| --- \| --- \| --- \| --- \| --- \| --- \| --- \| --- \| --- \| --- \| --- \| --- \| --- \| --- \| --- \| --- \| --- \| --- \| --- \| --- \| --- \| --- \| --- \| --- \| --- \| --- \| --- \| --- \| --- \| --- \| --- \| --- \| --- \| --- \| --- \| --- \| --- \| --- \| --- \| --- \| --- \| --- \| --- \| --- \| --- \| --- \| --- \| --- \| --- \| --- \| --- \| --- \| --- \| --- \| --- \| --- \| --- \| --- \| --- \| --- \| --- \| --- \| --- \| --- \| --- \| --- \| --- \| --- \| --- \| --- \| --- \| --- \| --- \| --- \| --- \| --- \| --- \| --- \| --- \| --- \| --- \| --- \| --- \| --- \| --- \| --- \| --- \| --- \| --- \| --- \| --- \| --- \| --- \| --- \| --- \| --- \| --- \| --- \| --- \| --- \| --- \| --- \| --- \| --- \| --- \| --- \| --- \| --- \| --- \| --- \| --- \| --- \| --- \| --- \| --- \| --- \| --- \| --- \| --- \| --- \| --- \| --- \| --- \| --- \| --- \| --- \| --- \| --- \| --- \| --- \| --- \| --- \| --- \| --- \| --- \| --- \| --- \| --- \| --- \| --- \| --- \| --- \| --- \| --- \| --- \| --- \| --- \| --- \| --- \| --- \| --- \| --- \| --- \| --- \| --- \| --- \| --- \| --- \| --- \| --- \| --- \| --- \| --- \| --- \| --- \| --- \| --- \| --- \| --- \| --- \| --- \| --- \| --- \| --- \| --- \| --- \| --- \| --- \| --- \| --- \| --- \| --- \| --- \| --- \| --- \| --- \| --- \| --- \| --- \| --- \| --- \| --- \| --- \| --- \| --- \| --- \| --- \| --- \| --- \| --- \| --- \| --- \| --- \| --- \| --- \| --- \| --- \| --- \| --- \| --- \| --- \| --- \| --- \| --- \| --- \| --- \| --- \| --- \| --- \| --- \| --- \| --- \| --- \| --- \| --- \| --- \| --- \| --- \| --- \| --- \| --- \| --- \| --- \| --- \| --- \| --- \| --- \| --- \| --- \| --- \| --- \| --- \| --- \| --- \| --- \| --- \| --- \| --- \| --- \| --- \| --- \| --- \| --- \| --- \| --- \| --- \| --- \| --- \| --- \| --- \| --- \| --- \| --- \| --- \| --- \| --- \| --- \| --- \| --- \| --- \| --- \| --- \| --- \| --- \| --- \| --- \| --- \| --- \| --- \| --- \| --- \| --- \| --- \| --- \| --- \| --- \| --- \| --- \| --- \| --- \| --- \| --- \| --- \| --- \| --- \| --- \| --- \| --- \| --- \| --- \| --- \| --- \| --- \| --- \| --- \| --- \| --- \| --- \| --- \| --- \| --- \| --- \| --- \| --- \| --- \| --- \| --- \| --- \| --- \| --- \| --- \| --- \| --- \| --- \| --- \| --- \| --- \| --- \| --- \| --- \| --- \| --- \| --- \| --- \| --- \| --- \| --- \| --- \| --- \| --- \| --- \| --- \| --- \| --- \| --- \| --- \| --- \| --- \| --- \| --- \| --- \| --- \| --- \| --- \| --- \| --- \| --- \| --- \| --- \| --- \| --- \| --- \| --- \| --- \| --- \| --- \| --- \| --- \| --- \| --- \| --- \| --- \| --- \| --- \| --- \| --- \| --- \| --- \| --- \| --- \| --- \| --- \| --- \| --- \| --- \| --- \| --- \| --- \| --- \| --- \| --- \| --- \| --- \| --- \| --- \| --- \| --- \| --- \| --- \| --- \| --- \| --- \| --- \| --- \| --- \| --- \| --- \| --- \| --- \| --- \| --- \| --- \| --- \| --- \| --- \| --- \| --- \| --- \| --- \| --- \| --- \| --- \| --- \| --- \| --- \| --- \| --- \| --- \| --- \| --- \| --- \| --- \| --- \| --- \| --- \| --- \| --- \| --- \| --- \| --- \| --- \| --- \| --- \| --- \| --- \| --- \| --- \| --- \| --- \| --- \| --- \| --- \| --- \| --- \| --- \| --- \| --- \| --- \| --- \| --- \| --- \| --- \| --- \| --- \| --- \| --- \| --- \| --- \| --- \| --- \| --- \| --- \| --- \| --- \| --- \| --- \| --- \| --- \| --- \| --- \| --- \| --- \| --- \| --- \| --- \| --- \| --- \| --- \| --- \| --- \| --- \| --- \| --- \| --- \| --- \| --- \| --- \| --- \| --- \| --- \| --- \| --- \| --- \| --- \| --- \| --- \| --- \| --- \| --- \| --- \| --- \| --- \| --- \| --- \| --- \| --- \| --- \| --- \| --- \| --- \| --- \| --- \| --- \| --- \| --- \| --- \| --- \| --- \| --- \| --- \| --- \| --- \| --- \| --- \| --- \| --- \| --- \| --- \| --- \| --- \| --- \| --- \| --- \| --- \| --- \| --- \| --- \| --- \| --- \| --- \| --- \| --- \| --- \| --- \| --- \| --- \| --- \| --- \| --- \| --- \| --- \| --- \| --- \| --- \| --- \| --- \| --- \| --- \| --- \| --- \| --- \| --- \| --- \| --- \| --- \| --- \| --- \| --- \| --- \| --- \| --- \| --- \| --- \| --- \| --- \| --- \| --- \| --- \| --- \| --- \| --- \| --- \| --- \| --- \| --- \| --- \| --- \| --- \| --- \| --- \| --- \| --- \| --- \| --- \| --- \| --- \| --- \| --- \| --- \| --- \| --- \| --- \| --- \| --- \| --- \| --- \| --- \| --- \| --- \| --- \| --- \| --- \| --- \| --- \| --- \| --- \| --- \| --- \| --- \| --- \| --- \| --- \| --- \| --- \| --- \| --- \| --- \| --- \| --- \| --- \| --- \| --- \| --- \| --- \| --- \| --- \| --- \| --- \| --- \| --- \| --- \| --- \| --- \| --- \| --- \| --- \| --- \| --- \| --- \| --- \| --- \| --- \| --- \| --- \| --- \| --- \| --- \| --- \| --- \| --- \| --- \| --- \| --- \| --- \| --- \| --- \| --- \| --- \| --- \| --- \| --- \| --- \| --- \| --- \| --- \| --- \| --- \| --- \| --- \| --- \| --- \| --- \| --- \| --- \| --- \| --- \| --- \| --- \| --- \| --- \| --- \| --- \| --- \| --- \| --- \| --- \| --- \| --- \| --- \| --- \| --- \| --- \| --- \| --- \| --- \| --- \| --- \| --- \| --- \| --- \| --- \| --- \| --- \| --- \| --- \| --- \| --- \| --- \| --- \| --- \| --- \| --- \| --- \| --- \| --- \| --- \| --- \| --- \| --- \| --- \| --- \| --- \| --- \| --- \| --- \| --- \| --- \| --- \| --- \| --- \| --- \| --- \| --- \| --- \| --- \| --- \| --- \| --- \| --- \| --- \| --- \| --- \| --- \| --- \| --- \| --- \| --- \| --- \| --- \| --- \| --- \| --- \| --- \| --- \| --- \| --- \| --- \| --- \| --- \| --- \| --- \| --- \| --- \| --- \| --- \| --- \| --- \| --- \| --- \| --- \| --- \| --- \| --- \| --- \| --- \| --- \| --- \| --- \| --- \| --- \| --- \| --- \| --- \| --- \| --- \| --- \| --- \| --- \| --- \| --- \| --- \| --- \| --- \| --- \| --- \| --- \| --- \| --- \| --- \| --- \| --- \| --- \| --- \| --- \| --- \| --- \| --- \| --- \| --- \| --- \| --- \| --- \| --- \| --- \| --- \| --- \| --- \| --- \| --- \| --- \| --- \| --- \| --- \| --- \| --- \| --- \| --- \| --- \| --- \| --- \| --- \| --- \| --- \| --- \| --- \| --- \| --- \| --- \| --- \| --- \| --- \| --- \| --- \| --- \| --- \| --- \| --- \| --- \| --- \| --- \| --- \| --- \| --- \| --- \| --- \| --- \| --- \| --- \| --- \| --- \| --- \| --- \| --- \| --- \| --- \| --- \| --- \| --- \| --- \| --- \| --- \| --- \| --- \| --- \| --- \| --- \| --- \| --- \| --- \| --- \| --- \| --- \| --- \| --- \| --- \| --- \| --- \| --- \| --- \| --- \| --- \| --- \| --- \| --- \| --- \| --- \| --- \| --- \| --- \| --- \| --- \| --- \| --- \| --- \| --- \| --- \| --- \| --- \| --- \| --- \| --- \| --- \| --- \| --- \| --- \| --- \| --- \| --- \| --- \| --- \| --- \| --- \| --- \| --- \| --- \| --- \| --- \| --- \| --- \| --- \| --- \| --- \| --- \| --- \| --- \| --- \| --- \| --- \| --- \| --- \| --- \| --- \| --- \| --- \| --- \| --- \| --- \| --- \| --- \| --- \| --- \| --- \| --- \| --- \| --- \| --- \| --- \| --- \| --- \| --- \| --- \| --- \| --- \| --- \| --- \| --- \| --- \| --- \| --- \| --- \| --- \| --- \| --- \| --- \| --- \| --- \| --- \| --- \| --- \| --- \| --- \| --- \| --- \| --- \| --- \| --- \| --- \| --- \| --- \| --- \| --- \| --- \| --- \| --- \| --- \| --- \| --- \| --- \| --- \| --- \| --- \| --- \| --- \| --- \| --- \| --- \| --- \| --- \| --- \| --- \| --- \| --- \| --- \| --- \| --- \| --- \| --- \| --- \| --- \| --- \| --- \| --- \| --- \| --- \| --- \| --- \| --- \| --- \| --- \| --- \| --- \| --- \| --- \| --- \| --- \| --- \| --- \| --- \| --- \| --- \| --- \| --- \| --- \| --- \| --- \| --- \| --- \| --- \| --- \| --- \| --- \| --- \| --- \| --- \| --- \| --- \| --- \| --- \| --- \| --- \| --- \| --- \| --- \| --- \| --- \| --- \| --- \| --- \| --- \| --- \| --- \| --- \| --- \| --- \| --- \| --- \| --- \| --- \| --- \| --- \| --- \| --- \| --- \| --- \| --- \| --- \| --- \| --- \| --- \| --- \| --- \| --- \| --- \| --- \| --- \| --- \| --- \| --- \| --- \| --- \| --- \| --- \| --- \| --- \| --- \| --- \| --- \| --- \| --- \| --- \| --- \| --- \| --- \| --- \| --- \| --- \| --- \| --- \| --- \| --- \| --- \| --- \| --- \| --- \| --- \| --- \| --- \| --- \| --- \| --- \| --- \| --- \| --- \| --- \| --- \| --- \| --- \| --- \| --- \| --- \| --- \| --- \| --- \| --- \| --- \| --- \| --- \| --- \| --- \| --- \| --- \| --- \| --- \| --- \| --- \| --- \| --- \| --- \| --- \| --- \| --- \| --- \| --- \| --- \| --- \| --- \| --- \| --- \| --- \| --- \| --- \| --- \| --- \| --- \| --- \| --- \| --- \| --- \| --- \| --- \| --- \| --- \| --- \| --- \| --- \| --- \| --- \| --- \| --- \| --- \| --- \| --- \| --- \| --- \| --- \| --- \| --- \| --- \| --- \| --- \| --- \| --- \| --- \| --- \| --- \| --- \| --- \| --- \| --- \| --- \| --- \| --- \| --- \| --- \| --- \| --- \| --- \| --- \| --- \| --- \| --- \| --- \| --- \| --- \| --- \| --- \| --- \| --- \| --- \| --- \| --- \| --- \| --- \| --- \| --- \| --- \| --- \| --- \| --- \| --- \| --- \| --- \| --- \| --- \| --- \| --- \| --- \| --- \| --- \| --- \| --- \| --- \| --- \| --- \| --- \| --- \| --- \| --- \| --- \| --- \| --- \| --- \| --- \| --- \| --- \| --- \| --- \| --- \| --- \| --- \| --- \| --- \| --- \| --- \| --- \| --- \| --- \| --- \| --- \| --- \| --- \| --- \| --- \| --- \| --- \| --- \| --- \| --- \| --- \| --- \| --- \| --- \| --- \| --- \| --- \| --- \| --- \| --- \| --- \| --- \| --- \| --- \| --- \| --- \| --- \| --- \| --- \| --- \| --- \| --- \| --- \| --- \| --- \| --- \| --- \| --- \| --- \| --- \| --- \| --- \| --- \| --- \| --- \| --- \| --- \| --- \| --- \| --- \| --- \| --- \| --- \| --- \| --- \| --- \| --- \| --- \| --- \| --- \| --- \| --- \| --- \| --- \| --- \| --- \| --- \| --- \| --- \| --- \| --- \| --- \| --- \| --- \| --- \| --- \| --- \| --- \| --- \| --- \| --- \| --- \| --- \| --- \| --- \| --- \| --- \| --- \| --- \| --- \| --- \| --- \| --- \| --- \| --- \| --- \| --- \| --- \| --- \| --- \| --- \| --- \| --- \| --- \| --- \| --- \| --- \| --- \| --- \| --- \| --- \| --- \| --- \| --- \| --- \| --- \| --- \| --- \| --- \| --- \| --- \| --- \| --- \| --- \| --- \| --- \| --- \| --- \| --- \| --- \| --- \| --- \| --- \| --- \| --- \| --- \| --- \| --- \| --- \| --- \| --- \| --- \| --- \| --- \| --- \| --- \| --- \| --- \| --- \| --- \| --- \| --- \| --- \| --- \| --- \| --- \| --- \| --- \| --- \| --- \| --- \| --- \| --- \| --- \| --- \| --- \| --- \| --- \| \| \| \| \| *** Misclassified observation** \| \| --- \| \| \| --- \| --- \| \| \|  \| \| \| \| \| \| **Discriminant Analysis Results** \| \| --- \| \| \| --- \| --- \| \| \| \| **The DISCRIM Procedure Classification Summary for Calibration Data: WORK.SORTTEMPTABLESORTED Cross-validation Summary using Linear Discriminant Function** \| \| --- \| \| \| --- \| --- \| \| \| \| **Number of Observations and Percent Classified into DIST** \| \| \| \| \| \| \| \| \| --- \| --- \| --- \| --- \| --- \| --- \| --- \| --- \| \| **From DIST** \| **1** \| **2** \| **3** \| **4** \| **5** \| **6** \| **Total** \| \| 1 \| \| 19 \| \| --- \| \| 38.00 \| \| \| 10 \| \| --- \| \| 20.00 \| \| \| 2 \| \| --- \| \| 4.00 \| \| \| 12 \| \| --- \| \| 24.00 \| \| \| 5 \| \| --- \| \| 10.00 \| \| \| 2 \| \| --- \| \| 4.00 \| \| \| 50 \| \| --- \| \| 100.00 \| \| \| 2 \| \| 12 \| \| --- \| \| 19.05 \| \| \| 42 \| \| --- \| \| 66.67 \| \| \| 3 \| \| --- \| \| 4.76 \| \| \| 5 \| \| --- \| \| 7.94 \| \| \| 0 \| \| --- \| \| 0.00 \| \| \| 1 \| \| --- \| \| 1.59 \| \| \| 63 \| \| --- \| \| 100.00 \| \| \| 3 \| \| 0 \| \| --- \| \| 0.00 \| \| \| 10 \| \| --- \| \| 23.26 \| \| \| 25 \| \| --- \| \| 58.14 \| \| \| 0 \| \| --- \| \| 0.00 \| \| \| 6 \| \| --- \| \| 13.95 \| \| \| 2 \| \| --- \| \| 4.65 \| \| \| 43 \| \| --- \| \| 100.00 \| \| \| 4 \| \| 8 \| \| --- \| \| 14.55 \| \| \| 1 \| \| --- \| \| 1.82 \| \| \| 0 \| \| --- \| \| 0.00 \| \| \| 44 \| \| --- \| \| 80.00 \| \| \| 0 \| \| --- \| \| 0.00 \| \| \| 2 \| \| --- \| \| 3.64 \| \| \| 55 \| \| --- \| \| 100.00 \| \| \| 5 \| \| 1 \| \| --- \| \| 1.67 \| \| \| 0 \| \| --- \| \| 0.00 \| \| \| 11 \| \| --- \| \| 18.33 \| \| \| 2 \| \| --- \| \| 3.33 \| \| \| 46 \| \| --- \| \| 76.67 \| \| \| 0 \| \| --- \| \| 0.00 \| \| \| 60 \| \| --- \| \| 100.00 \| \| \| 6 \| \| 0 \| \| --- \| \| 0.00 \| \| \| 2 \| \| --- \| \| 3.85 \| \| \| 0 \| \| --- \| \| 0.00 \| \| \| 0 \| \| --- \| \| 0.00 \| \| \| 16 \| \| --- \| \| 30.77 \| \| \| 34 \| \| --- \| \| 65.38 \| \| \| 52 \| \| --- \| \| 100.00 \| \| \| Total \| \| 40 \| \| --- \| \| 12.38 \| \| \| 65 \| \| --- \| \| 20.12 \| \| \| 41 \| \| --- \| \| 12.69 \| \| \| 63 \| \| --- \| \| 19.50 \| \| \| 73 \| \| --- \| \| 22.60 \| \| \| 41 \| \| --- \| \| 12.69 \| \| \| 323 \| \| --- \| \| 100.00 \| \| \| Priors \| \| 0.1548 \| \| --- \| \|  \| \| \| 0.19505 \| \| --- \| \|  \| \| \| 0.13313 \| \| --- \| \|  \| \| \| 0.17028 \| \| --- \| \|  \| \| \| 0.18576 \| \| --- \| \|  \| \| \| 0.16099 \| \| --- \| \|  \| \| \|  \| \| --- \| \|  \| \| \| \| --- \| --- \| --- \| --- \| --- \| --- \| --- \| --- \| --- \| --- \| --- \| --- \| --- \| --- \| --- \| --- \| --- \| --- \| --- \| --- \| --- \| --- \| --- \| --- \| --- \| --- \| --- \| --- \| --- \| --- \| --- \| --- \| --- \| --- \| --- \| --- \| --- \| --- \| --- \| --- \| --- \| --- \| --- \| --- \| --- \| --- \| --- \| --- \| --- \| --- \| --- \| --- \| --- \| --- \| --- \| --- \| --- \| --- \| --- \| --- \| --- \| --- \| --- \| --- \| --- \| --- \| --- \| --- \| --- \| --- \| --- \| --- \| --- \| --- \| --- \| --- \| --- \| --- \| --- \| --- \| --- \| --- \| --- \| --- \| --- \| --- \| --- \| --- \| --- \| --- \| --- \| --- \| --- \| --- \| --- \| --- \| --- \| --- \| --- \| --- \| --- \| --- \| --- \| --- \| --- \| --- \| --- \| --- \| --- \| --- \| --- \| --- \| --- \| --- \| --- \| --- \| --- \| --- \| --- \| --- \| --- \| --- \| --- \| --- \| --- \| --- \| --- \| --- \| --- \| --- \| --- \| --- \| --- \| --- \| --- \| --- \| --- \| --- \| --- \| --- \| --- \| --- \| --- \| --- \| --- \| --- \| --- \| --- \| --- \| --- \| --- \| --- \| --- \| --- \| --- \| --- \| --- \| --- \| --- \| --- \| --- \| --- \| --- \| --- \| --- \| --- \| --- \| --- \| --- \| --- \| --- \| --- \| --- \| --- \| --- \| --- \| --- \| --- \| --- \| --- \| --- \| --- \| --- \| --- \| --- \| --- \| --- \| --- \| --- \| --- \| --- \| --- \| --- \| \| \| **Error Count Estimates for DIST** \| \| \| \| \| \| \| \| \| --- \| --- \| --- \| --- \| --- \| --- \| --- \| --- \| \|  \| **1** \| **2** \| **3** \| **4** \| **5** \| **6** \| **Total** \| \| Rate \| 0.6200 \| 0.3333 \| 0.4186 \| 0.2000 \| 0.2333 \| 0.3462 \| 0.3498 \| \| Priors \| 0.1548 \| 0.1950 \| 0.1331 \| 0.1703 \| 0.1858 \| 0.1610 \|  \| \| \| \|  \| \| \| \| \| \| **Discriminant Analysis Results** \| \| --- \| \| \| --- \| --- \| \| \| \| **The DISCRIM Procedure Classification Results for Calibration Data: WORK.SORTTEMPTABLESORTED Cross-validation Results using Linear Discriminant Function** \| \| --- \| \| \| --- \| --- \| \| \| \| **Number of Observations and Average Posterior Probabilities Classified into DIST** \| \| \| \| \| \| \| \| --- \| --- \| --- \| --- \| --- \| --- \| --- \| \| **From DIST** \| **1** \| **2** \| **3** \| **4** \| **5** \| **6** \| \| 1 \| \| 19 \| \| --- \| \| 0.6161 \| \| \| 10 \| \| --- \| \| 0.6034 \| \| \| 2 \| \| --- \| \| 0.3760 \| \| \| 12 \| \| --- \| \| 0.5446 \| \| \| 5 \| \| --- \| \| 0.3975 \| \| \| 2 \| \| --- \| \| 0.7604 \| \| \| 2 \| \| 12 \| \| --- \| \| 0.3881 \| \| \| 42 \| \| --- \| \| 0.7289 \| \| \| 3 \| \| --- \| \| 0.7036 \| \| \| 5 \| \| --- \| \| 0.4251 \| \| \| 0 \| \| --- \| \| . \| \| \| 1 \| \| --- \| \| 1.0000 \| \| \| 3 \| \| 0 \| \| --- \| \| . \| \| \| 10 \| \| --- \| \| 0.7205 \| \| \| 25 \| \| --- \| \| 0.6871 \| \| \| 0 \| \| --- \| \| . \| \| \| 6 \| \| --- \| \| 0.6389 \| \| \| 2 \| \| --- \| \| 0.6886 \| \| \| 4 \| \| 8 \| \| --- \| \| 0.4424 \| \| \| 1 \| \| --- \| \| 0.2637 \| \| \| 0 \| \| --- \| \| . \| \| \| 44 \| \| --- \| \| 0.7072 \| \| \| 0 \| \| --- \| \| . \| \| \| 2 \| \| --- \| \| 0.4702 \| \| \| 5 \| \| 1 \| \| --- \| \| 0.3316 \| \| \| 0 \| \| --- \| \| . \| \| \| 11 \| \| --- \| \| 0.5471 \| \| \| 2 \| \| --- \| \| 0.4149 \| \| \| 46 \| \| --- \| \| 0.7526 \| \| \| 0 \| \| --- \| \| . \| \| \| 6 \| \| 0 \| \| --- \| \| . \| \| \| 2 \| \| --- \| \| 0.3291 \| \| \| 0 \| \| --- \| \| . \| \| \| 0 \| \| --- \| \| . \| \| \| 16 \| \| --- \| \| 0.7191 \| \| \| 34 \| \| --- \| \| 0.8247 \| \| \| Total \| \| 40 \| \| --- \| \| 0.5058 \| \| \| 65 \| \| --- \| \| 0.6889 \| \| \| 41 \| \| --- \| \| 0.6356 \| \| \| 63 \| \| --- \| \| 0.6446 \| \| \| 73 \| \| --- \| \| 0.7116 \| \| \| 41 \| \| --- \| \| 0.8019 \| \| \| Priors \| \| 0.1548 \| \| --- \| \|  \| \| \| 0.19505 \| \| --- \| \|  \| \| \| 0.13313 \| \| --- \| \|  \| \| \| 0.17028 \| \| --- \| \|  \| \| \| 0.18576 \| \| --- \| \|  \| \| \| 0.16099 \| \| --- \| \|  \| \| \| \| --- \| --- \| --- \| --- \| --- \| --- \| --- \| --- \| --- \| --- \| --- \| --- \| --- \| --- \| --- \| --- \| --- \| --- \| --- \| --- \| --- \| --- \| --- \| --- \| --- \| --- \| --- \| --- \| --- \| --- \| --- \| --- \| --- \| --- \| --- \| --- \| --- \| --- \| --- \| --- \| --- \| --- \| --- \| --- \| --- \| --- \| --- \| --- \| --- \| --- \| --- \| --- \| --- \| --- \| --- \| --- \| --- \| --- \| --- \| --- \| --- \| --- \| --- \| --- \| --- \| --- \| --- \| --- \| --- \| --- \| --- \| --- \| --- \| --- \| --- \| --- \| --- \| --- \| --- \| --- \| --- \| --- \| --- \| --- \| --- \| --- \| --- \| --- \| --- \| --- \| --- \| --- \| --- \| --- \| --- \| --- \| --- \| --- \| --- \| --- \| --- \| --- \| --- \| --- \| --- \| --- \| --- \| --- \| --- \| --- \| --- \| --- \| --- \| --- \| --- \| --- \| --- \| --- \| --- \| --- \| --- \| --- \| --- \| --- \| --- \| --- \| --- \| --- \| --- \| --- \| --- \| --- \| --- \| --- \| --- \| --- \| --- \| --- \| --- \| --- \| --- \| --- \| --- \| --- \| --- \| --- \| --- \| --- \| --- \| --- \| --- \| --- \| --- \| --- \| --- \| --- \| --- \| --- \| --- \| --- \| --- \| --- \| --- \| --- \| --- \| --- \| --- \| \| \| **Posterior Probability Error Rate Estimates for DIST** \| \| \| \| \| \| \| \| \| --- \| --- \| --- \| --- \| --- \| --- \| --- \| --- \| \| **Estimate** \| **1** \| **2** \| **3** \| **4** \| **5** \| **6** \| **Total** \| \| Stratified \| 0.5953 \| 0.2893 \| 0.3940 \| 0.2617 \| 0.1343 \| 0.3677 \| 0.3297 \| \| Unstratified \| 0.5953 \| 0.2893 \| 0.3940 \| 0.2617 \| 0.1343 \| 0.3677 \| 0.3297 \| \| Priors \| 0.1548 \| 0.1950 \| 0.1331 \| 0.1703 \| 0.1858 \| 0.1610 \|  \| \| \| \|  \| \| \| |
| --- | --- | --- | --- | --- | --- | --- | --- | --- | --- | --- | --- | --- | --- | --- | --- | --- | --- | --- | --- | --- | --- | --- | --- | --- | --- | --- | --- | --- | --- | --- | --- | --- | --- | --- | --- | --- | --- | --- | --- | --- | --- | --- | --- | --- | --- | --- | --- | --- | --- | --- | --- | --- | --- | --- | --- | --- | --- | --- | --- | --- | --- | --- | --- | --- | --- | --- | --- | --- | --- | --- | --- | --- | --- | --- | --- | --- | --- | --- | --- | --- | --- | --- | --- | --- | --- | --- | --- | --- | --- | --- | --- | --- | --- | --- | --- | --- | --- | --- | --- | --- | --- | --- | --- | --- | --- | --- | --- | --- | --- | --- | --- | --- | --- | --- | --- | --- | --- | --- | --- | --- | --- | --- | --- | --- | --- | --- | --- | --- | --- | --- | --- | --- | --- | --- | --- | --- | --- | --- | --- | --- | --- | --- | --- | --- | --- | --- | --- | --- | --- | --- | --- | --- | --- | --- | --- | --- | --- | --- | --- | --- | --- | --- | --- | --- | --- | --- | --- | --- | --- | --- | --- | --- | --- | --- | --- | --- | --- | --- | --- | --- | --- | --- | --- | --- | --- | --- | --- | --- | --- | --- | --- | --- | --- | --- | --- | --- | --- | --- | --- | --- | --- | --- | --- | --- | --- | --- | --- | --- | --- | --- | --- | --- | --- | --- | --- | --- | --- | --- | --- | --- | --- | --- | --- | --- | --- | --- | --- | --- | --- | --- | --- | --- | --- | --- | --- | --- | --- | --- | --- | --- | --- | --- | --- | --- | --- | --- | --- | --- | --- | --- | --- | --- | --- | --- | --- | --- | --- | --- | --- | --- | --- | --- | --- | --- | --- | --- | --- | --- | --- | --- | --- | --- | --- | --- | --- | --- | --- | --- | --- | --- | --- | --- | --- | --- | --- | --- | --- | --- | --- | --- | --- | --- | --- | --- | --- | --- | --- | --- | --- | --- | --- | --- | --- | --- | --- | --- | --- | --- | --- | --- | --- | --- | --- | --- | --- | --- | --- | --- | --- | --- | --- | --- | --- | --- | --- | --- | --- | --- | --- | --- | --- | --- | --- | --- | --- | --- | --- | --- | --- | --- | --- | --- | --- | --- | --- | --- | --- | --- | --- | --- | --- | --- | --- | --- | --- | --- | --- | --- | --- | --- | --- | --- | --- | --- | --- | --- | --- | --- | --- | --- | --- | --- | --- | --- | --- | --- | --- | --- | --- | --- | --- | --- | --- | --- | --- | --- | --- | --- | --- | --- | --- | --- | --- | --- | --- | --- | --- | --- | --- | --- | --- | --- | --- | --- | --- | --- | --- | --- | --- | --- | --- | --- | --- | --- | --- | --- | --- | --- | --- | --- | --- | --- | --- | --- | --- | --- | --- | --- | --- | --- | --- | --- | --- | --- | --- | --- | --- | --- | --- | --- | --- | --- | --- | --- | --- | --- | --- | --- | --- | --- | --- | --- | --- | --- | --- | --- | --- | --- | --- | --- | --- | --- | --- | --- | --- | --- | --- | --- | --- | --- | --- | --- | --- | --- | --- | --- | --- | --- | --- | --- | --- | --- | --- | --- | --- | --- | --- | --- | --- | --- | --- | --- | --- | --- | --- | --- | --- | --- | --- | --- | --- | --- | --- | --- | --- | --- | --- | --- | --- | --- | --- | --- | --- | --- | --- | --- | --- | --- | --- | --- | --- | --- | --- | --- | --- | --- | --- | --- | --- | --- | --- | --- | --- | --- | --- | --- | --- | --- | --- | --- | --- | --- | --- | --- | --- | --- | --- | --- | --- | --- | --- | --- | --- | --- | --- | --- | --- | --- | --- | --- | --- | --- | --- | --- | --- | --- | --- | --- | --- | --- | --- | --- | --- | --- | --- | --- | --- | --- | --- | --- | --- | --- | --- | --- | --- | --- | --- | --- | --- | --- | --- | --- | --- | --- | --- | --- | --- | --- | --- | --- | --- | --- | --- | --- | --- | --- | --- | --- | --- | --- | --- | --- | --- | --- | --- | --- | --- | --- | --- | --- | --- | --- | --- | --- | --- | --- | --- | --- | --- | --- | --- | --- | --- | --- | --- | --- | --- | --- | --- | --- | --- | --- | --- | --- | --- | --- | --- | --- | --- | --- | --- | --- | --- | --- | --- | --- | --- | --- | --- | --- | --- | --- | --- | --- | --- | --- | --- | --- | --- | --- | --- | --- | --- | --- | --- | --- | --- | --- | --- | --- | --- | --- | --- | --- | --- | --- | --- | --- | --- | --- | --- | --- | --- | --- | --- | --- | --- | --- | --- | --- | --- | --- | --- | --- | --- | --- | --- | --- | --- | --- | --- | --- | --- | --- | --- | --- | --- | --- | --- | --- | --- | --- | --- | --- | --- | --- | --- | --- | --- | --- | --- | --- | --- | --- | --- | --- | --- | --- | --- | --- | --- | --- | --- | --- | --- | --- | --- | --- | --- | --- | --- | --- | --- | --- | --- | --- | --- | --- | --- | --- | --- | --- | --- | --- | --- | --- | --- | --- | --- | --- | --- | --- | --- | --- | --- | --- | --- | --- | --- | --- | --- | --- | --- | --- | --- | --- | --- | --- | --- | --- | --- | --- | --- | --- | --- | --- | --- | --- | --- | --- | --- | --- | --- | --- | --- | --- | --- | --- | --- | --- | --- | --- | --- | --- | --- | --- | --- | --- | --- | --- | --- | --- | --- | --- | --- | --- | --- | --- | --- | --- | --- | --- | --- | --- | --- | --- | --- | --- | --- | --- | --- | --- | --- | --- | --- | --- | --- | --- | --- | --- | --- | --- | --- | --- | --- | --- | --- | --- | --- | --- | --- | --- | --- | --- | --- | --- | --- | --- | --- | --- | --- | --- | --- | --- | --- | --- | --- | --- | --- | --- | --- | --- | --- | --- | --- | --- | --- | --- | --- | --- | --- | --- | --- | --- | --- | --- | --- | --- | --- | --- | --- | --- | --- | --- | --- | --- | --- | --- | --- | --- | --- | --- | --- | --- | --- | --- | --- | --- | --- | --- | --- | --- | --- | --- | --- | --- | --- | --- | --- | --- | --- | --- | --- | --- | --- | --- | --- | --- | --- | --- | --- | --- | --- | --- | --- | --- | --- | --- | --- | --- | --- | --- | --- | --- | --- | --- | --- | --- | --- | --- | --- | --- | --- | --- | --- | --- | --- | --- | --- | --- | --- | --- | --- | --- | --- | --- | --- | --- | --- | --- | --- | --- | --- | --- | --- | --- | --- | --- | --- | --- | --- | --- | --- | --- | --- | --- | --- | --- | --- | --- | --- | --- | --- | --- | --- | --- | --- | --- | --- | --- | --- | --- | --- | --- | --- | --- | --- | --- | --- | --- | --- | --- | --- | --- | --- | --- | --- | --- | --- | --- | --- | --- | --- | --- | --- | --- | --- | --- | --- | --- | --- | --- | --- | --- | --- | --- | --- | --- | --- | --- | --- | --- | --- | --- | --- | --- | --- | --- | --- | --- | --- | --- | --- | --- | --- | --- | --- | --- | --- | --- | --- | --- | --- | --- | --- | --- | --- | --- | --- | --- | --- | --- | --- | --- | --- | --- | --- | --- | --- | --- | --- | --- | --- | --- | --- | --- | --- | --- | --- | --- | --- | --- | --- | --- | --- | --- | --- | --- | --- | --- | --- | --- | --- | --- | --- | --- | --- | --- | --- | --- | --- | --- | --- | --- | --- | --- | --- | --- | --- | --- | --- | --- | --- | --- | --- | --- | --- | --- | --- | --- | --- | --- | --- | --- | --- | --- | --- | --- | --- | --- | --- | --- | --- | --- | --- | --- | --- | --- | --- | --- | --- | --- | --- | --- | --- | --- | --- | --- | --- | --- | --- | --- | --- | --- | --- | --- | --- | --- | --- | --- | --- | --- | --- | --- | --- | --- | --- | --- | --- | --- | --- | --- | --- | --- | --- | --- | --- | --- | --- | --- | --- | --- | --- | --- | --- | --- | --- | --- | --- | --- | --- | --- | --- | --- | --- | --- | --- | --- | --- | --- | --- | --- | --- | --- | --- | --- | --- | --- | --- | --- | --- | --- | --- | --- | --- | --- | --- | --- | --- | --- | --- | --- | --- | --- | --- | --- | --- | --- | --- | --- | --- | --- | --- | --- | --- | --- | --- | --- | --- | --- | --- | --- | --- | --- | --- | --- | --- | --- | --- | --- | --- | --- | --- | --- | --- | --- | --- | --- | --- | --- | --- | --- | --- | --- | --- | --- | --- | --- | --- | --- | --- | --- | --- | --- | --- | --- | --- | --- | --- | --- | --- | --- | --- | --- | --- | --- | --- | --- | --- | --- | --- | --- | --- | --- | --- | --- | --- | --- | --- | --- | --- | --- | --- | --- | --- | --- | --- | --- | --- | --- | --- | --- | --- | --- | --- | --- | --- | --- | --- | --- | --- | --- | --- | --- | --- | --- | --- | --- | --- | --- | --- | --- | --- | --- | --- | --- | --- | --- | --- | --- | --- | --- | --- | --- | --- | --- | --- | --- | --- | --- | --- | --- | --- | --- | --- | --- | --- | --- | --- | --- | --- | --- | --- | --- | --- | --- | --- | --- | --- | --- | --- | --- | --- | --- | --- | --- | --- | --- | --- | --- | --- | --- | --- | --- | --- | --- | --- | --- | --- | --- | --- | --- | --- | --- | --- | --- | --- | --- | --- | --- | --- | --- | --- | --- | --- | --- | --- | --- | --- | --- | --- | --- | --- | --- | --- | --- | --- | --- | --- | --- | --- | --- | --- | --- | --- | --- | --- | --- | --- | --- | --- | --- | --- | --- | --- | --- | --- | --- | --- | --- | --- | --- | --- | --- | --- | --- | --- | --- | --- | --- | --- | --- | --- | --- | --- | --- | --- | --- | --- | --- | --- | --- | --- | --- | --- | --- | --- | --- | --- | --- | --- | --- | --- | --- | --- | --- | --- | --- | --- | --- | --- | --- | --- | --- | --- | --- | --- | --- | --- | --- | --- | --- | --- | --- | --- | --- | --- | --- | --- | --- | --- | --- | --- | --- | --- | --- | --- | --- | --- | --- | --- | --- | --- | --- | --- | --- | --- | --- | --- | --- | --- | --- | --- | --- | --- | --- | --- | --- | --- | --- | --- | --- | --- | --- | --- | --- | --- | --- | --- | --- | --- | --- | --- | --- | --- | --- | --- | --- | --- | --- | --- | --- | --- | --- | --- | --- | --- | --- | --- | --- | --- | --- | --- | --- | --- | --- | --- | --- | --- | --- | --- | --- | --- | --- | --- | --- | --- | --- | --- | --- | --- | --- | --- | --- | --- | --- | --- | --- | --- | --- | --- | --- | --- | --- | --- | --- | --- | --- | --- | --- | --- | --- | --- | --- | --- | --- | --- | --- | --- | --- | --- | --- | --- | --- | --- | --- | --- | --- | --- | --- | --- | --- | --- | --- | --- | --- | --- | --- | --- | --- | --- | --- | --- | --- | --- | --- | --- | --- | --- | --- | --- | --- | --- | --- | --- | --- | --- | --- | --- | --- | --- | --- | --- | --- | --- | --- | --- | --- | --- | --- | --- | --- | --- | --- | --- | --- | --- | --- | --- | --- | --- | --- | --- | --- | --- | --- | --- | --- | --- | --- | --- | --- | --- | --- | --- | --- | --- | --- | --- | --- | --- | --- | --- | --- | --- | --- | --- | --- | --- | --- | --- | --- | --- | --- | --- | --- | --- | --- | --- | --- | --- | --- | --- | --- | --- | --- | --- | --- | --- | --- | --- | --- | --- | --- | --- | --- | --- | --- | --- | --- | --- | --- | --- | --- | --- | --- | --- | --- | --- | --- | --- | --- | --- | --- | --- | --- | --- | --- | --- | --- | --- | --- | --- | --- | --- | --- | --- | --- | --- | --- | --- | --- | --- | --- | --- | --- | --- | --- | --- | --- | --- | --- | --- | --- | --- | --- | --- | --- | --- | --- | --- | --- | --- | --- | --- | --- | --- | --- | --- | --- | --- | --- | --- | --- | --- | --- | --- | --- | --- | --- | --- | --- | --- | --- | --- | --- | --- | --- | --- | --- | --- | --- | --- | --- | --- | --- | --- | --- | --- | --- | --- | --- | --- | --- | --- | --- | --- | --- | --- | --- | --- | --- | --- | --- | --- | --- | --- | --- | --- | --- | --- | --- | --- | --- | --- | --- | --- | --- | --- | --- | --- | --- | --- | --- | --- | --- | --- | --- | --- | --- | --- | --- | --- | --- | --- | --- | --- | --- | --- | --- | --- | --- | --- | --- | --- | --- | --- | --- | --- | --- | --- | --- | --- | --- | --- | --- | --- | --- | --- | --- | --- | --- | --- | --- | --- | --- | --- | --- | --- | --- | --- | --- | --- | --- | --- | --- | --- | --- | --- | --- | --- | --- | --- | --- | --- | --- | --- | --- | --- | --- | --- | --- | --- | --- | --- | --- | --- | --- | --- | --- | --- | --- | --- | --- | --- | --- | --- | --- | --- | --- | --- | --- | --- | --- | --- | --- | --- | --- | --- | --- | --- | --- | --- | --- | --- | --- | --- | --- | --- | --- | --- | --- | --- | --- | --- | --- | --- | --- | --- | --- | --- | --- | --- | --- | --- | --- | --- | --- | --- | --- | --- | --- | --- | --- | --- | --- | --- | --- | --- | --- | --- | --- | --- | --- | --- | --- | --- | --- | --- | --- | --- | --- | --- | --- | --- | --- | --- | --- | --- | --- | --- | --- | --- | --- | --- | --- | --- | --- | --- | --- | --- | --- | --- | --- | --- | --- | --- | --- | --- | --- | --- | --- | --- | --- | --- | --- | --- | --- | --- | --- | --- | --- | --- | --- | --- | --- | --- | --- | --- | --- | --- | --- | --- | --- | --- | --- | --- | --- | --- | --- | --- | --- | --- | --- | --- | --- | --- | --- | --- | --- | --- | --- | --- | --- | --- | --- | --- | --- | --- | --- | --- | --- | --- | --- | --- | --- | --- | --- | --- | --- | --- | --- | --- | --- | --- | --- | --- | --- | --- | --- | --- | --- | --- | --- | --- | --- | --- | --- | --- | --- | --- | --- | --- | --- | --- | --- | --- | --- | --- | --- | --- | --- | --- | --- | --- | --- | --- | --- | --- | --- | --- | --- | --- | --- | --- | --- | --- | --- | --- | --- | --- | --- | --- | --- | --- | --- | --- | --- | --- | --- | --- | --- | --- | --- | --- | --- | --- | --- | --- | --- | --- | --- | --- | --- | --- | --- | --- | --- | --- | --- | --- | --- | --- | --- | --- | --- | --- | --- | --- | --- | --- | --- | --- | --- | --- | --- | --- | --- | --- | --- | --- | --- | --- | --- | --- | --- | --- | --- | --- | --- | --- | --- | --- | --- | --- | --- | --- | --- | --- | --- | --- | --- | --- | --- | --- | --- | --- | --- | --- | --- | --- | --- | --- | --- | --- | --- | --- | --- | --- | --- | --- | --- | --- | --- | --- | --- | --- | --- | --- | --- | --- | --- | --- | --- | --- | --- | --- | --- | --- | --- | --- | --- | --- | --- | --- | --- | --- | --- | --- | --- | --- | --- | --- | --- | --- | --- | --- | --- | --- | --- | --- | --- | --- | --- | --- | --- | --- | --- | --- | --- | --- | --- | --- | --- | --- | --- | --- | --- | --- | --- | --- | --- | --- | --- | --- | --- | --- | --- | --- | --- | --- | --- | --- | --- | --- | --- | --- | --- | --- | --- | --- | --- | --- | --- | --- | --- | --- | --- | --- | --- | --- | --- | --- | --- | --- | --- | --- | --- | --- | --- | --- | --- | --- | --- | --- | --- | --- | --- | --- | --- | --- | --- | --- | --- | --- | --- | --- | --- | --- | --- | --- | --- | --- | --- | --- | --- | --- | --- | --- | --- | --- | --- | --- | --- | --- | --- | --- | --- | --- | --- | --- | --- | --- | --- | --- | --- | --- | --- | --- | --- | --- | --- | --- | --- | --- | --- | --- | --- | --- | --- | --- | --- | --- | --- | --- | --- | --- | --- | --- | --- | --- | --- | --- | --- | --- | --- | --- | --- | --- | --- | --- | --- | --- | --- | --- | --- | --- | --- | --- | --- | --- | --- | --- | --- | --- | --- | --- | --- | --- | --- | --- | --- | --- | --- | --- | --- | --- | --- | --- | --- | --- | --- | --- | --- | --- | --- | --- | --- | --- | --- | --- | --- | --- | --- | --- | --- | --- | --- | --- | --- | --- | --- | --- | --- | --- | --- | --- | --- | --- | --- | --- | --- | --- | --- | --- | --- | --- | --- | --- | --- | --- | --- | --- | --- | --- | --- | --- | --- | --- | --- | --- | --- | --- | --- | --- | --- | --- | --- | --- | --- | --- | --- | --- | --- | --- | --- | --- | --- | --- | --- | --- | --- | --- | --- | --- | --- | --- | --- | --- | --- | --- | --- | --- | --- | --- | --- | --- | --- | --- | --- | --- | --- | --- | --- | --- | --- | --- | --- | --- | --- | --- | --- | --- | --- | --- | --- | --- | --- | --- | --- | --- | --- | --- | --- | --- | --- | --- | --- | --- | --- | --- | --- | --- | --- | --- | --- | --- | --- | --- | --- | --- | --- | --- | --- | --- | --- | --- | --- | --- | --- | --- | --- | --- | --- | --- | --- | --- | --- | --- | --- | --- | --- | --- | --- | --- | --- | --- | --- | --- | --- | --- | --- | --- | --- | --- | --- | --- | --- | --- | --- | --- | --- | --- | --- | --- | --- | --- | --- | --- | --- | --- | --- | --- | --- | --- | --- | --- | --- | --- | --- | --- | --- | --- | --- | --- | --- | --- | --- | --- | --- | --- | --- | --- | --- | --- | --- | --- | --- | --- | --- | --- | --- | --- | --- | --- | --- | --- | --- | --- | --- | --- | --- | --- | --- | --- | --- | --- | --- | --- | --- | --- | --- | --- | --- | --- | --- | --- | --- | --- | --- | --- | --- | --- | --- | --- | --- | --- | --- | --- | --- | --- | --- | --- | --- | --- | --- | --- | --- | --- | --- | --- | --- | --- | --- | --- | --- | --- | --- | --- | --- | --- | --- | --- | --- | --- | --- | --- | --- | --- | --- | --- | --- | --- | --- | --- | --- | --- | --- | --- | --- | --- | --- | --- | --- | --- | --- | --- | --- | --- | --- | --- | --- | --- | --- | --- | --- | --- | --- | --- | --- | --- | --- | --- | --- | --- | --- | --- | --- | --- | --- | --- | --- | --- | --- | --- | --- | --- | --- | --- | --- | --- | --- | --- | --- | --- | --- | --- | --- | --- | --- | --- | --- | --- | --- | --- | --- | --- | --- | --- | --- | --- | --- | --- | --- | --- | --- | --- | --- | --- | --- | --- | --- | --- | --- | --- | --- | --- | --- | --- | --- | --- | --- | --- | --- | --- | --- | --- | --- | --- | --- | --- | --- | --- | --- | --- | --- | --- | --- | --- | --- | --- | --- | --- | --- | --- | --- | --- | --- | --- | --- | --- | --- | --- | --- | --- | --- | --- | --- | --- | --- | --- | --- | --- | --- | --- | --- | --- | --- | --- | --- | --- | --- | --- | --- | --- | --- | --- | --- | --- | --- | --- | --- | --- | --- | --- | --- | --- | --- | --- | --- | --- | --- | --- | --- | --- | --- | --- | --- | --- | --- | --- | --- | --- | --- | --- | --- | --- | --- | --- | --- | --- | --- | --- | --- | --- | --- | --- | --- | --- | --- | --- | --- | --- | --- | --- | --- | --- | --- | --- | --- | --- | --- | --- | --- | --- | --- | --- | --- | --- | --- | --- | --- | --- | --- | --- | --- | --- | --- | --- | --- | --- | --- | --- | --- | --- | --- | --- | --- | --- | --- | --- | --- | --- | --- | --- | --- | --- | --- | --- | --- | --- | --- | --- | --- | --- | --- | --- | --- | --- | --- | --- | --- | --- | --- | --- | --- | --- | --- | --- | --- | --- | --- | --- | --- | --- | --- | --- | --- | --- | --- | --- | --- | --- | --- | --- | --- | --- | --- | --- | --- | --- | --- | --- | --- | --- | --- | --- | --- | --- | --- | --- | --- | --- | --- | --- | --- | --- | --- | --- | --- | --- | --- | --- | --- | --- | --- | --- | --- | --- | --- | --- | --- | --- | --- | --- | --- | --- | --- | --- | --- | --- | --- | --- | --- | --- | --- | --- | --- | --- | --- | --- | --- | --- | --- | --- | --- | --- | --- | --- | --- | --- | --- | --- | --- | --- | --- | --- | --- | --- | --- | --- | --- | --- | --- | --- | --- | --- | --- | --- | --- | --- | --- | --- | --- | --- | --- | --- | --- | --- | --- | --- | --- | --- | --- | --- | --- | --- | --- | --- | --- | --- | --- | --- | --- | --- | --- | --- | --- | --- | --- | --- | --- | --- | --- | --- | --- | --- | --- | --- | --- | --- | --- | --- | --- | --- | --- | --- | --- | --- | --- | --- | --- | --- | --- | --- | --- | --- | --- | --- | --- | --- | --- | --- | --- | --- | --- | --- | --- | --- | --- | --- | --- | --- | --- | --- | --- | --- | --- | --- | --- | --- | --- | --- | --- | --- | --- | --- | --- | --- | --- | --- | --- | --- | --- | --- | --- | --- | --- | --- | --- | --- | --- | --- | --- | --- | --- | --- | --- | --- | --- | --- | --- | --- | --- | --- | --- | --- | --- | --- | --- | --- | --- | --- | --- | --- | --- | --- | --- | --- | --- | --- | --- | --- | --- | --- | --- | --- | --- | --- | --- | --- | --- | --- | --- | --- | --- | --- | --- | --- | --- | --- | --- | --- | --- | --- | --- | --- | --- | --- | --- | --- | --- | --- | --- | --- | --- | --- | --- | --- | --- | --- | --- | --- | --- | --- | --- | --- | --- | --- | --- | --- | --- | --- | --- | --- | --- | --- | --- | --- | --- | --- | --- | --- | --- | --- | --- | --- | --- | --- | --- | --- | --- | --- | --- | --- | --- | --- | --- | --- | --- | --- | --- | --- | --- | --- | --- | --- | --- | --- | --- | --- | --- | --- | --- | --- | --- | --- | --- | --- | --- | --- | --- | --- | --- | --- | --- | --- | --- | --- | --- | --- | --- | --- | --- | --- | --- | --- | --- | --- | --- | --- | --- | --- | --- | --- | --- | --- | --- | --- | --- | --- | --- | --- | --- | --- | --- | --- | --- | --- | --- | --- | --- | --- | --- | --- | --- | --- | --- | --- | --- | --- | --- | --- | --- | --- | --- | --- | --- | --- | --- | --- | --- | --- | --- | --- | --- | --- | --- | --- | --- | --- | --- | --- | --- | --- | --- | --- | --- | --- | --- | --- | --- | --- | --- | --- | --- | --- | --- | --- | --- | --- | --- | --- | --- | --- | --- | --- | --- | --- | --- | --- | --- | --- | --- | --- | --- | --- | --- | --- | --- | --- | --- | --- | --- | --- | --- | --- | --- | --- | --- | --- | --- | --- | --- | --- | --- | --- | --- | --- | --- | --- | --- | --- | --- | --- | --- | --- | --- | --- | --- | --- | --- | --- | --- | --- | --- | --- | --- | --- | --- | --- | --- | --- | --- | --- | --- | --- | --- | --- | --- | --- | --- | --- | --- | --- | --- | --- | --- | --- | --- | --- | --- | --- | --- | --- | --- | --- | --- | --- | --- | --- | --- | --- | --- | --- | --- | --- | --- | --- | --- | --- | --- | --- | --- | --- | --- | --- | --- | --- | --- | --- | --- | --- | --- | --- | --- | --- | --- | --- | --- | --- | --- | --- | --- | --- | --- | --- | --- | --- | --- | --- | --- | --- | --- | --- | --- | --- | --- | --- | --- | --- | --- | --- | --- | --- | --- | --- | --- | --- | --- | --- | --- | --- | --- | --- | --- | --- | --- | --- | --- | --- | --- | --- | --- | --- | --- | --- | --- | --- | --- | --- | --- | --- | --- | --- | --- | --- | --- | --- | --- | --- | --- | --- | --- | --- | --- | --- | --- | --- | --- | --- | --- | --- | --- | --- | --- | --- | --- | --- | --- | --- | --- | --- | --- | --- | --- | --- | --- | --- | --- | --- | --- | --- | --- | --- | --- | --- | --- | --- | --- | --- | --- | --- | --- | --- | --- | --- | --- | --- | --- | --- | --- | --- | --- | --- | --- | --- | --- | --- | --- | --- | --- | --- | --- | --- | --- | --- | --- | --- | --- | --- | --- | --- | --- | --- | --- | --- | --- | --- | --- | --- | --- | --- | --- | --- | --- | --- | --- | --- | --- | --- | --- | --- | --- | --- | --- | --- | --- | --- | --- | --- | --- | --- | --- | --- | --- | --- | --- | --- | --- | --- | --- | --- | --- | --- | --- | --- | --- | --- | --- | --- | --- | --- | --- | --- | --- | --- | --- | --- | --- | --- | --- | --- | --- | --- | --- | --- | --- | --- | --- | --- | --- | --- | --- | --- | --- | --- | --- | --- | --- | --- | --- | --- | --- | --- | --- | --- | --- | --- | --- | --- | --- | --- | --- | --- | --- | --- | --- | --- | --- | --- | --- | --- | --- | --- | --- | --- | --- | --- | --- | --- | --- | --- | --- | --- | --- | --- | --- | --- | --- | --- | --- | --- | --- | --- | --- | --- | --- | --- | --- | --- | --- | --- | --- | --- | --- | --- | --- | --- | --- | --- | --- | --- | --- | --- | --- | --- | --- | --- | --- | --- | --- | --- | --- | --- | --- | --- | --- | --- | --- | --- | --- | --- | --- | --- | --- | --- | --- | --- | --- | --- | --- | --- | --- | --- | --- | --- | --- | --- | --- | --- | --- | --- | --- | --- | --- | --- | --- | --- | --- | --- | --- | --- | --- | --- | --- | --- | --- | --- | --- | --- | --- | --- | --- | --- | --- | --- | --- | --- | --- | --- | --- | --- | --- | --- | --- | --- | --- | --- | --- | --- | --- | --- | --- | --- | --- | --- | --- | --- | --- | --- | --- | --- | --- | --- | --- | --- | --- | --- | --- | --- | --- | --- | --- | --- | --- | --- | --- | --- | --- | --- | --- | --- | --- | --- | --- | --- | --- | --- | --- | --- | --- | --- | --- | --- | --- | --- | --- | --- | --- | --- | --- | --- | --- | --- | --- | --- | --- | --- | --- | --- | --- | --- | --- | --- | --- | --- | --- | --- | --- | --- | --- | --- | --- | --- | --- | --- | --- | --- | --- | --- | --- | --- | --- | --- | --- | --- | --- | --- | --- | --- | --- | --- | --- | --- | --- | --- | --- | --- | --- | --- | --- | --- | --- | --- | --- | --- | --- | --- | --- | --- | --- | --- | --- | --- | --- | --- | --- | --- | --- | --- | --- | --- | --- | --- | --- | --- | --- | --- | --- | --- | --- | --- | --- | --- | --- | --- | --- | --- | --- | --- | --- | --- | --- | --- | --- | --- | --- | --- | --- | --- | --- | --- | --- | --- | --- | --- | --- | --- | --- | --- | --- | --- | --- | --- | --- | --- | --- | --- | --- | --- | --- | --- | --- | --- | --- | --- | --- | --- | --- | --- | --- | --- | --- | --- | --- | --- | --- | --- | --- | --- | --- | --- | --- | --- | --- | --- | --- | --- | --- | --- | --- | --- | --- | --- | --- | --- | --- | --- | --- | --- | --- | --- | --- | --- | --- | --- | --- | --- | --- | --- | --- | --- | --- | --- | --- | --- | --- | --- | --- | --- | --- | --- | --- | --- | --- | --- | --- | --- | --- | --- | --- | --- | --- | --- | --- | --- | --- | --- | --- | --- | --- | --- | --- | --- | --- | --- | --- | --- | --- | --- | --- | --- | --- | --- | --- | --- | --- | --- | --- | --- | --- | --- | --- | --- | --- | --- | --- | --- | --- | --- | --- | --- | --- | --- | --- | --- | --- | --- | --- | --- | --- | --- | --- | --- | --- | --- | --- | --- | --- | --- | --- | --- | --- | --- | --- | --- | --- | --- | --- | --- | --- | --- | --- | --- | --- | --- | --- | --- | --- | --- | --- | --- | --- | --- | --- | --- | --- | --- | --- | --- | --- | --- | --- | --- | --- | --- | --- | --- | --- | --- | --- | --- | --- | --- | --- | --- | --- | --- | --- | --- | --- | --- | --- | --- | --- | --- | --- | --- | --- | --- | --- | --- | --- | --- | --- | --- | --- | --- | --- | --- | --- | --- | --- | --- | --- | --- | --- | --- | --- | --- | --- | --- | --- | --- | --- | --- | --- | --- | --- | --- | --- | --- | --- | --- | --- | --- | --- | --- | --- | --- | --- | --- | --- | --- | --- | --- | --- | --- | --- | --- | --- | --- | --- | --- | --- | --- | --- | --- | --- | --- | --- | --- | --- | --- | --- | --- | --- | --- | --- | --- | --- | --- | --- | --- | --- | --- | --- | --- | --- | --- | --- | --- | --- | --- | --- | --- | --- | --- | --- | --- | --- | --- | --- | --- | --- | --- | --- | --- | --- | --- | --- | --- | --- | --- | --- | --- | --- | --- | --- | --- | --- | --- | --- | --- | --- | --- | --- | --- | --- | --- | --- | --- | --- | --- | --- | --- | --- | --- | --- | --- | --- | --- | --- | --- | --- | --- | --- | --- | --- | --- | --- | --- | --- | --- | --- | --- | --- | --- | --- | --- | --- | --- | --- | --- | --- | --- | --- | --- | --- | --- | --- | --- | --- | --- | --- | --- | --- | --- | --- | --- | --- | --- | --- | --- | --- | --- | --- | --- | --- | --- | --- | --- | --- | --- | --- | --- | --- | --- | --- | --- | --- | --- | --- | --- | --- | --- | --- | --- | --- | --- | --- | --- | --- | --- | --- | --- | --- | --- | --- | --- | --- | --- | --- | --- | --- | --- | --- | --- | --- | --- | --- | --- | --- | --- | --- | --- | --- | --- | --- | --- | --- | --- | --- | --- | --- | --- | --- | --- | --- | --- | --- | --- | --- | --- | --- | --- | --- | --- | --- | --- | --- | --- | --- | --- | --- | --- | --- | --- | --- | --- | --- | --- | --- | --- | --- | --- | --- | --- | --- | --- | --- | --- | --- | --- | --- | --- | --- | --- | --- | --- | --- | --- | --- | --- | --- | --- | --- | --- | --- | --- | --- | --- | --- | --- | --- | --- | --- | --- | --- | --- | --- | --- | --- | --- | --- | --- | --- | --- | --- | --- | --- | --- | --- | --- | --- | --- | --- | --- | --- | --- | --- | --- | --- | --- | --- | --- | --- | --- | --- | --- | --- | --- | --- | --- | --- | --- | --- | --- | --- | --- | --- | --- | --- | --- | --- | --- | --- | --- | --- | --- | --- | --- | --- | --- | --- | --- | --- | --- | --- | --- | --- | --- | --- | --- | --- | --- | --- | --- | --- | --- | --- | --- | --- | --- | --- | --- | --- | --- | --- | --- | --- | --- | --- | --- | --- | --- | --- | --- | --- | --- | --- | --- | --- | --- | --- | --- | --- | --- | --- | --- | --- | --- | --- | --- | --- | --- | --- | --- | --- | --- | --- | --- | --- | --- | --- | --- | --- | --- | --- | --- | --- | --- | --- | --- | --- | --- | --- | --- | --- | --- | --- | --- | --- | --- | --- | --- | --- | --- | --- | --- | --- | --- | --- | --- | --- | --- | --- | --- | --- | --- | --- | --- | --- | --- | --- | --- | --- | --- | --- | --- | --- | --- | --- | --- | --- | --- | --- | --- | --- | --- | --- | --- | --- | --- | --- | --- | --- | --- | --- | --- | --- | --- | --- | --- | --- | --- | --- | --- | --- | --- | --- | --- | --- | --- | --- | --- | --- | --- | --- | --- | --- | --- | --- | --- | --- | --- | --- | --- | --- | --- | --- | --- | --- | --- | --- | --- | --- | --- | --- | --- | --- | --- | --- | --- | --- | --- | --- | --- | --- | --- | --- | --- | --- | --- | --- | --- | --- | --- | --- | --- | --- | --- | --- | --- | --- | --- | --- | --- | --- | --- | --- | --- | --- | --- | --- | --- | --- | --- | --- | --- | --- | --- | --- | --- | --- | --- | --- | --- | --- | --- | --- | --- | --- | --- | --- | --- | --- | --- | --- | --- | --- | --- | --- | --- | --- | --- | --- | --- | --- | --- | --- | --- | --- | --- | --- | --- | --- | --- | --- | --- | --- | --- | --- | --- | --- | --- | --- | --- | --- | --- | --- | --- | --- | --- | --- | --- | --- | --- | --- | --- | --- | --- | --- | --- | --- | --- | --- | --- | --- | --- | --- | --- | --- | --- | --- | --- | --- | --- | --- | --- | --- | --- | --- | --- | --- | --- | --- | --- | --- | --- | --- | --- | --- | --- | --- | --- | --- | --- | --- | --- | --- | --- | --- | --- | --- | --- | --- | --- | --- | --- | --- | --- | --- | --- | --- | --- | --- | --- | --- | --- | --- | --- | --- | --- | --- | --- | --- | --- | --- | --- | --- | --- | --- | --- | --- | --- | --- | --- | --- | --- | --- | --- | --- | --- | --- | --- | --- | --- | --- | --- | --- | --- | --- | --- | --- | --- | --- | --- | --- | --- | --- | --- | --- | --- | --- | --- | --- | --- | --- | --- | --- | --- | --- | --- | --- | --- | --- | --- | --- | --- | --- | --- | --- | --- | --- | --- | --- | --- | --- | --- | --- | --- | --- | --- | --- | --- | --- | --- | --- | --- | --- | --- | --- | --- | --- | --- | --- | --- | --- | --- | --- | --- | --- | --- | --- | --- | --- | --- | --- | --- | --- | --- | --- | --- | --- | --- | --- | --- | --- | --- | --- | --- | --- | --- | --- | --- | --- | --- | --- | --- | --- | --- | --- | --- | --- | --- | --- | --- | --- | --- | --- | --- | --- | --- | --- | --- | --- | --- | --- | --- | --- | --- | --- | --- | --- | --- | --- | --- | --- | --- | --- | --- | --- | --- | --- | --- | --- | --- | --- | --- | --- | --- | --- | --- | --- | --- | --- | --- | --- | --- | --- | --- | --- | --- | --- | --- | --- | --- | --- | --- | --- | --- | --- | --- | --- | --- | --- | --- | --- | --- | --- | --- | --- | --- | --- | --- | --- | --- | --- | --- | --- | --- | --- | --- | --- | --- | --- | --- | --- | --- | --- | --- | --- | --- | --- | --- | --- | --- | --- | --- | --- | --- | --- | --- | --- | --- | --- | --- | --- | --- | --- | --- | --- | --- | --- | --- | --- | --- | --- | --- | --- | --- | --- | --- | --- | --- | --- | --- | --- | --- | --- | --- | --- | --- | --- | --- | --- | --- | --- | --- | --- | --- | --- | --- | --- | --- | --- | --- | --- | --- | --- | --- | --- | --- | --- | --- | --- | --- | --- | --- | --- | --- | --- | --- | --- | --- | --- | --- | --- | --- | --- | --- | --- | --- | --- | --- | --- | --- | --- | --- | --- | --- | --- | --- | --- | --- | --- | --- | --- | --- | --- | --- | --- | --- | --- | --- | --- | --- | --- | --- | --- | --- | --- | --- | --- | --- | --- | --- | --- | --- | --- | --- | --- | --- | --- | --- | --- | --- | --- | --- | --- | --- | --- | --- | --- | --- | --- | --- | --- | --- | --- | --- | --- | --- | --- | --- | --- | --- | --- | --- | --- | --- | --- | --- | --- | --- | --- | --- | --- | --- | --- | --- | --- | --- | --- | --- | --- | --- | --- | --- | --- | --- | --- | --- | --- | --- | --- | --- | --- | --- | --- | --- | --- | --- | --- | --- | --- | --- | --- | --- | --- | --- | --- | --- | --- | --- | --- | --- | --- | --- | --- | --- | --- | --- | --- | --- | --- | --- | --- | --- | --- | --- | --- | --- | --- | --- | --- | --- | --- | --- | --- | --- | --- | --- | --- | --- | --- | --- | --- | --- | --- | --- | --- | --- | --- | --- | --- | --- | --- | --- | --- | --- | --- | --- | --- | --- | --- | --- | --- | --- | --- | --- | --- | --- | --- | --- | --- | --- | --- | --- | --- | --- | --- | --- | --- | --- | --- | --- | --- | --- | --- | --- | --- | --- | --- | --- | --- | --- | --- | --- | --- | --- | --- | --- | --- | --- | --- | --- | --- | --- | --- | --- | --- | --- | --- | --- | --- | --- | --- | --- | --- | --- | --- | --- | --- | --- | --- | --- | --- | --- | --- | --- | --- | --- | --- | --- | --- | --- | --- | --- | --- | --- | --- | --- | --- | --- | --- | --- | --- | --- | --- | --- | --- | --- | --- | --- | --- | --- | --- | --- | --- | --- | --- | --- | --- | --- | --- | --- | --- | --- | --- | --- | --- | --- | --- | --- | --- | --- | --- | --- | --- | --- | --- | --- | --- | --- | --- | --- | --- | --- | --- | --- | --- | --- | --- | --- | --- | --- | --- | --- | --- | --- | --- | --- | --- | --- | --- | --- | --- | --- | --- | --- | --- | --- | --- | --- | --- | --- | --- | --- | --- | --- | --- | --- | --- | --- | --- | --- | --- | --- | --- | --- | --- | --- | --- | --- | --- | --- | --- | --- | --- | --- | --- | --- | --- | --- | --- | --- | --- | --- | --- | --- | --- | --- | --- | --- | --- | --- | --- | --- | --- | --- | --- | --- | --- | --- | --- | --- | --- | --- | --- | --- | --- | --- | --- | --- | --- | --- | --- | --- | --- | --- | --- | --- | --- | --- | --- | --- | --- | --- | --- | --- | --- | --- | --- | --- | --- | --- | --- | --- | --- | --- | --- | --- | --- | --- | --- | --- | --- | --- | --- | --- | --- | --- | --- | --- | --- | --- | --- | --- | --- | --- | --- | --- | --- | --- | --- | --- | --- | --- | --- | --- | --- | --- | --- | --- | --- | --- | --- | --- | --- | --- | --- | --- | --- | --- | --- | --- | --- | --- | --- | --- | --- | --- | --- | --- | --- | --- | --- | --- | --- | --- | --- | --- | --- | --- | --- | --- | --- | --- | --- | --- | --- | --- | --- | --- | --- | --- | --- | --- | --- | --- | --- | --- | --- | --- | --- | --- | --- | --- | --- | --- | --- | --- | --- | --- | --- | --- | --- | --- | --- | --- | --- | --- | --- | --- | --- | --- | --- | --- | --- | --- | --- | --- | --- | --- | --- | --- | --- | --- | --- | --- | --- | --- | --- | --- | --- | --- | --- | --- | --- | --- | --- | --- | --- | --- | --- | --- | --- | --- | --- | --- | --- | --- | --- | --- | --- | --- | --- | --- | --- | --- | --- | --- | --- | --- | --- | --- | --- | --- | --- | --- | --- | --- | --- | --- | --- | --- | --- | --- | --- | --- | --- | --- | --- | --- | --- | --- | --- | --- | --- | --- | --- | --- | --- | --- | --- | --- | --- | --- | --- | --- | --- | --- | --- | --- | --- | --- | --- | --- | --- | --- | --- | --- | --- | --- | --- | --- | --- | --- | --- | --- | --- | --- | --- | --- | --- | --- | --- | --- | --- | --- | --- | --- | --- | --- | --- | --- | --- | --- | --- | --- | --- | --- | --- | --- | --- | --- | --- | --- | --- | --- | --- | --- | --- | --- | --- | --- | --- | --- | --- | --- | --- | --- | --- | --- | --- | --- | --- | --- | --- | --- | --- | --- | --- | --- | --- | --- | --- | --- | --- | --- | --- | --- | --- | --- | --- | --- | --- | --- | --- | --- | --- | --- | --- | --- | --- | --- | --- | --- | --- | --- | --- | --- | --- | --- | --- | --- | --- | --- | --- | --- | --- | --- | --- | --- | --- | --- | --- | --- | --- | --- | --- | --- | --- | --- | --- | --- | --- | --- | --- | --- | --- | --- | --- | --- | --- | --- | --- | --- | --- | --- | --- | --- | --- | --- | --- | --- | --- | --- | --- | --- | --- | --- | --- | --- | --- | --- | --- | --- | --- | --- | --- | --- | --- | --- | --- | --- | --- | --- | --- | --- | --- | --- | --- | --- | --- | --- | --- | --- | --- | --- | --- | --- | --- | --- | --- | --- | --- | --- | --- | --- | --- | --- | --- | --- | --- | --- | --- | --- | --- | --- | --- | --- | --- | --- | --- | --- | --- | --- | --- | --- | --- | --- | --- | --- | --- | --- | --- | --- | --- | --- | --- | --- | --- | --- | --- | --- | --- | --- | --- | --- | --- | --- | --- | --- | --- | --- | --- | --- | --- | --- | --- | --- | --- | --- | --- | --- | --- | --- | --- | --- | --- | --- | --- | --- | --- | --- | --- | --- | --- | --- | --- | --- | --- | --- | --- | --- | --- | --- | --- | --- | --- | --- | --- | --- | --- | --- | --- | --- | --- | --- | --- | --- | --- | --- | --- | --- | --- | --- | --- | --- | --- | --- | --- | --- | --- | --- | --- | --- | --- | --- | --- | --- | --- | --- | --- | --- | --- | --- | --- | --- | --- | --- | --- | --- | --- | --- | --- | --- | --- | --- | --- | --- | --- | --- | --- | --- | --- | --- | --- | --- | --- | --- | --- | --- | --- | --- | --- | --- | --- | --- | --- | --- | --- | --- | --- | --- | --- | --- | --- | --- | --- | --- | --- | --- | --- | --- | --- | --- | --- | --- | --- | --- | --- | --- | --- | --- | --- | --- | --- | --- | --- | --- | --- | --- | --- | --- | --- | --- | --- | --- | --- | --- | --- | --- | --- | --- | --- | --- | --- | --- | --- | --- | --- | --- | --- | --- | --- | --- | --- | --- | --- | --- | --- | --- | --- | --- | --- | --- | --- | --- | --- | --- | --- | --- | --- | --- | --- | --- | --- | --- | --- | --- | --- | --- | --- | --- | --- | --- | --- | --- | --- | --- | --- | --- | --- | --- | --- | --- | --- | --- | --- | --- | --- | --- | --- | --- | --- | --- | --- | --- | --- | --- | --- | --- | --- | --- | --- | --- | --- | --- | --- | --- | --- | --- | --- | --- | --- | --- | --- | --- | --- | --- | --- | --- | --- | --- | --- | --- | --- | --- | --- | --- | --- | --- | --- | --- | --- | --- | --- | --- | --- | --- | --- | --- | --- | --- | --- | --- | --- | --- | --- | --- | --- | --- | --- | --- | --- | --- | --- | --- | --- | --- | --- | --- | --- | --- | --- | --- | --- | --- | --- | --- | --- | --- | --- | --- | --- | --- | --- | --- | --- | --- | --- | --- | --- | --- | --- | --- | --- | --- | --- | --- | --- | --- | --- | --- | --- | --- | --- | --- | --- | --- | --- | --- | --- | --- | --- | --- | --- | --- | --- | --- | --- | --- | --- | --- | --- | --- | --- | --- | --- | --- | --- | --- | --- | --- | --- | --- | --- | --- | --- | --- | --- | --- | --- | --- | --- | --- | --- | --- | --- | --- | --- | --- | --- | --- | --- | --- | --- | --- | --- | --- | --- | --- | --- | --- | --- | --- | --- | --- | --- | --- | --- | --- | --- | --- | --- | --- | --- | --- | --- | --- | --- | --- | --- | --- | --- | --- | --- | --- | --- | --- | --- | --- | --- | --- | --- | --- | --- | --- | --- | --- | --- | --- | --- | --- | --- | --- | --- | --- | --- | --- | --- | --- | --- | --- | --- | --- | --- | --- | --- | --- | --- | --- | --- | --- | --- | --- | --- | --- | --- | --- | --- | --- | --- | --- | --- | --- | --- | --- | --- | --- | --- | --- | --- | --- | --- | --- | --- | --- | --- | --- | --- | --- | --- | --- | --- | --- | --- | --- | --- | --- | --- | --- | --- | --- | --- | --- | --- | --- | --- | --- | --- | --- | --- | --- | --- | --- | --- | --- | --- | --- | --- | --- | --- | --- | --- | --- | --- | --- | --- | --- | --- | --- | --- | --- | --- | --- | --- | --- | --- | --- | --- | --- | --- | --- | --- | --- | --- | --- | --- | --- | --- | --- | --- | --- | --- | --- | --- | --- | --- | --- | --- | --- | --- | --- | --- | --- | --- | --- | --- | --- | --- | --- | --- | --- | --- | --- | --- | --- | --- | --- | --- | --- | --- | --- | --- | --- | --- | --- | --- | --- | --- | --- | --- | --- | --- | --- | --- | --- | --- | --- | --- | --- | --- | --- | --- | --- | --- | --- | --- | --- | --- | --- | --- | --- | --- | --- | --- | --- | --- | --- | --- | --- | --- | --- | --- | --- | --- | --- | --- | --- | --- | --- | --- | --- | --- | --- | --- | --- | --- | --- | --- | --- | --- | --- | --- | --- | --- | --- | --- | --- | --- | --- | --- | --- | --- | --- | --- | --- | --- | --- | --- | --- | --- | --- | --- | --- | --- | --- | --- | --- | --- | --- | --- | --- | --- | --- | --- | --- | --- | --- | --- | --- | --- | --- | --- | --- | --- | --- | --- | --- | --- | --- | --- | --- | --- | --- | --- | --- | --- | --- | --- | --- | --- | --- | --- | --- | --- | --- | --- | --- | --- | --- | --- | --- | --- | --- | --- | --- | --- | --- | --- | --- | --- | --- | --- | --- | --- | --- | --- | --- | --- | --- | --- | --- | --- | --- | --- | --- | --- | --- | --- | --- | --- | --- | --- | --- | --- | --- | --- | --- | --- | --- | --- | --- | --- | --- | --- | --- | --- | --- | --- | --- | --- | --- | --- | --- | --- | --- | --- | --- | --- | --- | --- | --- | --- | --- | --- | --- | --- | --- | --- | --- | --- | --- | --- | --- | --- | --- | --- | --- | --- | --- | --- | --- | --- | --- | --- | --- | --- | --- | --- | --- | --- | --- | --- | --- | --- | --- | --- | --- | --- | --- | --- | --- | --- | --- | --- | --- | --- | --- | --- | --- | --- | --- | --- | --- | --- | --- | --- | --- | --- | --- | --- | --- | --- | --- | --- | --- | --- | --- | --- | --- | --- | --- | --- | --- | --- | --- | --- | --- | --- | --- | --- | --- | --- | --- | --- | --- | --- | --- | --- | --- | --- | --- | --- | --- | --- | --- | --- | --- | --- | --- | --- | --- | --- | --- | --- | --- | --- | --- | --- | --- | --- | --- | --- | --- | --- | --- | --- | --- | --- | --- | --- | --- | --- | --- | --- | --- | --- | --- | --- | --- | --- | --- | --- | --- | --- | --- | --- | --- | --- | --- | --- | --- | --- | --- | --- | --- | --- | --- | --- | --- | --- | --- | --- | --- | --- | --- | --- | --- | --- | --- | --- | --- | --- | --- | --- | --- | --- | --- | --- | --- | --- | --- | --- | --- | --- | --- | --- | --- | --- | --- | --- | --- | --- | --- | --- | --- | --- | --- | --- | --- | --- | --- | --- | --- | --- | --- | --- | --- | --- | --- | --- | --- | --- | --- | --- | --- | --- | --- | --- | --- | --- | --- | --- | --- | --- | --- | --- | --- | --- | --- | --- | --- | --- | --- | --- | --- | --- | --- | --- | --- | --- | --- | --- | --- | --- | --- | --- | --- | --- | --- | --- | --- | --- | --- | --- | --- | --- | --- | --- | --- | --- | --- | --- | --- | --- | --- | --- | --- | --- | --- | --- | --- | --- | --- | --- | --- | --- | --- | --- | --- | --- | --- | --- | --- | --- | --- | --- | --- | --- | --- | --- | --- | --- | --- | --- | --- | --- | --- | --- | --- | --- | --- | --- | --- | --- | --- | --- | --- | --- | --- | --- | --- | --- | --- | --- | --- | --- | --- | --- | --- | --- | --- | --- | --- | --- | --- | --- | --- | --- | --- | --- | --- | --- | --- | --- | --- | --- | --- | --- | --- | --- | --- | --- | --- | --- | --- | --- | --- | --- | --- | --- | --- | --- | --- | --- | --- | --- | --- | --- | --- | --- | --- | --- | --- | --- | --- | --- | --- | --- | --- | --- | --- | --- | --- | --- | --- | --- | --- | --- | --- | --- | --- | --- | --- | --- | --- | --- | --- | --- | --- | --- | --- | --- | --- | --- | --- | --- | --- | --- | --- | --- | --- | --- | --- | --- | --- | --- | --- | --- | --- | --- | --- | --- | --- | --- | --- | --- | --- | --- | --- | --- | --- | --- | --- | --- | --- | --- | --- | --- | --- | --- | --- | --- | --- | --- | --- | --- | --- | --- | --- | --- | --- | --- | --- | --- | --- | --- | --- | --- | --- | --- | --- | --- | --- | --- | --- | --- | --- | --- | --- | --- | --- | --- | --- | --- | --- | --- | --- | --- | --- | --- | --- | --- | --- | --- | --- | --- | --- | --- | --- | --- | --- | --- | --- | --- | --- | --- | --- | --- | --- | --- | --- | --- | --- | --- | --- | --- | --- | --- | --- | --- | --- | --- | --- | --- | --- | --- | --- | --- | --- | --- | --- | --- | --- | --- | --- | --- | --- | --- | --- | --- | --- | --- | --- | --- | --- | --- | --- | --- | --- | --- | --- | --- | --- | --- | --- | --- | --- | --- | --- | --- | --- | --- | --- | --- | --- | --- | --- | --- | --- | --- | --- | --- | --- | --- | --- | --- | --- | --- | --- | --- | --- | --- | --- | --- | --- | --- | --- | --- | --- | --- | --- | --- | --- | --- | --- | --- | --- | --- | --- | --- | --- | --- | --- | --- | --- | --- | --- | --- | --- | --- | --- | --- | --- | --- | --- | --- | --- | --- | --- | --- | --- | --- | --- | --- | --- | --- | --- | --- | --- | --- | --- | --- | --- | --- | --- | --- | --- | --- | --- | --- | --- | --- | --- | --- | --- | --- | --- | --- | --- | --- | --- | --- | --- | --- | --- | --- | --- | --- | --- | --- | --- | --- | --- | --- | --- | --- | --- | --- | --- | --- | --- | --- | --- | --- | --- | --- | --- | --- | --- | --- | --- | --- | --- | --- | --- | --- | --- | --- | --- | --- | --- | --- | --- | --- | --- | --- | --- | --- | --- | --- | --- | --- | --- | --- | --- | --- | --- | --- | --- | --- | --- | --- | --- | --- | --- | --- | --- | --- | --- | --- | --- | --- | --- | --- | --- | --- | --- | --- | --- | --- | --- | --- | --- | --- | --- | --- | --- | --- | --- | --- | --- | --- | --- | --- | --- | --- | --- | --- | --- | --- | --- | --- | --- | --- | --- | --- | --- | --- | --- | --- | --- | --- | --- | --- | --- | --- | --- | --- | --- | --- | --- | --- | --- | --- | --- | --- | --- | --- | --- | --- | --- | --- | --- | --- | --- | --- | --- | --- | --- | --- | --- | --- | --- | --- | --- | --- | --- | --- | --- | --- | --- | --- | --- | --- | --- | --- | --- | --- | --- | --- | --- | --- | --- | --- | --- | --- | --- | --- | --- | --- | --- | --- | --- | --- | --- | --- | --- | --- | --- | --- | --- | --- | --- | --- | --- | --- | --- | --- | --- | --- | --- | --- | --- | --- | --- | --- | --- | --- | --- | --- | --- | --- | --- | --- | --- | --- | --- | --- | --- | --- | --- | --- | --- | --- | --- | --- | --- | --- | --- | --- | --- | --- | --- | --- | --- | --- | --- | --- | --- | --- | --- | --- | --- | --- | --- | --- | --- | --- | --- | --- | --- | --- | --- | --- | --- | --- | --- | --- | --- | --- | --- | --- | --- | --- | --- | --- | --- | --- | --- | --- | --- | --- | --- | --- | --- | --- | --- | --- | --- | --- | --- | --- | --- | --- | --- | --- | --- | --- | --- | --- | --- | --- | --- | --- | --- | --- | --- | --- | --- | --- | --- | --- | --- | --- | --- | --- | --- | --- | --- | --- | --- | --- | --- | --- | --- | --- | --- | --- | --- | --- | --- | --- | --- | --- | --- | --- | --- | --- | --- | --- | --- | --- | --- | --- | --- | --- | --- | --- | --- | --- | --- | --- | --- | --- | --- | --- | --- | --- | --- | --- | --- | --- | --- | --- | --- | --- | --- | --- | --- | --- | --- | --- | --- | --- | --- | --- | --- | --- | --- | --- | --- | --- | --- | --- | --- | --- | --- | --- | --- | --- | --- | --- | --- | --- | --- | --- | --- | --- | --- | --- | --- | --- | --- | --- | --- | --- | --- | --- | --- | --- | --- | --- | --- | --- | --- | --- | --- | --- | --- | --- | --- | --- | --- | --- | --- | --- | --- | --- | --- | --- | --- | --- | --- | --- | --- | --- | --- | --- | --- | --- | --- | --- | --- | --- | --- | --- | --- | --- | --- | --- | --- | --- | --- | --- | --- | --- | --- | --- | --- | --- | --- | --- | --- | --- | --- | --- | --- | --- | --- | --- | --- | --- | --- | --- | --- | --- | --- | --- | --- | --- | --- | --- | --- | --- | --- | --- | --- | --- | --- | --- | --- | --- | --- | --- | --- | --- | --- | --- | --- | --- | --- | --- | --- | --- | --- | --- | --- | --- | --- | --- | --- | --- | --- | --- | --- | --- | --- | --- | --- | --- | --- | --- | --- | --- | --- | --- | --- | --- | --- | --- | --- | --- | --- | --- | --- | --- | --- | --- | --- | --- | --- | --- | --- | --- | --- | --- | --- | --- | --- | --- | --- | --- | --- | --- | --- | --- | --- | --- | --- | --- | --- | --- | --- | --- | --- | --- | --- | --- | --- | --- | --- | --- | --- | --- | --- | --- | --- | --- | --- | --- | --- | --- | --- | --- | --- | --- | --- | --- | --- | --- | --- | --- | --- | --- | --- | --- | --- | --- | --- | --- | --- | --- | --- | --- | --- | --- | --- | --- | --- | --- | --- | --- | --- | --- | --- | --- | --- | --- | --- | --- | --- | --- | --- | --- | --- | --- | --- | --- | --- | --- | --- | --- | --- | --- | --- | --- | --- | --- | --- | --- | --- | --- | --- | --- | --- | --- | --- | --- | --- | --- | --- | --- | --- | --- | --- | --- | --- | --- | --- | --- | --- | --- | --- | --- | --- | --- | --- | --- | --- | --- | --- | --- | --- | --- | --- | --- | --- | --- | --- | --- | --- | --- | --- | --- | --- | --- | --- | --- | --- | --- | --- | --- | --- | --- | --- | --- | --- | --- | --- | --- | --- | --- | --- | --- | --- | --- | --- | --- | --- | --- | --- | --- | --- | --- | --- | --- | --- | --- | --- | --- | --- | --- | --- | --- | --- | --- | --- | --- | --- | --- | --- | --- | --- | --- | --- | --- | --- | --- | --- | --- | --- | --- | --- | --- | --- | --- | --- | --- | --- | --- | --- | --- | --- | --- | --- | --- | --- | --- | --- | --- | --- | --- | --- | --- | --- | --- | --- | --- | --- | --- | --- | --- | --- | --- | --- | --- | --- | --- | --- | --- | --- | --- | --- | --- | --- | --- | --- | --- | --- | --- | --- | --- | --- | --- | --- | --- | --- | --- | --- | --- | --- | --- | --- | --- | --- | --- | --- | --- | --- | --- | --- | --- | --- | --- | --- | --- | --- | --- | --- | --- | --- | --- | --- | --- | --- | --- | --- | --- | --- | --- | --- | --- | --- | --- | --- | --- | --- | --- | --- | --- | --- | --- | --- | --- | --- | --- | --- | --- | --- | --- | --- | --- | --- | --- | --- | --- | --- | --- | --- | --- | --- | --- | --- | --- | --- | --- | --- | --- | --- | --- | --- | --- | --- | --- | --- | --- | --- | --- | --- | --- | --- | --- | --- | --- | --- | --- | --- | --- | --- | --- | --- | --- | --- | --- | --- | --- | --- | --- | --- | --- | --- | --- | --- | --- | --- | --- | --- | --- | --- | --- | --- | --- | --- | --- | --- | --- | --- | --- | --- | --- | --- | --- | --- | --- | --- | --- | --- | --- | --- | --- | --- | --- | --- | --- | --- | --- | --- | --- | --- | --- | --- | --- | --- | --- | --- | --- | --- | --- | --- | --- | --- | --- | --- | --- | --- | --- | --- | --- | --- | --- | --- | --- | --- | --- | --- | --- | --- | --- | --- | --- | --- | --- | --- | --- | --- | --- | --- | --- | --- | --- | --- | --- | --- | --- | --- | --- | --- | --- | --- | --- | --- | --- | --- | --- | --- | --- | --- | --- | --- | --- | --- | --- | --- | --- | --- | --- | --- | --- | --- | --- | --- | --- | --- | --- | --- | --- | --- | --- | --- | --- | --- | --- | --- | --- | --- | --- | --- | --- | --- | --- | --- | --- | --- | --- | --- | --- | --- | --- | --- | --- | --- | --- | --- | --- | --- | --- | --- | --- | --- | --- | --- | --- | --- | --- | --- | --- | --- | --- | --- | --- | --- | --- | --- | --- | --- | --- | --- | --- | --- | --- | --- | --- | --- | --- | --- | --- | --- | --- | --- | --- | --- | --- | --- | --- | --- | --- | --- | --- | --- | --- | --- | --- | --- | --- | --- | --- | --- | --- | --- | --- | --- | --- | --- | --- | --- | --- | --- | --- |
