# Supplementary material for: Morpho-biometric characterization of indigenous chicken ecotypes in north-western Ethiopia
Source: PLoS One. 2023 Jun 2;18(6):e0286299. doi: 10.1371/journal.pone.0286299 (PMC10237646; doi:10.1371/journal.pone.0286299)
Supplement: S6 File — (DOCX) [file pone.0286299.s006.docx]

setwd("E:/my data with R")

library(readr)

BOTH4T <- read_csv("E:/my data with R/DATA/4cluster/BOTH4T.csv")

View(BOTH)

Both4fm <- scale(BOTH)

res.dist <- dist(Both4fm, method = "euclidean")

as.matrix(res.dist)[1:6, 1:6]

HC <- hclust(d = res.dist, method = "ward.D2")

library("factoextra")

fviz_dend(HC, cex = 0.6)

plot(HC, cex = 0.6)

rect.hclust(HC, k = 4, border = 2:5)

library(readr)

F6T <- read_csv("E:/my data with R/DATA/4cluster/F6T.csv")

View(F6T)

HCPC(F6T, nb.clust = -1, min = 4, max = NULL, graph = TRUE)

library(FactoMineR)

# Compute PCA with ncp = 3

res.pca <- PCA(F6T, ncp = 3, graph = FALSE)

# Compute hierarchical clustering on principal components

res.hcpc <- HCPC(res.pca, graph = FALSE)

fviz_dend(res.hcpc,

cex = 0.7, # Label size

palette = "jco", # Color palette see ?ggpubr::ggpar

rect = TRUE, rect_fill = TRUE, # Add rectangle around groups

rect_border = "jco", # Rectangle color

labels_track_height = 0.8 # Augment the room for labels)

fviz_cluster(res.hcpc,

repel = TRUE, # Avoid label overlapping

show.clust.cent = TRUE, # Show cluster centers

palette = "jco", # Color palette see ?ggpubr::ggpar

ggtheme = theme_minimal(),

main = "Factor map")

# Principal components + tree

plot(res.hcpc, choice = "3D.map")

##HCPC#MALE#6 QUAN TRAITS WITH HIGH DISCRIMINATING POWER#

library(readr)

M6T <- read_csv("E:/my data with R/DATA/4cluster/M6T.csv")

View(M6T)

HCPC(M6T, nb.clust = 0, min = 4, max = NULL, graph = TRUE)

library(FactoMineR)

# Compute PCA with ncp = 4

res.pca <- PCA(M6T, ncp = 4, graph = FALSE)

# Compute hierarchical clustering on principal components

res.hcpc <- HCPC(res.pca, graph = FALSE)

fviz_dend(res.hcpc,

cex = 0.7, # Label size

palette = "jco", # Color palette see ?ggpubr::ggpar

rect = TRUE, rect_fill = TRUE, # Add rectangle around groups

rect_border = "jco", # Rectangle color

labels_track_height = 0.8 # Augment the room for labels

)

fviz_cluster(res.hcpc,

repel = TRUE, # Avoid label overlapping

show.clust.cent = TRUE, # Show cluster centers

palette = "jco", # Color palette see ?ggpubr::ggpar

ggtheme = theme_minimal(),

main = "Factor map"

)

# Principal components + tree

plot(res.hcpc, choice = "3D.map")
